# Antiproliferative and Tubulin-Destabilising Effects of 3-(Prop-1-en-2-yl)azetidin-2-Ones and Related Compounds in MCF-7 and MDA-MB-231 Breast Cancer Cells

**DOI:** 10.3390/ph16071000

**Published:** 2023-07-13

**Authors:** Shu Wang, Azizah M. Malebari, Thomas F. Greene, Shubhangi Kandwal, Darren Fayne, Seema M. Nathwani, Daniela M. Zisterer, Brendan Twamley, Niamh M. O’Boyle, Mary J. Meegan

**Affiliations:** 1 School of Pharmacy and Pharmaceutical Sciences, Trinity Biomedical Sciences Institute, Trinity College Dublin, 152-160 Pearse Street, Dublin 2, D02 R590 Dublin, Ireland; 2 Department of Pharmaceutical Chemistry, College of Pharmacy, King Abdulaziz University, Jeddah 21589, Saudi Arabia; 3 Molecular Design Group, School of Biochemistry and Immunology, Trinity Biomedical Sciences Institute, Trinity College Dublin, 152-160 Pearse Street, Dublin 2, D02 R590 Dublin, Ireland; 4 School of Biochemistry and Immunology, Trinity Biomedical Sciences Institute, Trinity College Dublin, 152-160 Pearse Street, Dublin 2, D02 R590 Dublin, Ireland; 5 School of Chemistry, Trinity College Dublin, Dublin 2, D02 PN40 Dublin, Ireland

**Keywords:** combretastatin A-4, 3-(prop-1-en-2-yl)azetidin-2-one, 3-(buta-1,3-dien-1-yl)azetidin-2-ones, 3-allylazetidin-2-ones, β-lactam, antiproliferative activity, tubulin polymerization inhibitor, antimitotic

## Abstract

A series of novel 3-(prop-1-en-2-yl)azetidin-2-one, 3-allylazetidin-2-one and 3-(buta-1,3-dien-1-yl)azetidin-2-one analogues of combretastatin A-4 (CA-4) were designed and synthesised as colchicine-binding site inhibitors (CBSI) in which the ethylene bridge of CA-4 was replaced with a β-lactam (2-azetidinone) scaffold. These compounds, together with related prodrugs, were evaluated for their antiproliferative activity, cell cycle effects and ability to inhibit tubulin assembly. The compounds demonstrated significant in vitro antiproliferative activities in MCF-7 breast cancer cells, particularly for compounds **9h, 9q, 9r, 10p, 10r** and **11h**, with IC_50_ values in the range 10–33 nM. These compounds were also potent in the triple-negative breast cancer (TBNC) cell line MDA-MB-231, with IC_50_ values in the range 23–33 nM, and were comparable with the activity of CA-4. The compounds inhibited the polymerisation of tubulin in vitro, with significant reduction in tubulin polymerization, and were shown to interact at the colchicine-binding site on tubulin. Flow cytometry demonstrated that compound **9q** arrested MCF-7 cells in the G_2_/M phase and resulted in cellular apoptosis. The antimitotic properties of **9q** in MCF-7 human breast cancer cells were also evaluated, and the effect on the organization of microtubules in the cells after treatment with compound **9q** was observed using confocal microscopy. The immunofluorescence results confirm that β-lactam **9q** is targeting tubulin and resulted in mitotic catastrophe in MCF-7 cells. In silico molecular docking supports the hypothesis that the compounds interact with the colchicine-binding domain of tubulin. Compound **9q** is a novel potent microtubule-destabilising agent with potential as a promising lead compound for the development of new antitumour agents.

## 1. Introduction

Breast cancer is the most commonly reported cancer among women in developed countries. [1]. Over the last decade, the clinical treatment options for breast cancer patients have significantly improved with the approval of multiple drugs for various indications [2,3]. The hormone receptor (HR) positive/human epidermal growth factor receptor 2 (HER2)-negative subtype is the most commonly identified subtype and is found in approximately 70% of breast cancers diagnosed. Endocrine therapies (tamoxifen, fulvestrant, anastrozole, exemestane and letrozole) are conventionally used for treatment of this HR+/HER2- breast cancer subtype. Targeted therapies such as CDK4/6 inhibitors palbociclib, ribociclib and abemaciclib [4] in combination with endocrine therapies show improved therapeutic effects in HR+/HER2- and metastatic breast cancer (MBC) [5]. Alpelisib, a PI3K inhibitor, is approved for patients with HR+, HER2- and PIK3CA-mutated cancers in combination with fulvestrant [6], while the PARP inhibitor olaparib targets BRCA mutations in early breast cancer [7]. Recently approved breast cancer drugs include the antibody–drug conjugate (ADC) trastuzumab deruxtecan [8] and the estrogen receptor degrader (SERD) elacestrant [9].

**Figure 1:**
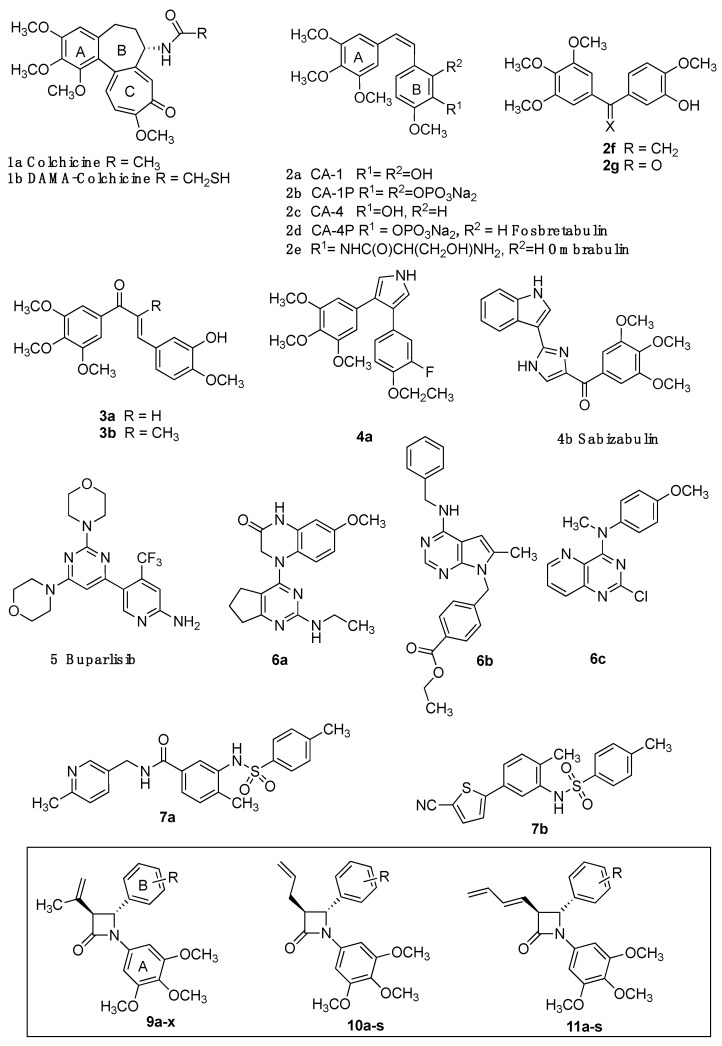
Small molecules targeting the colchicine-binding site of tubulin. Colchicine **1a**, DAMA-colchicine **1b,** combretastatins **2a**-**c**, fosbretabulin **2d**, ombrabulin **2e,** isocombretastatin **2f**, phenstatin **2g**, chalcones **3a**, **3b**; colchicine-binding site inhibitors **4a**, sabizabulin **4b,** buparlisib **5,** dihydroquinoxalinone **6a,** pyrrolopyrimidine **6b,** pyridopyrimidine **6c**, sulfonamides **7a, 7b** and β-lactam target structures **9a**-**x, 10a**-**s, 11a**-**s**.

Microtubule-targeting agents (MTAs) such as paclitaxel and docetaxel and the vinca alkaloid vinorelbine are widely used in breast cancer chemotherapy. [10,11] MTAs interact with tubulin at eight major binding sites: the binding sites for taxane and vinca alkaloid [12], gatorbulin [13], laulimalide/peloruside [14], maytansine [15], pironetin [16], cevipabulin [17] and colchicine **1a** (Figure 1) [18]. Eribulin, a potent inhibitor of microtubule dynamics, is used to treat refractory breast cancers [19,20]. Although colchicine (**1a**) inhibits mitosis, the clinical value of the drug is limited by its low therapeutic index (Figure 1) [21]. Many colchicine-type binding site inhibitors (CBSI) act as angiogenesis inhibitors and vasculature-disrupting agents (VDAs). Combretastatins CA-1 (**2a**) and CA-4 (**2c**) and structural analogues demonstrate potent cytotoxicity against human cancer cells (Figure 1) [22]. The water-soluble CA-1 diphosphate (**2b**), CA-4 phosphate (**2d**), CA-4 serine prodrug ombrabulin (**2e**) [23] and CA-4 phosphoramidate [24] have been explored as novel anticancer prodrugs.

Isocombretastatin **2f** [25], dihydronaphthalene OXi6196 [26,27], phenstatin **2g** [28] and chalcones **3a** and **3b** display comparable biological activity to CA-4 [29]; however, poor water solubility and *cis/trans* isomerization of combretastatins remain problematic [30]. CA-4 analogues are reported in which a heterocycle replaces the olefinic bond of CA-4 and prevents *cis/trans* isomerization [22,31,32,33]. The pyrrole **4a** induces significant apoptosis [34], while the *bis*-indole sabizabulin **4b** is effective in metastatic breast [35,36] and prostate cancers [37]. The 4-aryl-4*H*-chromene crolibulin [38], diketopiperazine plinabulin [39] and pyrimidine PI3K inhibitor buparlisib **5** inhibit tubulin polymerization, with promising activity in metastatic triple-negative breast cancer (TNBC) (Figure 1) [40].

Among recently reported colchicine-binding site inhibitors are dihydroquinoxalinone **6a** (SB226) [41], pyrrolopyrimidine **6b** [42] and sulfonamides **7a** and **7b** [43,44]. Interestingly **5**, **6a**, **6b**, **7a** and **7b**, together with the recently reported quinazoline **6c** [45], lack the characteristic 3,4,5-trimethoxyaryl pharmacophore.

CA-4 analogues containing the β-lactam heterocycle have been reported [46,47,48], while chiral azetidin-2-ones disrupt tubulin polymerization and suppress angiogenesis [49,50,51]. We previously reported the antimitotic properties of novel β-lactam analogues of CA-4, which were synthesised using optimised Staudinger and Reformatsky chemistry, with preferred *trans* stereochemistry for the C3 and C4 ring substituents [52,53,54]. These β-lactam compounds are distinguished by introduction of the 3,4,5-trimethoxyphenyl ring A (as in CA-4), required for activity, a β-lactam ring as the linking group to replace the double bond of CA-4 (thus preventing *E/Z* isomerisation in aqueous conditions) and a substituted aryl ring at C-4 as ring B.

To progress our understanding of effects of C-3 substitution on the antiproliferative activity of these β-lactam compounds, we synthesised a series of azetidin-2-ones having prop-1-en-2-yl, allyl and buta-1,3-dien-1-yl substituents at C3 of the azetidin-2-one ring (see Figure 1 target structures). The cytotoxic effects of the novel compounds and prodrugs in ER+ human MCF-7 breast cancer cells and triple-negative MDA-MB-231 breast cancer cells were determined together with their pro-apoptotic and tubulin-targeting effects.

## 2. Results and Discussion

### 2.1. Chemistry: Synthesis of β-Lactams

**Scheme 1:**
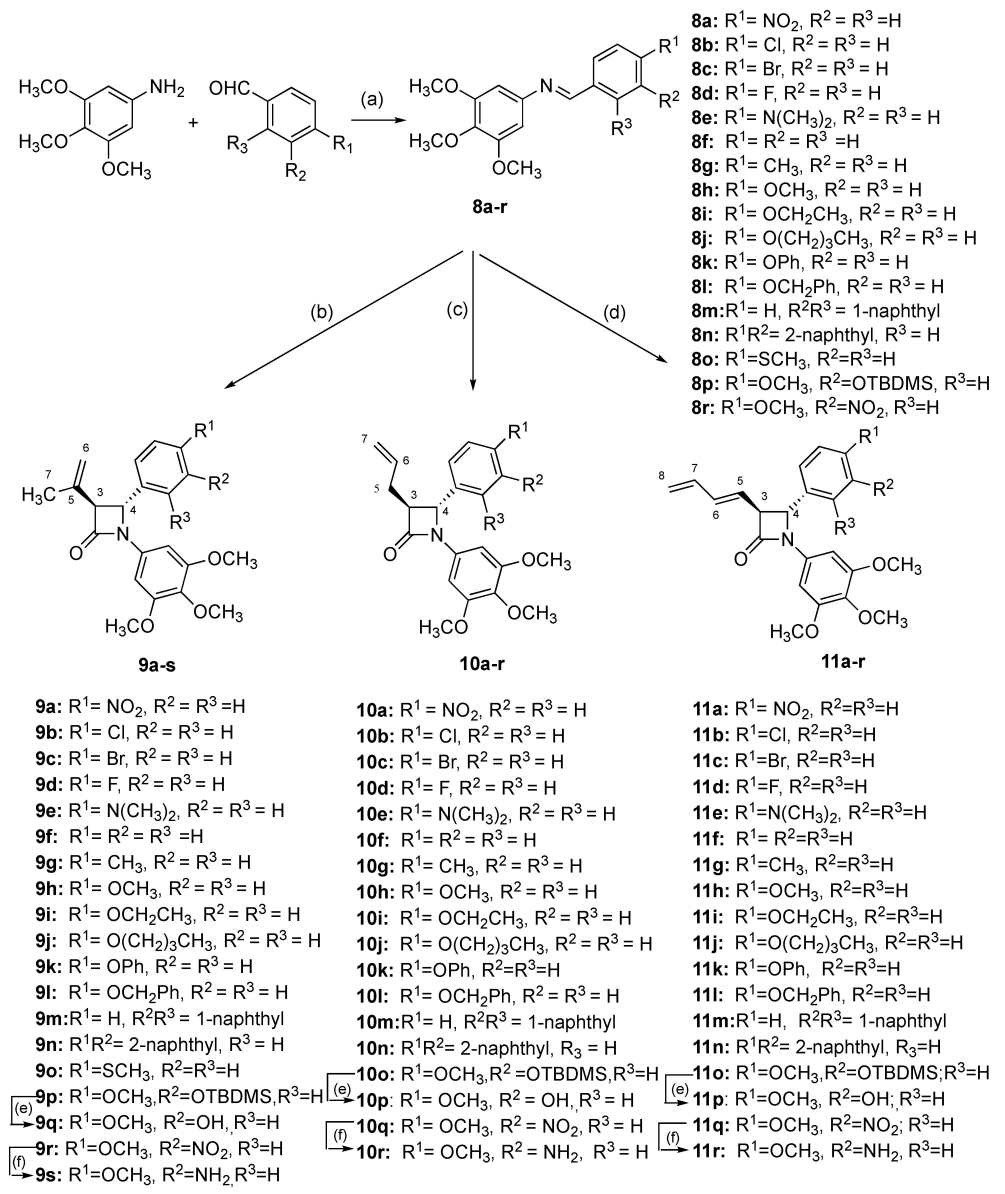
Synthesis of β-lactam compounds **9a**-**s**, **10a**-**r**, **11a**-**r**. Scheme reagents and conditions: (**a**) EtOH, conc. H_2_SO_4_, reflux, 4 h; (**b**) 3,3-dimethylacryloyl chloride, triethylamine, dry DCM, reflux, 5 h, N_2_ (7–51%); (**c**) 4-pentenoyl chloride, triethylamine, dry DCM, reflux, 5 h, N_2_ (11–96%); (**d**) (i) sorbic acid, tripropylamine, dry DCM, 2-chloro-1-methylpyridinium iodide, reflux, 12 h, N_2_; (ii) imine, DCM, 12 h reflux (16–59%); (**e**) TBAF, dry THF, 0 °C, 30 min, N_2_ (22–37%); (**f**) Zn dust, acetic acid, 7 days, N_2_, 20 °C (48–96%). Products obtained as a mixture of enantiomers; one enantiomer illustrated.

**Scheme 2:**
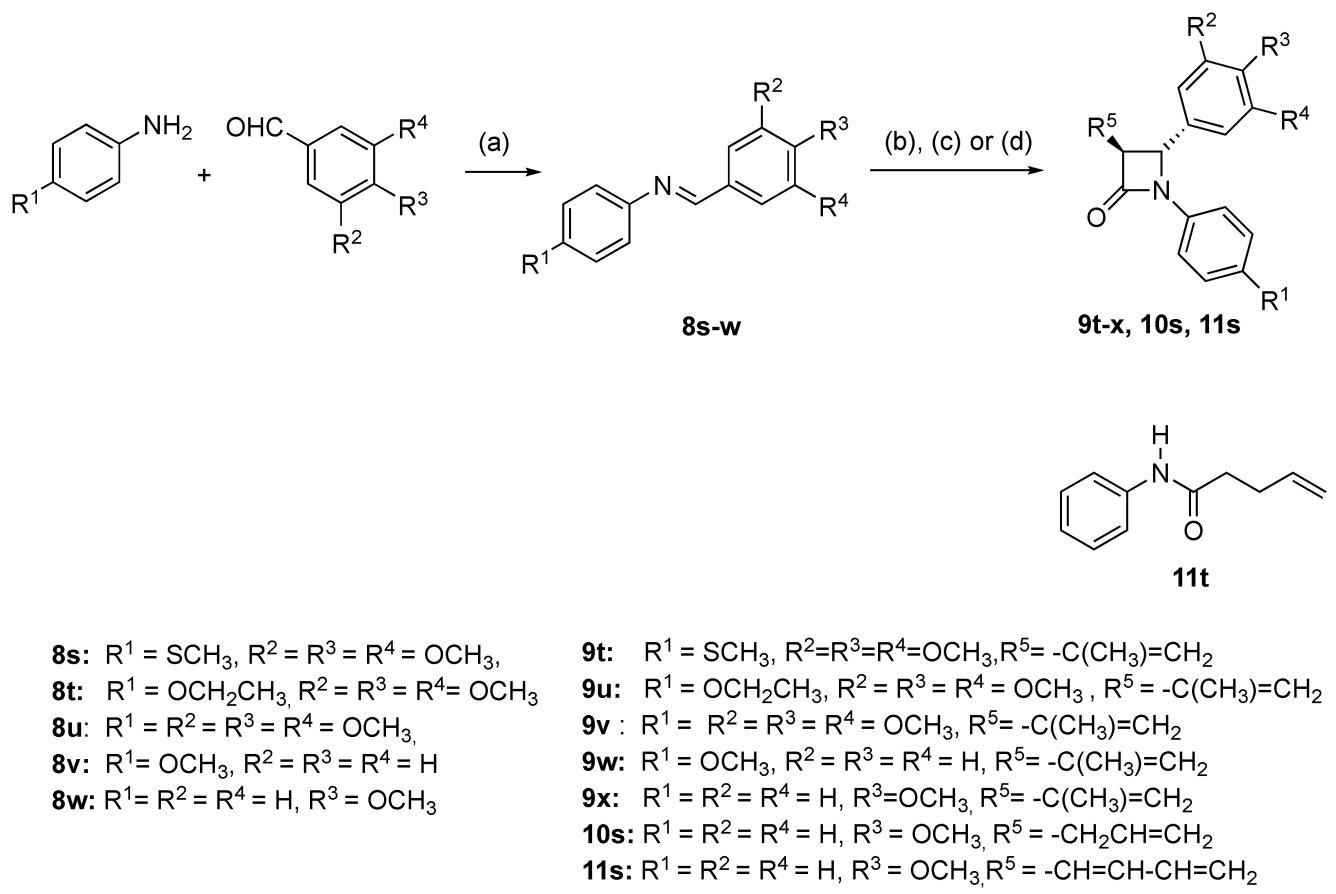
Synthesis of compounds **9t**-**x**, **10s**, **11s**, **11t**. Reagents and conditions: (**a**) EtOH, reflux, 4-5 h. (**b**) For compounds **9t**-**9x**: 3,3-dimethylacryloyl chloride, triethylamine, dry DCM, reflux, 5 h, N_2_ (4.6–45%). (**c**) For compound **10s:** 4-pentenoyl chloride, triethylamine, dry DCM, reflux, 5 h, N_2_ (40%). (**d**) For compound **11s, 11t:** (i) sorbic acid, tripropylamine, dry DCM, 2-chloro-1-methylpyridinium iodide, reflux, 12 h, N_2_; (ii) imine, DCM, 12 h reflux (45%). β-lactam products obtained as a mixture of enantiomers; one enantiomer illustrated.

β-Lactams are the most widely used group of antibiotic drugs for bacterial infections [55]; β-lactam-containing molecules are also useful as intermediates in organic synthesis, and many synthetic routes are available for the construction of the β-lactam ring [56,57,58], including the recently reported carbonylative formal cycloaddition of alkylarenes with imines [59]. In the present work, the required series of 3-isopropenyl β-lactams with a variety of ring B aryl substituents located at C-4 of the β-lactam ring were obtained by Staudinger reaction of imines with 3,3-dimethylacryloyl chloride. Schiff bases **8a**-**8r** were prepared by the condensation of the appropriately substituted arylaldehyde with the 3,4,5-trimethoxyaniline catalysed by sulphuric acid (Scheme 1). The imines contained the C-4 aryl ring B substituents e.g., nitro (**8a**), halogen (**8b**-**8d**), alkyl (**8g**), amino (**8e**), ethers (**8h**-**8l, 8p**), thioether (**8o**) and naphthyl (**8m** and **8n**). The Schiff bases (**8s**-**8u**) where ring B is located at C-1 of the imine were obtained by reaction of 3,4,5-trimethoxybenzaldehyde with appropriate anilines (Scheme 2), together with additional imines **8v**-**w**. The 3,4,5-trimethoxy-substituted A-ring of CA-4 provides important interactions with the colchicine-binding site residues of tubulin [30]. The crystal structures of the imines **8h** and **8i** demonstrated the *E* configuration of the imines (Supplementary Materials Tables S1 and S13).

The novel 3-(prop-1-en-2-yl)azetidin-2-ones (**9a**-**p, 9r**) were obtained by reaction of imines **8a**-**8r** with 3,3-dimethylacryloyl chloride using the Staudinger reaction conditions with triethylamine as the base [60,61,62] (Scheme 1; Scheme 2). The β-lactam compounds (**9t**-**v)**, were also prepared containing the 3,4,5-trimethoxyphenyl substituent (ring A) at the C-4 position, together with structurally related β-lactams **9w** and **9x** (Scheme 2). All products are obtained as a racemic mixture, and one enantiomer is illustrated. β-Lactam **9q** containing the ring B phenol was obtained by reaction of the *tert*butylsilyloxy imine **8p** and 3,3-dimethylacryloyl chloride to afford the silyl ether **9p**, which was subsequently deprotected with *t*BAF to afford **9q** (Scheme 1). Interesting biochemical activity has been demonstrated by synthetic CA-4 analogues in which the phenol on ring B is replaced by the amino substituent [63]. The amino-substituted β-lactam **9s** was obtained by reduction of the nitro compound **9r** using zinc dust in the presence of acetic acid (Scheme 1).

The β-lactams **9a**-**9x** were obtained with exclusively *trans* configuration with coupling constant *J* = 1–3 Hz for H-3 and H-4 protons (*J* = 5–6 Hz are usually observed for β-lactams with *cis* stereochemistry [53]). For compound **9q**, the aliphatic signal at δ 1.88 pm was assigned to the methyl group of the C-3 isopropenyl substituent, with the corresponding carbon observed at δ 20.17 ppm in ^13^C NMR spectrum. H-3 was identified as a doublet at δ 3.72 coupled with H-4 at δ 4.71 (*J* = 2.52 Hz). The corresponding C-3 and C4 carbons appear at 60.50 and 66.37 ppm, respectively, in the ^13^C NMR spectrum. The geminal protons of H-6 appear in the ^1^H NMR spectrum at δ 5.02 and 5.07 as broad singlet signals, and C-6 is observed as a negative signal in the DEPT 90 spectrum at δ 113.93 ppm. The β-lactam carbonyl C-2 appeared furthest downfield in the ^13^C NMR spectrum at δ 164.81 ppm. (See Supplementary Materials Figures S1–S20). The β-lactams **9a**-**9x** demonstrated the characteristic β-lactam IR absorption at 1750 cm^−1^.

The X-ray crystal structure of compound **9h** (Table 1) confirmed the *trans* configuration for H-3 and H-4 and is consistent with data previously reported for colchicine [18], combretastatins [64,65] and monocyclic β-lactams [66,67] (Table S12). Hydrogen bonding is observed between the β-lactam carbonyl oxygen and the *ortho* hydrogen of ring A [68].

A series of *trans*-3-allyl-β-lactams, **10a**-**s**, with a variety of substituents at C4 of the β-lactam ring were next synthesised as CA-4 analogues from imines **8a**-**r** by Staudinger reaction using 4-pentenoyl chloride (Scheme 1 and Scheme 2). The compounds were obtained exclusively with *trans* stereochemistry in moderate yields (11–96%) after chromatographic purification. In the ^1^H NMR spectrum for **10h,** H-5 appeared as a multiplet (δ 2.55–2.74), with H-3 as a multiplet at δ 3.19–3.23. The doublet at δ 4.63 ppm was assigned to H-4 (*J* = 2.48 Hz), indicating the *trans* isomer was isolated. Two alkene methylene protons (H-7) were observed as a multiplet signal at δ 5.13–5.19, with the alkene methine proton (H-6) as a multiplet at δ 5.83–5.89. In the ^13^C NMR spectrum, the signals at 32.34 and 117.17 ppm (negative in DEPT-90 spectrum) are assigned to C-5 and C-7, respectively, and C-3 and C-4 of the β-lactam ring are observed at 60.06 and 60.49 ppm, respectively (see Supplementary Materials Figures S1–S20). The *trans* stereochemistry at C3 and C4 for **10h** was confirmed by X-ray crystallography analysis (Table 1 and Supplementary Materials, Tables S1 and S12). The phenolic compound **10p** was obtained in 22% overall yield on treatment of the silyl ether **10o** with TBAF, while the ring B amino compound **10r** was obtained by reduction of the nitro compound **10q** (96%).

The buta-1,3-dienyl-containing azetidinones **11a**-**o**, **11q** and **11s** were next prepared in moderate yield (16–55%) by direct reaction of the carboxylic acid (sorbic acid) with the panel of imines **8a**-**8r** using 2-chloro-N-methylpyridinium iodide as an acid-activating agent and tripropylamine as the base (Scheme 1 and Scheme 2) [69]. Exclusively *trans* isomer products were obtained. Additionally, **11s** could also be obtained by reaction of sorbyl chloride with imine **8w** using triethylamine as base [70]. Deprotection of the silyl ether **11o** with TBAF afforded the phenol **11p**, while reduction (Zn/acetic acid) of the 4-nitrophenyl-β-lactam **11q** yielded the amine **11r**.

The ^1^H NMR spectrum of the β-lactam **11c** confirmed the assigned structure, with the multiplet signal at δ 3.76–3.77 assigned to H-3 and the doublet at δ 4.74 (*J* = 2.00 Hz) assigned to H-4, confirming the *trans* isomer. The H-8 alkene protons were observed as multiplet signals (δ 5.17–5.29) and the alkene H-5 as a double doublet at δ 5.87 (*J*_5,6_ = 14.06 Hz and *J*_5,3_ = 8.02 Hz), while the multiplet signals δ 6.31-6.40 were diagnostic for the alkenes H-6 and H-7. In the ^13^C NMR spectrum, the β-lactam C-3 and C-4 signals are observed at 55.63 and 62.75 ppm, respectively; the signal at 118.46 ppm (negative in DEPT-90 spectrum) was assigned to the methylene C-8. The X-ray structure of compound **11h** demonstrates the *trans* configuration for H-3 and H-4 (Table 1 and Supplementary Materials, Tables S1 and S12). The amide **11t** [71], obtained by acylation of the sorbic acid, was isolated as a minor product in the preparation of **11s** (see X-ray crystal structure in Supplementary Materials Table S13).

**Scheme 3:**
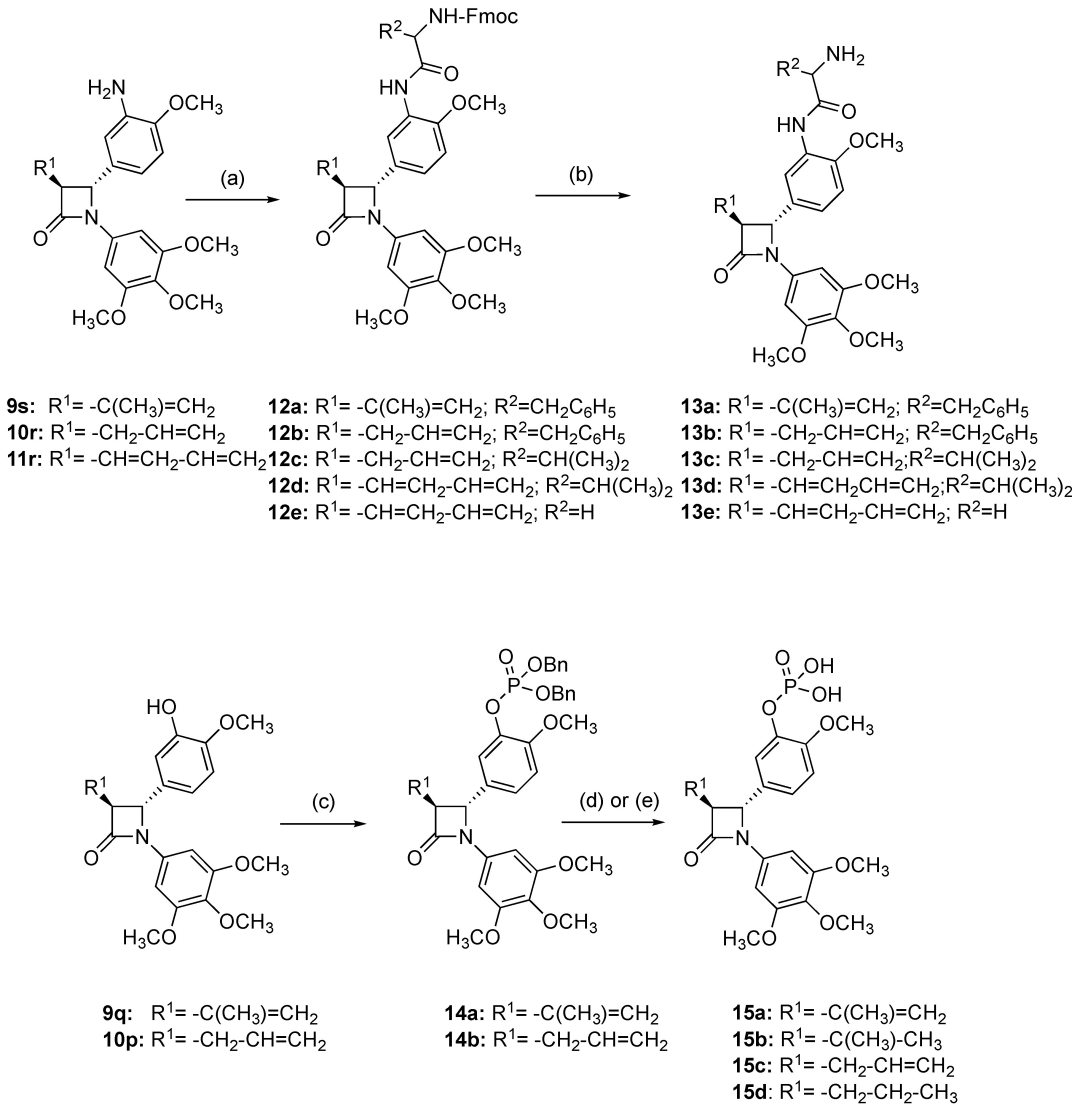
Synthesis of β-lactam prodrugs **13a**-**e, 15a**-**d.** Scheme reagents and conditions: Amino acid prodrugs **13a**-**e**: (**a**) Fmoc amino acid phenylalanine, glycine or alanine, anhydrous DMF, DCC, HOBt.H_2_O, 20 °C, 24 h (18–85%); (**b**) 2N NaOH aq, MeOH, 20 °C, 24 h (38–59%). Phosphate prodrugs **15a**-**d**: (**c**) dibenzyl phosphite, DIPEA, DMAP, CCl_4_, CH_3_CN, −10 °C (27–45%); (**d**) for compounds **15a** and **15c**, (i) bromotrimethylsilane, dry DCM, 45 min, (ii) Na_2_S_2_O_3_ (57–96%); (**e**) for compounds **15b** and **15d,** Pd/C (10%), EtOAc, 3 h (88–98%). Products obtained as a mixture of diastereomers (**12a**-**d**, **13a**-**d**) and enantiomers (**12e, 13e**, **14a, 14b** and **15a**); one enantiomer illustrated.

Prodrugs of amino-containing azetidinones **9s, 10r** and **11r** were prepared by reaction with the Fmoc-protected amino acids L-phenylalanine, L-valine and glycine using HOBt and the coupling reagent DCC (Scheme 3) [72]. The Fmoc protecting group was removed from products **12a**-**e** under mild basic conditions with aqueous sodium hydroxide in methanol (Scheme 3) to afford the β-lactam conjugates (**13a**-**e**) in 38–59% yield following purification by flash chromatography. The ^1^H NMR spectra of products **12a**-**e** and **13a**-**e** are complex, possibly due to the presence of rotamers arising from the amino acid moieties [73]. The ^1^H NMR spectrum of the diastereomeric prodrug **13e** confirmed the *trans* stereochemistry with H-4 as a doublet, δ 5.32 (*J*_3,4_ = 1.48 Hz), while an additional amide carbonyl signal in the region 1680–1690 cm^−1^ is observed in the IR spectra of these prodrugs (see Supplementary Materials Figures S1–S20).

Phosphate ester prodrugs of the phenols **9q** and **10p** were prepared by controlled esterification [74] to afford the dibenzyl phosphate esters (**14a**, **14b**) (Scheme 3). In the ^1^H NMR spectrum of **14a**, the H-4 signal was observed at δ 4.68 as a doublet (*J*_4,3_ = 2.52 Hz) and demonstrated *trans* stereochemistry with H-3 (δ 3.68, *J*_3,4_ = 2.04 Hz). The methyl protons were identified at δ 1.86; the alkene protons were assigned as broad singlets at δ 5.02 and δ 5.05, while the benzylic methylene signals occurring as a multiplet at δ 5.13-5.17 were confirmed with negative signals in the DEPT experiment (69.48, 69.50 ppm).

Treatment of the phosphate esters **14a** and **14b** with bromotrimethylsilane afforded the desired phosphates **15a** and **15c**. Reduction of **14a** and **14b** with hydrogen and palladium/carbon catalyst also allowed removal of the dibenzyl protecting groups; however, reduction of the C-3 alkene occurred to afford compounds **15b** and **15d** (without decomposition of the main four-member ring). The ^1^H NMR spectrum of compound **15a** confirms the removal of the benzylic group protons, with the isopropyl methyl signal at δ 1.86, and the β−lactam and vinylic protons overlapping as a multiplet at δ 4.91–5.06 (H-3, H-4, H6). In the reduced product **15b**, the methyl (δ 1.00–1.08) and methine (δ 2.06) signals for the C-3 isopropyl substituent are clearly identified together with the β-lactam H-3 and H-4 signals at δ 2.99 and δ 4.64, respectively.

**Scheme 4:**
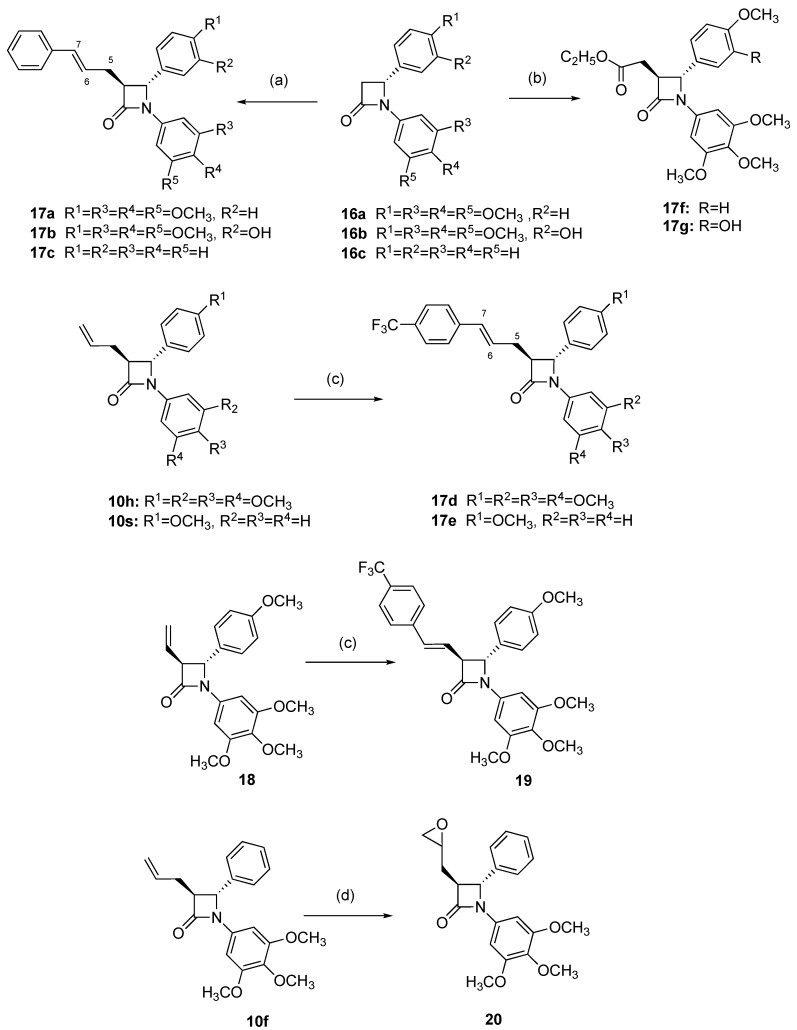
Synthesis of β-lactam compounds **17a**-**g**, **19** and **21**. Scheme reagents and conditions: (**a**) LDA, −78 °C, THF followed by 3-bromo-1-phenylpropene, 30 min, then 20 °C for 5 min (14–90%); (**b**) LDA, −78 °C, THF followed by ethyl bromoacetate, 30 min, then 20 °C for 5 min (60–77%); (**c**) 1-Bromo-4-(trifluoromethyl)benzene, Pd(II)(OAc)_2_, PPh_3_, KOAc, Bu_4_NCl, DMF, N_2_, 80 °C, 18 h (19–72%); (**d**) *m*CPBA, CH_2_Cl_2_, 24 h, 20 °C (67%). Products obtained as a mixture of enantiomers; one enantiomer illustrated.

The synthesis of structurally related β-lactams that contain alkene and ester substituents at C-3 was investigated (Scheme 4). Lithium enolates of 3-unsubstituted β-lactams react with numerous electrophiles to provide 3-substituted compounds [75]. In the present work, alkylation of the enolate of 3-unsubstituted β-lactams **16a**-**c** with cinnamyl bromide cleanly afforded the 3-substituted β-lactams (**17a**-**c**) (Scheme 4). Similarly, treatment of the enolates of compounds **16a** and **16b** with ethyl bromoacetate afforded the ester products **17f** and **17g**, respectively. The enolate chemistry is stereoselective, and compounds **17a**-**c**, **17f** and **17g** were obtained with exclusively *trans* stereochemistry. The structural assignment for compound **17a** was confirmed from the ^1^H NMR,^13^C NMR and HH COSY spectra (see Supplementary Materials, Figures S1–S20). The multiplet signals δ 2.69–2.93 were assigned as H-5 with coupling to H-3, H-6 and H-5, while the corresponding carbon appears as a negative signal at 31.68 ppm in the DEPT 90 spectrum. The multiplet signal δ 3.28–3.33 was attributed to H-3. The doublet at δ 4.69 (*J* = 2.28 Hz) was assigned to H-4 of the β-lactam ring. The multiplet (δ 6.24–6.31) was assigned to the alkene H-6 with coupling to H-5 and H-7. The signal at δ 6.51 is diagnostic for the alkene H-7 with a *trans* vicinal coupling constant of 15.8 Hz.

To explore the biological effects of additional structural modifications of the 3-(prop-1-en-2-yl), 3-allyl and 3-(buta-1,3-dien-1-yl)-1,4-diarylazetidin-2-ones, the alkenes **10s**, **10h** and **18** were successfully coupled with 4-trifluoromethylphenyl bromide using the catalyst PdOAc_2_/PPh_3_ to obtain the products **17d, 17e** and **19** with retention of the *trans* β-lactam stereochemistry (Scheme 4). Introduction of the trifluoromethyl substituent may be useful for exploring potential protein ligand interactions for these compounds [76]. Treatment of the 3-allyl-β-lactam **10f** with *meta*-chloroperbenzoic acid (*m*CPBA) afforded the epoxide **20** (67%).

**Scheme 5:**
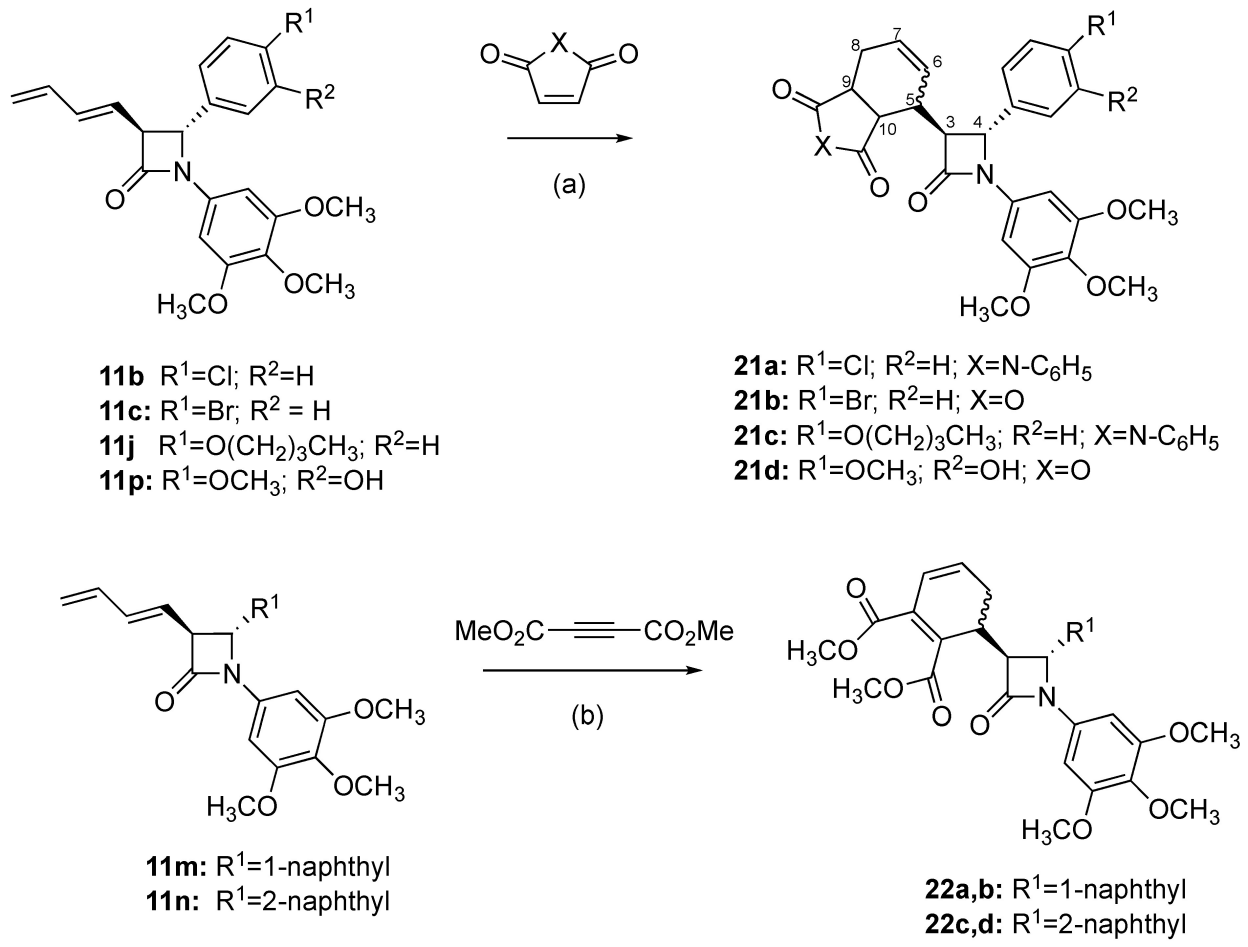
Synthesis of maleimide, maleic anhydride and dimethylacetylene dicarboxylate adducts **21a**-**d**, **22a**-**d**. Scheme reagents and conditions: (**a**) maleic anhydride or N-phenylmaleimide, toluene, reflux, 1 h (24–70%); (**b**) toluene, reflux, 6–7 h, N_2_ (8–14%).

In a further series of structural modifications, complex β-lactams were obtained by Diels–Alder cycloaddition reactions of 3-butadienylazetidiones **11b, 11c, 11j** and **11p** with maleic anhydride and N-phenylmaleimide as dienophiles to afford products **21a**-**d**, in moderate yields (Scheme 5). Diels–Alder cycloaddition reactions of 3-butadienylazetidinones with dienophiles such as acrylic acid and benzoquinone have been reported [70,77,78]. The structure of **21d** was confirmed from ^1^H, ^13^C and HMBC NMR spectra (see Supplementary Materials, Figures S1–S20). H-3 was observed as a double doublet (δ 3.94, *J* 2.00 Hz), with coupling to H-5 and H-4, while H-4 was identified as a doublet (δ 4.64) with *trans* coupling (*J*_3,4_ 2.00 Hz). The β-lactams **11m** and **11n** reacted with dimethylacetylene dicarboxylate to afford products **22a, 22b** and **22c, 22d** as diastereoisomeric mixtures in low yield (22–23%) (Scheme 5). Separation by flash chromatography afforded each of the pure diastereomeric β-lactams (**22a**, **22b** and **22c**, **22d**), and the diastereomeric ratio was determined for the isolated products to be 1.75:1 for **22a**:**22b** and 1.3:1 for **22c**:**22d**. The *trans* stereochemistry of the β-lactam products was confirmed from the ^1^H NMR spectrum, with *J* _3,4_ = 2 Hz.

### 2.2. Stability Study of β-Lactams

Stability evaluation of compounds was carried out to avoid subsequent significant loss of pharmacological activity in vivo. The stability of representative β-lactams **9q, 10h, 10q, 10p, 10r, 15a** and **CA**-**4** was evaluated by HPLC at relevant in vivo acidic, neutral and basic conditions (pH 4, 7.4 and 9) and in plasma (see Supplementary Materials, Tables S2 and S3 and Figure S21). The half-life (t_½_) for **9q** (the most potent compound in the series, containing the phenolic ring B) was determined to be 13 h at both pH 4 and pH 7.4 and 6 h at pH 9. However, the t_½_ in plasma for compound **9q** was greater than 24 h. The t_½_ of the corresponding phosphate prodrug phosphate ester **15a** was greater than 24 h at pH 4 and pH 7.4 and in human blood plasma. CA-4 was stable at pH 4.0, 7.4 and 9.0 and in human plasma for more than 7 h. Based on this stability study, β-lactam **9q** and the phosphate **15a** would be suitable for further development. The 3-allyl β-lactam compounds **10h** and **10q** demonstrated poor stability over the pH range studied. The 3-allyl β-lactam phenolic compound **10p** demonstrated superior stability at all pH values, with over 95% remaining after 11 days, compared to **10h** (24–41%) and **10q** (22–26%).

Compound **10r** (containing the 3-amino-4-methoxyphenyl ring A) showed similar results to phenolic compound **10p** (t_½_ > 24h). The resilience of **10p** and **10r** against ring opening degradation may be due to the additional electron-donating hydroxyl and amino substituent groups in ring B. Compound **10h** was the most stable of the 3-allyl compounds in plasma, with 100% recovery after 3 days, while compounds **10p, 10q** and **10r** showed 97%, 89% and 96% recovery respectively. Based on these stability studies, β-lactams **10p** and **10r** would be suitable for further development and compared well with CA-4. Clearly, the 3-allylic β-lactam compound **10p** is superior in stability to the corresponding isomeric 3-(prop-1-en-2-yl)-β-lactam **9q**. In an additional study, the stability of compound **10p** was determined under degradation conditions and was stable after 4 h treatment in acidic (0.1 M HCl), alkaline (0.1M NaOH), oxidative (3% H_2_O_2_), heat (60 °C) and UV light conditions, with 91%, 95%, 79%, 100% and 100% of the compound remaining, respectively (Supplementary Materials, Tables S2 and S3 and Figure S21).

### 2.3. Biological Results and Discussion

#### 2.3.1. In Vitro Antiproliferative Activity of 3-(Prop-1-en-2-yl)azetidinones **9a**-**9x**, 3-Allylazetidinones **10a**-**10s** and 3-butadienyl-β-lactams **11a**-**11s** in the MCF-7 Breast Cancer Cell Line

The antiproliferative activity of the novel 3-(prop-1-en-2-yl)azetidinones **9a**-**9x,** 3-allylazetidinones **10a**-**10s** and 3-butadienyl-β-lactams **11a**-**11s** was initially examined in the human breast cancer cell line MCF-7 (ER positive); CA-4 was used as the reference compound (IC_50_ = 3.9 nM in MCF-7 cells). The results are displayed in Table 2, Table 3 and Table 4. The β-lactams were evaluated as the *trans* isomers, which were isolated exclusively in these synthetic reactions as enantiomeric mixtures. The 3-(prop-1-en-2-yl)azetidinones **9a**-**9s** contain the characteristic 3,4,5-trimethoxyphenyl ring A at the N1 position of the azetidinone, while compounds **9t, 9u** and **9v** contain the 3,4,5-trimethoxyphenyl substituent (ring A of CA-4) at the C4 position. Compounds **9x, 10s** and **11s** contain a single aryl *para*-methoxy substituent at the C4 position, while compound **9w** contains a single aryl methoxy substituent at the N1 position.

The phenolic β-lactam compound **9q** containing the 3-hydroxy-4-methoxyphenyl ring B, designed as a direct analogue of CA-4, was identified as the most potent in the series, with an IC_50_ value of 10 nM (Table 2). Compound **9s** containing the ring B 3-amino-4-methoxyphenyl substitution pattern demonstrated notable potency, with IC_50_ = 0.033 mM; such amino ring B substitution is reported to contribute to antitubulin activity in many CA-4 analogues [63]. Compounds **9h** and **9i**, having methoxy and ethoxy electron-releasing substituents at C-4 of ring B, displayed potent antiproliferative effects, with IC_50_ = 33 nM and 81 nM, respectively. Among the other members of this series displaying sub-micromolar antiproliferative activity in the MCF-7 cell line were **9o** (4-thiomethyl)**, 9g** (4-methyl), **9e** (4-dimethylamino), **9b** (4-chloro) and **9c** (4-bromo) compounds. Interestingly, the 2-naphthyl compound **9n** (IC_50_ = 139 nM) was 58-fold more potent than the 1-naphthyl compound **9m**, as previously observed for stilbene CA-4 analogues [79]. Antiproliferative activity was minimal for compounds **9t, 9u** and **9v** containing the 3,4,5-trimethoxyphenyl ring A at the C-4 position, thus confirming the optimal N-1 position for activity.

**Table 2:** Antiproliferative activities of β-lactams **9a**-**9o, 9q**-**9x, 13a, 14b, 15a** and **15b** in MCF-7 human breast cancer cells.

Compound Number	Antiproliferative Activity IC_50_ (µM) ^a^	cLogP ^b^
**9a**	4.957 ± 0.69	3.06
**9b**	0.630 ± 0.07	4.03
**9d**	0.691 ± 0.10	4.18
**9e**	5.950 ± 0.92	3.46
**9f**	0.494 ± 0.14	3.32
**9h ^c^**	4.892 ± 0.53	3.49
**9j**	0.161 ± 0.010	3.82
**9k**	0.033 ± 0.005	3.24
**9l**	0.081 ± 0.009	3.77
**9m**	44.1 ± 2.57	4.83
**9n**	78.1 ± 5.47	5.42
**9o ^c^**	271.9 ± 26.4	5.01
**9r**	8.066 ± 0.93	4.50
**9s**	0.139 ± 0.04	4.50
**9t ^d^**	0.131 ± 0.04	3.88
**9u ^d^**	0.010 ± 0.001	2.32
**9v ^d^**	1.165 ± 0.12	2.70
**9w**	0.033 ± 0.005	2.19
**9x**	<10% inhibition	3.88
**13a**	<10% inhibition	3.77
**15a**	<10% inhibition	3.24
**15b**	144.6 ± 7.76	4.18
**CA-4 ^e^**	3.567 ± 0.32	4.18

^a^ IC_50_ values are half-maximal inhibitory concentrations required to inhibit growth stimulation of MCF-7 cells. Values represent the mean ± SEM (error values × 10^−6^) for at least three experiments performed in triplicate. Treatment at eight different concentrations in the range 1–50 μM over 72 h was used for determination of the IC_50_ values for each compound in comparison to the control compound CA-4. ^b^clogP values were calculated with ChemBioDraw 13.0 and are documented in the table. ^c^ [80] ^d^ IC_50_ values were not calculated; the percentage shown is the inhibition of cell viability at a concentration of 10 μM. ^e^ IC_50_ value determined for CA-4 (0.0039 μM) in MCF-7 cells is in agreement with reported values [81,82].

The series of β-lactams with the 3-allyl substituent at C-3 position of the β-lactam ring was next investigated (compounds **10a**-**10s**), and the results are displayed in Table 3, together with the cLogP values. As observed for the 3-(prop-1-en-2-yl)azetidinones series, the most potent compounds in the 3-allyl series were identified as **10p** (3-hydroxy-4-methoxyphenyl ring B, IC_50_ = 31 nM), **10h** (4-methoxyphenyl ring B, IC_50_ = 35 nM) and **10r** (3-amino-4-methoxyphenyl ring B, IC_50_ = 35 nM). The 4-ethoxyphenyl compound **10i** also displayed excellent antiproliferative activity against MCF-7 cells, with an IC_50_ value of 91 nM. The remaining compounds displayed reduced activity.

The 3-Butadienyl β-lactam compounds **11a**-**11s** were also evaluated for antiproliferative activity in MCF-7 human breast cancer cells and showed trends that were consistent with the 3-propenyl and 3-allyl β-lactam series (Table 4). The most active compounds in the series were again identified as the ring B *para*-methoxy **11h** (IC_50_ = 33 nM), the ring B 3-amino-4-methoxyphenyl compound **11r** (IC_50_ = 36 nM) and the ring B 3-hydroxy-4-methoxyphenyl compound **11p** (IC_50_ = 61 nM). The remaining compounds showed reduced antiproliferative activities against MCF-7 cells.

**Table 3:** Antiproliferative activities of β-lactams **10a**-**10n**, **10p**-**10s**, **13c**, **13d** and **15d** in MCF-7 human breast cancer cells.

Compound Number	Antiproliferative Activity IC_50_ (µM) ^a^	cLogP ^c^
**10a**	4.128 ± 0.62	2.99
**10b**	0.621 ± 0.095	3.96
**10c**	0.944 ± 0.10	4.11
**10d**	33.80 ± 9.04	3.39
**10e**	4.46 ± 0.11	3.42
**10f**	9.609 ± 1.20	3.25
**10g**	0.199 ± 0.03	3.75
**10h**	0.035 ± 0.005	3.17
**10i**	0.091 ± 0.01	3.7
**10j**	17.41 ± 1.23	4.76
**10k**	7.494 ± 0.60	5.35
**10l**	7.388 ± 0.67	4.94
**10m**	0.210 ± 0.026	4.43
**10n**	0.318 ± 0.03	4.43
**10p**	0.031 ± 0.002	2.25
**10q**	0.619 ± 0.07	2.63
**10r**	0.035 ± 0.004	2.12
**10s**	84.50 ± 8.10	4.11
**13b**	10.67 ± 1.94	2.45
**13c**	13.02 ± 1.25	1.93
**15c**	0.094 ± 0.015	1.17
**15d**	0.032 ± 0.005	1.65
**CA-4 ^b^**	0.0039 ± 0.00032	3.32

^a^ IC_50_ values are half-maximal inhibitory concentrations required to block the growth stimulation of MCF-7 cells. Treatment at eight different concentrations [0.001–50 μM] was used for the determination of the IC_50_ values for each compound in comparison to the control compound CA-4. Values represent the mean ± SEM (error values × 10^−6^) for at least three experiments performed in triplicate. ^b^ IC_50_ value determined for CA-4 (0.0039 μM) in MCF-7 cells is in agreement with reported values [81,82]. ^c^ clogP values were calculated using ChemBioDraw 13.0 and are documented in the table.

**Table 4:** Antiproliferative activities of β-lactams **11a**-**n**, **11p**-**s**, **13d** and **13e** in MCF-7 human breast cancer cells.

Compound Number	Antiproliferative Activity IC_50_ (µM) ^a^	cLogP ^c^
**11a**	0.784 ± 0.13	3.3
**11b**	0.315 ± 0.04	4.27
**11c**	0.428 ± 0.042	4.42
**11d**	3.214 ± 0.62	3.7
**11e**	1.929 ± 0.34	3.72
**11f**	19.4 ± 4.03	3.56
**11g**	0.200 ± 0.04	4.06
**11h**	0.033 ± 0.005	3.48
**11i**	0.261 ± 0.05	4
**11j**	182.4 ± 19.4	5.06
**11k**	37.89 ± 3.80	5.65
**11l**	144.9 ± 11.66	5.24
**11m**	0.576 ± 0.10	4.73
**11n**	0.296 ± 0.05	4.73
**11p**	0.061 ± 0.008	2.56
**11q**	0.511 ± 0.09	2.94
**11r**	0.036 ± 0.005	2.03
**11s**	15.21 ± 1.41	4.42
**13d**	24.16 ± 3.91	2.23
**13e**	9.359 ± 1.21	0.99
**CA-4 ^b^**	0.0039 ± 0.00032	3.32

^a^ IC_50_ values are half-maximal inhibitory concentrations required to block the growth stimulation of MCF-7 cells. Treatment at eight different concentrations [0.001–50 μM] was used for the determination of the IC_50_ values for each compound in comparison to the control compound CA-4. Values represent the mean ± SEM (error values × 10^−6^) for at least three experiments performed in triplicate ^b^ IC_50_ value determined for CA-4 (0.0039 μM) in MCF-7 cells is in agreement with reported values [81,82]. ^c^ clogP values were calculated using ChemBioDraw 13.0.

The antiproliferative activity of the amino acid and phosphate prodrugs synthesised was next evaluated in MCF-7 cells. The antiproliferative activity for the phenylalanine prodrug **13a** (IC_50_ = 4.915 μM) was low, indicating that in vivo hydrolysis of the amide is required to release the active amine **9s** (Table 2) [83]. Reduced activity was also observed for amino acid prodrugs **13b**-**e** (Table 3 and Table 4). However, the 3-isopropenyl phosphate ester **15a** displayed impressive antiproliferative activity, with an IC_50_ value of 19 nM (Table 2), compared with parent phenol **9q** (IC_50_ = 10 nM), suggesting that this compound is a useful prodrug, as in vivo dephosphorylation to produce **9q** would be expected to occur as observed for CA-4P [84]. The 3-isopropyl group in compound **15b** resulted in reduced activity (Table 2). However, the phosphates **15c** and **15d** prepared from the 3-allyl phenolic **10p** retained excellent activity, with IC_50_ values of 94 nM and 32 nM, respectively (Table 3).

#### 2.3.2. In Vitro Antiproliferative Activity of Structurally Modified β-Lactams **17a**-**g**, **19**, **20**, **21a**-**d**, **22a**-**d** in the MCF-7 Breast Cancer Cell Line

To determine the effect of modification of the alkene substituent at C-3 of the β-lactam ring of the designed compounds, a number of structurally related β-lactams were synthesised for evaluation. Compounds **17a**-**e** were designed to determine the effect of the introduction of an aryl substituent at the C-3 allylic position of potent 3-allyl-β-lactam compounds **10h** and **10p**, while all aryl ring A and ring B methoxy substituents were removed in compound **17c**. In compounds **17f** and **17g**, an ethyl ester group was introduced to replace the C-3 alkene substituent (isopropenyl, allyl and butadiene). In compounds **17d**, **17e** and **19**, a trifluoromethylstyryl substituent was located at C-3 in place of the 3-(prop-1-en-2-yl) and 3-butadienyl series compounds.

The compounds were evaluated in the MCF-7 cell line, and the results are displayed in Table 5. Compounds **17a**, **17b and 17c** demonstrated reduced antiproliferative effects, compared with the parent compounds **10h** and **10p.** Interestingly, the ester compounds **17f** and **17g** demonstrated the most potent antiproliferative effects of the modified series in MCF-7 cells, with IC_50_ values of 35 nM and 75 nM, respectively. Compound **19**, having a trifluoromethylstyryl substituent at C-3 to replace the alkene, and **17e**, having a trifluoroaryl substituent at the C-3 allylic position, both displayed moderate activity, while activity was poor for the ring A unsubstituted compound **17f** and the epoxide **20**.

The antiproliferative activity of modified compounds **21a**-**d** and **22a**-**d** is presented in Table 5. A significant decrease in activity for all β-lactam compounds was observed compared with the parent 3-butadienyl β-lactam compounds and CA-4, which indicated that reduction in antiproliferative activity may result from increasing the substituent group size at the C-3 position of β-lactam ring, in line with our previous observations [85]. The most potent compound of the series was the *trans* diastereoisomer **22d** with IC_50_ = 5.33 µM, which was 3-fold more potent than the diastereomer **22c** (IC_50_ = 15.33 µM).

#### 2.3.3. Evaluation of Antiproliferative Activity of Selected β-Lactam Compounds in the MDA-MB-231, HL60, HT29 and SW60 Cell Lines

Selected examples of the more potent compounds were evaluated in the triple-negative MDA-MB-231 breast cancer cell line (Table 6). Triple-negative MDA-MB-231 breast cancer cells do not express the ER, progesterone (PR) or HER2 receptors and possess mutant p53 [86]. Triple-negative breast cancers (TNBCs) account for 10–15% of breast cancers diagnosed, have poor prognosis and are not responsive to hormone therapies, e.g., tamoxifen, to aromatase inhibitors such as anastrozole or to the HER2-receptor-targeting antibody Herceptin.

Of the 3-isopropenyl series, the phenolic compound **9q** was found to be the most effective, with an IC_50_ value of 23.9 nM in the triple-negative MDA-MB-231 cell line. It compared favourably with the positive control CA-4 (IC_50_ = 43 nM) in this cell line [87,88] and also with the result for **9q** in the MCF-7 cell line (IC_50_ = 10 nM). The *p*-methoxy compound **9h** and the phosphate ester **15a** were also very effective in the MDA-MB-231 cell line, with IC_50_ values of 31.3 nM and 32 nM, respectively. Of the 3-allyl and butadienyl compounds evaluated in the MDA-MB-231 cell line, the phenolic **10p** was notable, with IC_50_ = 27 nM, together with the amine **10r** (IC_50_ = 35 nM), the methoxy **11h** (IC_50_ = 31 nM) and the amine derivative **11r** (IC_50_ = 33 nM). It is notable that the compounds retained potency in the MDA-MB-231 cell line, which may indicate their potential in development of therapeutic applications for this group of aggressive breast cancers.

**Figure 2:**
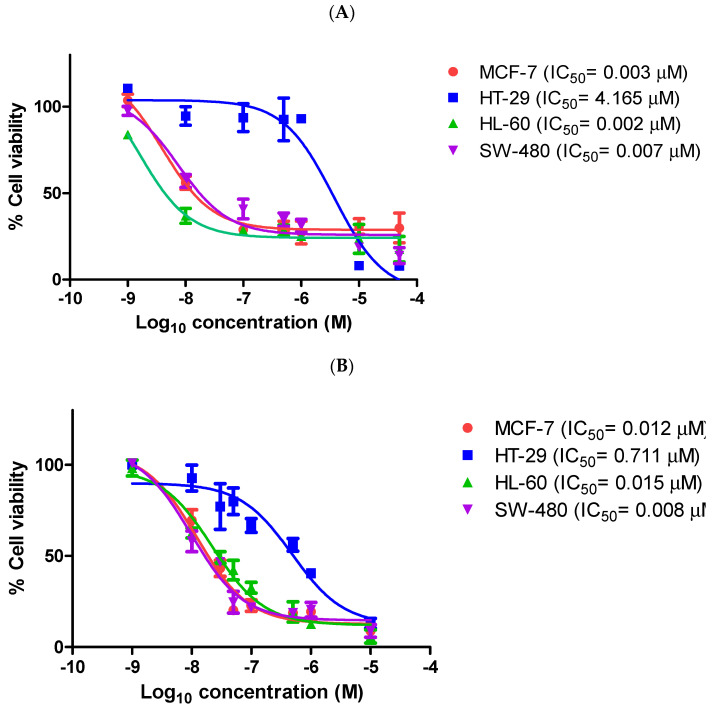
Antiproliferative activity of β-lactam compound **9q** and CA-4 in MCF-7 human breast cancer cells, HL-60 leukaemia cells, HT-29 and SW-480 colon cancer cells.

In addition, the 3-isopropenyl compound **9q** was found to be particularly effective in the chemoresistant HT-29 human colorectal adenocarcinoma cell line, with an IC_50_ value of 0.711 μM, in comparison to the control CA-4 (IC_50_ = 4.165 μM) in this cell line (Figure 2), indicating the potential of these compounds in chemoresistant colon cancers. Furthermore, **9q** was also evaluated in the human leukaemia cell line HL-60 (acute myeloid leukaemia) and the colon adenocarcinoma cell line SW-480, with IC_50_ values of 15 nM and 8 nM, respectively. It compares well with the control compound CA-4 (IC_50_ = 2 nM and 7 nM, respectively, in these cell lines) (Figure 2). The physicochemical properties of the most potent compounds were next investigated.

### 2.4. Physicochemical Properties

The physicochemical properties and various metabolic properties of eleven selected β-lactam compounds from the panel synthesised were also determined to establish their druggability (see Supplementary Materials Tables S4 and S5). The relevant physicochemical and pharmacokinetic properties of the most potent compounds, **9h**, **9q**, **9s**, **10h**, **10p**, **10r**, **11h**, **11p**, **11r, 15a** and **17g**, were obtained using Pipeline Pilot Professional [89]. The physicochemical properties of these compounds were found to comply with the requirements of Lipinski and Veber rules, with molecular weight range 445–457, hydrogen bond acceptor range 5–9, hydrogen bond donor range 0–2, 7–10 rotatable bonds and logP range 2.61–3.63. There is some correlation observed between the log P values calculated for the compounds and the antiproliferative activity determined (see Table 4, Table 5 and Table 6). The most potent compounds, **9q, 9s, 10q, 10r, 11q, 11r** and phosphate **15a**, with IC_50_ values in the range 10–61 nM in MCF-7 cells, have a low cLog P values in the range 1.235–2.55. However, the isomeric 1 and 2-naphthyl compounds **9m** and **9n**, each having higher cLogP values of 4.95, demonstrated a significant difference in potency, with IC_50_ values of 8.066 μM and 0.139 μM, respectively, in MCF-7 cells, indicating that the 2-naphthyl compound **9n** may be a better fit at the colchicine-binding site.

The pK_a_H value for potent compound **9q** was calculated with Marvin as 9.83, while the phosphate **15a** was ionised at physiological pH with pK_a_H values of 1.62 and 6.59. The calculated topological polar surface area (TPSA) of this panel was in the range 57–103 Å^2^, below the required limit of <140 Å^2^ for good intestinal absorption and membrane permeability. Additionally, three of the compounds, **9h**, **10h** and **11h**, followed the Pfizer rule and GSK rule (MW ≤ 400, log P ≤ 4), with a low log P value (log P < 3) and a low TPSA value (TPSA <75 Å^2^). The compounds exhibited good drug-likeness parameters with predicted lipophilic–hydrophilic balance, together with high blood–brain barrier (BBB) absorption and plasma-protein-binding properties (>90%), and were not predicted to inhibit the metabolic activity of CYP2D6 (see Supplementary Materials Tables S4 and S5 for details).

Good aqueous solubility (logSw = −3.4130 mol/L) was predicted for the phenolic ester compound **17g**, although lower aqueous solubility was predicted for the remaining panel of β-lactam compounds (see Supplementary Materials, Table S4); phosphate esters such as **15a**-**d** and amino acid prodrugs such as **13a**-**e** may result in increased water solubility, as reported for CA-4 and related compounds [72,74]. The most potent compounds identified in the preliminary screening panel [**9h**, **9q**, **9s**, **10h**, **10p**, **10r**, **11h**, **11p**, **11r**, **17g**] were confirmed as free from pan-assay interference compounds (PAINS) alerts [90] and were identified as suitable candidate compounds for subsequent in vitro biochemical investigations based on the phenotypic screening and Tier-1 profiling of their drug-like properties (Tables S4 and S5, Supplementary Materials) [91].

Cells were grown in 96-well plates and treated with (**A**) CA-4 or (**B**) β-lactam compound **9q** at 0.001–50 μM for 72 h. Cell viability was expressed as a percentage of vehicle control (ethanol 1% (*v*/*v*))-treated cells. The values represent the mean ± S.E.M. for three independent experiments performed in triplicate.

#### 2.4.1. National Cancer Institute 60 Cell Line Screening for Azetidinones **9h**, **9q**, **9s**, **10h**, **10p**, **10r**, **11h**, **11p**, **11r**, **15a** and **15b**

The more potent compounds **9h**, **9q**, **9s**, **10h**, **10p**, **10r**, **11h**, **11p**, **11r**, **15a** and **15b** were selected for screening for antiproliferative activity by the National Cancer Institute (NCI)/Division of Cancer Treatment and Diagnosis (DCTD)/Developmental Therapeutics Program (DTP) [92] in their drug screening programme and evaluated using approximately 60 different human cancer cell lines. The initial NCI 60 cell line screen used the sulforhodamine B (SRB) protein assay [93], at one dose concentration (10 μM), and subsequently, a five-dose screening in the concentration range 0.01–100 μM was carried out. GI_50_ (50% growth inhibition), TGI (total growth inhibition) and LC_50_ (50% lethal concentration) were determined (see Table 7 and Supplementary Materials Tables S6–S8). The compounds demonstrated excellent broad-spectrum antiproliferative activity against a range of tumour cell lines contained in the NCI panel of cancer cell lines, e.g., leukaemia, colon cancer, CNS cancer, melanoma, non-small-cell lung cancer, ovarian cancer, renal cancer, breast cancer and prostate cancer, and the results confirmed our in-house evaluations in the MCF-7 cell line.

**Figure 3:**
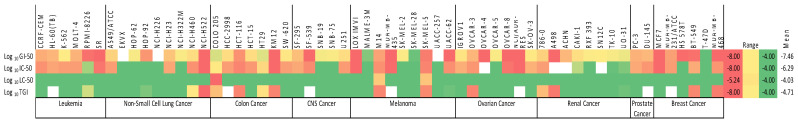
Heatmap of the activity of compound **9q** across cell lines in the NCI-60 screen using three different values (growth-inhibitory effect, GI_50_; drug concentration at which the response is reduced by half, IC_50_; cytostatic effect, TGI; cytotoxic effect, LC_50_; concentration in molar).

The ring B phenolic 3-isopropenyl compound **9q** was identified as the most potent compound synthesised in our panel, with a mean GI_50_ value obtained across the entire NCI panel of cell lines screened of 39.81 nM. (See Figure 3 for a heatmap of the activity of compound **9q** across the cell lines in the NCI-60 screen). This result compares favourably with the mean GI_50_ for CA-4 of 99.30 nM in the NCI-60 cell panel, demonstrating the superior inhibitory potency of the β-lactam analogue (see Table 7 and Supplementary Materials Tables S6–S8 for further details). Significantly, the GI_50_ values for **9q** were in the sub-micromolar range for all cell lines investigated, except for the breast cancer cell line T-47D (ERα+/PR+ and HER2-). Furthermore, **9q** displayed significant potency in all other breast cancer cell lines evaluated in the panel (MCF-7, MDA-MB-231, HS 578T, BT-549, MDA-MB-468), with GI_50_ values in the range <10–16 nM.

GI_50_ values below 50 nM were obtained for compound **9q** in 48 of the panel cell lines tested, with activity against non-small-cell lung, colon, CNS, ovarian and prostate cell lines tested. Figure 3 displays a heatmap of the activity of compound **9q** across the cell lines in the NCI-60 screen. In addition, compounds **9s, 11h** and **11r** were found to be particularly effective in the chemoresistant HT-29 human colorectal adenocarcinoma cell line, with IC_50_ values of 34, 33 and 34 nM, in comparison to the control compound CA-4 (IC_50_ = 4.165 μM). Compounds **9h, 9q, 9s, 10p, 11h, 11r** and **15a** displayed significant potency when evaluated in the leukaemia cell line HL-60 (acute myeloid leukaemia), with GI_50_ values of < 34 nM.

The mean GI_50_ values for the panel of 60 cell lines for the most potent compounds evaluated (**9h, 9q, 9s, 10h, 10p, 10r, 11h** and **11r**) were determined to be <91.20 nM, as shown in Table 7. These results compare very favourably with the mean GI_50_ value determined for CA-4 of 99.3 nM in this 60-cell-line panel.

The COMPARE programme developed by the NCI was used to further investigate the mechanism of action of the β-lactam series [95]. The antiproliferative profiles of potent compounds **9q** and **9s** were compared with compounds possessing known mechanisms of antiproliferative action in the NCI Standard Agents Database. Based on GI_50_, TGI and LC_50_, the compounds **9q** and **9s** demonstrated a good correlation to tubulin-targeting agents such as maytansine (r = 0.741) and also to the clinically utilised tubulin-targeting anticancer drugs vincristine (r = 0.664), vinblastine (r = 0.632) and paclitaxel (r = 0.768) (see Supplementary Materials, Tables S9 and S10).

#### 2.4.2. Effect of β-Lactam 9q on Non-Carcinogenic HEK-293T Cells

The 3-(prop-1-en-2-yl)azetidin-2-one compounds **9h** and **9q** were next examined for cytotoxic effects in MCF-7 cells at 10 μM concentration using the lactate dehydrogenase (LDH) assay, in which the release of cytoplasmic LDH is used as a measure of cell lysis [96]. The b-lactams **9h** and **9q** resulted in 30% and 32% cell death, respectively. CA-4 was used as the positive control in this assay, with 12% cell death at 10 μM.

**Figure 4:**
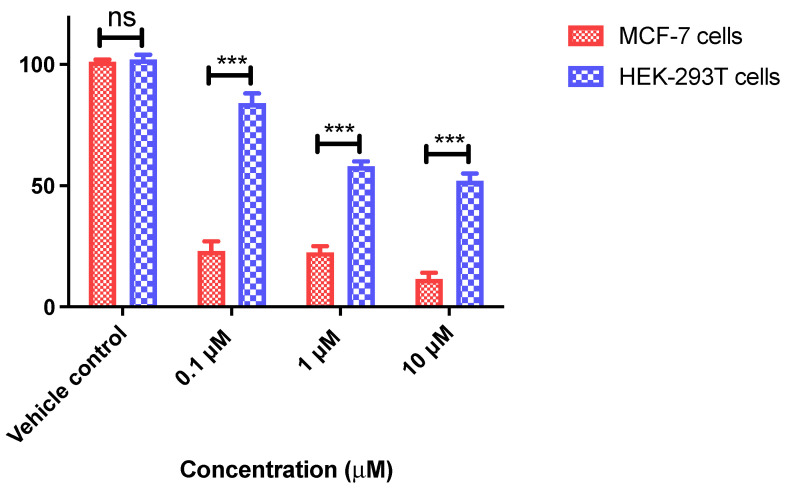
Antiproliferative activity of β-lactam compound **9q** in human breast cancer MCF-7 cells and non-tumorigenic human embryonic kidney HEK-293T cells. MCF-7 and HEK-293T cells were grown in 96-well plates and treated with compound **9q** at 0.1, 1 and 10 μM for 72 h. Cell viability (as a percentage of vehicle control (ethanol 1% (*v*/*v*) treated cells) was determined in an AlamarBlue assay. Values represent the mean ± SEM (error values × 10^−6^) obtained for at least three independent experiments. Two-way ANOVA (Bonferroni post-test) was used to test for statistical significance (***, *p* < 0.05). Cell cycle and pro-apoptotic effects of 3-(prop-1-en-2-yl)azetidinone **9q**.

The cytotoxicity of the most potent example of the 3-isopropenyl series, **9q**, was also investigated in the non-tumorigenic cell line HEK-293T (normal human epithelial embryonic kidney cells). The viability of the normal HEK-293T cells was demonstrated to be significantly higher than the treated MCF-7 cells following exposure to compound **9q** for 72 h. The cell viabilities observed in the HEK-293T cells were 83%, 60% and 50% at the concentrations of 0.1 μM, 1 μM and 10 μM, respectively (Figure 4); this result compares very favourably with the viabilities obtained in MCF-7 cells of 22%, 21% and 11% at the concentrations of 0.1 μM, 1 μM and 10 μM, respectively, with IC_50_ = 10 nM in the MCF-7 cell line, demonstrating that β-lactam **9q** was selectively less toxic to human normal cells (HEK-293T) than cancer cells (MCF-7).

These results indicate that compound **9q** is suitable for further development as an anticancer agent for breast cancers with selective lower cytotoxicity to normal cells. Additionally, the mean TGI (total growth inhibition) value for the potent compound **9q** over the NCI-60 cancer cell line panel was 32 mM (compared with TGI for CA-4 of 10.30 μM), with TGI values >100 μM for 34 of the cell lines tested indicating a wide therapeutic window for the compound (Table 7).

LC_50_ values obtained from the NCI screen over the 60 cancer cell lines were also useful in assessing the cytotoxicity of these compounds (Table 7). For compound **9q**, LC_50_ values were greater than 100 μM in all but two of the cell lines tested (melanoma M14 and colon COLO 205), which indicated minimal toxicity and suggested the potential for this compound in a number of therapeutic applications (Tables S6–S8 Supporting Information). For compound **9h**, the LC_50_ values were greater than 100 μM in all but one of the cell lines tested (melanoma SK-MEL-5). Similarly low cytotoxicity was also obtained for the related compounds evaluated in the series, e.g., compounds **9s, 15a, 15b, 10r, 10p, 11r** and **11p**. The antiproliferative activity determined for **9q** (IC_50_ <10 nM against the MCF-7 cell line) demonstrated a significant therapeutic window between the drug concentration required for cancer cell growth inhibition (GI_50_) and the concentration that is toxic to these MCF-7 cells, LC_50_ (>100 μM). For compound **9q**, the mean GI_50_ value over all 60 cell lines was 39.81 nM, while the mean LC_50_ value was 81.28 μM.

#### 2.4.3. Cell Cycle and Pro-Apoptotic Effects of 3-(Prop-1-en-2-yl)azetidinone **9q**

**Figure 5:**
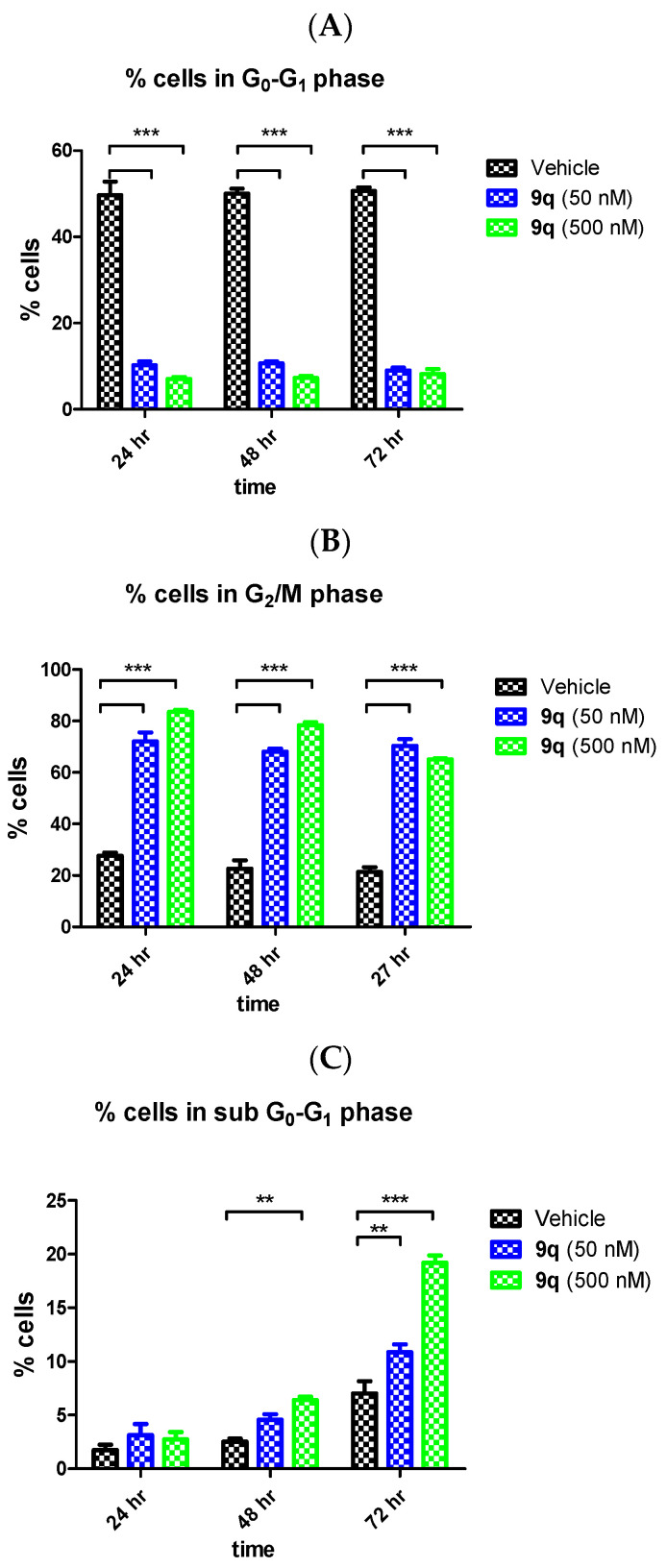
Effect of β-lactam compound **9q** on the cell cycle and apoptosis in MCF-7 cells. MCF-7 cells were treated with either vehicle (0.1% ethanol (*v*/*v*)) or **9q** (50 nM and 500 nM) for 24 h, 48 h and 72 h. The MCF-7 cells were then fixed and stained with propidium iodide, and the distribution was analysed by flow cytometry. Cell cycle analysis was obtained from histograms of gated counts per DNA area (FL2-A). The number of cells with <2N (sub-G_1_) (**A**), 2N (G_0_G_1_) (**B**) and 4N (G_2_/M) (**C**) DNA content was determined using CellQuest software. The results obtained represent the mean of three independent experiments performed. Two-way ANOVA (Bonferroni post-test) was used to test for statistical significance (**, *p* < 0.05; ***, *p* < 0.001).

The effects of the β-lactam **9q** on the cell cycle in MCF-7 cells were next explored in flow cytometry studies. Tubulin-destabilising agents such as colchicine and CA-4 arrest the cell cycle in the G_2_/M phase, promoting depolymerisation of microtubules and disruption of the cytoskeleton, while the antimitotic action of paclitaxel is to stabilise the microtubule polymer and prevent disassembly. MCF-7 cells were treated with **9q** and analysed by propidium iodide staining (Figure 5). The effect of compound **9q** on the mitotic phase G_2_/M (50 μM and 500 μM) at 24 h, 48 h and 72 h was first determined (Figure 5B). For the 50 μM concentration, the G_2_/M population increased at each of the time points examined to 72% (24 h), 69% (48 h) and 70% (72 h), while this cell population also increased when treated at 500 nM to 85% (24 h), 81% (48 h) and 70% (72 h). In contrast, the G_0_G_1_ cell population decreased (10% and 7% (24 h), 10% and 7% (48h) and 8% and 8% (72 h)) when treated with **9q** at 50 nM and 500 nM, respectively (Figure 5A). The vehicle control (ethanol 0.1% *v*/*v*) is shown for comparison (Figure 5). A proapoptotic effect (sub-G_0_G_1_) was evident for compound **9q** (10% and 19% at 50 nM and 500 nM, respectively) at 72 h, compared with vehicle control (6%) (Figure 5C).

**Figure 6:**
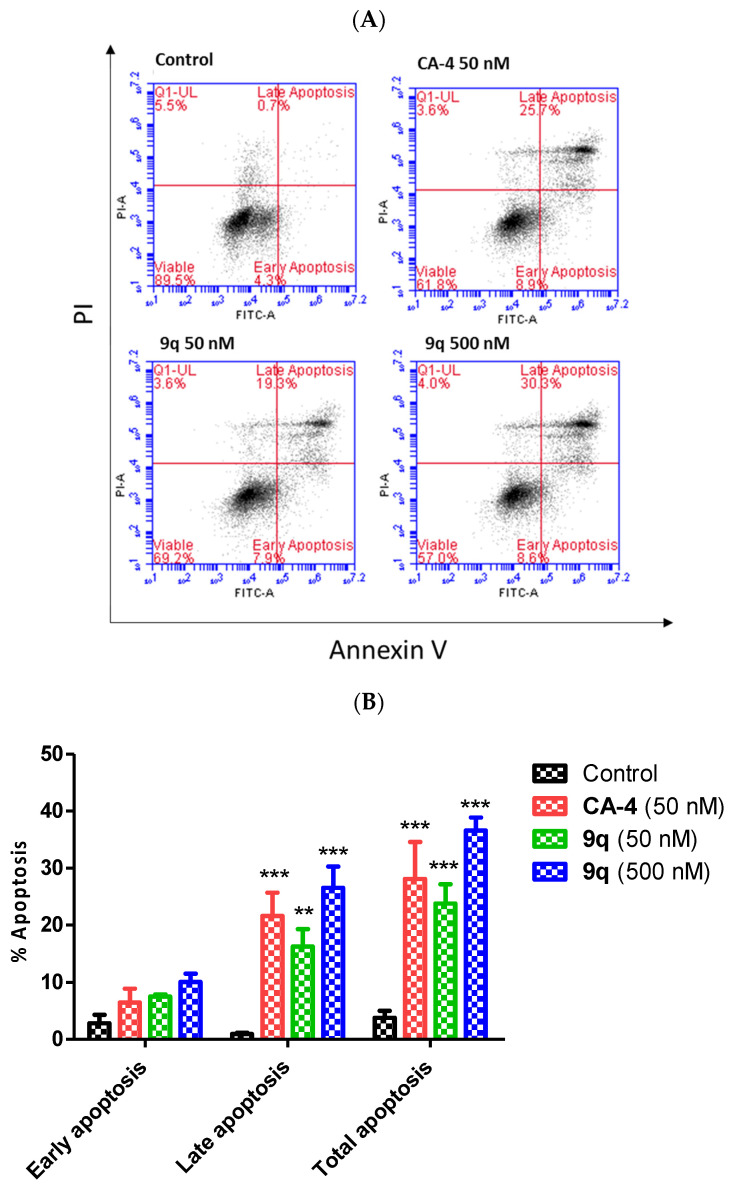
β-Lactam compound **9q** induces apoptosis in MCF-7 cells (annexin-V / PI FACS). (**A**) The effect of β-lactam compound **9q** on the induction of apoptosis in MCF-7 cells was analysed by flow cytometry following double staining of the MCF-7 cells with annexin-V-FITC and PI. The MCF-7 cells were treated with 50 and 500 nM concentrations of β-lactam compound **9q** and CA-4 (50 nM) for 72 h (control 0.1% (*v*/*v*) ethanol). Cells were then collected and processed for analysis. (**B**) The percentage of cells in each phase of the cell cycle in MCF-7 cells treated with the indicated concentration of **9q** is shown. Two-way ANOVA (Bonferroni post-test) was used to test for statistical significance (**, *p* < 0.05; ***, *p* < 0.001).

To further investigate and confirm the induction of apoptosis by **9q** in MCF-7 cells, a dual staining with annexin-V and with propidium iodide (PI) was performed (Figure 6). Live cells (annexin-V-/PI-), early apoptotic cells (annexin-V+/PI-), late apoptotic cells (annexin-V+/PI+) and necrotic cells (annexin-V-/PI+) can be identified. Compound **9q** induced an increase in apoptosis (observed as annexin-V-positive cells) in a concentration-dependent manner when compared to CA-4 (Figure 6) when examined at 72 h, with 27% of cells undergoing apoptosis (early+late) at 50 nM concentration of **9q**, and 38.9% at 500 nM. CA-4 (50 nM) induced apoptosis in 34.6% of the MCF-7 cells. Cell death may also be due to autophagy following treatment with **9q** [97]. Collectively, these findings indicate that the β-lactam compound **9q** demonstrated tubulin-targeting effects, e.g., G_2_/M arrest followed by apoptosis, on cell cycle in MCF-7 cells. The effects of the compounds on tubulin polymerisation were further examined.

#### 2.4.4. Tubulin Polymerisation Effects of 3-(Prop-1-en-2-yl)azetidinones, 3-Allylazetidinones and 3-butadienylazetidinones

**Figure 7:**
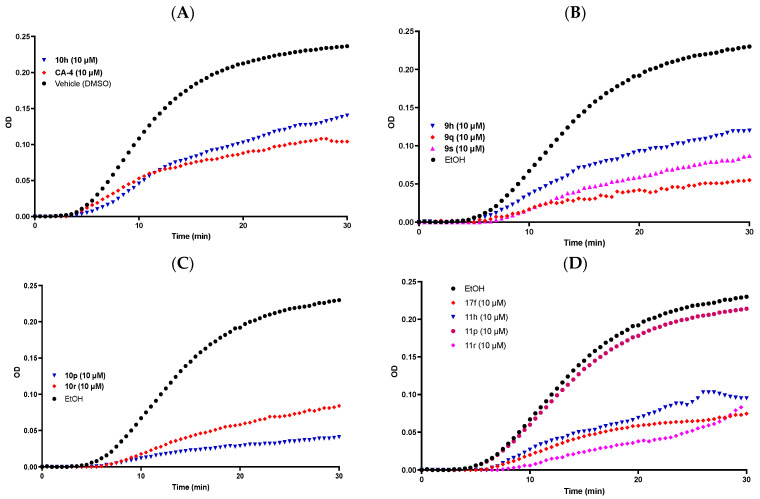
β-Lactam compounds **9h, 9q, 9s, 10h, 10p, 10r, 11h, 11p, 11r, 17f** induce tubulin polymerization in vitro. (**A**) Compounds CA-4, **10h** (10 μM) and vehicle DMSO (1% *v*/*v*); (**B**) compounds **10h**, **10q**, **10s** (10 μM) and vehicle ethanol (1%*v*/*v*); (**C**) compounds **10p**, **10r** (10 μM) and vehicle ethanol (1% *v*/*v*); (**D**) compounds **17f**, **11h, 11p**, **11r** (10 μM) and vehicle ethanol (1% *v*/*v*). The tubulin polymerization reaction was initiated by gently warming a solution of purified bovine tubulin and GTP (mixed in a 96-well plate) from 4 °C to 37 °C. Ethanol or DMSO (1% *v*/*v*) was used as a vehicle control. CA-4 (10 μM) was used as a reference. β-Lactam compounds were tested at a final concentration of 10 μM. Tubulin polymerisation was monitored at 37 °C with a Spectramax 340PC spectrophotometer at 340 nm for 30 min at 30 s time intervals. The inhibition (fold) of the tubulin polymerization reaction was calculated using the V_max_ value for each reaction. The representative results of three separate experiments are shown.

A panel of β-lactam compounds that demonstrated the most potent antiproliferative effects in vitro were selected for study of their inhibitory effect on tubulin assembly using a light-scattering assay. CA-4 was used as the positive control for the tubulin polymerization experiment, which effectively inhibits the assembly of bovine tubulin; paclitaxel was the positive control for polymerisation, with ethanol and DMSO as the solvent vehicles. (Figure 7). The V_max_ value for the polymerization reaction for each compound was determined (Table 8), in addition to the fold-changes in the V_max_ values for the polymerisation reaction [98].

The ring B phenolic 3-allyl **10p** was identified as the most potent polymerisation inhibitor in the series (V_max_ 0.0015 mOD/min), with 4.5-fold reduction in V_max_ value at 10 μM concentration. This result compares favourably to CA-4 (10 μM), for which a 6.3-fold inhibition in the V_max_ for polymerisation was observed. These V_max_ results show correlation with the antiproliferative data recorded for both CA-4 (IC_50_ = 4.2 nM) and **10p** (IC_50_ = 31 nM) in the MCF-7 line and indicate that tubulin is the molecular target for this series of 3-allyl-β-lactams. The most potent antiproliferative compound, 3-(prop-1-en-2-yl)azetidinone **9q** (IC_50_ = 10 nM), was also found to be a potent inhibitor of tubulin polymerization, demonstrating a 4.27-fold reduction in V_max_ value at 10 μM concentration, together with the ring B amino 3-allyl compound **10r** (3.4-fold reduction in V_max_ value) and the ring B methoxy-3-butadienyl-β-lactam **11h** (2.9-fold reduction in V_max_ value).

In a further investigation of the mechanism of action of the series of β-lactams, the interaction of the most potent antiproliferative compound, 3-(prop-1-en-2-yl)azetidinone **9q**, at the colchicine-binding site of tubulin was examined. The colchicine-binding site of tubulin is characterised by the Cys239 and Cys354 thiol-containing residues. The alkylating reagent N,N’-ethylene-bis(iodoacetamide) (EBI) reacts with the thiol-containing amino acid residues of cysteine 239 and cysteine 354 to crosslink [99,100].

**Figure 8:**
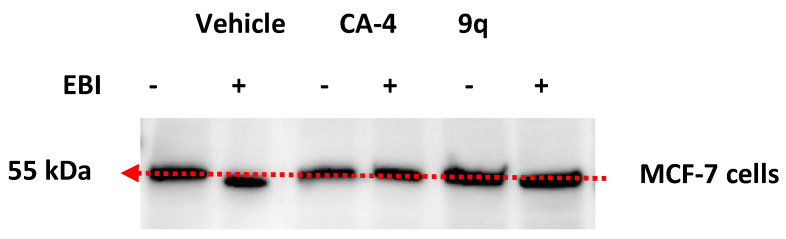
Colchicine-binding assay. Effect of β-lactam compound **9q** on the inhibition of bisthioalkylation of β-tubulin amino acids Cys239 and Cys354 by N,N’-ethylene-bis(iodoacetamide) (EBI). MCF-7 cells were treated with vehicle control (ethanol 0.1% (*v*/*v*)), **CA**-**4** and β-lactam **9q** (10 μM) for 2 h; samples were then treated with EBI for an additional 1.5 h. Cells were harvested, lysed and then analysed using sedimentation followed by Western blotting to identify β-tubulin and β-tubulin-EBI adduct.

In the present work, the β-lactam **9q** (10 μM) or CA-4 was used to treat MCF-7 cells for 2 h; EBI was then added, and the cells were incubated for 15 h (Figure 8). Vehicle-treated control samples were observed at a lower position on the gel, confirming the formation of the β-tubulin-EBI adduct and demonstrating that the alkylating reagent EBI had formed cross links on β-tubulin with the cysteine thiol residues Cys239 and Cys354. The formation of the EBI adduct was prevented when the MCF-7 cells were treated with β-lactam **9q** and also with CA-4, demonstrating that the β-lactam **9q** interacts with tubulin at the colchicine site of tubulin.

**Figure 9:**
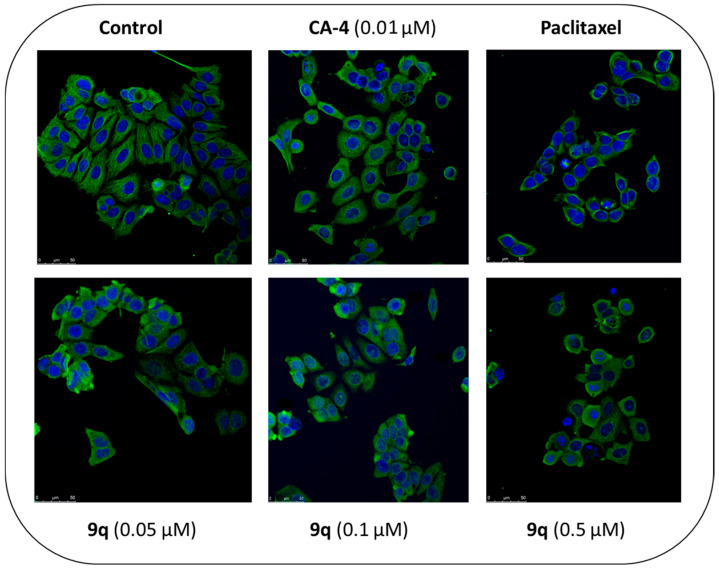
β-lactam compound **9q** induces depolymerization of the microtubule network in MCF-7 breast cancer cells.

Evidence of the effects of the β-lactam **9q** on the molecular structure of the target tubulin was investigated using immunofluorescence and confocal microscopy studies, through which the changes in the microtubule network structure produced by β-lactam **9q** in MCF-7 cells can be observed. MCF-7 cells demonstrated an organised microtubular network structure following staining with α-tubulin mAb (Figure 9) in the presence of the vehicle control (1% ethanol (*v*/*v*)). The cells were also treated with paclitaxel, which acts as a microtubule-stabilising agent [101]; paclitaxel clearly induced the formation of microtubule bundles and pseudo-asters. When the MCF-7 cells were treated with CA-4 or β-lactam **9q** for 16 h, microtubule formation was inhibited and resulted in depolymerised microtubules (Figure 9), with multiple micronuclei present in these cells. Mitotic catastrophe, characterised by the appearance of multinucleated cells, is a type of programmed cell death occurring during mitosis. It results from a combination of deficient cell-cycle checkpoints (in particular, the DNA structure checkpoints and the spindle assembly checkpoint) and also cellular damage [102]. Induction of mitotic catastrophe by CA-4 was previously reported in non-small-cell lung cancer and breast cancer cells (MCF-7) [103]. The confocal imaging results support the proposed tubulin-targeting action of the 3-(prop-1-en-2-yl)azetidinone **9q**.

CA-4 and β-lactam **9q** depolymerise the microtubule network in MCF-7 cells. The MCF-7 cells were treated with vehicle control (1% ethanol (*v*/*v*)), paclitaxel (1 μM), CA-4 (10 nM) top panels and β-lactam **9q** (0.05, 0.1 and 0.5 μM) bottom panels. After 16 h, the cells were fixed in 4% paraformaldehyde and stained with mouse monoclonal anti-α-tubulin−FITC antibody (clone DM1A) (green), Alexa Fluor 488 dye, and counterstained with DAPI (blue). The confocal images were obtained by Leica SP8 confocal microscopy with Leica application suite X software. Representative confocal micrograph images of three separate experiments are shown above [scale bar: 50 μM].

### 2.5. Computational Modelling of β-Lactam Compounds

The 3-(prop-1-en-2-yl)azetidin-2-one compound **9q** was identified as the most potent compound synthesised in this study (IC_50_ = 10 nM in MCF-7 breast cancer cells) and also as an inhibitor of tubulin polymerisation. The tubulin-binding and related immunofluorescence studies of **9q** indicated that the colchicine-binding site of tubulin is the target for the series of compounds. The structural similarity of the β-lactam compounds with CA-4 was demonstrated by X-ray studies revealing the similar torsional angle observed between rings A and B and a flexible alignment of the 3-butadienyl β-lactam compound **11p** with CA-4 (Supplementary Figure S22), which showed excellent overlap with the 3,4,5-trimethoxyphenyl ring A and phenolic ring B of both compounds. In a further comparison of the energy-minimised structures of compound **11p** and CA-4, the inter-atomic distances of the methoxy oxygens of ring A and ring B is 9.16 Å, while for CA-4, this distance is slightly longer (9.27 Å) (Supplementary Figure S23).

**Figure 10:**
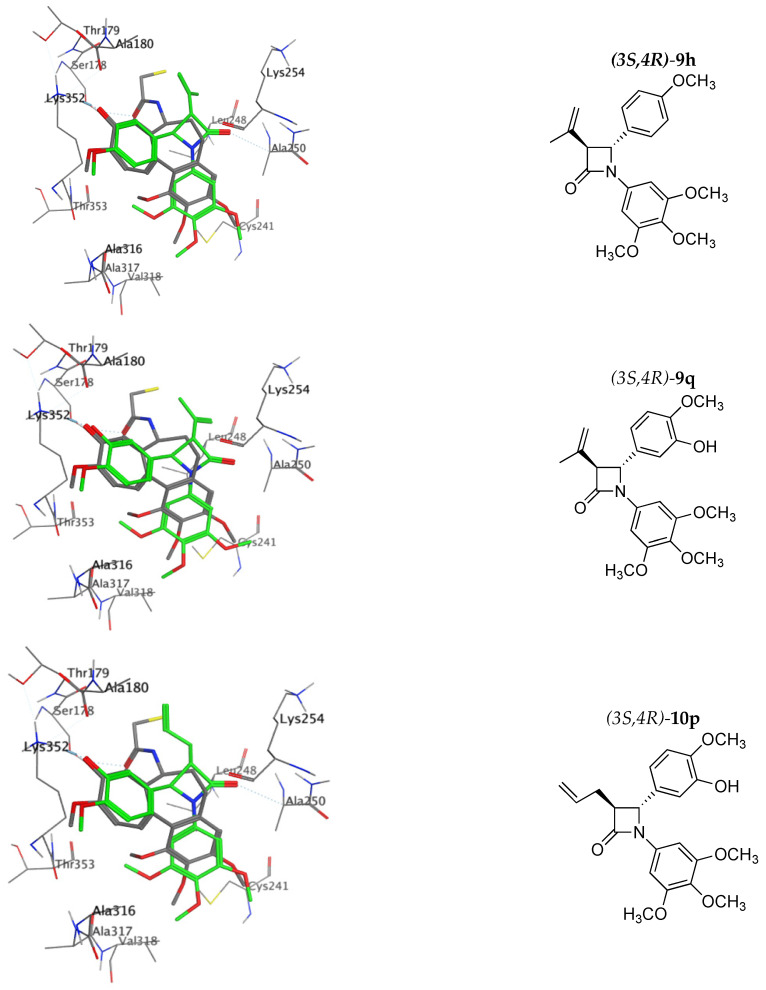

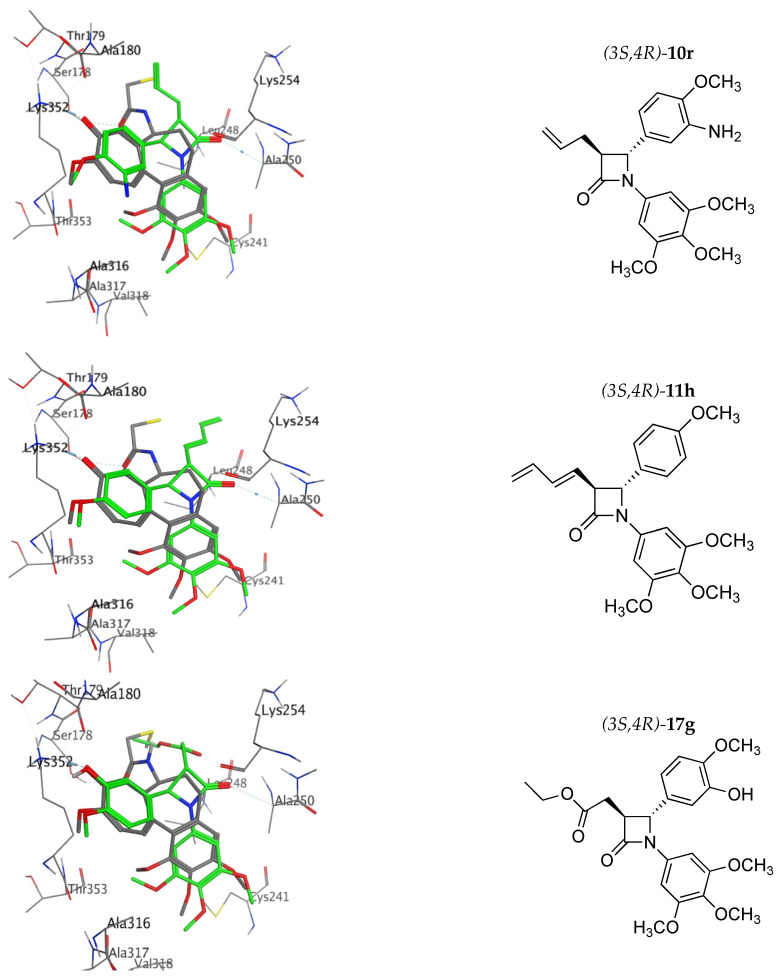
Overlay of the X-ray structure of tubulin co-crystallised with DAMA-colchicine (PDB entry 1SA0) on the best-ranked docked pose of the *3S*,*4R* enantiomer of **9h**, **9q**, **10p**, **10r**, **11h**, **17g**. Ligands are rendered as tubes and amino acids as lines. Tubulin amino acids and DAMA-colchicine are coloured by atom type: carbon = grey, hydrogen = white, oxygen = red, nitrogen = blue. The beta-lactam is depicted with a green backbone. The atoms are coloured by element type, key amino acid residues are labelled, and multiple residues are hidden to enable a clearer view.

Computational docking calculations using MOE 2022.02 [104] were undertaken for the most potent compounds identified (**9h, 9q, 9s, 10h, 10p, 10r, 11h, 11p, 11r** and **17d**) using the X-ray structure of bovine tubulin co-crystallised with N-deacetyl-N-(2-mercaptoacetyl)-colchicine (DAMA-colchicine) **1b** 1SA0 [18] (see Figure 10). ^1^H NMR spectroscopy confirmed that only the *trans* isomers of the compounds were obtained in the synthesis. All trimethoxy compounds overlaid their B-rings on the C-ring of DAMA-colchicine (forming HBA interactions with Lys352 and with the 4-methoxy group close to Thr353), co-located the 3,4,5-trimethoxyphenyl-substituted A-rings (with interactions possible with Cys241 and Ala326, Ala317 and Val318) and positioned the 3-alkenyl or ester side chain in an open region of the tubulin-binding site at the dimer interface (all amino acid residues refer to α-tubulin). The predicted docking ranking from best to worst was **17d, 11p, 9q, 11h, 9s, 9h, 10p, 10h, 11r** and **10r** (see Supplementary Materials, Table S11).

Although the compounds were biologically evaluated as racemates, it was interesting that the 3*S*,4*R* enantiomer of each compound was found to be ranked at lower energy in the docking study than the corresponding 3*R*,4*S* enantiomer. We had previously reported stereochemical selectivity in docking energy calculated for related β-lactam compounds [54,105]. However, a very small difference was observed in the cellular efficacy of this series of compounds in the modelling study (e.g., IC_50_ values in the range 10–61 nM), so it would not be expected to see a large difference in ranking. Indeed, the docking scores only differ by <0.8 from best- to worst-ranked (see Supplementary Materials, Table S11). Thus, docking studies are not ideal for studying changes in cellular efficacy associated with small changes in the β-lactam scaffold substituents located at C-3.

The top three ranked compounds, 3-acetate ester **17e**, 3-butadienyl-β-lactam **11p** and 3-(prop-1-en-2-yl)-β-lactam **9q**, all contained a *meta*-hydroxyl substituent on the B ring. Figure 10 shows the best docking pose of the top ranked phenolic compounds, **9q, 10p, 11p** and **17e**. For the 3-(prop-1-en-2-yl) compound **9s**, the HBA interaction of the ring B amino group with Lys252 is clearly observed, while for the ring B methoxy compound **11h**, the interaction of this methoxy group with the Thr353 residue is also illustrated, together with the β-lactam carbonyl interaction with Ala250 and Leu β248 (hydrophobic), which are residues of the T7 loop H8 helix, observed for all β-lactam compounds and also for colchicine. Although all molecules are located slightly deeper in the binding pocket than DAMA-colchicine, nevertheless, they demonstrate the observed ligand–protein interactions. (See Supplementary Materials, Figures S24–S26 for additional molecular modelling illustrations for compounds **9h**, **9q**, **9s**, **10h**, **11p** and **11r**).

**Figure 11:**
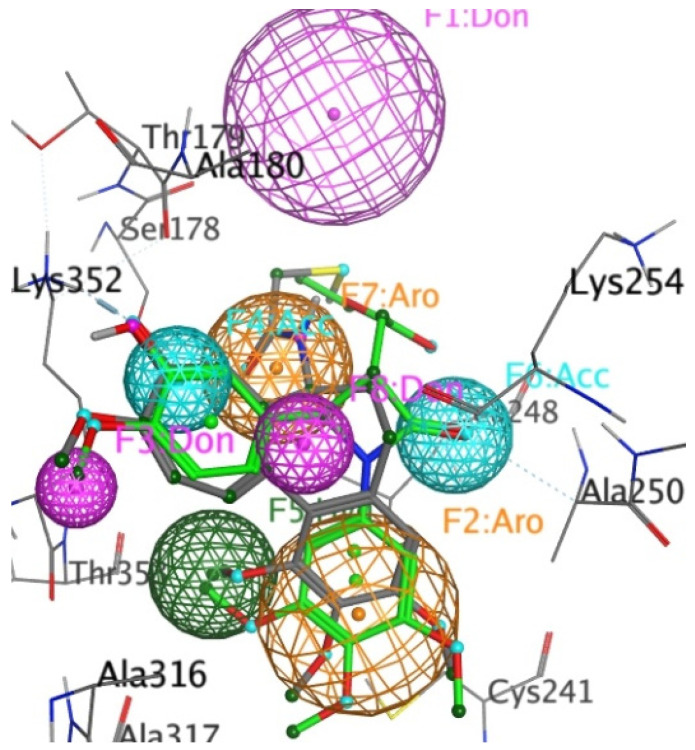
Mapping of the X-ray structure of tubulin co-crystallised with DAMA-colchicine (PDB entry 1SA0) and the best ranked docked pose of the *3S,4R* enantiomer **17g**, with a pharmacophore created by MoPBS. The ligand and protein colouring is explained in the legend above. The pharmacophore feature colours are HBA, light blue; HBD, pink; aromatic, orange; and hydrophobic, green.

We recently developed a novel pharmacophore generation tool, MoPBS [106]. In brief, MoPBS floods the protein-binding site with fragments describing classic molecular interaction features (acetate ion, benzene, methane and methylammonium) and independently minimising their positions within the binding site. Each fragment clusters in the region of complementary amino acids, thereby mapping out preferred interaction locations. By applying K-means clustering algorithms, we, in this case, reduce the clusters to eight pharmacophore features. Application of this tool to the 1SA0 binding site yielded a pharmacophore shown in Figure 11. As expected, many of the features mapped to those present in DAMA-colchicine and **17g**, such as the two aromatic cores and the hydrogen bond acceptor (HBA) interacting with Lys352. Interestingly, a unique feature is present in the β-lactam, in that the carbonyl oxygen atom maps to a HBA feature. The pharmacophores also point towards future synthetic possibilities, such as introducing a hydrogen bond donor (F3 HBD) in place of the methoxy group on the compound **17g** B-ring, including more hydrophobicity off the trimethoxy phenyl ring (F5) or extending the compound towards the region of space occupied by the F1 HBD feature.

## 3. Experimental Section

### 3.1. Materials and Methods: Chemistry

Melting points were measured on a Gallenkamp SMP 11 melting point apparatus and are uncorrected. Infrared (IR) spectra were recorded as thin film on NaCl plates or as potassium bromide discs on a Perkin Elmer FT-IR Spectrum 100 spectrometer. ^1^H and ^13^C nuclear magnetic resonance (NMR) spectra were recorded at 27 °C on a Bruker Avance DPX 400 spectrometer (400.13 MHz, ^1^H; 100.61 MHz, ^13^C) at 20 °C in CDCl_3_ (internal standard tetramethylsilane TMS) or DMSO-d_6_ by Dr. John O’Brien and Dr. Manuel Ruether, School of Chemistry, Trinity College Dublin. For CDCl_3_, ^1^H-NMR spectra were assigned relative to the TMS peak at 0.00 δ and ^13^C-NMR spectra were assigned relative to the middle CDCl_3_ triplet at 77.00 ppm. Electrospray ionisation mass spectrometry (ESI-MS) on a liquid chromatography time-of-flight (TOF) mass spectrometer (Micromass LCT, Waters Ltd., Manchester, UK) equipped with electrospray ionization (ES) interface operated in the positive ion mode with high-resolution mass measurement accuracies of <±5 ppm. R_f_ values are quoted for thin-layer chromatography on silica gel Merck F-254 plates. Flash column chromatography was carried out on Merck Kieselgel 60 (particle size 0.040–0.063 mm) and also on Biotage SP4 instruments. All products isolated were homogenous on TLC. Analytical high-performance liquid chromatography (HPLC) for purity determination of products was performed using a Waters 2487 Dual Wavelength Absorbance detector, Waters 1525 binary HPLC pump, Waters In-Line Degasser AF, Waters 717plus Autosampler and Varian Pursuit XRs C18 reverse-phase 150 × 4.6 mm chromatography column with detection at 254 nm. Imines **8a**-**w** and azetidine-2-ones **16a**-**c** and **18** were prepared following the reported procedures [52,53].

### 3.2. General Method I: Preparation of 3-(Prop-1-en-2-yl)-2-azetidinones (***9a***-***x***)

To a stirring, refluxing solution of imine (5 mmol) and triethylamine (6 mmol) in anhydrous dichloromethane (40 mL), a solution of 3,3-dimethylacryloyl chloride (6 mmol) in anhydrous dichloromethane (10 mL) was added dropwise over 45 min under nitrogen. The reaction was then heated at reflux for 5–8 h and stirred at 20 °C for 16 h. The reaction mixture was washed with water (2 × 100 mL), organic extract was dried over anhydrous sodium sulphate, and the solvent was removed under reduced pressure. The crude product was purified by flash chromatography over silica gel (eluent: *n*-hexane:ethyl acetate, 4:1).

4-(4-Nitrophenyl)-1-(3,4,5-trimethoxyphenyl)-3-(prop-1-en-2-yl)azetidin-2-one (**9a**)

Preparation as described in general method I above from 3,3-dimethylacryloyl chloride and (4-nitrobenzylidene)-3,4,5-trimethoxyphenylamine (**8a**) afforded the product as a yellow solid, yield 24%, Mp 129–130 °C (HPLC: 80.0%). IR (KBr) ν_max_: 2952 (C-H), 1754 (C=O, β-lactam), 1587 (C=C), 1506 (NO_2_), 1344 (NO_2_), 1236 (C-O) cm^−1^. ^1^H NMR (400 MHz, CDCl_3_): δ 1.92 (s, 3H), 3.72 (d, *J* = 2.00 Hz, 1H), 3.74 (s, 6H), 3.79 (s, 3H), 4.95 (d, *J* = 2.48 Hz, 1H), 5.09 (br s, 1H), 5.10 (br s, 1H), 6.52 (s, 2H), 7.57 (d, *J* = 9.04 Hz, 2H), 8.20–8.28 (m, 2H). ^13^C NMR (100 MHz, CDCl_3_): δ 19.99, 55.73, 60.52, 60.61, 66.64, 94.16, 115.08, 124.17, 126.24, 132.68, 134.45, 136.81, 146.18, 148.79, 153.29, 153.24, 163.76 (C=O). HRMS (*m*/*z*) calculated for C_21_H_23_N_2_O_6_ (M^+^+H): 399.1556, found 399.1556.

4-(4-Chlorophenyl)-1-(3,4,5-trimethoxyphenyl)-3-(prop-1-en-2-yl)azetidine-2-one (**9b**)

Preparation as described in general method I above from 3,3-dimethylacryloyl chloride and (4-chlorobenzylidene)-(3,4,5-trimethoxyphenyl)amine (**8b**) afforded the product as a yellow solid, yield 24%, Mp 137–138 °C (HPLC: 97.9%). IR (KBr) ν_max_: 2987 (C-H), 1746 (C=O, β-lactam), 1586 (C=C), 1503 (C=C), 1235 (C-O) cm^−1^. ^1^H NMR (400 MHz, CDCl_3_): δ 1.90 (s, 3H), 3.70 (d, *J* = 2.00 Hz, 1H), 3.74 (s, 6H), 3.79 (s, 3H), 4.80 (d, *J* = 2.48 Hz, 1H), 5.05 (br s, 1H), 5.09 (br s, 1), 6.54 (s, 2H), 7.33 (d, *J* = 8.52 Hz, 2H), 7.40 (d, *J* = 8.52 Hz, 2H). ^13^C NMR (100 MHz, CDCl_3_): δ 20.54, 55.60, 59.38, 60.52, 66.51, 94.16, 114.41, 126.77, 129.07, 133.03, 133.89, 134.05, 135.81, 137.30, 153.12, 164.39. HRMS (*m*/*z*) calculated for C_21_H_22_^35^ClNO_4_Na (M^+^+Na): 410.1135, found 410.1133.

4-(4-Bromophenyl)-1-(3,4,5-trimethoxyphenyl)-3-(prop-1-en-2-yl)azetidine-2-one (**9c**)

Preparation as described in general method I above from 3,3-dimethylacryloyl chloride and (4-bromobenzylidene)-3,4,5-trimethoxyphenylamine (**8c**) afforded the product as yellow solid, yield 19%, Mp 130–131 °C (HPLC: 97.6%). IR (KBr) ν_max_: 2940 (C-H), 1749 (C=O, β-lactam), 1585 (C=C), 1502 (C=C), 1235 (C-O) cm^−1^. ^1^H NMR (400 MHz, CDCl_3_): δ 1.90 (s, 3H), 3.70 (br s, 1H), 3.75 (s, 6H), 3.79 (s, 3H), 4.80 (d, *J* = 1.84 Hz, 1H), 5.03 (br s, 1H), 5.09 (br s, 1H), 6.54 (s, 2H), 7.28 (d, *J* = 7.92 Hz, 2H), 7.56 (d, *J* = 8.56 Hz, 2H). ^13^C NMR (100 MHz, CDCl_3_): δ 20.54, 56.07, 59.88, 60.97, 66.94, 94.65, 114.89, 122.58, 127.54, 132.48, 133.48, 134.62, 136.82, 137.76, 153.60, 164.83. HRMS (*m*/*z*) calculated for C_21_H_23_^79^BrNO_4_ (M^+^+H): 432.0810, found 432.0832.

4-(4-Fluorophenyl)-1-(3,4,5-trimethoxyphenyl)-3-(prop-1-en-2-yl)azetidin-2-one (**9d**)

Preparation as described in the general method I above from 3,3-dimethylacryloyl chloride and (4-fluorobenzylidene)-3,4,5-trimethoxyphenylamine (**8d**) afforded the product as colourless crystals, yield 14%, Mp 99–100 °C (HPLC: 97.9%). IR (KBr) ν_max_: 2941 (C-H), 1746 (C=O, β-lactam), 1585 (C=C), 1508 (C=C), 1228 (C-O) cm^−1^. ^1^H NMR (400 MHz, CDCl_3_): δ 1.89 (s, 3H), 3.71 (d, *J* = 2.00 Hz, 1H), 3.73 (s, 6H), 3.78 (s, 3H), 4.81 (d, *J* = 2.52 Hz, 1H), 5.04 (br s, 1H), 5.09 (br s, 1H), 6.54 (s, 2H), 7.09–7.13 (m, 2H), 7.36–7.39 (m, 2H). ^13^C NMR (100 MHz, CDCl_3_): δ 20.10, 55.56, 59.42, 60.50, 66.55, 94.17, 114.27, 115.77, 115.98, 127.14, 127.23, 133.03, 133.10, 134.04, 137.41, 153.09, 161.05, 164.50. HRMS (*m*/*z*) calculated for C_21_H_22_FNO_4_Na (M^+^+Na): 394.1431, found 394.1443.

4-(4-Dimethylaminophenyl)-1-(3,4,5-trimethoxyphenyl)-3-(prop-1-en-2-yl)azetidine-2-one (**9e**)

Preparation as described in general method I above from 3,3-dimethylacryloyl chloride and (4-(dimethylamino)benzylidene)-3,4,5-trimethoxyphenylamine (**8e**) afforded the product as a brown oil, yield 34% (HPLC: 92.4%). IR (NaCl) ν_max_: 2942 (C-H), 1742 (C=O, β-lactam), 1597 (C=C), 1586 (C=C), 1506 (C=C), 1234 (C-O) cm^−1^. ^1^H NMR (400 MHz, CDCl_3_): δ 1.88 (s, 3H), 2.98 (s, 6H), 3.74 (s, 6H), 3.75 (d, *J* = 2.00 Hz, 1H), 3.77 (s, 3H), 4.73 (d, *J* = 2.44 Hz, 1H), 5.00 (br s, 1H), 5.07 (br s, 1H), 6.61 (s, 2H), 6.74 (d, *J* = 6.84 Hz, 2H), 7.27 (d, *J* = 9.28 Hz, 2H). ^13^C NMR (100 MHz, CDCl_3_): δ 20.17, 40.05, 55.53, 60.22, 60.48, 66.28, 94.23, 112.34, 113.70, 126.64, 133.51, 133.73, 134.78, 137.95, 140.56, 152.95, 165.19. HRMS (*m*/*z*) calculated for C_23_H_29_N_2_O_4_ (M^+^+H): 397.2127; found 397.2124.

4-Phenyl-1-(3,4,5-trimethoxyphenyl)-3-(prop-1-en-2-yl)azetidin-2-one (**9f**)

Preparation as described in general method I above from 3,3-dimethylacryloyl chloride and benzylidene-(3,4,5-trimethoxyphenyl)amine (**8f**) afforded the product as colourless crystals, yield 24%, Mp 108–109 °C (HPLC: 99.9%). IR (KBr) ν_max_: 2934 (C-H), 1746 (C=O, β-lactam), 1587 (C=C), 1506, 1237 cm^−1^. ^1^H NMR (400 MHz, CDCl_3_): δ 1.91 (s, 3H), 3.72 (s, 6H), 3.76 (d, *J* = 2.48 Hz, 1H), 3.78 (s, 3H), 4.82 (d, *J* = 2.48 Hz, 1H), 5.04 (br s, 1H), 5.10 (br s, 1H), 6.57 (s, 2H), 7.35–7.44 (m, 5H). ^13^C NMR (100 MHz, CDCl_3_): δ 20.60, 55.99, 60.64, 60.94, 66.84, 94.70, 114.55, 125.97, 128.70, 129.27, 133.76, 134.33, 137.70, 138.10, 153.51, 165.16. HRMS (*m*/*z*) calculated for C_21_H_23_NO_4_Na (M^+^+Na): 376.1525, found 376.1522.

4-*p*-Tolyl-1-(3,4,5-trimethoxyphenyl)-3-(prop-1-en-2-yl)azetidine-2-one (**9g**)

Preparation as described in general method I above from 3,3-dimethylacryloyl chloride and (4-methylbenzylidene)-(3,4,5-trimethoxyphenyl)amine (**8g**) afforded the product as a colourless solid, yield 12%, Mp 102–104 °C (HPLC: 99.9%). IR (KBr) ν_max_: 2938 (C-H), 1746 (C=O, β-lactam), 1587 (C=C), 1505 (C=C), 1236 (C-O) cm^−1^. ^1^H NMR (400 MHz, CDCl_3_): δ 1.89 (s, 3H), 2.38 (s, 3H), 3.73 (s, 7H), 3.78 (s, 3H), 4.78 (d, *J* = 2.52 Hz, 1H), 5.02 (br s, 1H), 5.08 (br s, 1H), 6.57 (s, 2H), 7.22 (d, *J* = 8.04 Hz, 2H), 7.29 (d, *J* = 8.04 Hz, 2H). ^13^C NMR (100 MHz, CDCl_3_): δ 20.15, 20.77, 55.54, 60.04, 60.49, 66.40, 94.20, 113.97, 125.45, 129.46, 133.34, 133.87, 134.16, 137.71, 138.10, 153.01, 164.83. HRMS (*m*/*z*) calculated for C_22_H_25_NO_4_Na (M^+^+Na): 390.1681, found 390.1680.

4-(4-Methoxyphenyl)-1-(3,4,5-trimethoxyphenyl)-3-(prop-1-en-2-yl)azetidin-2-one (**9h**)

Preparation as described in general method I above from 3,3-dimethylacryloyl chloride and (4-methoxybenzylidene)-3,4,5-trimethoxyphenylamine (**8h**) afforded the product as a yellow solid, yield 40%, Mp 103–105 °C (HPLC: 87.1%). [80] IR (KBr) ν_max_: 2995 (C-H), 1744 (C=O, β-lactam), 1588 (C=C), 1508 (C=C), 1249 (C-O) cm^−1^. ^1^H NMR (400 MHz, CDCl_3_): δ 1.89 (s, 3H), 3.73 (s, 7H, OCH_3_), 3.78 (s, 3H), 3.83 (s, 3H), 4.77 (d, *J* = 2.52 Hz, 1H), 5.02 (br s, 1H), 5.08 (br s, 1H), 6.57 (s, 2H), 6.94 (d, *J* = 8.52 Hz, 2H), 7.32 (d, *J* = 8.52 Hz, 2H). ^13^C NMR (100 MHz, CDCl_3_): δ 20.14, 54.88, 55.53, 59.85, 60.49, 66.46, 94.19, 113.94, 114.15, 126.80, 129.04, 133.33, 133.85, 137.71, 153.00, 159.36, 164.87 (C=O). HRMS (*m*/*z*) calculated for C_22_H_25_NO_5_Na (M^+^+Na): 406.1630, found 406.1618.

4-(4-Ethoxyphenyl)-1-(3,4,5-trimethoxyphenyl)-3-(prop-1-en-2-yl)azetidin-2-one (**9i**)

Preparation as described in general method I above from 3,3-dimethylacryloyl chloride and (4-ethoxybenzylidene)-(3,4,5-trimethoxyphenyl)amine (**8i**) afforded the product as a colourless solid, yield 12%, Mp 100–102 °C [80] (HPLC: 83.9%). IR (KBr) ν_max_: 2984 (C-H), 1745 (C=O, β-lactam), 1591 (C=C), 1587 (C=C), 1509 (C=C), 1240 (C-O) ^−1^. ^1^H NMR (400 MHz, CDCl_3_): δ 1.44 (t, *J* = 4.64 Hz, 3H), 1.90 (s, 3H), 3.74 (s, 7H), 3.78 (s, 3H), 4.05 (q, *J* = 4.88 Hz, 2H), 4.77 (d, *J* = 1.48 Hz, 1H), 5.03 (br s, 1H), 5.09 (br s, 1H), 6.58 (s, 2H), 6.93 (d, *J* = 5.88 Hz, 2H), 7.29–7.32 (m, 2H). ^13^C NMR (100 MHz, CDCl_3_): δ 14.62, 20.43, 55.86, 60.24, 60.76, 63.42, 66.77, 94.65, 114.14, 115.00, 127.08, 129.23, 133.68, 134.31, 138.07, 153.34, 159.10, 165.16. HRMS (*m*/*z*) calculated for C_23_H_27_NO_5_Na (M^+^+Na): 420.1787, found 420.1772.

4-(4-Butoxyphenyl)-1-(3,4,5-trimethoxyphenyl)-3-(prop-1-en-2-yl)azetidine-2-one (**9j**)

Preparation as described in general method I above from 3,3-dimethylacryloyl chloride and (4-butoxybenzylidene)-3,4,5-trimethoxyphenylamine (**8j**) afforded the product as an off-white solid, yield 7%, Mp 55–57 °C (HPLC: 99.9%). IR (KBr) ν_max_: 2936 (C-H), 1747 (C=O, β-lactam), 1603 (C=C), 1587 (C=C), 1509 (C-C), 1243 (C-O) cm^−1^. ^1^H NMR (400 MHz, CDCl_3_): δ 0.99 (t, J = 7.28Hz, 3H), 1.49–1.51 (m, 2H), 1.76–1.80 (m, 2H), 1.89 (s, 3H), δ 3.73 (s, 7H), 3.78 (s, 3H), 3.97 (t, *J* = 6.28 Hz, 2H), 4.76 (d, *J* = 2.48Hz, 1H), 5.02 (br s, 1H), 5.08 (br s, 1H), 6.57 (s, 2H), 6.92 (d, *J* = 8.52 Hz, 2H), 7.31 (d, *J* = 9.04 Hz, 2H). ^13^C NMR (100 MHz, CDCl_3_): δ 13.40, 18.78, 20.15, 30.80, 55.54, 59.90, 60.49, 66.45, 67.33, 94.20, 113.91, 114.67, 126.77, 128.78, 133.36, 133.84, 137.75, 153.30, 158.98, 164.90. HRMS (*m*/*z*) calculated for C_25_H_31_NO_5_Na (M^+^+Na): 448.2100, found 448.2106.

4-(4-Phenoxyphenyl)-1-(3,4,5-trimethoxyphenyl)-3-(prop-1-en-2-yl)azetidin-2-one (**9k**)

Preparation as described in general method I above from 3,3-dimethylacryloyl chloride and (4-phenoxylbenzylidene)-(3,4,5-trimethoxyphenyl)amine (**8k**) afforded the product as a brown solid, yield 20%, Mp 126–127 °C (HPLC: 89.1%). IR (KBr) ν_max_: 2938 (C-H), 1748 (C=O, β-lactam), 1587 (C=C), 1507 (C=C), 1239 (C-O) cm^−1^. ^1^H NMR (400 MHz, CDCl_3_): δ 1.91 (s, 3H), 3.75 (s, 7H), 3.79 (s, 3H), 4.81 (d, *J* = 2.52 Hz, 1H), 5.04 (br s, 1H), 5.10 (br s, 1H), 6.58 (s, 2H), 7.02–7.05 (m, 4H), 7.14–7.17 (m, 1H), 7.35–7.39 (m, 4H). ^13^C NMR (100 MHz, CDCl_3_): δ 20.15, 55.57, 59.69, 60.52, 66.45, 94.24, 114.13, 118.72, 118.80, 123.38, 127.00, 129.44, 131.64, 133.24, 133.99, 137.59, 153.07, 156.09, 157.36, 164.71. HRMS (*m/z*) calculated for C_27_H_27_NO_5_Na (M^+^+Na): 468.1787, found 468.1786.

4-(4-Benzyloxyphenyl)-1-(3,4,5-trimethoxyphenyl)-3-(prop-1-en-2-yl)azetidin-2-one (**9l**)

Preparation as described in general method I above from 3,3-dimethylacryloyl chloride and (4-benzyloxybenzylidene)-3,4,5-trimethoxyphenylamine (**8l**) afforded the product as a yellow green resin, yield 35% (HPLC: 82.4%). IR (NaCl) ν_max_: 2976 (C-H), 1744 (C=O, β-lactam), 1587 (C=C), 1508 (C=C), 1275 (C-O) cm^−1^. ^1^H NMR (400 MHz, CDCl_3_): δ 1.89 (s, 3H), 3.73 (s, 7H), 3.78 (s, 3H), 4.78 (d, *J* = 2.48 Hz, 1H), 5.03 (br s, 1H), 5.09 (br s, 3H), 6.57 (s, 2H), 7.02 (d, *J* = 8.56 Hz, 2H), 7.31–7.43 (m, 7H). ^13^C NMR (100 MHz, CDCl_3_): δ 20.16, 55.55, 59.84, 60.51, 66.43, 69.61, 94.20, 113.98, 115.09, 126.85, 127.03, 127.68, 128.19, 129.33, 133.33, 133.86, 136.14, 137.70, 153.01, 158.53, 164.85. HRMS (*m/z*) calculated for C_28_H_29_NO_5_Na (M^+^+Na): 482.1943, found 482.1937.

4-Naphthalen-1-yl-1-(3,4,5-trimethoxyphenyl)-3-(prop-1-en-2-yl)azetidin-2-one (**9m**)

Preparation as described in general method I above from 3,3-dimethylacryloyl chloride and naphthalen-1-ylmethylene-(3,4,5-trimethoxyphenyl)amine (**8m**) afforded the product as a brown oil, yield 51% (HPLC: 86.8%). IR (NaCl) ν_max_: 2984(C-H), 1749 (C=O, β-lactam), 1588 (C=C), 1506 (C=C), 1235 (C-O) cm^−1^. ^1^H NMR (400 MHz, CDCl_3_): δ 2.02 (s, 3H), 3.70 (s, 6H), 3.81 (s, 4H), 5.11 (br s, 1H), 5.18 (br s, 1H), 5.66 (d, *J* = 2.00 Hz, 1H), 6.65 (s, 2H), 7.45–8.03 (m, 7H). ^13^C NMR (100 MHz, CDCl_3_): δ 19.62, 55.47, 55.66, 60.52, 66.59, 94.40, 116.16, 122.20, 122.58, 124.44, 125.11, 125.70, 126.25, 128.19, 128.73, 129.99, 133.41, 133.47, 134.06, 137.75, 153.15, 164.88. HRMS (*m/z*) calculated for C_25_H_25_NO_4_Na (M^+^+Na): 426.1681, found 426.1687.

4-Naphthalen-2-yl-1-(3,4,5-trimethoxyphenyl)-3-(prop-1-en-2-yl)azetidin-2-one (**9n**)

Preparation as described in general method I above from 3,3-dimethylacryloyl chloride and naphthalen-2-ylmethylene-(3,4,5-trimethoxyphenyl)amine (**8n**) afforded the product as colourless solid, yield 15%, Mp 123–124 °C (HPLC: 98.8%). IR (KBr) ν_max_: 2978 (C-H), 1746 (C=O, β-lactam), 1586 (C=C), 1505 (C=C), 1235 (C-O) cm^−1^. ^1^H NMR (400 MHz, CDCl_3_): δ 1.94 (s, 3H), 3.69 (s, 6H), 3.77 (s, 3H), 3.83 (d, *J* = 2.00 Hz, 1H), 5.00 (d, *J* = 3.00 Hz, 1H), 5.06 (br s, 1H), 5.12 (br s, 1H), 6.63 (s, 2H), 7.48–7.87 (m, 7H). ^13^C NMR (100 MHz, CDCl_3_): δ 20.17, 55.56, 60.33, 60.49, 66.47, 94.23, 114.25, 122.51, 124.95, 126.11, 126.32, 127.39, 127.42, 129.02, 132.88, 132.92, 133.39, 134.00, 134.73, 137.59, 153.08, 164.75. HRMS (*m/z*) calculated for C_25_H_25_NO_4_Na (M^+^+Na): 426.1681, found 426.1674.

4-(4-(Methylthio)phenyl)-3-(prop-1-en-2-yl)-1-(3,4,5-trimethoxyphenyl)azetidin-2-one (**9o**)

Preparation as described in general method I above from imine (**8o)** and 3,3-dimethylacryloyl chloride afforded the product as a yellow powder, yield 56%, Mp 99–100 °C [80] (HPLC: 99.1%). IR ν_max_: 1741.7 (C=O) cm^−1^. ^1^H NMR (400 MHz, CDCl_3_): δ 1.85 (s, 3 H), 2.46 (s, 3 H), 3.66–3.69 (m, 1 H), 3.69 (s, 6 H), 3.75 (s, 3 H), 4.74 (d, *J*=2.44 Hz, 1 H), 4.99–5.07 (m, 2 H), 5.60–5.91 (m, 1 H), 6.53 (s, 2 H), 7.24–7.29 (m, 4 H). ^13^C NMR (100 MHz, CDCl_3_): δ 15.09, 20.13, 55.60, 59.78, 60.51, 66.44, 94.22, 114.16, 125.44, 126.47, 133.81, 137.56, 138.93, 153.07, 164.68. HRMS (*m/z*) calculated for C_22_H_25_KNO_4_S [M^+^+K]: 438.1141; found 438.1141.

4-(3-Hydroxy-4-methoxyphenyl)-1-(3,4,5-trimethoxyphenyl)-3-(prop-1-en-2-yl)azetidin-2-one (**9q**)

Following the general method I above, a solution of the protected TBDMS imine **8p** (5 mmol) and triethylamine (6 mmol) in anhydrous dichloromethane (40 mL) was treated with a solution of 3,3-dimethylacryloyl chloride (6 mmol) in anhydrous dichloromethane (10 mL) to afford the intermediate β-lactam (**9p**), which was immediately deprotected. To a stirring solution of **9p** (5 mmol) under nitrogen at 0 ^0^C in dry THF was added dropwise t-BAF solution (1.0 M) in hexanes (5 mL, 5 mmol). The resulting solution was left to stir at 0 °C until reaction was completed by TLC. The reaction mixture was then diluted with ethyl acetate (75 mL) and washed with aqueous HCl (0.1M, 100 mL). Following repeated extraction with ethyl acetate (2 × 25 mL), the organic layers were combined and washed with water (100 mL) and saturated brine (100 mL) and dried over Na_2_SO_4_. The solvent was removed under reduced pressure to yield the desired product as a brown solid, which was recrystallised from ethanol, yield 22%, Mp 82–84 °C (HPLC: 88.9%). IR (KBr) ν_max_: 3390 (OH), 1740 (C=O, β-lactam), 1593 (C=C), 1508 (C=C), 1275 (C-O) cm^−1^. ^1^H NMR (400 MHz, CDCl_3_): δ 1.88 (s, 3H), 3.72 (d, *J* = 2.00 Hz, 1H), 3.74 (s, 6H), 3.78 (s, 3H), 3.91 (s, 3H), 4.71 (d, *J* = 2.52 Hz, 1H), 5.01 (br s, 1H), 5.07 (br s, 1H), 5.76 (br s, 1H), 6.58 (s, 2H), 6.85–6.96 (m, 3H). ^13^C NMR (100 MHz, CDCl_3_): δ 20.17, 55.55, 55.58, 59.87, 60.50, 66.37, 94.24, 110.52, 111.54, 113.93, 117.28, 130.30, 133.32, 133.88, 137.70, 145.81, 146.31, 153.01, 164.81. HRMS (*m/z*) calculated for C_22_H_25_NO_6_Na (M^+^+Na): 422.1580, found 422.1592.

4-(4-Methoxy-3-nitrophenyl)-1-(3,4,5-trimethoxyphenyl)-3-(prop-1-en-2-yl)azetidin-2-one (**9r**)

Preparation as described following the general method I above from 3,3-dimethylacryloyl chloride and (4-methoxy-3-nitrobenzylidene)-(3,4,5-trimethoxyphenyl) amine (**8r**) afforded the product as a brown oil, yield 19% (HPLC: 75.5%). IR (NaCl) ν_max_: 2940 (C-H), 1751 (C=O, β-lactam), 1535 (NO_2_), 1507 (C=C), 1281 (NO_2_) cm^−1^. ^1^H NMR (400 MHz, CDCl_3_): δ 1.90 (s, 3H), 3.73 (d, *J* = 2.24 Hz, 1H), 3.76 (s, 6H), 3.79 (s, 3H), 4.00 (s, 3H), 4.84 (d, *J* = 2.76 Hz, 1H), 5.07 (br s, 1H), 5.09 (br s, 1H), 6.54 (s, 2H), 7.16 (d, *J* = 8.76 Hz, 1H), 7.55–7.58 (m, 1H), 7.91 (d, *J* = 2.48 Hz, 1H). ^13^C NMR (100 MHz, CDCl_3_): δ 20.00, 55.70, 56.26, 58.54, 60.50, 66.56, 94.31, 114.17, 114.74, 123.12, 129.69, 130.67, 132.74, 134.45, 136.96, 139.42, 152.60, 153.26, 164.11. HRMS (*m/z*) calculated for C_22_H_24_N_2_O_7_Na (M^+^+Na): 451.1481, found 451.1481.

4-(3-Amino-4-methoxyphenyl)-1-(3,4,5-trimethoxyphenyl)-3-(prop-1-en-2-yl)azetidin-2-one (**9s**)

To a mixture of 3-(prop-1-en-2-yl)-4-(4-methoxy-3-nitrophenyl)-1-(3,4,5-trimethoxyphenyl)azetidin-2-one (**9r**) (0.25 mmol) and zinc powder 10 μm (2.5 mmol) was added acetic acid (15 mL), and the reaction mixture was stirred for 7 days at 20 °C under nitrogen. The mixture was filtered through a celite pad, and solvent was removed under reduced pressure. Purification by flash chromatography over silica gel (elutant ethyl acetate-*n*-hexane, 1:1) yielded the title compound as a yellow oil, yield 48% (HPLC: 91.6%). IR (NaCl) ν_max_: 3376 (NH_2_), 1726 (C=O, β-lactam)**,** 1594 (C=C), 1509 (C=C), 1237 (C-O) cm^−1^. ^1^H NMR (400 MHz, CDCl_3_): δ 1.87 (s, 3H), 3.72 (br s, 1H), 3.74 (s, 6H), 3.78 (s, 3H), 3.87 (s, 3H), 4.67 (d, *J* = 2.52 Hz, 1H), 5.00 (br s, 1H), 5.06 (br s, 1H), 6.60 (s, 2H), 6.74–6.78 (m, 3H). ^13^C NMR (100 MHz, CDCl_3_): δ 20.16, 55.11, 55.56, 60.11, 60.49, 66.31, 94.21, 109.99, 111.31, 113.82, 116.09, 129.66, 133.47, 133.80, 136.13, 137.84, 147.14, 152.98, 165.02. HRMS (*m/z*) calculated for C_22_H_27_N_2_O_5_ (M^+^+H): 399.1920, found 399.1900.

1-(4-(Methylthio)phenyl)-3-(prop-1-en-2-yl)-4-(3,4,5-trimethoxyphenyl)azetidin-2-one (**9t**)

Preparation following the general method I above from imine **8s** and dimethylacryloyl chloride afforded the product as a yellow powder, yield 45%, Mp 113–115 °C (HPLC: 96%). IRν_max_ (ATR): 1737.1 (C=O, β-lactam) cm^−1^. ^1^H NMR (400 MHz, CDCl_3_): δ ppm 1.90 (s, 3 H), 2.45 (s, 3 H), 3.72–3.77 (m, 1 H), 3.81–3.89 (m, 9 H), 4.74 (d, *J* = 2.51 Hz, 1 H), 5.04 (s, 1 H), 5.09 (s, 1 H), 6.55 (s, 2 H), 7.19 (d, *J* = 8.53 Hz, 2 H), 7.28 (d, *J* = 8.53 Hz, 2 H). ^13^C NMR (100 MHz, CDCl_3_): δ 16.08, 20.07, 55.78, 60.12, 60.41, 66.67, 102.03, 114.37, 117.17, 125.44, 127.45, 133.06, 134.66, 137.52, 153.51, 164.78. HRMS (*m/z*) calculated for C_22_H_25_NNaO_4_S [M + Na]^+^: 422.1402, found 422.1413.

1-(4-Ethoxyphenyl)-3-(prop-1-en-2-yl)-4-(3,4,5-trimethoxyphenyl)azetidin-2-one (**9u**)

Preparation following the general method I above from imine **8t** and dimethylacryloyl chloride afforded the product as yellow powder, yield 38%, Mp 106–108 °C (HPLC: 98%). IRν_max_ (ATR): 1729.4 (C=O, β-lactam) cm^−1^. ^1^H NMR (400 MHz, CDCl_3_): δ ppm 1.40 (t, *J* = 7.03 Hz, 3 H), 1.91 (s, 3 H), 3.73 (s, 1 H), 3.80 (s, 6 H), 3.89 (s, 3 H), 3.94–4.04 (m, 2 H) 4.73 (d, *J* = 2.51 Hz, 1 H), 5.04 (s, 1 H), 5.09 (s, 1 H), 6.55 (s, 2 H), 6.82 (d, *J* = 9.03 Hz, 2 H), 7.27 (d, *J* = 9.03 Hz, 2 H). ^13^C NMR (100 MHz, CDCl_3_): δ 14.38, 20.06, 55.76, 60.14, 60.43, 63.22, 66.60, 94.65, 102.07, 114.30, 117.93, 130.52, 133.07, 137.73, 153.46, 164.41. HRMS (*m/z*) calculated for C_23_H_27_NNaO_5_ [M + Na]^+^: 420.1787, found 420.1787.

1-(4-Methoxyphenyl)-3-(prop-1-en-2-yl)-4-(3,4,5-trimethoxyphenyl)azetidin-2-one (**9v**)

Preparation following the general method I above from imine **8u** and dimethylacryloyl chloride afforded the product as a pale yellow powder; yield 35%, Mp 128–130 °C (HPLC: 99%). IRνmax (ATR): 1727.5 (C=O, β-lactam) cm-^1^. ^1^H NMR (400 MHz, CDCl_3_): δ ppm 1.91 (s, 3 H, H-7), 3.74 (s, 1 H, H-3), 3.78 (s, 3 H, OCH_3_), 3.83 (s, 6 H, OCH_3_), 3.87 (s, 3 H, OCH_3_), 4.73 (d, *J* = 2.01 Hz, 1 H, H-4), 5.04 (s, 1 H, H-6), 5.09 (s, 1 H, H-6), 6.56 (s, 2 H, ArH), 6.83 (d, *J* = 9.03 Hz, 2 H, ArH), 7.26–7.35 (m, 2 H, ArH). ^13^C NMR (100 MHz, CDCl_3_): δ 20.06, 55.00, 55.76, 60.16, 60.43, 66.60, 102.09, 113.85, 114.30, 117.95, 130.66, 133.06, 137.46, 137.73, 153.48, 155.68, 164.43 (C2, C=O). HRMS (*m/z*) calculated for C_22_H_25_NNaO_5_ [M + Na]^+^: 406.1630, found 406.1631.

1-(4-Methoxyphenyl)-4-phenyl-3-(prop-1-en-2-yl)azetidin-2-one (**9w**)

Preparation followed the general method I above from dimethylacryloyl chloride and imine **8v**. The product was obtained as a colourless solid, yield 4.6%, 68 mg, melting point 111–113 °C (HPLC: 100.0%). IR (KBr) νmax: 1727 (C=O, β-lactam), 1624 (C=C), 1506 (C=C), 1253 (C-O) cm^−1^. ^1^H NMR (400 MHz, CDCl_3_): δ 1.90 (s, 3H, H-7), 3.72 (s, 1H, H-3), 3.76 (s, 3H, OCH_3_), 4.82 (d, *J* = 2.52 Hz, 1H, H-4), 5.03 (br s, 1H, H-6), 5.09 (br s, 1H, H-6), 6.81 (d, *J* = 9.04 Hz, 2H, H-3′, H-5′), 7.27 (d, *J* = 9.00 Hz, 2H, H-2′, H-6′), 7.36–7.39 (m, 5H, H-2c″, H-3″, H-4″, H-5″, H-6″). ^13^C NMR (100MHz, CDCl_3_): δ 20.45 (C-7), 55.35 (OCH_3_), 60.19 (C-3), 66.95 (C-4), 114.22 (C-3′, C-5′), 114.40 (C-6), 118.28 (C-2′, C-6′), 125.79, 128.41, 129.10 (C-2″, C-3″, C-4″, C-5″, C-6″), 131.00 (C-1′), 137.72 (C-1″), 138.16 (C-5), 155.96 (C-4′), 164.67 (C-2). HRMS (*m/z*) calculated for C_19_H_20_NO_2_ [M+H]^+^: 294.1494, found 294.1500.

4-(4-Methoxyphenyl)-1-phenyl-3-(prop-1-en-2-yl)azetidin-2-one (**9x**)

Preparation following the general method I above from dimethylacryloyl chloride and imine **8w** afforded the product as a colourless solid, yield 34%, 497 mg, melting point 104–105 °C (HPLC: 99.7%). IR (KBr) νmax: 2928 (C-H), 1749 (C=O, β-lactam), 1635 (C=C), 1513 (C=C), 1250 (C-O), 1087 (C-O) cm^−1^. ^1^H NMR (400MHz, CDCl_3_): δ 1.89 (s, 3H, H-7), 3.72 (d, *J* = 2.00 Hz, 1H, H-3), 3.82 (s, 3H, OCH_3_), 4.82 (d, *J* = 2.48 Hz, 1H, H-4), 5.02 (br s, 1H, H-6), 5.08 (br s, 1H, H-6), 6.92–6.94 (m, 2H, H-3″ H-5″), 7.04–7.08 (m, 1H, H-4′), 7.25–7.34 (m, 6H, H-2″, H-6″, H-2′, H-3′, H-5′, H-6′). ^13^C NMR (100 MHz, CDCl_3_): δ 20.12 (C-7), 54.87 (OCH_3_), 59.44 (C-3), 66.67 (C-4), 114.00 (C-6), 114.15 (C-’’, C-5″), 116.63 (C-2′, C6′), 123.43 (C-4′), 126.70 (C-2″, C-6″), 128.59 (C-3′, C-5′), 129.12 (C-1″), 137.10 (C-1′), 137.74 (C-5), 159.30 (C-4″), 165.03 (C-2). HRMS (*m/z*) calculated for C_19_H_19_NO_2_Na [M+Na]^+^: 316.1313, found 316.1311.

### 3.3. General Method II: Preparation of β-Lactams (***10a***-***s***)

To a stirring, refluxing solution of the appropriate imine (5 mmol) and triethylamine (6 mmol) in anhydrous dichloromethane (40 mL), a solution of 4-pentenoyl chloride (6 mmol) in anhydrous dichloromethane (10 mL) was added dropwise over 45 min under nitrogen. The reaction mixture was heated at reflux for 5 h, and then stirred at 20 °C for 20 h. The reaction mixture was washed with water (2 × 100 mL), the organic layer was dried (Na_2_SO_4_), and the solvent was removed under reduced pressure. The crude product was purified by flash chromatography over silica gel (eluent: 4:1 *n*-hexane: ethyl acetate).

3-Allyl-4-(4-nitrophenyl)-1-(3,4,5-trimethoxyphenyl)azetidin-2-one (**10a**)

Preparation followed the general method II above from 4-pentenoyl chloride and (4-nitrobenzylidene)-3,4,5-trimethoxyphenylamine (**8a**). The product was isolated as a yellow oil (yield 16%) (HPLC: 98.9%). IR (NaCl) ν_max_: 2942 (C-H), 1751 (C=O, β-lactam), 1585 (C=C), 1521 (NO_2_), 1344 (NO_2_), 1232 (C-O) cm^−1^. ^1^H NMR (400 MHz, CDCl_3_): δ 2.57–2.65 (m, 1H), 2.75–2.82 (m, 1H), 3.19–3.24 (m, 1H), 3.74 (s, 6H), 3.79 (s, 3H), 4.80 (d, *J* = 2.00 Hz, 1H), 5.20–5.26 (m, 2H) 5.83–5.93 (m, 1H), 6.49 (s, 2H), 7.54 (d, *J* = 9.04 Hz, 2H), 8.28 (d, *J* = 9.04 Hz, 2H). ^13^C NMR (100 MHz, CDCl_3_): δ 32.40, 55.67, 58.85, 59.27, 60.52, 94.07, 117.89, 124.07, 126.40, 132.85, 133.18, 134.36, 135.92, 144.84, 153.26, 165.51. HRMS (*m/z*) calculated for C_21_H_22_N_2_O_6_Na [M^+^+Na]: 421.1376, found 421.1397.

3-Allyl-4-(4-chlorophenyl)-1-(3,4,5-trimethoxyphenyl)azetidin-2-one (**10b**)

Preparation followed the general method II above from 4-pentenoyl chloride and (4-chlorobenzylidene)-(3,4,5-trimethoxyphenyl)amine (**8b**). The product was isolated as a yellow oil (yield 21%) (HPLC: 98.1%). IR (NaCl) ν_max_: 2994 (C-H), 1749 (C=O, β-lactam), 1586 (C=C), 1503 (C=C), 1233 (C-O) cm^−1^. ^1^H NMR (400 MHz, CDCl_3_): δ 2.54–2.61 (m, 1H), 2.71–2.77 (m, 1H), 3.17–3.21 (m, 1H), 3.73 (s, 6H), 3.78 (s, 3H), 4.66 (d, *J* = 2.04 Hz, 1H), 5.15–5.21 (m, 2H), 5.82–5.92 (m, 1H), 6.51 (s, 2H), 7.30 (d, *J* = 8.04 Hz, 2H), 7.38 (d, *J* = 8.52 Hz, 2H). ^13^C NMR (100 MHz, CDCl_3_): δ 32.35, 55.58, 58.82, 59.65, 60.51, 94.08, 117.46, 126.93, 128.96, 133.20, 133.42, 133.86, 134.02, 135.91, 153.10, 166.19. HRMS (*m/z*) calculated for C_21_H_22_^35^ClNO_4_Na [M^+^+Na]: 410.1135, found 410.1139.

3-Allyl-4-(4-bromophenyl)-1-(3,4,5-trimethoxyphenyl)azetidin-2-one (**10c**)

Preparation followed the general method II above from 4-pentenoyl chloride and (4-bromobenzylidene)-3,4,5-trimethoxyphenylamine (**8c**). The product was isolated as a yellow oil (yield 21%) (HPLC: 98.9%). IR (NaCl) ν_max_: 2997 (C-H), 1746 (C=O, β-lactam), 1643 (C=C), 1587 (C=C), 1503 (C=C), 1234 (C-O) cm^−1^. ^1^H NMR (400 MHz, CDCl_3_): δ 2.53–2.61 (m, 1H), 2.71–2.77 (m, 1H), 3.17–3.20 (m, 1H), 3.74 (s, 6H), 3.78 (s, 3H), 4.64 (d, *J* = 1.52 Hz, 1H), 5.15–5.21 (m, 2H), 5.82–5.92 (m, 1H), 6.51 (s, 2H), 7.25 (d, *J* = 8.56 Hz, 2H), 7.53 (d, *J* = 8.04 Hz, 2H). ^13^C NMR (100 MHz, CDCl_3_): δ 32.35, 55.59, 58.79, 59.68, 60.51, 94.07, 117.17, 121.94, 127.23, 131.91, 133.18, 133.41, 134.03, 136.45, 153.11, 166.17. HRMS (*m/z*) calculated for C_21_H_22_^80^BrNO_4_Na [M^+^+Na]: 454.0630, found 454.0645.

3-Allyl-4-(4-fluorophenyl)-1-(3,4,5-trimethoxyphenyl) azetidin-2-one (**10d**)

Preparation followed the general method II above from 4-pentenoyl chloride and (4-fluorobenzylidene)-3,4,5-trimethoxyphenylamine (**8d**). The product was isolated as colourless crystals (yield 13%); mp: 89–90 °C (HPLC: 100.0%). IR (KBr) ν_max_: 2947 (C-H), 1748 (C=O, β-lactam), 1585 (C=C), 1508 (C=C), 1228 (C-O) cm^−1^. ^1^H NMR (400 MHz, CDCl_3_): δ 2.54–2.62 (m, 1H), 2.70–2.77 (m, 1H), 3.18–3.22 (m, 1H), 3.72 (s, 6H), 3.78 (s, 3H), 4.66 (d, *J* = 2.00 Hz, 1H), 5.15–5.21 (m, 2H), 5.83–5.93 (m, 1H), 6.51 (s, 2H), 7.07–7.11 (m, 2H), 7.33–7.36 (m, 2H). ^13^C NMR (100 MHz, CDCl_3_): δ 32.34, 55.54, 58.82, 59.68, 60.51, 94.07, 115.65, 115.87, 117.38, 127.25, 127.34, 133.09, 133.26, 133.48, 133.93, 153.07, 160.20, 166.33. HRMS (*m/z*) calculated for C_21_H_22_FNO_4_Na [M^+^+Na]: 394.1431, found 394.1435.

3-Allyl-4-(4-dimethylaminophenyl)-1-(3,4,5-trimethoxyphenyl)azetidin-2-one (**10e**)

Preparation followed the general method II above from 4-pentenoyl chloride and (4-(dimethylamino)benzylidene)-3,4,5-trimethoxyphenylamine (**8e**). The product was isolated as a yellow oil (yield 11%) (HPLC: 94.7%). IR (NaCl) ν_max_: 2991 (C-H), 1741 (C=O, β-lactam), 1656 (C=C), 1587 (C=C), 1504 (C=C), 1231 (C-O) cm^−1^. ^1^H NMR (400 MHz, CDCl_3_): δ 2.52–2.60 (m, 1H), 2.67–2.74 (m, 2H), 2.97 (s, 6H), 3.21–3.25 (m, 1H), 3.73 (s, 6H), 3.77 (s, 3H), 4.58 (d, *J* = 2.52 Hz, 1H), 5.11–5.18 (m, 2H), 5.84–5.94 (m, 1H), 6.58 (s, 2H), 6.72 (d, *J* = 7.52 Hz, 2H), 7.24 (d, *J* = 8.52 Hz, 2H). ^13^C NMR (100 MHz, CDCl_3_): δ 32.35, 40.00, 55.52, 58.47, 60.39, 60.48, 94.15, 112.19, 117.01, 124.24, 126.70, 133.71, 133.75, 135.01, 150.13, 152.92, 167.06. HRMS (*m/z*) calculated for C_23_H_29_N_2_O_4_ [M^+^+H]: 397.2127, found 397.2133.

3-Allyl-4-phenyl-1-(3,4,5-trimethoxyphenyl)azetidin-2-one (**10f**)

Preparation followed the general method II above from 4-pentenoyl chloride and benzylidene-(3,4,5-trimethoxyphenyl)amine (**8f**). The product was obtained as a yellow resin (yield 35%) (HPLC: 100.0%). IR (NaCl) ν_max_: 2979 (C-H), 1748 (C=O, β-lactam), 1588 (C=C), 1505 (C=C), 1236 (C-O) cm^−1^. ^1^H NMR (400 MHz, CDCl_3_): δ 2.56–2.64 (m, 1H), 2.71–2.78 (m, 1H), 3.23–3.27 (m, 1H), 3.72 (s, 6H), 3.78 (s, 3H), 4.68 (d, *J* = 1.96 Hz, 1H), 5.15–5.22 (m, 2H), 5.86–5.95 (m, 1H), 6.54 (s, 2H), 7.35–7.38 (m, 5H). ^13^C NMR (100 MHz, CDCl_3_): δ 32.39, 55.50, 58.68, 60.39, 60.50, 94.10, 117.31, 125.63, 126.99, 128.09, 128.71, 133.46, 133.54, 136.33, 137.31, 153.02, 166.55. HRMS (*m/z*) calculated for C_21_H_23_NO_4_Na [M^+^+Na]: 376.1525, found 376.1524.

3-Allyl-4-*p*-tolyl-1-olyl-1-(3,4,5-trimethoxyphenyl)azetidin-2-one (**10g**)

Preparation followed the general method II above from 4-pentenoyl chloride and (4-methylbenzylidene)-(3,4,5-trimethoxyphenyl)amine (**8g**). The product was obtained as a yellow oil (yield 28%) (HPLC: 92.8%). IR (NaCl) ν_max_: 2979 (C-H), 1747 (C=O, β-lactam), 1588 (C=C), 1506 (C=C), 1236 (C-O) cm^−1^. ^1^H NMR (400 MHz, CDCl_3_): δ 2.37 (s, 3H), 2.54–2.61 (m, 1H), 2.69–2.76 (m, 1H), 3.20–3.24 (m, 1H), 3.72 (s, 6H), 3.77 (s, 3H), 4.64 (d, *J* = 2.48 Hz, 1H), 5.13–5.20 (m, 2H), 5.85–5.95 (m, 1H), 6.54 (s, 2H), 7.20 (d, *J* = 8.00 Hz, 2H), 7.26 (d, *J* = 8.52 Hz, 2H). ^13^C NMR (100 MHz, CDCl_3_): δ 20.75, 32.37, 55.52, 58.68, 60.24, 60.49, 94.12, 117.21, 125.57, 129.36, 133.50, 133.59, 133.77, 134.26, 137.93, 152.99, 166.67. HRMS (*m/z*) calculated for C_22_H_26_NO_4_ [M^+^+H]: 368.1862, found 368.1869.

3-Allyl-4-(4-methoxyphenyl)-1-(3,4,5-trimethoxyphenyl)azetidin-2-one (**10h**)

Preparation followed the general method II above from 4-pentenoyl chloride and (4-methoxybenzylidene)-3,4,5-trimethoxyphenylamine (**8h**). The product was obtained as a yellow solid (yield 36%); mp: 86–88 °C (HPLC: 98.95%). IR (KBr) ν_max_: 2984 (C-H), 1746 (C=O, β-lactam), 1604 (C=C), 1508 (C=C), 1250 (C-O) cm^−1^. ^1^H NMR (400 MHz, CDCl_3_): δ 2.55–2.61 (m, 1H), 2.69–2.74 (m, 1H), 3.19–3.23 (m, 1H), 3.72 (s, 6H), 3.77 (s, 3H), 3.82 (s, 3H), 4.63 (d, *J* = 2.48 Hz, 1H), 5.13–5.19 (m, 2H), 5.85–5.89 (m, 1H), 6.55 (s, 2H), 6.92 (d, *J* = 8.52 Hz, 2H), 7.30 (d, *J* = 8.52 Hz, 2H). ^13^C NMR (100 MHz, CDCl_3_): δ 32.34, 54.87, 55.53, 58.69, 60.06, 60.49, 94.12, 114.04, 117.17, 126.92, 129.18, 133.50, 133.61, 133.78, 153.00, 159.25, 166.71. HRMS (*m/z*) calculated for C_22_H_25_NO_5_Na [M^+^+Na]: 406.1630, found 406.1638.

3-Allyl-4-(4-ethoxyphenyl)-1-(3,4,5-trimethoxyphenyl)azetidin-2-one (**10i**)

Preparation followed the general method II above from 4-pentenoyl chloride and (4-ethoxybenzylidene)-(3,4,5-trimethoxyphenyl)amine (**8i**). The product was isolated as a yellow oil (yield 41%) (HPLC 94.8%). IR (NaCl) ν_max_: 2983 (C-H), 1746 (C=O, β-lactam), 1588 (C=C), 1508 (C=C), 1239 (C-O) cm^−1 1^H NMR (400 MHz, CDCl_3_): δ 1.43 (t, *J* = 7.02 Hz, 3H), 2.53–2.59 (m, 1H), 2.61–2.75 (m, 1H), 3.19–3.24 (m, 1H), 3.72 (s, 6H), 3.77 (s, 3H), 4.04 (q, *J* = 7.02 Hz, 2H), 4.62 (d, *J* = 2.52 Hz, 1H), 5.13–5.19 (m, 2H), 5.84–5.95 (m, 1H), 6.55 (s, 2H), 6.91 (d, *J* = 8.56 Hz, 2H), 7.28 (d, *J* = 8.52 Hz, 2H). ^13^C NMR (100 MHz, CDCl_3_): δ 14.34, 32.34, 55.52, 58.66, 60.09, 60.50, 63.07, 94.10, 114.55, 117.16, 126.90, 128.98, 133.52, 133.62, 133.87, 152.98, 158.63, 166.72. HRMS (*m/z*) calculated for C_23_H_27_NO_5_Na [M^+^+Na]: 420.1787, found 420.1783.

3-Allyl-4-(4-butoxyphenyl)-1-(3,4,5-trimethoxyphenyl)azetidin-2-one (**10j**)

Preparation followed the general method II above from 4-pentenoyl chloride and (4-butoxybenzylidene)-3,4,5-trimethoxyphenylamine (**8j**). The product was isolated as a yellow oil (yield 46%) (HPLC: 96.2%). IR (NaCl) ν_max_: 2940 (C-H), 1747 (C=O, β-lactam), 1602 (C=C), 1599 (C=C) 1507 (C=C), 1241 (C-O) cm^−1^. ^1^H NMR (400 MHz, CDCl_3_): δ 0.99 (t, *J* = 7.28 Hz, 3H), 1.47–1.53 (m, 2H), 1.76–1.80 (m, 2H), 2.53–2.61 (m, 1H), 2.69–2.75 (m, 1H), 3.19–3.24 (m, 1H), 3.72 (s, 6H), 3.77 (s, 3H), 3.97 (t, *J* = 6.54 Hz, 2H), 4.62 (d, *J* = 2.00 Hz, 1H), 5.13–5.19 (m, 2H), 5.83–5.93 (m, 1H), 6.55 (s, 2H), 6.91 (d, *J* = 8.56 Hz, 2H), 7.28 (d, *J* = 8.52 Hz, 2H). ^13^C NMR (100 MHz, CDCl_3_): δ 13.40, 18.78, 30.81, 32.34, 55.52, 58.67, 60.10, 60.50, 67.32, 94.11, 114.58, 117.15, 126.88, 128.91, 133.52, δ133.63, 133.75, 152.98, 158.85, 166.74. HRMS (*m/z*) calculated for C_25_H_31_NO_5_Na [M^+^+Na] 448.2100, found 448.2090.

3-Allyl-4-(4-phenoxyphenyl)-1-(3,4,5-trimethoxyphenyl)azetidin-2-one (**10k**)

Preparation followed the general method II above from 4-pentenoyl chloride and (4-phenoxylbenzylidene)-(3,4,5-trimethoxyphenyl)amine (**8k**). The product was obtained as a yellow oil (yield 31%) (HPLC: 100.0%). IR (NaCl) ν_max_: 2978 (C-H), 2941 (C-H), 1748 (C=O, β-lactam), 1587 (C=C), 1506 (C=C), 1238 (C-O) cm^−1^. ^1^H NMR (400 MHz, CDCl_3_): δ 2.55–2.63 (m, 1H, CH_2_), 2.71–2.78 (m, 1H, CH_2_), 3.22–3.26 (m, 1H), 3.74 (s, 6H), 3.79 (s, 3H), 4.66 (d, *J* = 2.52 Hz, 1H), 5.15–5.22 (m, 2H), 5.85–5.96 (m, 1H), 6.55 (s, 2H), 7.01–7.03 (m, 4H), 7.13–7.17 (m, 1H), 7.32–7.39 (m, 4H). ^13^C NMR (100 MHz, CDCl_3_): δ 32.37, 55.55, 58.72, 59.91, 60.52, 94.14, 117.31, 118.67, 118.74, 123.32, 127.11, 129.42, 131.78, 133.41, 133.55, 133.88, 153.05, 156.14, 157.19, 166.52. HRMS (*m/z*) calculated for C_27_H_27_NO_5_Na [M^+^+Na]: 468.1787, found 468.1786.

3-Allyl-4-(4-benzyloxyphenyl)-1-(3,4,5-trimethoxyphenyl)azetidin-2-one (**10l**)

Preparation followed the general method II above from 4-pentenoyl chloride and (4-benzyloxybenzylidene)-3,4,5-trimethoxyphenylamine (**8l**). The product was isolated as a grey solid (yield 34.7%); mp: 140–141 °C (HPLC: 99.8%). IR (KBr) ν_max_: 2827 (C-H), 1744 (C=O, β-lactam), 1605 (C=C), 1642 (C=C), 1508 (C=C), 1238 (C-O) cm^−1^. ^1^H NMR (400 MHz, CDCl_3_): δ 2.53–2.61 (m, 1H), 2.69–2.76 (m, 1H), 3.20–3.24 (m, 1H), 3.72 (s, 6H), 3.78 (s, 3H), 4.63 (d, *J* = 2.00 Hz, 1H), 5.08 (s, 2H), 5.13–5.20 (m, 2H), 5.84–5.94 (m, 1H), 6.54 (s, 2H), 7.00 (d, *J* = 8.52 Hz, 2H), 7.30 (d, *J* = 8.52 Hz, 2H), 7.35–7.45 (m, 5H). ^13^C NMR (100 MHz, CDCl_3_): δ 32.35, 55.53, 58.67, 60.05, 60.51, 69.60, 94.12, 115.01, 117.20, 126.95, 127.02, 127.66, 128.18, 129.48, 133.49, 133.61, 136.18, 139.82, 153.00, 158.42, 166.68. HRMS (*m/z*) calculated for C_28_H_29_NO_5_Na [M+Na]^+^: 482.1943, found 482.1941.

3-Allyl-4-naphthalen-1-yl-1-(3,4,5-trimethoxyphenyl)azetidin-2-one (**10m**)

Preparation followed the general method II above from 4-pentenoyl chloride and naphthalen-1-ylmethylene-(3,4,5-trimethoxyphenyl)amine (**8m**). The product was isolated as yellow solid (yield 41.7%); mp: 139–140 °C (HPLC: 98.7%). IR (KBr) ν_max_: 2990 (C-H), 1747 (C=O, β-lactam), 1641 (C=C), 1587 (C=C), 1505 (C=C), 1235 (C-O) cm^−1^. ^1^H NMR (400 MHz, CDCl_3_): δ 2.82–2.85 (m, 2H), 3.29–3.33 (m, 1H), 3.66 (s, 6H), 3.79 (s, 3H), 5.21–5.36 (m, 2H), 5.53 (d, *J* = 2.00 Hz, 1H), 5.91–6.02 (m, 1H), 6.59 (s, 2H), 7.44–8.31 (m, 7H). ^13^C NMR (100 MHz, CDCl_3_): δ 32.55, 55.57, 56.16, 58.71, 60.51, 94.30, 117.94, 121.76, 122.79, 125.26, 125.62, 126.21, 128.24, 128.87, 130.03, 132.57, 133.36, 133.47, 133.92, 153.09, 166.61. HRMS (*m/z*) calculated for C_25_H_25_NO_4_Na [M+Na]^+^: 426.1681, found 426.1685.

3-Allyl-4-naphthalen-2-yl-1-(3,4,5-trimethoxyphenyl)azetidin-2-one (**10n**)

Preparation followed the general method II above from 4-pentenoyl chloride and naphthalen-2-ylmethylene-(3,4,5-trimethoxyphenyl)amine (**8n**). The product was isolated as a yellow oil (yield 36%) (HPLC: 98.9%). IR (NaCl) ν_max_: 2999 (C-H), 1746 (C=O, β-lactam), 1587 (C=C), 1505 (C=C), 1236 (C-O) cm^−1^. ^1^H NMR (400 MHz, CDCl_3_): δ 2.60–2.68 (m, 1H), 2.75–2.81 (m, 1H), 3.30–3.34 (m, 1H), 3.68 (s, 6H), 3.76 (s, 3H), 4.85 (d, *J* = 2.00 Hz, 1H), 5.16–5.24 (m, 2H), 5.86–6.00 (m, 1H), 6.60 (s, 2H), 7.46–7.90 (m, 7H). ^13^C NMR (100 MHz, CDCl_3_): δ 32.41, 55.54, 58.76, 60.49, 60.52, 94.13, 117.38, 122.76, 125.00, 126.0, 126.24, 127.38, 127.40, 128.88, 132.86, 133.16, 133.54, 134.84, 135.03, 153.06, 166.58. HRMS (*m/z*) calculated for C_25_H_25_NO_4_Na [M+Na]^+^: 426.1681, found 426.1689.

4-(3-Hydroxy-4-methoxyphenyl)-1-(3,4,5-trimethoxyphenyl)-3-allylazetidin-2-one (**10p**)

Preparation followed the general method II above from 4-pentenoyl chloride and TBDMS-protected imine **8p**. The intermediate 4-(3-((*tert*-butyldimethylsilyl)oxy)-4-methoxyphenyl)-1-(3,4,5-trimethoxyphenyl)-3-allylazetidin-2-one **10o** was obtained as described above for compound **9q.** Following deprotection with TBAF, the title compound was purified by flash chromatography over silica gel (eluent, 4:1 *n*-hexane: ethyl acetate) to afford the product as a grey-green solid (yield 22%); mp: 96–98 °C (HPLC: 98.08%). IR (KBr) ν_max_: 3400 (OH), 1746 (C=O, β-lactam), 1592 (C=C), 1506 (C=C), 1275 (C-O) cm^−1^. ^1^H NMR (400 MHz, CDCl_3_): δ 2.51–2.59 (m, 1H), 2.68–2.74 (m, 1H), 3.18–3.22 (m, 1H), 3.73 (s, 6H), 3.77 (s, 3H), 3.91 (s, 3H), 4.57 (d, *J* = 2.52 Hz, 1H), 5.12–5.19 (m, 2H), 5.71 (br s, 1H), 5.82–5.92 (m, 1H), 6.56 (s, 2H), 6.85–6.94 (m, 3H). ^13^C NMR (100 MHz, CDCl_3_): δ 32.34, 55.53, 55.55, 58.64, 60.02, 60.49, 94.13, 110.46, 111.69, 117.20, 117.36, 130.42, 133.49, 133.59, 133.77, 145.71, 146.18, 152.98, 166.64. HRMS (*m/z*) calculated for C_22_H_25_NO_6_Na [M+Na]^+^: 422.1580, found 422.1582.

3-Allyl-4-(4-methoxy-3-nitrophenyl)-1-(3,4,5-trimethoxyphenyl)azetidin-2-one (**10q**)

Preparation following the general method II above from 4-pentenoyl chloride and (4-methoxy-3-nitrobenzylidene)-(3,4,5-trimethoxyphenyl)amine (**8r**) afforded the product as a colourless solid (yield 18%); mp: 108–110 °C (HPLC: 99.14%). IR (KBr) ν_max_: 1750 (C=O, β-lactam), 1587 (C=C), 1504 (NO_2_), 1280 (NO_2_) cm^−1^. ^1^H NMR (400 MHz, CDCl_3_): δ 2.55–2.63 (m, 1H), 2.72–2.78 (m, 1H), 3.19–3.24 (m, 1H), 3.76 (s, 6H), 3.79 (s, 3H), 3.99 (s, 3H), 4.68 (d, *J* = 2.00 Hz, 1H), 5.18–5.23 (m, 2H), 5.82–5.92 (m, 1H), 6.51 (s, 2H), 7.13 (d, *J* = 8.52 Hz, 1H), 7.52–7.55 (m, 1H), 7.88 (d, *J* = 2.52 Hz, 1H). ^13^C NMR (100 MHz, CDCl_3_): δ 32.27, 55.68, 56.25, 58.78, 58.85, 60.52, 94.10, 114.02, 117.77, 123.27, 129.77, 130.85, 132.92, 133.20, 134.24, 139.30, 152.49, 153.21, 165.89. HRMS (*m/z*) calculated for C_22_H_24_N_2_O_7_Na [M+Na]^+^: 451.1481, found 451.1477.

3-Allyl-4-(3-amino-4-methoxyphenyl)-1-(3,4,5-trimethoxyphenyl)azetidin-2-one (**10r**)

To a mixture of the β-lactam 3-allyl-4-(4-methoxy-3-nitrophenyl)-1-(3,4,5-trimethoxyphenyl)azetidin-2-one **10q** (0.25 mmol) and zinc powder (10 μm, 2.5 mmol) was added acetic acid (15 mL) at room temperature under N_2_, and the reaction was stirred for 7 days. The reaction mixture was filtered through a celite pad, and the solvent was removed under reduced pressure. The residue was purified by flash chromatography over silica gel (eluent, 1:1 ethyl acetate: *n*-hexane) to afford the title compound. The product was isolated as a brown oil (yield 96%) (HPLC: 97.93%). IR (NaCl) ν_max_: 3376 (NH_2_), 1727 (C=O, β-lactam), 1592 (C=C), 1507 (C=C), 1292 (C-O) cm^−1^. ^1^H NMR (400 MHz, CDCl_3_): δ 2.52–2.57 (m, 1H), 2.67–2.74 (m, 1H), 3.19–3.23 (m, 1H), 3.74 (s, 6H), 3.77 (s, 3H), 3.89 (s, 3H), 4.58 (d, *J* = 2.00 Hz, 1H), 5.13–5.20 (m, 2H), 5.83–5.93 (m, 1H), 6.56 (s, 2H), 6.85–7.02 (m, 3H). ^13^C NMR (100 MHz, CDCl_3_): δ 32.32, 55.28, 55.60, 58.56, 60.05, 60.50, 94.13, 110.30, 113.61, 117.25, 123.85, 129.57, 133.52, 133.58, 133.77, 133.97, 147.80, 152.99, 166.79. HRMS (*m/z*) calculated for C_22_H_27_N_2_O_5_ [M+H]^+^: 399.1920, found 399.1907.

3-Allyl-4-(4-Methoxyphenyl)-1-phenylazetidin-2-one (**10s**)

Preparation following the general method II above from 4-pentenoyl chloride and the imine **8v** (4-methoxybenzylidene)(phenyl)amine afforded the product as a yellow oil, yield 40%, 590 mg (HPLC 100.0%). IR (NaCl, film) ν_max_: 2979 (C-H), 1749 (C=O, β-lactam), 1640 (C=C), 1602 (C=C), 1588 (C=C), 1513 (C=C), 1251 (C-O) cm^−1^. ^1^H NMR (400 MHz, CDCl_3_): δ 2.53–2.61 (m, 1H), 2.70–2.76 (m, 1H), 3.17–3.22 (m, 1H), 3.82 (s, 3H), 4.67 (d, *J* = 2.04 Hz, 1H), 5.13–5.20 (m, 2H), 5.84–5.94 (m, 1H), 6.91 (d, *J* = 8.52 Hz, 2H), 7.02–7.07 (m, 1H), 7.23–7.31 (m, 6H). ^13^C NMR (100 MHz, CDCl_3_): δ 32.83, 55.32, 59.35, 60.13, 114.51, 117.02, 117.61, 123.76, 127.29, 129.03, 129.72, 134.16, 137.73, 159.63, 167.32. HRMS (*m/z*) calculated for C_19_H_19_NO_2_Na [M+Na]^+^: 316.1313, found 316.1320.

### 3.4. General Method III: Preparation of β-Lactams (***11a***-***11s***)

Sorbic acid (2 mmol) was mixed with 2-chloro-1-methylpyridinium iodide (2.4 mmol) and tripropylamine (6 mmol) in anhydrous dichloromethane (30 mL) under a nitrogen atmosphere at room temperature. The suspension was then heated to reflux for 12 h to afford a clear solution. A solution of the appropriate imine (2 mmol) in anhydrous dichloromethane (10 mL) was added, and reaction mixture was heated at reflux for 24 h. The solution was then cooled and washed with water, HCl (2%, aqueous solution) and water. The organic layer was dried over anhydrous Na_2_SO_4_, and the solvent was removed under reduced pressure. The crude product was purified by column chromatography over silica gel (eluent, 4:1 *n*-hexane and ethyl acetate).

(*E*)-3-(Buta-1,3-dien-1-yl)-4-(4-nitrophenyl)-1-(3,4,5-trimethoxyphenyl)azetidin-2-one (**11a**)

Preparation following the general method III above from sorbic acid and (4-nitrobenzylidene)-3,4,5-trimethoxyphenylamine **8a** afforded the product as a yellow solid (yield 33%); mp: 133–134 °C (HPLC: 85.8%). IR (KBr) ν_max_: 2972 (C-H), 1749 (C=O, β-lactam), 1653 (C=C), 1568 (C=C), 1506 (NO_2_), 1345 (NO_2_), 1238 (C-O) cm^−1^. ^1^H NMR (400 MHz, CDCl_3_): δ 3.74 (s, 6H), 3.77 (br s, 1H), 3.79 (s, 3H), 4.91 (d, *J* = 2.00 Hz, 1H), 5.21–5.23 (m, 1H), 5.28–5.32 (m, 1H), 5.89 (dd, *J*_5,6_ = 13.54 Hz, *J*_5,3_ = 8.54 Hz, 1H), 6.31–6.44 (m, 2H), 6.50 (s, 2H), 7.57 (d, *J* = 8.52 Hz, 2H), 8.29 (d, *J* = 8.04 Hz, 2H). ^13^C NMR (100 MHz, CDCl_3_): δ 55.69, 60.53, 60.60, 62.91, 94.20, 118.98, 123.98, 124.17, 126.24, 132.72, 134.52, 135.10, 135.90, 144.23, 147.65, 153.29, 163.91. HRMS (*m/z*) calculated for C_22_H_22_N_2_O_6_Na [M+Na]^+^: 433.1376, found 433.1392.

(*E*)-3-(Buta-1,3-dien-1-yl)-4-(4-chlorophenyl)-1-(3,4,5-trimethoxyphenyl)azetidin-2-one (**11b**)

Preparation following the general method III above from sorbic acid and (4-chlorobenzylidene)-(3,4,5-trimethoxyphenyl)amine **8b** afforded the product as a colourless solid (yield 32%); mp: 145–147 °C (HPLC: 95.4%). IR (KBr) ν_max_: 2971 (C-H), 1748 (C=O, β-lactam), 1655 (C=C), 1584 (C=C), 1506 (C=C), 1238 (C-O) cm^−1^. ^1^H NMR (400 MHz, CDCl_3_): δ 3.74 (s, 6H), 3.77–3.78 (m, 1H), 3.79 (s, 3H), 4.76 (d, *J* = 2.52 Hz, 1H), 5.18–5.20 (m, 1H), 5.26–5.30 (m, 1H), 5.88 (dd, *J*_5,6_ = 14.16 Hz, *J*_5,3_ = 8.16 Hz, 1H), 6.31–6.43 (m, 2H), 6.53 (s, 2H), 7.32–7.34 (m, 2H), 7.39–7.41 (m, 2H). ^13^C NMR (100 MHz, CDCl_3_): δ 55.62, 60.52, 60.95, 62.79, 94.21, 118.44, 126.79, 128.67, 129.06, 133.06, 134.12, 134.17, 135.31, 135.34, 135.36, 153.12, 164.51. HRMS (*m/z*) calculated for C_22_H_22_^35^ClNO_4_Na [M+Na]^+^: found 422.1130.

(*E*)-4-(4-Bromophenyl)-3-(buta-1,3-dien-1-yl)-1-(3,4,5-trimethoxyphenyl)azetidin-2-one (**11c**)

Preparation following the general method III above from sorbic acid and (4-bromobenzylidene)-3,4,5-trimethoxyphenylamine **8c** afforded the product as a colourless solid (yield 44%); mp: 153–155 °C (HPLC: 93.5%). IR (KBr) ν_max_: 2969, 1748 (C=O, β-lactam), 1657 (C=C), 1583 (C=C), 1505 (C=C), 1239 (C-O) cm^−1^. ^1^H NMR (400 MHz, CDCl_3_): δ 3.74 (s, 6H), 3.76–3.77 (m, 1H), 3.79 (s, 3H), 4.74 (d, *J* = 2.00 Hz, 1H), 5.17–5.20 (m, 1H), 5.25–5.29 (m, 1H), 5.87 (dd, *J*_5,6_ = 14.06 Hz, *J*_5,3_ = 8.02 Hz, 1H), 6.31–6.40 (m, 2H), 6.52 (s, 2H), 7.27 (d, *J* = 8.00 Hz, 2H), 7.55 (d, *J* = 8.52 Hz, 2H). ^13^C NMR (100 MHz, CDCl_3_): δ 55.63, 60.52, 61.00, 62.75, 94.22, 118.46, 122.20, 124.61, 127.08, 132.01, 133.03, 134.20, 135.30, 135.35, 135.89, 153.13, 164.49. HRMS (*m/z*) calculated for C_22_H_22_^80^BrNO_4_N [M+Na]^+^: 466.0630, found 466.0622.

(*E*)-3-(Buta-1,3-dien-1-yl)-4-(4-fluorophenyl)-1-(3,4,5-trimethoxyphenyl)azetidin-2-one (**11d**)

Preparation following the general method III above from sorbic acid and (4-fluorobenzylidene)-3,4,5-trimethoxyphenylamine (**8d**) afforded the product as a colourless solid (yield 20%); mp: 137–139 °C (HPLC: 100.0%). IR (KBr). ν_max_: 2968 (C-H), 1748 (C=O, β-lactam), 1658 (C=C), 1583 (C=C), 1505 (C=C), 1227 (C-O) cm^−1^. ^1^H NMR (400 MHz, CDCl_3_): δ 3.73 (s, 6H), 3.76–3.77 (m, 1H), 3.79 (s, 3H), 4.77 (d, *J* = 2.00 Hz, 1H), 5.17–5.19 (m, 1H), 5.25–5.29 (m, 1H), 5.85–5.91 (m, 1H), 6.34–6.40 (m, 2H), 6.53 (s, 2H), 7.09–7.14 (m, 2H), 7.36–7.39 (m, 2H). ^13^C NMR (100MHz, CDCl_3_): δ 55.59, 60.52, 61.01, 62.82, 94.22, 115.77, 115.98, 118.36, 124.74, 127.17, 127.24, 132.60, 133.13, 133.94, 135.24, 135.35, 153.10, 163.57, 164.63. HRMS (*m/z*) calculated for C_22_H_22_FNO_4_Na [M+Na]^+^: 406.1431, found 406.1421.

(*E*)-3-(Buta-1,3-dien-1-yl)-4-(4-dimethylaminophenyl)-1-(3,4,5-trimethoxyphenyl)azetidin-2-one (**11e**)

Preparation following the general method III above from sorbic acid and (4-(dimethylamino)benzylidene)-3,4,5-trimethoxyphenylamine **8e** afforded the product as a brown oil (yield 16%) (HPLC: 91.1%). IR (NaCl) ν_max_: 2968 (C-H), 1743 (C=O, β-lactam), 1656 (C=C), 1584 (C=C), 1505 (C=C), 1236 (C=O) cm^−1^. ^1^H NMR (400 MHz, CDCl_3_): δ 2.98 (s, 6H), 3.73 (s, 6H), 3.77 (s, 3H), 3.80–3.82 (m, 1H), 4.68 (d, *J* = 2.52 Hz, 1H), 5.13–5.16 (m, 1H), 5.22–5.26 (m, 1H,), 5.88 (dd, *J*_5,6_ = 14.06 Hz, *J*_5,3_ = 8.02 Hz, 1H), 6.29–6.42 (m, 2H), 6.60 (s, 2H), 6.74 (d, *J* = 8.04 Hz, 2H), 7.25–7.27 (m, 2H). ^13^C NMR (100 MHz, CDCl_3_): δ 40.02, 55.55, 60.49, 61.74, 62.57, 94.29, 112.22, 117.81, 125.51, 126.67, 133.55, 133.79, 133.89, 134.72, 135.58, 145.67, 152.95, 165.30. HRMS (*m/z*) calculated for C_24_H_29_N_2_O_4_ [M+H]^+^: 409.2127, found 409.2141.

(*E*)-3-(Buta-1,3-dien-1-yl)-4-phenyl-1-(3,4,5-trimethoxyphenyl)azetidin-2-one (**11f**)

Preparation following the general method III above from sorbic acid and benzylidene-(3,4,5-trimethoxyphenyl)amine **8f** afforded the product as a yellow solid (yield 37%); mp: 90–93 °C (HPLC: 94.1%). IR (NaCl) ν_max_: 2969 (C-H), 1747 (C=O, β-lactam), 1660 (C=C), 1585 (C=C), 1505 (C=C), 1239 (C-O) cm^−1^. ^1^H NMR (400 MHz, CDCl_3_): δ 3.71 (s, 6H), 3.78 (s, 3H), 3.80–3.83 (m, 1H), 4.77 (d, *J* = 2.52 Hz, 1H), 5.16–5.18 (m, 1H), 5.24–5.28 (m, 1H), 5.89 (dd, *J*_5,6_ = 14.06 Hz, *J*_5,3_ = 8.02 Hz, 1H), 6.32–6.43 (m, 2H), 6.55 (s, 2H), 7.35–7.39 (m, 5H). ^13^C NMR (100 MHz, CDCl_3_): δ 55.54, 60.51, 61.71, 62.65, 94.23, 118.18, 125.02, 125.52, 128.33, 128.81, 133.31, 133.98, 135.10, 135.44, 136.77, 153.04, 164.82. HRMS (*m/z*) calculated for C_22_H_24_NO_4_ [M+H]^+^: 366.1705, found 366.1695.

3-(Buta-1,3-dienyl)-4-*p*-tolyl-1-(3,4,5-trimethoxyphenyl)azetidin-2-one (**11g**)

Preparation following the general method III above from sorbic acid and (4-methylbenzylidene)-(3,4,5-trimethoxyphenyl)amine **8g** afforded the product as a yellow solid (yield 40%); mp: 88–89 °C (HPLC: 97.0%). IR (KBr) ν_max_: 2960 (C-H), 1748 (C=O, β-lactam), 1659 (C=C), 1640 (C=C), 1588 (C=C), 1505 (C=C), 1238 (C-O) cm^−1^. ^1^H NMR (400 MHz, CDCl_3_): δ 2.37 (s, 3H), 3.72 (s, 6H), 3.78 (s, 3H), 3.80–3.81 (m, 1H), 4.74 (d, *J* = 2.48 Hz, 1H), 5.15–5.17 (m, 1H), 5.23–5.27 (m, 1H), 5.88 (dd, *J*_5,6_ = 14.28 Hz, *J*_5,3_ = 8.28 Hz, 1H), 6.30–6.40 (m, 2H), 6.56 (s, 2H), 7.22 (d, *J* = 8.04 Hz, 2H), 7.28 (d, *J* = 8.04 Hz, 2H). ^13^C NMR (100 MHz, CDCl_3_): δ 20.77, 55.55, 60.50, 61.56, 62.68, 94.24, 118.08, 125.15, 125.47, 129.45, 133.37, 133.72, 133.94, 134.98, 135.48, 138.21, 153.02, 164.93. HRMS (*m/z*) calculated for C_23_H_26_NO_4_ [M+H]^+^: 380.1862, found 380.1862.

3-(Buta-1,3-dienyl)-4-(4-methoxyphenyl)-1-(3,4,5-trimethoxyphenyl)azetidin-2-one (**11h**)

Preparation following the general method III above from sorbic acid and (4-methoxybenzylidene)-3,4,5-trimethoxyphenylamine **8h** afforded the product as a colourless solid (yield 31%); mp: 109–110 °C (HPLC: 99.7%). IR (KBr) ν_max_: 2969 (C-H), 1745 (C=O, β-lactam), 1656 (C=C), 1581 (C=C), 1508 (C=C), 1247 (C-O) cm^−1^. ^1^H NMR (400 MHz, CDCl_3_): δ 3.73 (s, 6H), 3.78 (s, 3H), 3.80 (m, 1H), 3.83 (s, 3H), 4.73 (d, *J* = 2.00 Hz, 1H), 5.15–5.18 (m, 1H), 5.24–5.28 (m, 1H), 5.88 (dd, *J*_5,6_ = 14.28 Hz, *J*_5,3_ = 8.28 Hz, 1H), 6.31–6.43 (m, 2H), 6.56 (s, 2H), 6.94 (d, *J* = 9.00 Hz, 2H), 7.32 (d, *J* = 9.04 Hz, 2H). ^13^C NMR (100 MHz, CDCl_3_): δ 54.90, 55.56, 60.51, 61.38, 62.74, 94.25, 114.15, 118.07, 125.15, 126.84, 128.62, 133.36, 133.94, 134.96, 135.48, 153.02, 159.44, 164.98. HRMS (*m/z*) calculated for C_23_H_26_NO_5_ [M+H]^+^: 396.1811, found 396.1800.

(*E*)-3-(Buta-1,3-dien-1-yl)-4-(4-ethoxyphenyl)-1-(3,4,5-trimethoxyphenyl)-azetidin-2-one (**11i**)

Preparation following the general method III above from sorbic acid and (4-ethoxybenzylidene)-(3,4,5-trimethoxyphenyl)amine **8i** afforded the product as a yellow solid (yield 41%); mp: 73–75 °C (HPLC: 99.8%). IR (KBr) ν_max_: 2969 (C-H), 1747 (C=O, β-lactam), 1658 (C=C), 1584 (C=C), 1511 (C=C), 1244 (C-O) cm^−1^. ^1^H NMR (400 MHz, CDCl_3_): δ 1.43 (t, *J* = 7.02 Hz, 3H), 3.73 (s, 6H), 3.78 (s, 3H), 3.79–3.80 (m, 1H), 4.05 (q, *J* = 7.02 Hz, 2H), 4.72 (d, *J* = 2.52 Hz, 1H), 5.15–5.17 (m, 1H), 5.24–5.28 (m, 1H), 5.88 (dd, *J*_5,6_ = 14.04 Hz, *J*_5,3_ = 8.52 Hz, 1H), 6.31–6.42 (m, 2H), 6.56 (s, 2H), 6.91–6.93 (m, 2H), 7.29–7.32 (m, 2H). ^13^C NMR (100 MHz, CDCl_3_): δ 14.33, 55.55, 60.51, 61.42, 62.72, 63.11, 94.24, 114.66, 118.04, 125.19, 126.82, 128.42, 133.39, 133.92, 134.93, 135.48, 153.01, 158.83, 165.00. HRMS (*m/z*) calculated for C_24_H_27_NO_5_Na [M+Na]^+^: 432.1787, found 432.1792.

(*E*)-3-(Buta-1,3-dien-1-yl)-4-(4-butoxyphenyl)-1-(3,4,5-trimethoxyphenyl)-azetidin-2-one (**11j**)

Preparation following the general method III above from sorbic acid and (4-butoxybenzylidene)-3,4,5-trimethoxyphenylamine **8j** afforded the product as a yellow oil (yield 48%) (HPLC: 94.0%). IR (NaCl) ν_max_: 2968 (C-H), 1747 (C=O, β-lactam), 1657 (C=C), 1583 (C=C), 1508 (C=C), 1245 (C-O) cm^−1^. ^1^H NMR (400 MHz, CDCl_3_): δ 0.99 (t, *J* = 7.54 Hz, 3H), 1.48–1.53 (m, 2H), 1.74–1.81 (m, 2H), 3.73 (s, 6H), 3.78 (s, 3H), 3.79–3.80 (m, 1H), 3.97 (t, *J* = 6.28Hz, 2H), 4.72 (d, *J* = 2.52 Hz, 1H), 5.14–5.17 (m, 1H), 5.23–5.27 (m, 1H), 5.88 (dd, *J*_5,6_ = 14.30 Hz, *J*_5,3_ = 8.26 Hz, 1H), 6.30–6.42 (m, 2H), 6.56 (s, 2H), 6.92 (d, *J* = 8.52 Hz, 2H), 7.30 (d, *J* = 8.52 Hz, 2H). ^13^C NMR (100 MHz, CDCl_3_): δ 13.39, 18.77, 30.79, 55.55, 60.50, 61.42, 62.73, 67.34, 94.26, 114.68, 118.03, 125.20, 126.80, 128.35, 133.39, 133.93, 134.92, 135.48, 153.01, 159.04, 165.00. HRMS (*m/z*) calculated for C_26_H_31_NO_5_Na [M+Na]^+^: 460.2100, found 460.2103.

(*E*)-3-(Buta-1,3-dien-1-yl)-4-(4-phenoxyphenyl)-1-(3,4,5-trimethoxyphenyl)azetidin-2-one (**11k**)

Preparation following the general method III above from sorbic acid and (4-phenoxylbenzylidene)-(3,4,5-trimethoxyphenyl)amine **8k** afforded the product as a colourless solid (yield 33%); mp: 119–120 °C (HPLC: 90.3%). IR (KBr) ν_max_: 2973 (C-H), 1748 (C=O, β-lactam), 1654 (C=C), 1587 (C=C), 1507 (C=C), 1239 (C-O) cm^−1^. ^1^H NMR (400 MHz, CDCl_3_): δ 3.75 (s, 6H), 3.79 (s, 3H), 3.82–3.83 (m, 1H), 4.76 (d, *J* = 2.48 Hz, 1H), 5.16–5.19 (m, 1H), 5.25–5.29 (m, 1H), 5.89 (dd, *J*_5,6_ = 14.06 Hz, *J*_5,3_ = 8.02 Hz, 1H), 6.33–6.43 (m, 2H), 6.56 (s, 2H), 7.02–7.05 (m, 4H), 7.14–7.17 (m, 1H), 7.34–7.39 (m, 4H). ^13^C NMR (100 MHz, CDCl_3_): δ 55.59, 60.52, 61.27, 62.73, 94.31, 118.19, 118.74, 118.76, 123.37, 124.98 (CH), 127.03, 129.44, 131.22, 133.26, 133.95, 135.10, 135.43, 153.08, 156.11, 157.42, 164.79. HRMS (*m/z*) calculated for C_28_H_28_NO_5_ [M+H]: 458.1967, found 458.1974.

(*E*)-3-(Buta-1,3-dien-1-yl)-4-(4-benzyloxyphenyl)-1-(3,4,5-trimethoxyphenyl)azetidin-2-one (**11l**)

Preparation following the general method III above from sorbic acid and (4-benzyloxybenzylidene)-3,4,5-trimethoxyphenylamine **8l** afforded the product as a yellow oil (yield 41%) (HPLC: 94.1%). IR (NaCl) ν_max_: 2969 (C-H), 1746 (C=O, β-lactam), 1658 (C=C), 1584 (C=C), 1508 (C=C), 1240 (C-O) cm^−1^. ^1^H NMR (400 MHz, CDCl_3_): δ 3.72 (s, 6H), 3.78 (s, 3H), 3.79–3.80 (m, 1H), 4.72 (d, *J* = 2.52 Hz, 1H), 5.09 (s, 2H), 5.15–5.18 (m, 1H), 5.24–5.28 (m, 1H), 5.88 (dd, *J*_5,6_ = 14.06 Hz, *J*_5,3_ = 8.02 Hz, 1H), 6.31–6.42 (m, 2H), 6.56 (s, 2H), 7.01 (d, *J* = 8.04 Hz, 2H), 7.31–7.45 (m, 7H). ^13^C NMR (100 MHz, CDCl_3_): δ 55.56, 60.51, 61.38, 62.7, 69.61, 94.25, 115.10, 118.08, 125.14, 126.87, 127.01, 127.67, 128.19, 128.90, 133.35, 133.95, 134.98, 135.47, 136.13, 153.02, 158.59, 164.96. HRMS (*m/z*) calculated for C_29_H_29_NO_5_Na [M+Na]^+^: 494.1943, found 494.1931.

(*E*)-3-(Buta-1,3-dien-1-yl)-4-(naphthalen-1-yl)-1-(3,4,5-trimethoxyphenyl)azetidin-2-one (**11m**)

Preparation following the general method III above from sorbic acid and naphthalen-1-ylmethylene-(3,4,5-trimethoxyphenyl)amine **8m** afforded the product as a grey solid (yield 27%); mp: 113–114 °C (HPLC: 95.4%). IR (KBr) ν_max_: 2968 (C=C), 1748 (C=O, β-lactam), 1657 (C=C), 1584 (C=C), 1505 (C=C), 1237 (C-O) cm^−1^. ^1^H NMR (400 MHz, CDCl_3_): δ 3.71 (s, 6H), 3.78–3.81 (m, 1H), 3.82 (s, 3H), 5.20–5.23 (m, 1H), 5.26–5.30 (m, 1H), 5.56 (d, *J* = 2.00 Hz, 1H), 6.09 (dd, *J*_5,6_ = 14.80 Hz, *J*_5,3_ = 8.80 Hz, 1H), 6.36–6.52 (m, 2H), 6.66 (s, 2H), 7.43–8.01 (m, 7H). ^13^C NMR (100 MHz, CDCl_3_): δ 55.71, 58.95, 60.53, 62.35, 94.56, 118.40, 122.22, 122.39, 125.10, 125.40, 125.44, 125.71, 126.30, 128.21, 128.74, 129.95, 132.14, 133.45, 133.57, 135.48, 136.04, 153.19, 164.88. HRMS (*m/z*) calculated for C_26_H_25_NO_4_Na [M+Na]^+^: 438.1681, found 438.1682.

(*E*)-3-(Buta-1,3-dien-1-yl)-4-(naphthalen-2-yl)-1-(3,4,5-trimethoxyphenyl)azetidin-2-one (**11n**)

Preparation following the general method III above from sorbic acid and naphthalen-2-ylmethylene-(3,4,5-trimethoxyphenyl)amine **8n** afforded the product as a yellow oil (yield 53%) (HPLC: 86.4%). IR (NaCl) ν_max_: 2968 (C-H), 1747 (C=O, β-lactam), 1659 (C=C), 1585 (C=C), 1506 (C=C), 1238 (C-O) cm^−1^. ^1^H NMR (400MHz, CDCl_3_): δ 3.69 (s, 6H), 3.77 (s, 3H), 3.87–3.90 (m, 1H), 4.95 (d, *J* = 2.96 Hz, 1H), 5.16–5.19 (m, 1H), 5.25–5.29 (m, 1H), 5.94 (dd, *J*_5,6_ = 14.16 Hz, *J*_5,3_ = 8.32 Hz, 1H), 6.35–6.42 (m, 2H), 6.62 (s, 2H), 7.47–7.92 (m, 7H). ^13^C NMR (100 MHz, CDCl_3_): δ 55.58, 60.48, 61.89, 62.75, 94.33, 118.21, 122.45, 124.98, 126.12, 126.33, 127.39, 127.42, 129.01, 132.87, 132.95, 133.42, 134.13, 134.28, 135.19, 135.43, 153.09, 164.84. HRMS (*m/z*) calculated for C_26_H_26_NO_4_ [M+H]^+^: 416.1862, found 416.1863.

(*E*)-3-(Buta-1,3-dien-1-yl)-4-(3-hydroxy-4-methoxyphenyl)-1-(3,4,5-trimethoxyphenyl)azetidin-2-one (**11p**)

Preparation followed the general method III above from sorbic acid and imine **8p** as described above for compound **9q**. The intermediate 4-(3-((*tert*-butyldimethylsilyl)oxy)-4-methoxyphenyl)-1-(3,4,5-trimethoxyphenyl)-3-(buta-1,3-dienyl) azetidin-2-one **11o** was isolated as described for compound **9q**. Following deprotection with TBAF, the solvent was removed and the crude product was purified by flash chromatography over silica gel (eluent, 4:1 *n*-hexane: ethyl acetate) to afford the title compound as a grey-brown oil (yield 37%) (HPLC: 97.8%). IR (NaCl) ν_max_: 3359 (OH), 1748 (C=O, β-lactam), 1594 (C=C), 1509 (C=C), 1280 (C-O) cm^−1^. ^1^H NMR (400 MHz, CDCl_3_): δ 3.72 (s, 6H), 3.75–3.76 (m, 1H), 3.77 (s, 3H), 3.82 (s, 3H), 4.67 (d, *J* = 2.36 Hz, 1H), 5.15–5.17 (m, 1H), 5.24–5.28 (m, 1H), 5.85–5.90 (m, 1H), 6.31–6.40 (m, 2H), 6.56 (s, 2H), 6.82–6.96 (m, 3H). ^13^C NMR (100 MHz, CDCl_3_): δ 55.06, 55.51, 60.49, 61.34, 62.60, 94.30, 111.89, 117.96, 118.03, 119.06, 125.20, 129.04, 133.30, 133.91, 134.91, 135.50, 145.23, 150.86, 152.99, 164.95. HRMS (*m/z*) calculated for C_23_H_24_NO_6_ [M-H]^-^: 410.1604, found 410.1605.

(*E*)-3-(Buta-1,3-dien-1-yl)-4-(4-methoxy-3-nitrophenyl)-1-(3,4,5-trimethoxyphenyl)azetidin-2-one (**11q**)

Preparation following the general method III above from sorbic acid and (4-methoxy-3-nitrobenzylidene)-(3,4,5-trimethoxyphenyl)amine **8r** afforded the product as a yellow solid (yield 25%); mp: 88–90 °C (HPLC: 95.7%). IR (KBr) ν_max_: 2969 (C-H), 1748 (C=O, β-lactam), 1656 (C=C), 1584 (C=C), 1506 (C=C), 1280 (C-O) cm^−1 1^H NMR (400 MHz, CDCl_3_): δ 3.76 (s, 6H), 3.79 (s, 3H), 3.84–3.87 (m, 1H), 3.99 (s, 3H), 4.79 (d, *J* = 2.52 Hz, 1H), 5.18–5.21 (m, 1H), 5.27–5.30 (m, 1H), 5.86 (dd, *J*_5,6_ = 14.32 Hz, *J*_5,3_ = 8.28 Hz, 1H), 6.34–6.40 (m, 2H), 6.52 (s, 2H), 7.15 (d, *J* = 8.52 Hz, 1H), 7.55–7.57 (m, 1H), 7.90 (d, *J* = 2.00 Hz, 1H). ^13^C NMR (100 MHz, CDCl_3_): δ 55.70, 56.28, 60.18, 60.52, 62.77, 94.27, 114.13, 118.72, 123.16, 124.19, 129.19, 130.72, 132.77, 133.04, 135.18, 135.60, 139.29, 152.65, 153.23, 164.27. HRMS (*m/z*) calculated for C_23_H_24_N_2_O_7_Na [M+Na]^+^: 463.1481, found 463.1465.

(*E*)-4-(3-Amino-4-methoxyphenyl)-3-(buta-1,3-dien-1-yl)-1-(3,4,5-trimethoxyphenyl)azetidin-2-one (**11r**)

Preparation from *(E)*-3-(buta-1,3-dienyl)-4-(4-methoxy-3-nitrophenyl)-1-(3,4,5-trimethoxyphenyl) azetidin-2-one **11q** followed the general method III described above for **9s**. The product was isolated as a yellow solid (yield 59%); mp: 136–138 °C (HPLC: 98.0%). IR (KBr) ν_max_: 3376 (NH_2_), 1741 (C=O, β-lactam), 1593 (C=C), 1504 (C=C), 1292 (C-O), 1234 cm^−1^. ^1^H NMR (400 MHz, CDCl_3_): δ 3.75 (s, 6H), 3.77 (d, *J* = 2.48 Hz, 1H), 3.78 (s, 3H), 3.89 (s, 3H), 4.66 (d, *J* = 2.52 Hz, 1H), 5.14–5.17 (m, 1H), 5.23–5.28 (m, 1H), 5.84–5.89 (m, 1H), 6.30–6.42 (m, 2H), 6.59–6.62 (m, 2H), 6.82–6.91 (m, 3H). ^13^C NMR (100 MHz, CDCl_3_): δ 55.76, 56.10, 60.94, 61.92, 63.11, 94.82, 110.90, 114.00, 118.45, 125.65, 126.64, 129.84, 133.88, 134.50, 134.89, 135.39, 135.96, 144.75, 153.51, 165.46. HRMS (*m/z*) calculated for C_23_H_26_N_2_O_5_Na [M+Na]^+^: 433.1739, found 433.1729.

(*E*)-3-(Buta-1,3-dien-1-yl)-4-(4-methoxyphenyl)-1-phenylazetidin-2-one (**11s**)

Preparation following the general method III above from sorbic acid and imine **8v** afforded the product as a yellow oil, yield 45%, 685 mg [70] (HPLC: 87.1%). IR (NaCl, film) ν_max_: 2969 (C-H), 1749 (C=O, β-lactam), 1656 (C=C), 1602 (C=C), 1574 (C=C), 1513 (C=C), 1250 (C-O) cm^−1^. ^1^H NMR (400 MHz, CDCl_3_): δ 3.75–3.78 (m, 1H), 3.82 (s, 3H), 4.77 (d, *J* = 2.52 Hz, 1H), 5.15–5.17 (m, 1H), 5.23–5.27 (m, 1H), 5.89 (dd, *J*_5,6_ = 13.54 Hz, *J*_5,3_ = 8.02 Hz, 1H), 6.33–6.40 (m, 2H), 6.91–6.94 (m, 2H), 7.02–7.08 (m, 2H), 7.24–7.33 (m, 5H). ^13^C NMR (100 MHz, CDCl_3_): δ 54.88, 60.96, 62.99, 114.14, 116.65, 118.01, 123.51, 126.74, 128.61, 128.65, 129.21, 134.98, 135.51, 137.12, 159.36, 165.14. HRMS (*m/z*) calculated for C_20_H_20_NO_2_ [M+H]^+^: 306.1494, found 306.1496.

### 3.5. General Method IV: Preparation of Fmoc-Protected β-Lactams (***12a***-***e***)

To a stirred solution of the amino β-lactam (4.76 mmol) in anhydrous DMF (30 mL) were added DCC (5.7 mmol), Fmoc-protected amino acid (5.6 mmol) and HOBt.H_2_O (7.3 mmol) at room temperature. The mixture was stirred overnight. After 24 h, ethyl acetate (50 mL) was added, and the mixture was then filtered. The DMF was removed by washing with water (5 × 50 mL). The organic solvent was removed *in vacuo*, and the product was isolated by flash column chromatography over silica gel (eluent, dichloromethane: methanol gradient).

(1-((2-Methoxy-5-(4-oxo-3-(prop-1-en-2-yl)-1-(3,4,5-trimethoxyphenyl)azetidin-2-yl) phenyl)amino)-1-oxo-3-phenylpropan-2-yl)carbamaic acid 9*H*-fluoren-9-ylmethyl ester (**12a**)

Following the general method IV above, to a stirred solution of β-lactam **9s** (4.76 mmol) in anhydrous DMF (30 mL) were added DCC (5.7 mmol), Fmoc-phenylalanine (5.6 mmol) and HOBt.H_2_O (7.3 mmol) at room temperature. The product was isolated by flash column chromatography over silica gel (eluent: dichloromethane: methanol gradient) as a colourless solid, yield 32%, 229 mg, Mp 132–133 °C. IR (KBr) ν_max_: 3321 (NH), 1725 (C=O), 1650 (C=O), 1598 (C=C), 1507 (C=C) cm^−1^. ^1^H NMR (400 MHz, CDCl_3_): δ 1.90 (s, 3H), 3.19 (br s, 2H), 3.74 (s, 6H), 3.75 (s, 3H), 3.78 (s, 3H), 3.87 (br s, 1H), 4.22 (t, *J* = 7.02 Hz, 1H), 4.40–4.46 (m, 2H), 4.61–4.63 (m, 1H), 4.79 (br s, 1H), 5.03 (br s, 1H), 5.09 (br s, 1H), 6.60 (s, 2H), 6.82–7.79 (m, 16H), 8.06 (br s, 1H), 8.48 (s, 1H). ^13^C NMR (100 MHz, CDCl_3_): δ 20.12, 38.20, 46.63, 55.35, 55.63, 60.50, 59.84, 61.39, 66.36, 66.83, 94.29, 110.15, 114.12, 117.66, 119.59, 120.65, 124.53, 126.64, 126.75, 127.35, 128.39, 128.80, 128.84, 129.60, 133.30, 133.88, 137.62, 140.85, 143.14, 143.19, 147.53, 153.03, 156.71, 164.86, 168.49. HRMS (*m/z*) calculated for C_46_H_45_N_3_O_8_Na (M^+^+Na): 790.3104, found 790.3113.

(1-((5-(3-Allyl-4-oxo-1-(3,4,5-trimethoxyphenyl)azetidin-2-yl)-2-methoxyphenyl) amino)-1-oxo-3-phenylpropan-2-yl)carbamaic acid 9*H*-fluoren-9-ylmethyl ester (**12b**)

Following the general method IV above, the desired product was obtained from 3-allyl-4-(3-amino-4-methoxyphenyl)-1-(3,4,5-trimethoxyphenyl)azetidin-2-one **10r** and Fmoc-phenylalanine as a yellow oil (yield 20%). IR (NaCl) ν_max_: 3317 (NH), 1738 (C=O, β-lactam), 1693 (C=O) cm^−1^. ^1^H NMR (400 MHz, CDCl_3_): δ 2.47–2.63 (m, 2H), 3.19–3.27 (m, 2H), 3.45–3.51 (m, 1H), 3.72 (s, 6H), 3.74 (s, 3H), 3.77 (s, 3H), 4.21–4.59 (m, 3H), 4.64 (br s, 1H), 5.15–5.17 (m, 2H), 5.22 (m, 1H), 5.87–5.93 (m, 1H), 6.57 (s, 2H), 6.80–7.79 (m, 16H), 8.05 (br s, 1H), 8.44 (s, 1H). ^13^C NMR (100 MHz, CDCl_3_): δ 32.25, 33.30, 46.63, 48.96, 55.33, 55.61, 58.59, 59.97, 60.50, 66.82, 94.18, 110.04, 117.42, 119.59, 124.50, 126.64, 126.74, 126.81, 127.34, 128.38, 128.80, 128.84, 129.73, 132.60, 133.36, 133.43, 140.85, 143.19, 143.19, 147.44, 153.00, 166.67, 168.45, 170.76. HRMS (*m/z*) calculated for C_46_H_45_N_3_O_8_Na [M+Na]^+^: 790.3104, found 790.3094.

(1-((5-(3-Allyl-4-oxo-1-(3,4,5-trimethoxyphenyl)azetidin-2-yl)-2-methoxyphenyl) amino)-3-methyl-1-oxo-butan-2-yl) carbamaic acid 9*H*-fluoren-9-ylmethyl ester (**12c**)

Following the general method IV above, the desired product was obtained from 3-allyl-4-(3-amino-4-methoxyphenyl)-1-(3,4,5-trimethoxyphenyl)azetidin-2-one **10r** and Fmoc valine as a yellow oil (yield 18%). IR (NaCl) ν_max_: 3336 (NH), 2935 (C-H), 1724 (C=O, β-lactam), 1669 (C=O), 1607 (C=C) cm^−1^. ^1^H NMR (400 MHz, CDCl_3_): δ 1.02–1.04 (m, 6H), 2.23–2.62 (m, 2H), 2.69–2.72 (m, 1H), 3.25–3.27 (m, 1H), 3.73 (s, 6H), 3.77 (s, 3H), 3.86 (s, 3H), 4.24–4.43 (m, 4H), 4.65 (br s, 1H), 5.14–5.21 (m, 2H), 5.85–5.92 (m, 1H), 6.58 (s, 2H), 6.86–7.79 (m, 11H), 8.19 (br s, 1H), 8.48 (s, 1H). ^13^C NMR (100 MHz, CDCl_3_): δ 18.78, 32.21, 33.20, 46.72, 55.47, 55.60, 58.56, 59.98, 60.48, 62.43, 65.58, 66.76, 94.21, 110.15, 117.46, 119.58, 120.74, 124.53, 124.65, 126.63, 127.25, 127.32, 128.43, 129.90, 132.60, 133.36, 133.46, 140.86, 143.19, 143.30, 147.63, 153.00, 156.05, 166.74, 170.82. HRMS (*m/z*) calculated for C_42_H_45_N_3_O_8_Na [M+Na]^+^: 742.3104, found 742.3100.

(1-((5-(3-(Buta-1,3-dien-1-yl)-4-oxo-1-(3,4,5-trimethoxyphenyl)azetidin-2-yl)-2-methoxyphenyl)amino)-3-methyl-1-oxo-butan-2-yl) carbamaic acid 9*H*-fluoren-9-ylmethyl ester (**12d**)

Following the general method IV above, the desired product was obtained from *(E)*-3-(buta-1,3-dienyl)-4-(3-amino-4-methoxyphenyl)-1-(3,4,5-trimethoxyphenylazetidin-2-one **11r** and Fmoc valine as a yellow resin (yield 85%); IR (NaCl) ν_max_: 3400 (NH), 1745 (C=O, β-lactam), 1679 cm^−1^ (C=O) cm^−1^. ^1^H NMR (400 MHz, CDCl_3_): δ 1.02–1.05 (m, 6H), 2.53–2.65 (m, 1H), 3.74 (s, 6H), 3.77 (s, 3H), 3.83 (br s, 1H), 3.86 (s, 3H), 4.25–4.49 (m, 4H), 4.74 (br s, 1H), 5.14–5.17 (m, 1H), 5.23–5.27 (m, 1H), 5.85–5.90 (m, 1H), 6.31–6.39 (m, 2H), 6.59 (s, 2H), 6.84–7.79 (m, 9H), 8.20 (br s, 1H), 8.52 (br s, 1H). ^13^C NMR (100 MHz, CDCl_3_): δ 18.80, 30.70, 46.71, 55.62, 55.65, 60.50, 59.97, 60.99, 62.46, 65.89, 66.76, 94.34, 110.21, 117.87, 118.04, 119.58, 124.52, 125.08, 126.64, 127.33, 128.39, 129.26, 132.60, 133.33, 133.95, 134.91, 135.51, 140.86, 143.19, 147.63, 153.03, 155.99, 164.96, 169.06. HRMS (*m/z*) calculated for C_43_H_45_N_3_O_8_Na [M+Na]^+^: 754.3104, found 754.3132.

(2-((5-(*(E)*-3-(Buta-1,3-dien-1-yl)-4-oxo-1-(3,4,5-trimethoxyphenyl)azetidin-2-yl)-2-methoxyphenyl)amino)-2-oxo-ethyl) carbamaic acid 9*H*-fluoren-9-ylmethyl ester (**12e**)

Following the general method IV above, the desired product was obtained from *(E)*-3-(buta-1,3-dienyl)-4-(3-amino-4-methoxyphenyl)-1-(3,4,5-trimethoxyphenyl)azetidin-2-one **11r** and Fmoc glycine as a colourless solid (yield 49%); mp: 167–168 °C; IR (NaCl) ν_max_: 3333 (NH), 1727 (C=O, β-lactam), 1655 (C=O) cm^−1^. ^1^H NMR (400 MHz, CDCl_3_): δ 3.74 (s, 3H), 3.79 (s, 6H), 3.85 (s, 3H), 3.80–3.82 (m, 1H), 4.08 (br s, 2H), 4.27–4.29 (m, 1H), 4.47–4.49 (m, 2H), 4.75 (br s, 1H), 5.15–5.17 (m, 1H), 5.23–5.27 (m, 1H), 5.87 (dd, *J*_5,6_ = 13.69 Hz, *J*_5,3_ = 8.28 Hz, 1H), 6.33–6.39 (m, 2H), 6.58 (s, 2H), 6.88–7.29 (m, 3H), 7.34–7.81 (m, 8H), 8.29 (br s, 1H), 8.49 (s, 1H). ^13^C NMR (100 MHz, CDCl_3_): δ 44.65, 46.61, 55.49, 55.65, 60.51, 61.39, 62.62, 67.00, 94.30, 110.21, 118.07, 119.62, 120.66, 124.53, 125.05, 126.66, 127.37, 129.34, 133.31, 133.95, 134.96, 135.49, 140.86, 143.18, 147.52, 153.03, 156.78, 164.93, 166.47. HRMS (*m/z*) calculated for C_40_H_39_N_3_O_8_Na [M+Na]^+^: 712.2635, found 712.2627.

### 3.6. General Method V: Synthesis of Amino Acid Prodrugs (***13a***-***e***)

To the appropriate amino acid amide **12a**-**e** (1.56 mmol) in methanol (10 mL)/CH_2_Cl_2_ (10 mL) was added 2N NaOH (3.4 mmol) at room temperature, and the mixture was stirred for 24 h. Saturated aq. NaHCO_3_ was added, and the mixture was extracted with dichloromethane (25 mL × 3). The organic solution was dried, the solvent was removed, and the crude product was dissolved in diethyl ether and extracted with 2N HCl (5 × 50 mL). Then, 2N NaOH was added to neutralise the mixture, and the mixture was extracted with diethyl ether (5 × 50 mL). The organic solution was dried (Na_2_SO_4_), the solvent was removed, and the product was obtained by flash chromatography over silica gel (eluent, dichloromethane-methanol gradient).

2-Amino-*N*-(2-methoxy-5-(1-(3,4,5-trimethoxyphenyl)-4-oxo-3-(prop-1-en-2-yl) azetidin-2-yl)phenyl)-3-phenylpropanamide (**13a**)

Following the general method V above, to amino acid amide **10** (1.56 mmol) in methanol (10 mL)/CH_2_Cl_2_ (10 mL) was added 2N NaOH (3.4 mmol) at room temperature, and the mixture was stirred for 24 h. The product was isolated by flash chromatography over silica gel (eluent, dichloromethane-methanol gradient) as a yellow oil, yield 58% (HPLC: 99.1%). IR (NaCl) ν_max_: 3318 (NH_2_), 2917 (C-H), 1733 (C=O, β-lactam), 1678 (C=O), 1617 (C=C), 1598 (C=C), 1512 (C=C) cm^−1^. ^1^H NMR (400 MHz, CDCl_3_): δ 1.60 (br s, 3H, CH_3_), 3.42–3.52 (m, 2H), 3.78 (s, 9H), 3.83 (br s, 1H), 3.88 (s, 3H), 3.89 (br s, 1H), 5.32 (d, *J* = 3.48 Hz, 1H), 6.66 (s, 2H), 6.87–6.89 (m, 1H), 7.10–7.12 (m, 1H), 7.28–7.37 (m, 8H). ^13^C NMR (100 MHz, CDCl_3_): δ 19.37, 32.15, 51.31, 55.45, 55.57, 59.95, 60.03, 60.47, 94.16, 110.16, 117.42, 120.07, 125.07, 127.33, 127.43, 129.62, 133.34, 133.53, 133.67, 134.26, 147.83, 152.96, 166.79, 174.18. HRMS (*m/z*) calculated for C_31_H_34_N_3_O_6_ (M^+^-H): 544.2448, found 544.2455.

*N*-(5-(3-Allyl-1-(3,4,5-trimethoxyphenyl)-4-oxoazetidin-2-yl)-2-methoxyphenyl)-2-amino-3-phenylpropanamide (**13b**)

Preparation was as described in the general method V above from the Fmoc-protected β-lactam **12b**. The desired product was isolated as a brown oil, yield 37.6%, 139 mg (HPLC: 91.7%). IR (NaCl, film) ν_max_: 3343 (NH), 1743 (C=O, β-lactam), 1697 (C=O), 1598 (C=C), 1503 (C=C) cm^−1^. ^1^H NMR (400 MHz, CDCl_3_): δ 2.21–2.51 (m, 2H), 2.72–2.93 (m, 2H), 3.40–3.47 (m, 1H), 3.71 (s, 3H), 3.75 (s, 6H), 3.86 (s, 3H), 3.89 (m, 1H), 4.52 (br s, 1H), 5.02–5.09 (m, 2H), 5.68–5.77 (m, 1H), 5.82 (s, 2H), 6.60–7.37 (m, 8H). ^13^C NMR (100 MHz, CDCl_3_): δ 34.23, 34.28, 52.01, 55.32, 55.37, 58.79, 60.49, 65.42, 94.17, 109.43, 116.97, 117.28, 121.13, 126.47, 127.25, 127.28, 128.38, 128.82, 129.00, 133.30, 133.36, 134.26, 137.46, 147.22, 153.19, 165.57, 172.03. HRMS (*m/z*) calculated for C_31_H_34_N_3_O_6_ [M-H]^-^: 544.2448, found 544.2464.

*N*-(5-(3-Allyl-1-(3,4,5-trimethoxyphenyl)-4-oxoazetidin-2-yl)-2-methoxyphenyl)-2-amino-3-methylbutanamide (**13c**)

Preparation was as described in the general method V above from Fmoc-protected β-lactam **12c.** The desired product was isolated as a yellow oil, yield 59%, 59 mg (HPLC: 92.3%). IR (NaCl, film) ν_max_: 3343 (NH), 1746 (C=O, β-lactam), 1674 (C=O), 1607 (C=C), 1523 (C=C) cm^−1^. ^1^H NMR (400 MHz, CDCl_3_): δ 0.88–1.07 (m, 6H), 2.44–2.63 (m, 2H), 2.67–2.68 (m, 1H), 3.26–3.29 (m, 1H), 3.73 (s, 6H), 3.76 (s, 3H), 3.90 (s, 3H), 4.14 (br s, 1H), 4.65 (br s, 1H), 5.12–5.20 (m, 2H), 5.81–5.94 (m, 1H), 6.58 (s, 2H), 6.79–7.05 (m, 3H). ^13^C NMR (100 MHz, CDCl_3_): δ 19.30, 30.43, 32.15, 55.45, 55.57, 58.53, 59.96, 60.03, 60.47, 94.17, 110.16, 117.42, 117.98, 120.07, 127.33, 133.34, 133.40, 133.53, 133.67, 134.26, 147.83, 153.17, 166.75, 174.18. HRMS (*m/z*) calculated for C_27_H_34_N_3_O_6_ [M-H]^-^: 496.2448, found 496.2437.

2-Amino-*N*-(5-(3-((*E*)-buta-1,3-dienyl)-1-(3,4,5-trimethoxyphenyl)-4-oxoazetidin-2-yl)-2-methoxyphenyl)-3-methylbutanamide (**13d**)

Preparation was as described in the general method V above from Fmoc-protected β-lactam **12d**. The desired product was isolated as a yellow oil, yield 49%, 102 mg (HPLC: 96.9%). IR (NaCl, film) ν_max_: 3321 (NH), 2931 (C-H), 1734 (C=O, β-lactam), 1681 (C=O), 1612 (C=C), 1598 (C=C), 1513 (C=C) cm^−1^. ^1^H NMR (400 MHz, CDCl_3_): δ 1.04–1.06 (m, 6H), 2.40–2.41 (m, 1H), 3.76 (br s, 3H), 3.77 (br s, 1H), 3.88 (s, 6H), 3.89 (br s, 1H), 3.90 (s, 3H), 5.44 (br s, 1H), 6.24–6.29 (m, 1H), 6.66 (s, 2H), 6.82–7.42 (m, 7H). ^13^C NMR (100 MHz, CDCl_3_): δ 18.67, 30.44, 55.56, 55.63, 60.47, 59.94, 60.61, 62.71, 93.89, 110.47, 119.01, 119.34, 124.90, 126.91, 129.51, 132.60, 133.16, 133.95, 134.87, 135.35, 147.03, 153.29, 161.70, 172.36. HRMS (*m/z*) calculated for C_28_H_36_N_3_O_6_ [M+H]^+^: 510.2604, found 510.2607.

2-Amino-*N*-(5-(3-(buta-1,3-dienyl)-1-(3,4,5-trimethoxyphenyl)-4-oxoazetidin-2-yl)-2-methoxyphenyl)acetamide (**13e**)

Preparation was as described in the general method V above from Fmoc-protected β-lactam **12e**. The desired product was isolated as a yellow oil, yield 56%, 68 mg (HPLC: 90.0%). IR (NaCl, film) ν_max_: 3335 (NH), 1754 (C=O, β-lactam), 1655 (C=O), 1567 (C=C), 1503 (C=C) cm^−1^. ^1^H NMR (400 MHz, CDCl_3_): δ 3.48–3.52 (m, 2H), 3.55 (s, 6H), 3.69 (s, 3H), 3.78 (s, 3H), 3.82–3.83 (m, 1H), 5.32 (d, *J* = 1.48 Hz, 1H), 5.85–6.33 (m, 5H), 6.65 (s, 2H), 6.84–7.42 (m, 3H). ^13^C NMR (100 MHz, CDCl_3_): δ 45.20, 55.41, 55.62, 60.49, 60.62, 62.65, 94.31, 110.46, 117.44, 119.01, 120.97, 125.73, 133.20, 133.73, 134.97, 135.45, 147.90, 152.99, 161.70, 167.02. HRMS (*m/z*) calculated for C_25_H_28_N_3_O_6_ [M-H]^-^: 466.1978, found 466.1971.

### 3.7. 2-Methoxy-5-(1-(3,4,5-trimethoxyphenyl)-4-oxo-3-(prop-1-en-2-yl)azetidin-2-yl) phenyl dibenzyl phosphate (***14a***)

To a solution of phenol **9q** (17 mmol) in acetonitrile (100 mL), cooled to 0 °C, was added carbon tetrachloride (85 mmol). The solution was stirred for 10 min prior, and then diisopropylethylamine (35 mmol) and dimethylaminopyridine (1.7 mmol) were added. The dibenzyl phosphate (24.5 mmol) was then added dropwise to the mixture. When the reaction was complete, 0.5M KH_2_PO_4_ (aq) was added and the reaction mixture was warmed to room temperature. The mixture was extracted with ethyl acetate (3 × 50 mL), washed with saturated sodium chloride (aqueous, 100 mL) followed by water (100 mL) and dried (Na_2_SO_4_). The solvent was reduced *in vacuo*, and the product was isolated by flash column chromatography over silica gel (*n*-hexane: ethyl acetate gradient). Yield: 45%, 342 mg, brown oil. IR (NaCl, film) ν_max_: 2940 (C-H), 1740 (C=O, β-lactam), 1507 (C=C), 1304 (P=O), 1231 (C-O) cm^−1^. ^1^H NMR (400 MHz, CDCl_3_): δ 1.86 (s, 3H), 3.68 (d, *J* = 2.04 Hz, 1H), 3.72 (s, 6H), 3.76 (s, 3H), 3.82 (s, 3H), 4.68 (d, *J* = 2.52 Hz, 1H), 5.02 (br s, 1H), 5.05 (br s, 1H), 5.13–5.17 (m, 4H), 6.54 (s, 2H), 6.94–7.21 (m, 3H), 7.31–7.36 (m, 10H). ^13^C NMR (100 MHz, CDCl_3_): δ 20.08, 55.55, 55.60, 59.32, 60.47, 66.37, 69.44, 69.50, 69.55, 94.17, 112.82, 114.22, 119.21, 119.24, 122.70, 127.40, 127.48, 128.15, 128.21, 129.66, 133.17, 133.96, 135.12, 137.43, 139.57, 150.42, 153.07, 164.64. HRMS (*m/z*) calculated for C_36_H_39_NO_9_P (M^+^+H): 660.2362, found 660.2372.

5-(3-Allyl-1-(3,4,5-trimethoxyphenyl)-4-oxoazetidin-2-yl)-2-methoxyphenyl dibenzyl phosphate (**14b**)

Following the procedure described above for **14a**, to a solution of the phenol 3-allyl-4-(3-hydroxy-4-methoxyphenyl)-1-(3,4,5-trimethoxyphenyl)azetidin-2-one **10r** (17 mmol) in acetonitrile (100 mL), cooled to 0 °C, was added carbon tetrachloride (85 mmol). The mixture was treated with diisopropylethylamine (35 mmol), N,N-dimethylaminopyridine (1.7 mmol) and dibenzyl phosphate (24.5 mmol). The product was isolated by flash column chromatography over silica gel (eluent, *n*-hexane: ethyl acetate gradient) as a brown oil (yield 27%), 325 mg. IR (NaCl, film) ν_max_: 1746 (C=O, β-lactam), 1503 (C=C), 1300 (P=O), 1235 (C-O), 1126 cm^−1^. ^1^H NMR (400 MHz, CDCl_3_): δ 2.51–2.58 (m, 1H), 2.65–2.71 (m, 1H), 3.14–3.18 (m, 1H), 3.71 (s, 6H), 3.76 (s, 3H), 3.81 (s, 3H), 4.54 (br s, 1H), 5.12–5.14 (m, 2H), 5.15–5.18 (m, 4H), 5.80–5.88 (m, 1H), 6.53 (s, 2H), 6.92–7.21 (m, 3H), 7.33–7.35 (m, 10H). ^13^C NMR (100 MHz, CDCl_3_): δ 32.21, 55.53, 55.58, 58.67, 59.50, 60.47, 69.40, 69.46, 69.52, 94.07, 112.72, 117.43, 119.32, 122.85, 127.38, 127.47, 128.14, 128.19, 129.81, 133.33, 133.37, 133.86, 135.06, 139.51, 139.58, 150.36, 153.05, 166.43. HRMS (*m/z*) calculated for C_36_H_39_NO_9_P [M+H]^+^: 660.2362, found 660.2375.

### 3.8. 2-Methoxy-5-(4-oxo-3-(prop-1-en-2-yl)-1-(3,4,5-trimethoxyphenyl)azetidin-2-yl)-phenyl dihydrogen phosphate (***15a***)

Dibenzyl phosphate ester **14a** (0.27 mmol) was dissolved in dry dichloromethane (5 mL) under nitrogen at 0 °C. Bromotrimethylsilane (0.59 mmol) was added to reaction mixture and allowed to stir for 45 min. Sodium thiosulphate solution (10%, 5 mL) was added to the reaction, and stirring was continued for 5 min. The aqueous phase was extracted with ethyl acetate (3 × 25 mL). The combined organic phases were concentrated *in vacuo* and purified by flash chromatography on silica gel (eluent: *n*-hexane: ethyl acetate, 1:1) to afford the product as an off-yellow solid, yield 57%, Mp > 300 °C (HPLC: 91.7%). IR (KBr) ν_max_: 3452 (OH), 1742 (C=O, β-lactam), 1275 (P=O) cm^−1^. ^1^H NMR (400 MHz, DMSO-*d_6_*): δ 1.86 (s, 3H), 3.52 (s, 3H), 3.59 (s, 6H), 3.68 (s, 3H), 4.91–5.06 (m, 4H, H-3, H-4), 6.52 (s, 2H), 6.90–7.15 (m, 3H). ^13^C NMR (100 MHz, DMSO-*d_6_*): δ 20.53, 55.85, 56.17, 59.17, 60.49, 66.53, 94.93, 111.46, 114.82, 120.31, 129.00, 129.24, 133.89, 134.38, 137.01, 139.42, 152.46, 153.53, 165.52. HRMS (*m/z*) calculated for C_22_H_25_NO_9_P (M^+^-H): 478.1267, found 478.1239.

### 3.9. 5-(3-Isopropyl-1-(3,4,5-trimethoxyphenyl)-4-oxoazetidin-2-yl)-2-methoxyphenyl dihydrogen phosphate (***15b***)

The dibenzylphosphate ester **14a** (2 mmol) was dissolved in ethanol: ethyl acetate (50 mL; 1:1 mixture) and hydrogenated over 1.2 g of 10% palladium on carbon until complete on TLC, typically for less than 3 h. The catalyst was filtered, the solvent was removed *in vacuo*, and the product was isolated by flash column chromatography over silica gel (eluent, *n*-hexane: ethyl acetate gradient) to afford the desired product as a brown oil, 239 mg, yield 98% (HPLC: 73.9%). IR (NaCl, film) ν_max_: 3472 (OH), 2961 (C-H), 1738 (C=O), 1593 (C=C), 1300 (P=O), 1238 (C-O) cm^−1^. ^1^H NMR (400 MHz, CDCl_3_): δ 1.00–1.08 (m, 6H), 2.06 (br s, 1H), 2.98–2.99 (m, 1H), 3.68 (s, 6H), 3.73 (s, 6H), 4.64 (br s, 1H), 6.53 (s, 2H), 6.86–7.35 (m, 3H). ^13^C NMR (100 MHz, CDCl_3_): δ 19.41, 19.77, 28.21, 55.56, 58.20, 60.43, 66.05, 94.44, 112.57, 118.48, 122.60, 130.11, 132.93, 133.95, 137.59, 145.25, 152.97, 170.81. HRMS (*m/z*) calculated for C_22_H_27_NO_9_P (M^+^-H): 480.1423, found 480.1419.

### 3.10. 5-(3-Allyl-1-(3,4,5-trimethoxyphenyl)-4-oxoazetidin-2-yl)-2-methoxyphenyl dihydrogen phosphate (***15c***)

The dibenzyl phosphate ester **14b** (0.27 mmol) was dissolved in dry dichloromethane (5 mL) under nitrogen at 0 °C. Bromotrimethylsilane (0.59 mmol) was added to reaction mixture and stirred for 45 min. Sodium thiosulphate solution (10%, 5 mL) was added to the reaction, and stirring was continued for 5 min. The aqueous phase was extracted with ethyl acetate (3×25 mL), and the combined organic phases were concentrated *in vacuo*. Purification by flash chromatography on silica gel (eluent, 1:1 *n*-hexane: ethyl acetate) afforded the product as brown oil (yield 96%), IR (NaCl) ν_max_: 3483 (OH), 2938 (C-H), 1743 (C=O, β-lactam), 1592 (C=C), 1302 (P=O), 1237 (C-O) cm^−1^. ^1^H NMR (400MHz, CDCl_3_): δ 2.48–2.61 (m, 2H, CH_2_), 3.23 (br s, 1H, H-3), 3.68 (s, 6H, OCH_3_), 3.72 (s, 6H, OCH_3_), 4.58 (br s, 1H, H-4), 5.05–5.12 (m, 2H, CH_2_), 5.78–5.80 (m, 1H, CH), 6.51 (s, 2H, ArH), 6.85–7.54 (m, 3H, ArH). ^13^C NMR (100MHz, CDCl_3_): δ 32.09, 55.46, 55.55, 58.32 (OCH_3_), 59.64 (C-3), 60.44 (C-4), 94.30, 112.67, 117.44 (CH_2_), 119.27, 122.90, 129.18, 133.10, 133.27 (CH), 133.90, 139.86, 150.38, 152.99, 167.37 (C=O). HRMS (*m/z*) calculated for C_22_H_26_NO_9_PNa [M+Na]^+^: 502.1243, found 502.1226.

### 3.11. 2-Methoxy-5-(1-(3,4,5-trimethoxyphenyl)-4-oxo-3-propylazetidin-2-yl)phenyl dihydrogen phosphate (***15d***)

The dibenzyl phosphate ester **14b** (2 mmol) was dissolved in ethanol: ethyl acetate (50 mL; 1:1 mixture) and hydrogenated over 1.2 g of palladium (10%) on carbon until complete as monitored by TLC, approx. 3 h. The catalyst was filtered, the solvent was reduced *in vacuo*, and the product was isolated by flash column chromatography over silica gel (eluent, *n*-hexane: ethyl acetate gradient) to afford the product as a brown oil, yield 88%, 106 mg, IR (NaCl, film) ν_max_: 3492 (OH), 2933 (C-H), 1743 (C=O, β-lactam), 1592 (C=C), 1311 (P=O), 1231 (C-O) cm^−1^. ^1^H NMR (400 MHz, CDCl_3_): δ 0.94–0.96 (m, 3H), 1.42–1.44 (m, 2H), 1.71–1.81 (m, 2H), 3.15 (br s, 1H), 3.65 (s, 6H), 3.71 (s, 6H), 4.56 (br s, 1H), 6.51 (s, 2H), 6.83–7.40 (m, 3H). ^13^C NMR (100 MHz, CDCl_3_): δ 13.46, 19.85, 30.28, 55.41, 55.53, 59.45, 60.37, 60.50, 94.29, 112.40, 118.70, 122.13, 130.15, 133.23, 133.79, 140.59, 149.96, 152.95, 168.23. HRMS (*m/z*) calculated for C_22_H_28_NO_9_PNa [M+Na]^+^: 504.1399, found 504.1374.

### 3.12. General Method VI: Preparation of 3-Substituted β-lactams (***17a***-***c***, ***f***, ***g***)

The appropriate 3-unsubstituted β-lactam **16a**-**c** (0.8 mmol) was dissolved in dry THF (7 mL) under N_2_ atmosphere and cooled to −78 °C (dry ice and acetone). To this stirring solution, LDA 2.0 M solution (1.6 mmol) was added all at once and the reaction left to stir for 5 min prior to the dropwise addition of 3-bromo-1-phenylpropene or ethyl bromoacetate (1.2 mmol) in dry THF (2 mL). The reaction was allowed to stir at −78 °C for 30 min and then allowed to stir at room temperature for 5 min before being poured into saturated brine (50 mL). Following extraction with ethyl acetate (2 × 50 mL), the solution was dried over anhydrous sodium sulphate. Solvent was removed under reduced pressure, and the residue was purified by flash chromatography over silica gel (eluent, *n*-hexane: ethyl acetate, 4:1) to yield the title compound.

3-Cinnamyl-4-(4-methoxyphenyl)-1-(3,4,5-trimethoxyphenyl)azetidin-2-one (**17a**)

Preparation was as described in the general method VI above from β-lactam **16a** and 3-bromo-1-phenylpropene. The product was obtained as a yellow solid, yield: 39%, 145 mg. Mp 103–104 °C (HPLC: 95.0%). IR (KBr) ν_max_: 2937 (OH), 1747 (C=O, β-lactam), 1610 (C=C), 1590 (C=C), 1504 (C=C), 1247 (C-O) cm^−1^. ^1^H NMR (400 MHz, CDCl_3_): δ 2.69–2.77 (m, 1H), 2.87–2.93 (m, 1H), 3.28–3.32 (m, 1H), 3.73 (s, 6H), 3.78 (s, 3H), 3.83 (s, 3H), 4.69 (d, *J* = 2.28 Hz, 1H), 6.24–6.31 (m, 1H), 6.51 (d, *J* = 15.80 Hz, 1H), 6.55 (s, 2H), 6.92 (d, *J* = 8.56 Hz, 2H), 7.29–7.34 (m, 7H). ^13^C NMR (100 MHz, CDCl_3_): δ 31.68, 54.88, 55.53, 59.06, 60.16, 60.51, 94.16, 114.10, 125.15, 125.74, 126.89, 127.01, 128.14, 129.15, 132.07, 133.49, 133.80, 136.48, 153.00, 159.27, 166.65. HRMS (*m/z*) calculated for C_28_H_30_NO_5_ [M+H]^+^: 460.2124, found 460.2115.

3-Cinnamyl-4-(3-hydroxy-4-methoxyphenyl)-1-(3,4,5-trimethoxyphenyl)azetidin-2-one (**17b**)

Preparation was as described in the general method VI above from β-lactam **16b** and 3-bromo-1-phenylpropene. The product was afforded as a yellow oil, yield 14%, 33 mg (HPLC: 80.9%). IR (NaCl, film) ν_max_: 3333 (OH), 2934 (C-H), 1737 (C=O, β-lactam), 1650 (C=O), 1598 (C=C), 1502 (C=C), 1267 (C-O) cm^−1^. ^1^H NMR (400 MHz, CDCl_3_): δ 2.68–2.79 (m, 1H), 2.88–2.94 (m, 1H), 3.60–3.62 (m, 1H), 3.73 (s, 3H), 3.80 (s, 6H), 3.85 (s, 3H), 4.72 (br s, 1H), 6.23–6.34 (m, 1H), 6.57 (s, 2H), 6.68–6.76 (m, 1H), 6.85–7.34 (m, 8H). HRMS (*m/z*) calculated for C_28_H_29_NO_6_Na [M+Na]^+^: 498.1893, found 498.1885.

3-Cinnamyl-1,4-diphenylazetidin-2-one (**17c**)

Preparation was as described in the general method VI above from β-lactam **16c** and 3-bromo-1-phenylpropene. The product was obtained as a brown oil, yield 90%, 307 mg (HPLC: 76.7%). IR (NaCl, film) ν_max_: 2932 (C-H), 1747 (C=O, β-lactam), 1507 (C=C), 1234 (C-O) cm^−1^. ^1^H NMR (400 MHz, CDCl_3_): δ 3.78–3.79 (m, 1H), 4.18–4.20 (m, 1H), 4.78 (br s, 1H), 6.25–6.27 (m, 1H), 6.65–6.69 (m, 1H), 7.29–7.40 (m, 15H). HRMS (*m/z*) calculated for C_24_H_22_NO [M+H]^+^: 340.1701, found 340.1696.

Ethyl-2-(1-(3,4,5-trimethoxyphenyl)-2-(4-methoxyphenyl)-4-oxoazetidin-3-yl)acetate (**17f**)

Preparation was as described in the general method VI above from β-lactam **16a** and ethyl bromoacetate. The product was obtained as a brown oil, yield 64%, 131 mg (HPLC: 93.4%). IR (NaCl, film) ν_max_: 2945 (C-H), 1746 (C=O, β-lactam and acetate), 1589 (C=C), 1507 (C=C), 1245 (C-O) cm^−1^. ^1^H NMR (400 MHz, CDCl_3_): δ 1.18–1.30 (m, 3H), 2.91–2.95 (m, 2H), 3.48–3.53 (m, 1H), 3.70 (s, 6H), 3.74 (s, 3H), 3.79 (s, 3H), 4.16–4.24 (m, 2H), 4.91 (br s, 1H), 6.53 (s, 2H), 6.90 (d, *J* = 8.52 Hz), 7.31 (d, *J* = 8.52 Hz, 2H). ^13^C NMR (100 MHz, CDCl_3_): δ 13.53 (CO_2_CH_2_CH_3_), 46.85 (C-5), 53.62, 54.84, 55.50, 60.52, 60.91, 66.57, 93.96, 114.06, 126.84, 129.51, 133.62, 133.73, 152.96, 159.33, 164.16, 170.28. C_23_H_27_NO_7_Na [M+Na]^+^: 452.1685, found 452.1688.

Ethyl-2-(2-(3-hydroxy-4-methoxyphenyl)-1-(3,4,5-trimethoxyphenyl)-4-oxoazetidin-3-yl)acetate (**17g**)

Preparation was as described in the general method VI above from β-lactam **16b** and ethyl bromoacetate. The product was isolated as a brown oil, yield 77%, 275 mg (HPLC: 72.3%). IR (NaCl, film) ν_max_: 3300 (OH), 2938 (C-H), 1747 (C=O, β-lactam and acetate), 1591 (C=C), 1507 (C=C), 1237 (C=C) cm^−1^. ^1^H NMR (400 MHz, CDCl_3_): δ 1.23–1.28 (m, 3H), 2.90–2.96 (m, 1H), 3.50–3.55 (m, 1H), 3.73 (s, 6H), 3.77 (s, 3H), 3.89 (s, 3H), 4.10–4.18 (m, 3H, H_3_), 4.89–4.91 (m, 1H), 5.78 (br s, 1H), 6.53 (s, 2H), 6.84–7.02 (m, 3H). ^13^C NMR (100 MHz, CDCl_3_): δ 14.19, 46.85, 54.11, 54.18, 56.04, 60.40, 60.91, 66.53, 94.52, 111.99, 112.38, 120.24, 130.50, 134.02, 134.37, 147.83, 149.97, 153.48, 164.52, 168.52. HRMS (*m/z*) calculated for C_23_H_27_NO_8_Na [M+Na]^+^: 468.1634, found 468.1654.

### 3.13. General Method VII: Preparation of Compounds ***17d***, ***17e*** and ***19***

A solution of 1-bromo-4-(trifluoromethyl)benzene (2.75 mmol), palladium acetate (0.38 mmol), triphenylphosphine (0.41 mmol), potassium acetate (oven dried, 11.0 mmol), anhydrous tetrabutylammonium chloride (2.75 mmol) and appropriate β-lactam **10f, 10s** and **18** (2 mmol) in DMF (50 mL) under N_2_ was heated to 80 °C for 18 h. The solution was cooled, diluted with ethyl acetate, washed with water (3 × 50 mL), dried and concentrated *in vacuo.* The crude product mixture was purified by flash chromatography (eluent, *n*-hexane: ethyl acetate, 4:1).

3-(4-(Trifluoromethyl)cinnamyl)-4-(4-methoxyphenyl)-1-(3,4,5-trimethoxyphenyl) azetidin-2-one (**17d**)

Preparation as described in the general method VII above from β-lactam **10f** and 1-bromo-4-(trifluoromethyl)benzene afforded the product as a brown oil, yield 72%, 754 mg (HPLC: 81.8%). IR (NaCl, film) ν_max_: 1747 (C=O, β-lactam), 1677 (C=C), 1507 (C=C), 1320 (C-CF_3_), 1248 (C-O) cm^−1^. ^1^H NMR (400 MHz, CDCl_3_): δ 2.76–2.84 (m, 1H), 2.93–3.00 (m, 1H), 3.33–3.37 (m, 1H), 3.76 (s, 6H), 3.82 (s, 3H), 3.87 (s, 3H), 4.71 (d, *J* = 2.00 Hz, 1H), 6.39–6.46 (m, 1H), 6.55 (br s, 1H), 6.59 (s, 2H), 6.96 (d, *J* = 9.04 Hz, 2H), 7.33 (d, *J* = 8.00 Hz, 2H), 7.46 (d, *J* = 8.52 Hz, 2H), 7.61 (d, *J* = 8.56 Hz, 2H). ^13^C NMR (100 MHz, CDCl_3_): δ 31.72, 54.89, 55.53, 58.82, 60.26, 60.50, 94.16, 114.16, 125.08, 125.12, 125.86, 126.74, 126.85, 128.04, 128.97, 130.78, 133.40, 133.89, 139.88, 153.03, 159.37, 166.36. HRMS (*m/z*) calculated for C_29_H_28_F_3_NO_5_Na [M+Na]^+^: 550.1817, found 550.1816.

3-(4-(Trifluoromethyl)cinnamyl)-4-(4-methoxyphenyl)-1-phenylazetidin-2-one (**17e**)

Preparation as described in the general method VII above from β-lactam **10s** and 1-bromo-4-(trifluoromethyl)benzene afforded the product as a brown oil, yield 28%, 246 mg (HPLC: 92.1%). IR (NaCl, film) ν_max_: 1740 (C=O, β-lactam), 1589 (C=C), 1507 (C=C), 1339 (C-CF_3_), 1278 (C-O) cm^−1^. ^1^H NMR (400 MHz, CDCl_3_): δ 2.72–2.80 (m, 1H), 2.90–2.96 (m, 1H), 3.28–3.32 (m, 1H), 3.82 (s, 3H), 4.71 (d, *J* = 2.48 Hz, 1H), 6.35–6.42 (m, 1H), 6.54 (d, *J* = 16.80 Hz, 1H), 6.91 (d, *J* = 8.52 Hz, 2H), 7.04–7.08 (m, 2H), 7.24–7.60 (m, 9H). ^13^C NMR (100 MHz, CDCl_3_): δ 31.70, 54.87, 58.96, 59.87, 114.04, 114.14, 116.58, 123.46, 125.08, 125.86, 126.76, 127.69, 128.11, 128.61, 128.70, 130.77, 137.14, 139.89, 159.28, 166.55. HRMS (*m/z*) calculated for C_26_H_22_F_3_NO_2_Na [M+Na]^+^: 460.1500, found 460.1519.

3-(4-(Trifluoromethyl)styryl)-1-(3,4,5-trimethoxyphenyl)-4-(4-methoxyphenyl)azetidin-2-one (**19**)

Preparation as described in the general method VII above from β-lactam **18** and 1-bromo-4-(trifluoromethyl)benzene afforded the product as a yellow oil, yield 19%, 191 mg (HPLC: 82.6%). IR (NaCl, film) ν_max_: 1737 (C=O, β-lactam), 1588 (C=C), 1506 (C=C), 1326 (C-CF_3_), 1279 (C-O) cm^−1^**.** ^1^H NMR (400 MHz, CDCl_3_): δ 3.76 (s, 6H), 3.78 (s, 3H), 3.85 (s, 3H), 4.56–4.57 (m, 1H), 4.79 (d, J = 2.00 Hz, 1H), 6.60–6.64 (m, 1H), 6.75 (s, 2H), 6.93–6.96 (m, 1H), 7.18–7.49 (m, 8H). HRMS (*m/z*) calculated for C_28_H_27_F_3_NO_5_ [M+H]^+^: 514.1841, found 514.1845.

### 3.14. 1-(3,4,5-Trimethoxyphenyl)-3-((oxiran-2-yl)methyl)-4-phenylazetidin-2-one (***20***)

To a stirred solution of β-lactam **10f** (0.18 mmol, 1 eq) in DCM (1.3 mL) was added a solution of mCPBA (3 eq) dissolved in DCM (0.5 mL). The resulting mixture was stirred at room temperature for 24 h. The reaction was diluted with DCM (10 mL) and washed with 50 mL each of saturated NaHCO_3_ and H_2_O. The organic layer was collected and dried over anhydrous Na_2_SO_4_, and the solvent removed under reduced pressure. Further purification was performed by flash column chromatography over silica gel eluted with 2:1 *n*-hexane: ethyl acetate. The product was obtained as a brown oil, yield 13%, 24 mg (HPLC: 67%); IR (NaCl, film) ν_max_: 1747 (C=O, β-lactam), 1620 (C=C), 1256 (epoxy C-O), 917 (epoxy C-O), 747 (epoxy C-O) cm^−1^. HRMS (*m/z*) calculated for C_21_H_23_NO_5_Na [M+Na]^+^: 392.1474, found 392.1488.

### 3.15. General Method VIII: Maleic Anhydride and N-Phenylmaleimide adducts (***21a***-***d***)

A solution of the appropriate β-lactam (1.10 mmol) and maleic anhydride (1.11 mmol) or N-phenylmaleimide (1.12 mmol) in toluene (4 mL) was refluxed for 1 h. The solvent was removed under reduced pressure, and the crude product was purified by recrystallization from dichloromethane.

4-(2-(4-Chlorophenyl)-1-(3,4,5-trimethoxyphenyl)-4-oxoazetidin-3-yl)-3a,4,7,7a-tetrahydro-2-phenyl-2*H*-isoindole-1,3-dione (**21a**)

Preparation was as described in the general method VIII above from *(E)*-3-(buta-1,3-dienyl)-4-(4-chlorophenyl)-1-(3,4,5-trimethoxyphenyl)azetidin-2-one **11b** and N-phenylmaleimide. The product was obtained as a colourless solid (yield 70%); mp: 208–210 °C (HPLC: 84.5%). IR (KBr) ν_max_: 2950 (C-H), 1744 (C=O, β-lactam), 1708 (C=O), 1595 (C=C), 1503 (C=C) 1235 (C-O) cm^−1^. ^1^H NMR (400 MHz, CDCl_3_): δ 2.27–2.34 (m, 1H), 2.88–2.96 (m, 2H), 3.36–3.40 (m, 1H), 3.73 (s, 6H), 3.78 (s, 3H), 3.87–3.91, 4.12–4.17 (m, 2H), 4.72 (d, *J* = 2.48 Hz, 1H), 5.82–5.84, 6.12–6.16 (m, 2H), 6.53 (s, 2H), 7.18–7.46 (m, 9H). ^13^C NMR (100 MHz, CDCl_3_): δ 24.61, 36.65, 39.47, 41.60, 55.56, 59.34, 60.49, 60.52, 94.17, 125.95, 127.14, 128.23, 128.61, 129.12, 129.40, 129.50, 130.90, 131.18, 132.97, 134.21, 135.56, 153.11, 165.71, 176.16, 178.01. HRMS (*m/z*) calculated for C_32_H_29_^35^ClN_2_O_6_Na [M+Na]^+^: 595.1612, found 595.1602.

4-(2-(4-Bromophenyl)-1-(3,4,5-trimethoxyphenyl)-4-oxoazetidin-3-yl)-3a,4,7,7a-tetrahydroisobenzofuran-1,3-dione (**21b**)

Preparation was as described in the general method VIII above from *(E)*-3-(buta-1,3-dienyl)-(4-(4-bromophenyl)-1-(3,4,5-trimethoxyphenyl)azetidin-2-one **11c** and maleic anhydride. The product was isolated as a colourless solid (yield 24%); mp: 242–244 °C (HPLC: 97.9%). IR (KBr) ν_max_: 2950 (C-H), 1776 (C=O, β-lactam), 1746 (C=O), 1592 (C=C), 1507 (C=C), 1239 (C-O) cm^−1^. ^1^H NMR (400 MHz, CDCl_3_): δ 2.26–2.32 (m, 1H), 2.81–2.90 (m, 2H), 3.50–3.55 (m, 1H), 3.73 (s, 6H), 3.78 (s, 3H), 3.92–3.95, 3.98–4.02 (m, 2H), 4.72 (d, *J* = 2.00 Hz, 1H), 5.85–5.88, 6.14–6.18 (m, 2H), 6.51 (s, 2H), 7.34 (d, *J* = 8.56 Hz, 2H), 7.56 (d, *J* = 8.52 Hz, 2h). ^13^C NMR (100 MHz, CDCl_3_): δ 24.24, 35.84, 39.99, 42.53, 55.59, 58.75, 60.29, 60.52, 94.20, 122.53, 127.41, 129.67, 129.72, 130.78, 132.20, 133.60, 133.80, 152.99, 164.11, 171.11, 173.76. HRMS (*m/z*) calculated for C_26_H_25_^80^BrNO_7_ [M+H]^+^: 542.0814, found 542.0811.

4-(2-(4-Butoxyphenyl)-4-oxo-1-(3,4,5-trimethoxyphenyl)azetidin-3-yl)-2-phenyl-3a,4,7,7a-tetrahydro-1*H*-isoindole-1,3(2*H*)-dione (**21c**)

Preparation was as described in the general method VIII above from *(E)*-3-(buta-1,3-dienyl)4-(4-butoxyphenyl)-1-(3,4,5-trimethoxyphenyl) azetidin-2-one **11j** and N-phenylmaleimide. The product was obtained as colourless solid (yield 45%); mp: 163–164 °C (HPLC: 76.1%). IR (KBr) ν_max_: 2958 (C-H), 1745 (C=O, β-lactam), 1710 (C=O), 1591 (C=C), 1506 (C=C), 1245 (C-O) cm^−1^. ^1^H NMR (400 MHz, CDCl_3_): δ 0.98 (t, *J* = 7.54 Hz, 3H), 1.47–1.53 (m, 2H), 1.75–1.79 (m, 2H), 2.30–2.32 (m, 1H), 2.87–2.94 (m, 2H), 3.35–3.39 (m, 1H), 3.72 (s, 6H), 3.78 (s, 3H), 3.89–3.93, 4.16–4.20 (m, 2H), 3.96 (t, *J* = 6.52 Hz, 2H), 4.67 (d, *J* = 2.52 Hz, 1H), 5.83–5.86, 6.10–6.14 (m, 2H), 6.58 (s, 2H), 7.18–7.45 (m, 9H). ^13^C NMR (100 MHz, CDCl_3_): δ 13.39, 18.77, 24.59, 30.78, 36.76, 39.51, 41.52, 55.50, 59.10, 60.50, 61.03, 67.33, 94.20, 114.75, 125.98, 127.05, 128.16, 128.50, 128.57, 129.02, 129.95, 131.25, 133.36, 133.87, 153.00, 159.06, 166.19, 176.15, 178.11. HRMS (*m/z*) calculated for C_36_H_38_N_2_O_7_Na [M+Na]^+^: 633.2577, found 633.2571.

3a,4,7,7a-Tetrahydro-4-(2-(3-hydroxy-4-methoxyphenyl)-1-(3,4,5-trimethoxyphenyl)-4-oxoazetidin-3-yl)isobenzofuran-1,3-dione (**21d**)

Preparation was as described in the general method VIII above from *(E)*-3-(buta-1,3-dienyl)-4-(3-hydroxy-4-methoxyphenyl)-1-(3,4,5-trimethoxyphenyl)azetidin-2-one **11p** and maleic anhydride. The product was obtained as a colourless solid (yield 41%); mp: 224–225 °C (HPLC: 82.9%). IR (KBr) ν_max_: 3300 (OH), 2932 (C-H), 1778 (C=O, β-lactam), 1747 (C=O), 1591 (C=C), 1508 (C=C) cm^−1^. ^1^H NMR (400 MHz, CDCl_3_): δ 2.26–2.33 (m, 1H), 2.80–2.86 (m, 2H), 3.51 (t, *J* = 8.52 Hz, 1H), 3.71 (s, 6H), 3.77 (s, 3H), 3.83 (s, 3H), 3.92–3.96 (m, 1H), 4.01–4.05 (m, 1H), 4.64 (d, *J* = 2.00 Hz, 1H), 5.35 (bs, 1H), 5.87–5.89, 6.12–6.16 (m, 2H), 6.55 (s, 2H), 6.88–7.02 (m, 3H). ^13^C NMR (100 MHz, CDCl_3_): δ 24.23, 35.96, 40.03, 42.43, 55.10, 55.49, 60.50, 58.54, 60.75, 94.29, 112.15, 118.34, 119.09, 128.72, 129.31, 130.21, 133.03, 134.03, 145.28, 151.02, 153.03, 165.42, 170.90, 173.31. HRMS (*m/z*) calculated for C_27_H_26_NO_9_ [M-H]^-^: 508.1608, found 508.1589.

### 3.16. General Method IX: Adducts with Dimethylacetylene Dicarboxylate (***22a***-***d***)

Equivalent amounts of β-lactam and dimethylacetylene dicarboxylate (DMAD) were refluxed in dry toluene (5 mL) for 6–7 h under N_2_. The reaction mixture was then purified by flash chromatography over silica gel (eluent, 4:1 *n*-hexane: ethyl acetate).

Dimethyl-6-(1-(3,4,5-trimethoxyphenyl)-2-(naphthalen-1-yl)-4-oxoazetidin-3-yl)cyclohexa-1,3-diene-1,2-dicarboxylate (**22a**)

Preparation was as described in the general method IX above from *(E)*-3-(buta-1,3-dienyl)-4-(naphthalen-1-yl)-1-(3,4,5-trimethoxyphenyl)azetidin-2-one **11m.** The product was obtained as a yellow solid (yield 14%); mp: 120–122 °C (HPLC: 94.2%). IR (KBr) ν_max_: 2954 (C-H), 1750 (C=O, β-lactam), 1726 (C=O), 1587 (C=C), 1507 (C=C), 1267 (C-O) cm^−1^. ^1^H NMR (400 MHz, CDCl_3_): δ 3.01–3.09 (m, 1H), 3.25–3.33 (m, 1H), 3.50–3.51 (m, 1H), 3.57 (s, 6H), 3.61 (s, 3H), 3.76 (s, 3H), 3.81 (s, 3H), 4.01–4.03 (m, 1H), 5.71 (br s, 1H), 5.91–5.93 (m, 2H), 6.52 (s, 2H), 7.45–8.10 (m, 7H). ^13^C NMR (100 MHz, CDCl_3_): δ 27.79, 35.85, 52.00, 55.50, 59.96, 60.46, 62.95, 94.28, 121.56, 123.03, 123.25, 125.25, 125.65, 126.19, 128.44, 128.90, 130.12, 132.21, 133.18, 133.41, 133.46, 133.87, 134.41, 153.01, 164.46, 167.26, 167.48. HRMS (*m/z*) calculated for C_32_H_31_NO_8_Na [M+Na]^+^: 580.1947, found 580.1940.

Dimethyl-6-(1-(3,4,5-trimethoxyphenyl)-2-(naphthalen-1-yl)-4-oxoazetidin-3-yl)cyclohexa-1,3-diene-1,2-dicarboxylate (**22b**)

Preparation was as described in the general method IX above from *(E)*-3-(buta-1,3-dienyl)-4-(naphthalen-1-yl)-1-(3,4,5-trimethoxyphenyl)azetidin-2-one **11m**. The product was obtained as a yellow oil (yield 8%) (HPLC: 96.4%). IR (KBr) ν_max_: 2954 (C-H), 1746 (C=O, β-lactam), 1727 (C=O), 1590 (C=C), 1507 (C=C) cm^−1^. ^1^H NMR (400 MHz, CDCl_3_): δ 3.13–3.14 (m, 1H), 3.18 (s, 3H), 3.43–3.51 (m, 1H), 3.60 (s, 7H), 3.75 (s, 6H), 4.00 (br s, 1H), 5.50 (br s, 1H), 5.92–5.95 (m, 1H), 6.12–6.15 (m, 1H), 6.50 (s, 2H), 7.41–8.01 (m, 7H). ^13^C NMR (100 MHz, CDCl_3_): δ 27.64, 35.53, 51.27, 51.87, 55.49, 59.95, 60.45, 62.07, 94.25, 121.54, 122.33, 125.04, 125.54, 125.70, 126.16, 128.44, 128.57, 130.14, 131.96, 132.96, 133.29, 133.44, 133.72, 133.99, 153.04, 164.61, 166.62, 167.12. HRMS (*m/z*) calculated for C_32_H_31_NO_8_Na [M+Na]^+^: 580.1947, found 580.1950.

Dimethyl-6-(1-(3,4,5-trimethoxyphenyl)-2-(naphthalen-2-yl)-4-oxoazetidin-3-yl)cyclohexa-1,3-diene-1,2-dicarboxylate (**22c**)

Preparation was as described in the general method IX above from *(E)*-3-(buta-1,3-dienyl)-4-(naphthalen-2-yl)-1-(3,4,5-trimethoxyphenyl)azetidin-2-one **11n**. The product was obtained as a yellow solid (yield 13%); mp: 146–147 °C (HPLC: 98.9%). IR (KBr) ν_max_: 2958 (C-H), 1748 (C=O, β-lactam), 1726 (C=O), 1594 (C=C), 1506 (C=C), 1287 (C-O) cm^−1^. ^1^H NMR (400 MHz, CDCl_3_): δ 3.01–3.07 (m, 1H), 3.22–3.29 (m, 1H), 3.42–3.44 (m, 1H), 3.66 (s, 9H), 3.75 (s, 3H), 3.79 (s, 3H), 3.93–3.94 (m, 1H), 5.02 (d, *J* = 2.00 Hz, 1H), 5.89–5.91 (m, 1H), 5.95–5.98 (m, 1H), 6.56 (s, 2H), 7.40–7.88 (m, 7H). ^13^C NMR (100 MHz, CDCl_3_): δ 27.52, 36.00, 51.97, 52.05, 55.51, 58.18, 60.45, 63.10, 94.22, 122.76, 123.60, 124.95, 125.24, 126.09, 126.28, 127.35, 127.40, 128.88, 132.80, 132.91, 133.22, 133.85, 133.91, 134.17, 134.53, 152.98, 164.24, 167.12, 167.72. HRMS (*m/z*) calculated for C_32_H_31_NO_8_Na [M+Na]^+^: 580.1947, found 580.1948.

Dimethyl-6-(1-(3,4,5-trimethoxy-phenyl)-2-(naphthalen-2-yl)-4-oxo-azetidin-3-yl)-cyclohexa-1,3-diene-1,2-dicarboxylate (**22d**)

Preparation was as described in the general method IX above from *(E)*-3-(buta-1,3-dienyl)-4-(naphthalen-2-yl)-1-(3,4,5-trimethoxyphenyl)azetidin-2-one **11n.** The product was isolated as a yellow solid (yield 10%); mp: 144–146 °C. (HPLC: 98.2%). IR (KBr) ν_max_: 2955 (C-H), 1746 (C=O, β-lactam), 1727 (C=O), 1591 (C=C), 1507 (C=C), 1278 (C-O) cm^−1^. ^1^H NMR (400 MHz, CDCl_3_): δ 3.12–3.19 (m, 1H), 3.33 (s, 3H), 3.35–3.42 (m, 1H), 3.58–3.60 (m, 1H), 3.68 (s, 6H), 3.77 (s, 3H), 3.86 (s, 3H), 3.95–3.96 (m, 1H), 4.79 (d, *J* = 2.00 Hz, 1H), 5.91–5.93 (m, 1H), 6.06–6.09 (m, 1H), 6.58 (s, 2H), 7.36–7.83 (m, 7H). ^13^C NMR (100 MHz, CDCl_3_): δ 28.13, 34.70, 51.53, 52.07, 55.59, 56.62, 60.48, 62.12, 94.19, 122.60, 122.68, 124.97, 125.49, 125.88, 126.09, 127.33, 127.43, 128.58, 131.39, 132.74, 132.85, 133.20, 134.03, 134.18, 135.81, 153.08, 164.29, 166.03, 168.01. HRMS (*m/z*) calculated for C_32_H_31_NO_8_Na [M+Na]^+^: 580.1947, found 580.1951.

### 3.17. Biochemical Evaluation

#### 3.17.1. Cell Culture

All biochemical assays were performed in triplicate for the determination of mean values reported. The human breast carcinoma cell line MCF-7 was purchased from the European Collection of Animal Cell Cultures (ECACC) and was cultured in Eagle’s minimum essential medium with 10% fetal bovine serum, 2 mM L-glutamine and 100 μg/mL penicillin/streptomycin. The medium was supplemented with 1% non-essential amino acids. The human breast carcinoma cell line MDA-MB-231 was purchased from the ECACC. MDA-MB-231 cells were maintained in Dulbecco’s modified Eagle’s medium (DMEM) supplemented with 10% (*v*/*v*) fetal bovine serum, 2 mM L-glutamine and 100 μg/mL penicillin/streptomycin (complete medium). HEK-293T normal epithelial embryonic kidney cells were cultured in DMEM with GlutaMAX^TM^-I in the absence of non-essential amino acids. HL-60 cells were originally obtained from the ECACC. SW-480 cells were a kind gift from Dr. Emma Creagh, School of Biochemistry and Immunology, Trinity College Dublin. SW-480 cells were cultured in DMEM with GlutaMAX-I, with the same supplement in the absence of non-essential amino acids. HL-60 cells were cultured in RPMI-1640 Glutamax 1 medium supplemented with 10% FBS media, and 100 μg/mL penicillin/streptomycin. HT-29 cells originate from a human adenocarcinoma of the colon, were originally obtained from the ECACC and were grown in DMEM Glutamax media. HT-29 media were supplemented with 10% foetal bovine serum (FBS). Cells were maintained at 37 °C in 5% CO_2_ in a humidified incubator. All cells were sub-cultured 3 times/week by trypsinisation.

#### 3.17.2. Cell Viability Assay

Cells were seeded at a density of 5 × 10^3^ cells/well (MCF-7) in triplicate in 96-well plates. After 24 h, cells were then treated with medium alone, with vehicle (1% ethanol (*v*/*v*)) or with selected dilutions of control CA-4 or the synthesised azetidinone compounds in the concentration range 1–50 μM. Cell proliferation for MCF-7 and MDA-MB-231 cells was analysed using the Alamar Blue assay (Invitrogen Corp.). After 72 h, Alamar Blue (10% (*v*/*v*)) was added to the contents of each well, and plates were then incubated in the dark for 3–5 h at 37 °C. Fluorescence results were obtained with a 96-well fluorimeter operating with excitation (530 nm) and emission (590 nm), and the results were expressed as viability (%) relative to vehicle control (100%). IC_50_ values (concentration of drug resulting in 50% reduction in cell survival) were obtained from the dose response curves using Prism (GraphPad Software, Inc., La Jolla, CA, USA). Experiments were performed in triplicate on at least three separate occasions.

#### 3.17.3. Lactate Dehydrogenase Assay for Cytotoxicity

The cytotoxic effects of the compounds were determined using the CytoTox 96 non-radioactive cytotoxicity assay (Promega) [107] as previously reported [61]. The MCF-7 cells were seeded in 96-well plates and incubated for 24 h. Cells were treated with test compounds **16**, **26** and **30** at 10 μM concentration as described in the cell viability assay above. At 72 hr, 20 μL of ‘lysis solution (10X)’ was added to the control wells to ensure 100% death. The cells were incubated for a further 1 hr. Supernatant (50 μL) was removed from each well and transferred to a 96-well plate. Cytotox 96R Reagent (50 μL) was added to each well, and the plate was retained in darkness at 20 °C for 30 min. 50 μL of ‘stop solution’ was then added to each well, and the absorbance was determined at 490 nm using a Dynatech MR5000 plate reader. The cell death (%) at 10 μM was determined.

#### 3.17.4. Cell Cycle Analysis

MCF-7 cells were treated with compound **9q** (at concentration of 50 nM and 500 nM) and incubated for 72 h. Cells (adherent and detached) were collected, trypsinised and centrifuged (800× *g*, 15 min). Cells were washed with ice-cold PBS (x2) and fixed in ice-cold 70% ethanol overnight at −20 °C. Fixed cells were centrifuged (800× *g*, 15 min) and stained with PI (50 μg/mL) containing DNase-free RNase A (50 μg/mL) at 37 °C for 30 min. The DNA content of the cells (10,000 cells/experimental group) was recorded by flow cytometry at 488 nm using a FACSCalibur flow cytometer (BD Biosciences, San Jose, CA, USA) and analysed using cellQuest software (BD Biosciences, San Jose, CA, USA).

#### 3.17.5. Annexin-V/PI Apoptosis Assay

Flow cytometry using annexin-V and propidium iodide (PI) was used to detect apoptotic cell death in MCF-7 cells. Cells were seeded in 6-well plates (density 1 × 10^5^ cells/mL) and treated with either vehicle (0.1% (*v*/*v*) EtOH), CA-4 or β-lactam compound **9q** at concentrations of 50 nM and 500 nM for 72 h. Cells were then harvested and washed in 1X binding buffer (20X binding buffer: 0.1M HEPES, pH 7.4; 1.4 M NaCl; 25 mM CaCl_2_ diluted in dH_2_O). Cells were then incubated on ice in darkness for 30 min in annexin-V-containing binding buffer [1:100]. Cells were washed once in binding buffer and then re-suspended in propidium iodide containing binding buffer [1:1000]. Cells were analysed directly using the BD Accuri flow cytometer and Prism software. Four cell populations are produced in this assay: annexin-V- and PI-negative (Q4, healthy cells), annexin-V-positive and PI-negative (Q3, early apoptosis), annexin-V- and PI-positive (Q2, late apoptosis) and annexin-V-negative and PI-positive (Q1, necrosis). CA-4 (50 nM) was used as the positive control for apoptosis.

#### 3.17.6. Tubulin Polymerization Assay

Tubulin polymerization was monitored using the BK006 kit obtained from Cytoskeleton Inc. (Denver, CO, USA). [98] The values reported represent the average values determined from two independent assays. Purified bovine brain tubulin (>99%, 3 mg/mL) in buffer containing PIPES (80 mM, pH 6.9), EGTA (0.5 mM), MgCl_2_ (2 mM), GTP (1 mM) and glycerol (10%) was incubated at 37 °C in vehicle (1% DMSO or EtOH (*v*/*v*) in dH_2_O) or with compounds **9h, 9q, 9s, 10h, 10p, 10r, 11h, 11p, 11r, 17f** (all at 10 µM concentration); CA-4 (10 μM) was used as control. In this assay, light scattering is related to the concentration of polymerised microtubules produced. Tubulin assembly was monitored turbidimetrically at 340 nm at 37 °C in a Spectramax 340 PC spectrophotometer (Molecular Devices, Sunnyvale, CA, USA). The absorbance was measured at 30 s intervals over 30–60 min, and the V_max_ and fold reduction in V_max_ were determined.

#### 3.17.7. Colchicine-Binding Site Assay

MCF-7 cells were seeded at density of 5 × 10^4^ cells/well in 6-well plates and incubated overnight. Cells were treated with vehicle control (ethanol (0.1% *v*/*v*)) or compound **9q** (10 μM) for 2 h, followed by treatment with *N,N’*-ethylene-bis(iodoacetamide)(EBI) (100 μM solution in ethanol) (Santa Cruz Biotechnology, Dallas, TX, USA), for 1.5 h. Cells were then washed with ice-cold PBS (x2) and lysed by addition of Laemmli buffer. Samples were separated by SDS-PAGE, transferred to polyvinylidene difluoride membranes and examined with β-tubulin antibodies (Sigma-Aldrich, Milwaulkee, WI, USA) [99].

#### 3.17.8. Immunofluorescence Microscopy

The effects of β-lactam **9q** on MCF-7 cytoskeleton were determined using confocal microscopy as previously described. [80] MCF-7 cells were seeded at 1 × 10^5^ cells/mL in glass slides with eight chambers (BD Biosciences, San Jose, CA, USA). Cells were treated with vehicle (1% ethanol (*v*/*v*)), paclitaxel (1 μM), combretastatin A-4 (50 nM) or **9q** (10 nM, 100 nM and 500 nM) for 16 h. Following treatment, cells were gently washed in PBS, fixed for 20 min with 4% paraformaldehyde in PBS and permeabilised in 0.5% Triton X-100. The cells were then washed with PBS-T (PBS containing 0.1% Tween), blocked in 5% bovine serum albumin diluted in PBS-T and incubated with mouse monoclonal anti-α-tubulin-FITC antibody (clone DM1A) (Sigma, Milwaulkee, WI, USA) (1:100) for 2 h at 20 °C. The cells were washed again with PBS-T and incubated with Alexa Fluor 488 dye (1:450) for 1 h at 20 °C. Following washes in PBST, the cells were mounted in Ultra Cruz Mounting Media (Santa Cruz Biotechnology, Santa Cruz, CA, USA), which contained 4,6-diamino-2-phenolindol dihydrochloride (DAPI). The immunofluorescence cell images were captured by Leica SP8 confocal microscopy and analysed with Leica application suite X software. Microscopy experiments were performed on three independent occasions, and the images for these experiments were obtained on the same day using identical conditions.

#### 3.17.9. Stability Study for Compounds **9q**, **15a**, **10h**, **10q**, **10p**, **10r** and CA-4

Analytical high-performance liquid chromatography (HPLC) stability studies were performed as follows using Symmetry^®^ column (C_18_, 5 µm, 4.6 × 150 mm), Waters 2487 Dual Wavelength Absorbance detector, Waters 1525 binary HPLC pump and Waters 717 plus Autosampler (Waters Corporation, Milford, MA, USA) with detection at 254 nm as previously described [61]. Samples were analysed using mobile phase acetonitrile (80%): water (20%), flow rate (1 mL/min) over 10 min. Stock solutions of compounds **9q, 15a, 10h, 10q, 10p, 10r** and CA-4 (5 mg) in mobile phase (10 mL) were prepared (with and without the addition of 0.5% Tween) to aid solubility. (i) *Stability in phosphate buffers:* Phosphate buffers at the desired pH values (4, 7.4 and 9) were prepared as described in the European Pharmacopoeia (Ph. Eur., 11th edition, 2022). First, 30 µL of compound stock solution was diluted with appropriate buffer (1 mL); sample was shaken and injected immediately. Samples were withdrawn and analysed at the following time points: *t* = 0 min, 5 min, 30 min, 60 min, 90 min, 120 min, 24 h and 48 h. (ii) *Thermal stability:* Compound stock solution (1 mL) was placed in a glass vial on a heating block for 4 h at 60 °C. The sample was then cooled, diluted (acetonitrile) and analysed. (ii) *Oxidising conditions:* H_2_O_2_ (3%, 0.2 mL) was added to compound stock solution (0.8 mL). The vial was vortexed and retained at 20 °C, and the sample was analysed at 60, 120 180 and 240 min. (iv) A*cidic conditions:* HCl (0.1 M, 0.2 mL) was added to stock solutions (0.8 mL). The vial was mixed by vortex and retained at 20 °C. A sample was neutralised with NaOH (0.1 M, 0.2 mL) at 60, 120, 180 and 240 min, followed by HPLC analysis. (v) *Alkaline conditions*: NaOH (0.1 M, 0.2 mL) was added to stock solution (0.8 mL) of the compound in a vial. The vial was mixed by vortex and retained at 20 °C. A sample was neutralised with HCl (0.1 M, 0.2 mL) at 60, 120, 180 and 240 min followed by HPLC analysis. (vi) *Photostability study*: Compounds (stock solution, 1 mL) were placed in a vial, exposed to UV light for 4 h and then analysed by HPLC as above. *Stability studies in plasma:* Approval for this study was obtained from the School of Pharmacy and Pharmaceutical Sciences Trinity College Dublin Research Ethics Committee (2020–06-01-MS). Following informed consent, blood was withdrawn from healthy volunteers, and plasma was obtained by centrifugation and kept at −20 °C until further use. Then, 360 µL stock solution of compounds **9q, 15a, 10h, 10q, 10p, 10r** or CA-4 was added to buffered plasma (plasma: buffer = 1:9, 4 mL total volume) at 37 °C in HPLC vial. A sample (250 µL) was then added to the Eppendorf tube containing ZnSO_4_.7H_2_O solution (500 µL) (2% *w*/*v* ZnSO_4_ solution in acetonitrile:water, 1:1). The samples were centrifuged (10,000 rpm, 3 min), filtered (0.2 micron filter) and analysed by HPLC as above. Further samples were taken at one-hour intervals.

### 3.18. X-ray Crystallography

Data for **8h** and **8i** were measured on a Bruker APEX DUO, and data for **10h**-**11t** were measured on a Bruker D8 Quest ECO, using Mo Kα radiation (λ = 0.71073 Å). Each sample was mounted on a MiTeGen cryoloop, and data were collected at 100(2) K using an Oxford Cryosystems Cobra (**8h**, **8i**) or Cryostream (**10h**-**11t**) low-temperature device. Bruker APEX [108,109] software was used to collect and reduce data. Absorption corrections were applied using SADABS [110]. Structures were solved with the SHELXT structure solution program [111] using intrinsic phasing. All were refined using least-squares method on F^2^ with SHELXL [112]. All non-hydrogen atoms were refined anisotropically. Hydrogen atoms were assigned to calculated positions using a riding model with appropriately fixed isotropic thermal parameters. Molecular graphics were generated using OLEX2 [113]. Crystal data and details of data collection and refinement are provided in Table S1, Supplementary Materials. In **10h**, there are two independent molecules in the asymmetric unit. Part of one beta-lactam ring (C32/C32b) and the vinylic substituent are disordered and modelled in two positions with 49:51% occupancy with displacement restraints (SIMU). In **11t**, the donor hydrogens were located and refined, and in **11h**, the sample was weakly diffracting, with weak high-angle data resulting in a high R(int). Crystallographic data for the structures in this paper have been deposited with the Cambridge Crystallographic Data Centre as supplementary publications No. 1820359 [68], 2241430, 2241431, 2241432, 2241433, 2241434, 2241435, 2241436, 2241437, 2241438. Copies of the data can be obtained, free of charge, upon application to CCDC, 12 Union Road, Cambridge CB2 1EZ, UK (fax, +44-(0)1223–336033, or e-mail: deposit@ccdc.cam.ac.uk).

### 3.19. Computational Procedure: Molecular Docking Study

#### 3.19.1. Computational Procedure for Molecular Docking

The 1SA0 X-ray structure of bovine tubulin co-crystallised with N-deacetyl-N-(2-mercaptoacetyl)-colchicine (DAMA-colchicine) was downloaded from the PDB website [18]. A UniProt Align analysis confirmed a 100% sequence identity between human and bovine β tubulin. The crystal structure was prepared using QuickPrep (minimised to a gradient of 0.001 kcal/mol/Å), Protonate 3D, Residue pKa and Partial Charges protocols in MOE 2022 with the MMFF94x force field. Each compound was drawn in MOE, saved as an mdb and processed in MOE [104]. 3*S,*4*R trans* enantiomers of the compounds were examined. For each compound, MMFF94x partial charges were calculated, and each was energy-minimised to a gradient of 0.001 kcal/mol/Å. Default parameters were used for docking, except that 300 poses were sampled for each compound, and the top 50 docked poses generated for each compound were retained for subsequent analysis.

#### 3.19.2. Mapping of Protein-Binding Sites (MoPBS) Algorithm

The 1SA0 X-ray structure of bovine tubulin co-crystallised with N-deacetyl-N-(2-mercaptoacetyl)colchicine (DAMA-colchicine) [18] was used after the processing above was completed. As described above and in our recent paper [106], the binding site was flooded with 100 copies of each fragment—in this case, a more focused selection of four fragments (acetate ion, benzene, methane, methylammonium) compared to the nine fragments used in the original report. The DBSCAN K-means algorithm was used to cluster eight pharmacophore features from the 400 minimised fragments.

## 4. Conclusions

Microtubule-targeting agents (MTA) such as paclitaxel and docetaxel and the vinca alkaloids vincristine, vinblastine and vinorelbine are widely used in chemotherapy for a variety of different cancers. Extensive preclinical studies have shown that CBSIs are promising drug candidates for cancer therapy. CA-4-based codrugs (or mutual prodrugs) have recently been demonstrated to improve the therapeutic profile of clinically used drugs such as doxorubicin, floxuridine and tegafur, and they have achieved targeted delivery of drugs to cancer tissues [114]. Colchicine-binding-site-targeting compounds are of interest not only for their potent antimitotic effects [11,115,116,117]; in a recent study, the potent antimitotic sabizabulin **5** was evaluated in a clinical trial in metastatic breast cancer and also as an antiviral agent for the treatment of hospitalised COVID-19 patients at high risk for acute respiratory distress syndrome [118]. Sabizabulin binds to viral tubulin at the colchicine-binding site and disrupts the intracellular transport of the virus. Therefore, investigation of the non-covalent tubulin-targeting b-lactam compounds developed in the present work as potential antibacterial agents is also of current interest.

Increasing evidence is also available in relation to the non-mitotic effects of tubulin-targeting compounds, e.g., as vascular disrupting agents (VDA) and inhibitors of tumour angiogenesis [119]. Many small-molecule dual-targeting tubulin inhibitors have been reported with applications for cancer therapy [120]; e.g., novel CA-4 sulphamate derivatives identified for dual tubulin polymerization and arylsulphatase inhibitors having potential for applications in cancer therapy [121]. Dual CBSIs and Src kinase inhibitors are reported to be effective in regulating the overexpression of tubulin isotypes and can inhibit drug resistance, which is mediated by P-glycoprotein (P-gp), and other multidrug-resistance-associated proteins (MRP1, MRP2) [122]. Interestingly, it is suggested that microtubules may modulate immune responses, and therefore, the use of microtubule inhibitors together with immune therapy may offer a highly effective option for selected cancer treatments [123].

In this study, a series of ninety-six compounds based on the 2-azetidinone scaffold structure were designed and synthesised as CBSIs. These novel 3-(prop-1-en-2-yl)azetidin-2-one, 3-allylazetidin-2-one and 3-(buta-1,3-dien-1-yl)azetidin-2-one analogues of CA-4 were prepared using an efficient Staudinger procedure, and they allowed introduction of the alkene, allyl and diene substituents at the C-3 position of the β-lactam, together with structurally varied electron-releasing or electron-withdrawing substituent groups on the ring B pharmacophore. These compounds, together with some amino acid and phosphate prodrugs and their Diels–Alder adducts, were evaluated for their antiproliferative activity, cell cycle effects and inhibition of tubulin assembly.

The compounds were shown to have significant in vitro antiproliferative activities in MCF-7 breast cancer cells, particularly compounds **9h, 9q, 9r, 10p 10r, 11h**, with IC_50_ values in the range 10–33 nM. The phenolic 3-(prop-1-en-2-yl) β-lactam compound **9q** was identified as the most potent in MCF-7 breast cancer cells (IC_50_ = 10 nM, with drug-like properties. Significant antiproliferative effects were also demonstrated for the compounds in the TNBC cell line MDA-MB-231, with IC_50_ values in the range 23–33 μM, which were comparable with the activity of CA-4. The biological target of these compounds was identified as tubulin. They demonstrated significant reduction in tubulin polymerisation in vitro and were shown to interact non-covalently at the colchicine-binding site on tubulin. Cell cycle analysis through flow cytometry demonstrated that compound **9q** arrested MCF-7 cells in the G2/M phase, resulting in cellular apoptosis.

Microtubule depolymerisation was confirmed by confocal microscopy, and the immunofluorescence results confirm the antimitotic properties of β-lactam **9q**, which was observed to target tubulin and result in mitotic catastrophe. In addition, in silico molecular docking results indicate that the β-lactam compounds can interact with the colchicine-binding site of tubulin, further supporting the observed inhibitory effects of these compounds on tubulin polymerisation. Tubulin-targeted chemotherapy has been clinically successful against a wide spectrum of solid tumours and blood cancers. Compound **9q** is representative of a novel class of potent microtubule-destabilising agents having low toxicity with promising small-molecule, drug-like properties and translational potential for the development of new molecules with potent tubulin-inhibitory activity.

## Figures and Tables

**Table 1:** X-ray crystal structure of compounds **9h**, **10h** and **11h** ^a,b^.

**9h**	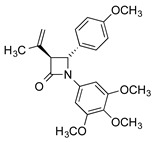	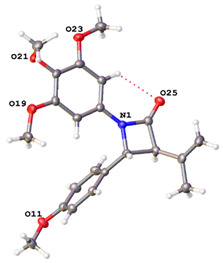
**10h**	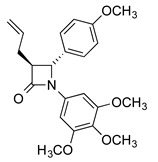	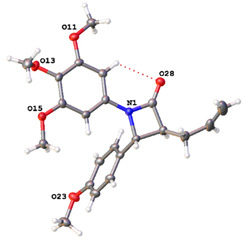
**11h**	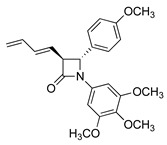	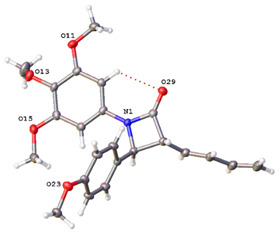

^a^ X-ray crystal structure of compounds **9h**, **10h** and **11h** with the thermal ellipsoids set at 50% probability. ^b^ Crystallographic data deposited with the Cambridge Crystallographic Data Centre (CCDC): 1820359 (**9h**), 2241433 (**10h**) and 2241437 (**11h**).

**Table 5:** Antiproliferative activities of β-lactams **17a**-**g**, **19**, **20**, **21a**-**d, 22a**-**d** in human MCF-7 breast cancer cells.

Compound Number	Antiproliferative Activity IC_50_ (µM) ^a^	cLogP ^c^
**17a**	0.332 ± 0.04	4.77
**17b**	1.090 ± 0.08	3.85
**17c**	75.4 ± 8.12	5.79
**17d**	49.5 ± 5.23	6.59
**17e**	4.757 ± 0.56	5.65
**17f**	0.035 ± 0.003	2.74
**17g**	0.075 ± 0.01	1.82
**19**	7.706 ± 0.78	5.32
**20**	51.5 ± 5.46	2.02
**21a**	106.1 ± 13.9	2.29
**21b**	96.2 ± 13.5	0.43
**21c**	38.6 ± 4.90	2.98
**21d**	91.1 ± 14.5	3.77
**22a**	141.8 ± 20.3	4.49
**22b**	9.688 ± 1.02	4.49
**22c**	15.3 ± 1.23	4.48
**22d**	5.336 ± 0.42	4.49
**CA-4 ^b^**	0.0039 ± 0.00032	3.32

^a^ IC_50_ values determined are half-maximal inhibitory concentrations required to block the growth stimulation of MCF-7 cells. Treatment at eight different concentrations (0.001–50 mM) for 72 h was used for the determination of the IC_50_ values for each compound in comparison to the control compound CA-4. Values represent the mean ± SEM (error values × 10^−6^) for at least three experiments performed in triplicate. ^b^ IC_50_ value determined for CA-4 (0.0039 μM) in MCF-7 cells is in agreement with reported values [81,82]. ^c^ clogP values were calculated from ChemBioDraw 13.0 and are documented in the table.

**Table 6:** Antiproliferative activities of selected β-lactams in MDA-MB-231 human breast cancer cells^a^.

Compound Number	Antiproliferative Activity IC_50_ (µM) ^a^	cLogP ^d^
**9g**	0.246 ± 0.017	3.82
**9h ^b^**	0.0313	3.24
**9i**	0.171 ± 0.011	3.77
**9n**	0.529 ± 0.08	4.50
**9q ^b^**	0.0239	2.32
**10g**	0.377 ± 0.06	3.75
**10h ^b^**	0.120	3.17
**10l**	0.486 ± 0.06	4.94
**10m**	0.656 ± 0.13	4.43
**10p ^b^**	0.0274	2.25
**10r ^b^**	0.0352	2.12
**11g**	0.160 ± 0.014	4.06
**11h ^b^**	0.0311	3.48
**11p ^b^**	0.113	2.56
**11r ^b^**	0.0337	2.03
**15a**	0.032 ± 0.005	1.24
**CA-4 ^c^**	0.043	3.32

^a^ IC_50_ values are half-maximal inhibitory concentrations required to block the growth stimulation of MDA-MB-231 cells. Values represent the mean ± SEM (error values × 10^−6^) for at least three experiments performed in triplicate. Treatment at eight concentrations (0.001-50 μM) over 72 h used for determination of IC_50_ values. ^b^Antiproliferative activity against MDA-MB-231 obtained from NCI. Treatment at five concentrations (0.01–100 μM) over 48 h. ^c^ The IC_50_ value determined for CA-4 is consistent with reported IC_50_ values [87,88]. ^d^ cLogP values were calculated using ChemBioDraw 13.0 and are documented in the table.

**Table 7:** Summary of NCI 60 cell line mean screening results (GI_50,_ TGI, LC_50_) for selected compounds.

NCI Reference Number	Compound Number	Mean GI_50_ (nM) ^a^	Mean TGI (µM) ^b^	Mean LC_50_ (µM) ^a^
**S-613729**	**CA-4**	99.30	10.30	85.50
**S-762037**	**9h**	58.88	15.49	87.10
**S-762032**	**9q**	39.81	32.36	81.28
**S-762042**	**9s**	54.95	22.39	75.86
**D-762038**	**10h**	75.86	17.38	81.28
**D-762033**	**10p**	61.66	35.48	93.33
**D-762043**	**10r**	85.11	53.70	79.43
**D-762039**	**11h**	58.88	8.91	77.62
**D-762034**	**11p**	288.40	31.62	91.20
**D-762044**	**11r**	91.20	38.02	77.62
**S-775044**	**15a**	138.0	43.65	85.11
**S-775045**	**15b**	524.8	44.66	93.32

^a^ GI_50_ and LC_50_ are the mean concentrations required to inhibit the growth and kill 50% of the cells in the assay (all cell lines), respectively [94]. ^b^ TGI is the mean concentration required to completely inhibit the growth of all cells [94].

**Table 8:** Inhibition of tubulin polymerisation for compounds **9h**, **9q, 9s**, **10h**, **10p, 10r**, **11h**, **11p**, **11r**, **17d**.

Compound	V_max_ (mOD/min) ^a^	Fold Reduction in V_max_^b^
**9h**	4.635	1.81
**9q**	1.968	4.27
**9s**	3.156	2.66
**10h**	0.0056	2.5
**10p**	0.0015	4.5
**10r**	0.0029	3.4
**11h**	0.0035	2.9
**11p**	0.0091	1.1
**11r**	0.0035	2.0
**17f**	0.0038	2.2
**DMSO**	0.0140	-
**Ethanol**	0.0069	-
**CA-4**	0.0022	6.3

^a^ Effects of β-lactams **9h, 9q, 9s, 10h, 10p, 10r, 11h, 11p, 11r, 17f** and CA-4 on in vitro tubulin polymerisation. The tubulin polymerization reaction was initiated by gently warming a solution of purified bovine tubulin and GTP (mixed in a 96-well plate) from 4 °C to 37 °C. All compounds were tested at a final concentration of 10 μM. Ethanol (1% *v*/*v*) was used as a vehicle control. CA-4 (10 μM) was used as a reference. Tubulin polymerisation was monitored at 37 °C with a Spectramax 340PC spectrophotometer at 340 nm for 30 min at 30 s time intervals. ^b^ The inhibition (fold) of the tubulin polymerization reaction was calculated using the V_max_ value for each reaction. The results represent the mean of three separate experiments.

## Data Availability

Data sharing not applicable.

## Supplementary Materials

The following supporting information can be downloaded at https://www.mdpi.com/article/10.3390/ph16071000/s1. Table S1: Crystal data and structure refinement for **8h**, **8i**, **9h**, **10f**, **10h**, **11b**-**d**, **11h**, **11t**; Table S2: Stability study for compounds **9q**, **15a**, **10h**, **10q**, **10p**, **10r** and CA-4 at pH 4.0, pH 7.5, pH 9.0 and in plasma; Table S3: Stability study for compounds **10h**, **10q**, **10p** and **10r** (HCl, NaOH, heat (80 °C), Light, H_2_O_2_); Table S4: Tier-1 Profiling Screen of Selected β-Lactams; Table S5: ADMET and Lipinski Properties for Selected β-Lactams; Table S6: Antitumour evaluations of compound **9q** in the NCI60 cell line in vitro primary one-dose screen; Table S7: Comparative antitumour evaluations of compounds **9h**, **9q**, **9s**, **10p**, **10h**, **10r**, **15a**, **15b** in the NCI60 leukaemia, non-small-cell lung cancer, colon cancer and CNS cancer cell lines in vitro primary screen; Table S8: Comparative antitumour evaluations of compounds **9h**, **9q**, **9s**, **10p**, **10h**, **10r**, **15a**, **15b** in the NCI60 melanoma, ovarian cancer, renal cancer and breast cancer cell lines in vitro primary screen; Table S9: Standard COMPARE analysis of β-lactam **9q**; Table S10: Standard COMPARE analysis of β-lactam **9s**; Table S11: Docking scores for selected β-lactams; Table S12: X-ray data and torsional angles for compounds **9h**, **10h**, **10f**, **11a**-**c**, **11h**; Table S13. X-ray crystal structure of compounds **8h**, **8i** and **11t**; Table S14: X-ray crystal structure of compounds **10f**, **11b**, **11c**, **11d**; Figures S1–S20: ^1^H NMR and ^13^C NMR spectra; Figure S21: Stability study for compounds **9q**, **15a**, **10h**, **10q**, **10p**, **10r** and CA-4 at pH 4.0, pH 7.5, pH 9.0 and in plasma; Figure S22: Flexible alignment of **11p** and combretastatin A-4; Figure S23: Inter-atomic distances between oxygen atoms for compounds **25**, **11p** and CA-4; Figure S24: Protein ligand interactions for β-Lactam **11p** docked in the colchicine-binding site of tubulin; Figure S25: Overlay of the X-ray structure of tubulin crystallised with DAMA colchicine with β-lactams; Figure S26: β-Lactams **9s**, **10h**, **11p** and **11r** docked in the colchicine-binding site of tubulin.

## Abbreviations

ADC	Antibody–drug conjugate
BRCA	Breast cancer gene
CA-4	Combretastatin A-4
CBSI	Colchicine-binding site inhibitor
CCDC	Cambridge Crystallographic Data Centre
CDK	Cyclin-dependent kinase
DAMA	N-deacetyl-N-(2-mercaptoacetyl)-colchicine
DCC	N,N’-Dicyclohexylcarbodiimide
DCM	Dichloromethane
DCTD	Division of Cancer Treatment and Diagnosis
DEPT	Distortionless enhancement by polarization transfer
DIPEA	N,N-diisopropylethylamine
DMAP	4-Dimethylaminopyridine
DMF	Dimethylformamide
DTP	Development Therapeutics Program
DMEM	Dulbecco’s modified Eagle’s medium
EBI	N,N′-Ethylene-bis(iodoacetamide)
ECACC	European Collection of Animal Cell Cultures
EI	Electron Impact
EMA	European Medicines Agency
ER	Estrogen receptor
FDA	United States Food and Drug Administration
Fmoc	Fluorenylmethyloxycarbonyl
GI_50_	50% growth inhibition
HER2	Human epidermal growth factor receptor 2
HRMS	High-resolution mass spectrometry
IC	Inhibitory concentration
IR	Infrared
LC_50_	50% lethal concentration
LDH	Lactate dehydrogenase
MBC	Metastatic breast cancer
*m*-CPBA	*meta*-Chloroperoxybenzoic acid
MDR	Multidrug resistance
MRP1, MRP2	Multidrug-resistance-associated proteins
MTD	Maximum tolerated dose
MTA	Microtubule-targeting agent
MS	Mass spectrometry
NCI	National Cancer Institute
NIH	National Institute of Health
NMR	Nuclear magnetic resonance
PAINS	Pan-assay interference compounds
PARP	Poly(adenosine diphosphate–ribose) polymerase
PBS	Phosphate buffer saline
*P*-*gp*	*P*-*glycoprotein*
PI	Propidium iodide
PI3K	Phosphoinositide 3-kinase
PR	Progesterone receptor
SAR	Structure–activity relationship
SERD	Selective estrogen receptor degrader
TBA	Tubulin-binding agent
TBAF	Tetrabutylammonium fluoride
TBDMS	*tert*-Butyldimethylchlorosilane
TEA	Triethylamine
TGI	Total growth inhibition
TLC	Thin-layer chromatography
TMS	Tetramethylsilane
TMCS	Trimethylchlorosilane
UV	Ultraviolet
VDA	Vascular disrupting agent
