# Correction: Wang et al. Antiproliferative and Tubulin-Destabilising Effects of 3-(Prop-1-en-2-yl)azetidin-2-Ones and Related Compounds in MCF-7 and MDA-MB-231 Breast Cancer Cells. *Pharmaceuticals* 2023, *16*, 1000

**DOI:** 10.3390/ph18081218

**Published:** 2025-08-19

**Authors:** Shu Wang, Azizah M. Malebari, Thomas F. Greene, Shubhangi Kandwal, Darren Fayne, Seema M. Nathwani, Daniela M. Zisterer, Brendan Twamley, Niamh M. O’Boyle, Mary J. Meegan

**Affiliations:** 1 School of Pharmacy and Pharmaceutical Sciences, Trinity Biomedical Sciences Institute, Trinity College Dublin, 152-160 Pearse Street, Dublin 2, D02 R590 Dublin, Ireland; 2 Department of Pharmaceutical Chemistry, College of Pharmacy, King Abdulaziz University, Jeddah 21589, Saudi Arabia; 3 Molecular Design Group, School of Biochemistry and Immunology, Trinity Biomedical Sciences Institute, Trinity College Dublin, 152-160 Pearse Street, Dublin 2, D02 R590 Dublin, Ireland; 4 School of Biochemistry and Immunology, Trinity Biomedical Sciences Institute, Trinity College Dublin, 152-160 Pearse Street, Dublin 2, D02 R590 Dublin, Ireland; 5 School of Chemistry, Trinity College Dublin, Dublin 2, D02 PN40 Dublin, Ireland

## Error in Figure

In the original publication [1], there was a mistake in Figure 9 as published. In Figure 9, CA-4 (0.05 μM) should be corrected to CA-4 (0.01 μM). Corrected Figure 9 appears below. 

## Text Correction

A correction has been made to the last paragraph of Section 2.4.4. Tubulin Polymerisation Effects of 3-(Prop-1-en-2-yl)azetidinones, 3-Allylazetidinones and 3-butadienylazetidinones: “CA-4 (50 nM)” should be “CA-4 (10 nM)”.

A correction has been made to Section 3.17.8. Immunofluorescence Microscopy: “**9q** (50 nM, 100 nM and 500 nM)” should be “**9q** (10 nM, 100 nM and 500 nM)”.

The authors state that the scientific conclusions are unaffected. This correction was approved by the Academic Editor. The original publication has also been updated.

## Figures and Tables

**Figure 9:**
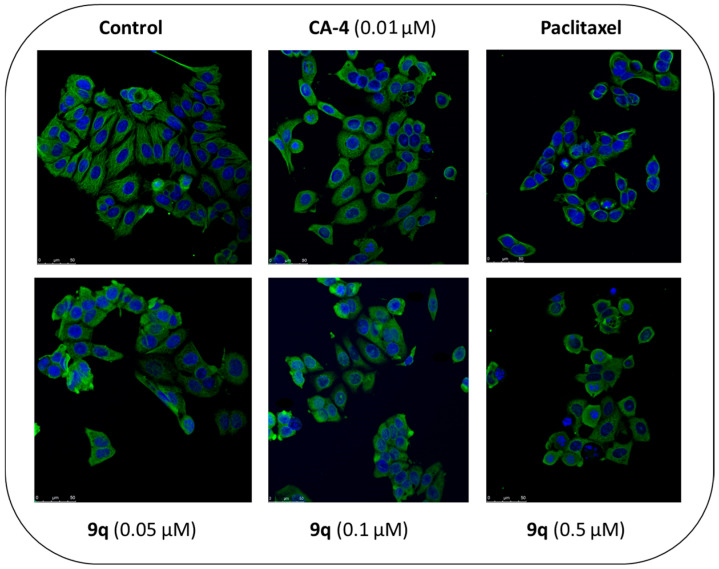
β-lactam compound **9q** induces depolymerization of the microtubule network in MCF-7 breast cancer cells.
