# Supplementary material for: Antiproliferative and Tubulin-Destabilising Effects of 3-(Prop-1-en-2-yl)azetidin-2-Ones and Related Compounds in MCF-7 and MDA-MB-231 Breast Cancer Cells
Source: Pharmaceuticals (Basel). 2023 Jul 13;16(7):1000. doi: 10.3390/ph16071000 (PMC10385824; doi:10.3390/ph16071000)
Supplement: Supplementary file 1 [file pharmaceuticals-16-01000-s001.zip › 10f and 10q.pdf]

SAMPLE INFORMATION

Sample Name:	H in 1mg/ml in MeOH(unfilter)	Acquired By:	System
Sample Type:	Unknown	Sample Set Name:	Test_Xbridgecolumn_01Jul10
Vial:	5	Acq. Method Set:	Azetidinone_ACN_H20_MS
Injection #:	1	Processing Method:	Azetidinone_PM
Injection Volume:	10.00 ul	Channel Name:	2487Channel 1
Run Time:	20.0 Minutes	Proc. Chnl. Descr.:	262nm
Date Acquired:	01/07/2010 09:51:14 IST		
Date Processed:	01/07/2010 11:00:23 IST		

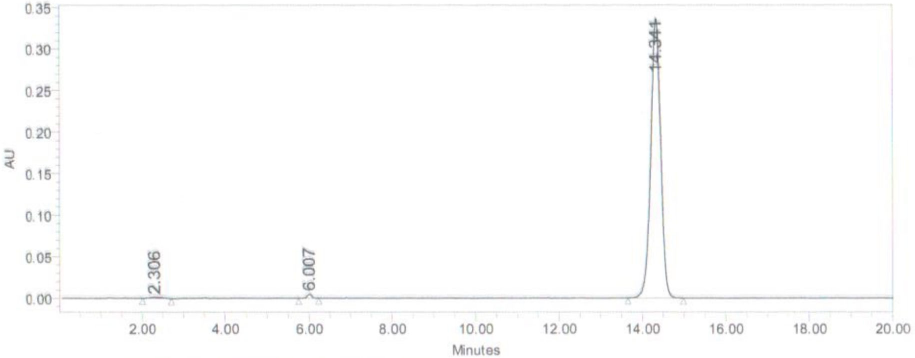

	RT	Area	% Area	Height
1	2.306	22951	0.41	1124
2	6.007	35928	0.64	4926
3	14.341	5531652	98.95	336943

SAMPLE INFORMATION

Sample Name:	Nitro 1mg/ml in ACN	Acquired By:	System
Sample Type:	Unknown	Sample Set Name:	Test_Xbridgecolumn_01Jul10
Vial:	42	Acq. Method Set:	Azetidinone_ACN_H20_MS
Injection #:	1	Processing Method:	Azetidinone_PM
Injection Volume:	10.00 ul	Channel Name:	2487Channel 1
Run Time:	25.0 Minutes	Proc. Chnl. Descr.:	262nm
Date Acquired:	01/07/2010 10:12:22 IST		
Date Processed:	01/07/2010 11:00:23 IST		

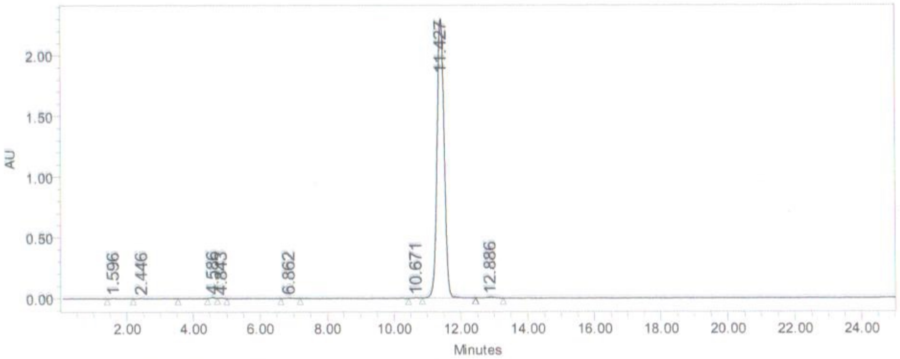

	RT	Area	% Area	Height
1	1.596	6814	0.02	1149
2	2.446	7823	0.02	711
3	4.586	57818	0.17	9109
4	4.843	25047	0.08	3921
5	6.862	33128	0.10	3438
6	10.671	27080	0.08	2392
7	11.427	32962918	99.14	2301660
8	12.886	127835	0.38	8135
