# Supplementary material for: Antiproliferative and Tubulin-Destabilising Effects of 3-(Prop-1-en-2-yl)azetidin-2-Ones and Related Compounds in MCF-7 and MDA-MB-231 Breast Cancer Cells
Source: Pharmaceuticals (Basel). 2023 Jul 13;16(7):1000. doi: 10.3390/ph16071000 (PMC10385824; doi:10.3390/ph16071000)
Supplement: Supplementary file 1 [file pharmaceuticals-16-01000-s001.zip › 10p.pdf]

Empower2 software

Result Set Report

Sample Set Name:	Test_Xbridgecolumn_01Jul10	Processed By:	System/Administrator
Sample Set Method:	Test_Xbridgecolumn_01Jul10	Printed By:	System
System Node:	Gx620image	Result Set ID:	1401
System Name:	HPLC_2695_2487	# of Results:	3
Acquired By:	System		
Sample Set Start Date:	01/07/2010 09:14:02 IST		
Sample Set Finish Date:	01/07/2010 10:37:34 IST		
Result Set Date:	01/07/2010 11:00:23 IST		

Sample Set Table

	Sample Name	Sample Type	Vial	Inj #	Run Time (Minutes)	Injection Volume (ul)	Acquistion Method Set	Sample Weight	Processed Channel Descr.	Dilution
1	Hydroxy 1mg/ml in MeOH(unfilter)	Unknown	45	1	20.00	10.00	Azetidinone_ACN_H20_MS	1.00000	262nm	1.00000
2	H in 1mg/ml in MeOH(unfilter)	Unknown	5	1	20.00	10.00	Azetidinone_ACN_H20_MS	1.00000	262nm	1.00000
3	Nitro 1mg/ml in ACN	Unknown	42	1	25.00	10.00	Azetidinone_ACN_H20_MS	1.00000	262nm	1.00000

SAMPLE INFORMATION

Sample Name:	Hydroxy 1mg/ml in MeOH(unfilter)	Acquired By:	System
Sample Type:	Unknown	Sample Set Name:	Test_Xbridgecolumn_01Jul10
Vial:	45	Acq. Method Set:	Azetidinone_ACN_H20_MS
Injection #:	1	Processing Method:	Azetidinone_PM
Injection Volume:	10.00 ul	Channel Name:	2487Channel 1
Run Time:	20.0 Minutes	Proc. Chnl. Descr.:	262nm
Date Acquired:	01/07/2010 09:30:14 IST		
Date Processed:	01/07/2010 11:00:23 IST		

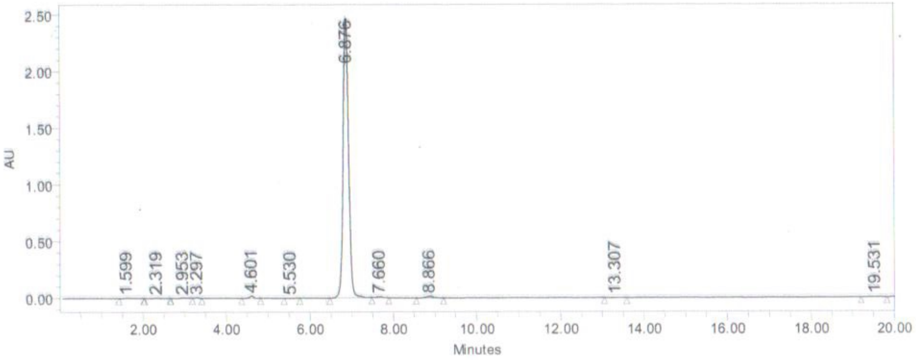

	RT	Area	% Area	Height
1	1.599	14968	0.07	2184
2	2.319	31538	0.14	1482
3	2.953	11280	0.05	700
4	3.297	13126	0.06	1964
5	4.601	109924	0.50	18823
6	5.530	6256	0.03	872
7	6.876	21679355	98.08	2478760
8	7.660	71103	0.32	8104
9	8.866	135350	0.61	12621
10	13.307	10079	0.05	699
11	19.531	20717	0.09	1172
