# Supplementary material for: Antiproliferative and Tubulin-Destabilising Effects of 3-(Prop-1-en-2-yl)azetidin-2-Ones and Related Compounds in MCF-7 and MDA-MB-231 Breast Cancer Cells
Source: Pharmaceuticals (Basel). 2023 Jul 13;16(7):1000. doi: 10.3390/ph16071000 (PMC10385824; doi:10.3390/ph16071000)
Supplement: Supplementary file 1 [file pharmaceuticals-16-01000-s001.zip › 10r.pdf]

Result Set Report

Sample Set Name:	NH2 test_22Jul, NH2 test_A_22Jul,	Processed By:	System/Administrator
Sample Set Method:	NH2 test_22Jul, NH2 test_A_22Jul,	Printed By:	System
System Node:	Gx620image	Result Set ID:	4572, 5171, 5176, 5183, 5186, 5191
System Name:	HPLC_2695_2487	# of Results:	23
Acquired By:	System		
Sample Set Start Date:	22/07/2010 09:57:52 IST, 22/07/2010 10:33:37 IST, 22/07/2010 12:01:36 IST, 22/07/2010 13:49:31		
Sample Set Finish Date:	22/07/2010 10:29:05 IST, 22/07/2010 11:35:42 IST, 22/07/2010 13:34:42 IST, 22/07/2010 14:15:34		
Result Set Date:	22/07/2010 10:34:43 IST, 27/07/2010 14:03:40 IST, 27/07/2010 14:03:53 IST, 27/07/2010 14:04:03		

Sample Set Table

	Sample Name	Sample Type	Vial	Inj #	Run Time (Minutes)	Injection Volume (ul)	Acquistion Method Set	Sample Weight	Processed Channel Descr.	Dilution
1	NH2 0.5mg/ml in ACN test	Unknown	25	1	30.00	10.00	Azetidinone_MS	1.00000	262nm	1.00000
2	NH2 0.5mg/ml in ACN test	Unknown	25	1	30.00	10.00	Azetidinone_MS	1.00000	262nm	1.00000
3	NH2 0.5mg/ml in ACN test	Unknown	25	1	30.00	10.00	Azetidinone_MS	1.00000	220nm	1.00000
4	NH2 0.025mg/ml in ACN test	Unknown	26	1	30.00	10.00	Azetidinone_MS	1.00000	262nm	1.00000
5	NH2 0.025mg/ml in ACN test	Unknown	26	1	30.00	10.00	Azetidinone_MS	1.00000	220nm	1.00000
6	NH2 0.5mg/ml in ACN test	Unknown	25	1	25.00	10.00	Azetidinone_NH2_MS	1.00000	262nm	1.00000
7	NH2 0.5mg/ml in ACN test	Unknown	25	1	25.00	10.00	Azetidinone_NH2_MS	1.00000	220	1.00000
8	NH2 0.025mg/ml in ACN test	Unknown	26	1	25.00	10.00	Azetidinone_NH2_MS	1.00000	262nm	1.00000
9	NH2 0.025mg/ml in ACN test	Unknown	26	1	25.00	10.00	Azetidinone_NH2_MS	1.00000	220	1.00000
10	NH2 0.0025mg/ml in ACN test	Unknown	27	1	25.00	10.00	Azetidinone_NH2_MS	1.00000	262nm	1.00000
11	NH2 0.0025mg/ml in ACN test	Unknown	27	1	25.00	10.00	Azetidinone_NH2_MS	1.00000	220	1.00000
12	NH2 0.025mg/ml in ACN test	Unknown	26	1	25.00	10.00	Azetidinone_NH2_MS	1.00000	262nm	1.00000
13	NH2 0.025mg/ml in ACN test	Unknown	26	1	25.00	10.00	Azetidinone_NH2_MS	1.00000	220	1.00000
14	NH2 0.5 mg/ml in ACN test	Unknown	25	1	20.00	10.00	Azetidinone_NH2_MS	1.00000	262nm	1.00000
15	NH2 0.5 mg/ml in ACN test	Unknown	25	1	20.00	10.00	Azetidinone_NH2_MS	1.00000	220	1.00000
16	NH2 0.025mg/ml in ACN test	Unknown	26	1	20.00	10.00	Azetidinone_NH2_MS	1.00000	262nm	1.00000
17	NH2 0.025mg/ml in ACN test	Unknown	26	1	20.00	10.00	Azetidinone_NH2_MS	1.00000	220	1.00000
18	NH2 0.5 mg/ml in ACN test	Unknown	25	1	20.00	10.00	Azetidinone_NH2_MS	1.00000	262nm	1.00000
19	NH2 0.5 mg/ml in ACN test	Unknown	25	1	20.00	10.00	Azetidinone_NH2_MS	1.00000	220	1.00000
20	NH2 0.025mg/ml in ACN test	Unknown	26	1	20.00	10.00	Azetidinone_NH2_MS	1.00000	262nm	1.00000
21	NH2 0.025mg/ml in ACN test	Unknown	26	1	20.00	10.00	Azetidinone_NH2_MS	1.00000	220	1.00000
22	NH2 0.0025mg/ml in ACN test	Unknown	27	1	20.00	10.00	Azetidinone_NH2_MS	1.00000	262nm	1.00000
23	NH2 0.0025mg/ml in ACN test	Unknown	27	1	20.00	10.00	Azetidinone_NH2_MS	1.00000	220	1.00000

SAMPLE INFORMATION

Sample Name:	NH2 0.5mg/ml in ACN test	Acquired By:	System
Sample Type:	Unknow n	Sample Set Name:	NH2 test_22Jul
Vial:	25	Acq. Method Set:	Azetidinone_MS
Injection #:	1	Processing Method:	Azetidinone_ PM
Injection Volume:	10.00 ul	Channel Name:	2487Channel 1
Run Time:	30.0 Minutes	Proc. Chnl. Descr.:	262nm
Date Acquired:	22/07/2010 09:58:54 IST		
Date Processed:	22/07/2010 10:34:43 IST		

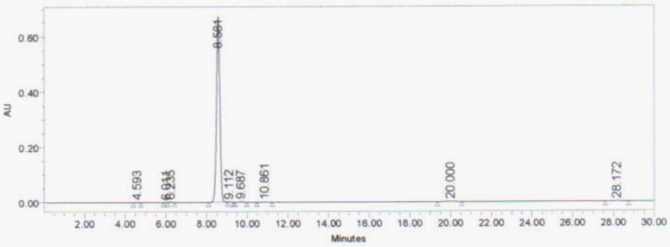

Channel Description 262nm

	Peak Name	RT	Area	% Area	Height	Channel Description
1	Aniline	3.200				262nm
2		4.593	1585	0.02	234	262nm
3	Aldehyde	4.900				262nm
4		6.011	1452	0.02	226	262nm
5		6.235	13992	0.18	1714	262nm
6		8.581	7722970	98.86	672714	262nm
7		9.112	5324	0.07	588	262nm
8		9.687	21976	0.28	1775	262nm
9	Imine	10.250				262nm
10		10.861	4400	0.06	298	262nm
11		20.000	32467	0.42	1342	262nm
12		28.172	8095	0.10	260	262nm

SAMPLE INFORMATION

Sample Name:	NH2 0.5mg/ml in ACN test	Acquired By:	System
Sample Type:	Unknow n	Sample Set Name:	NH2 test_A_22Jul
Vial:	25	Acq. Method Set:	Azetidinone_MS
Injection #:	1	Processing Method:	Azetidinone_ PM
Injection Volume:	10.00 ul	Channel Name:	2487Channel 1, 2487Channel 2
Run Time:	30.0 Minutes	Proc. Chnl. Descr.:	220nm, 262nm
Date Acquired:	22/07/2010 10:34:40 IST		
Date Processed:	27/07/2010 14:03:41 IST		

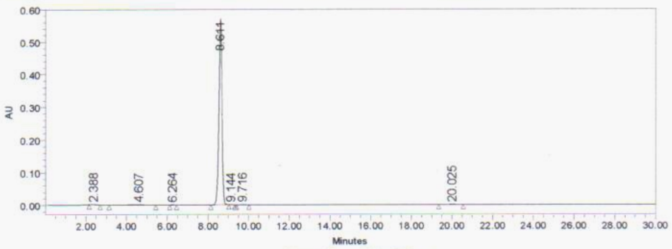

Channel Description 262nm

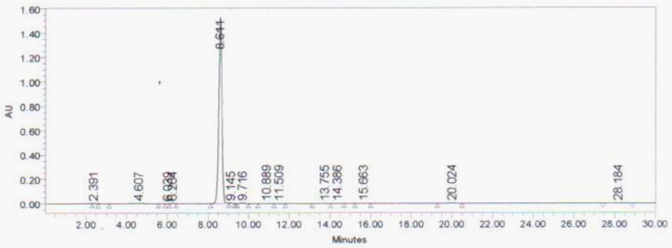

Channel Description 220nm

	Peak Name	RT	Area	% Area	Height	Channel Description
1		2.388	14391	0.22	1242	262nm

	Peak Name	RT	Area	% Area	Height	Channel Description
2		2.391	14272	0.08	1758	220nm
3	Aniline	3.200				262nm
4	Aniline	3.200				220nm
5		4.607	68055	1.06	1344	262nm
6		4.607	122648	0.70	2339	220nm
7	Aldehyde	4.900				262nm
8	Aldehyde	4.900				220nm
9		6.039	3432	0.02	539	220nm
10		6.264	31838	0.18	4009	220nm
11		6.264	11487	0.18	1464	262nm
12		8.611	6298322	97.77	572506	262nm
13		8.611	17094574	97.93	1528801	220nm
14		9.144	4959	0.08	556	262nm
15		9.145	14909	0.09	1670	220nm
16		9.716	17925	0.28	1494	262nm
17		9.716	53814	0.31	4462	220nm
18	Imine	10.250				262nm
19	Imine	10.250				220nm
20		10.889	9898	0.06	683	220nm
21		11.509	7865	0.05	562	220nm
22		13.755	6579	0.04	322	220nm
23		14.386	4952	0.03	305	220nm
24		15.663	7038	0.04	385	220nm
25		20.024	65922	0.38	2758	220nm
26		20.025	26574	0.41	1111	262nm
27		28.184	18650	0.11	556	220nm
