# Supplementary material for: Antiproliferative and Tubulin-Destabilising Effects of 3-(Prop-1-en-2-yl)azetidin-2-Ones and Related Compounds in MCF-7 and MDA-MB-231 Breast Cancer Cells
Source: Pharmaceuticals (Basel). 2023 Jul 13;16(7):1000. doi: 10.3390/ph16071000 (PMC10385824; doi:10.3390/ph16071000)
Supplement: Supplementary file 1 [file pharmaceuticals-16-01000-s001.zip › MJM revision 2 Supplementary information .pdf]

Supplementary Information

Supplementary Information

Table S1: Crystal data and structure refinement for **8h**, **8i**, **9h**, **10f**, **10h**, **11b-d 11h**, **11t**

Table S2: Stability study for compounds **9q,15a, 10h, 10q, 10p, 10r** and **CA-4** at pH 4.0, pH 7.5, pH 9.0 and in plasma

Table S3: Stability study for compounds **10h, 10q, 10p** and **10r** (HCl, NaOH, heat (80 °C), Light, H_2O_2)

Table S4: Tier-1 Profiling Screen of Selected β-Lactams

Table S5: ADMET and Lipinski Properties for Selected β-Lactams

Table S6: Antitumour Evaluations of compound **9q** in the NCI60 cell line *in vitro* primary one dose screen

Table S7: Comparative Antitumour Evaluations of compounds **9h, 9q, 9s, 10p, 10h, 10r, 15a, 15b** in the NCI60 *leukaemia, non-small cell lung cancer*, *colon cancer* and *CNS cancer* cell lines *in vitro* primary screen

Table S8: Comparative Antitumour Evaluations of compounds **9h, 9q, 9s, 10p, 10h, 10r, 15a, 15b** in the NCI60 *melanoma*, *ovarian cancer*, *renal cancer* and *breast cancer* cell line *in vitro* primary screen

Table S9: Standard COMPARE analysis of β-lactam **9q**

Table S10: Standard COMPARE analysis of β-lactam **9s**

Table S11: Docking scores for selected β-lactams

Table S12: X-Ray data and torsional angles for compounds **9h, 10h, 10f, 11a-c, 11h.**

Table S13. X-ray crystal structure of compounds **8h**, **8i** and **11t**

Table S14: X-ray crystal structure of compounds **10f, 11b, 11c, 11d.**

Figures S1-S20: ^{1}H NMR and ^{13}C NMR spectra

Figure S21: Stability study for compounds **9q, 15a, 10h, 10q, 10p, 10r** and **CA-4** at pH 4.0, pH 7.5, pH 9.0 and in plasma.

Figure S22: Flexible alignment of **11p** and combretastatin A-4

Figure S23: Inter-atomic distances between oxygen atoms for compounds **25, 11p** and CA-4

Figure S24: Protein ligand interactions for β-Lactam **11p** docked in the colchicine-binding site of tubulin

Figure S25: Overlay of the X-ray structure of tubulin crystallised with DAMA Colchicine with β-lactams

Figure S26: β-Lactams **9s, 10h, 11p** and **11r** docked in the colchicine-binding site of tubulin.

Table S1. Crystal data and structure refinement for 8h, 8i, 9h, 10f, 10h, 11b-d, 11h, 11t.

Identification code	**8h**	**8i**	**9h**†	**10f**	**10h**
Empirical formula	$C_{17}H_{19}NO_4$	$C_{18}H_{21}NO_4$	$C_{22}H_{25}NO_5$	$C_{21}H_{23}NO_4$	$C_{22}H_{25}NO_5$
Formula weight	301.33	315.36	383.43	353.40	383.43
Temperature/K	100(2)	100(2)	100(2)	100(2)	100(2)
Crystal system	monoclinic	monoclinic	monoclinic	monoclinic	monoclinic
Space group	$P2_1/c$	$P2_1/c$	$P2_1/c$	$P2_1/n$	$P2_1/c$
a (Å)	13.9299(9)	22.5651(4)	8.9915(3)	10.0918(11)	9.0246(6)
b (Å)	15.2516(10)	5.22320(10)	21.9379(8)	8.7491(9)	22.6999(13)
c (Å)	7.2194(5)	13.9585(2)	20.6602(8)	21.330(2)	19.3997(11)
α (°)	90	90	90	90	90
β (°)	94.187(2)	100.4590(10)	90.091(2)	99.292(3)	90.4597(17)
γ (°)	90	90	90	90	90
Volume ($Å^3$)	1529.69(18)	1617.84(5)	4075.3(3)	1858.6(3)	3974.0(4)
Z	4	4	8	4	8
ρ_{calc} (g/cm^3)	1.308	1.295	1.250	1.263	1.282
μ (mm^{-1})	0.093	0.091	0.724	0.087	0.091
F(000)	640.0	672.0	1632.0	752.0	1632.0
Crystal size (mm^3)	0.3 × 0.25 × 0.16	0.14 × 0.1 × 0.06	0.2 × 0.07 × 0.07	0.364 × 0.312 × 0.124	0.31 × 0.11 × 0.08
Radiation	Mo Kα	Mo Kα	Cu Kα	Mo Kα	Mo Kα
Reflections collected	26793	95360	43185	21577	69795
Independent reflections	3528 R_{int} = 0.0227, R_{sigma} = 0.0120	4759 R_{int} = 0.0496, R_{sigma} = 0.0232	7062 R_{int} = 0.0421, R_{sigma} = 0.0284	4630 R_{int} = 0.0337, R_{sigma} = 0.0254	8185 R_{int} = 0.1336, R_{sigma} = 0.0632
Data/restraints/parameters	3528/0/203	4759/0/212	7062/0/516	4630/0/239	8185/72/551
Goodness-of-fit on F^2	1.029	1.035	1.041	1.022	1.070
Final R indexes [I≥2σ (I)]*	R_1 = 0.0319, wR_2 = 0.0824	R_1 = 0.0436, wR_2 = 0.0996	R_1 = 0.0339, wR_2 = 0.0829	R_1 = 0.0388, wR_2 = 0.0916	R_1 = 0.0541, wR_2 = 0.1008
Final R indexes [all data]	R_1 = 0.0381, wR_2 = 0.0872	R_1 = 0.0672, wR_2 = 0.1127	R_1 = 0.0391, wR_2 = 0.0868	R_1 = 0.0537, wR_2 = 0.1014	R_1 = 0.1163, wR_2 = 0.1264
Largest diff. peak/hole (e $Å^{-3}$)	0.33/-0.19	0.44/-0.27	0.34/-0.24	0.30/-0.20	0.25/-0.24
CCDC No.	2241430	2241431	1820359	2241432	2241433

*$R_1 = \Sigma||F_o| - |F_c||/\Sigma|F_o|$, $wR_2 = [\Sigma w(F_o^2 - F_c^2)^2/\Sigma w(F_o^2)^2]^{1/2}$.

† See reference Shu Wang, Azizah M. Malebari, Thomas F. Greene, Niamh M. O'Boyle, Darren Fayne, Seema M. Nathwani, Brendan Twamley, Thomas McCabe, Niall O. Keely, Daniela M. Zisterer, Mary J. Meegan CCDC 1820359: Experimental Crystal Structure Determination, 2020, DOI: 10.5517/ccdc.csd.cc1z378l

Identification code	**11d**	**11b**	**11c**	**11h**	**11t**
Empirical formula	$C_{22}H_{22}FNO_4$	$C_{22}H_{22}ClNO_4$	$C_{22}H_{22}BrNO_4$	$C_{23}H_{25}NO_5$	$C_{11}H_{13}NO$
Formula weight	383.40	399.85	444.31	395.44	175.22
Temperature/K	100(2)	100(2)	100(2)	100(2)	100(2)
Crystal system	monoclinic	monoclinic	monoclinic	orthorhombic	triclinic
Space group	$P2_1/c$	$P2_1/c$	$P2_1/c$	Pbca	P-1
a (Å)	14.5661(11)	14.6264(9)	14.6668(8)	19.2285(12)	8.7662(5)
b (Å)	7.5756(5)	7.6621(5)	7.7384(5)	9.1143(6)	9.7900(5)
c (Å)	18.2634(13)	18.5583(11)	18.6524(10)	23.8043(16)	11.6028(6)
α (°)	90	90	90	90	78.3022(15)
β (°)	108.9192(19)	108.9354(14)	108.6557(14)	90	78.8925(15)
γ (°)	90	90	90	90	89.9494(15)
Volume ($Å^3$)	1906.4(2)	1967.3(2)	2005.8(2)	4171.8(5)	956.06(9)
Z	4	4	4	8	4
ρ_{calc} (g/cm^3)	1.336	1.350	1.471	1.259	1.217
μ (mm^{-1})	0.098	0.223	2.077	0.089	0.078
F(000)	808.0	840.0	912.0	1680.0	376.0
Crystal size (mm^3)	0.32 × 0.09 × 0.07	0.491 × 0.36 × 0.082	0.361 × 0.139 × 0.057	0.36 × 0.16 × 0.042	0.464 × 0.244 × 0.182
Radiation	Mo Kα	Mo Kα	Mo Kα	Mo Kα	Mo Kα
Reflections collected	41050	44829	48902	47449	37132
Independent reflections	4749<br>R_{int} = 0.1009,<br>R_{sigma} = 0.0484	4943<br>R_{int} = 0.0459,<br>R_{sigma} = 0.0229	5062<br>R_{int} = 0.0799,<br>R_{sigma} = 0.0363	4300<br>R_{int} = 0.1050,<br>R_{sigma} = 0.0427	4817<br>R_{int} = 0.0310,<br>R_{sigma} = 0.0193
Data/restraints/parameters	4749/0/257	4943/0/257	5062/0/257	4300/0/267	4817/0/244
Goodness-of-fit on F^2	1.058	1.050	1.052	1.082	1.038
Final R indexes [I≥2σ (I)]*	R_1 = 0.0523,<br>wR_2 = 0.0914	R_1 = 0.0358,<br>wR_2 = 0.0829	R_1 = 0.0326,<br>wR_2 = 0.0598	R_1 = 0.0480,<br>wR_2 = 0.0938	R_1 = 0.0382,<br>wR_2 = 0.0916
Final R indexes [all data]	R_1 = 0.0922,<br>wR_2 = 0.1076	R_1 = 0.0495,<br>wR_2 = 0.0911	R_1 = 0.0560,<br>wR_2 = 0.0675	R_1 = 0.0906,<br>wR_2 = 0.1130	R_1 = 0.0480,<br>wR_2 = 0.0987
Largest diff. peak/hole (e $Å^{-3}$)	0.36/-0.24	0.38/-0.27	0.51/-0.43	0.20/-0.21	0.31/-0.22
CCDC No.	2241434	2241435	2241436	2241437	2241438

$*R_1 = \Sigma||F_o| - |F_c||/\Sigma|F_o|$, $wR_2 = [\Sigma w(F_o^2 - F_c^2)^2/\Sigma w(F_o^2)^2]^{1/2}$.

Table S2: Stability study for compounds **9q, 15a, 10h, 10q, 10p, 10r** and CA-4 at pH 4.0, pH 7.5, pH 9.0 and in plasma [a]

Compound	pH 4.0 % Remaining	pH 7.4 % Remaining	pH 9.0 % Remaining	Plasma % Remaining
**9q**[b]	33	35	34	80
**15a**[b]	96	98	nd[e]	97
**10h**[c]	28	41	24	100
**10q**[c]	26	25	22	90
**10p**[c]	98	99	96	97
**10r**[c]	96	99	83	97
**CA-4**[d]	97	89	90	95

[a] The stability study for compounds **9q, 15a, 10h, 10q, 10p, 10r** and CA-4 was performed by analytical HPLC, [Symmetry® column (C18, 5 mm, 4.6 x150 mm), Dual Wavelength Absorbance detector (Waters 2487), binary HPLC pump (Waters 1525), and Autosampler (Waters 717 plus)], with mobile phase acetonitrile (80%)/water (20%), flow rate 1 mL/min over 15 min and detection at λ 254 nm. Stock solutions of the compounds (5 mg/mL in 10 mL mobile phase) were used with appropriate dilutions of 0.5 mg/mL, 0.25 mg/mL, 0.125 mg/mL, 0.0625 mg/mL, 0.03125 mg/mL, 0.015625 and 0.0078 mg/mL were prepared for the calibration curve. [b]Stability study over 24 h; [c] Stability study over 264 h; [d.]Stability study over 7 h; Nd compound not detected

Table S3: Stability study for compounds **10h, 10q, 10p** and **10r** (HCl, NaOH, heat (80 ^{o}C), Light, H_2O_2)[a]

Compound		10h	10q	10p	10r
% Remaining	1M HCl (0.1 mL, 20 ^{o}C, 4h)	97.5	99.2	91.3	91.8
% Remaining	1M HCl (0.2 mL, 50 ^{o}C, 4h)	99.6	98.7	91.3	92.2
% Remaining	1M NaOH (0.1 mL, 20 ^{o}C, 4h)	96	97.8	95.2	Nd
% Remaining	1M NaOH (0.2 mL, 50 ^{o}C, 4h)	Nd[b]	97.8	82.1	83.1
% Remaining	80 ^{o}C (heating block, 5 h)	100	100	100	100
% Remaining	Daylight (acetonitrile (80%):water (20%) 13 days)	100	100	100	93
% Remaining	Hydrogen peroxide (30 % H_2O_2, 0.2 mL), 20 ^{o}C, 6h	100	92	79	8

[a] All samples were analysed using acetonitrile (80%):water (20%) as the mobile phase over 10 min and a flow rate of 1 mL/min. Stock solutions are prepared by dissolving 5 mg of compounds in 10 mL of mobile phase. [b] Nd compound not detected.

Table S4. Tier-1 Profiling Screen of Selected β-Lactams[a]

Compound	Cpd No.	ADMET Solubility[b]	ADMET Solubility Level[c]	ADMET BBB[d]	ADMET BBB Level[e]	ADMET CYP2D6 Prediction[f]	ADMET Hepatotoxic Prediction[g]
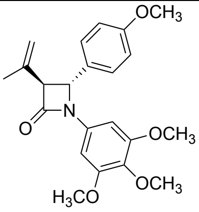	**9h**	-4.7100	2	0.070000	1	false	false
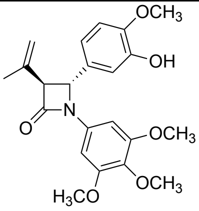	**9q**	-4.2720	2	-0.33400	2	false	true
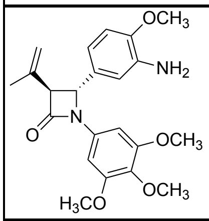	**9s**	-4.5090	2	-0.58100	3	false	false
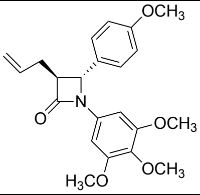	**10h**	-4.5660	2	0.073000	1	false	false
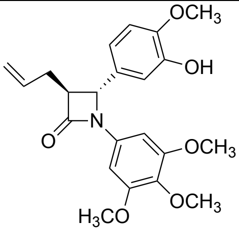	**10p**	-4.1280	2	-0.33100	2	false	false
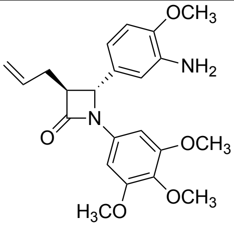	**10r**	-4.3640	2	-0.57800	3	false	false
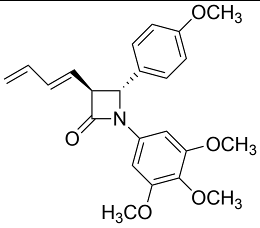	**11h**	-4.5610	2	0.077000	1	false	false

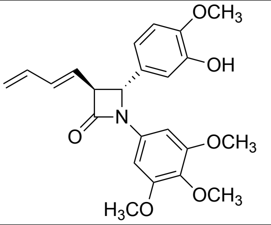	**11p**	-4.1170	2	-0.32700	2	false	false
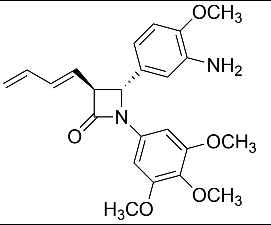	**11r**	-4.3550	2	-0.57400	3	false	false
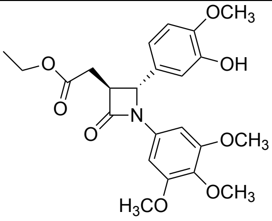	**17g**	-3.4130	3	-0.98400	3	false	false
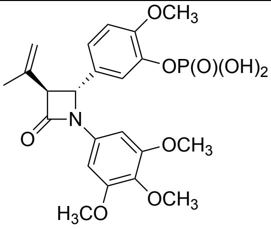	**15a**	-4.23	2	-	4	false	true

[a]Calculated using Pipeline Pilot Professional (v8.5.0.200) BIOVIA, Dassault Systèmes
[b]ADMET Solubility: Log of the water solubility at 25 °C (LogSw)(mol/L)
[c]ADMET Solubility Level: Ranking of the solubility values into the following classes: 0: Extremely Low; 1: Very Low; 2: Low; 3: Good; 4: Optimal; 5: Very Soluble
[d]ADMET BBB: Predicts the blood brain barrier penetration of a molecule, defined as the ratio of the concentrations of solute (compound) on the both sides of the membrane after oral administration.
[e]ADMET Blood Brain Barrier Absorption (BBB) Level: Ranking of LogBBB values into one of the following levels: 0: Very High; 1: High; 2: Medium; 3: Low; 4: Undefined (molecule is outside the confidence area of the regression model used to calculate LogBB)
[f]CYP2D6 inhibitor prediction

[g]Human hepatoxicity prediction

Table S5. Lipinski-Properties for Selected β-Lactams[a]

Compound	Cpd No.	ADMET Absorption Level[b]	ADMET PPB Prediction[c]	ALogP	MW	HBA	HBD	Rot Bonds	Molecular Volume	Molecular Polar Surface Area
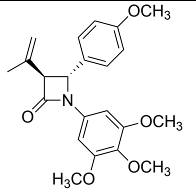	**9h**	0	true	3.6110	383.44	5	0	7	266.85	57.230
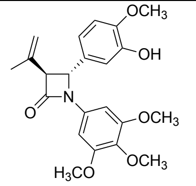	**9q**	0	true	3.3690	399.44	6	1	7	272.68	77.460
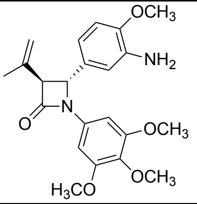	**9s**	0	true	2.8640	398.45	6	1	7	278.85	83.250
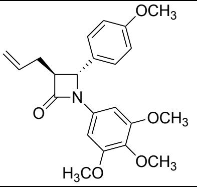	**10h**	0	true	3.6210	383.44	5	0	8	265.13	57.230
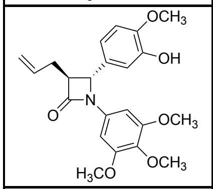	**10p**	0	true	3.3790	399.44	6	1	8	269.94	77.460
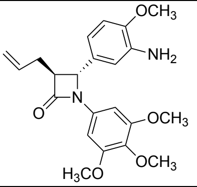	**10r**	0	true	2.8740	398.45	6	1	8	277.82	83.250
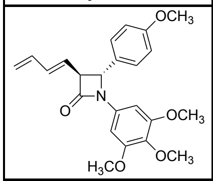	**11h**	0	true	3.6320	395.45	5	0	8	276.45	57.230
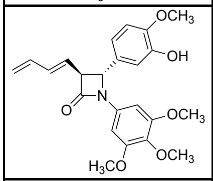	**11p**	0	true	3.3900	411.45	6	1	8	277.48	77.460
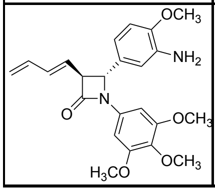	**11r**	0	true	2.8860	410.46	6	1	8	280.57	83.250

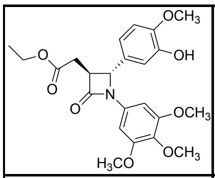	**17g**	0	true	2.6100	445.46	8	1	10	299.43	103.76
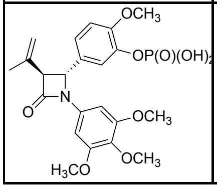	**15a**	1	true	3.12	479.42	9	2	9	305.95	133.80

[a]Calculated using Pipeline Pilot Professional (v8.5.0.200) BIOVIA, Dassault Systèmes

[b]ADMET Calculates ADMET Passive Intestinal Absorption properties. Accelrys passive intestinal absorption model. Absorption Level: Ranking of the molecule into one of the following levels: 0: Good; 1: Moderate; 2: Poor; 3: Very Poor

[c]ADMET Plasma Protein Binding (PPB) Prediction: If true, the compound is predicted to be a binder (>=90%). Otherwise, it is predicted to be a weak or nonbinder(<90%).

Table S6: Antitumour Evaluations of compound **9q** in the NCI60 cell line *in vitro* primary one dose screen[a]

Panel	*Cell line*	*% Growth*
Leukaemia		
	HL-60(TB)	-51.24
	K-562	2.20
	MOLT-4	-15.34
	RPMI-8226	11.06
	SR	-2.84
Non-Small Cell Lung Cancer		
	A549/A TCC	14.76
	EKVX	68.88
	HOP-62	22.22
	HOP-92	16.15
	NCI-H23	14.46,
	NCI-H460	3.52
	NCI-H522	-43.45
Colon Cancer		
	COLO 205	-27.00
	HCC-2998	-6.59
	HCT-116	-10.74
	HCT-15	15.23
	HT29	2.66
	KM12	-24.63
	SW-620	17.89
CNS Cancer		
	SF-268	19.27
	SF-295	20.26
	SF-539	-8.30
	SNB-19	37.93
	SNB-75	41.90
	U251	9.06
Melanoma		
	LOX IMVI	30.61
	MALME-3M	63.23
	M14	3.41
	MDA-MB-435	-13.45
	SK-MEL-2	9.55
	SK-MEL-28	65.72
	SK-MEL-5	-0.90
	UACC-257	74.47
	UACC-62	38.81
Ovarian Cancer		
	IGROV1	29.32
	OVCAR-3	-40.14
	OVCAR-4	25.51
	OVCAR-5	44.67
	OVCAR-8	14.64
	NCI/ADR-RES	-2.95
	SK-OV-3	8.15
Renal Cancer		
	786-0	4.28
	A498	-13.19
	ACHN	34.94
	CAKI-1	44.03
	RXF 393	18.79
	SN12C	31.39
	TK-10	46.63

	UO-31	29.39
Prostate Cancer		
	PC-3	27.31
	DU-145	-52.00
Breast Cancer		
	MCF7	15.44
	MDA-MB-231/ATCC	-6.67
	BT-549	-9.94
	T-47D	67.35
	MDA-MB-468	13.36
Mean	13.02	
Delta	65.02	
Range	126.47	

[a]NCI *in vitro* human tumour cell screen 5 dose assay for compounds **9q (S-762032)**: The compound was evaluated at 10 μM concentrations over the NCI 60 cell line panel and incubations were carried out over 48 h exposures to the drug.

Table S7: Comparative Antitumour Evaluations of compounds **9h, 9q, 9s, 10p, 11h, 10r, 15a, 15b** in the NCI60 *leukaemia, non-small cell lung cancer*, *colon cancer* and *CNS cancer* cell lines *in vitro* primary screen[a]

Cell line	9h GI_{50} (µM)	9q GI_{50} (µM)	9s GI_{50} (µM)	10p GI_{50} (µM)	11h GI_{50} (µM)	11r GI_{50} (µM)	15a GI_{50} (µM)	15b GI_{50} (µM)
NCI code	762037	762032	762042	762033	762039	762044	775044	775045
Leukemia								
CCRF-CEM	0.0375	0.0192	0.0377	0.0361	0.0378	0.0365	0.0557	0.356
HL-60(TB)	0.0331	0.0211	0.0283	0.0323	0.0338	0.0295	0.0279	0.305
K-562	0.0410	0.0352	0.0383	0.0431	0.0410	0.0393	0.045	0.374
MOLT-4	0.0397	0.0202	0.0372	0.0408	0.0369	0.0396	0.0852	0.493
RPMI-8226	0.0399	0.0111	0.0359	0.0406	0.0384	0.0348	0.105	0.478
SR	0.0385	< 0.010	0.0304	0.0314	0.0356	0.0335	0.0531	0.324
Non-Small Cell Lung Cancer								
A549/ATCC	0.0408	0.0278	0.0415	0.0373	0.0364	0.0380	0.0596	0.353
EKVX	0.0813	[c]Nd	0.587	0.0764	0.0834	0.0880	[c]Nd	[c]Nd
HOP-62	0.0576	0.0521	0.0521	0.0610	0.0558	0.0525	0.105	0.374
HOP-92	0.0719	0.0154	0.0437	0.0238	0.0244	0.0253	0.315	11.2
NCI-H226	[c]Nd	[c]Nd	0.0435	0.0560	0.0768	0.0564	>100	>100
NCI-H23	0.0237	0.0237	0.0258	0.0307	0.0321	0.0300	0.0902	0.368
NCI-H332M	[c]Nd	0.0477	0.0511	0.0512	0.0626	0.0579	0.082	0.592
NCI-H460	0.0356	0.0300	0.0332	0.0320	0.0332	0.0332	0.0448	0.325
NCI-H552	0.0222	<0.0100	0.0146	0.0151	0.0151	0.0169	0.026	0.237
Colon Cancer								

COLO 205	0.0271	0.284	0.0197	0.563	0.0265	0.0231	0.312	1.23
HCT-2998	0.0307	0.0301	0.0291	0.0299	0.0300	0.0270	0.238	0.452
HCT-116	0.0317	0.0149	0.0302	0.0249	0.0264	0.0272	0.0506	0.319
HCT-15	0.0406	0.0299	0.0416	0.0367	0.0405	0.0370	0.0707	0.298
HT29	0.0354	0.187	0.0337	0.360	0.0332	0.0336	0.577	3.47
KM12	0.0340	<0.0100	0.0231	0.0224	0.0304	0.0331	0.0381	0.18
SW-620	0.0424	0.0334	0.0394	0.0422	0.0438	0.0429	0.0807	0.373
CNS Cancer								
SF-268	[c]Nd	0.0229	[c]Nd	[c]Nd	[c]Nd	[c]Nd	0.0981	0.447
SF295	0.043	<0.0100	0.0344	0.0291	0.0309	0.0301	0.0324	0.137
SF539	0.0352	[c]Nd	0.0402	0.0382	0.0332	0.0390	0.0398	0.132
SNB-19	0.0675	0.0588	[c]Nd	0.0589	0.0654	0.0649	0.429	0.477
SNB-75	0.038	0.0473	0.0601	0.0448	0.0307	0.0368	0.0361	0.116
U251	0.0360	0.0305	0.0375	0.0317	0.0322	0.0357	0.0395	0.276
Prostate cancer								
PC-3	0.0453	0.0201	0.0379	0.0403	0.0404	0.0392	0.0494	0.278
DU-145	0.0291	0.0215	0.0236	0.0266	0.0270	0.0260	0.0809	0.368

[a] NCI *in vitro* human tumour cell screen 5 dose assay for compounds **9h (S-762037), 9q (S-762032), 9s (S-762042), 10p (D-762033), 11h (D-762039), 11r (D-762044), 15a (S-775044)** and **15b (S-775045)**; The compounds were evaluated using five different concentrations (100 µM, 10 µM, 1 µM, 0.1 µM and 0.01 µM) over the NCI 60 cell line panel and incubations were carried out over 48 h exposures to the drug; [b]GI_{50} is the molar concentration of the compound causing 50% inhibition of growth of the tumour cells; [c]Nd: Not determined.

Table S8: Results of Comparative Antitumour Evaluations of compounds **9h, 9q, 9s, 10p, 11h, 11r, 15a, 15b** in the NCI60 *melanoma*, *ovarian cancer*, *renal cancer* and *breast cancer* cell line *in vitro* primary screen[a]

**Cell line**	**9h** GI_{50} (µM)	**9q** GI_{50} (µM)	**9s** GI_{50} (µM)	**10p** GI_{50} (µM)	**11h** GI_{50} (µM)	**11r** GI_{50} (µM)	**15a** GI_{50} (µM)	**15b** GI_{50} (µM)
NCI code	762037	762032	762042	762033	762039	762044	775044	775045
Melanoma								
LOX IMVI	0.0388	<0.0100	0.0325	0.0339	0.0327	0.0344	0.0426	0.328
MALME-3M	>100	20.5	[c]Nd	0.0306	>100	[c]Nd	30.1	>100
M14	0.221	0.0173	0.0248	0.0176	0.0194	0.0223	0.123	0.291
MDA-MB-435	0.294	<0.0100	0.0260	0.0205	0.0191	0.0221	0.0259	0.0738
SK-MEL-2	18.6	0.0446	0.0676	0.301	3.25	[c]Nd	0.075	0.5
SK-MEL-28	[c]Nd	64.3	>100	76.2	16.0	>100	0.0831	0.389
SK-MEL-5	0.0243	0.0124	0.0232	0.0249	0.0260	0.0222	0.0406	0.164
UACC-257	[c]Nd	[c]Nd	>100	[c]Nd	11.2	[c]Nd	>100	>100
UACC-62	0.0392	<0.0100	0.0406	0.0320	0.0267	0.0396	0.0555	0.131
Ovarian cancer								
IGROV1	0.0499	0.0322	0.0357	0.0448	0.0507	0.0466	0.0628	0.434
OVCAR-3	0.0254	0.0207	0.0228	0.0233	0.0252	0.0262	0.0624	0.27
OVCAR-4	0.0391	[c]<0.0100	0.0407	0.0397	0.0373	0.0388	0.0739	0.282
OVCAR-5	0.0983	0.0494	0.0813	0.0618	0.0948	0.0937	0.535	2.34
OVCAR-8	0.0373	<0.0100	0.0308	0.0340	0.0374	0.0384	0.0506	0.3
NCI/ADR-RES	0.0213	<0.0100	0.0185	0.0206	0.0173	0.0196	0.0365	0.212
SK-OV-3	0.0381	0.0304	0.0317	0.0366	0.0373	0.0356	0.0791	0.416
Renal cancer								

786-0	0.0442	0.0268	0.0376	0.0255	0.0231	0.0327	0.39	0.439
A498	0.0243	<0.0100	0.0234	0.0175	0.0183	0.0223	0.0324	0.104
ACHN	0.0643	0.0213	0.0620	0.0532	0.0607	0.0614	0.0771	0.359
CAKI-1	0.0831	0.0308	0.0491	0.0440	0.0589	0.0631	0.0805	0.32
RXF 393	0.0375	0.0428	0.0396	0.0410	0.0301	0.0457	0.123	0.282
SN12C	0.0503	0.0370	0.0629	0.0298	0.0508	0.0591	0.192	0.463
TK-10	0.0965	0.0534	0.0649	0.0802	0.0617	0.0748	43.1	0.695
UO-31	0.505	0.0878	0.0740	0.0858	0.216	0.190	0.182	0.538
Breast cancer								
MCF-7	0.0368	<0.0100	0.0324	0.0353	0.0335	0.0336	0.0513	0.244
MDA-MB-231/ATCC	0.0322	0.0168	0.0315	0.0240	0.0296	0.0315	0.0534	0.215
HS 578T	0.0391	0.0106	0.0409	0.0517	0.0247	0.0530	0.0631	0.173
BT-549	0.0329	0.0110	0.0378	0.0188	0.0203	0.0253	0.0596	0.411
T-47D	26.5	>100	>100	63.5	56.2	>100	>100	>100
MDA-MB-468	0.0250	<0.0100	0.0216	0.0250	0.0233	0.0267	0.131	0.273

[a] NCI *in vitro* human tumour cell screen 5 dose assay for compounds **9h (S-762037), 9q (S-762032), 9s (S-762042), 10p (D-762033), 11h (D-762039), 11r (D-762044), 15a (S-775044)** and **15b (S-775045)**; The compounds were evaluated using five different concentrations (100 μM, 10 μM, 1 μM, 0.1 μM and 0.01 μM) over the NCI 60 cell line panel and incubations were carried out over 48 h exposures to the drug; [b]GI_{50} is the molar concentration of the compound causing 50% inhibition of growth of the tumour cells; [c]Nd: Not determined.

Table S9: Standard COMPARE analysis of β-lactam **9q**

Rank	Compound	r
	Based on GI_{50} mean graph	
1	Vincristine sulfate (hiConc = 10^{-3} M)	0.585
2	Vincristine sulfate (hiConc = 10^{-5} M)	0.522
3	Maytansine	0.514
4	Glycoxalic acid	0.481
5	Vinblastine sulfate (hiConc = 10^{-4} M)	0.465
	Based on TGI mean graph	
1	Vincristine sulfate (hiConc = 10^{-3} M)	0.664
2	Vinblastine sulfate (hiConc = $10^{-5.6}$ M)	0.636
3	Maytansine	0.602
4	Rhizoxin (hiConc = 10^{-4} M)	0.568
5	Rhizoxin (hiConc = 10^{-9} M)	0.555
	Based on LC_{50} mean graph	
1	*Tetraplatin*	0.896
2	*5-FUDR*	0.862
3	*Didemnin B*	0.853
4	Maytansine	0.826
5	B-TGDR	0.785

Table S10: Standard COMPARE analysis of β-lactam **9s**

Rank	Compound	r
	Based on GI_{50} mean graph	
1	Vincristine sulfate (hiConc = 10^{-5} M)	0.604
2	Maytansine	0.561
3	Vinblastine sulfate (hiConc = 10^{-4} M)	0.520
4	Vincristine sulfate (hiConc = 10^{-3} M)	0.520
5	Tiazofurin	0.484
	Based on TGI mean graph	
1	Vinblastine sulfate (hiConc = $10^{-5.6}$ M)	0.632
2	Maytansine	0.566
3	Vinblastine sulfate (hiConc = 10^{-4} M)	0.562
4	Vincristine sulfate (hiConc = 10^{-3} M)	0.538
5	A-TGDR	0.481
	Based on LC_{50} mean graph	
1	*Tetraplatin*	0.885
2	*B-TGDR*	0.880
3	*Didemnin B*	0.861
4	*Paclitaxel (Taxol)*	0.768
5	Maytansine	0.741

The target set was the standard agent database and the target set endpoints were selected to be equal to the seed end points. Standard COMPARE analysis was performed. Correlation values (r) are Pearson correlation coefficients. Vincristine sulfate appears at different concentrations as it has been tested by the NCI at multiple concentration ranges.

Table S11: Docking scores for selected β-lactams

Compound ID	Docking Score[a]
**17d**	-9.83
**11p**	-9.65
**9q**	-9.60
**11h**	-9.43
**9s**	-9.42
**9h**	-9.41
**10p**	-9.39
**10h**	-9.36
**11r**	-9.20
**10r**	-9.04

[a]Scores from docking with MOE 2022 using the MMFF94x force field of the best ranked pose of each compound

Table S12. X-Ray data and torsional angles for compounds **9h, 10h, 10f, 11a-c, 11h**

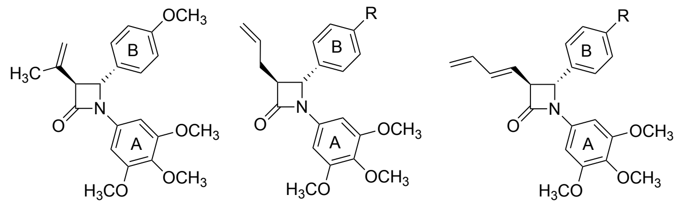

9h **10h, 10f** **11a-c, 11h**

Structure	Ring plane normal AB angle(°)	Ring A to β-lactam Torsion (°)[a]	Ring B to β-lactam Torsion (°)[b]	Ring AB Torsion (°)[c]	Ring B Vinyl Torsion (°)[d]
**9h**[e]	89.46(4)	-174.38(12)	-130.95(12)	61.87(16)	-117.26(12)
	96.83(4)	174.99(12)	131.59(12)	-58.22(16)	118.05(13)
**10h**[e], R=OCH_3	81.71(9)	-176.5(3)	-134.4(2)	58.9(3)	-125.8(3)
	80.88(8)	178.1(3)	138.9(2)	-57.0(3)	127.0(8)
**10f,** R=H	93.69(2)	177.33(3)	140.971(2)	-65.140(6)	123.91(5)
**11a,** R=F	84.75(6)	-174.62(16)	-131.95(18)	67.3(2)	-130.69(15)
**11b,** R=Cl	87.39(5)	-177.57(13)	-131.77(13)	64.78(18)	-128.27(11)
**11c,** R=Br	91.56(7)	-178.68(19)	-132.3(2)	64.0(3)	-127.03(19)
**11h,** R=OCH_3	76.78(3)	167.43(1)	132.97(2)	-55.57(4)	127.50(3)

[a]C14-C13-N1-C2; [b]C10-C5-C4-N1; [c]C13-N1-C4-C5; [d]C5-C4-C3-C26; only the first atom numbering scheme is outlined above and all measurements follow the same scheme; [e]2 independent molecules in the asymmetric unit

Table S13. X-ray crystal structure of compounds **8h**, **8i** and **11t** [a, b].

**8h**	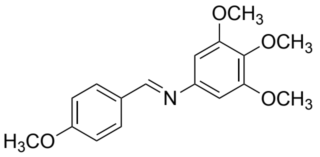	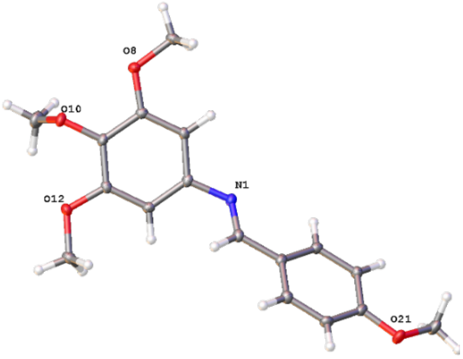
**8i**	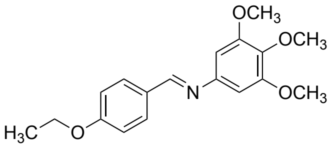	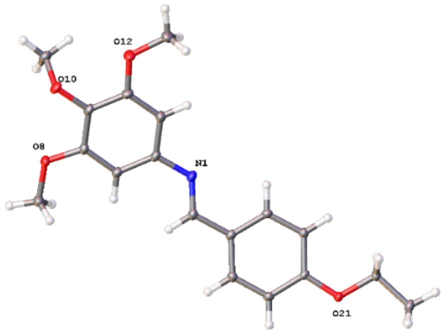
**11t**	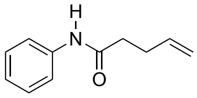	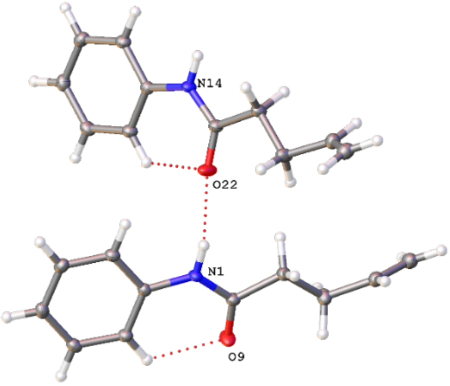

[a] X-ray crystal structure of compounds **8h**, **8i** and **11t** with the thermal ellipsoids set at 50% probability. [b]Crystallographic data deposited with the Cambridge Crystallographic Data Centre (CCDC) 2241430 (**8h**), 2241431 (**8i**), 2241438 (**11t**).

Table S14. X-ray crystal structure of compounds **10f, 11b, 11c, 11d.**

10f

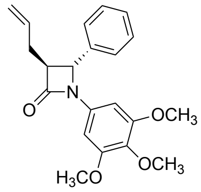

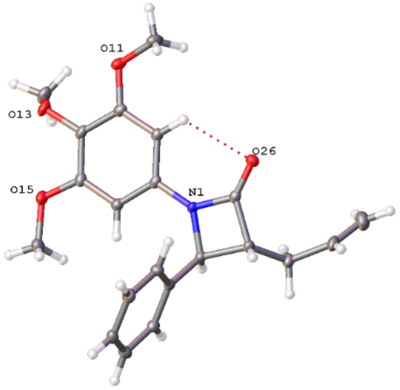

11b

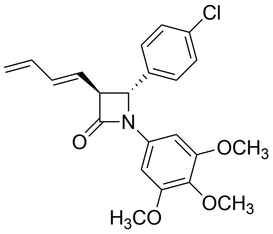

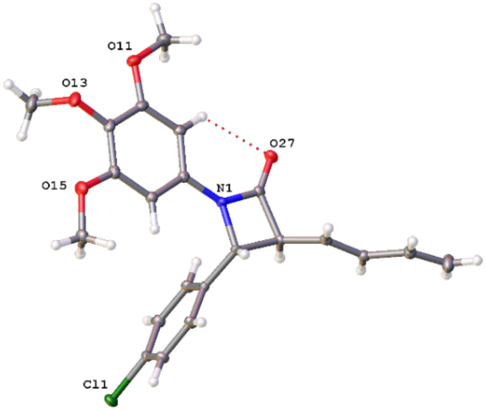

11c

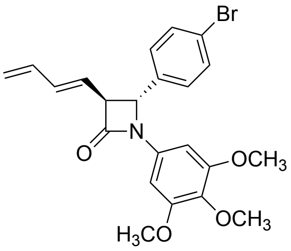

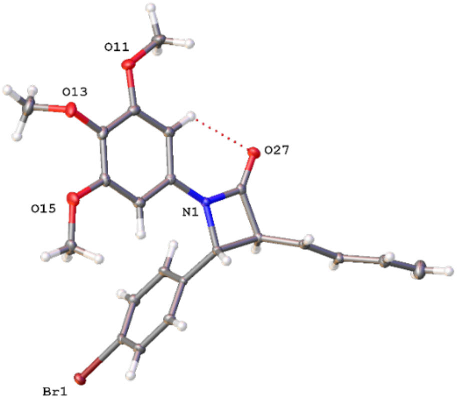

**11d**	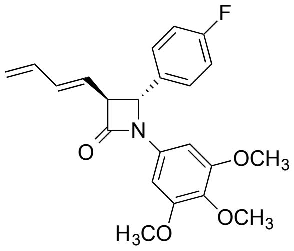	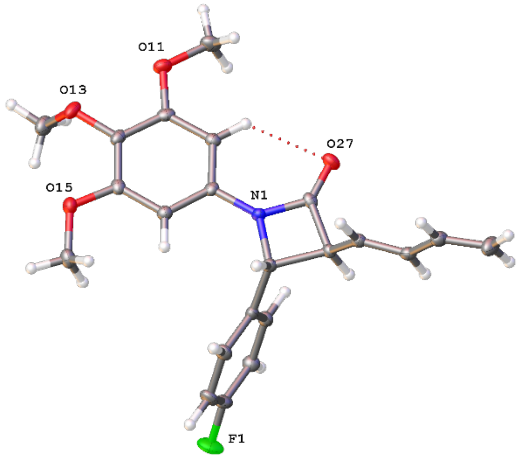

[a] X-ray crystal structure of compounds **10f, 11b, 11c** and **11d** with the thermal ellipsoids set at 50% probability.
[b]Crystallographic data deposited with the Cambridge Crystallographic Data Centre (CCDC) 2241430 (**10f**), 2241435 (**11b**), 2241436 (**11c**), and 2241434 (**11d**).

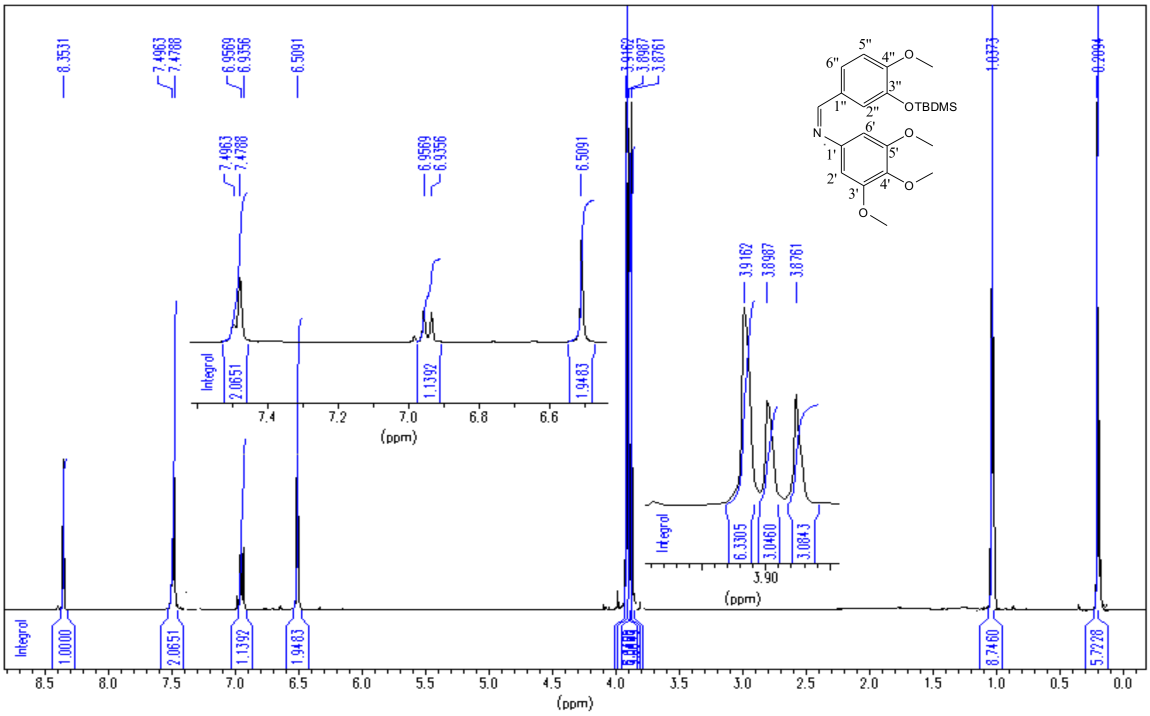

Figure S1: ^{1}H NMR spectrum for compound **8p**

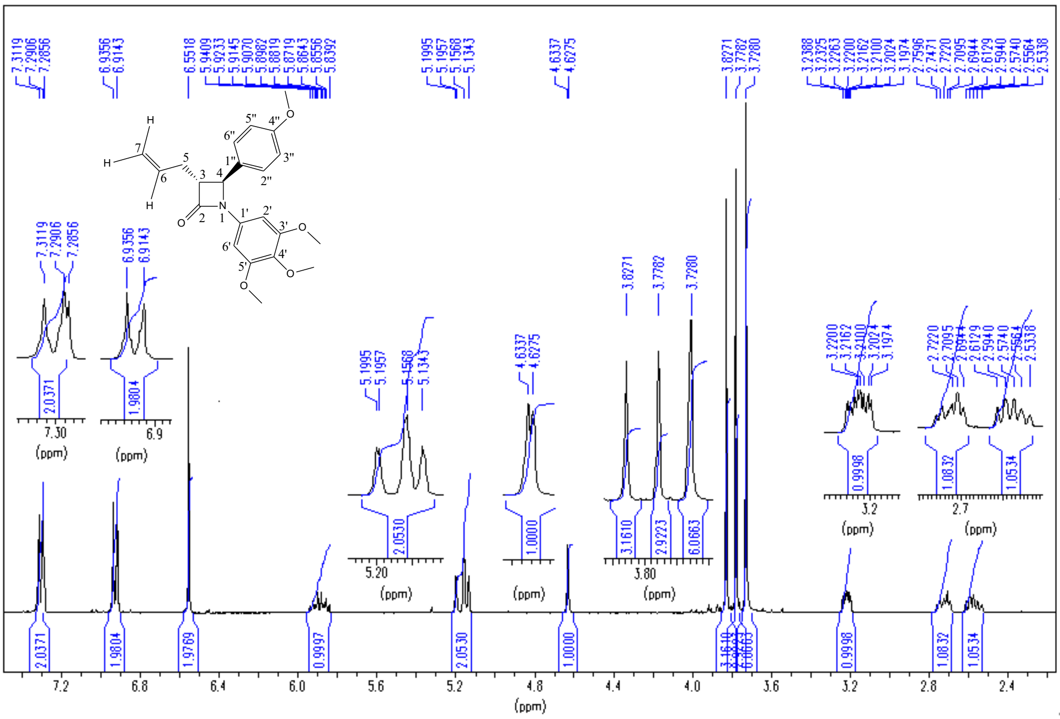

Figure S2: ^{1}H NMR spectrum of β-lactam **10h**

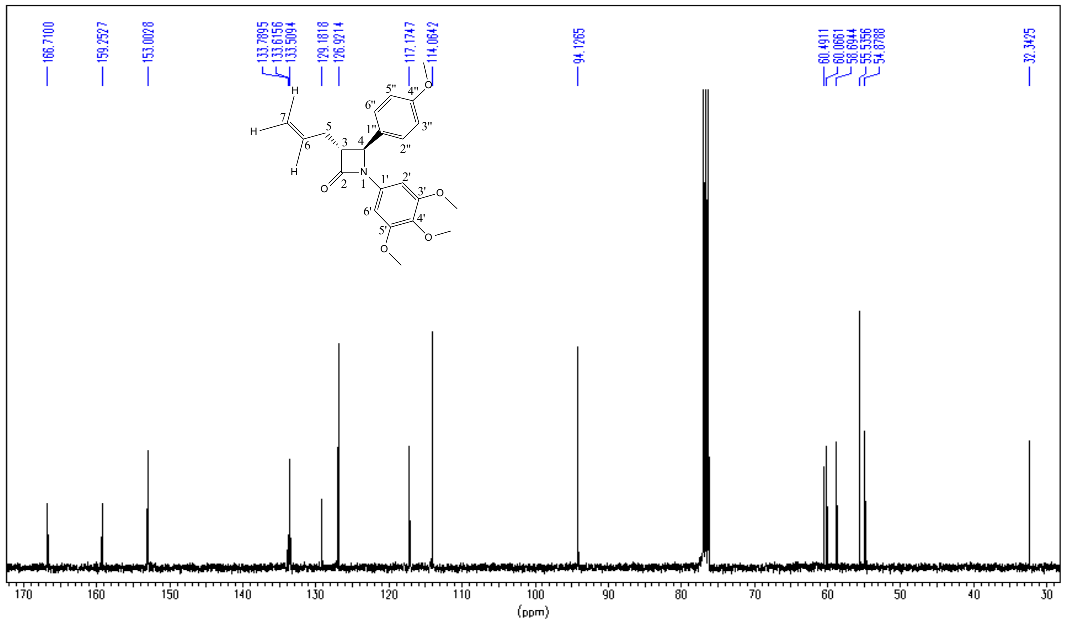

Figure S3: ^{13}C NMR spectrum of β-lactam **10h**

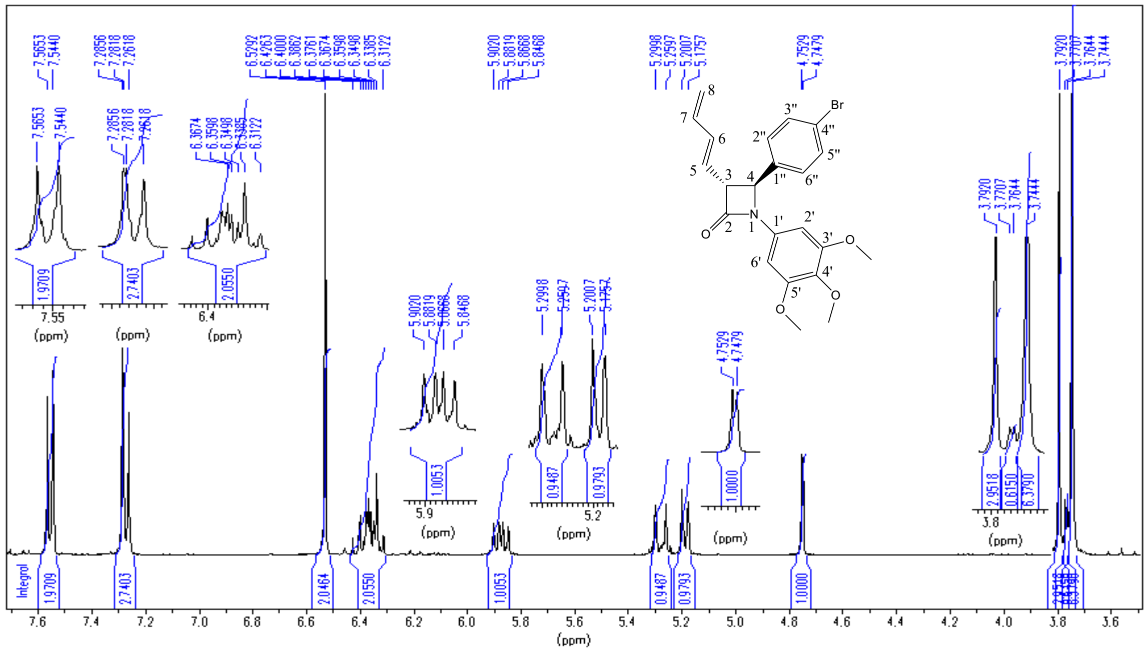

Figure S4: ^{1}H NMR spectrum of β-lactam **11c**

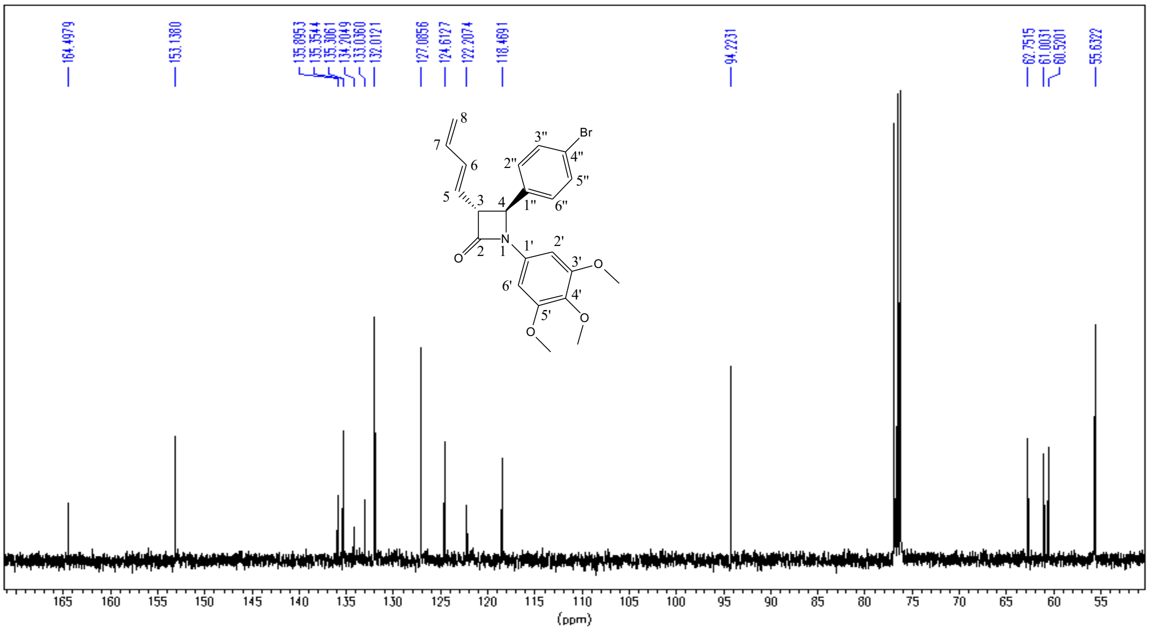

Figure S5: ^{13}C NMR spectrum of β-lactam **11c**

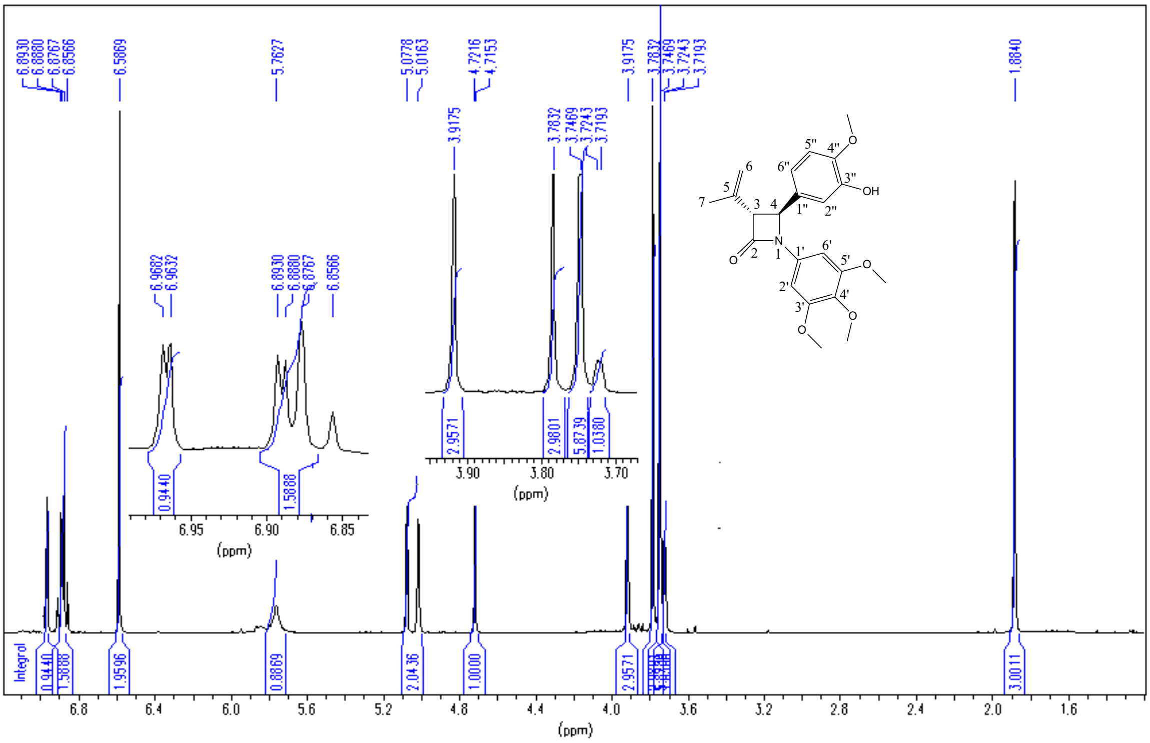

Figure S6: ^{1}H NMR spectrum of compound **9q**

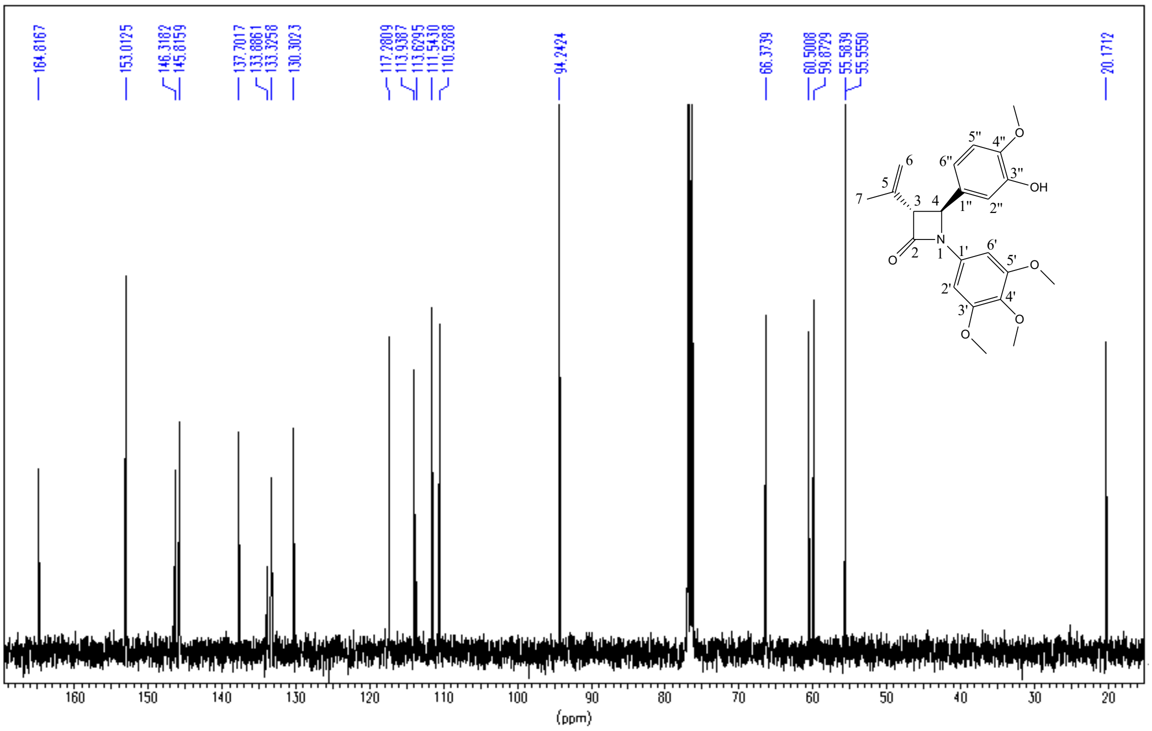

Figure S7: ^{13}C NMR spectrum of compound **9q**

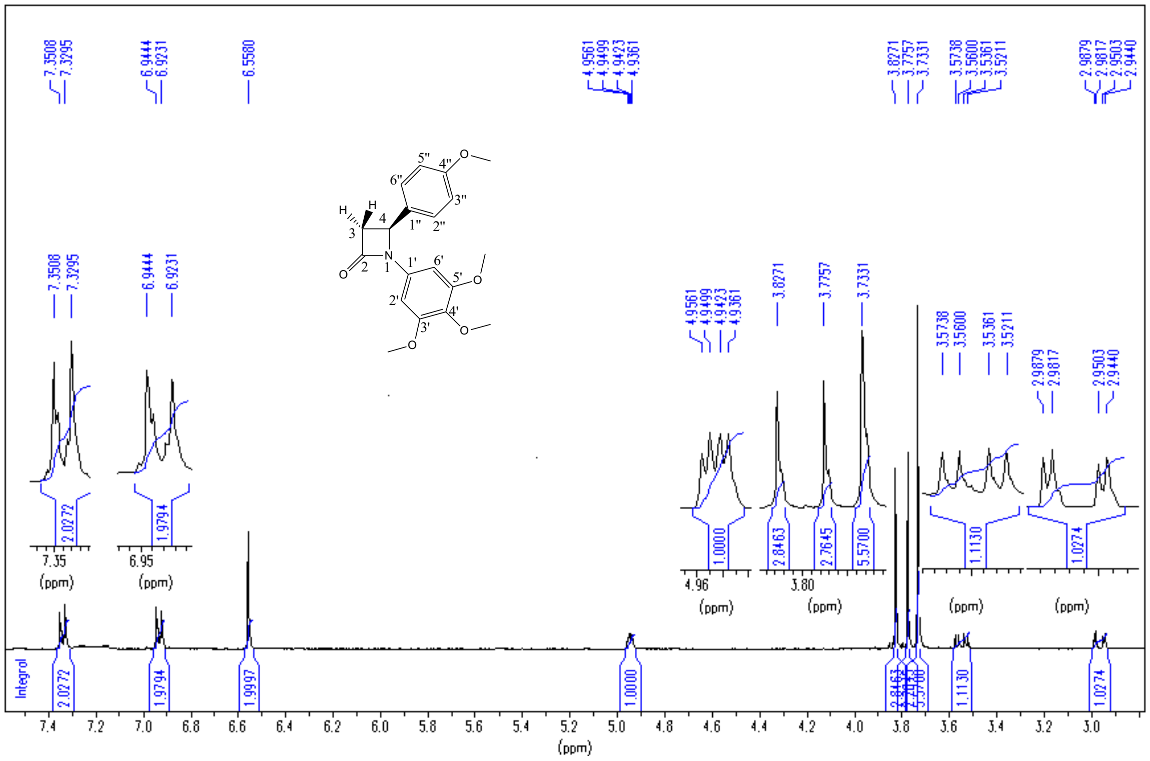

Figure S8: ^{1}H NMR spectrum of compound **16a**

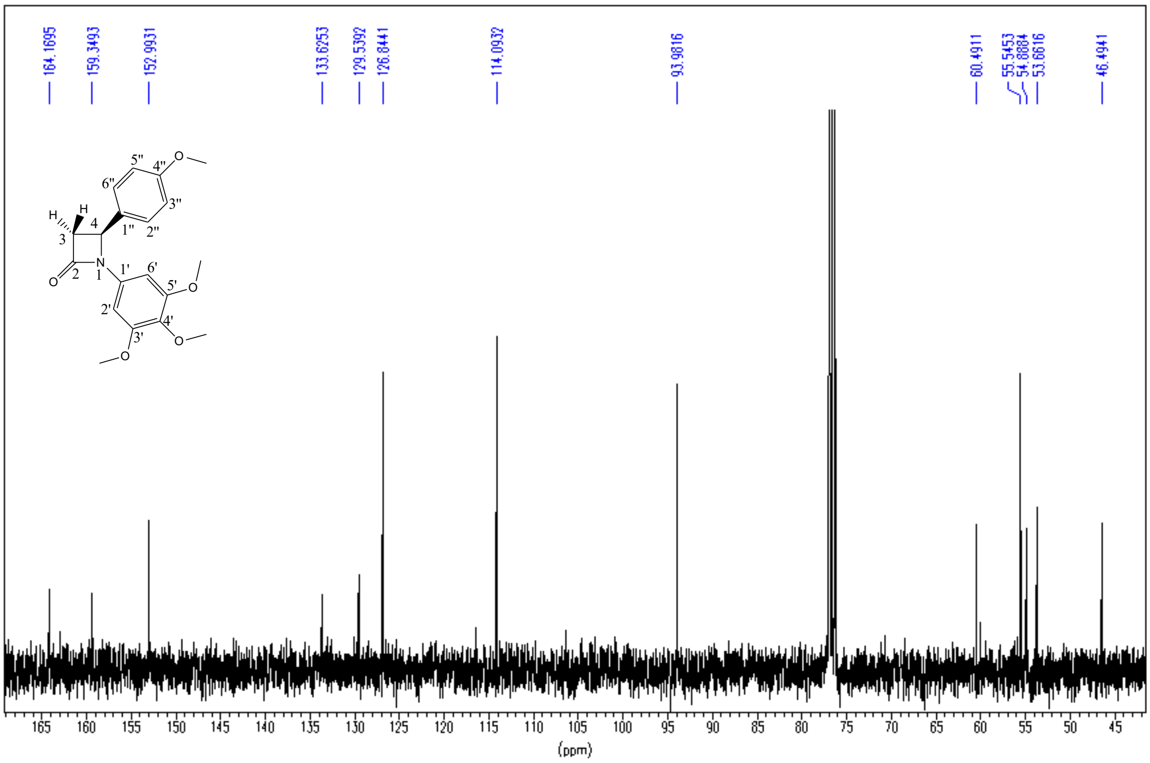

Figure S9: ^{13}C NMR spectrum of compound **16a**

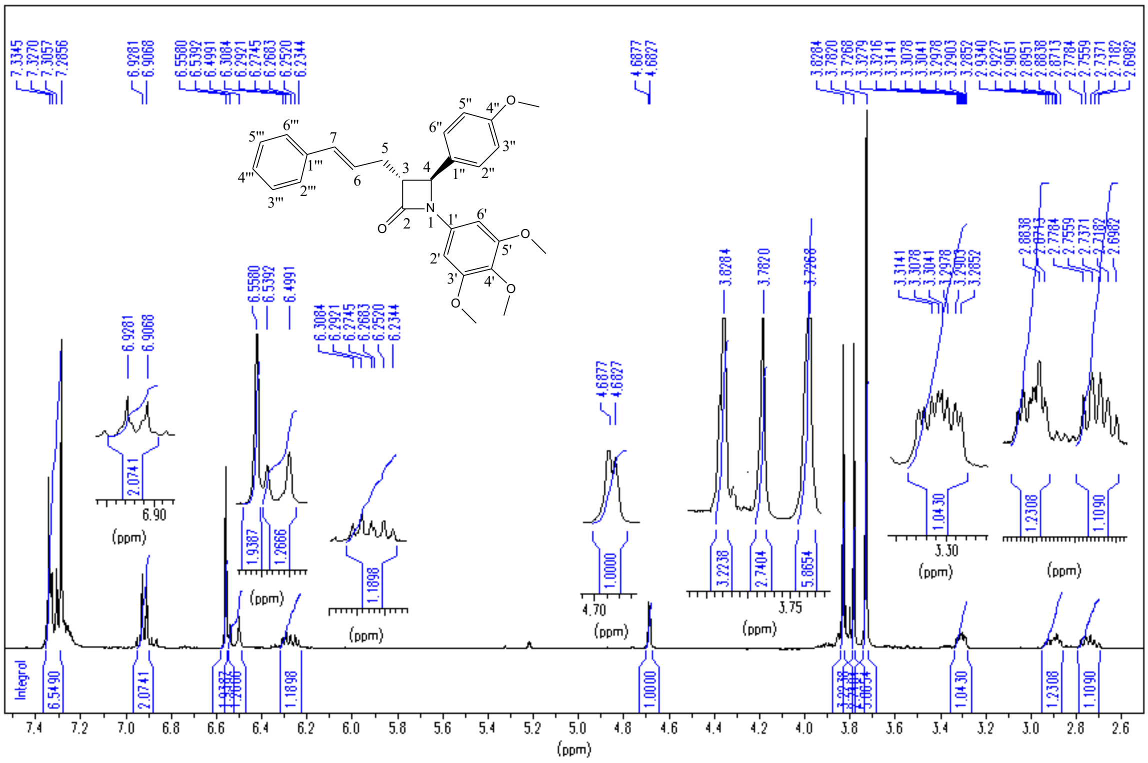

Figure S10: ^{1}H NMR spectrum of compound **17a**

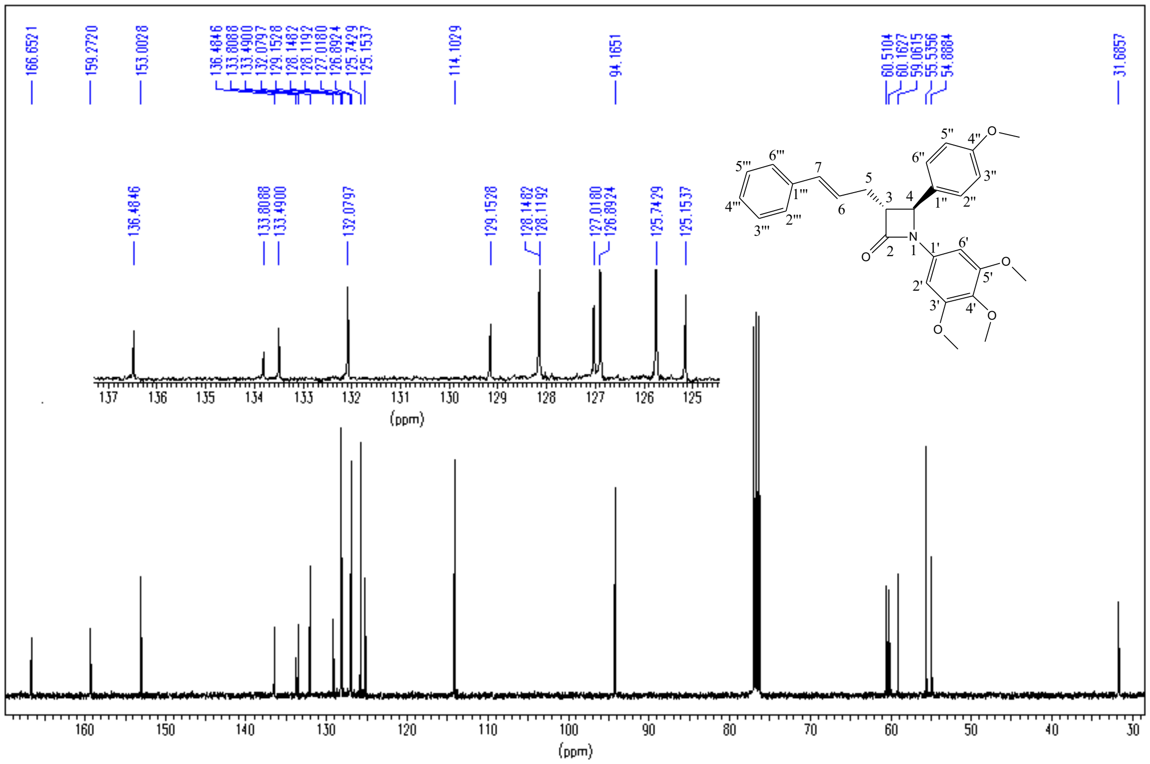

Figure S11: ^{13}C NMR spectrum of compound **17a**

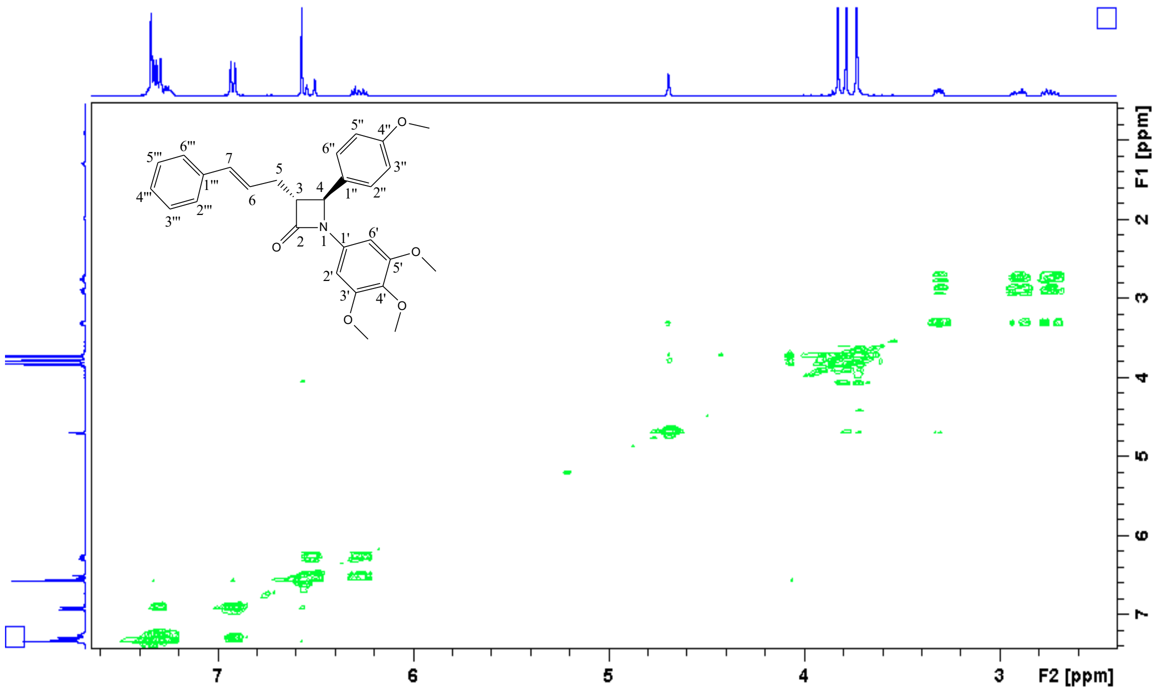

Figure S12: HH COSY spectrum for compound **17a**

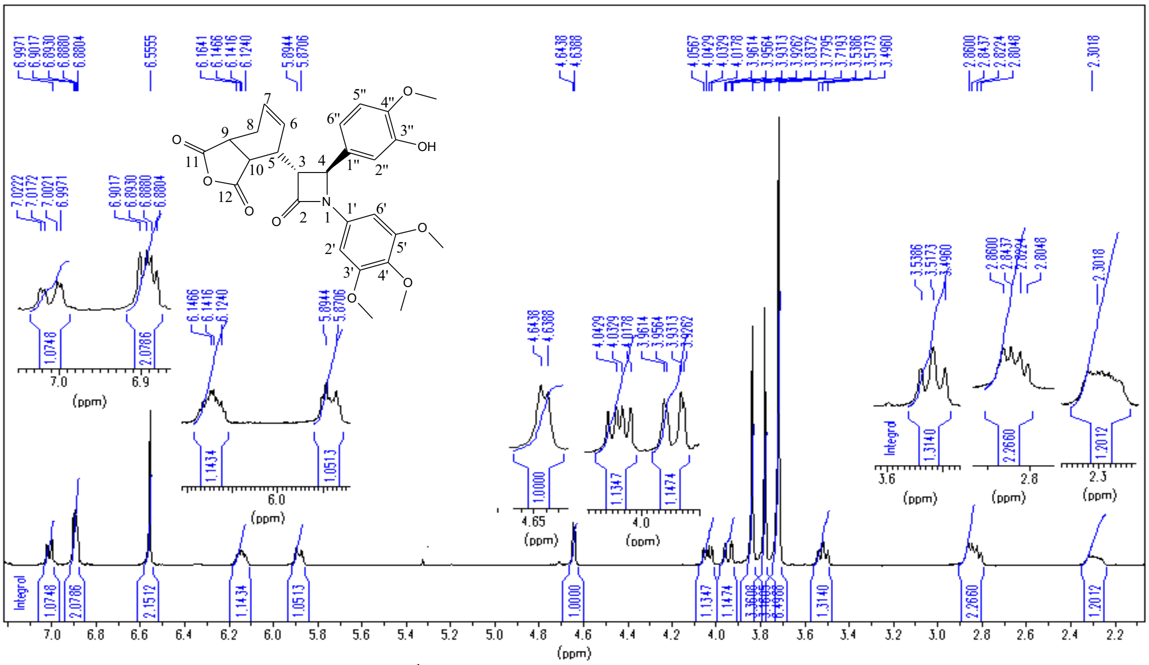

Figure S13: ^{1}H NMR spectrum for compound **22b**

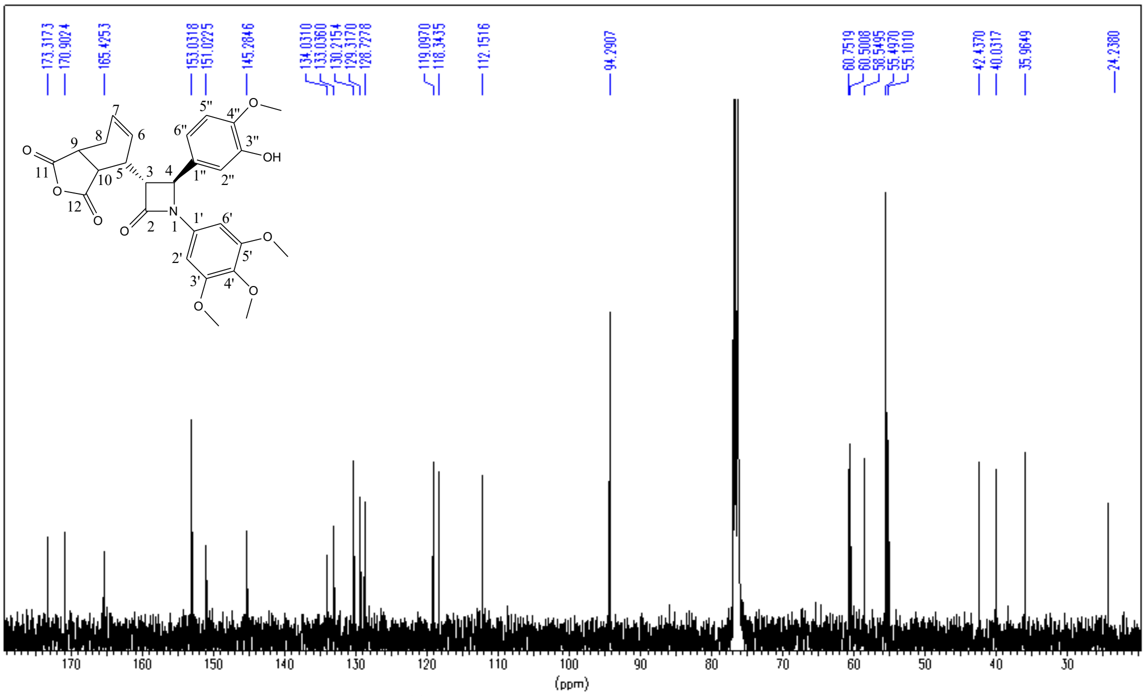

Figure S14: ^{13}C NMR spectrum for compound **22b**

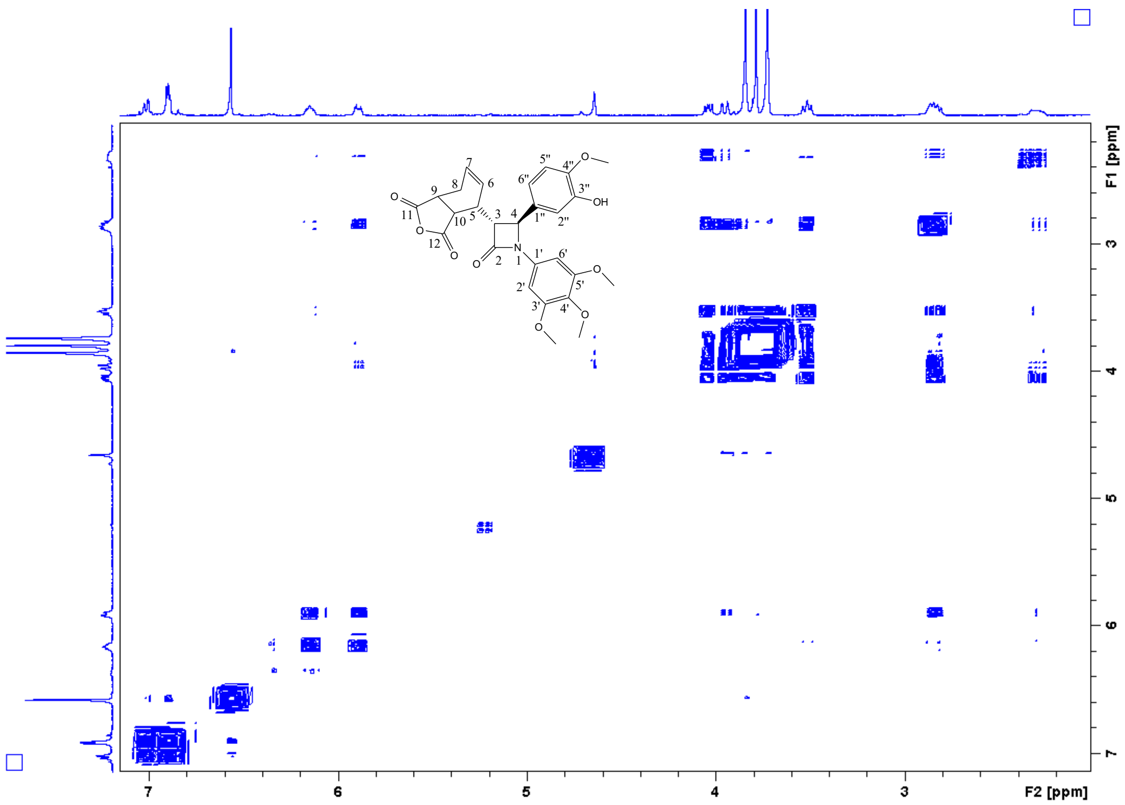

Figure S15: ^{1}H-^{1}H COSY for compound **22b**

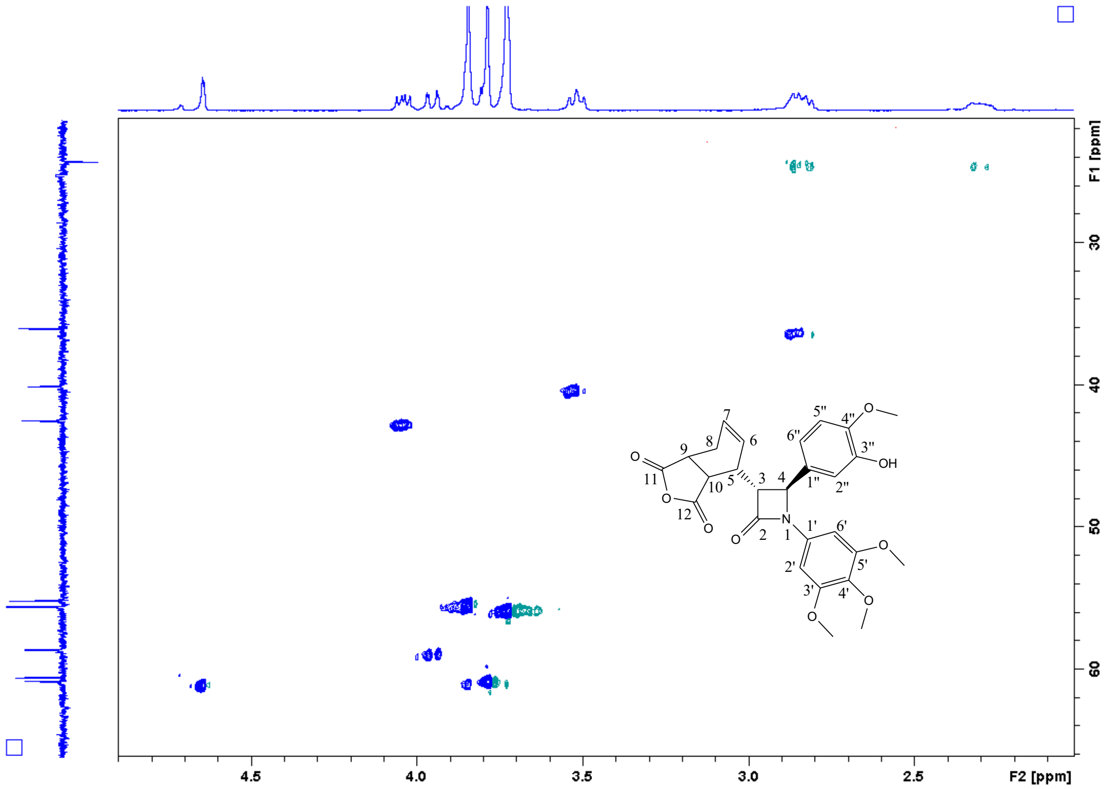

Figure S16: ^{1}H-^{13}C COSY for compound **22b**

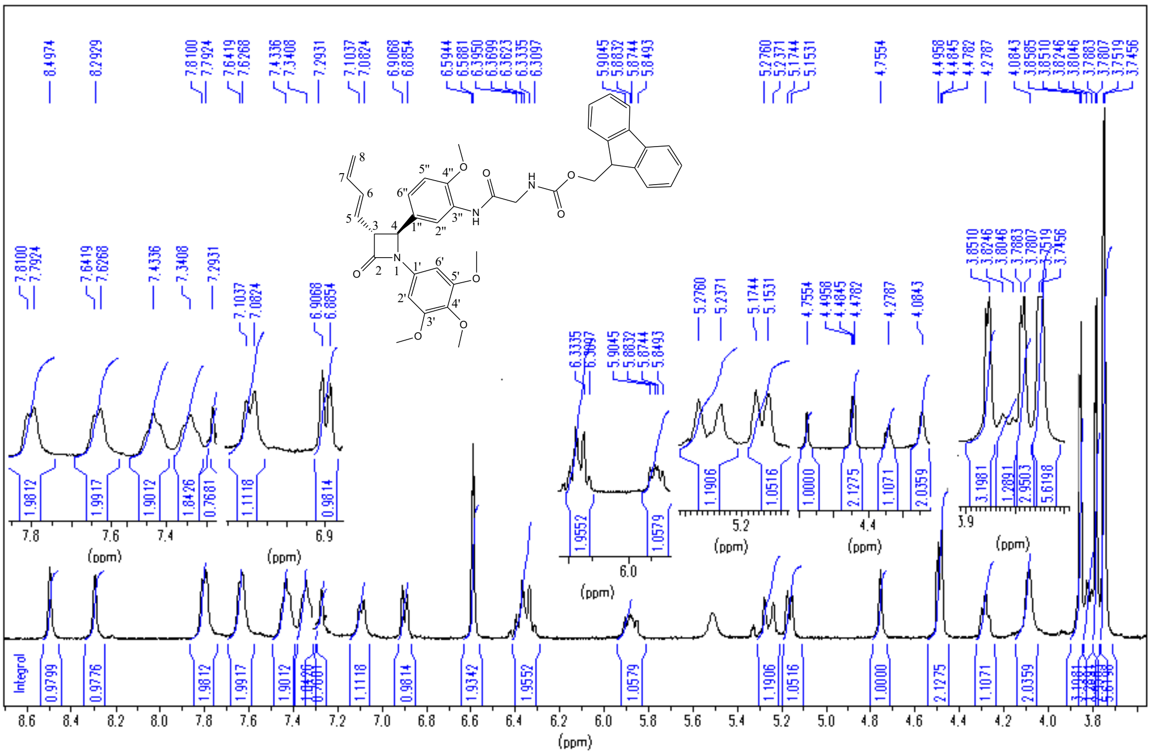

Figure S17: ^{1}H NMR spectrum of amino acid Fmoc prodrug **12e**

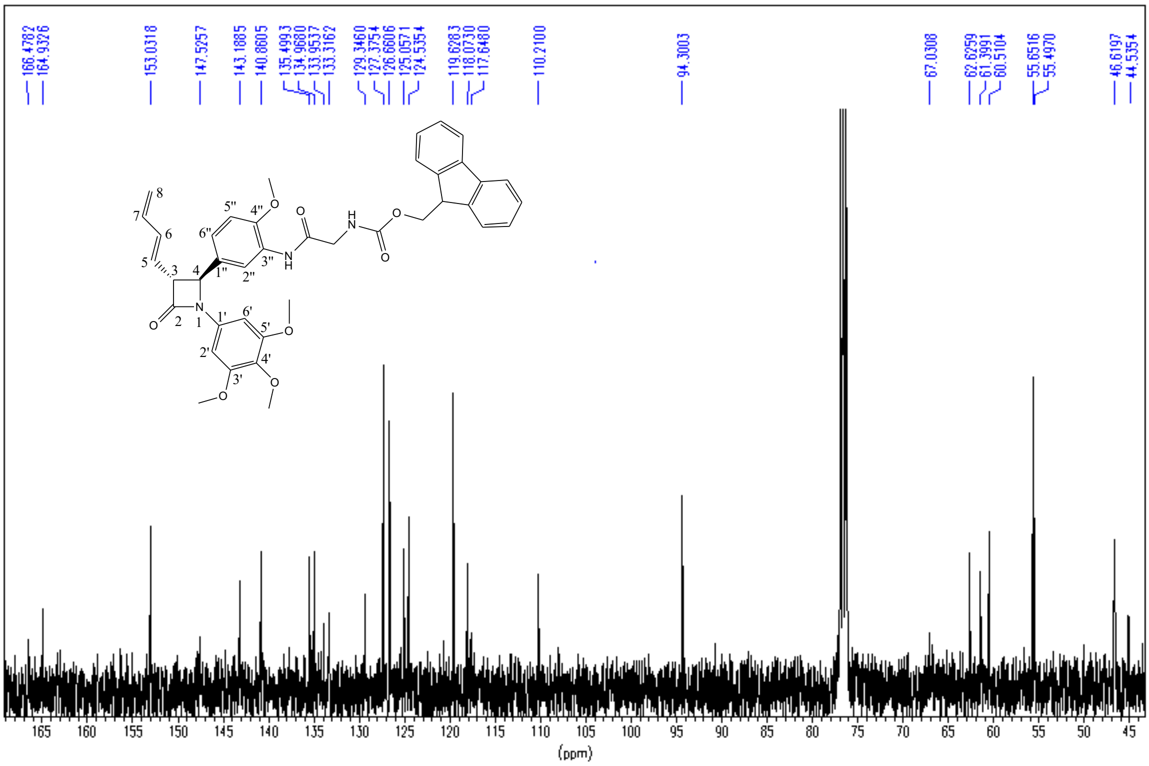

Figure S18: ^{13}C NMR spectrum of amino acid Fmoc prodrug **12e**

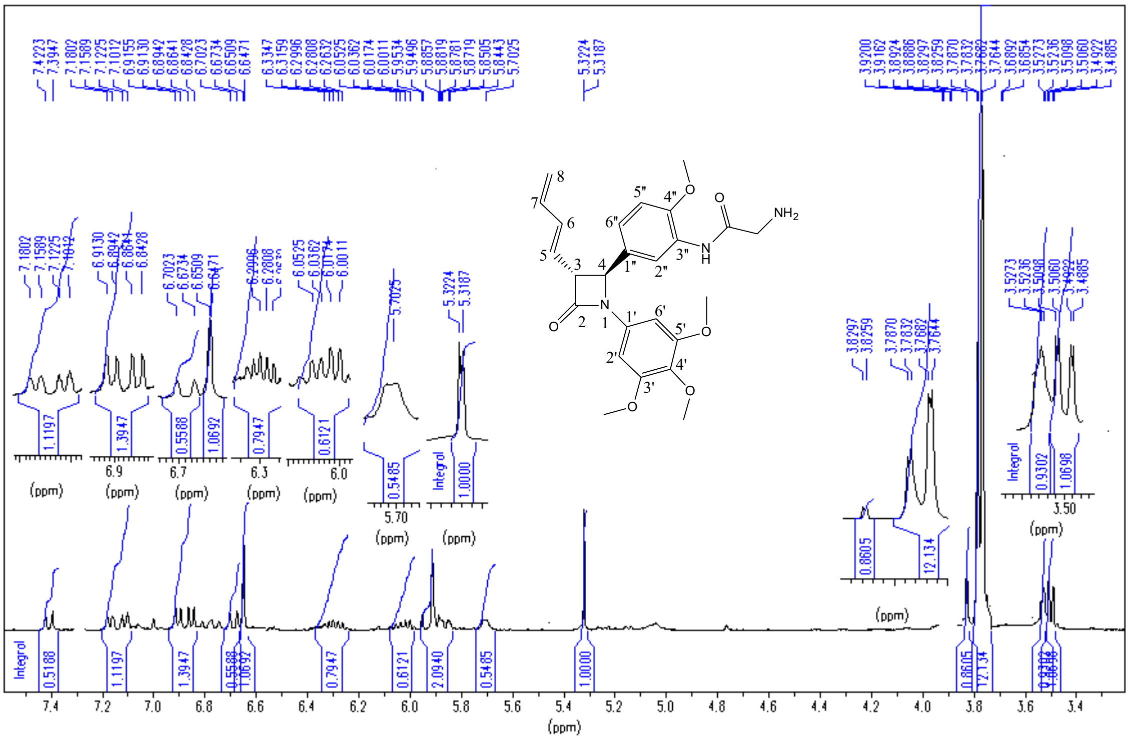

Figure S19: ^{1}H NMR spectrum collected for amino acid prodrug **13e**

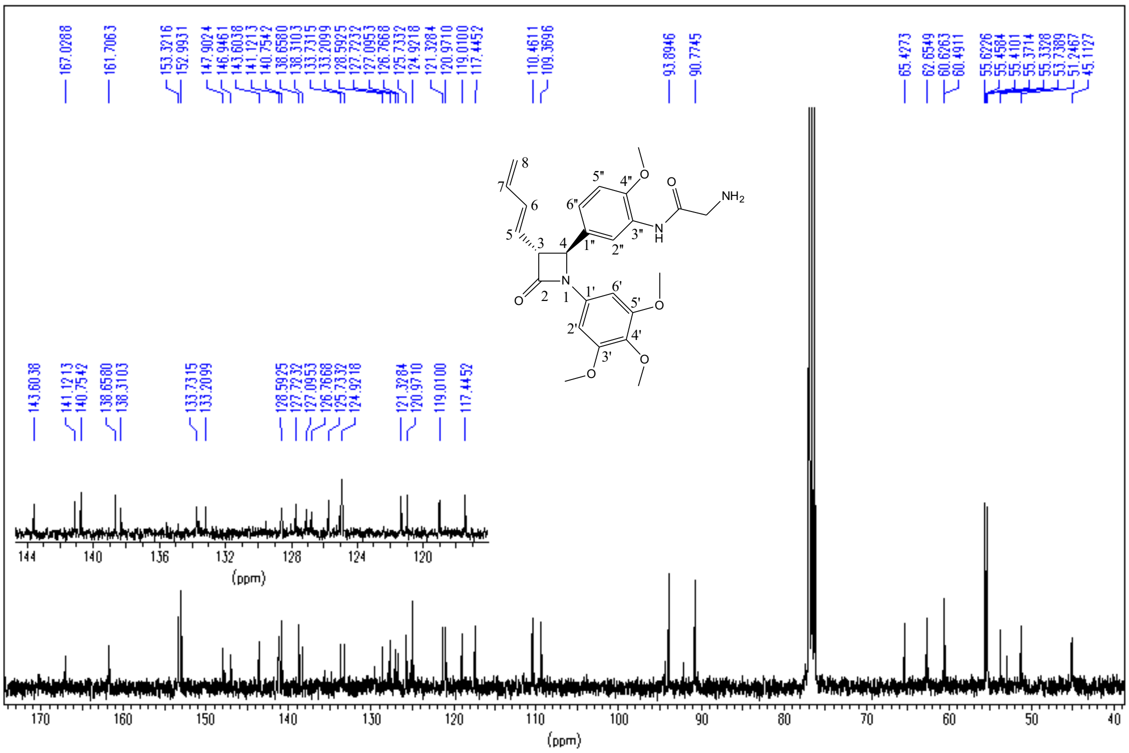

Figure S20: ^{13}C NMR spectrum collected for amino acid prodrug **13e**

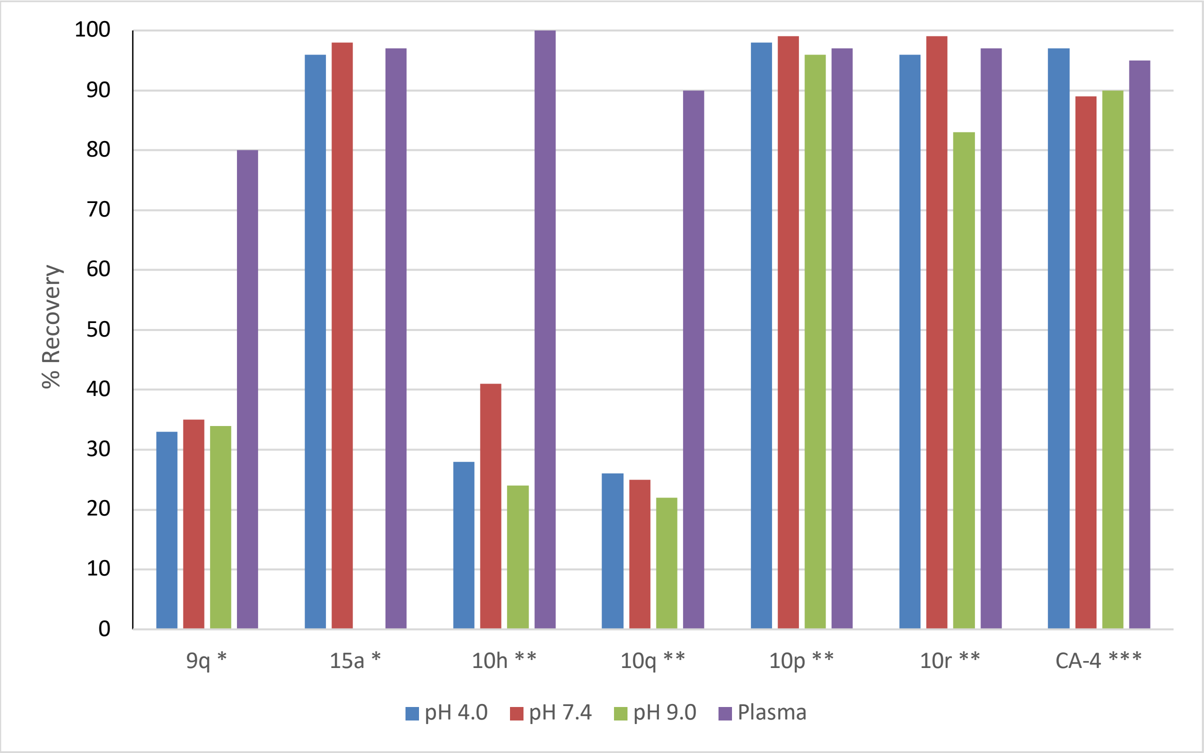

Figure S21: Stability study for compounds **9q, 15a, 10h, 10q, 10p, 10r** and **CA-4** at pH 4.0, pH 7.5, pH 9.0 and in plasma.

All samples were analysed using acetonitrile (80%):water (20%) as the mobile phase over 10 min and a flow rate of 1 mL/min. Stock solutions are prepared by dissolving 5 mg of compounds in 10 mL of mobile phase. [* Stability study over 24 h, * Stability study over 264 h , *** Stability study over 7 h] Precipitation was observed immediately when the prodrug **15a** was in contact with pH 9.0 buffer solution and compound **15a** was not detected at its retention time (5.2 minutes). Data presented represents the results of single experiments.

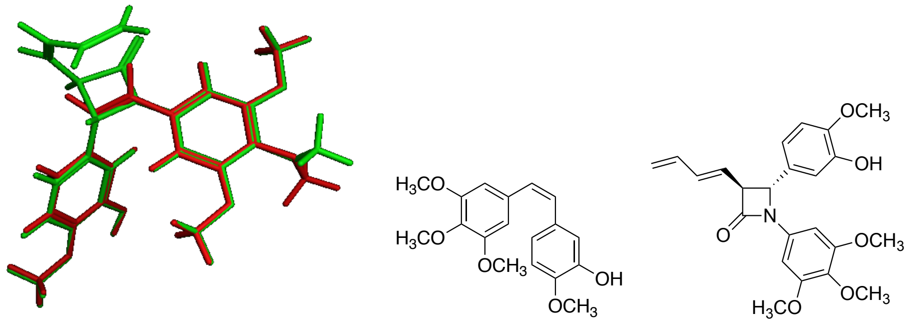

CA-4 **11p**

Figure S22: Flexible alignment of **11p** [green] and combretastatin A-4 [red]; 2D structures are shown for comparison

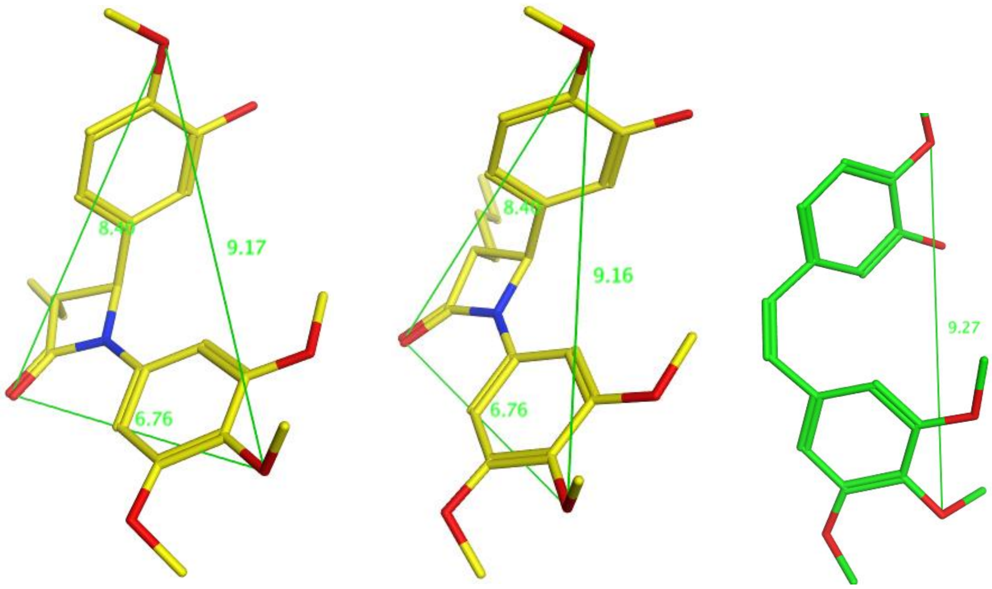

25 **11p** **CA-4**

Figure S23: Inter-atomic distances between oxygen atoms for compounds **25, 11p** and CA-4 (calculated using MOE 2011.10)

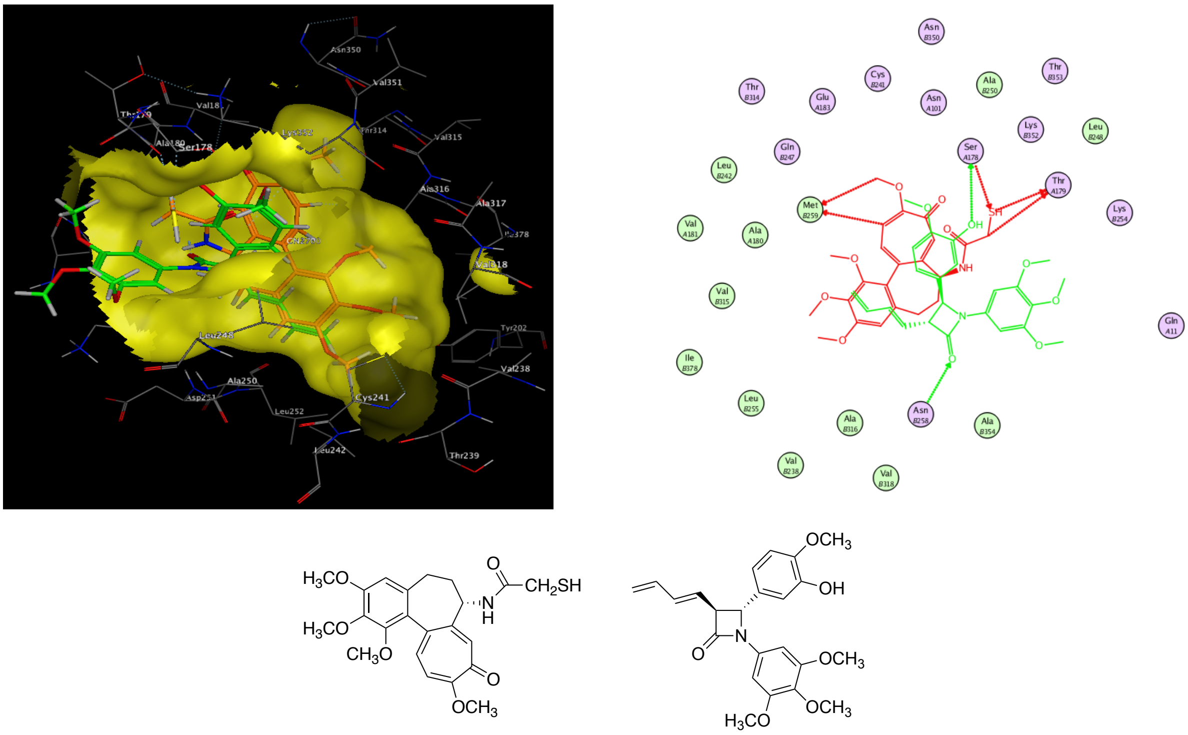

1b DAMA Colchicine

11p

Figure S24: Protein ligand interactions for β-Lactam **11p** docked in the colchicine-binding site of tubulin

(A) β-Lactam **11p** docked in the colchicine-binding site of tubulin (PDB entry 1SA0). Orange=colchicine; green=**11p**; **(B)** 2D representation of the binding interactions of **11p** and DAMA-colchicine **1b** with the colchicine binding site of tubulin

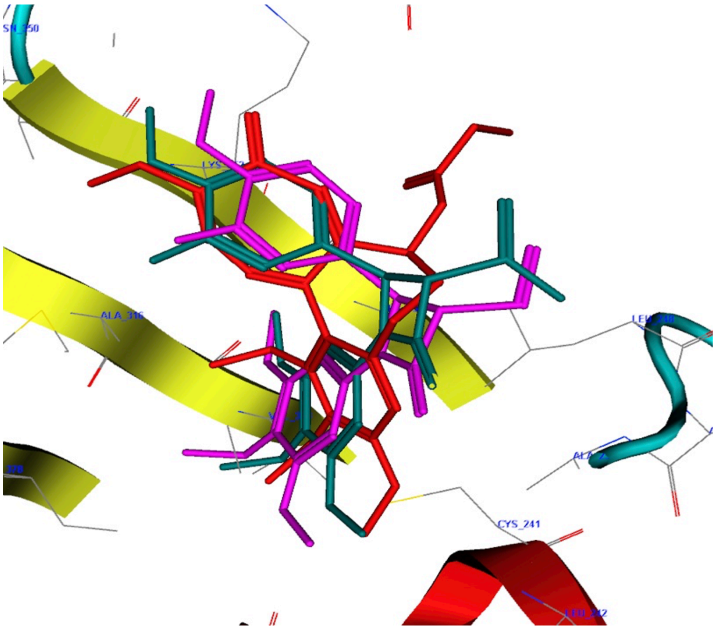

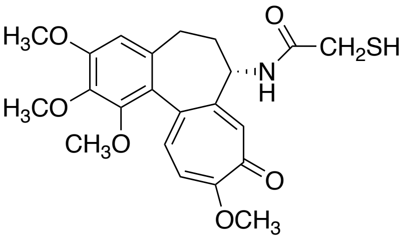

1b DAMA-Colchicine

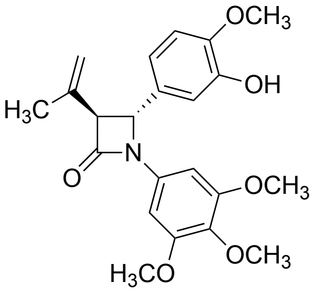

9q

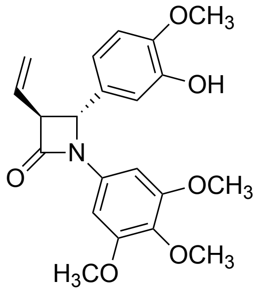

Figure S25: Overlay of the X-ray structure of tubulin crystallised with DAMA Colchicine (PDB entry 1SA0) **(1b)** (red) on docked solution of β-lactam **9q** (green) and a related 3-vinyl β-lactam (pink)

**(A)** 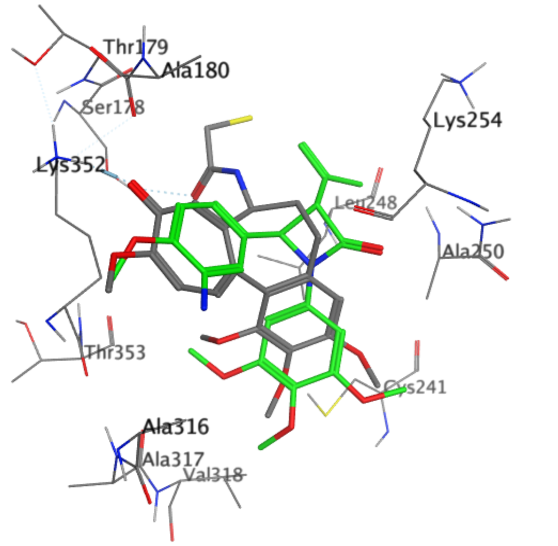	*(3S,4R)*-**9s** 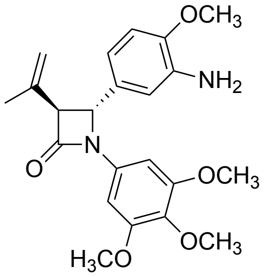
**(B)** 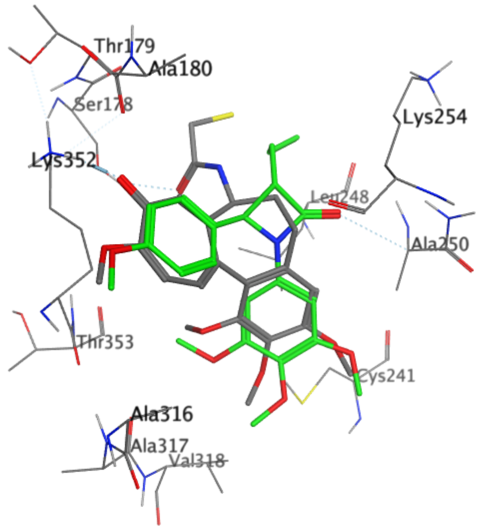	*(3S,4R)*-**10h** 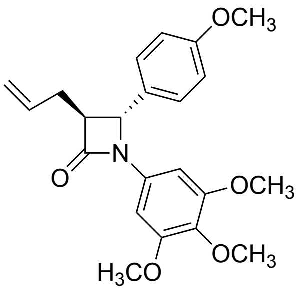
**(C)** 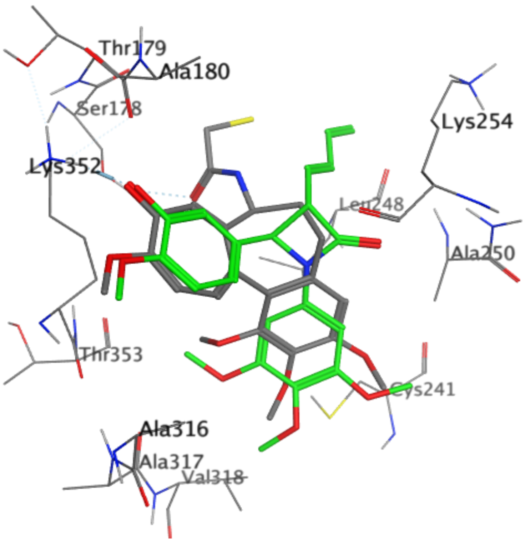	*(3S,4R)*-**11p** 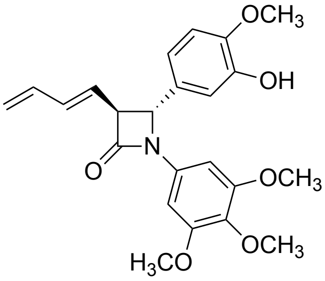

**(D)** 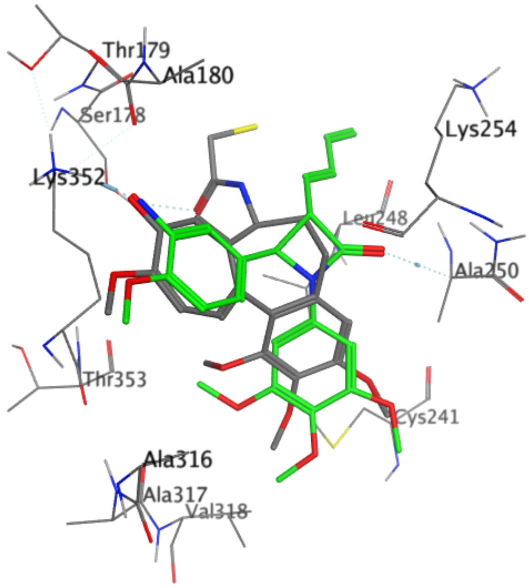	*(3S,4R)*-**11r** 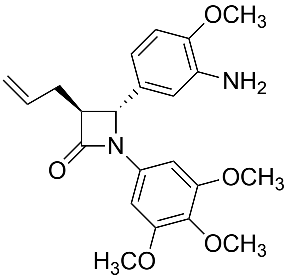

Figure S26. Overlay of the X-ray structure of tubulin co-crystallised with DAMA-colchicine (PDB entry 1SA0) on the best ranked docked pose of the 3*S,4R* enantiomer **9s, 10h, 11p** and **11r,** panels **A-D** respectively.

Ligands are rendered as tube and amino acids as line. Tubulin amino acids and DAMA-colchicine are coloured by atom type: carbon = grey, hydrogen = white, oxygen = red, nitrogen = blue. The β-lactam is depicted with a green backbone. The atoms are coloured by element type, Key amino acid residues are labelled, and multiple residues are hidden to enable a clearer view.
