# Supplementary figures and images for: Antiproliferative and Tubulin-Destabilising Effects of 3-(Prop-1-en-2-yl)azetidin-2-Ones and Related Compounds in MCF-7 and MDA-MB-231 Breast Cancer Cells (part 2 of 2)
Source: Pharmaceuticals (Basel). 2023 Jul 13;16(7):1000. doi: 10.3390/ph16071000 (PMC10385824; doi:10.3390/ph16071000)

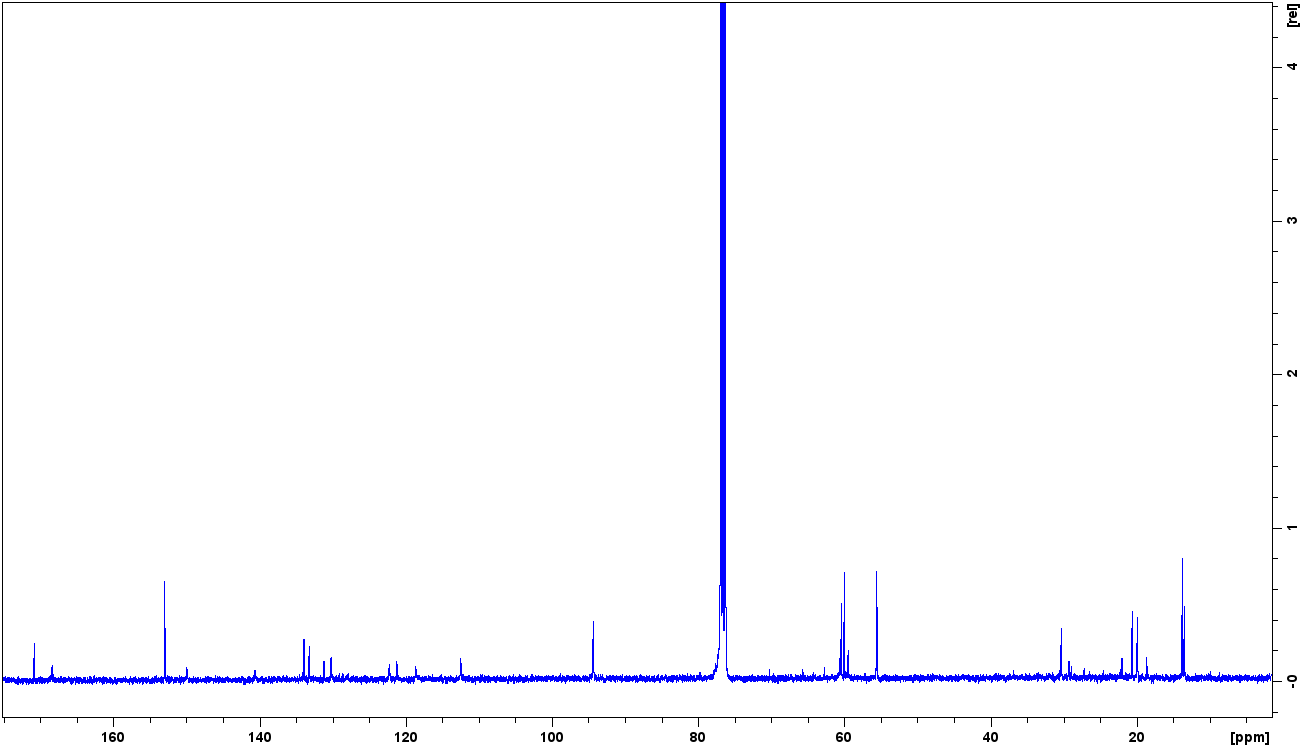

Supplement: Supplementary file 1 [file pharmaceuticals-16-01000-s001.zip › 15d mjm18018_13c.png]

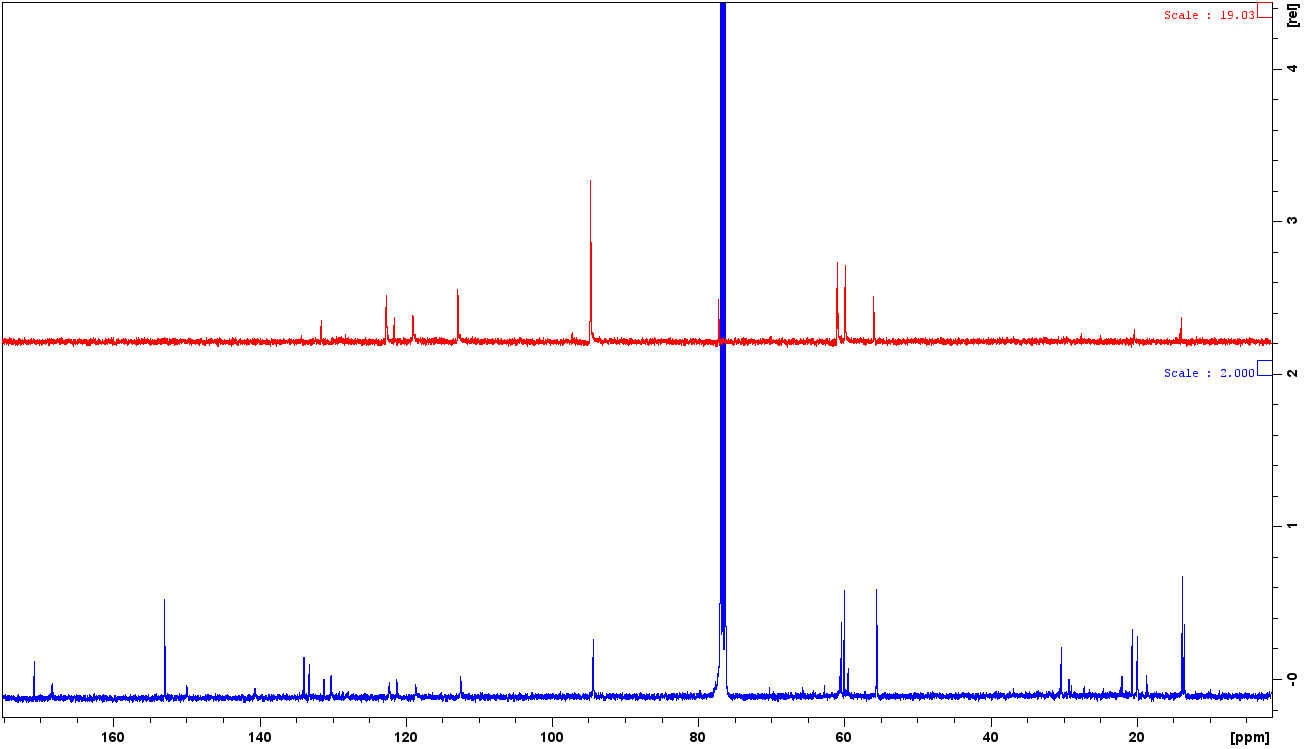

Supplement: Supplementary file 1 [file pharmaceuticals-16-01000-s001.zip › 15d mjm18018_13c_DEPT.png]

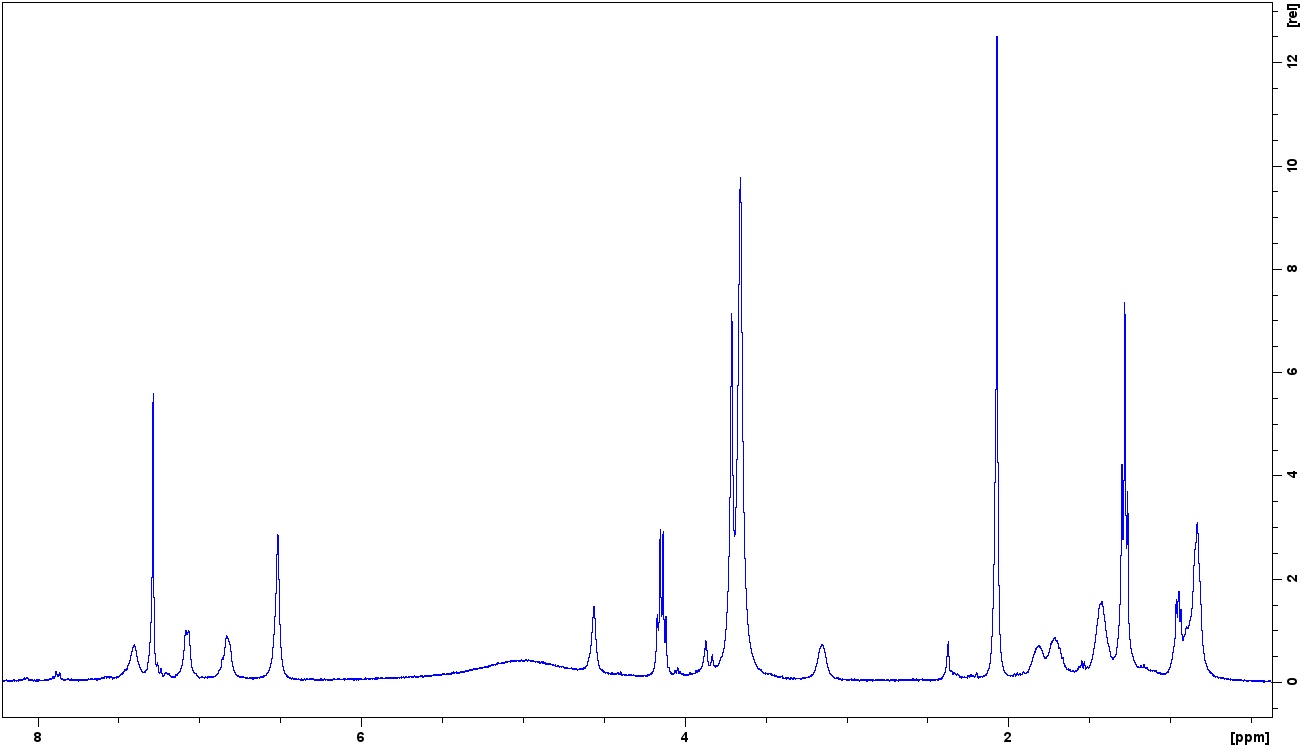

Supplement: Supplementary file 1 [file pharmaceuticals-16-01000-s001.zip › 15d mjm18018_1h.png]

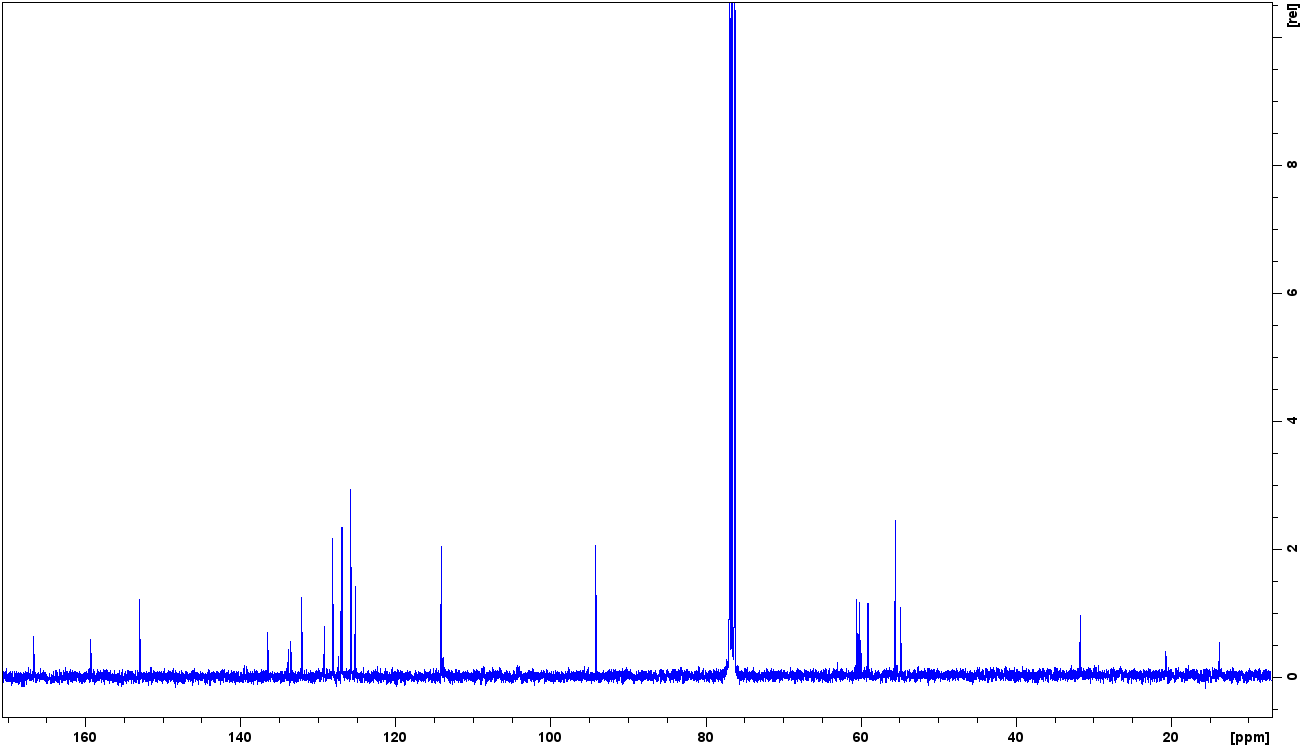

Supplement: Supplementary file 1 [file pharmaceuticals-16-01000-s001.zip › 17a mjm16843_13c.png]

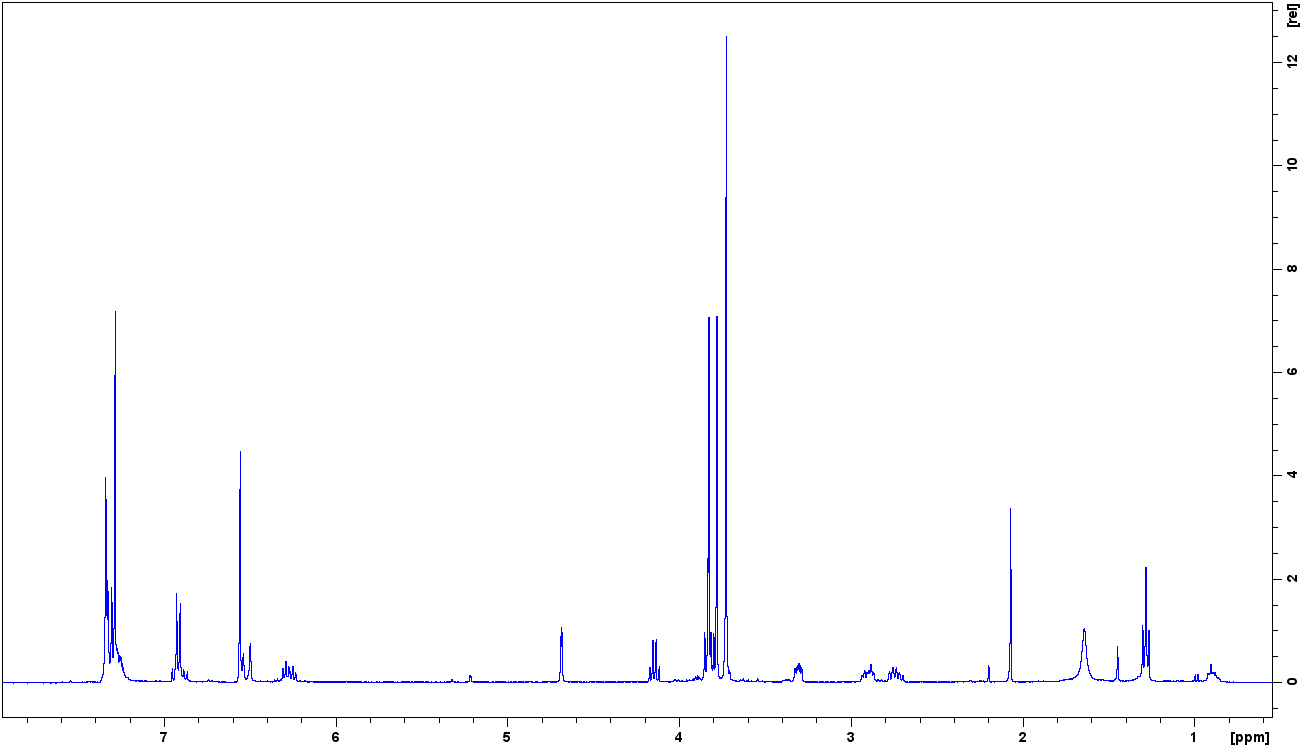

Supplement: Supplementary file 1 [file pharmaceuticals-16-01000-s001.zip › 17a mjm16843_1h.png]

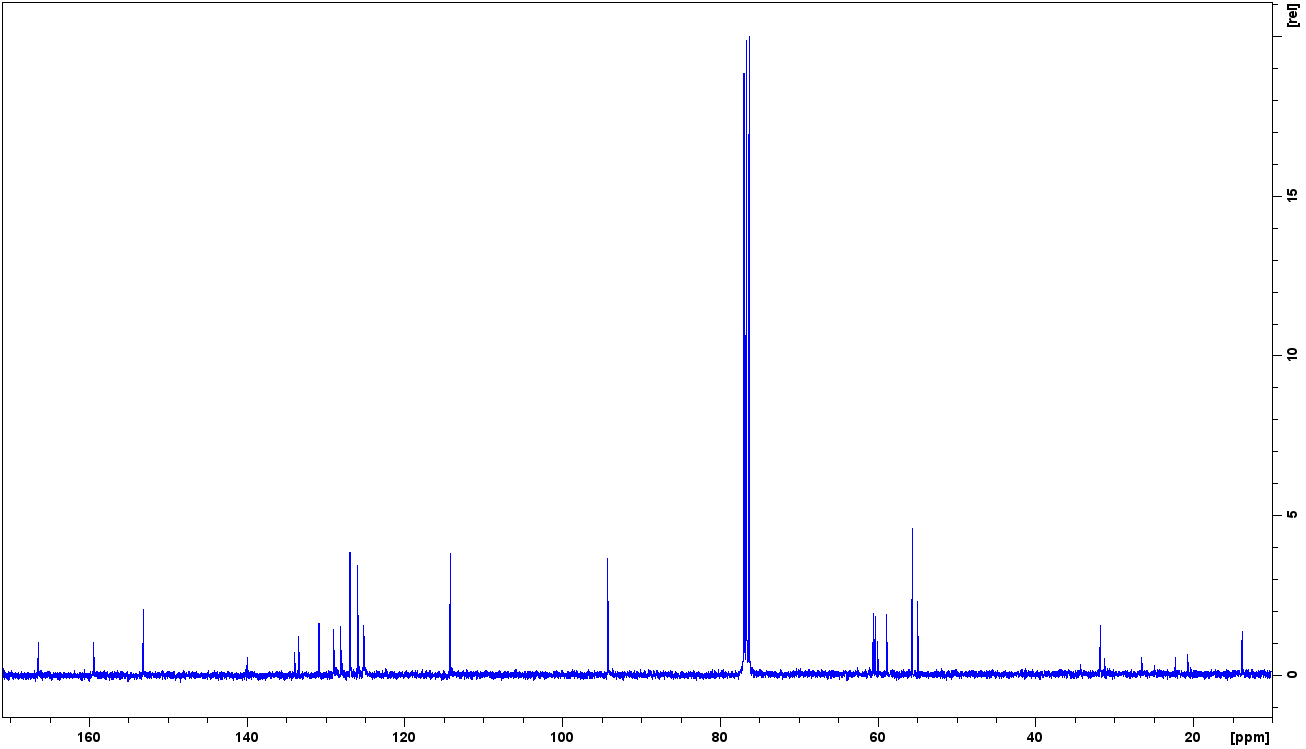

Supplement: Supplementary file 1 [file pharmaceuticals-16-01000-s001.zip › 17d mjm16728_13c.png]

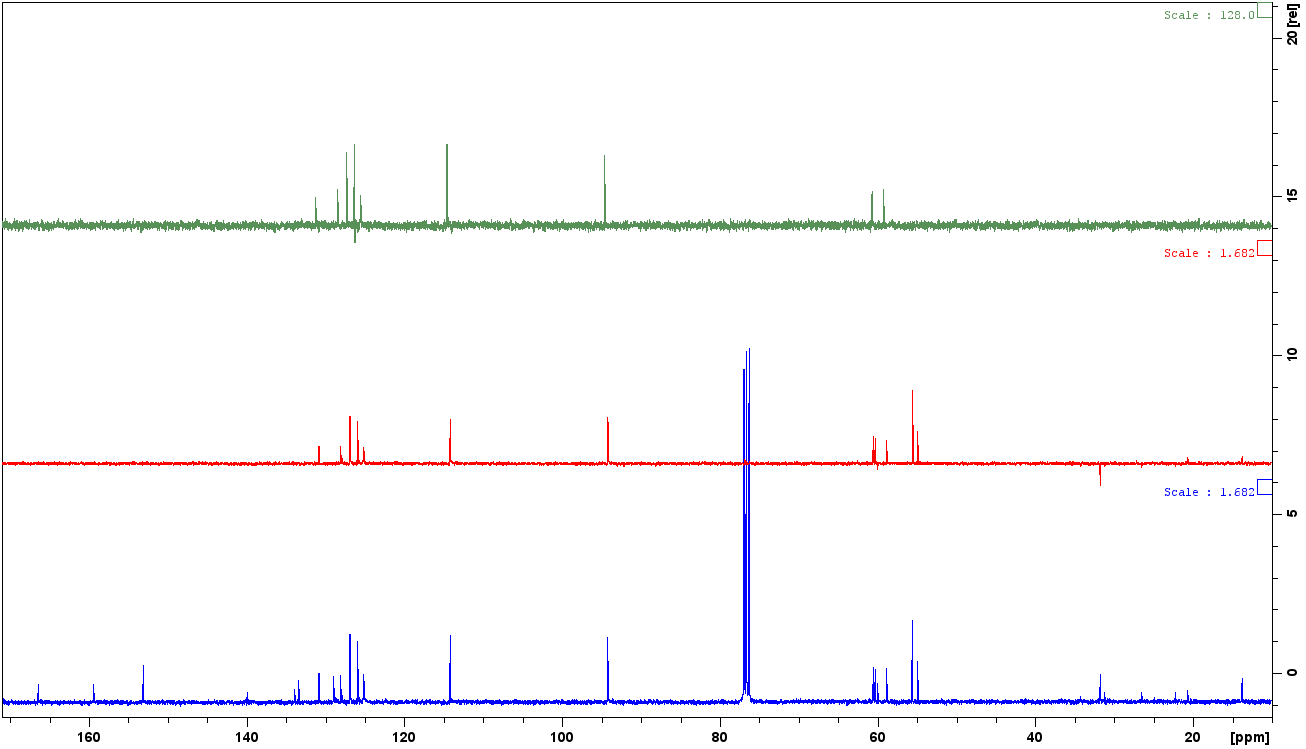

Supplement: Supplementary file 1 [file pharmaceuticals-16-01000-s001.zip › 17d mjm16728_13c_DEPTs.png]

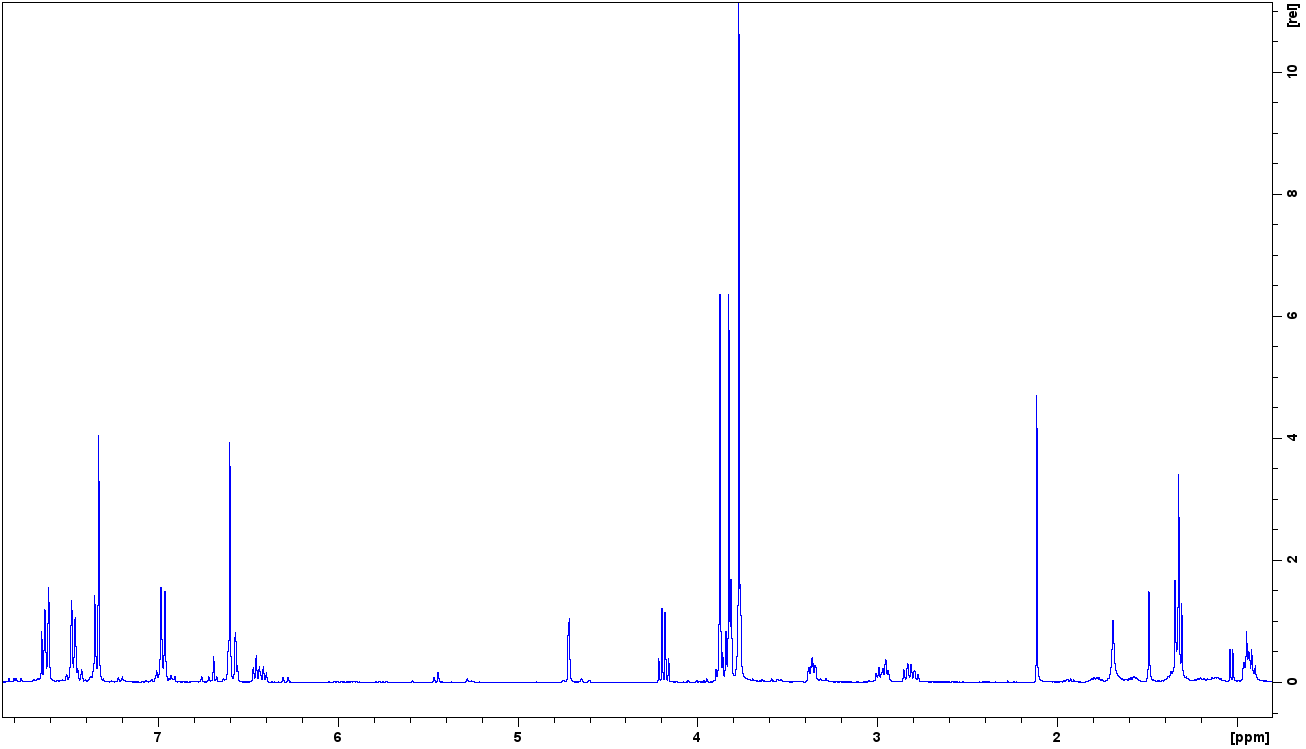

Supplement: Supplementary file 1 [file pharmaceuticals-16-01000-s001.zip › 17d mjm16728_1h.png]

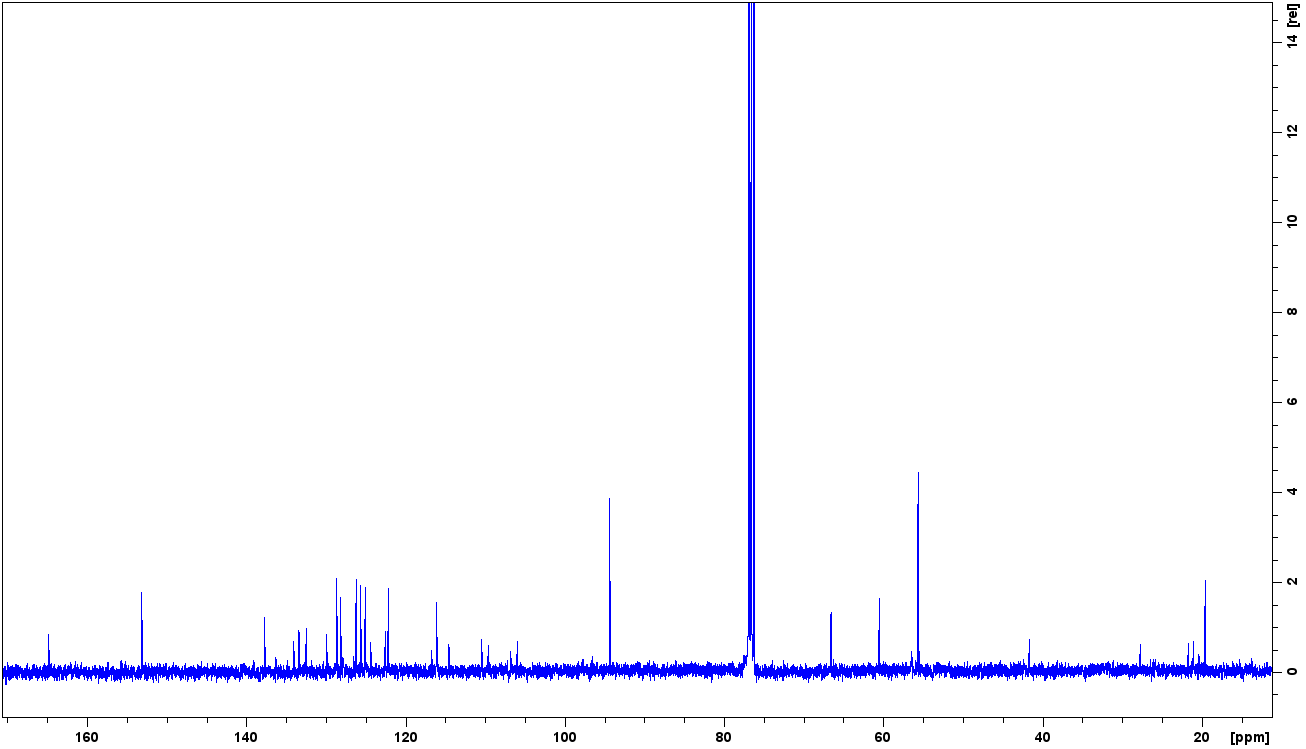

Supplement: Supplementary file 1 [file pharmaceuticals-16-01000-s001.zip › 17e mjm16778_13c.png]

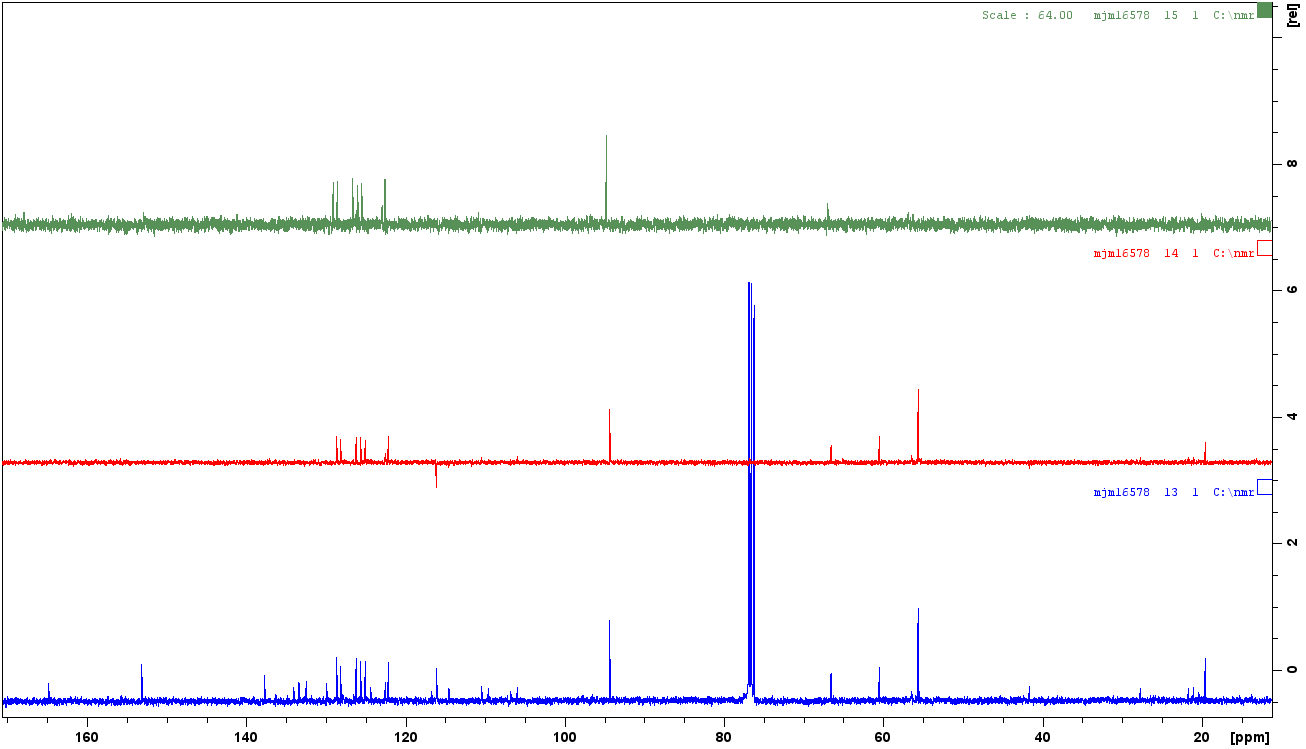

Supplement: Supplementary file 1 [file pharmaceuticals-16-01000-s001.zip › 17e mjm16778_13c_DEPTs.png]

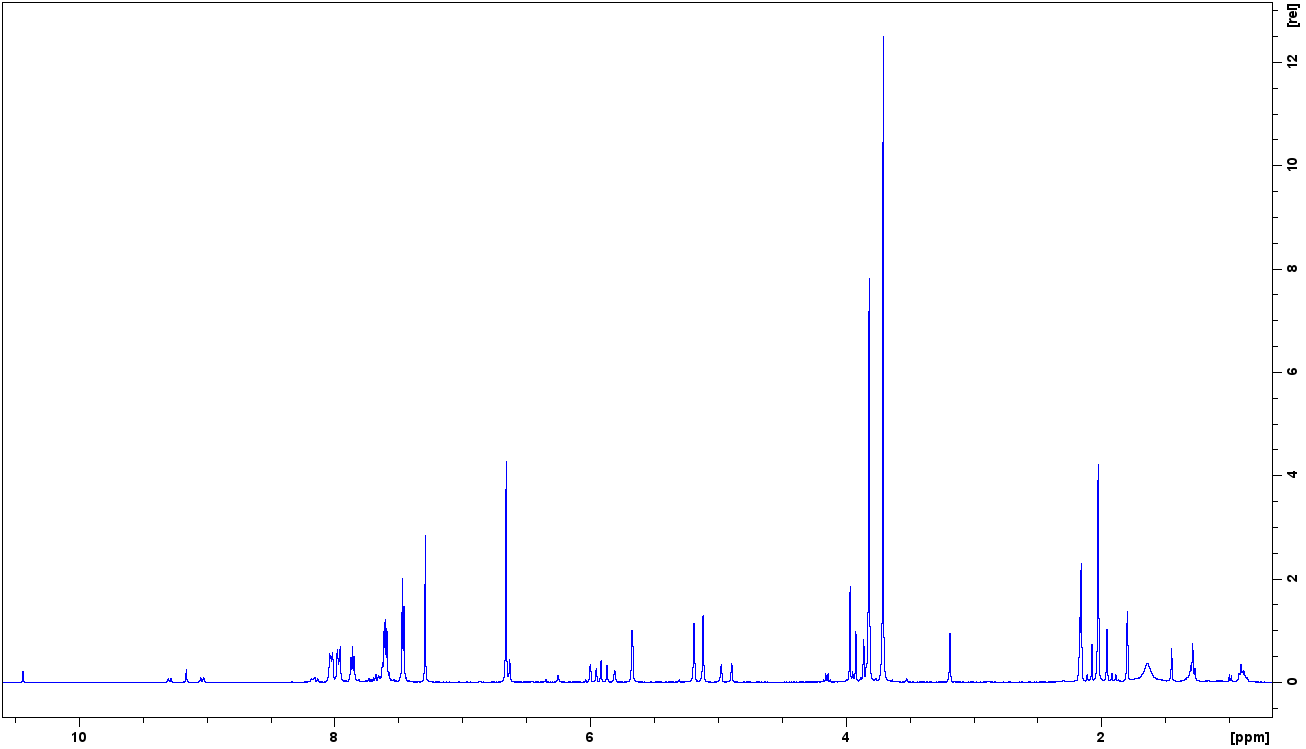

Supplement: Supplementary file 1 [file pharmaceuticals-16-01000-s001.zip › 17e mjm16778_1h.png]

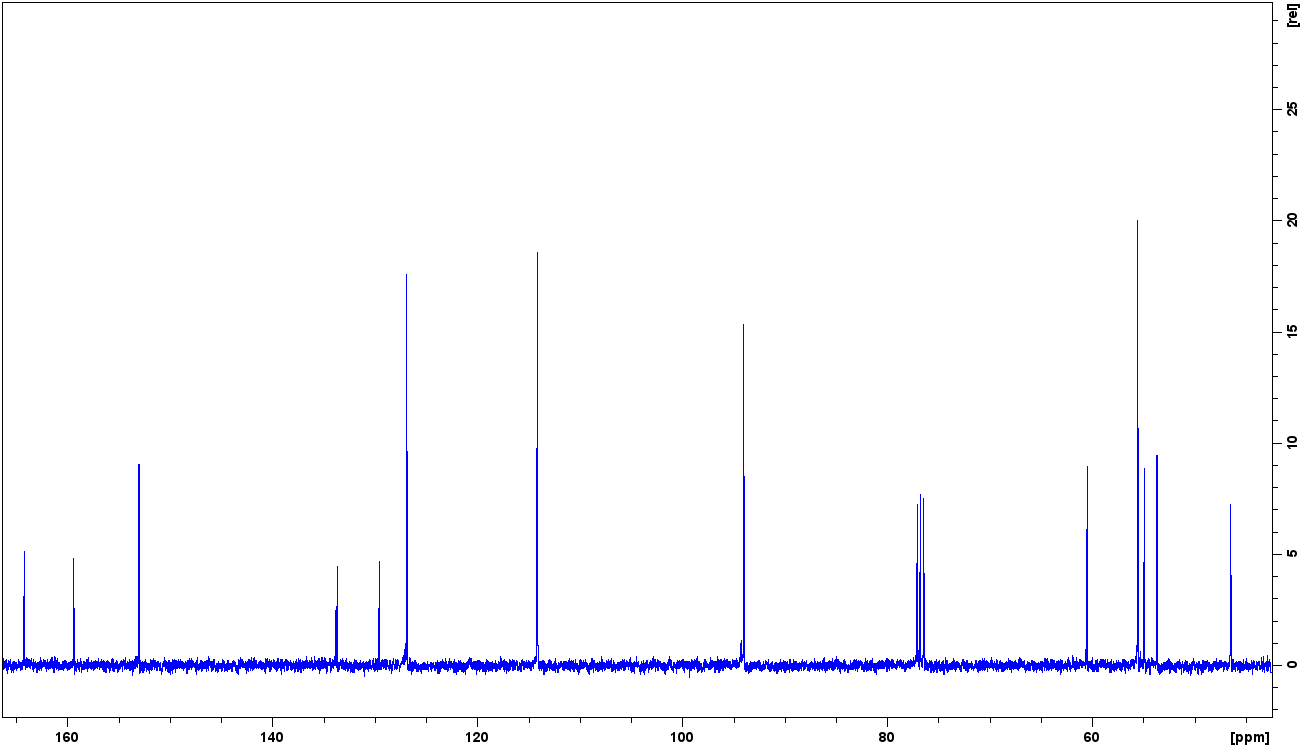

Supplement: Supplementary file 1 [file pharmaceuticals-16-01000-s001.zip › 17f mjm17064_13c.png]

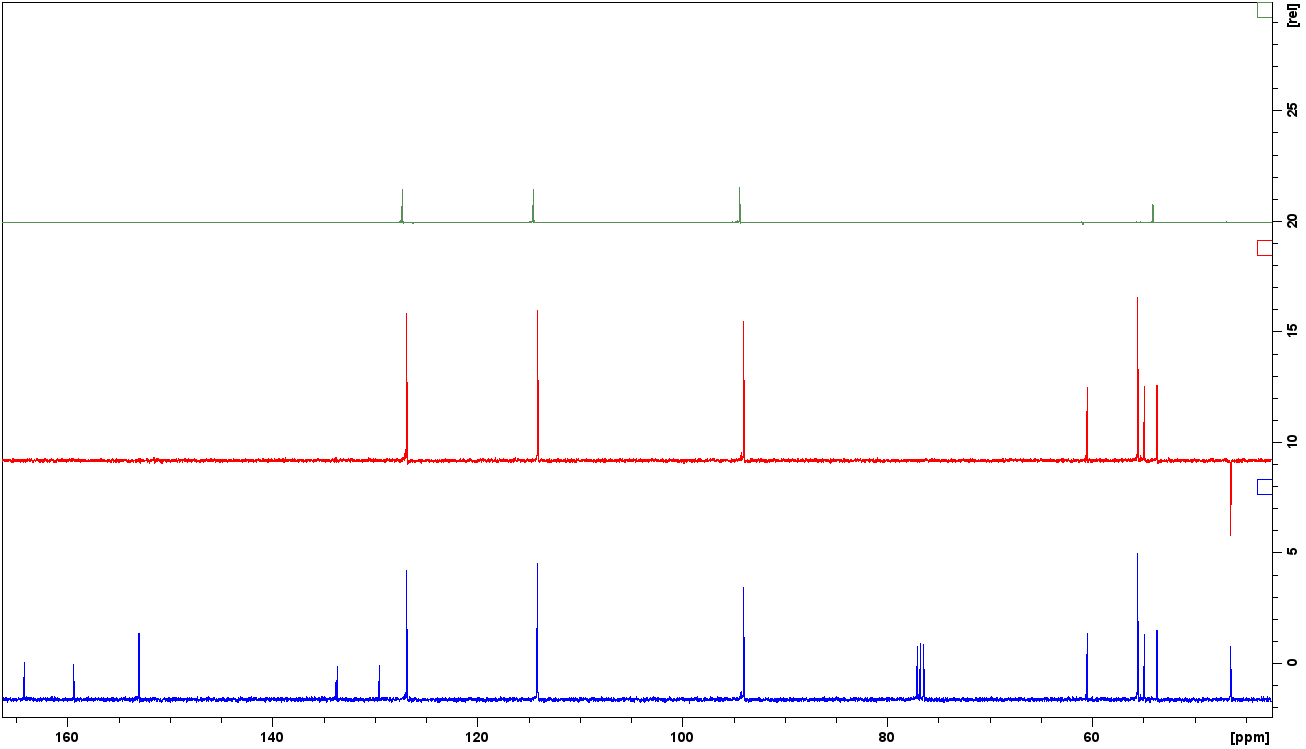

Supplement: Supplementary file 1 [file pharmaceuticals-16-01000-s001.zip › 17f mjm17064_13c_DEPTs.png]

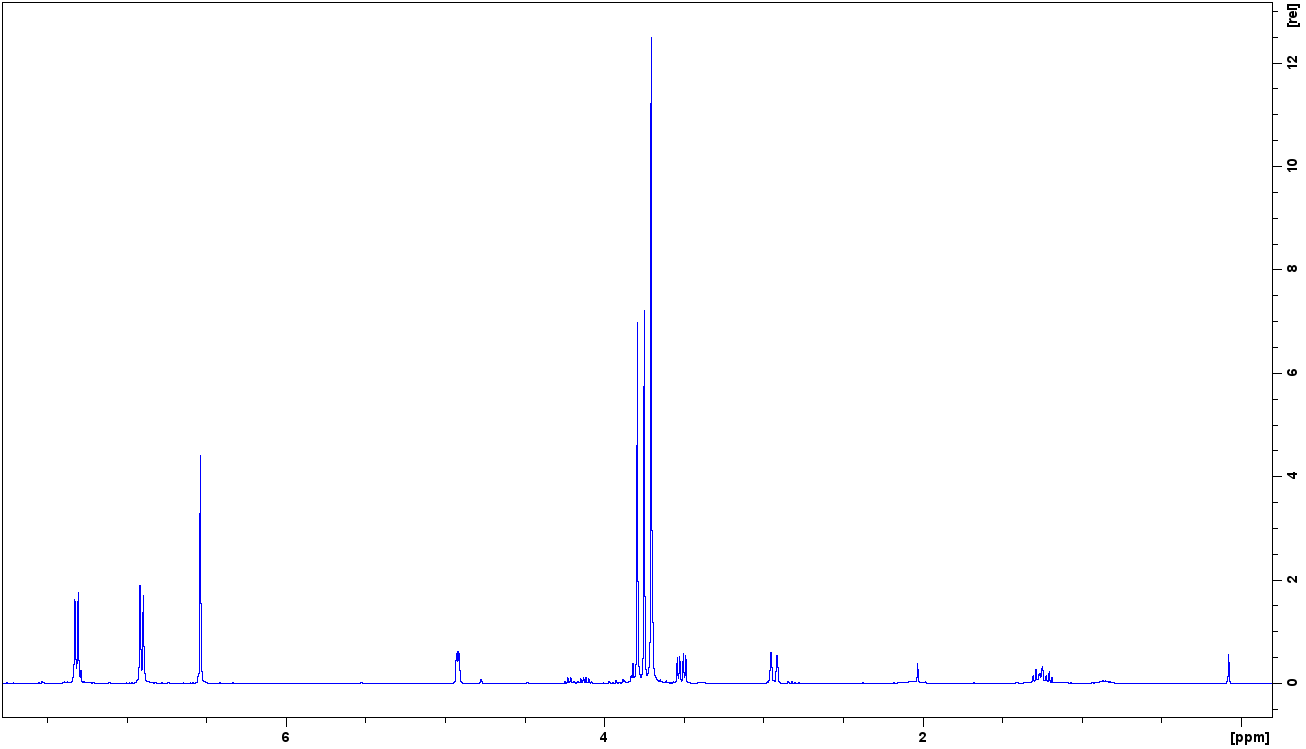

Supplement: Supplementary file 1 [file pharmaceuticals-16-01000-s001.zip › 17f mjm17064_1h.png]

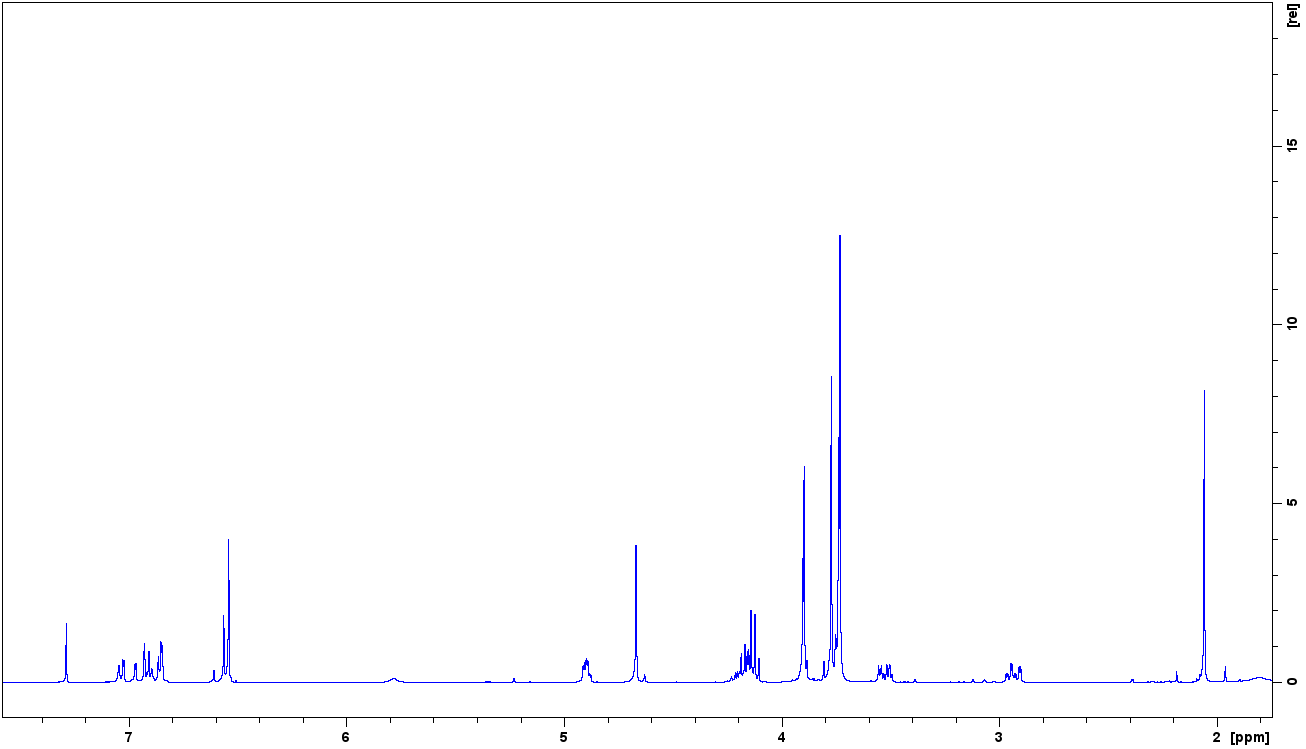

Supplement: Supplementary file 1 [file pharmaceuticals-16-01000-s001.zip › 17g mjm_17726_1h.png]

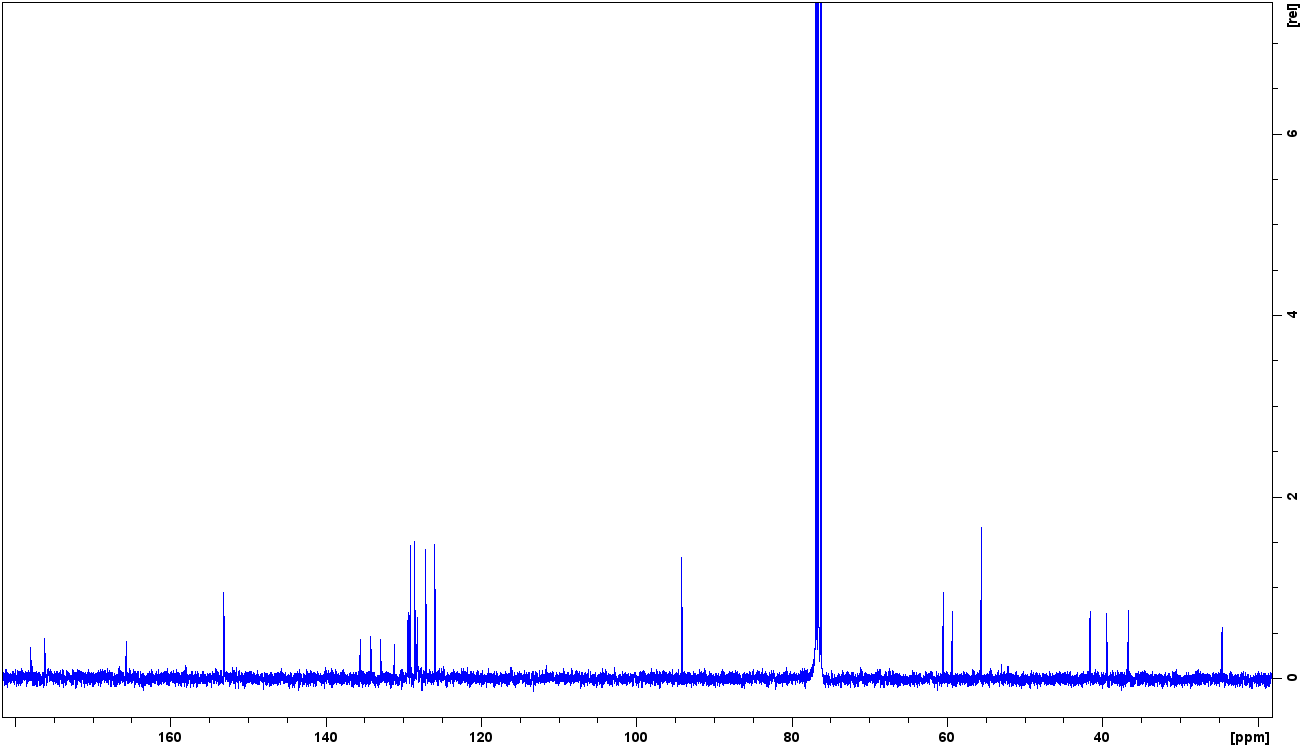

Supplement: Supplementary file 1 [file pharmaceuticals-16-01000-s001.zip › 21a mjm17790_13c.png]

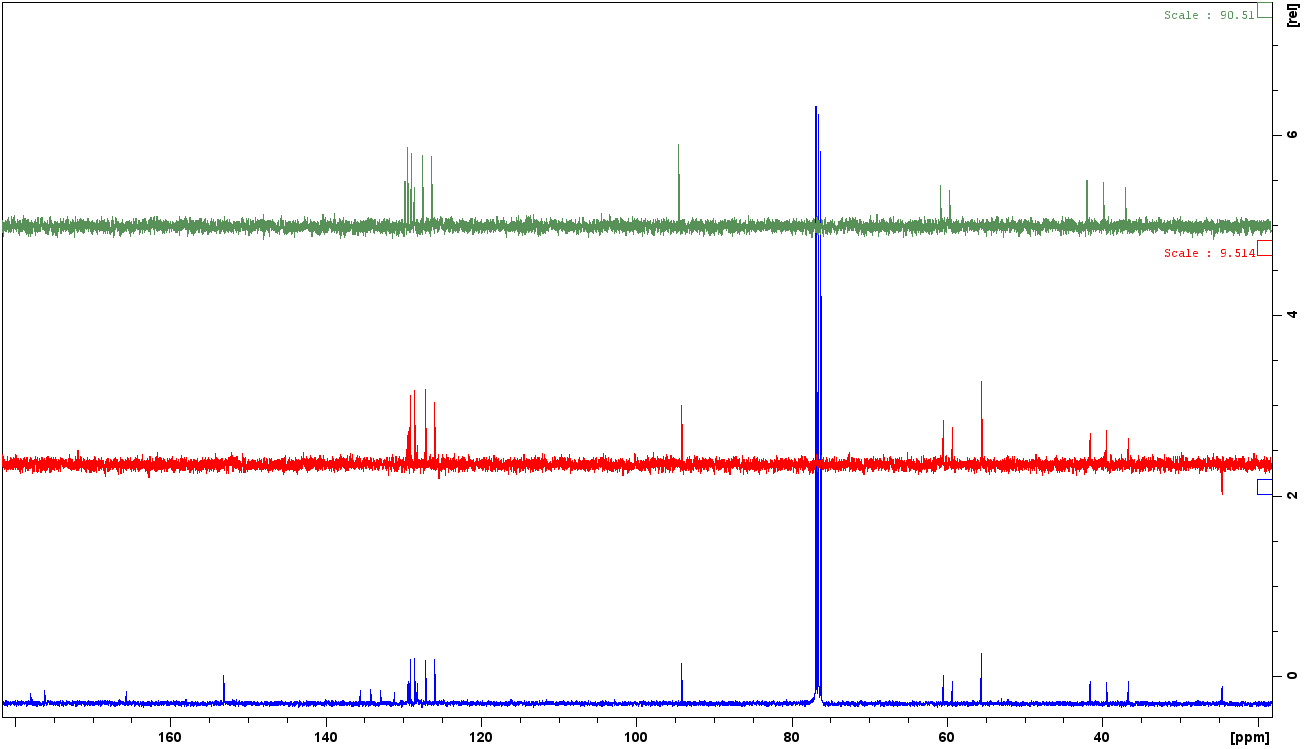

Supplement: Supplementary file 1 [file pharmaceuticals-16-01000-s001.zip › 21a mjm17790_13c_DEPTs.png]

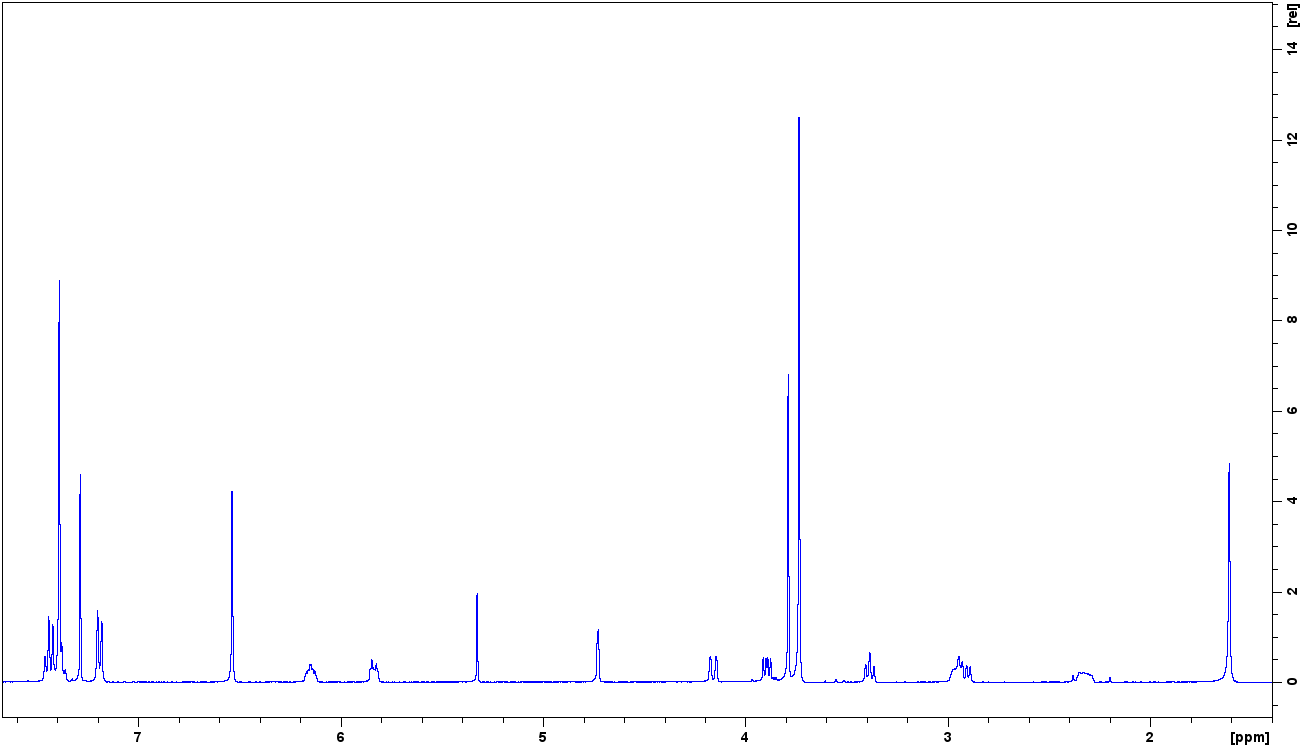

Supplement: Supplementary file 1 [file pharmaceuticals-16-01000-s001.zip › 21a mjm17790_1h.png]

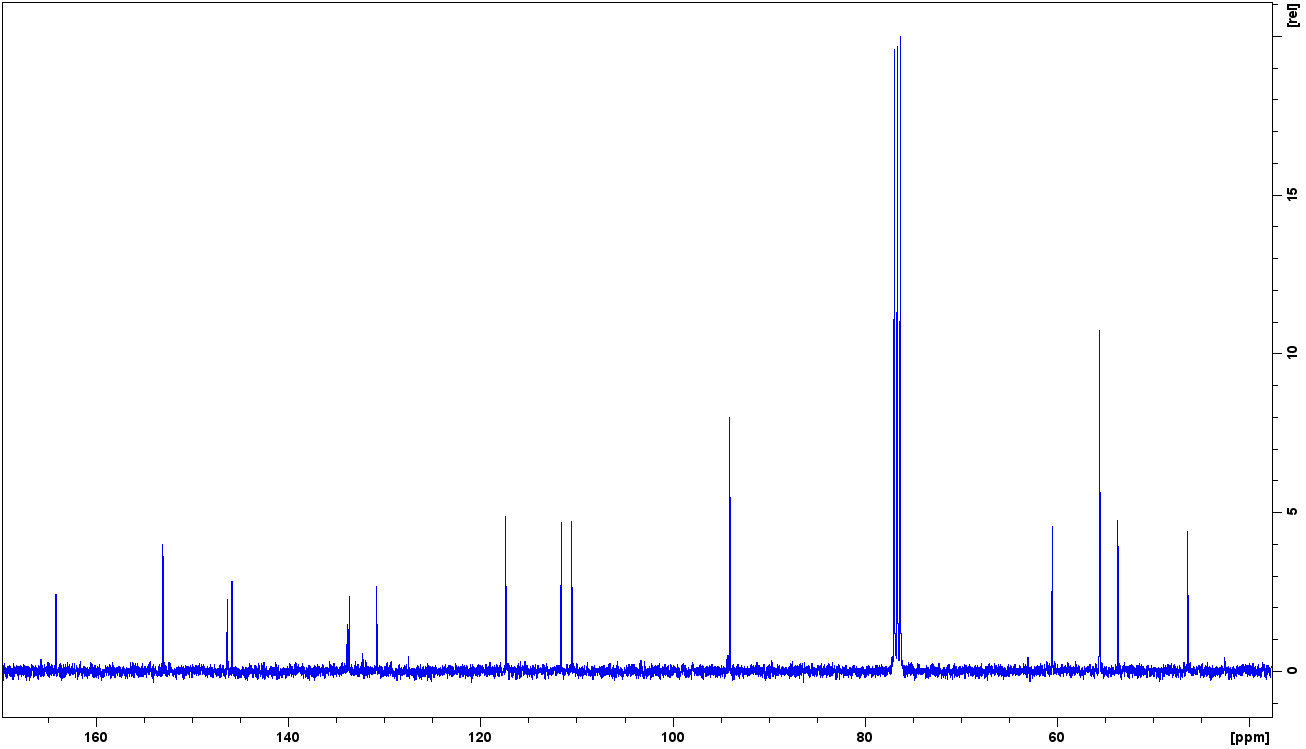

Supplement: Supplementary file 1 [file pharmaceuticals-16-01000-s001.zip › 21b mjm17752_13c.png]

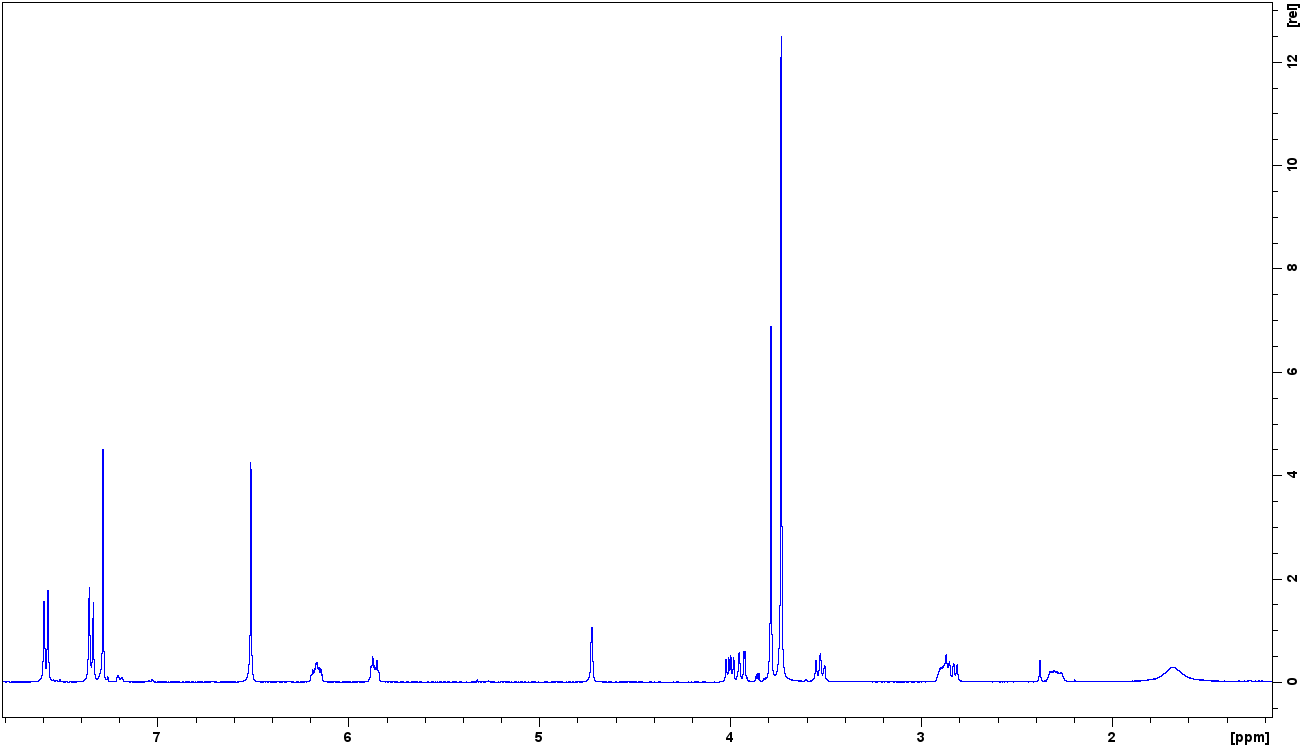

Supplement: Supplementary file 1 [file pharmaceuticals-16-01000-s001.zip › 21b mjm17752_1h.png]

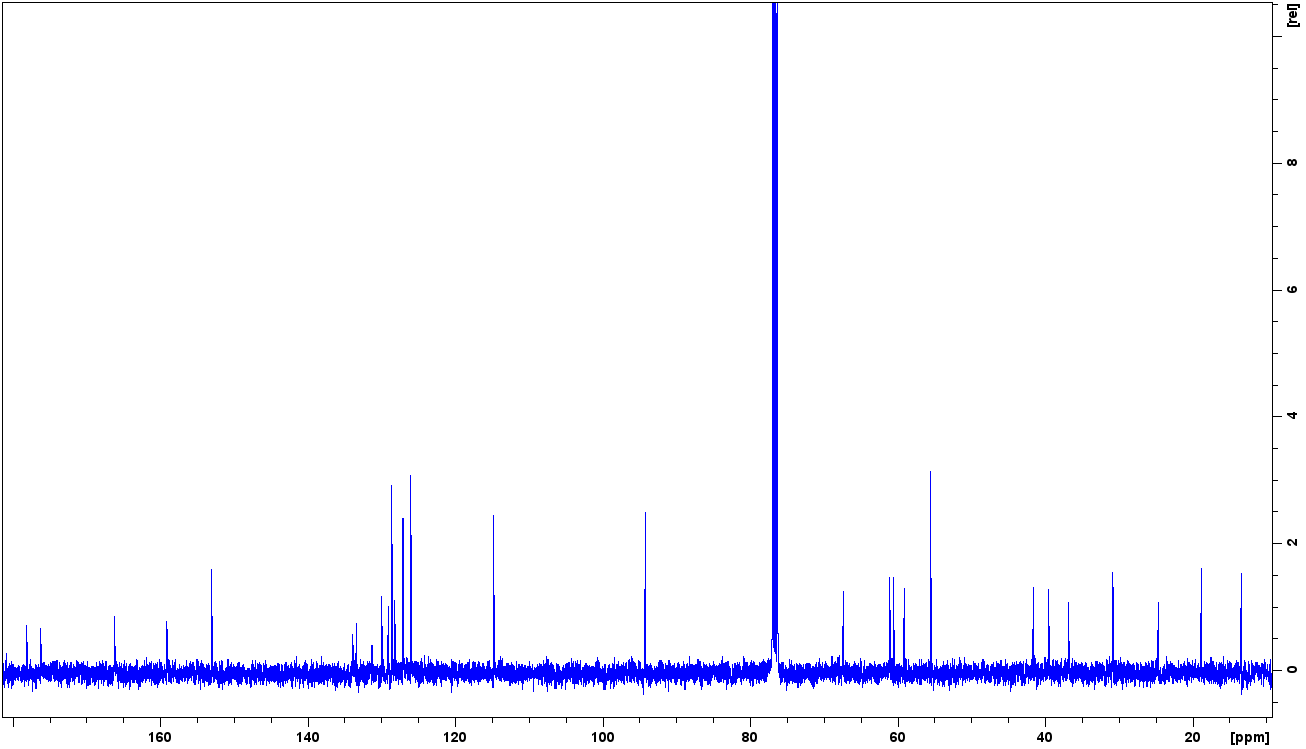

Supplement: Supplementary file 1 [file pharmaceuticals-16-01000-s001.zip › 21c mjm17804_13c.png]

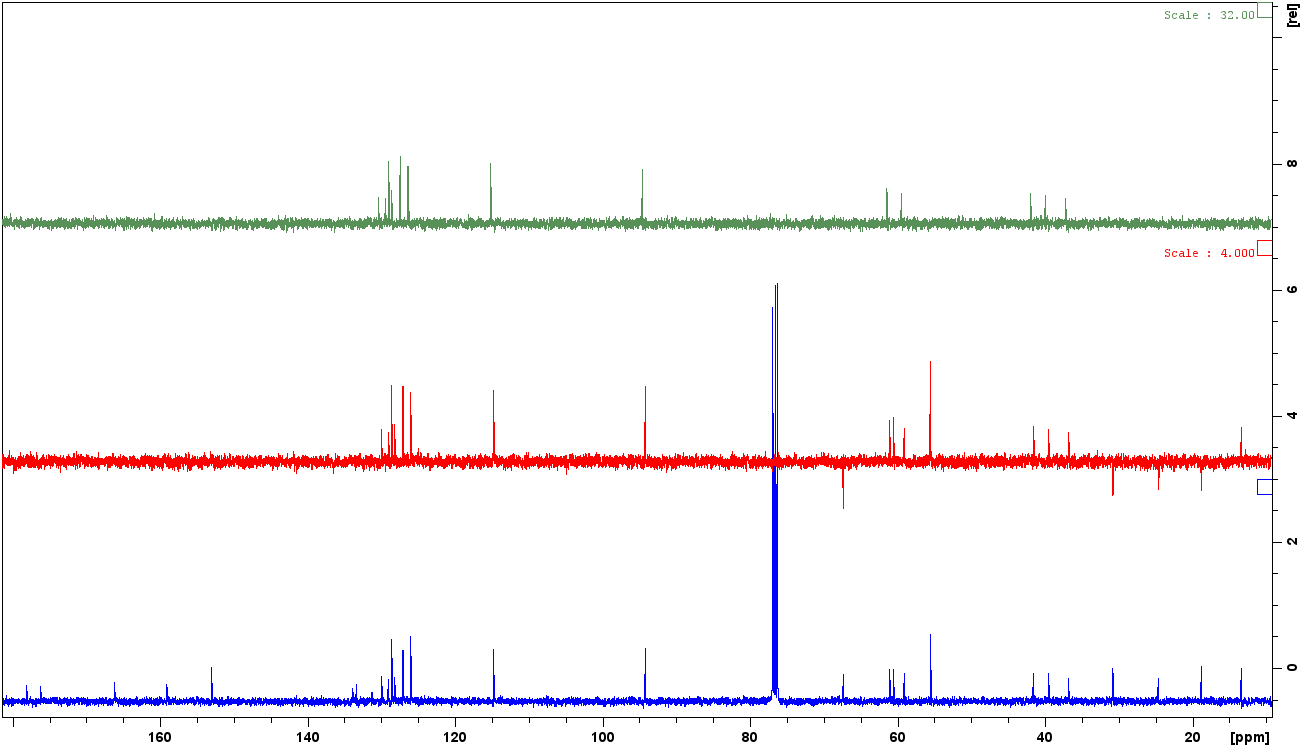

Supplement: Supplementary file 1 [file pharmaceuticals-16-01000-s001.zip › 21c mjm17804_13cDEPTs.png]

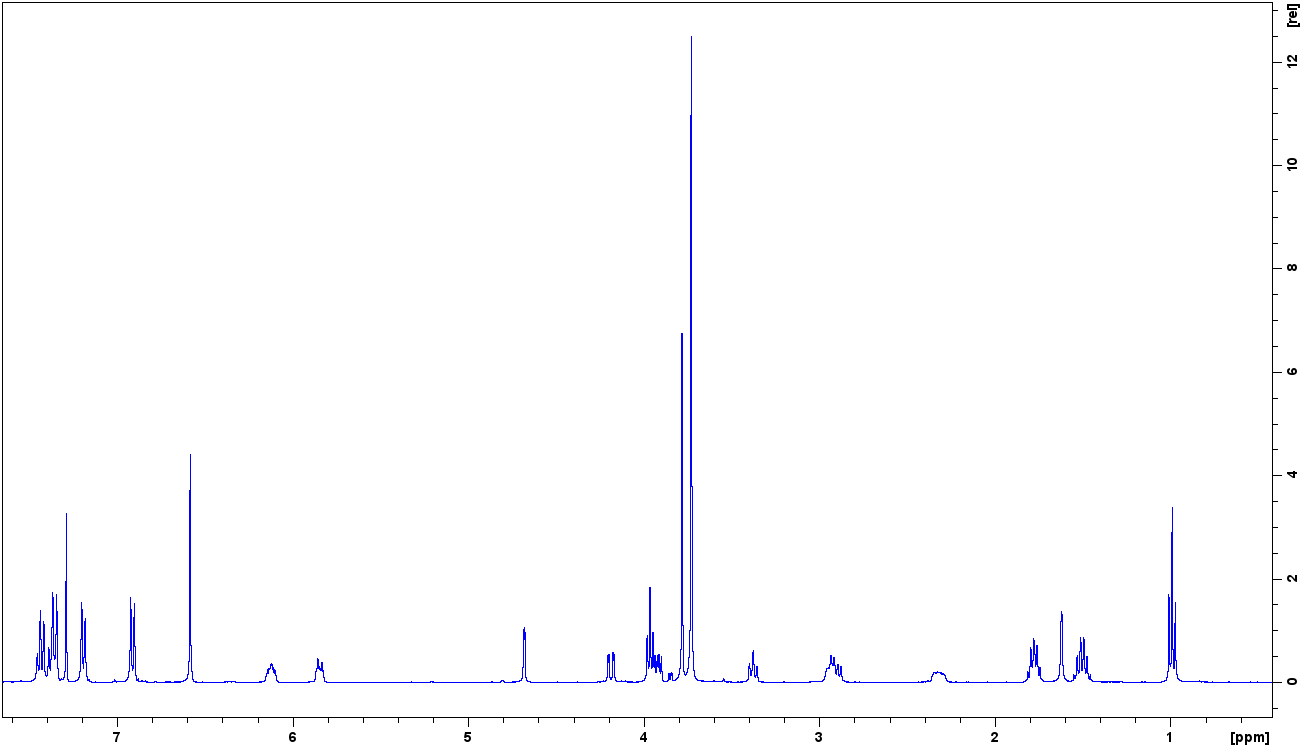

Supplement: Supplementary file 1 [file pharmaceuticals-16-01000-s001.zip › 21c mjm17804_1h.png]

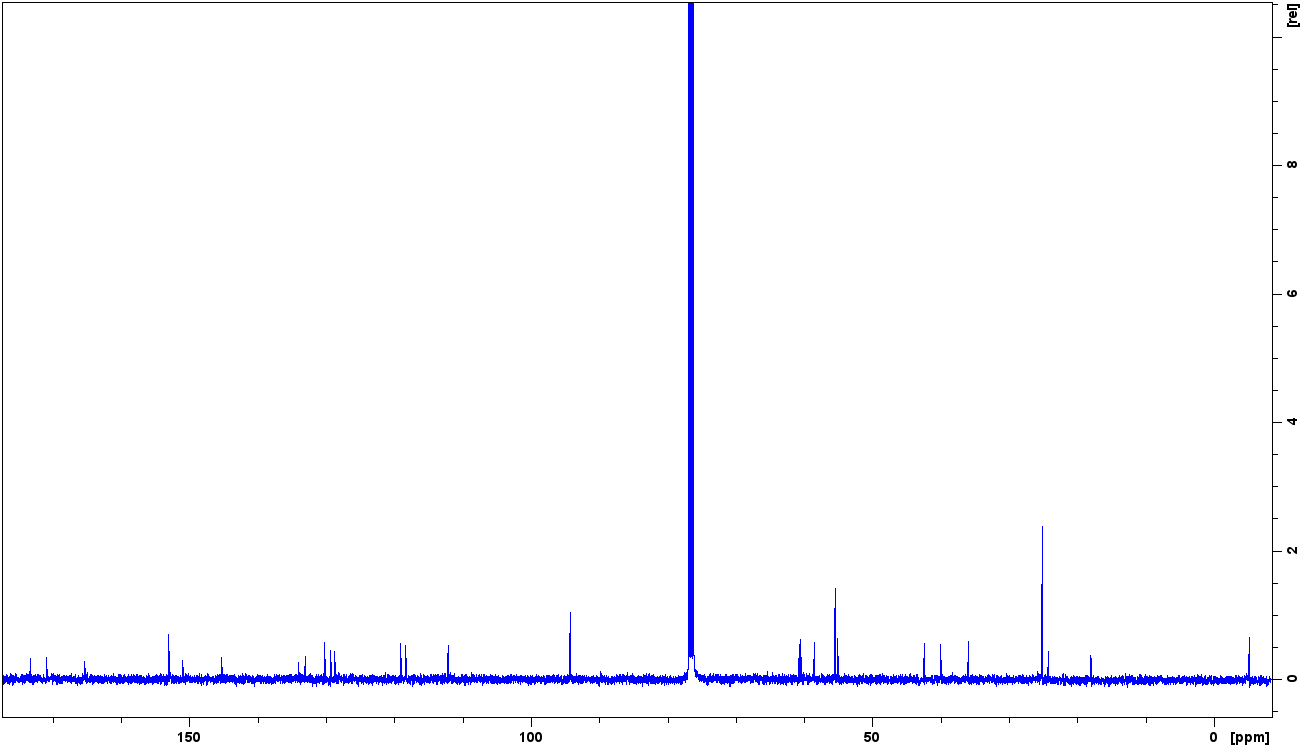

Supplement: Supplementary file 1 [file pharmaceuticals-16-01000-s001.zip › 21d mjm17772_13c.png]

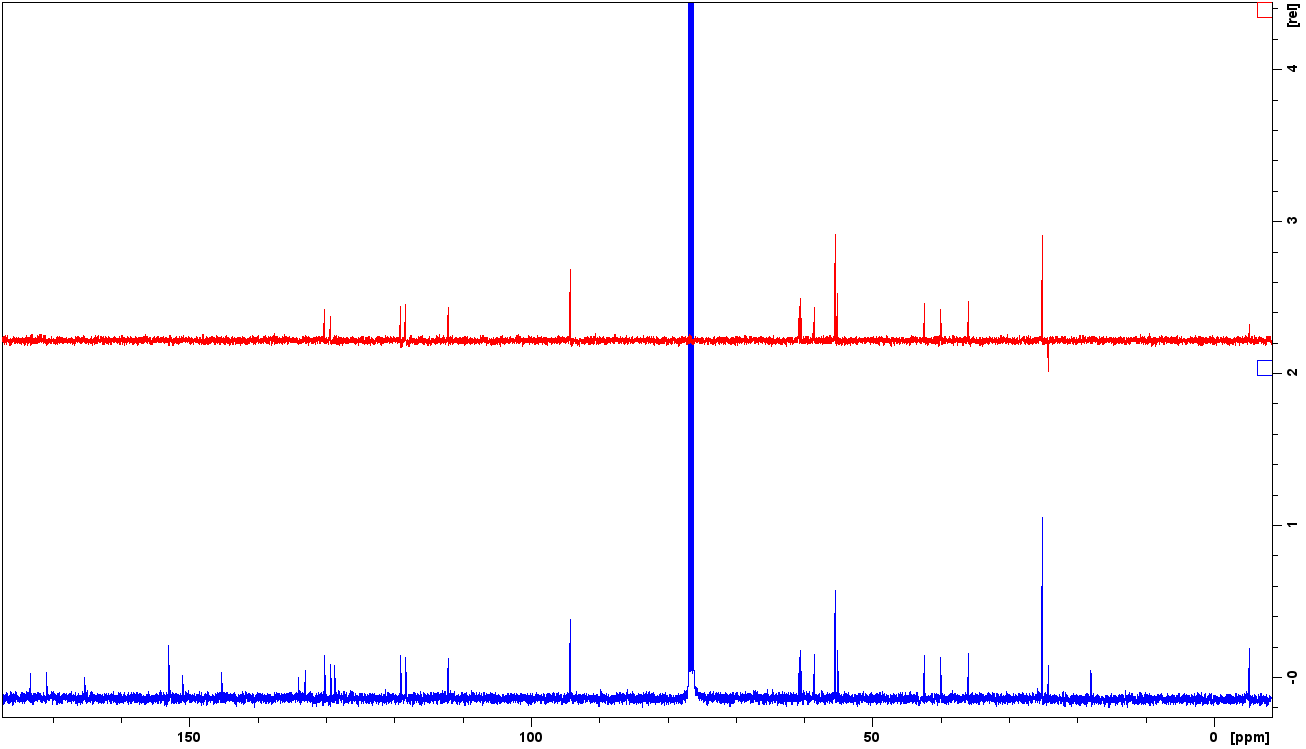

Supplement: Supplementary file 1 [file pharmaceuticals-16-01000-s001.zip › 21d mjm17772_13c_DEPT.png]

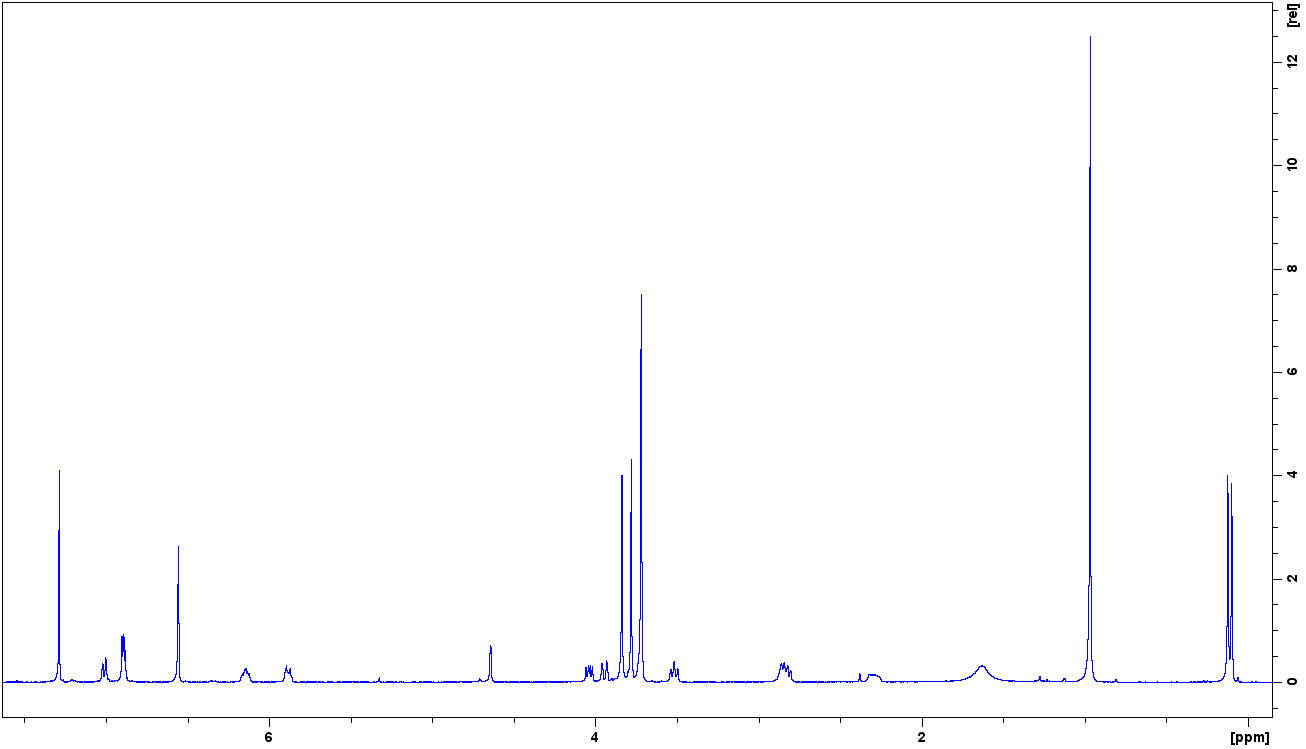

Supplement: Supplementary file 1 [file pharmaceuticals-16-01000-s001.zip › 21d mjm17772_1h.png]

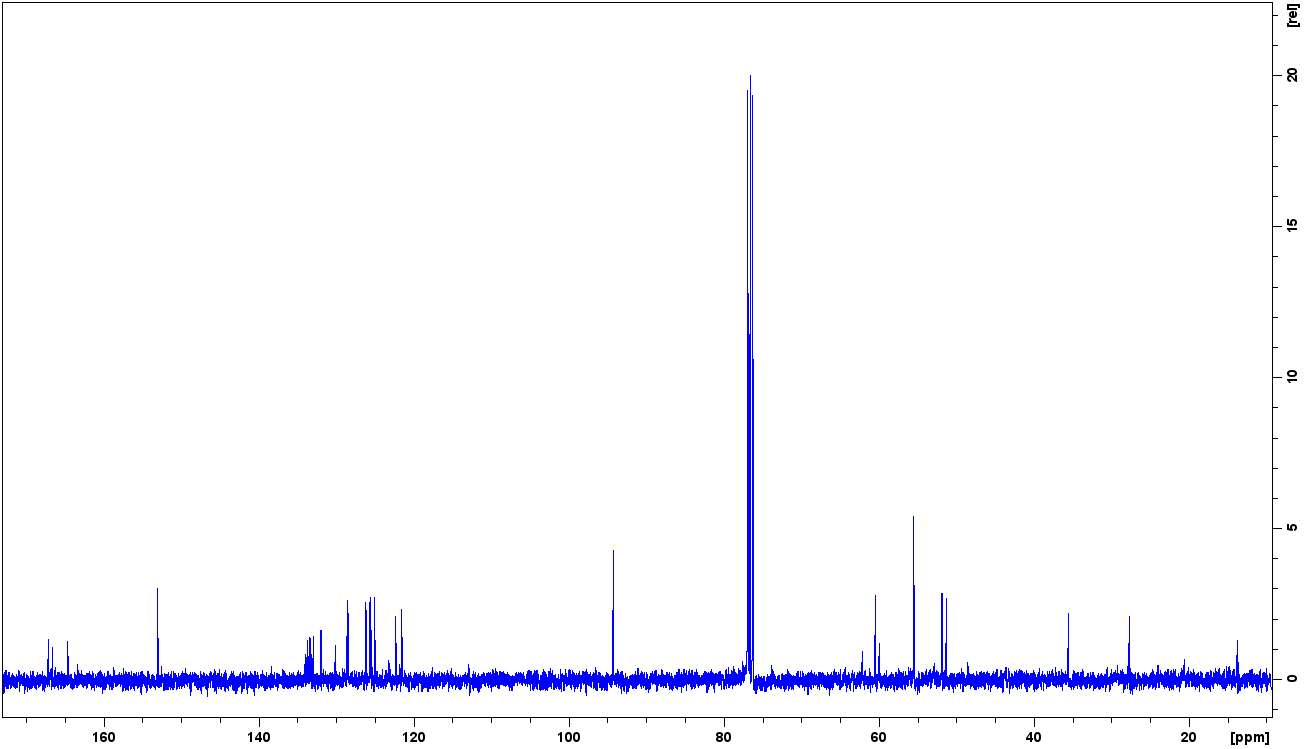

Supplement: Supplementary file 1 [file pharmaceuticals-16-01000-s001.zip › 22a mjm17806_13c.png]

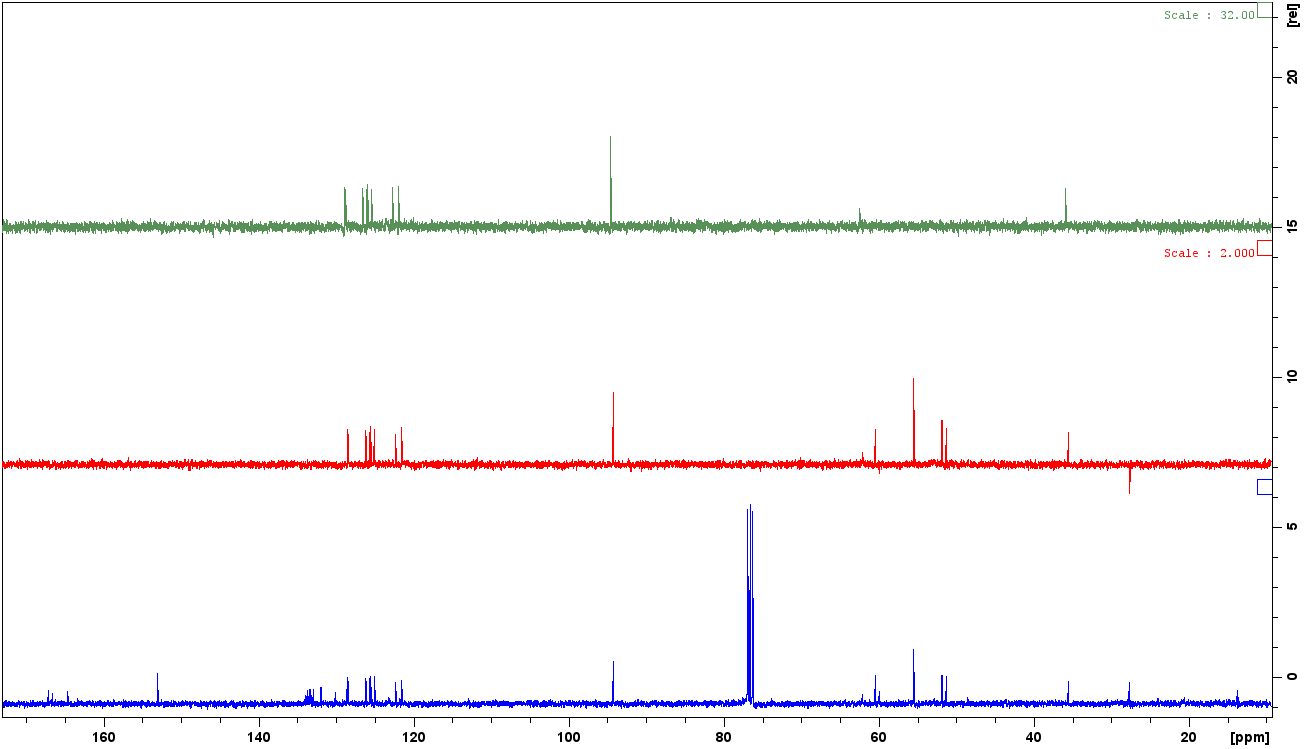

Supplement: Supplementary file 1 [file pharmaceuticals-16-01000-s001.zip › 22a mjm17806_13c_DEPTs.png]

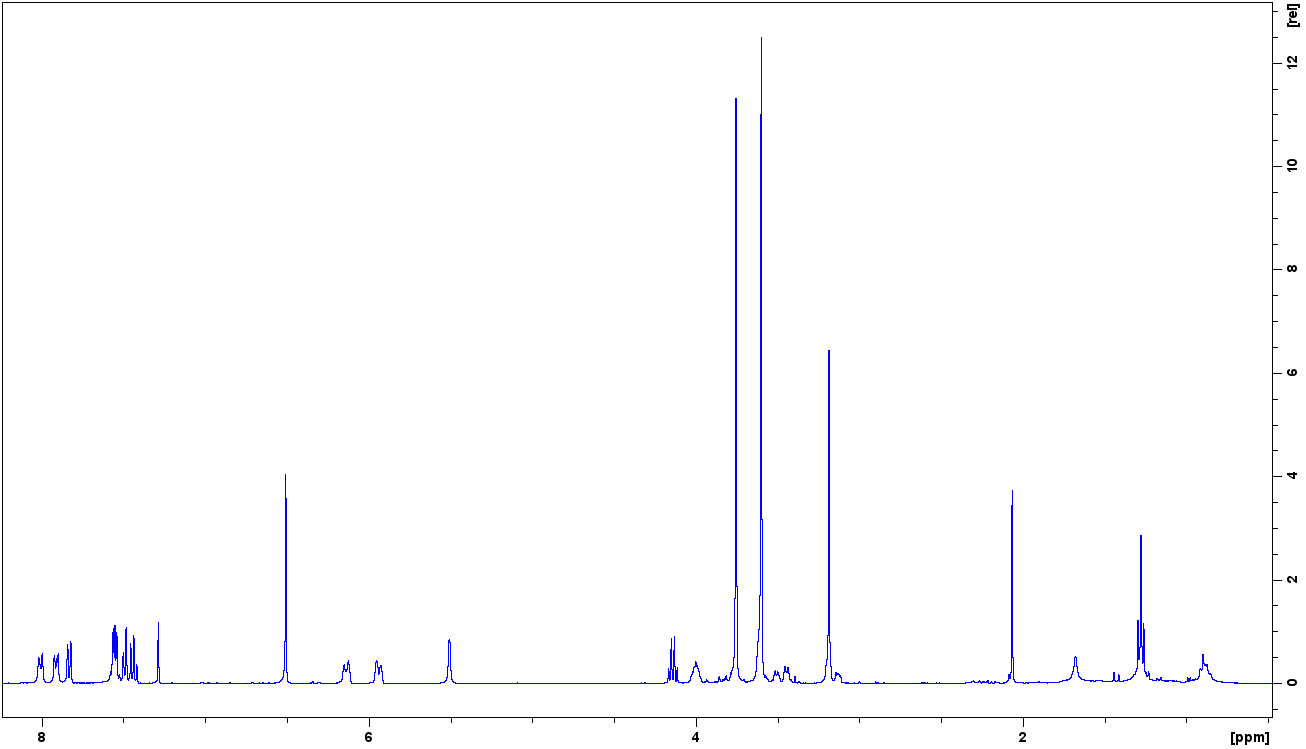

Supplement: Supplementary file 1 [file pharmaceuticals-16-01000-s001.zip › 22a mjm17806_1h.png]

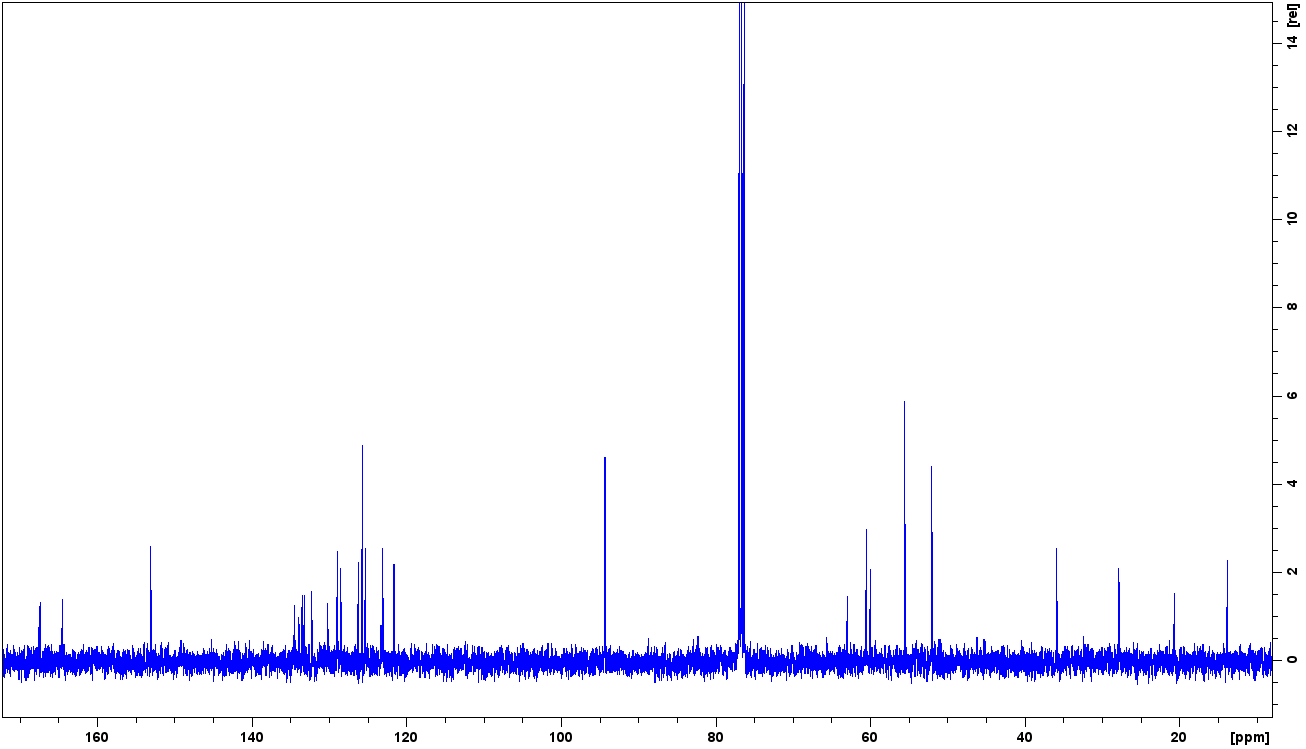

Supplement: Supplementary file 1 [file pharmaceuticals-16-01000-s001.zip › 22b mjm17807_13c.png]

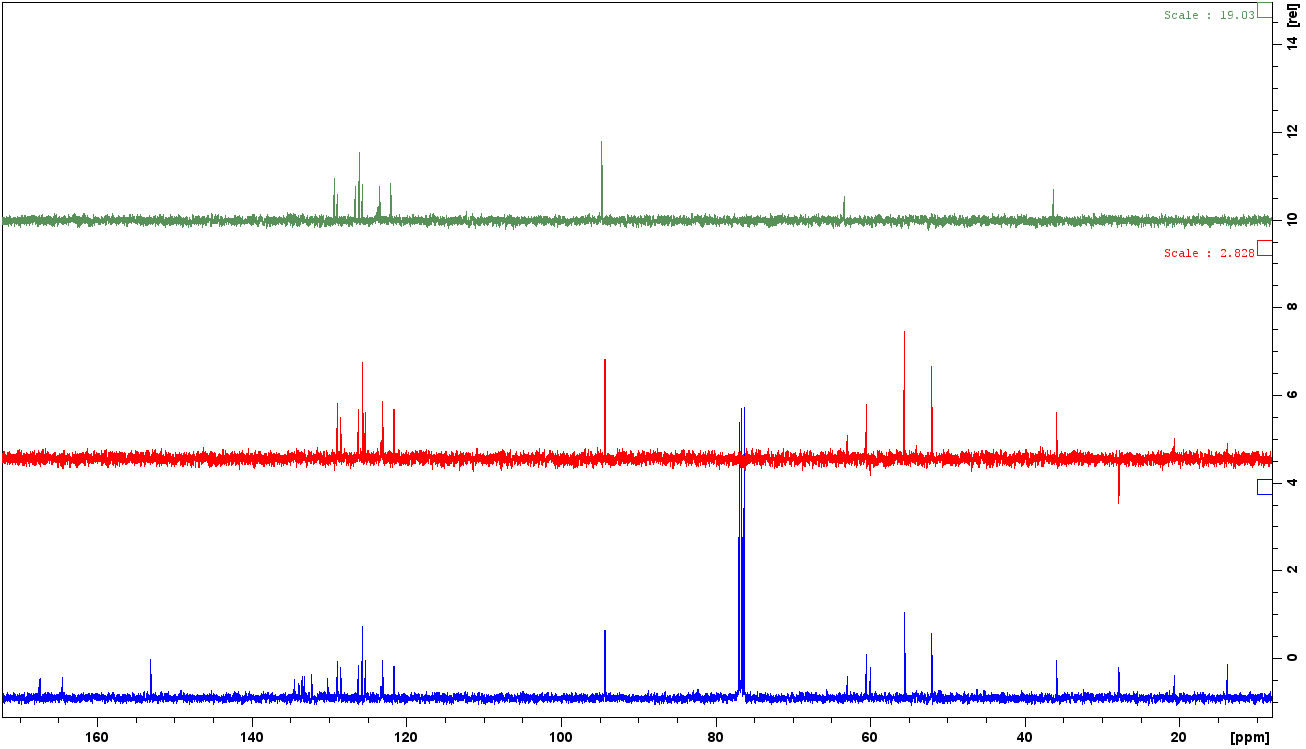

Supplement: Supplementary file 1 [file pharmaceuticals-16-01000-s001.zip › 22b mjm17807_13c_DEPTs.png]

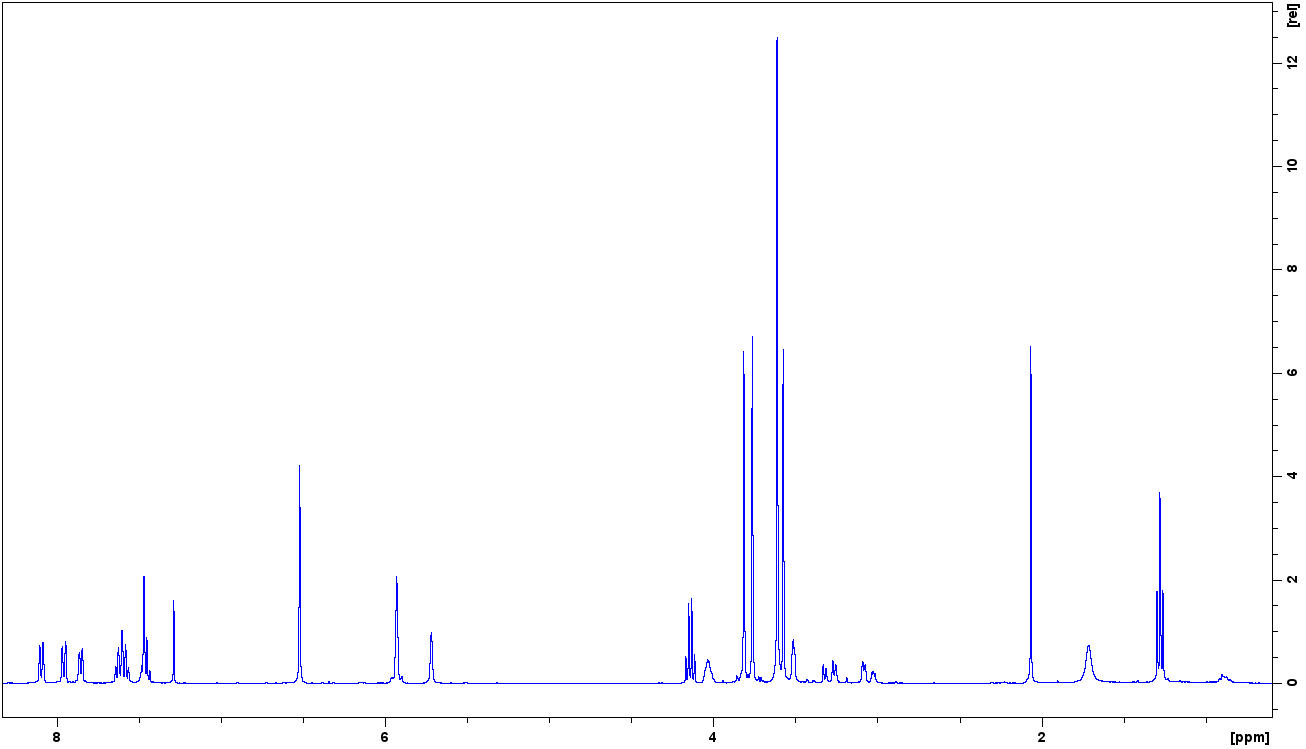

Supplement: Supplementary file 1 [file pharmaceuticals-16-01000-s001.zip › 22b mjm17807_1h.png]

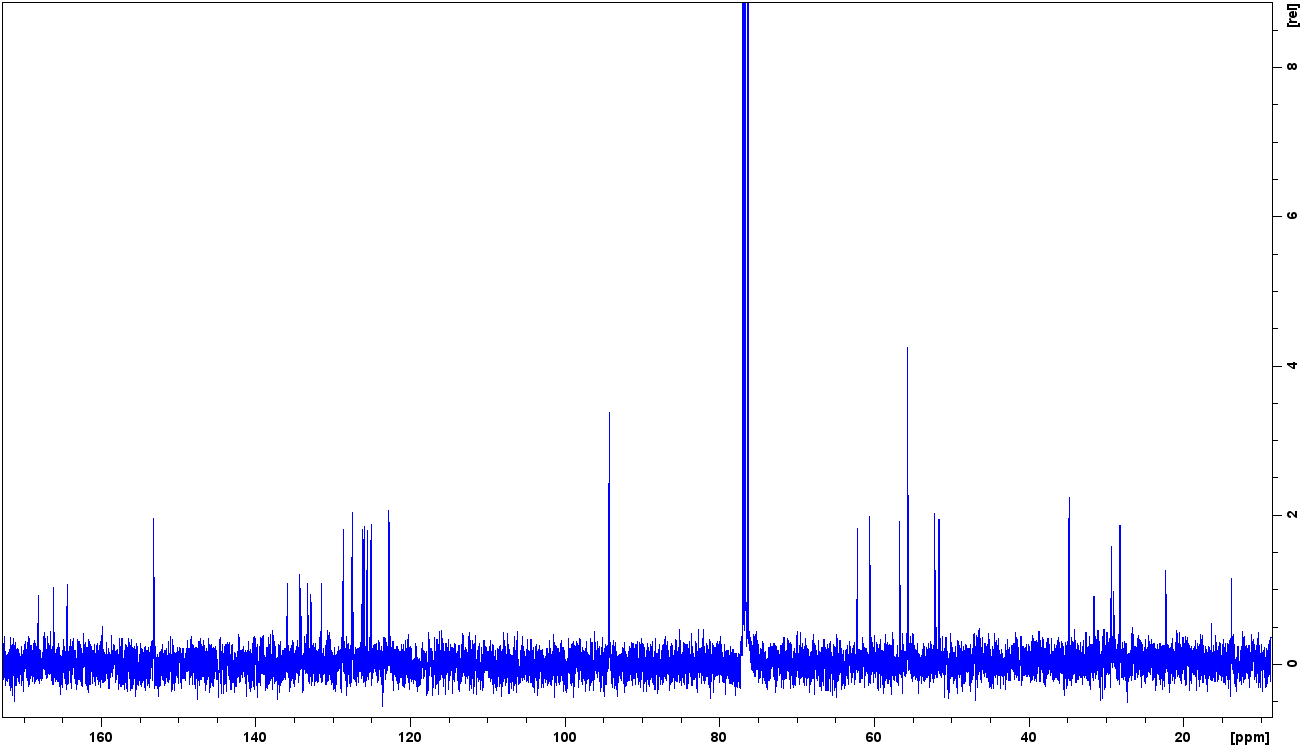

Supplement: Supplementary file 1 [file pharmaceuticals-16-01000-s001.zip › 22c mjm17797_13c.png]

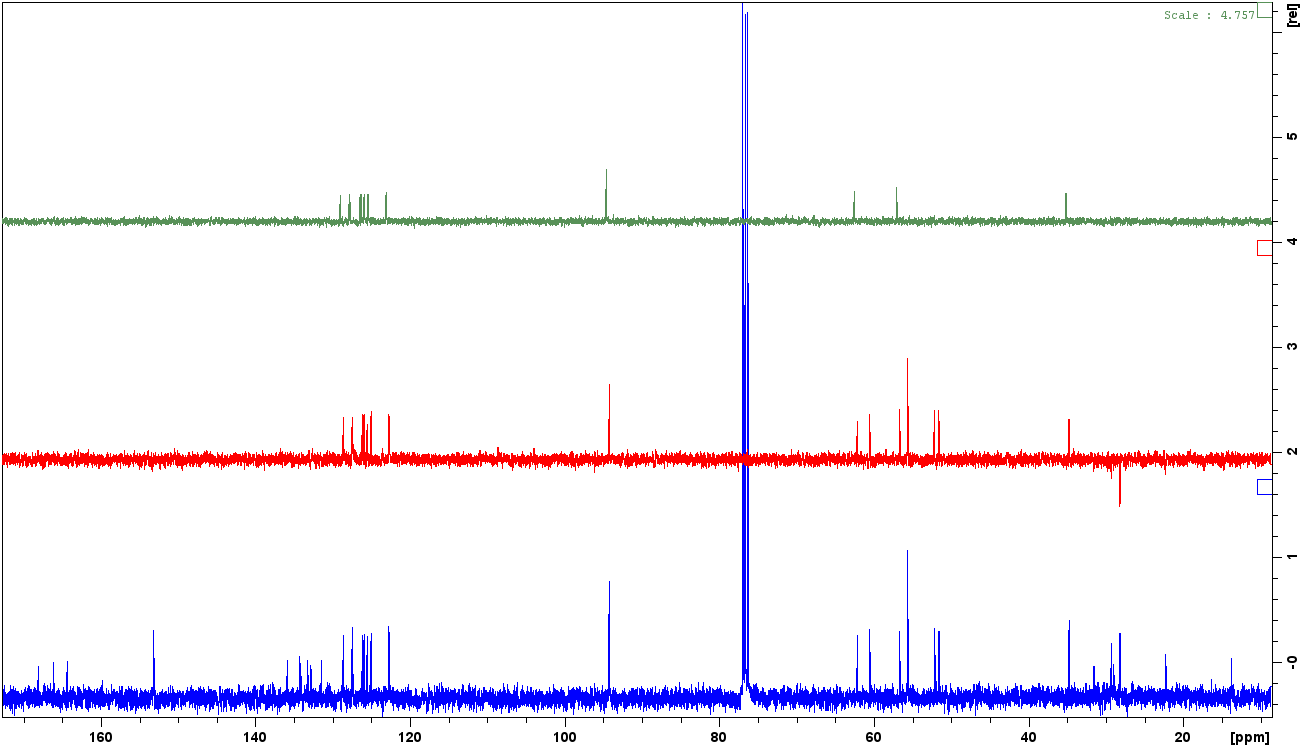

Supplement: Supplementary file 1 [file pharmaceuticals-16-01000-s001.zip › 22c mjm17797_13c_DEPTs.png]

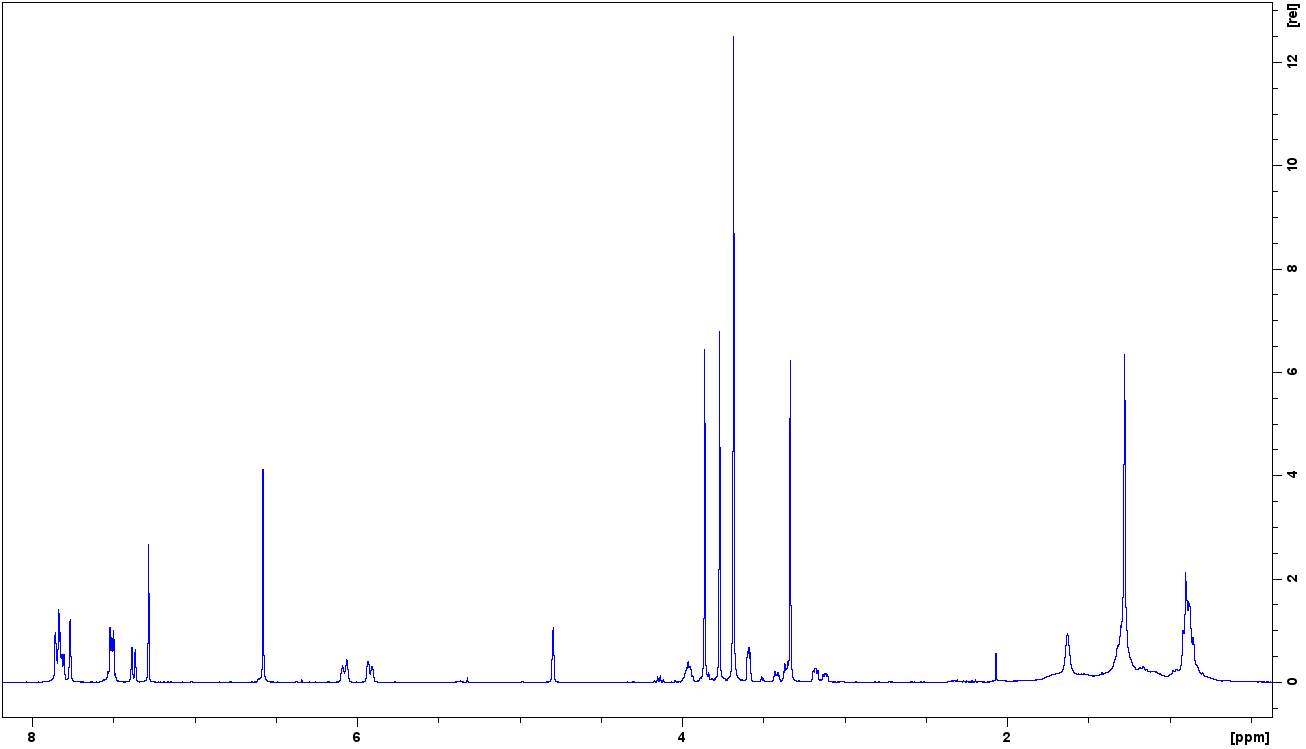

Supplement: Supplementary file 1 [file pharmaceuticals-16-01000-s001.zip › 22c mjm17797_1h.png]

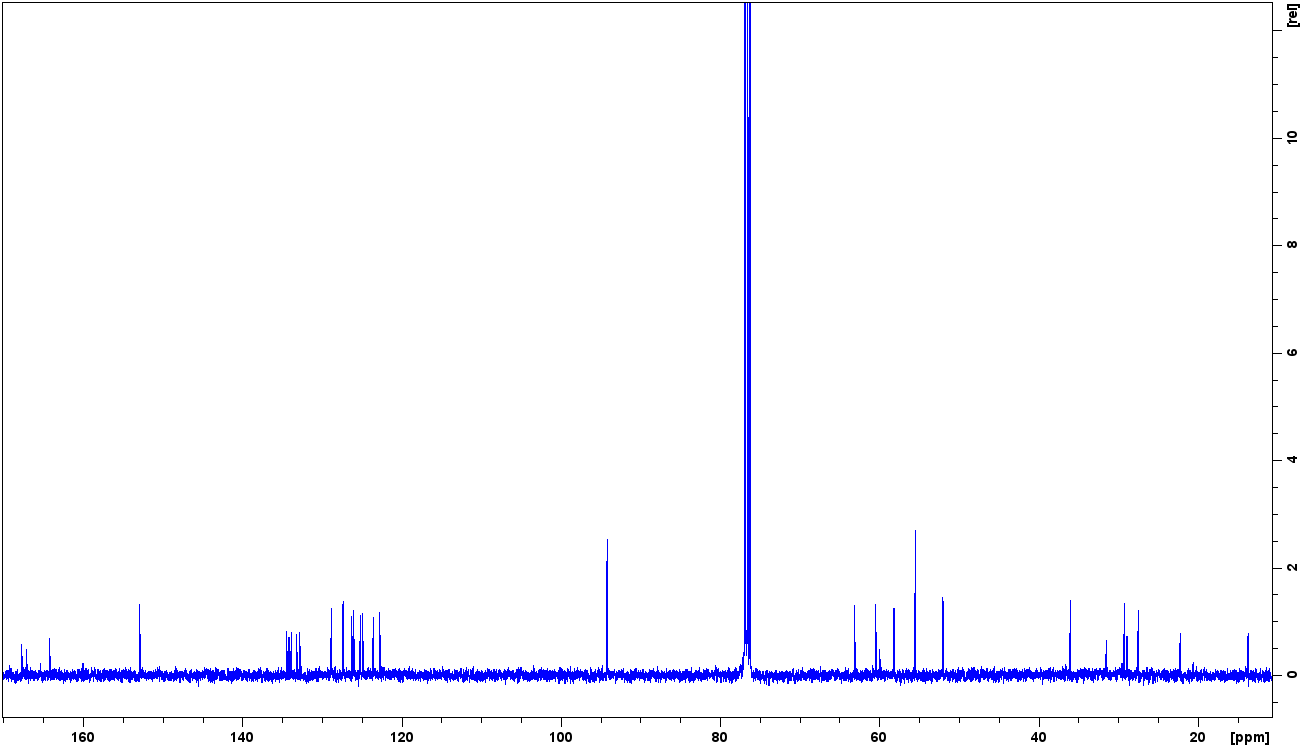

Supplement: Supplementary file 1 [file pharmaceuticals-16-01000-s001.zip › 22d mjm17798_13c.png]

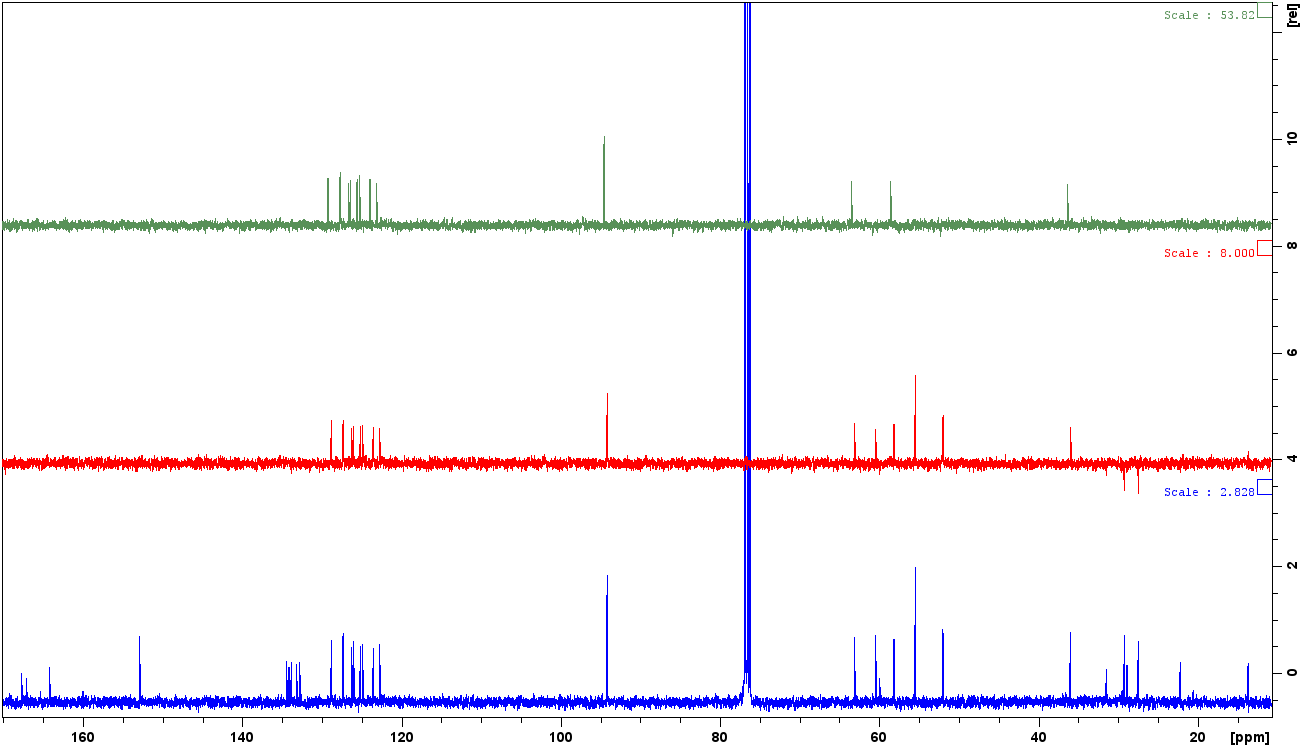

Supplement: Supplementary file 1 [file pharmaceuticals-16-01000-s001.zip › 22d mjm17798_13c_DEPTs.png]

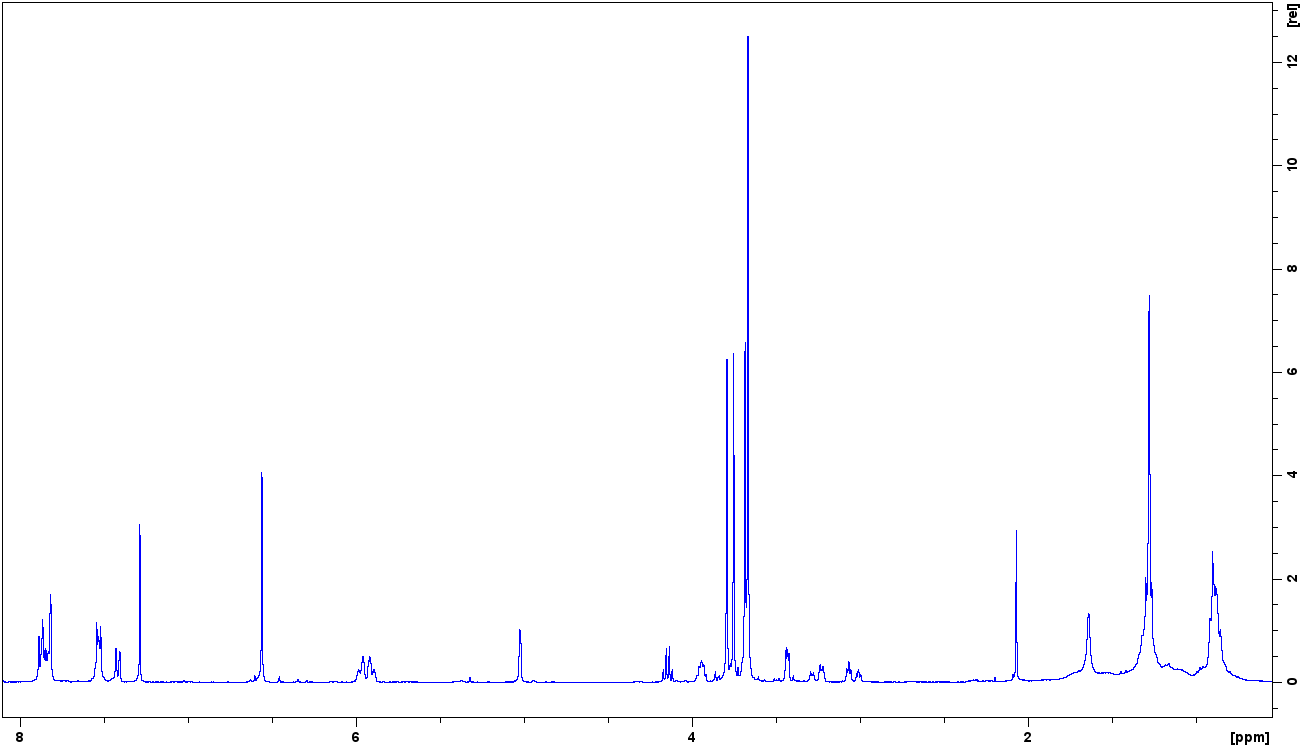

Supplement: Supplementary file 1 [file pharmaceuticals-16-01000-s001.zip › 22d mjm17798_1h.png]

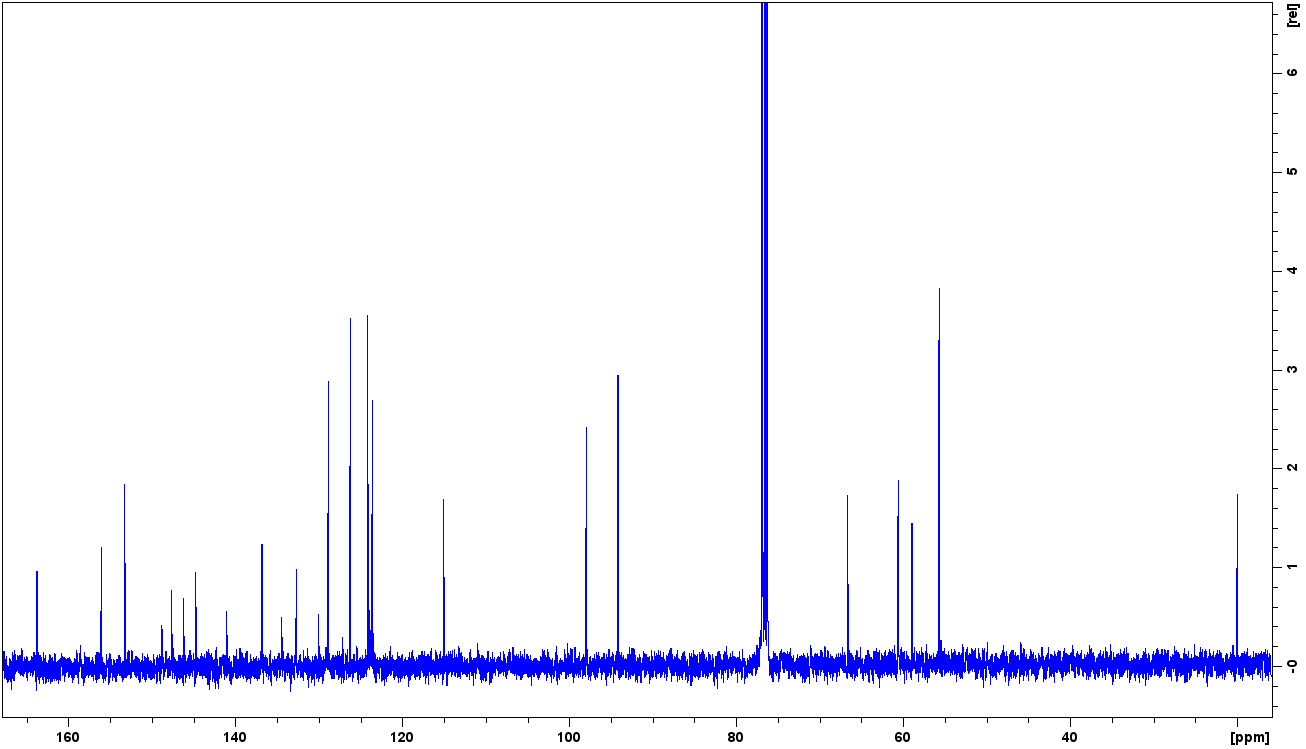

Supplement: Supplementary file 1 [file pharmaceuticals-16-01000-s001.zip › 9a mjm_15768_13c.png]

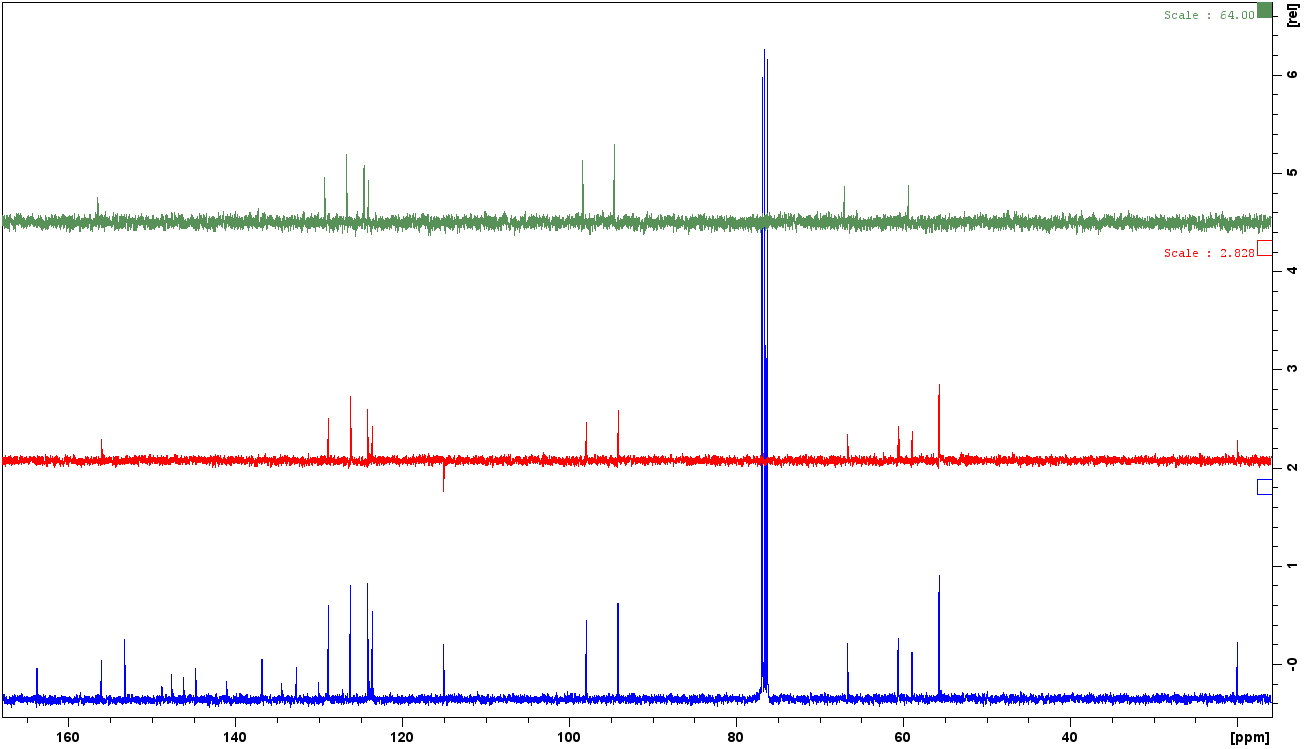

Supplement: Supplementary file 1 [file pharmaceuticals-16-01000-s001.zip › 9a mjm_15768_13c_DEPTs.png]

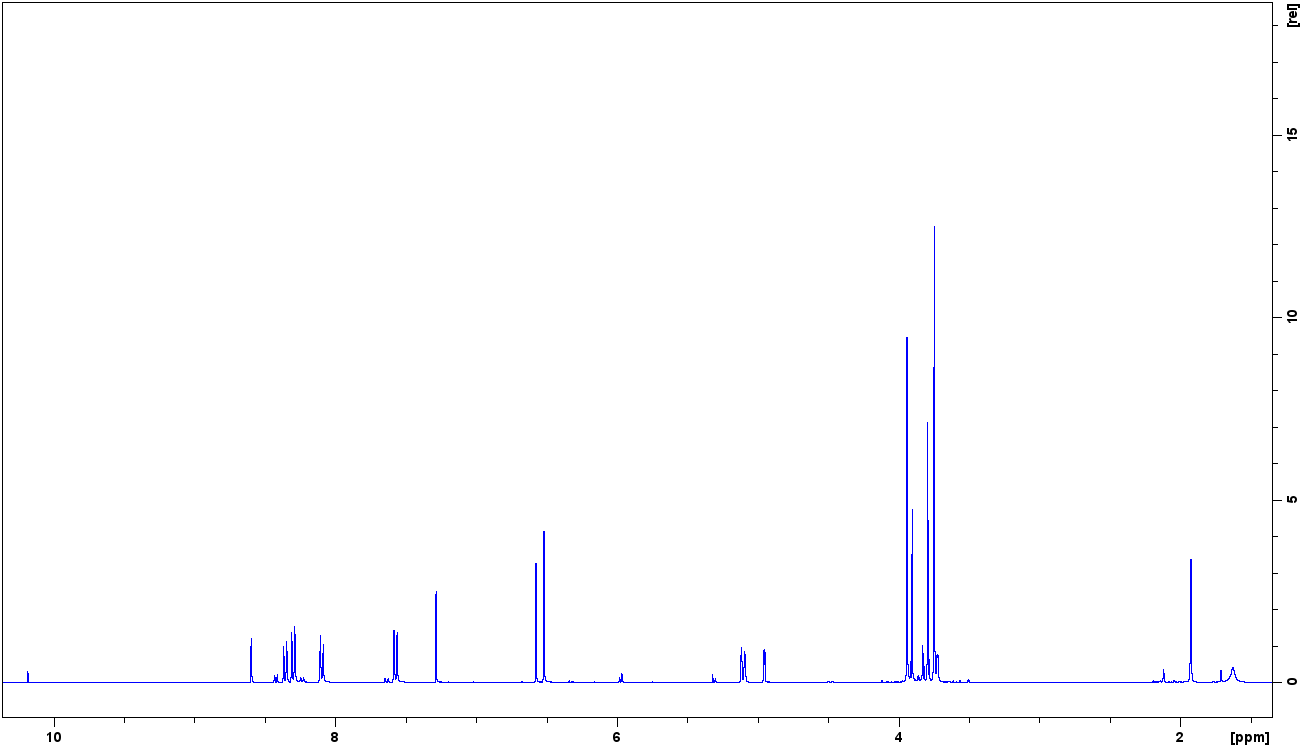

Supplement: Supplementary file 1 [file pharmaceuticals-16-01000-s001.zip › 9a mjm_15768_1h.png]

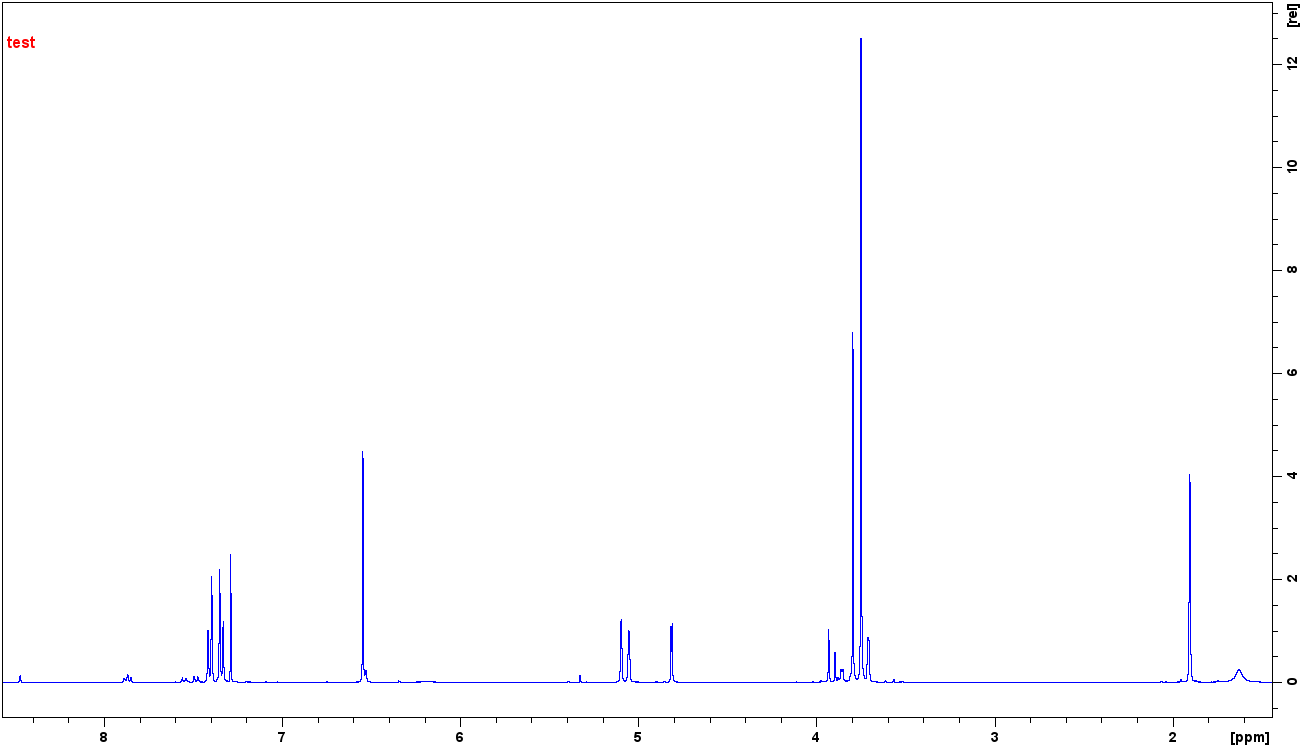

Supplement: Supplementary file 1 [file pharmaceuticals-16-01000-s001.zip › 9b mjm_15758_1h.png]

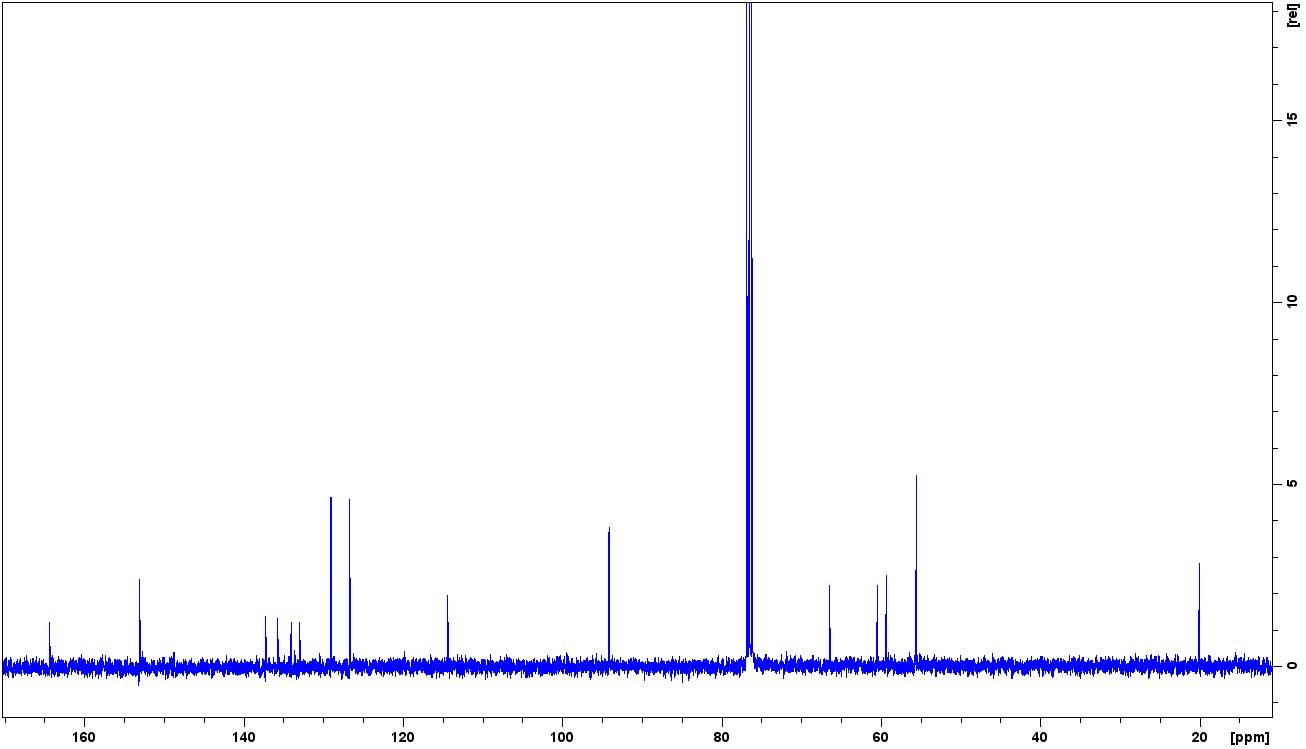

Supplement: Supplementary file 1 [file pharmaceuticals-16-01000-s001.zip › 9b mjm_15767_13c.png]

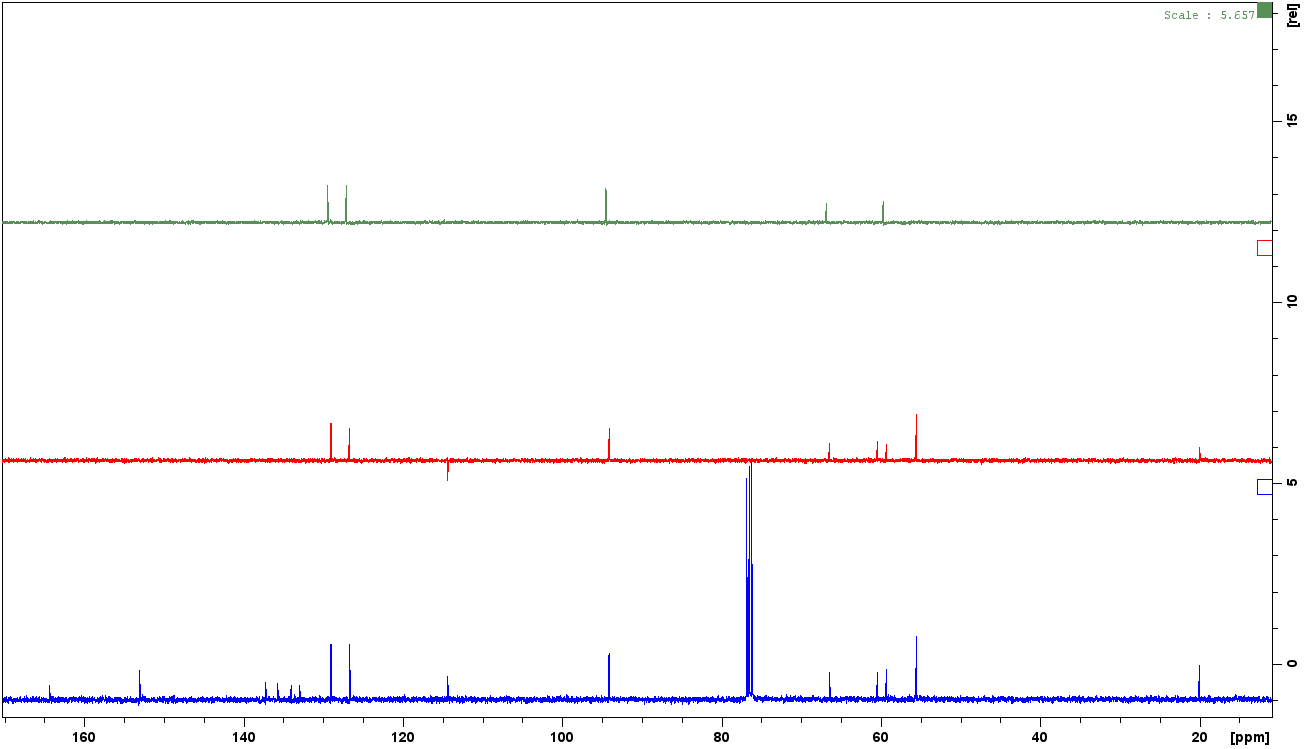

Supplement: Supplementary file 1 [file pharmaceuticals-16-01000-s001.zip › 9b mjm_15767_13c_DEPTs.png]

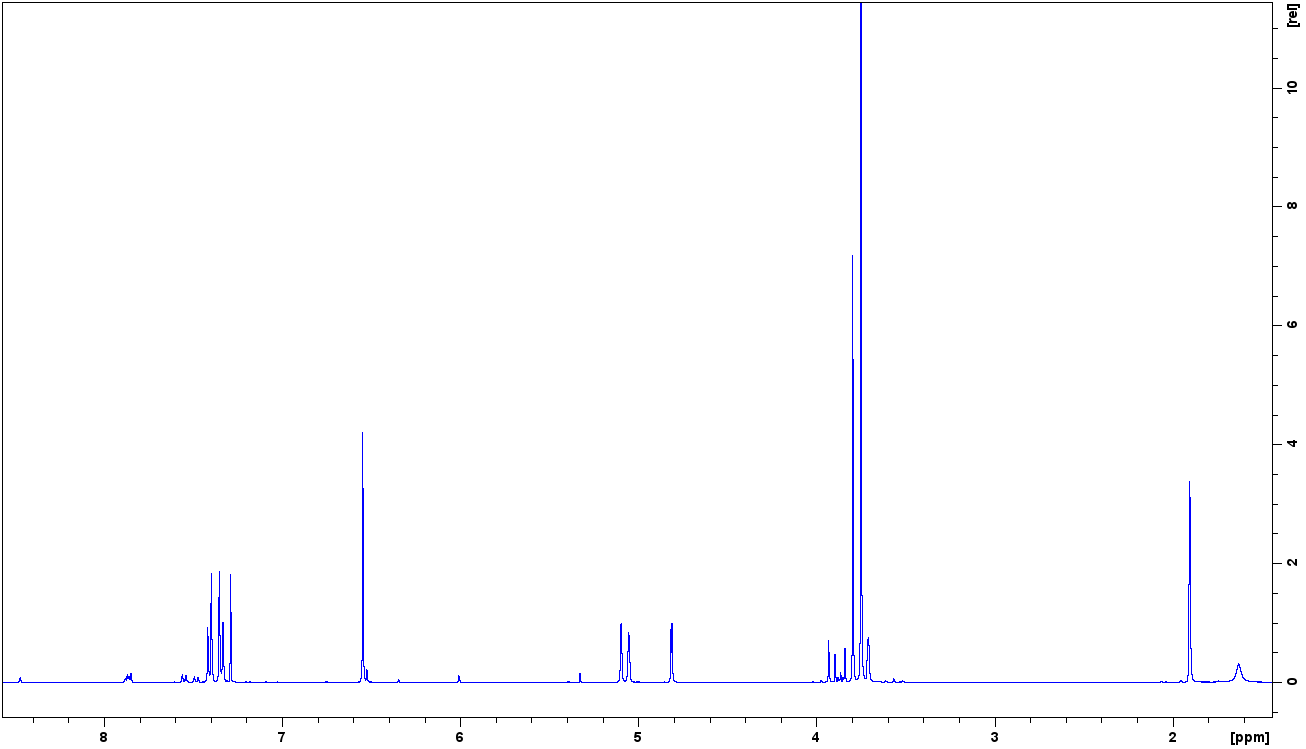

Supplement: Supplementary file 1 [file pharmaceuticals-16-01000-s001.zip › 9b mjm_15767_1h.png]

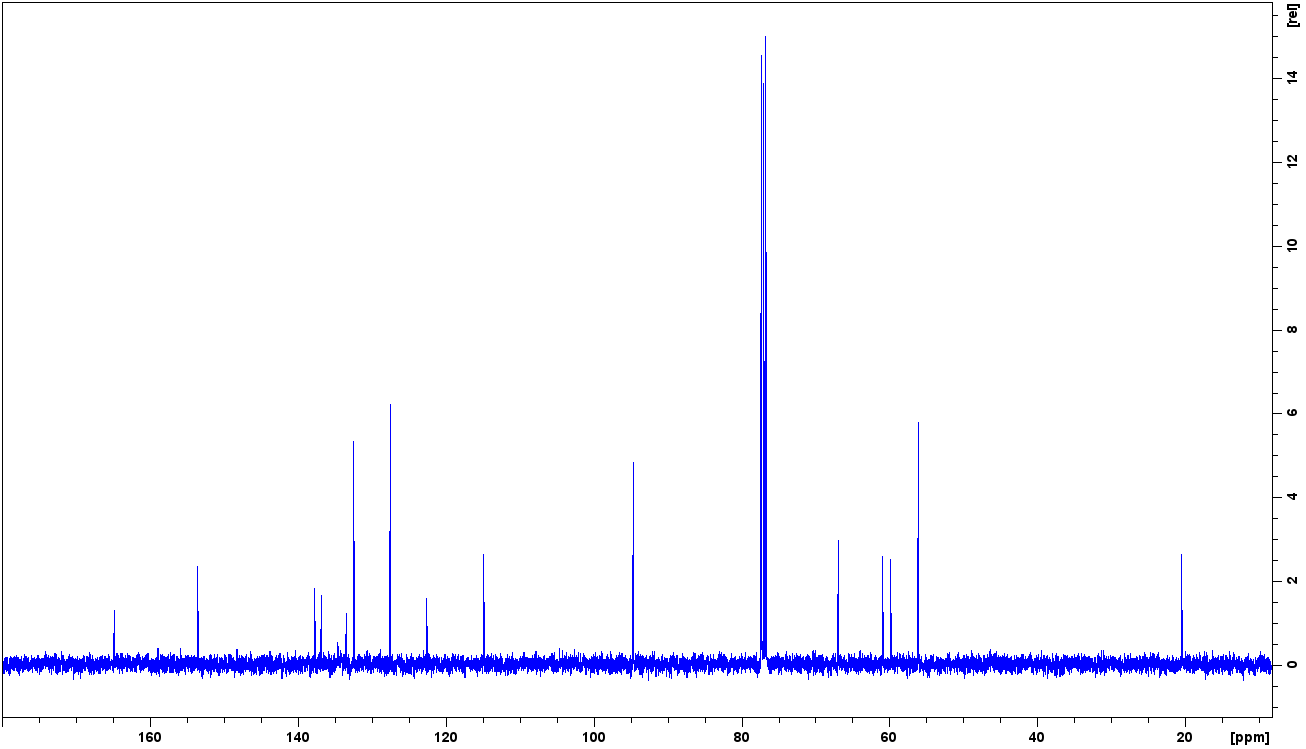

Supplement: Supplementary file 1 [file pharmaceuticals-16-01000-s001.zip › 9c mjm_15296_13c.png]

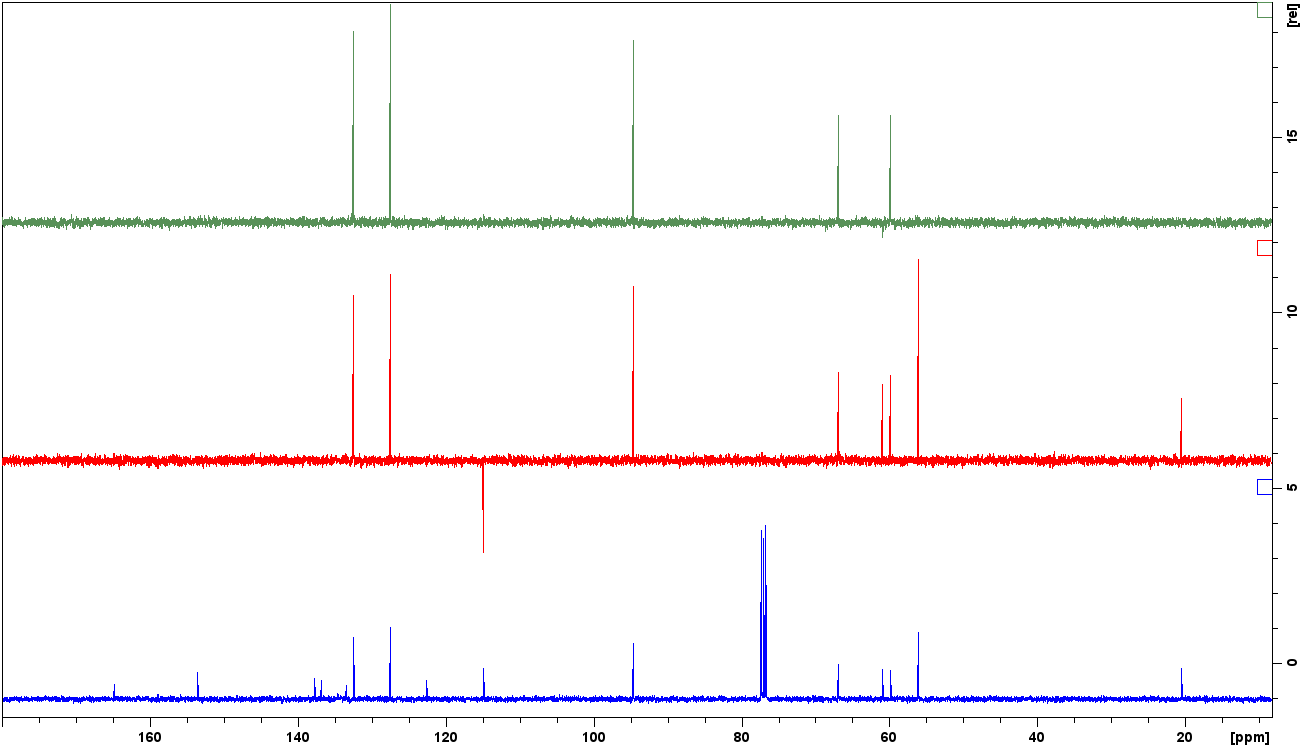

Supplement: Supplementary file 1 [file pharmaceuticals-16-01000-s001.zip › 9c mjm_15296_13c_DEPTs.png]

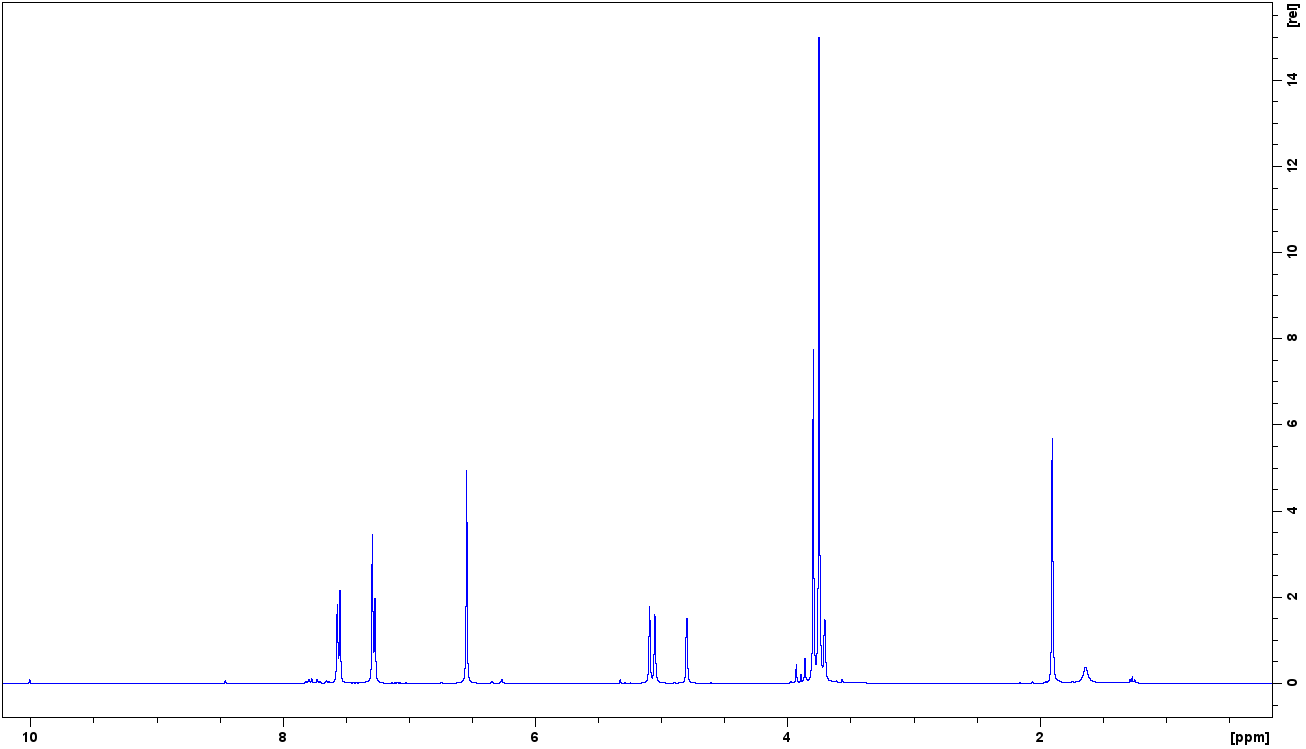

Supplement: Supplementary file 1 [file pharmaceuticals-16-01000-s001.zip › 9c mjm_15296_1h.png]

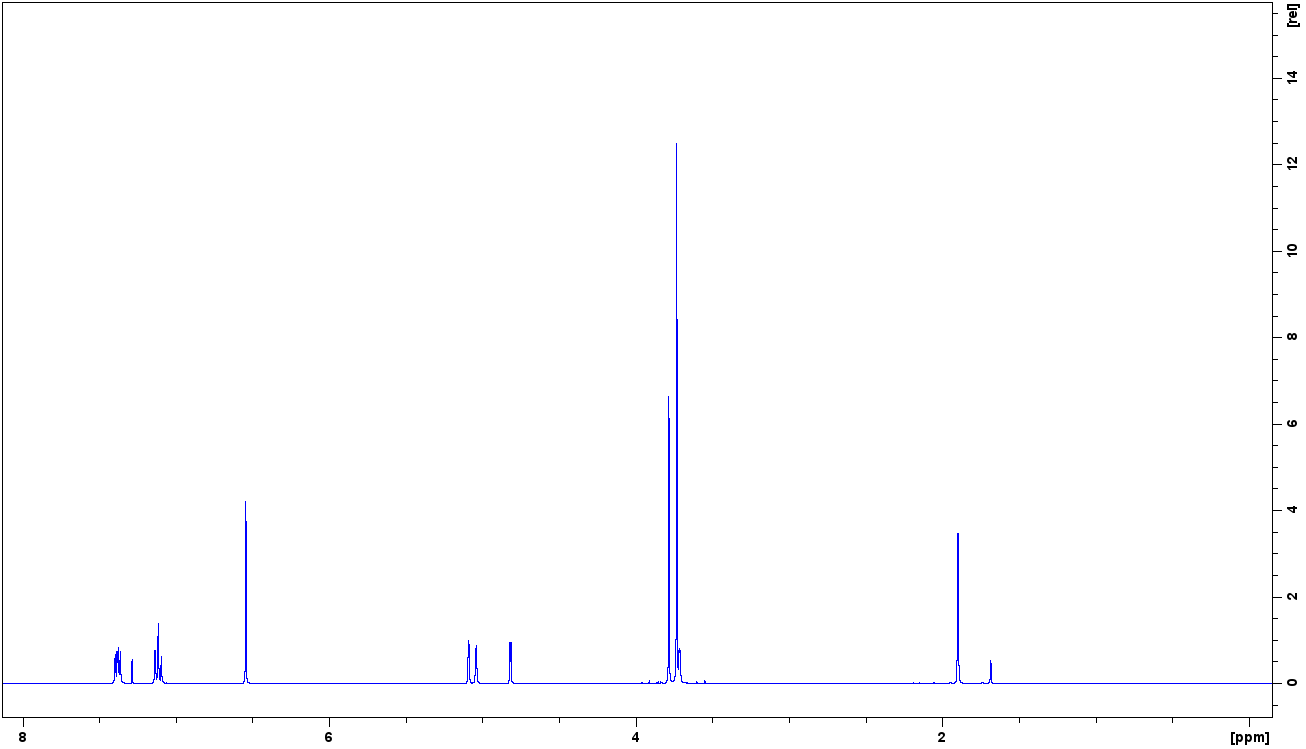

Supplement: Supplementary file 1 [file pharmaceuticals-16-01000-s001.zip › 9d mjm15437_1h.png]

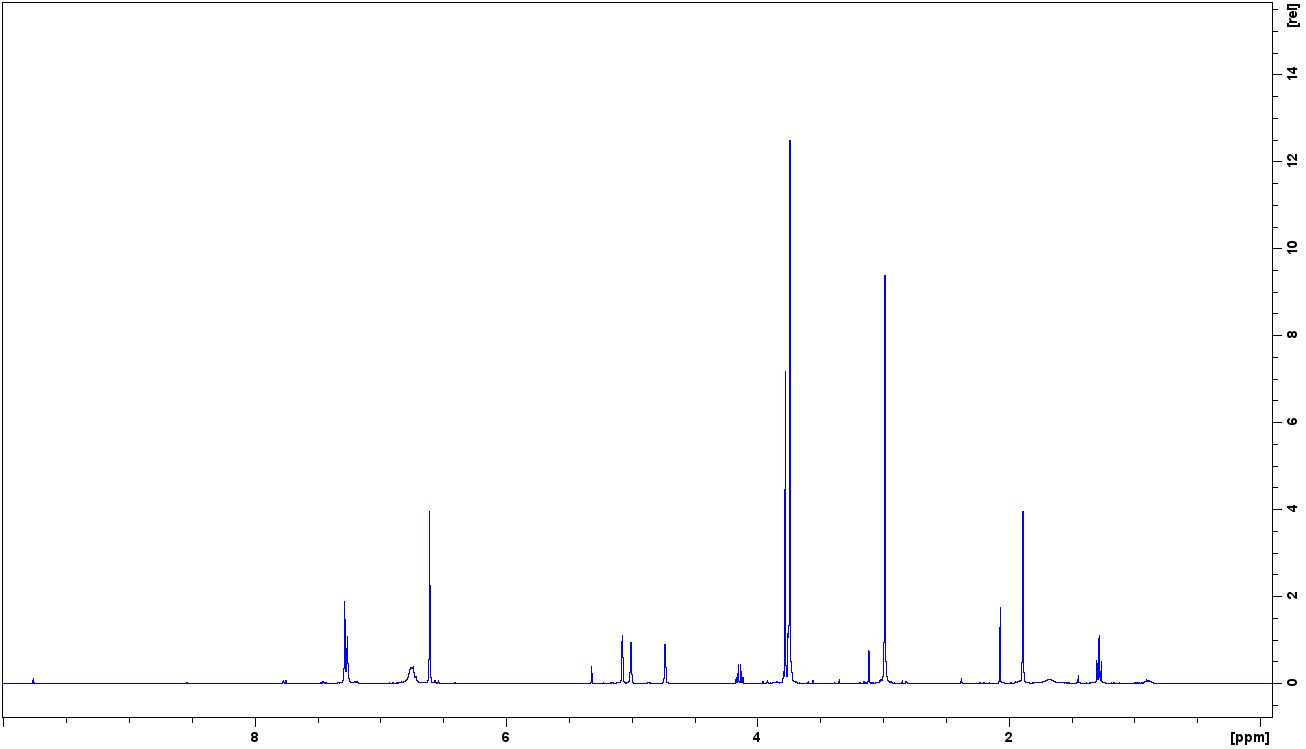

Supplement: Supplementary file 1 [file pharmaceuticals-16-01000-s001.zip › 9e mjm15375_1h.png]

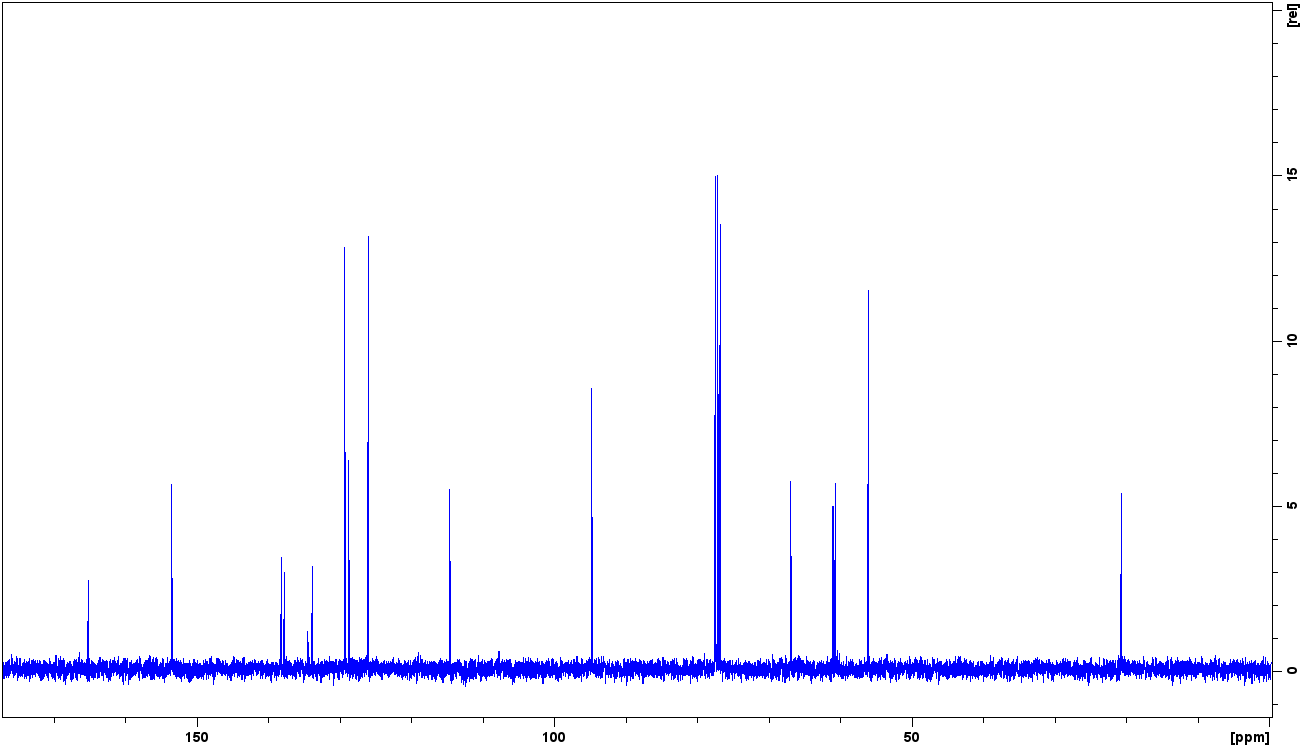

Supplement: Supplementary file 1 [file pharmaceuticals-16-01000-s001.zip › 9f mjm15493_13c.png]

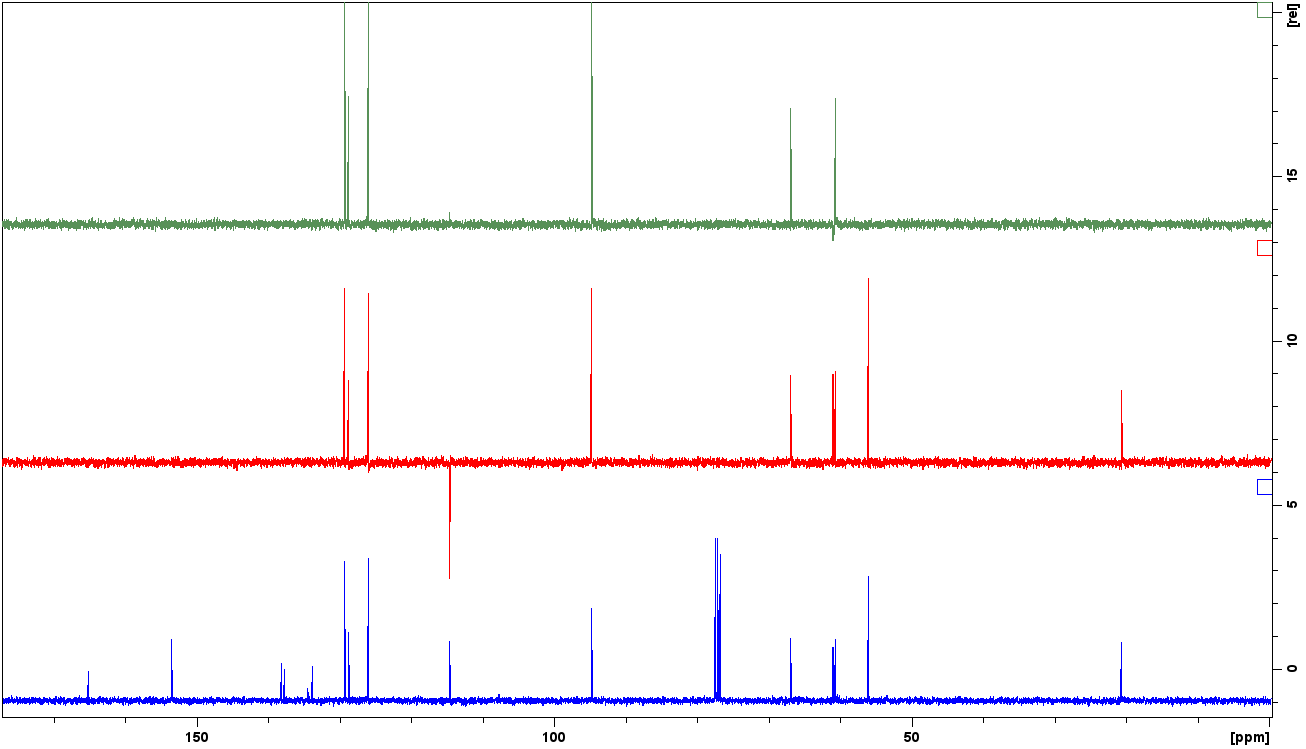

Supplement: Supplementary file 1 [file pharmaceuticals-16-01000-s001.zip › 9f mjm15493_13c_DEPTs.png]

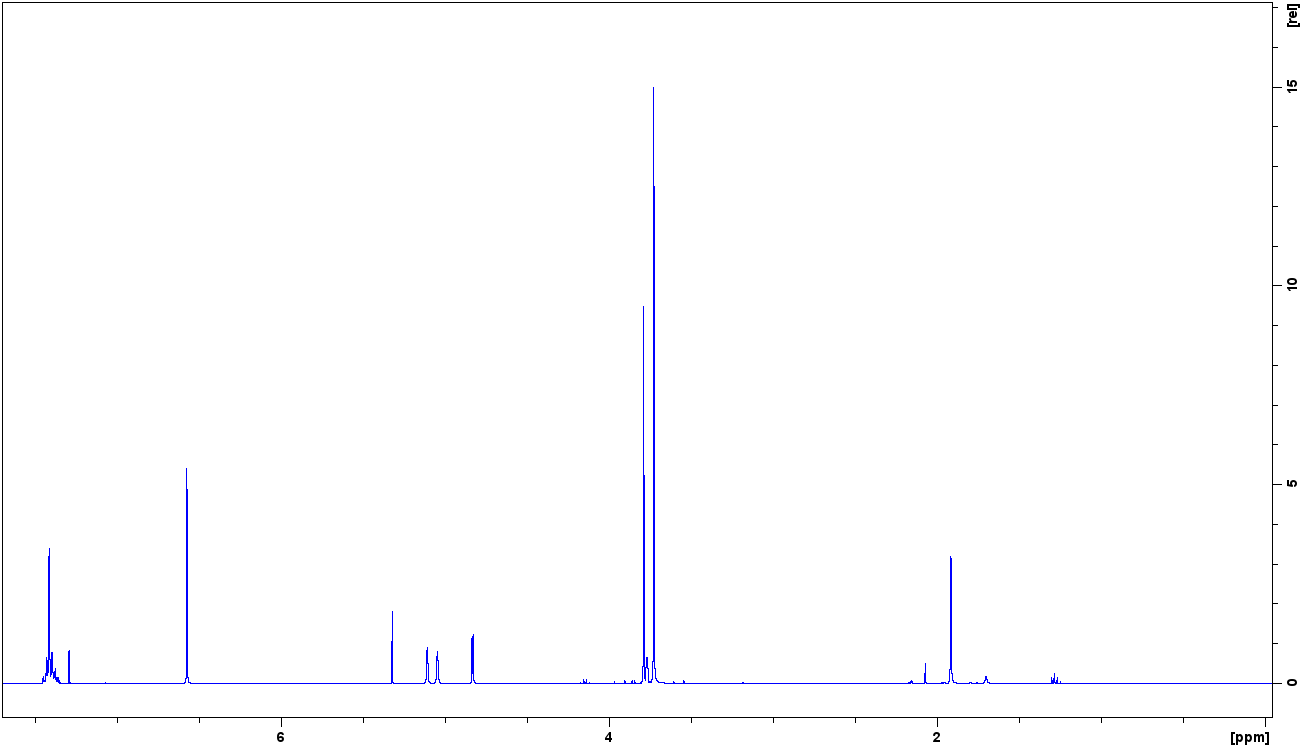

Supplement: Supplementary file 1 [file pharmaceuticals-16-01000-s001.zip › 9f mjm15493_1h.png]

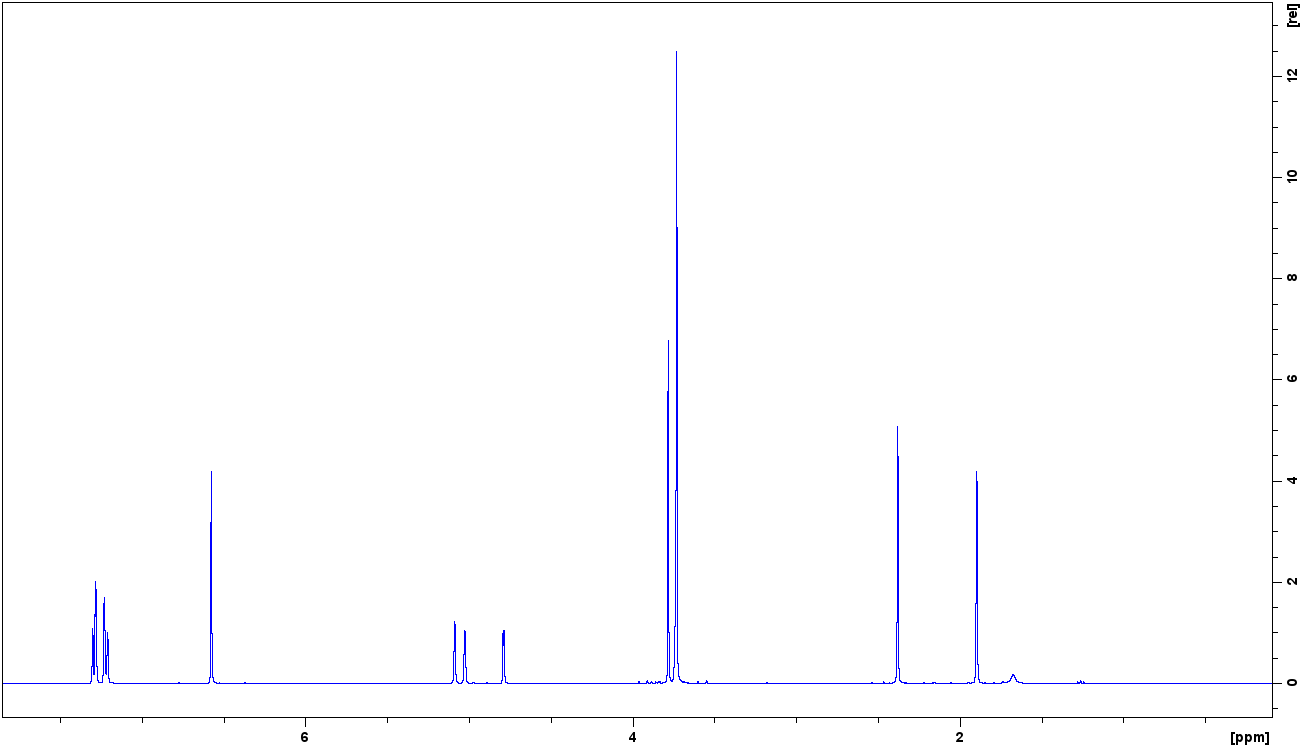

Supplement: Supplementary file 1 [file pharmaceuticals-16-01000-s001.zip › 9g mjm15620_1h.png]

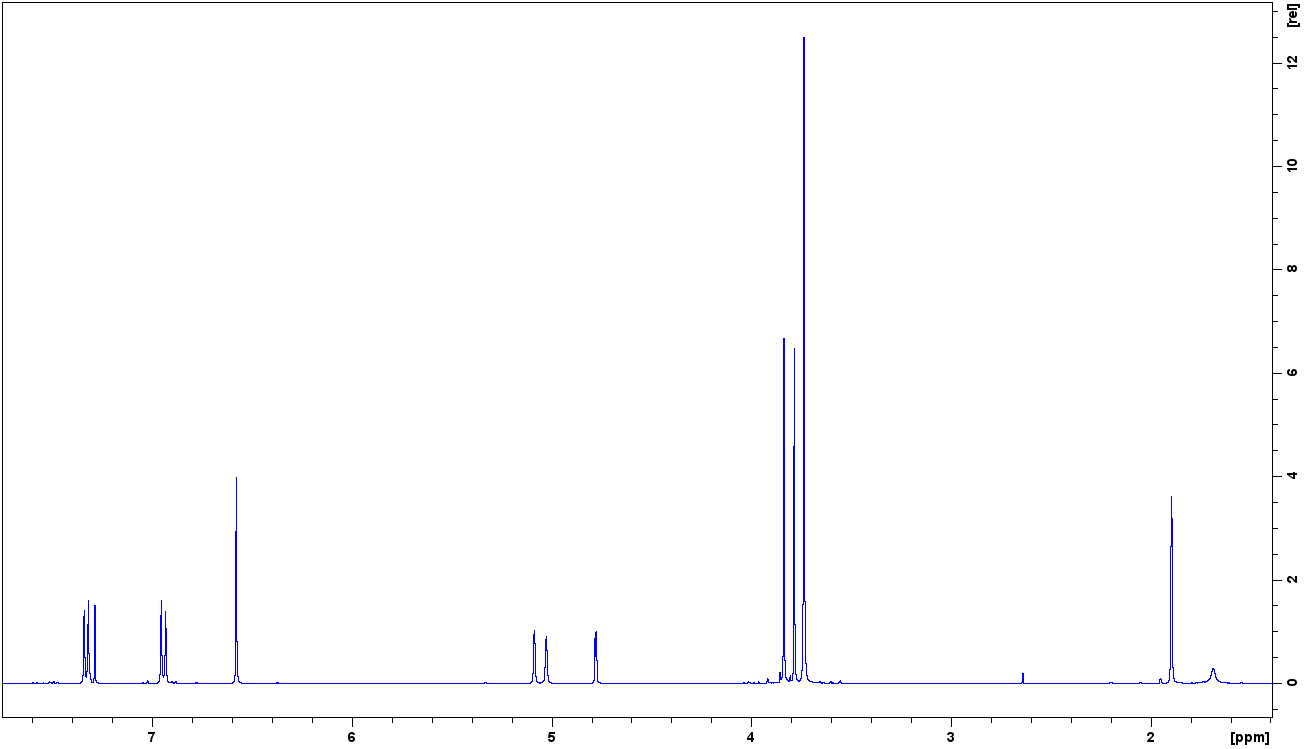

Supplement: Supplementary file 1 [file pharmaceuticals-16-01000-s001.zip › 9h mjm16640_1h.png]

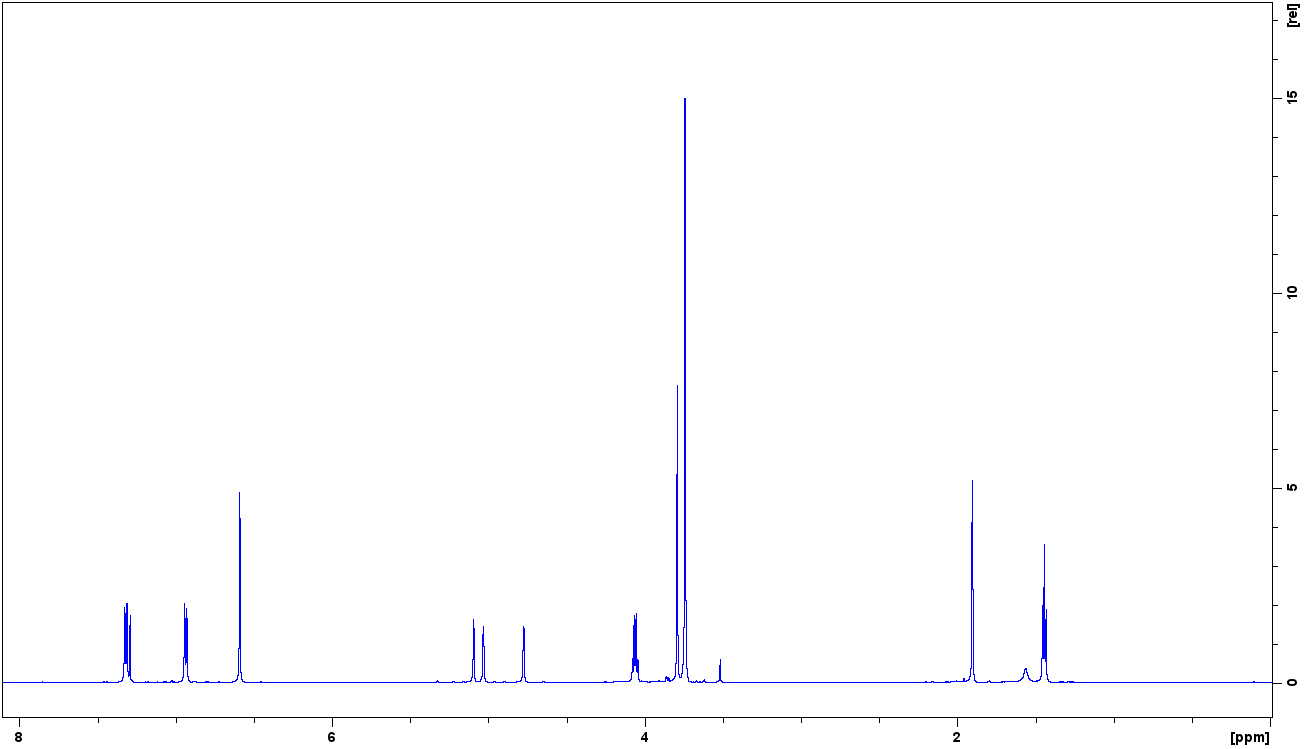

Supplement: Supplementary file 1 [file pharmaceuticals-16-01000-s001.zip › 9i imjm15421_1h.png]

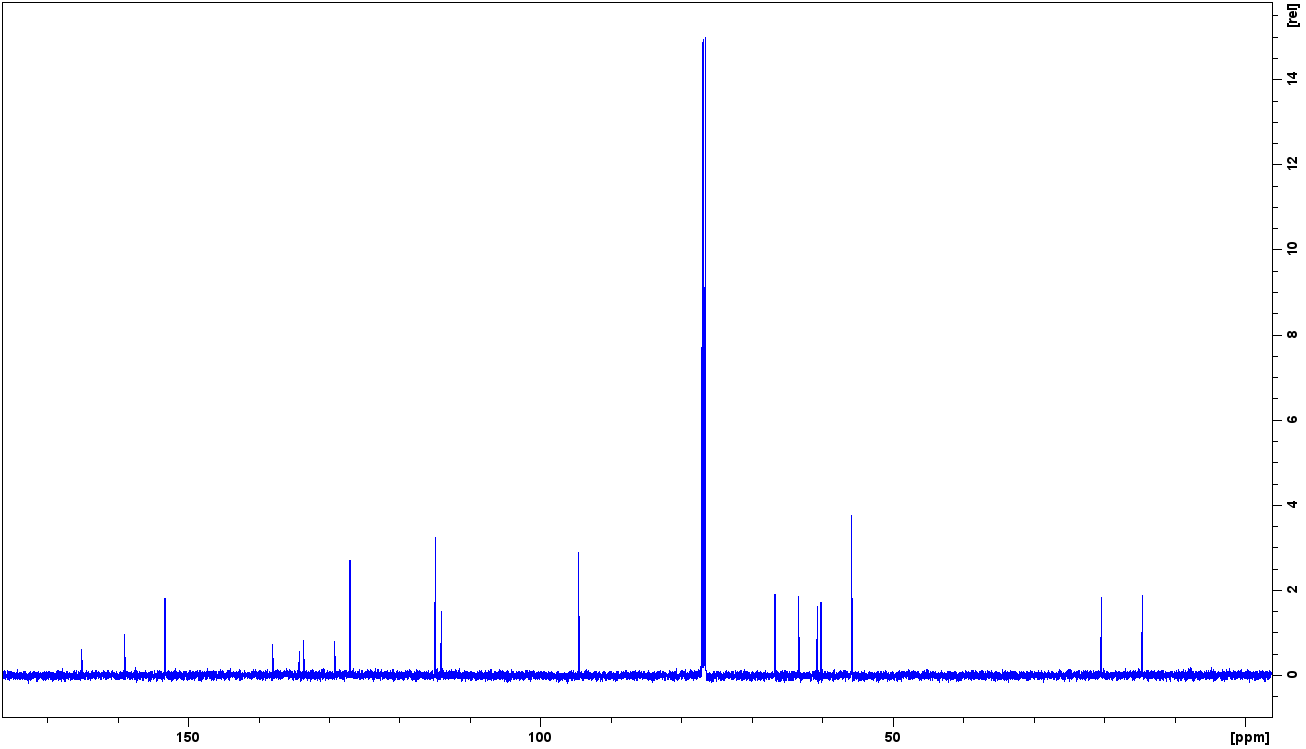

Supplement: Supplementary file 1 [file pharmaceuticals-16-01000-s001.zip › 9i mjm15421_13c.png]

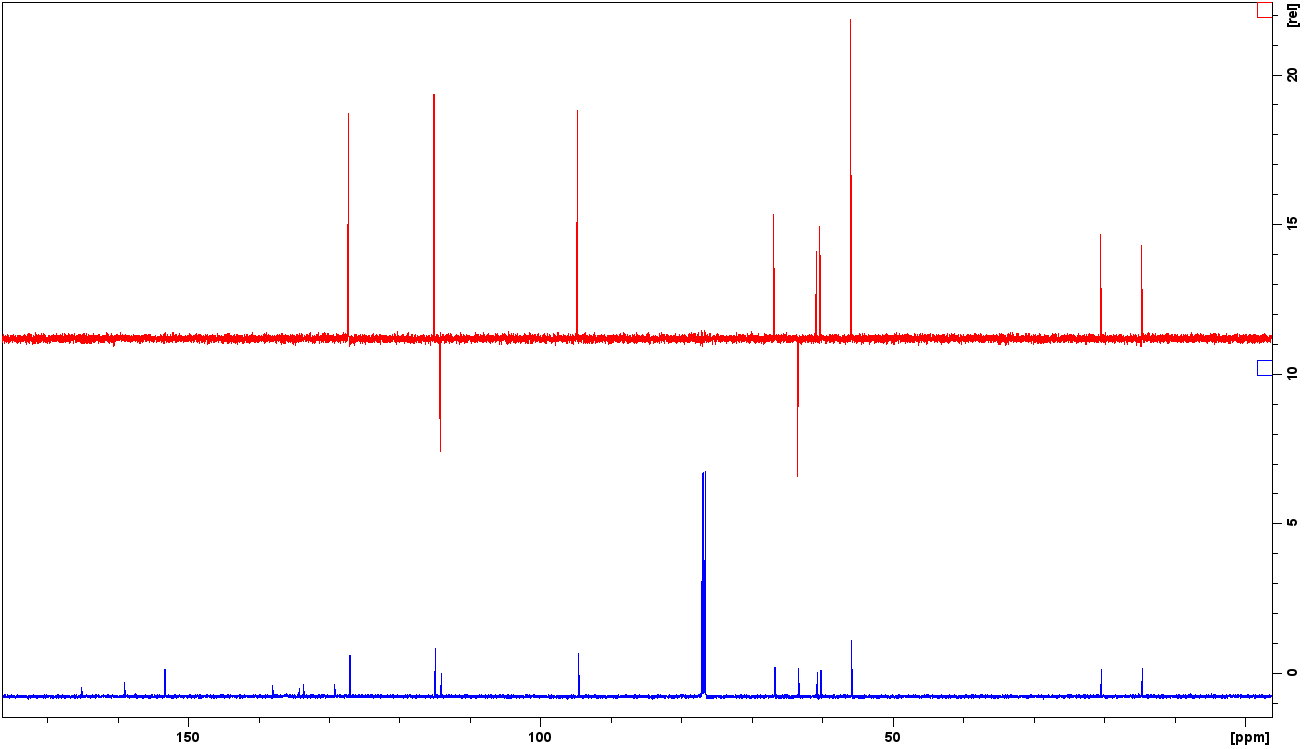

Supplement: Supplementary file 1 [file pharmaceuticals-16-01000-s001.zip › 9i mjm15421_13c_DEPT.png]

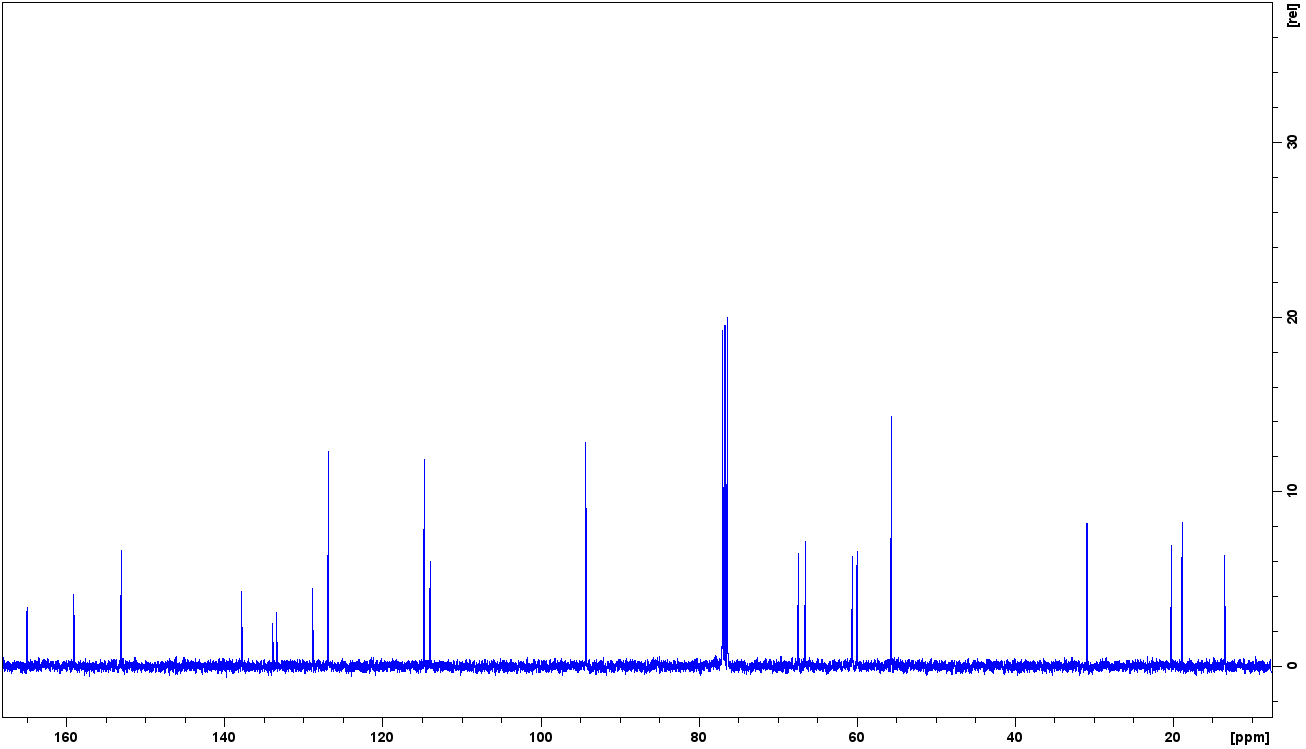

Supplement: Supplementary file 1 [file pharmaceuticals-16-01000-s001.zip › 9j mjm_16541_13c.png]

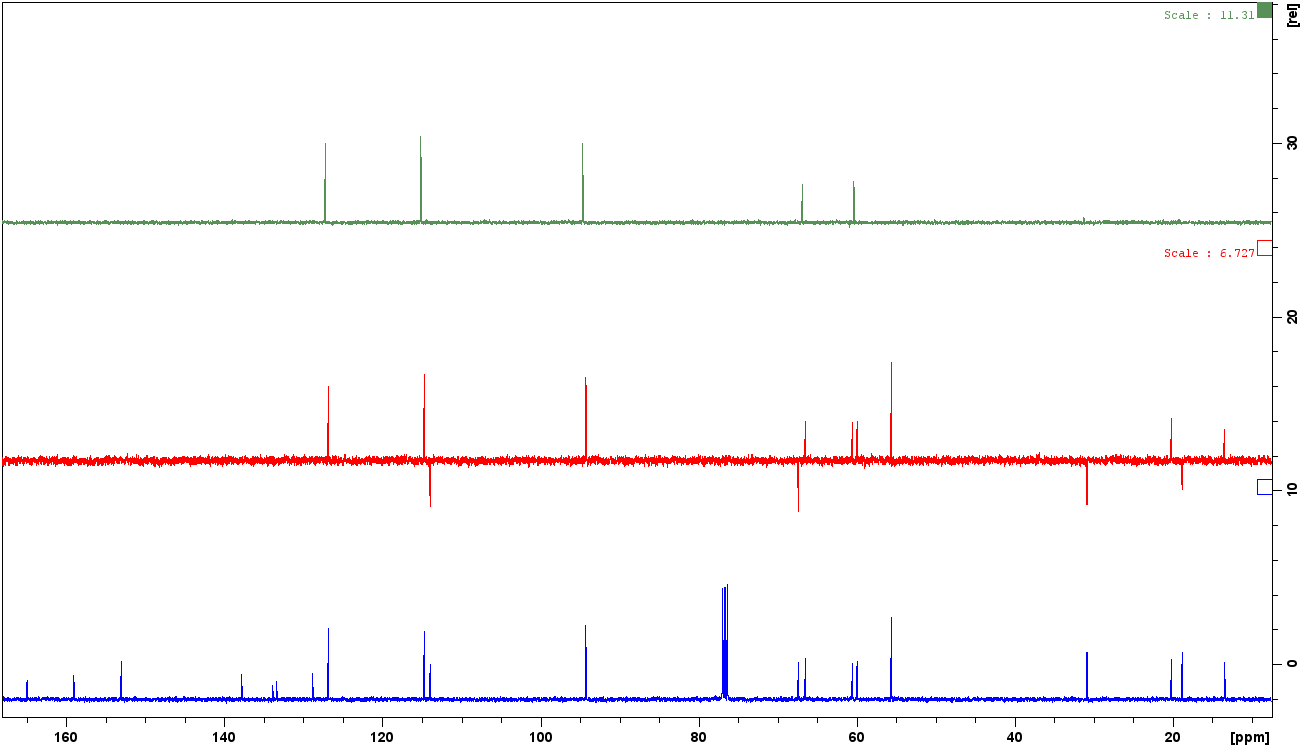

Supplement: Supplementary file 1 [file pharmaceuticals-16-01000-s001.zip › 9j mjm_16541_13c_DEPTs.png]

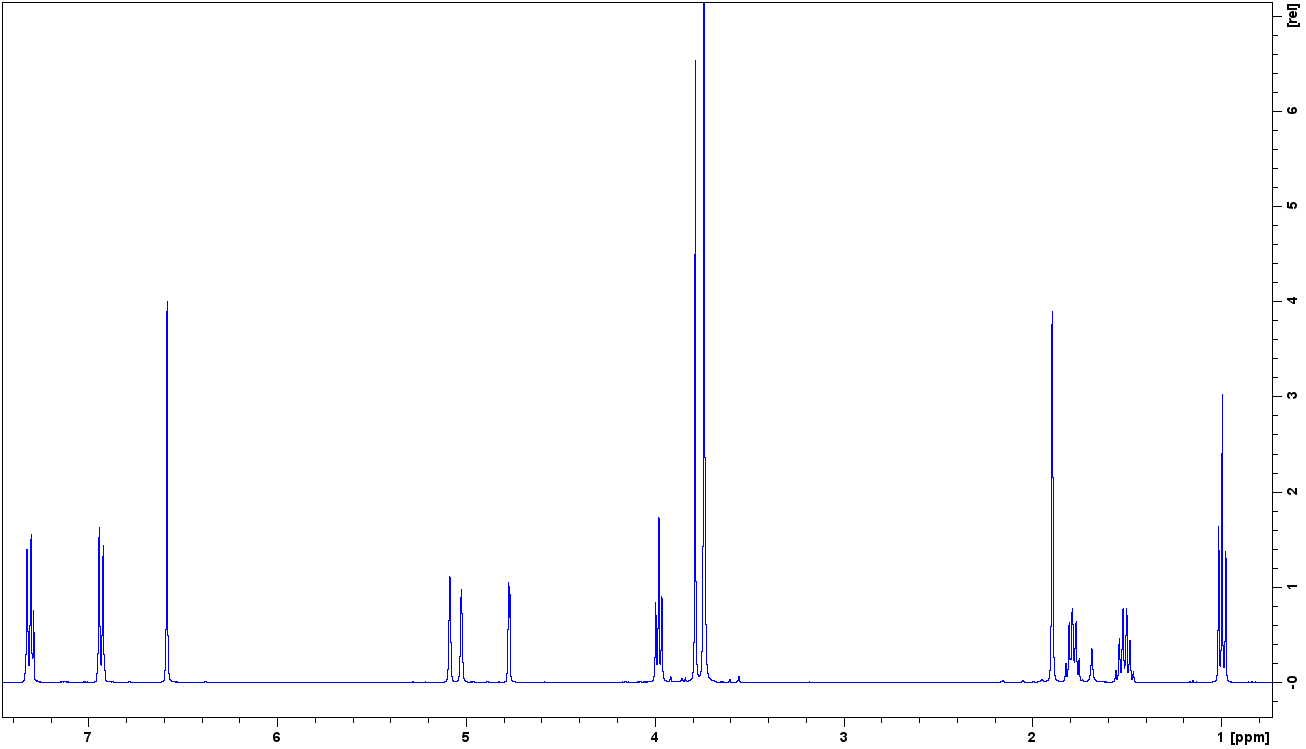

Supplement: Supplementary file 1 [file pharmaceuticals-16-01000-s001.zip › 9j mjm_16541_1h.png]

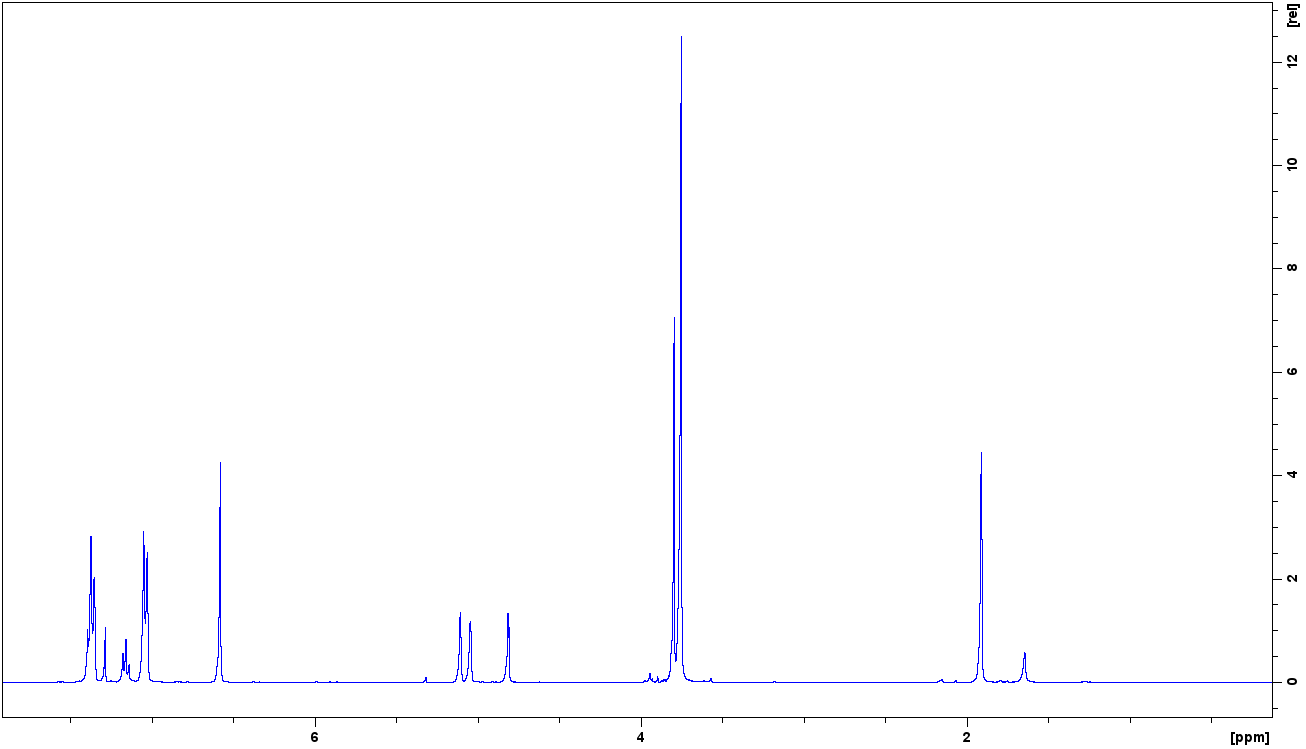

Supplement: Supplementary file 1 [file pharmaceuticals-16-01000-s001.zip › 9k mjm15650_1h.png]

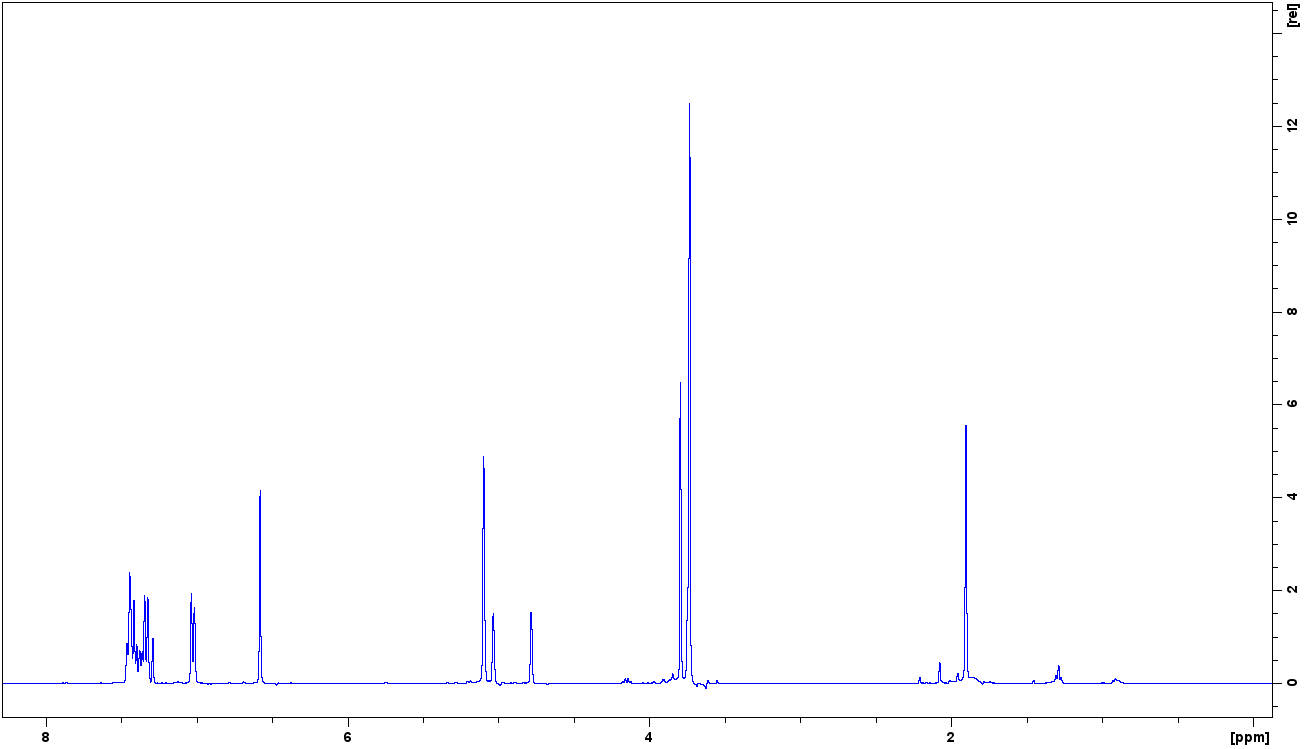

Supplement: Supplementary file 1 [file pharmaceuticals-16-01000-s001.zip › 9l mjm15388_1h.png]

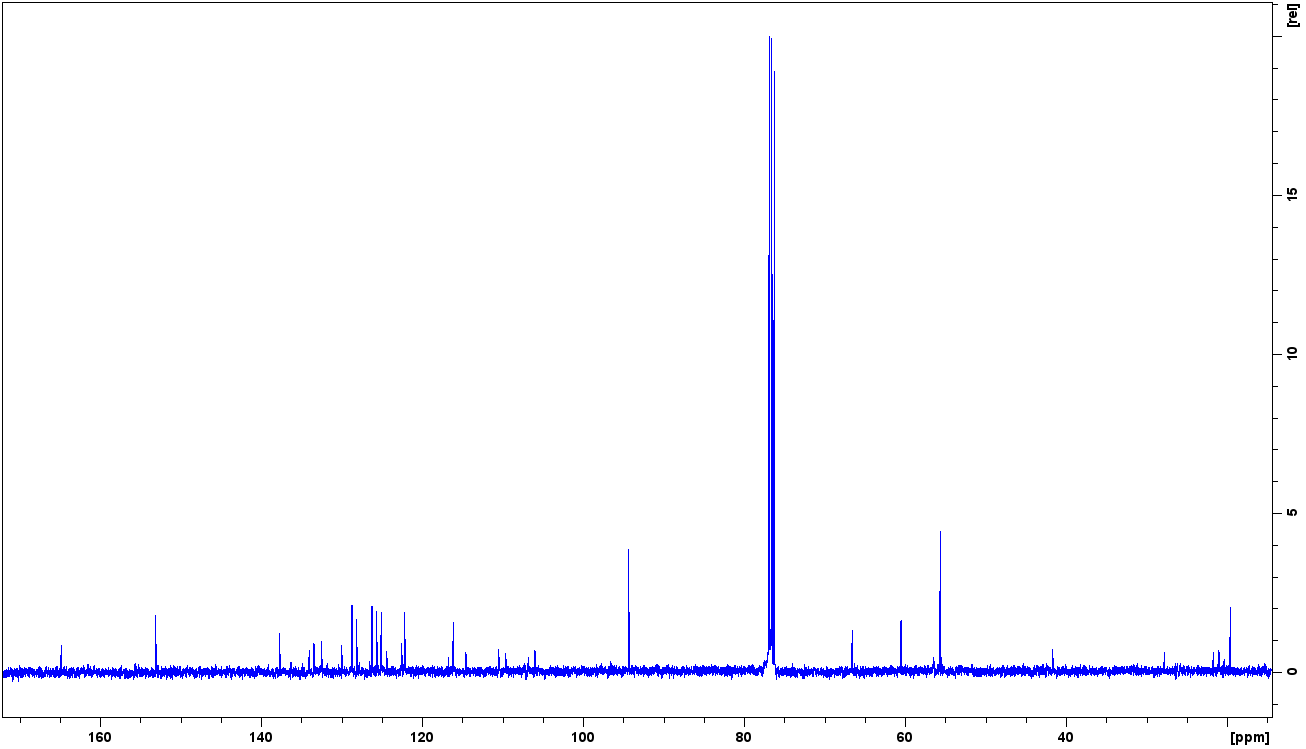

Supplement: Supplementary file 1 [file pharmaceuticals-16-01000-s001.zip › 9m mjm16578_13c.png]

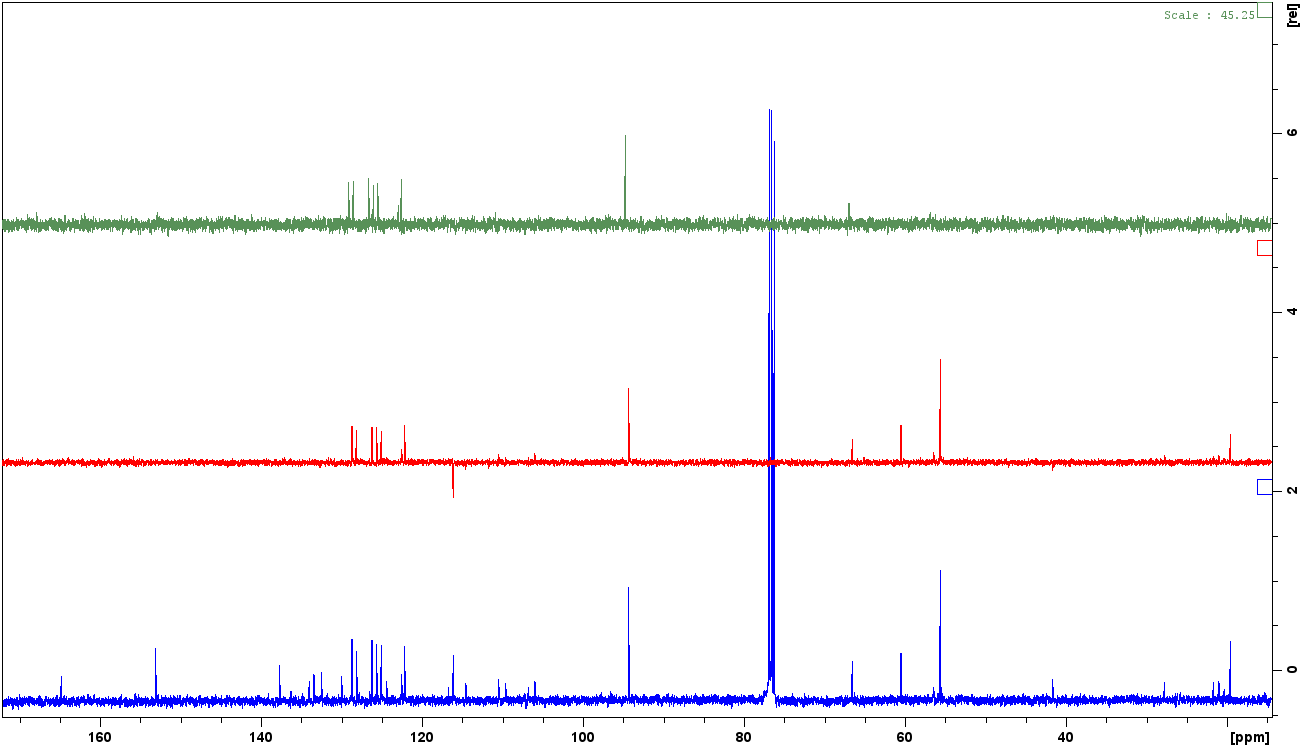

Supplement: Supplementary file 1 [file pharmaceuticals-16-01000-s001.zip › 9m mjm16578_13c_DEPT.png]

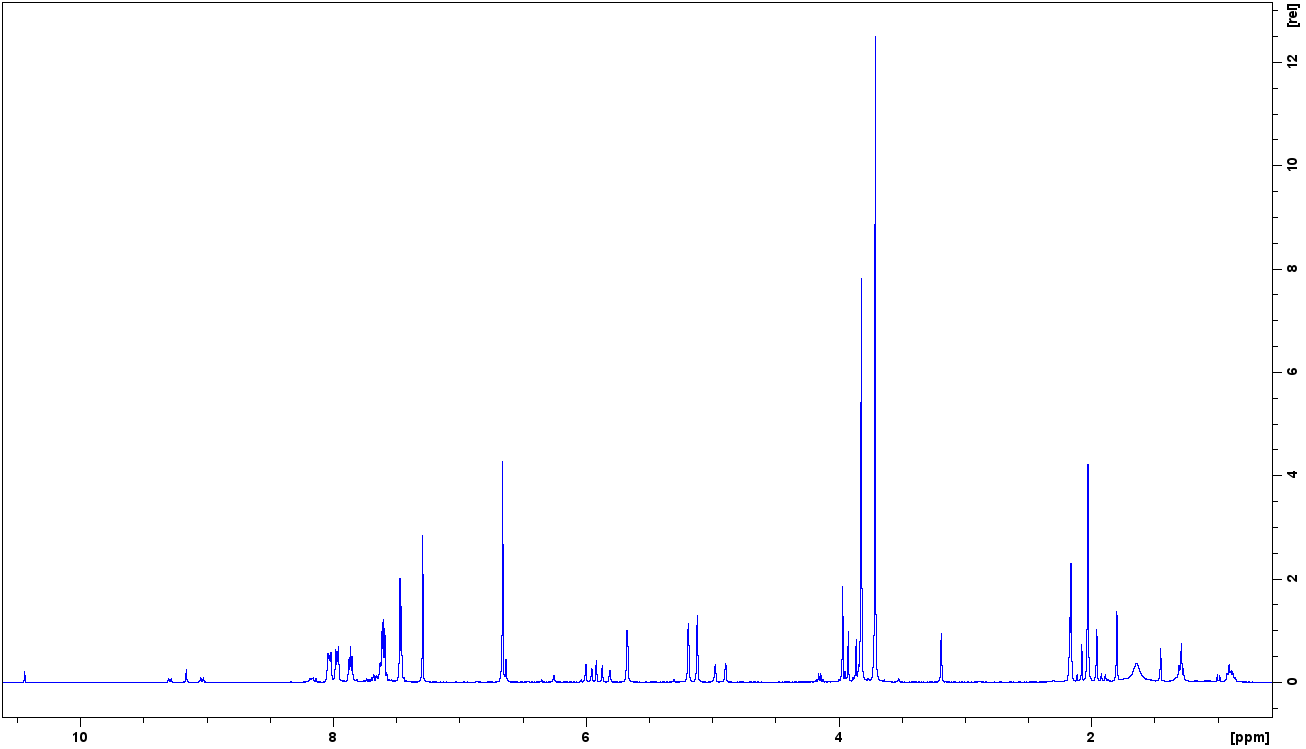

Supplement: Supplementary file 1 [file pharmaceuticals-16-01000-s001.zip › 9m mjm16578_1h.png]

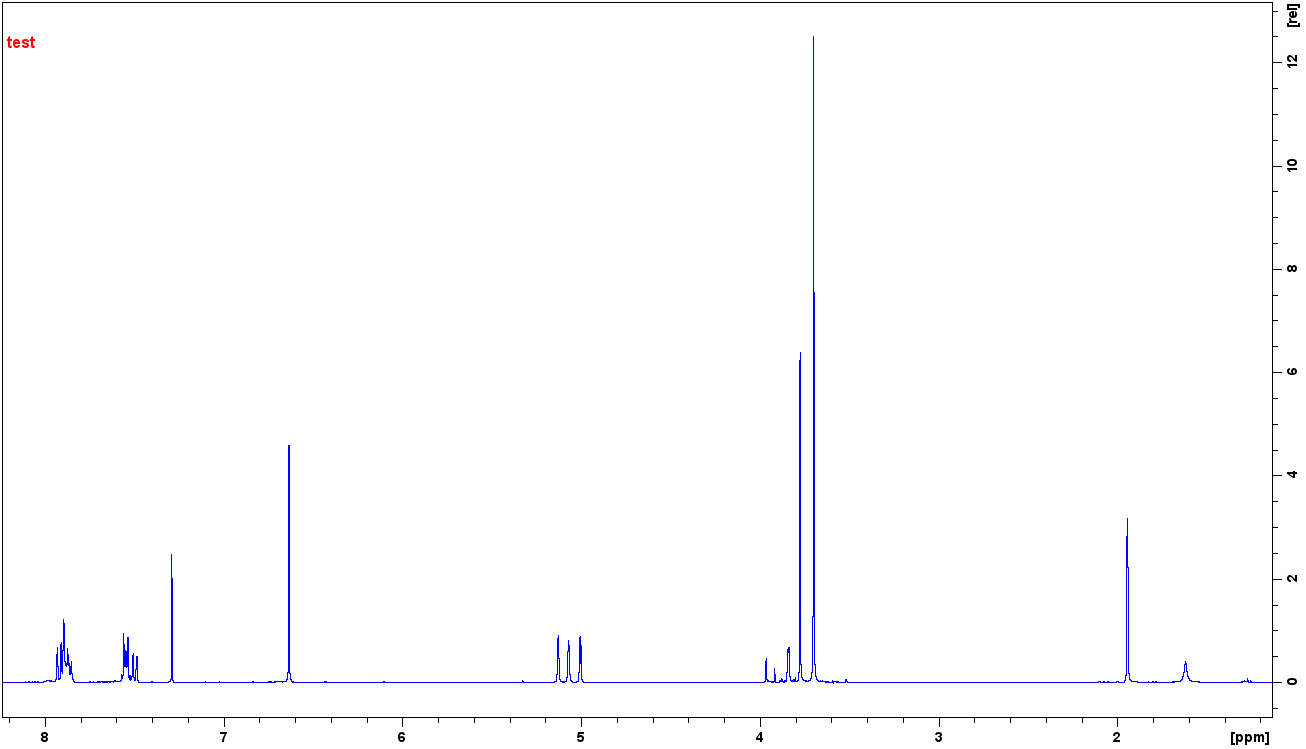

Supplement: Supplementary file 1 [file pharmaceuticals-16-01000-s001.zip › 9n mjm_15644_1h.png]

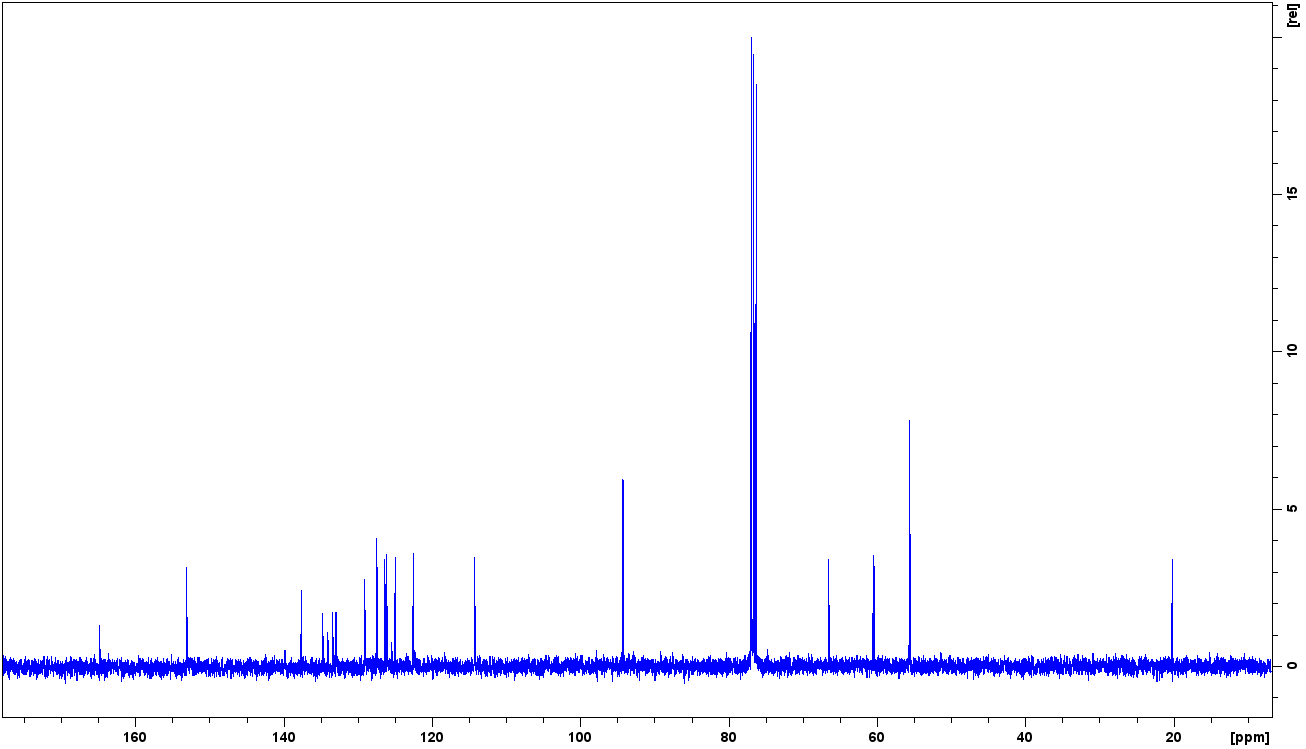

Supplement: Supplementary file 1 [file pharmaceuticals-16-01000-s001.zip › 9n mjm_15647_13c.png]

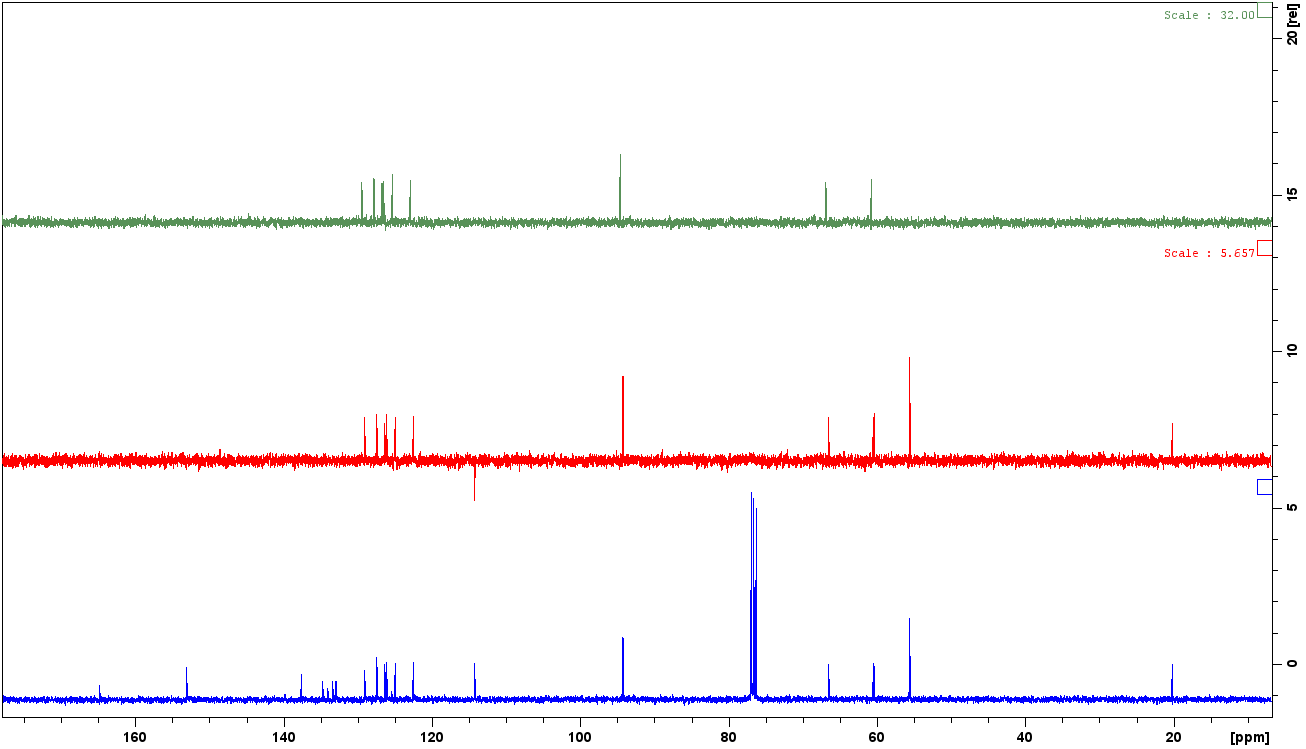

Supplement: Supplementary file 1 [file pharmaceuticals-16-01000-s001.zip › 9n mjm_15647_13c_DEPTs.png]

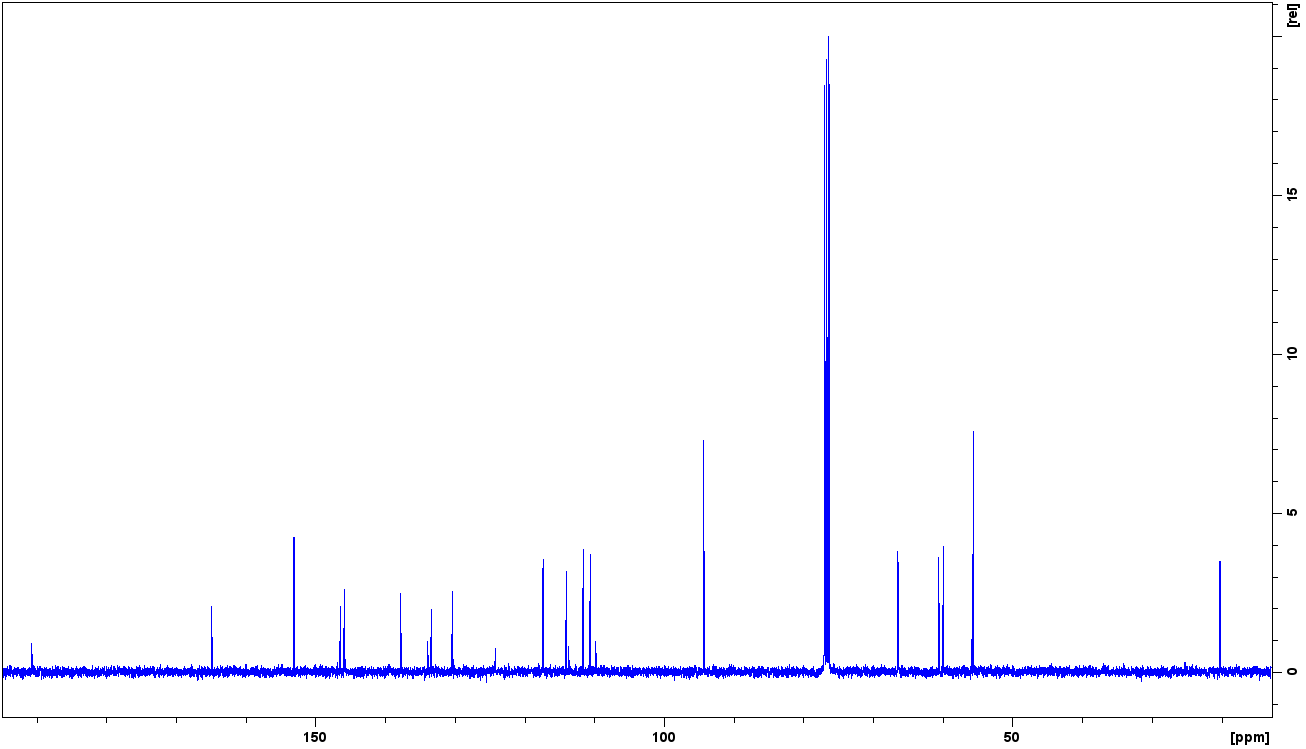

Supplement: Supplementary file 1 [file pharmaceuticals-16-01000-s001.zip › 9q mjm16542_13c.png]

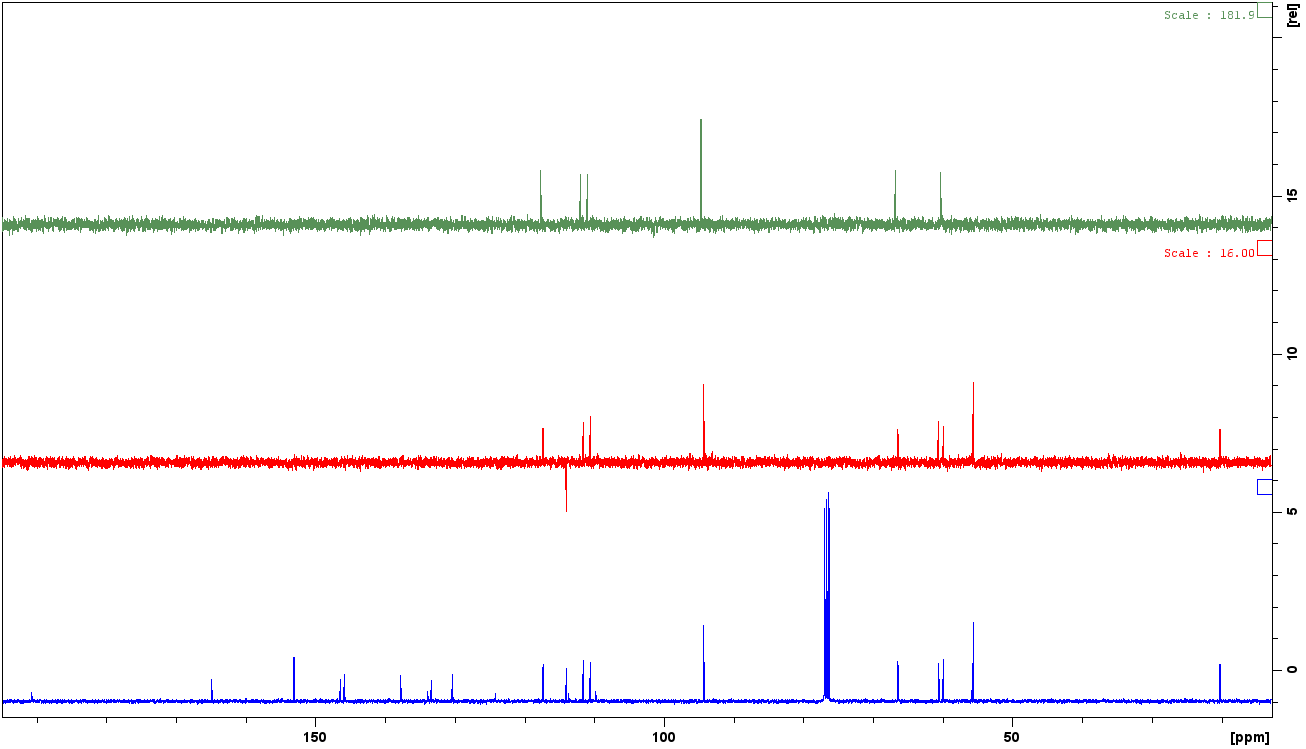

Supplement: Supplementary file 1 [file pharmaceuticals-16-01000-s001.zip › 9q mjm16542_13c_DEPTs.png]

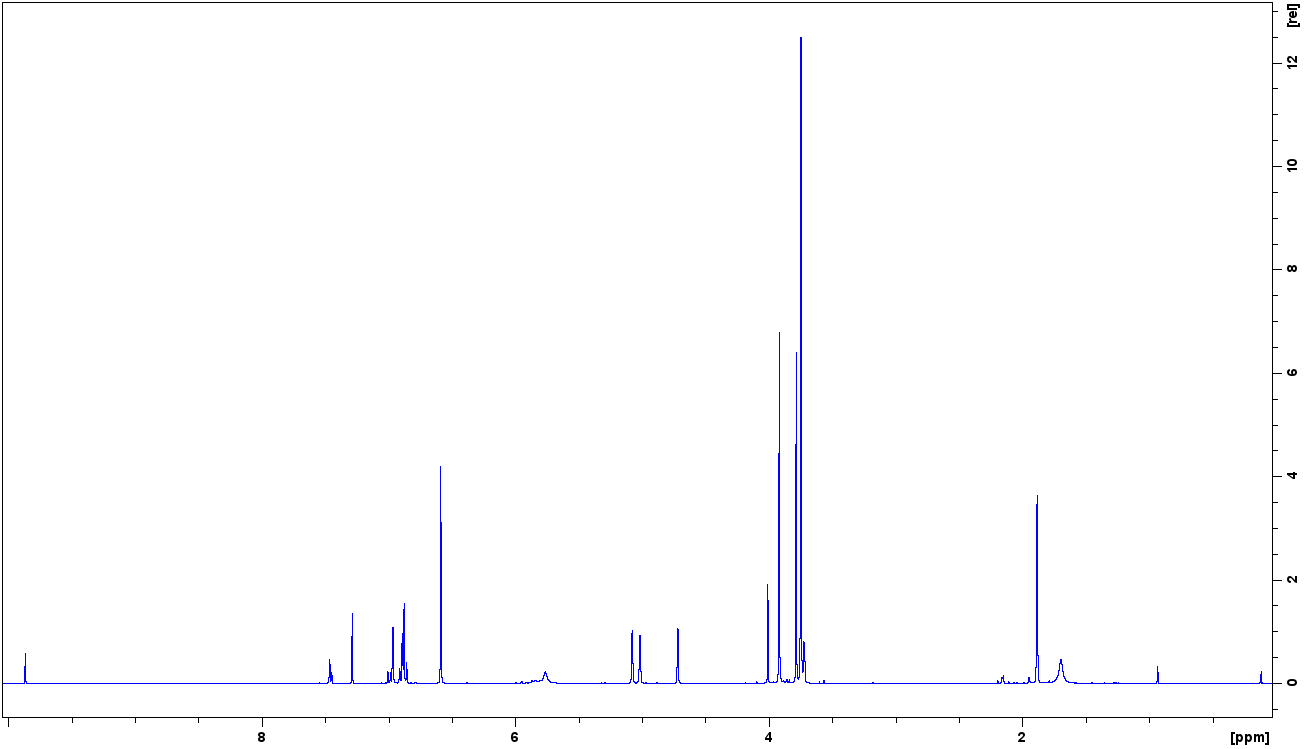

Supplement: Supplementary file 1 [file pharmaceuticals-16-01000-s001.zip › 9q mjm16542_1h.png]

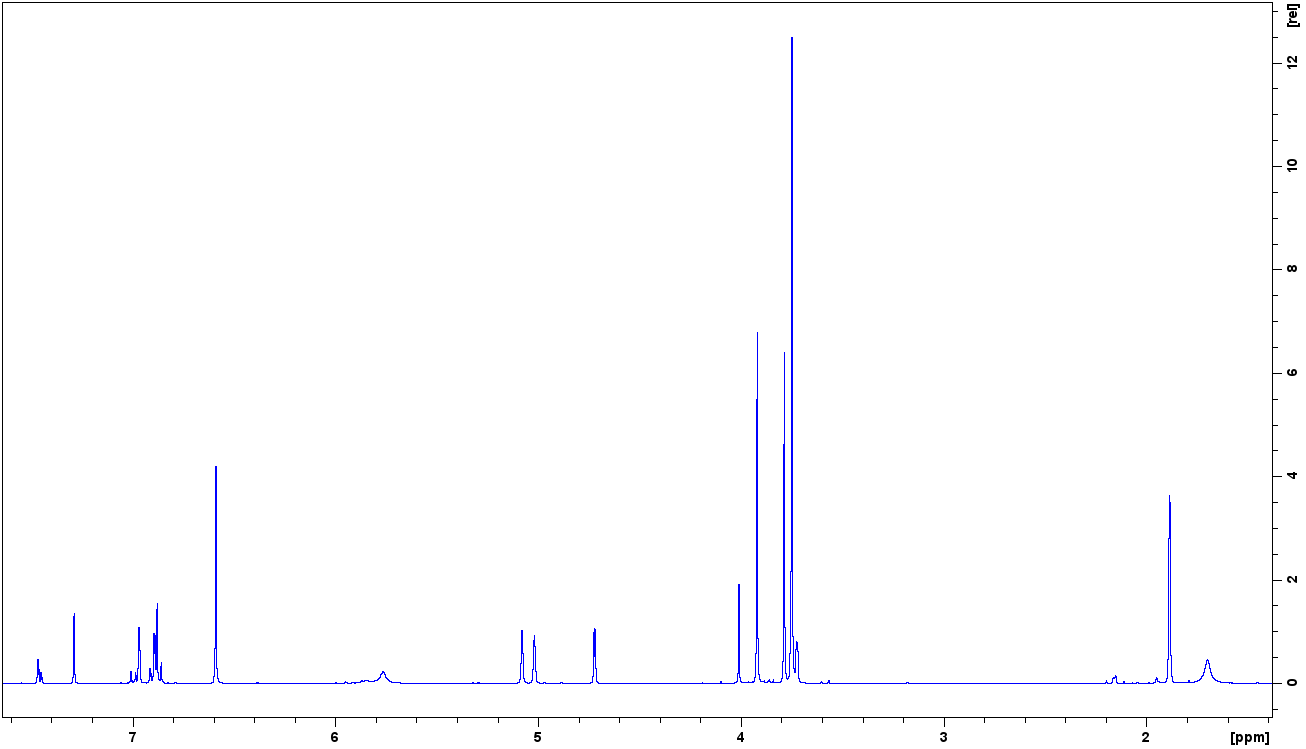

Supplement: Supplementary file 1 [file pharmaceuticals-16-01000-s001.zip › 9q mjm16542_1h_2.png]

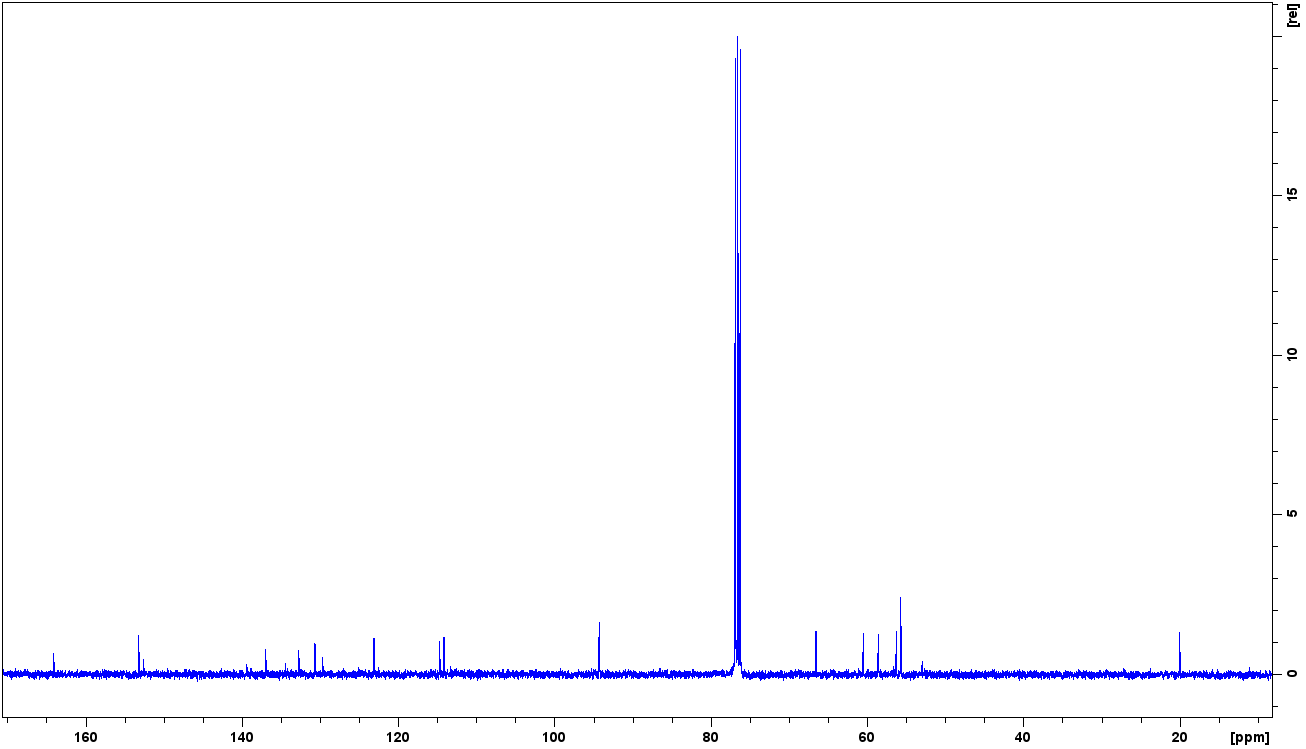

Supplement: Supplementary file 1 [file pharmaceuticals-16-01000-s001.zip › 9r mjm16503_13c.png]

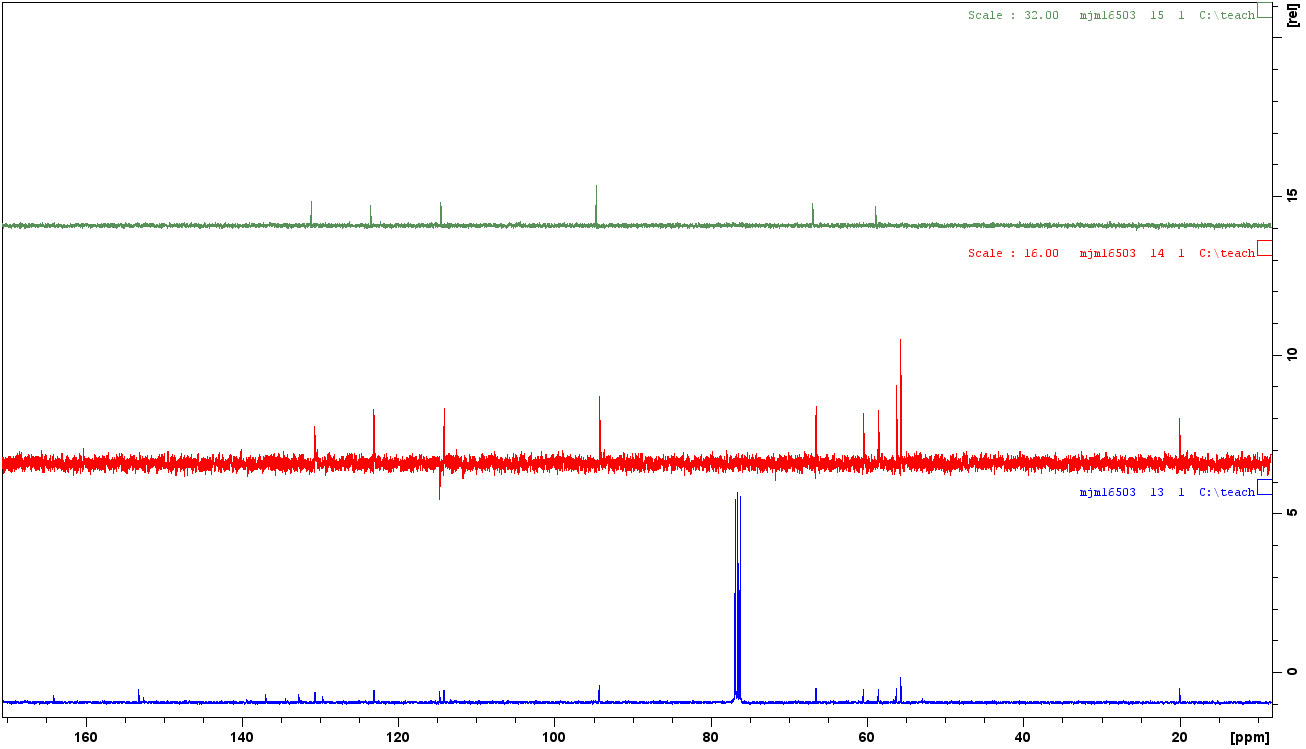

Supplement: Supplementary file 1 [file pharmaceuticals-16-01000-s001.zip › 9r mjm16503_13c_DEPTs.png]

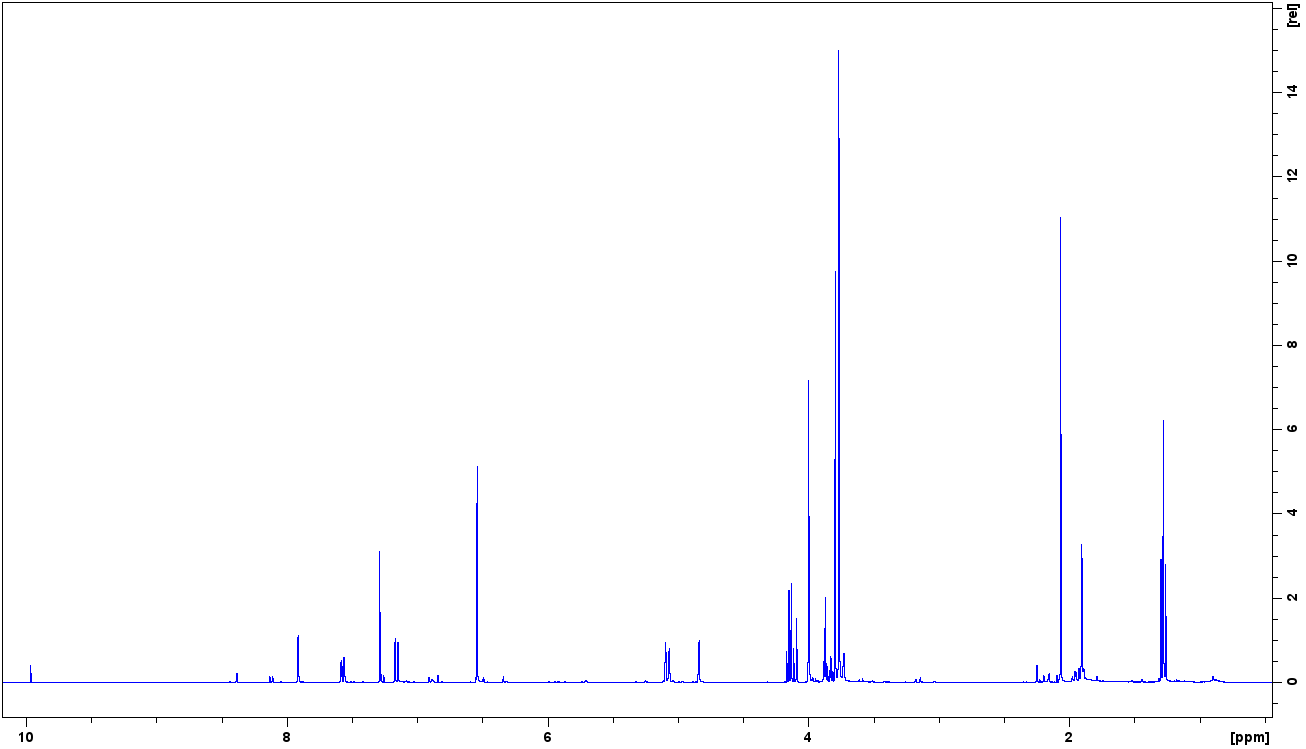

Supplement: Supplementary file 1 [file pharmaceuticals-16-01000-s001.zip › 9r mjm16503_1h.png]

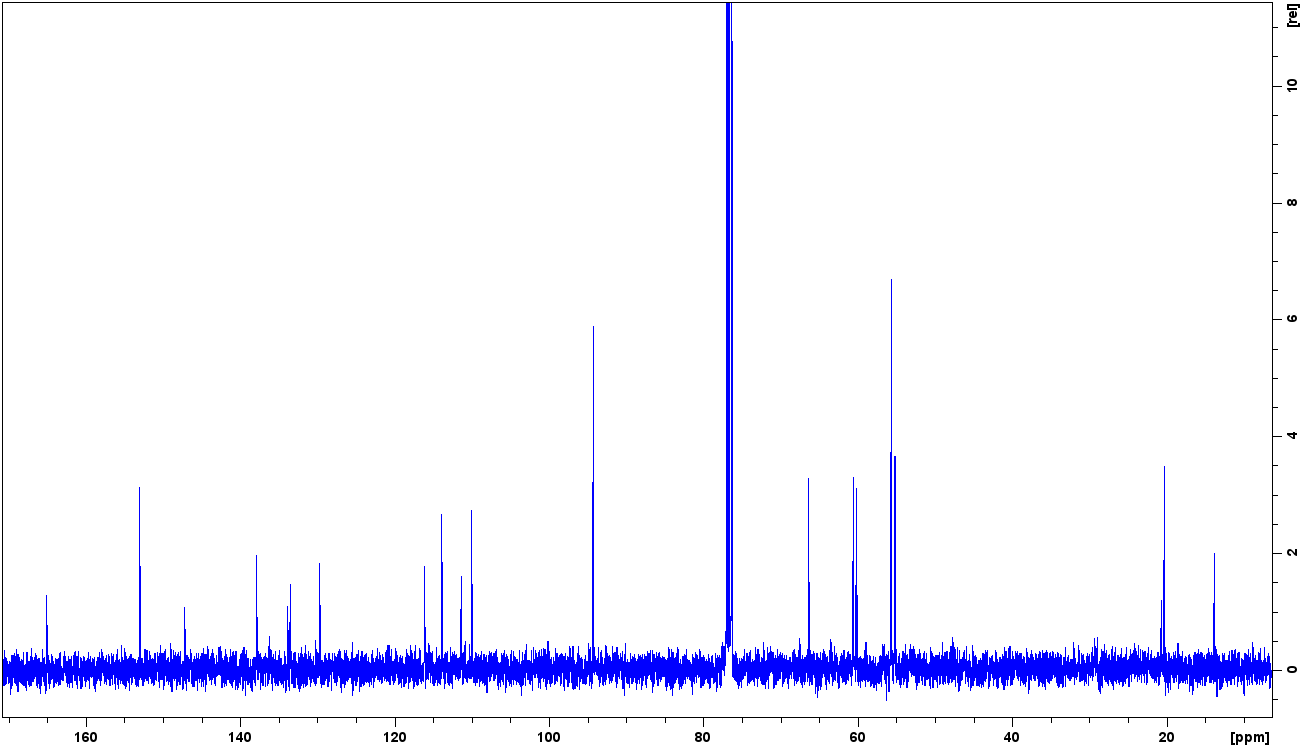

Supplement: Supplementary file 1 [file pharmaceuticals-16-01000-s001.zip › 9s mjm16533_13c.png]

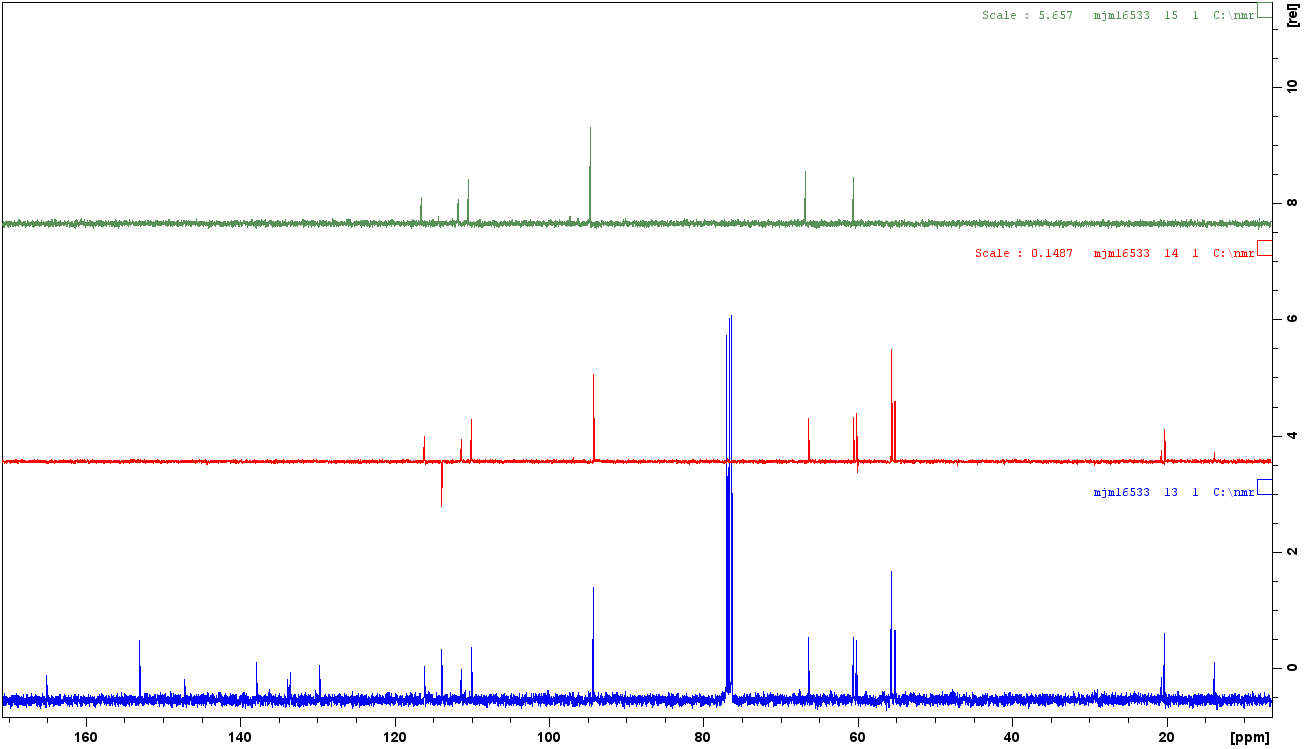

Supplement: Supplementary file 1 [file pharmaceuticals-16-01000-s001.zip › 9s mjm16533_13cDEPTs.png]

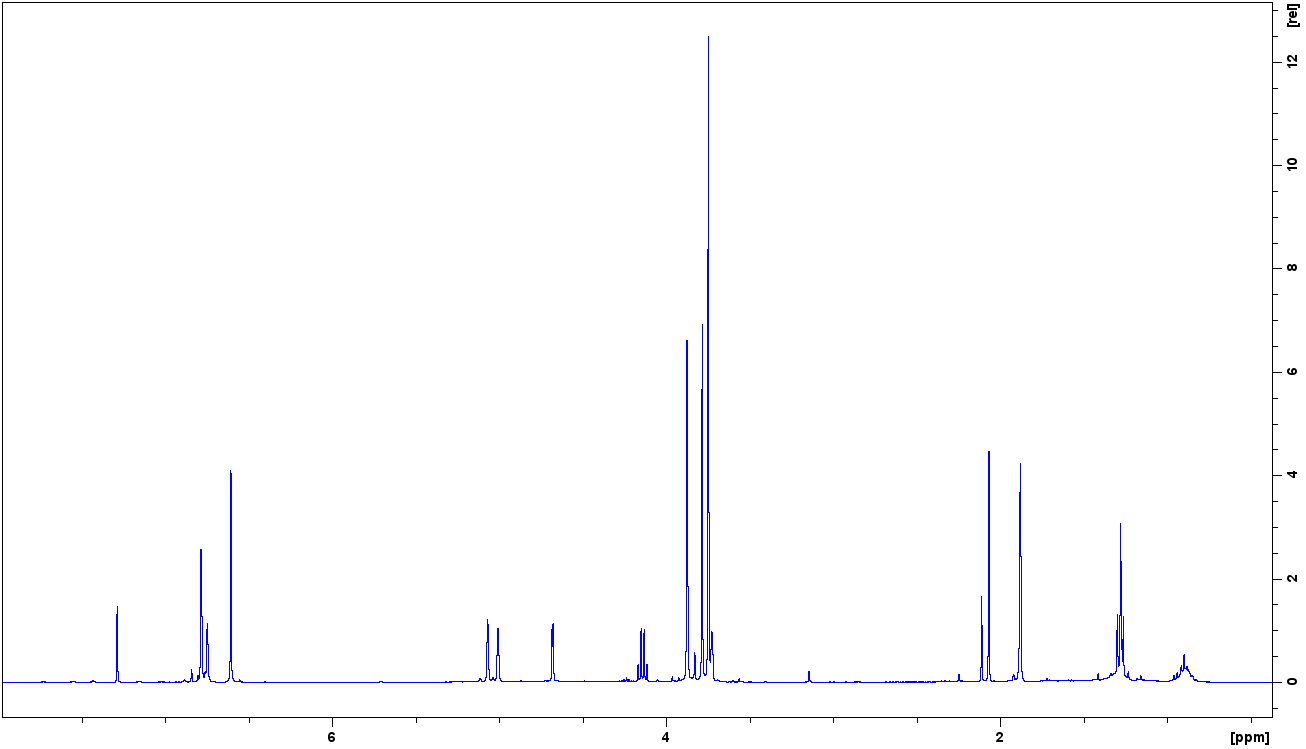

Supplement: Supplementary file 1 [file pharmaceuticals-16-01000-s001.zip › 9s mjm16533_1h.png]

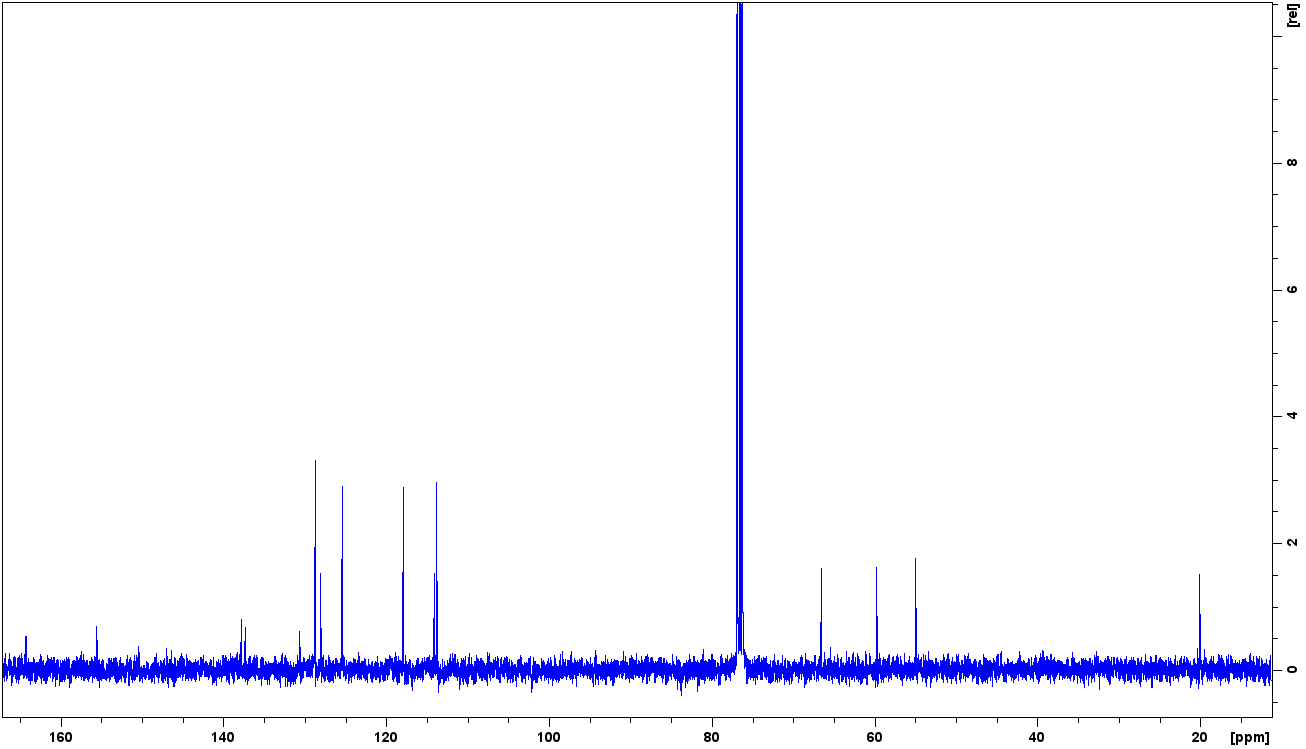

Supplement: Supplementary file 1 [file pharmaceuticals-16-01000-s001.zip › 9w mjm17400_13c.png]

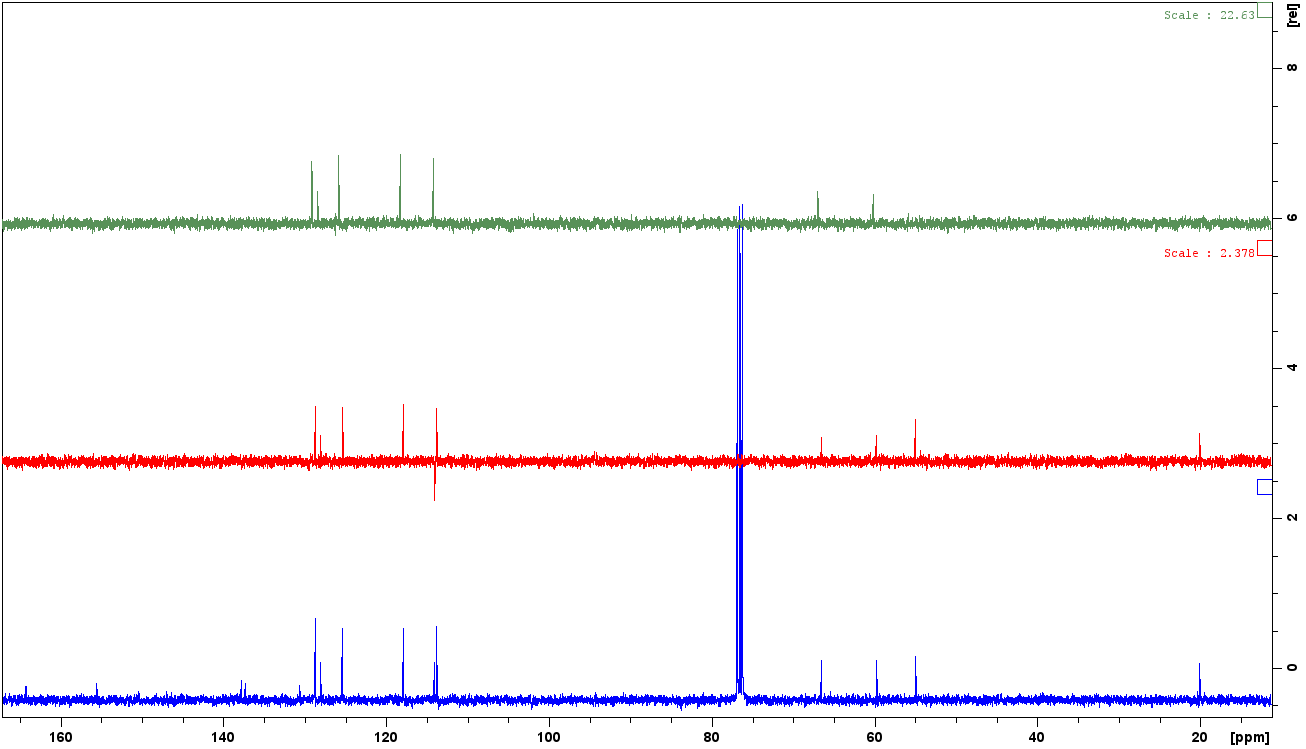

Supplement: Supplementary file 1 [file pharmaceuticals-16-01000-s001.zip › 9w mjm17400_13c_DEPTs.png]

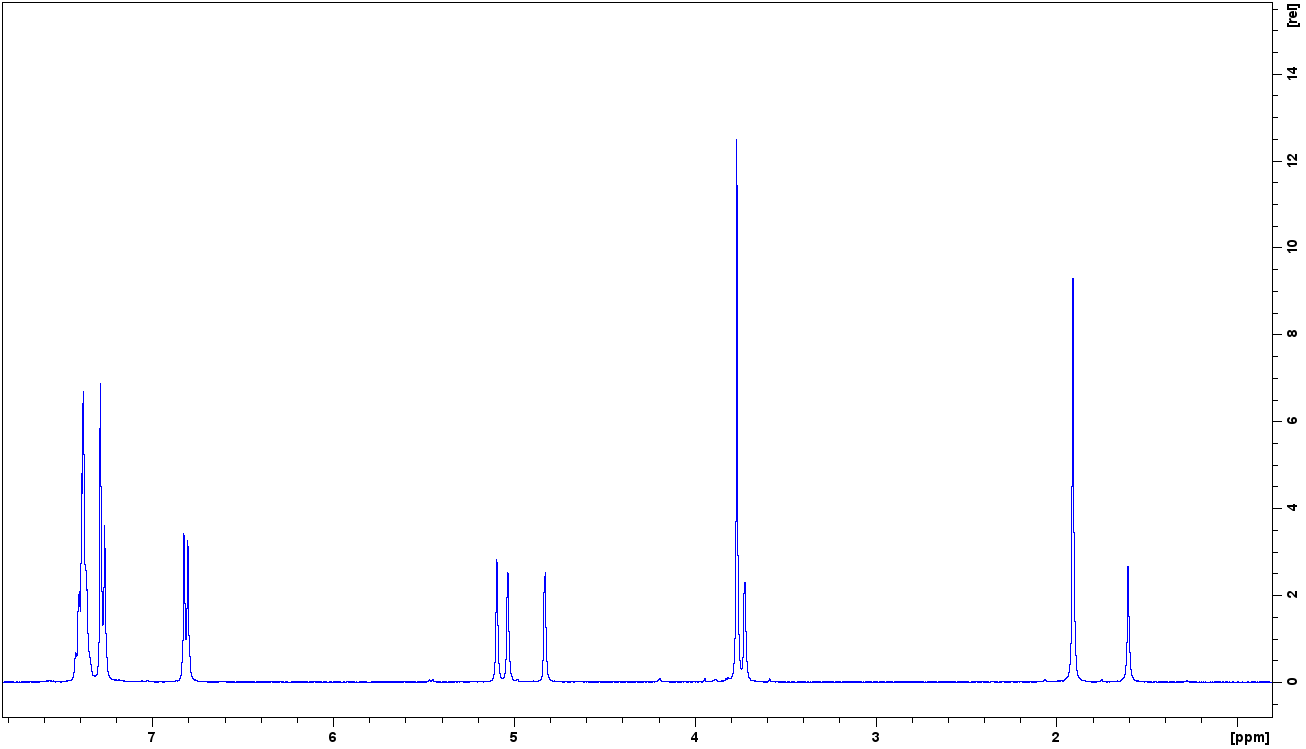

Supplement: Supplementary file 1 [file pharmaceuticals-16-01000-s001.zip › 9w mjm17400_1h.png]

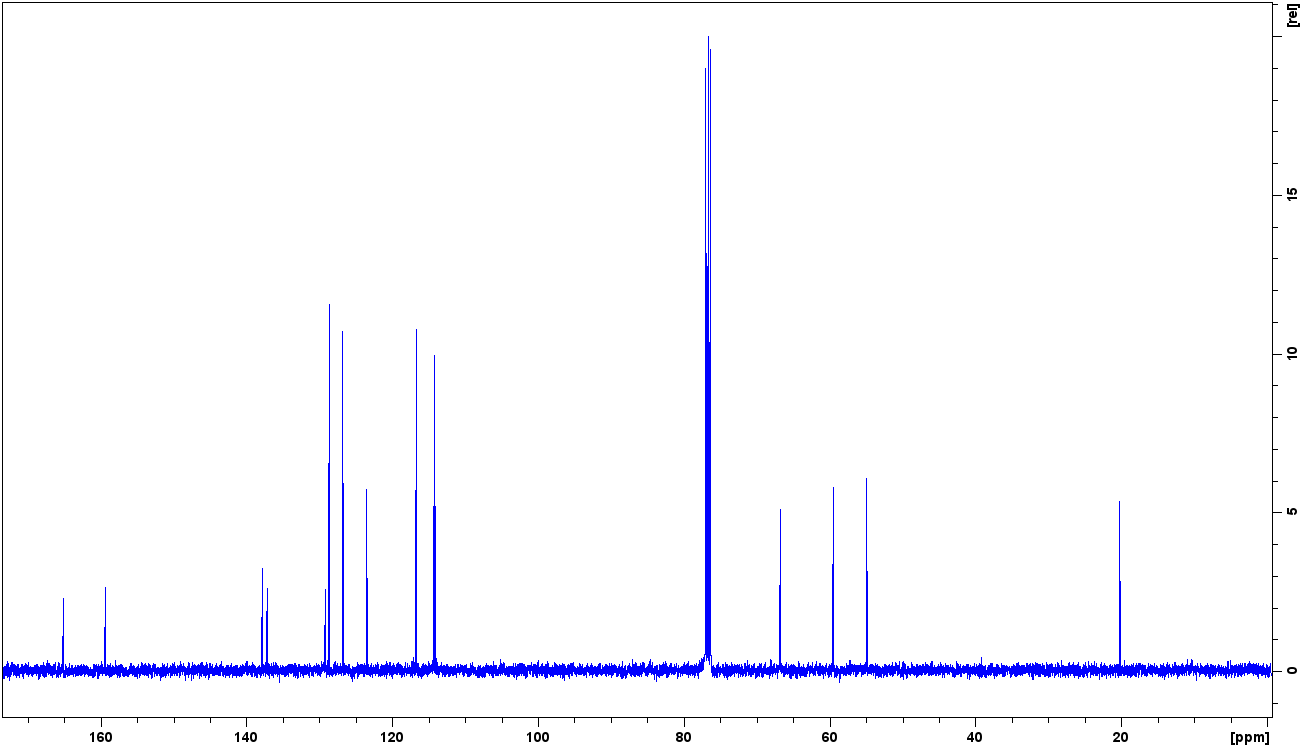

Supplement: Supplementary file 1 [file pharmaceuticals-16-01000-s001.zip › 9x mjm15469_13c.png]

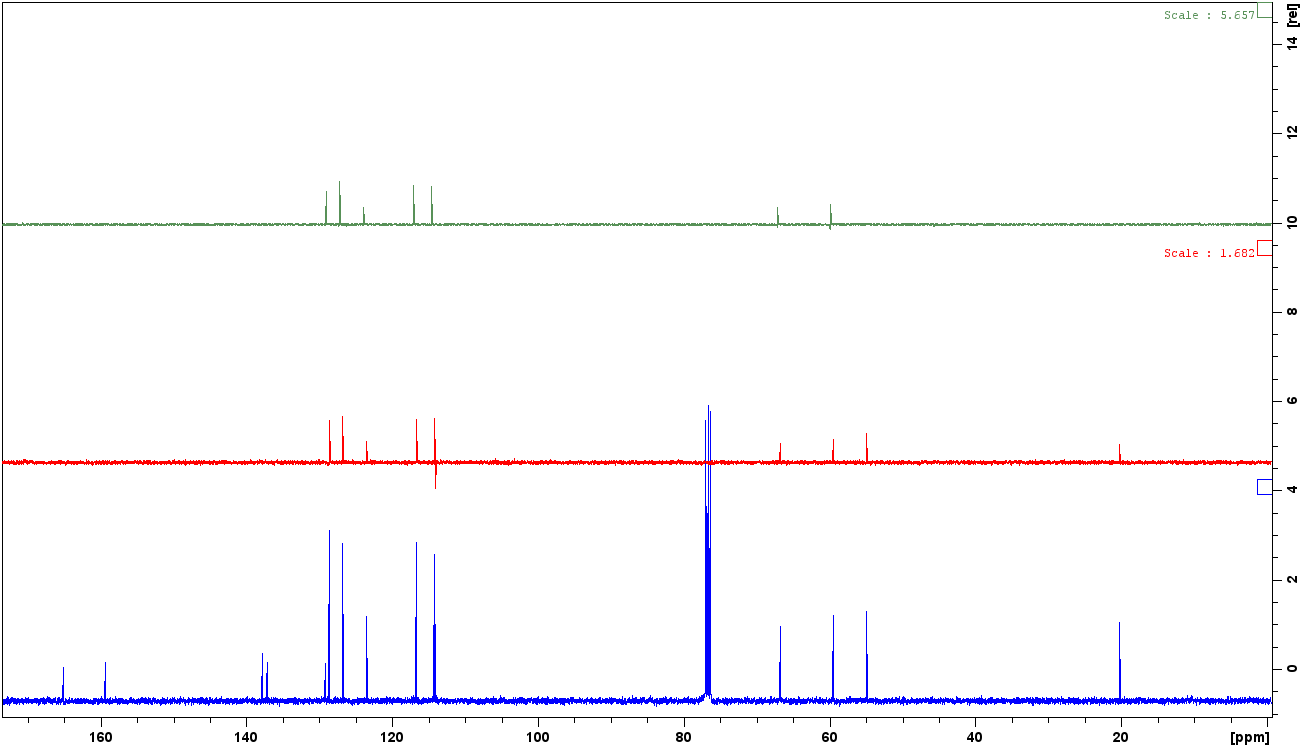

Supplement: Supplementary file 1 [file pharmaceuticals-16-01000-s001.zip › 9x mjm15469_13c_DEPTS.png]

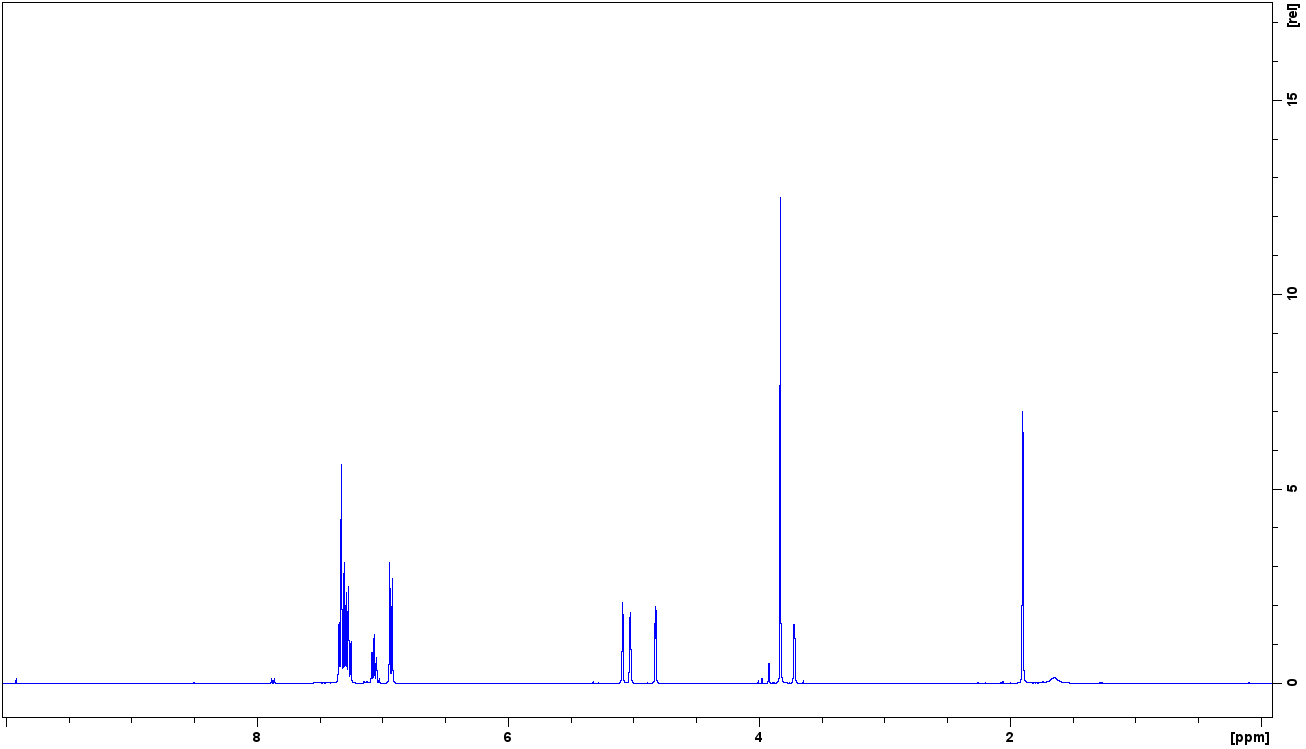

Supplement: Supplementary file 1 [file pharmaceuticals-16-01000-s001.zip › 9x mjm15469_1h.png]

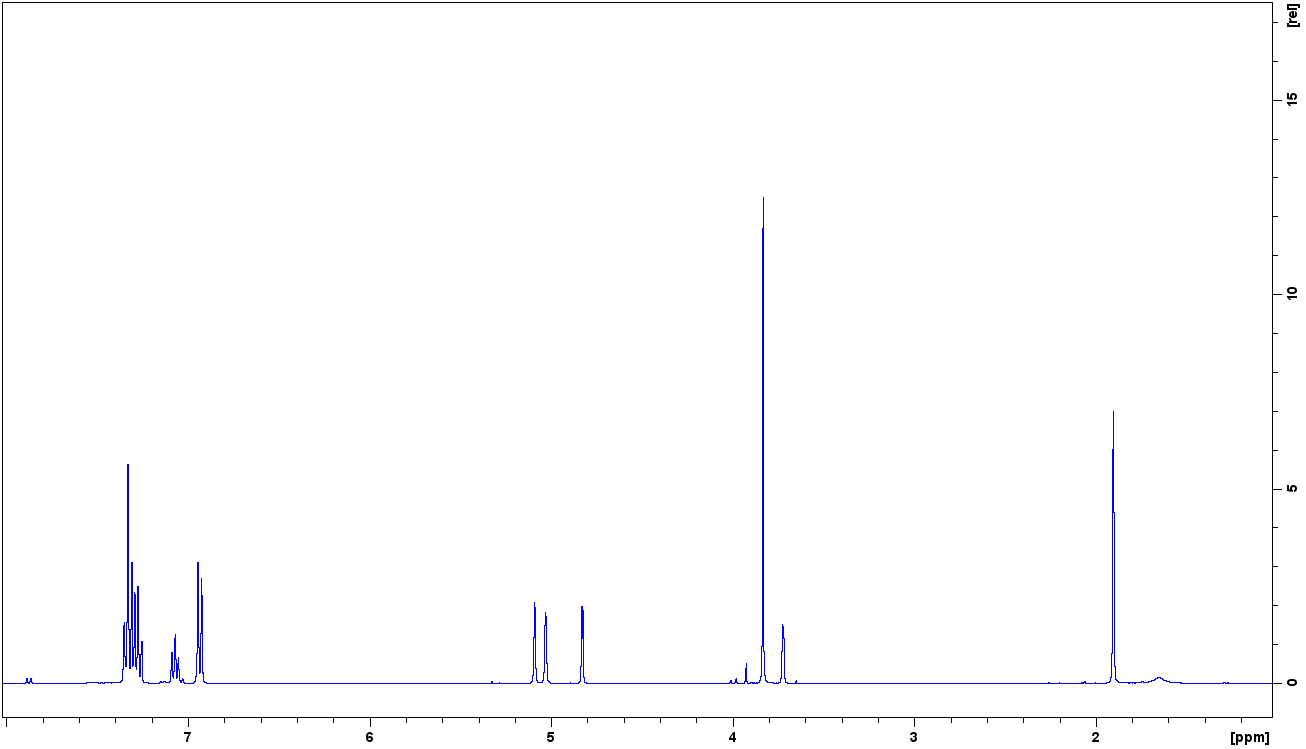

Supplement: Supplementary file 1 [file pharmaceuticals-16-01000-s001.zip › 9x mjm15469_1h_2.png]
