# Supplementary figures and images for: Antiproliferative and Tubulin-Destabilising Effects of 3-(Prop-1-en-2-yl)azetidin-2-Ones and Related Compounds in MCF-7 and MDA-MB-231 Breast Cancer Cells (part 1 of 2)
Source: Pharmaceuticals (Basel). 2023 Jul 13;16(7):1000. doi: 10.3390/ph16071000 (PMC10385824; doi:10.3390/ph16071000)

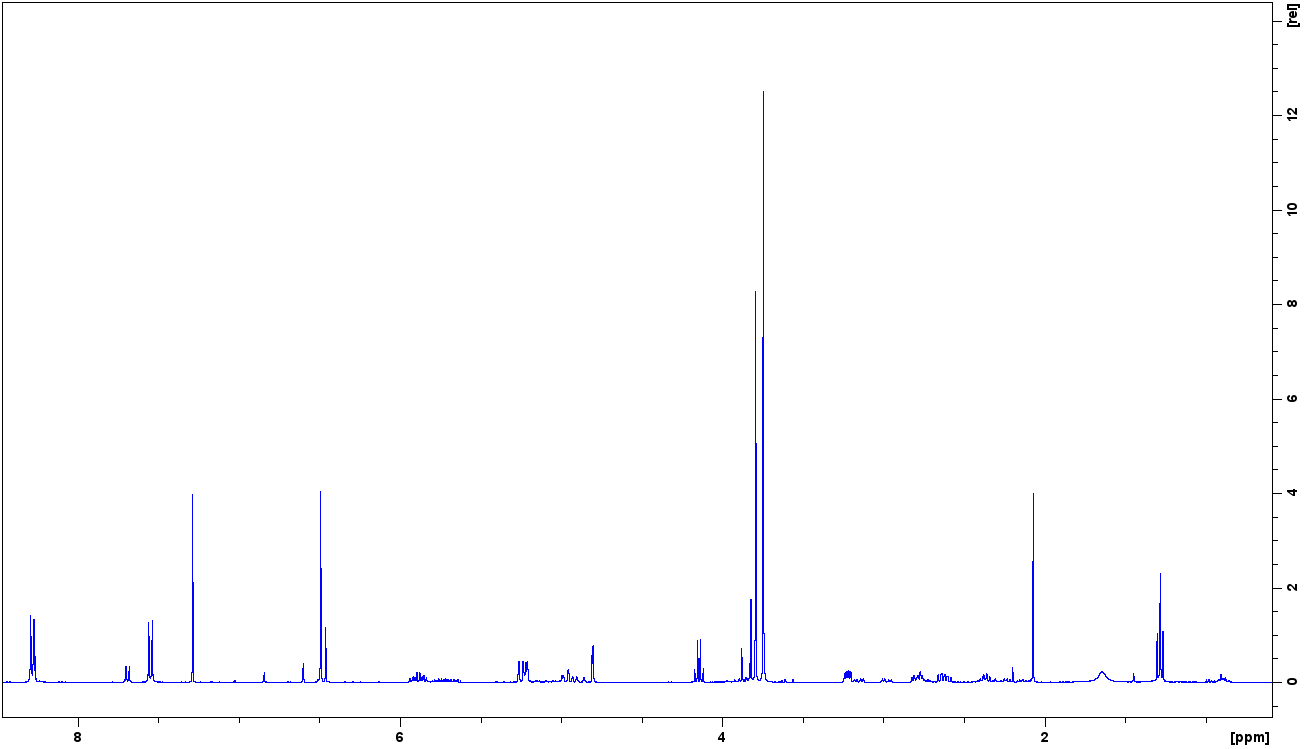

Supplement: Supplementary file 1 [file pharmaceuticals-16-01000-s001.zip › 10a mjm15903_1h.png]

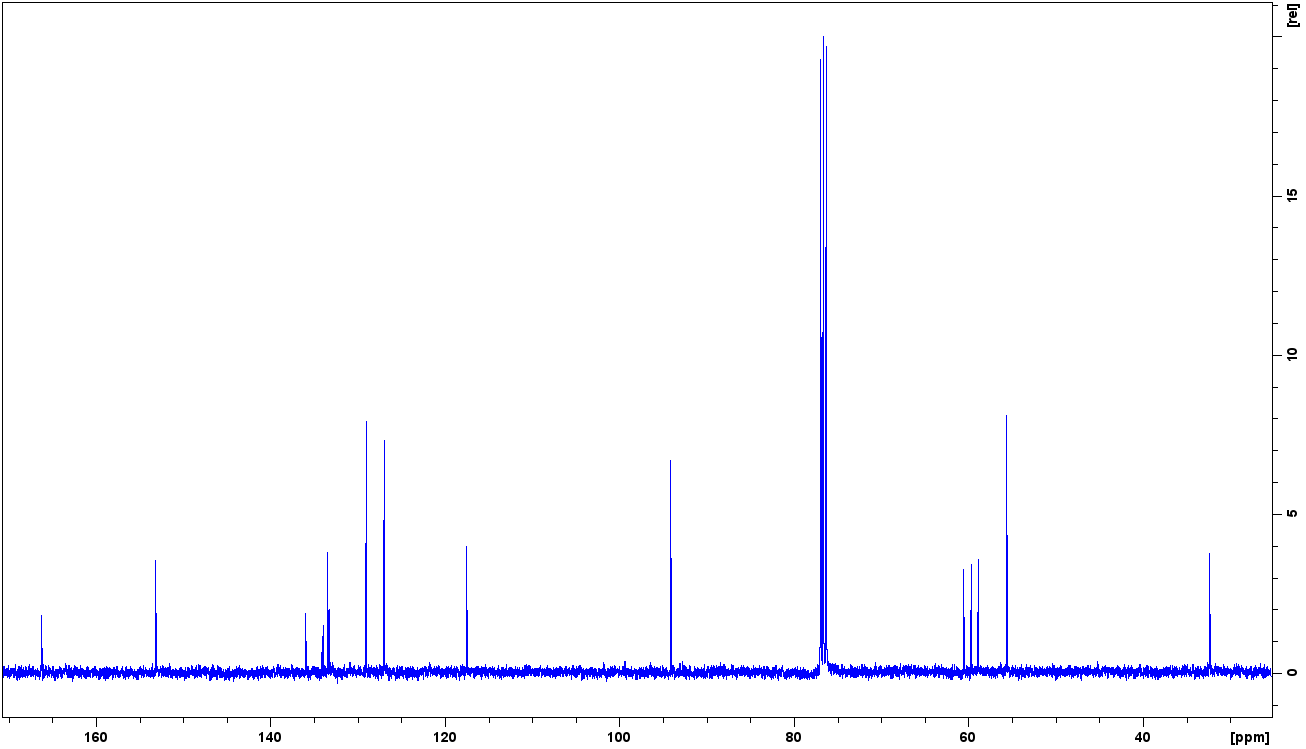

Supplement: Supplementary file 1 [file pharmaceuticals-16-01000-s001.zip › 10b mjm16576_13c.png]

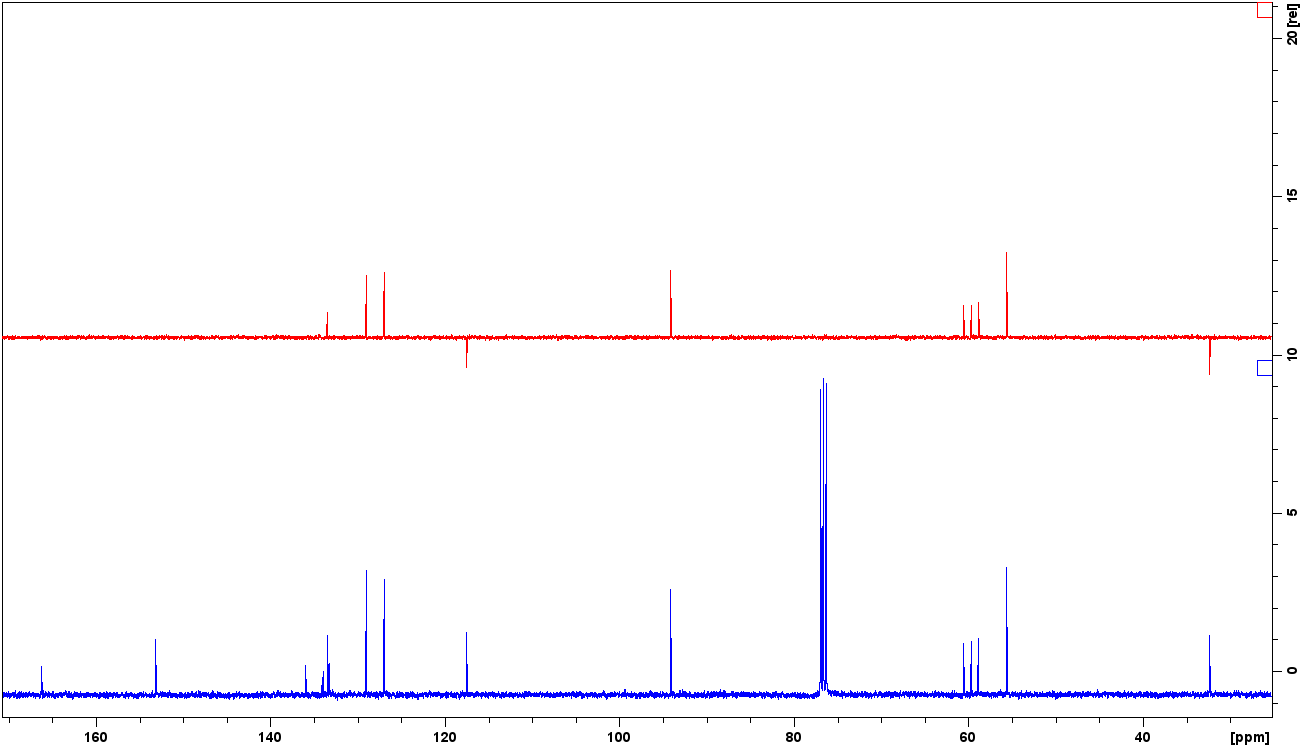

Supplement: Supplementary file 1 [file pharmaceuticals-16-01000-s001.zip › 10b mjm16576_13c_DEPT.png]

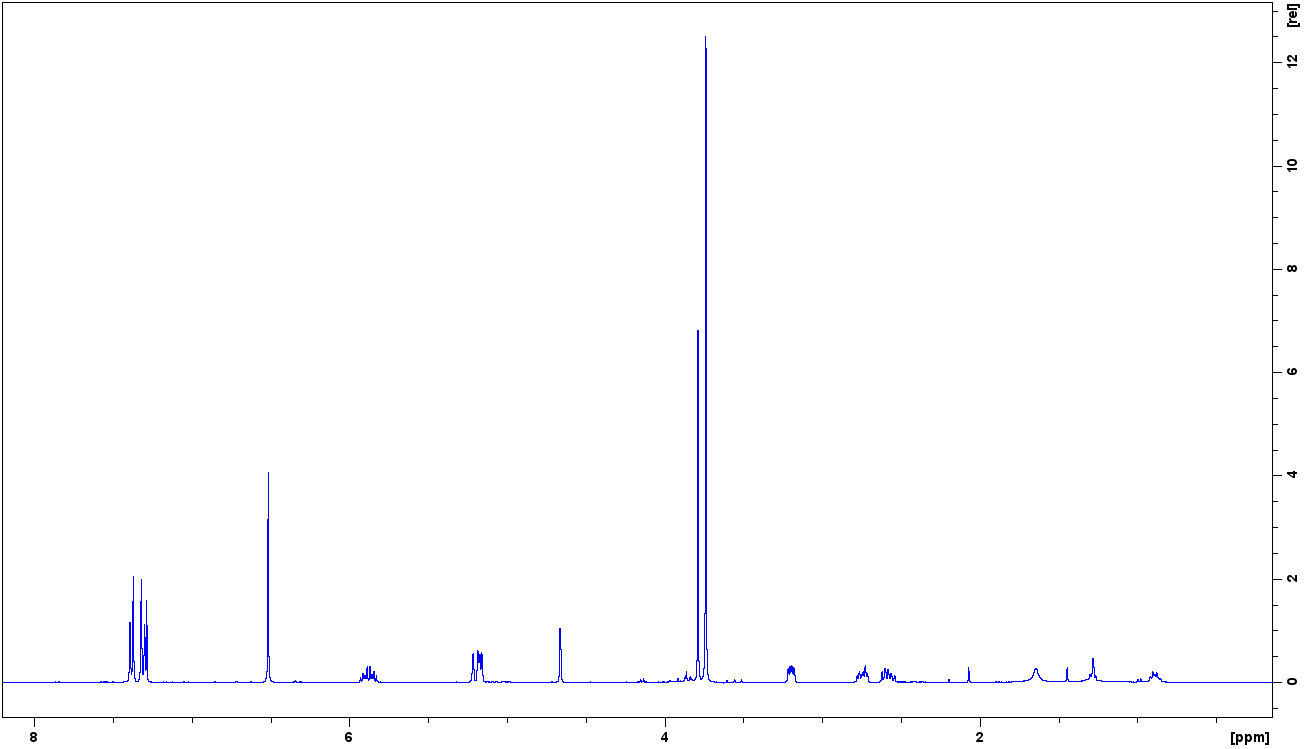

Supplement: Supplementary file 1 [file pharmaceuticals-16-01000-s001.zip › 10b mjm16576_1h.png]

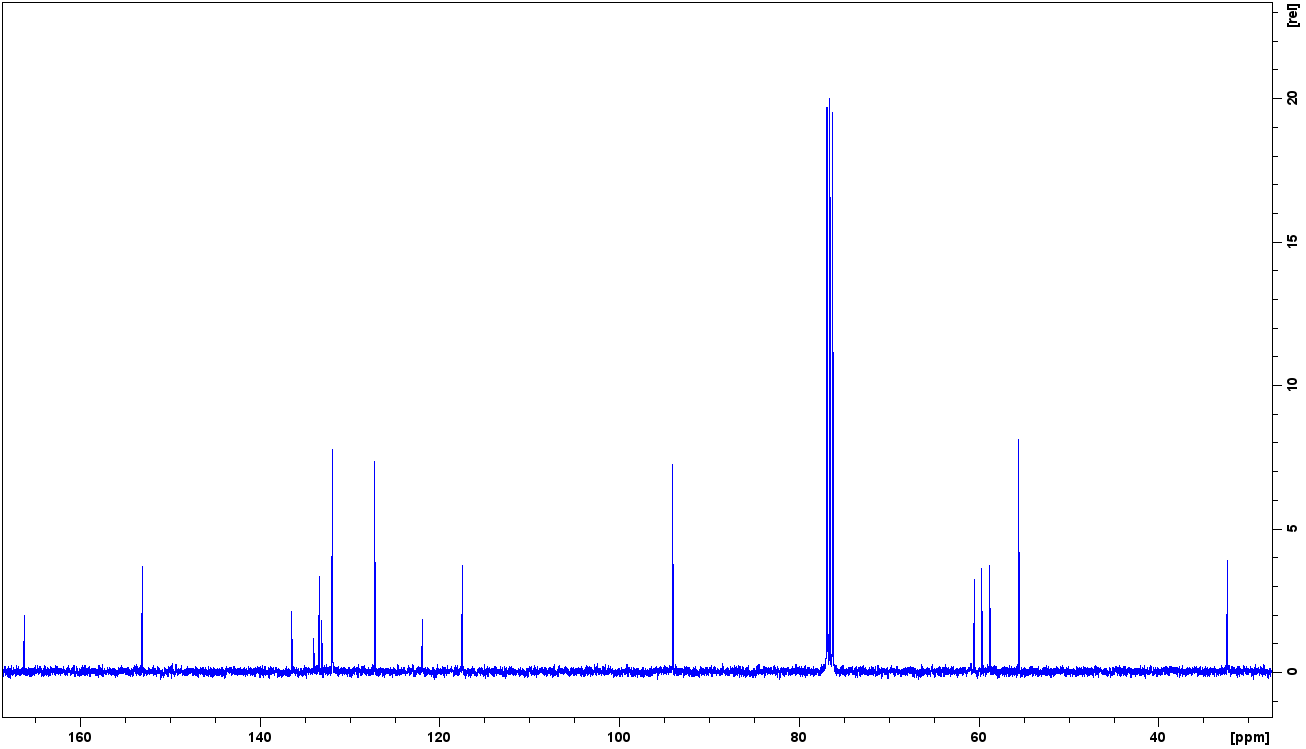

Supplement: Supplementary file 1 [file pharmaceuticals-16-01000-s001.zip › 10c mjm16570_13c.png]

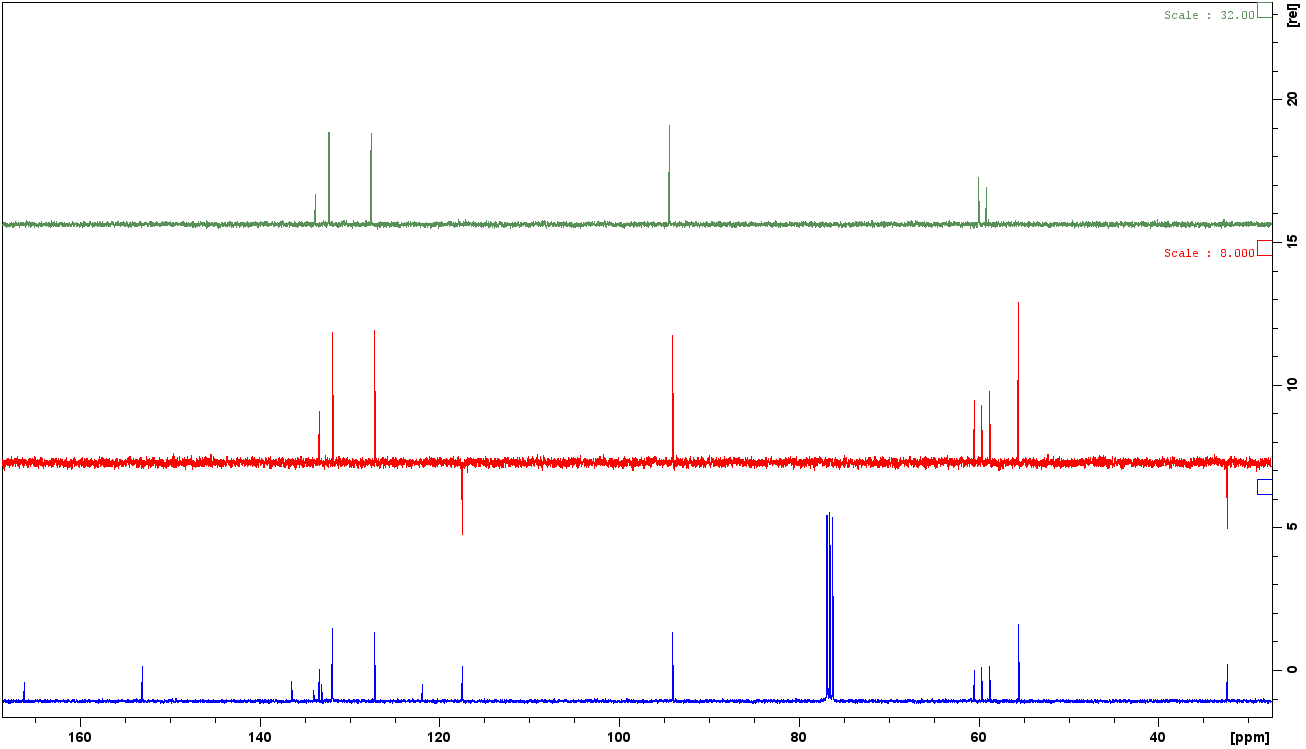

Supplement: Supplementary file 1 [file pharmaceuticals-16-01000-s001.zip › 10c mjm16570_13c_DEPTs.png]

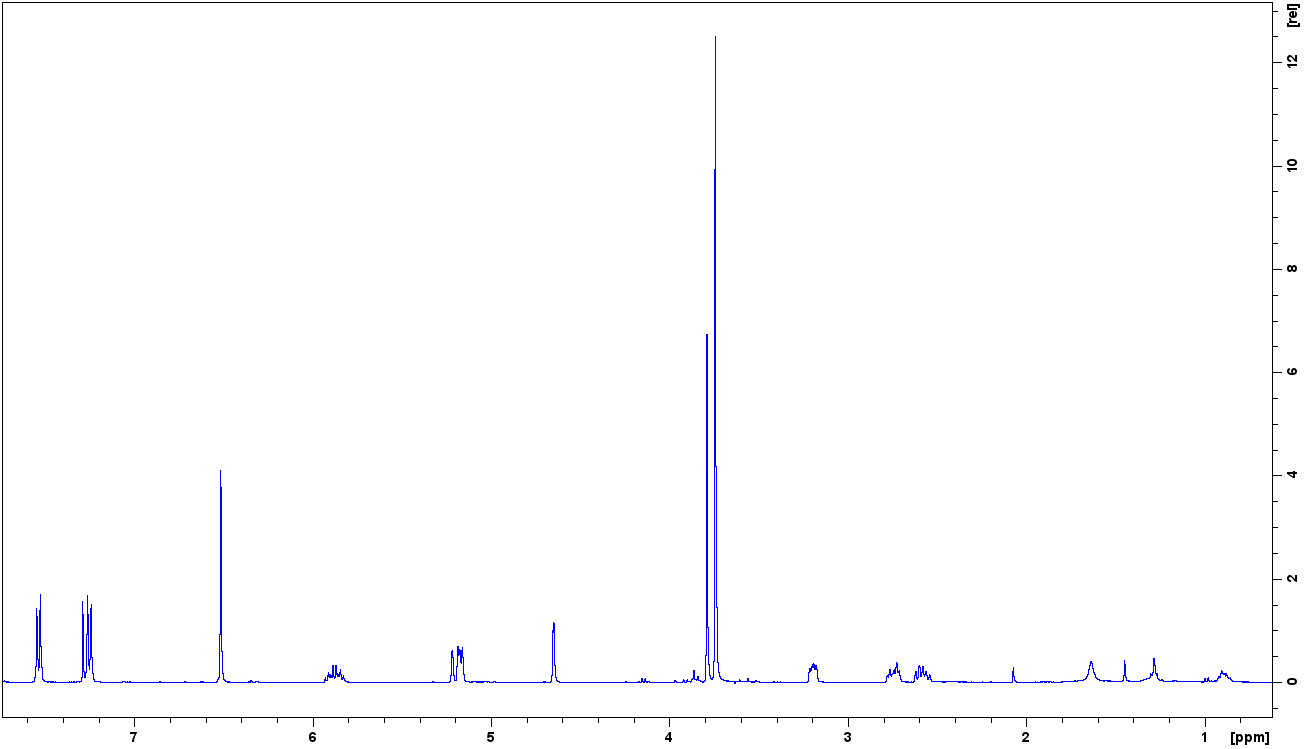

Supplement: Supplementary file 1 [file pharmaceuticals-16-01000-s001.zip › 10c mjm16570_1h.png]

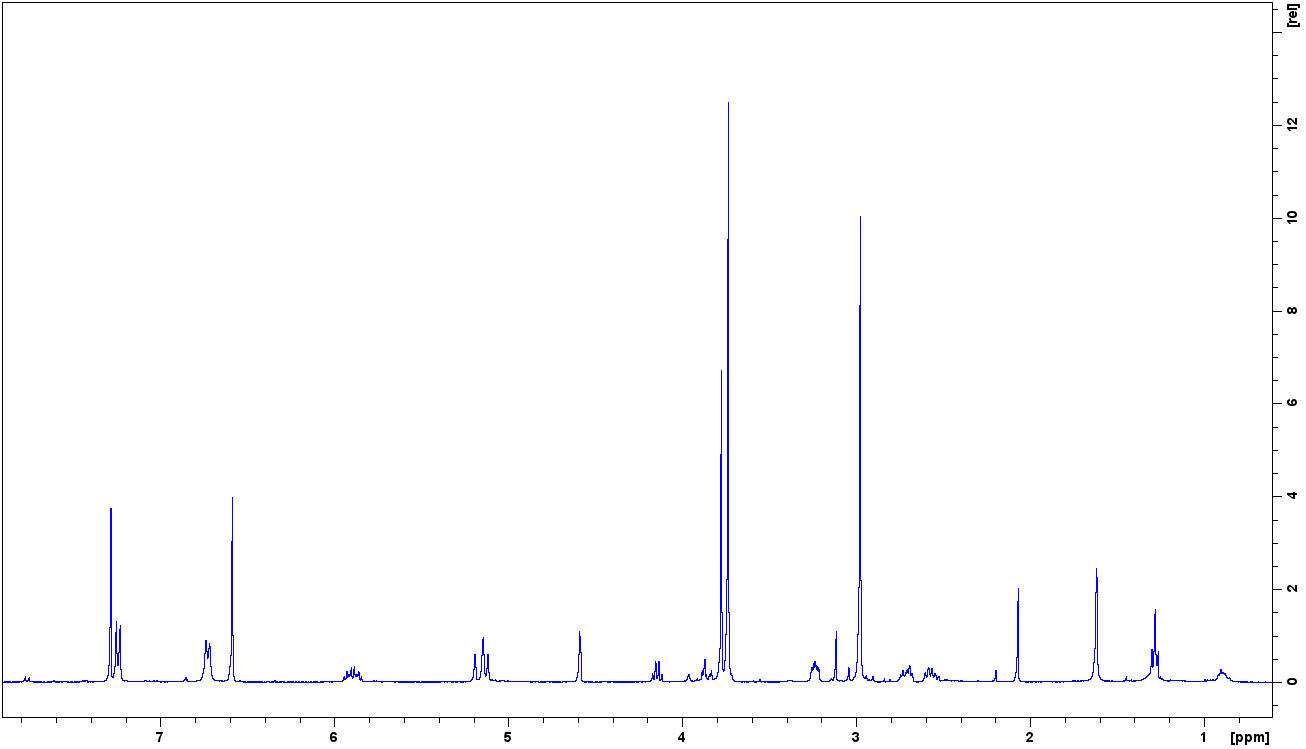

Supplement: Supplementary file 1 [file pharmaceuticals-16-01000-s001.zip › 10e mjm15970_1h.png]

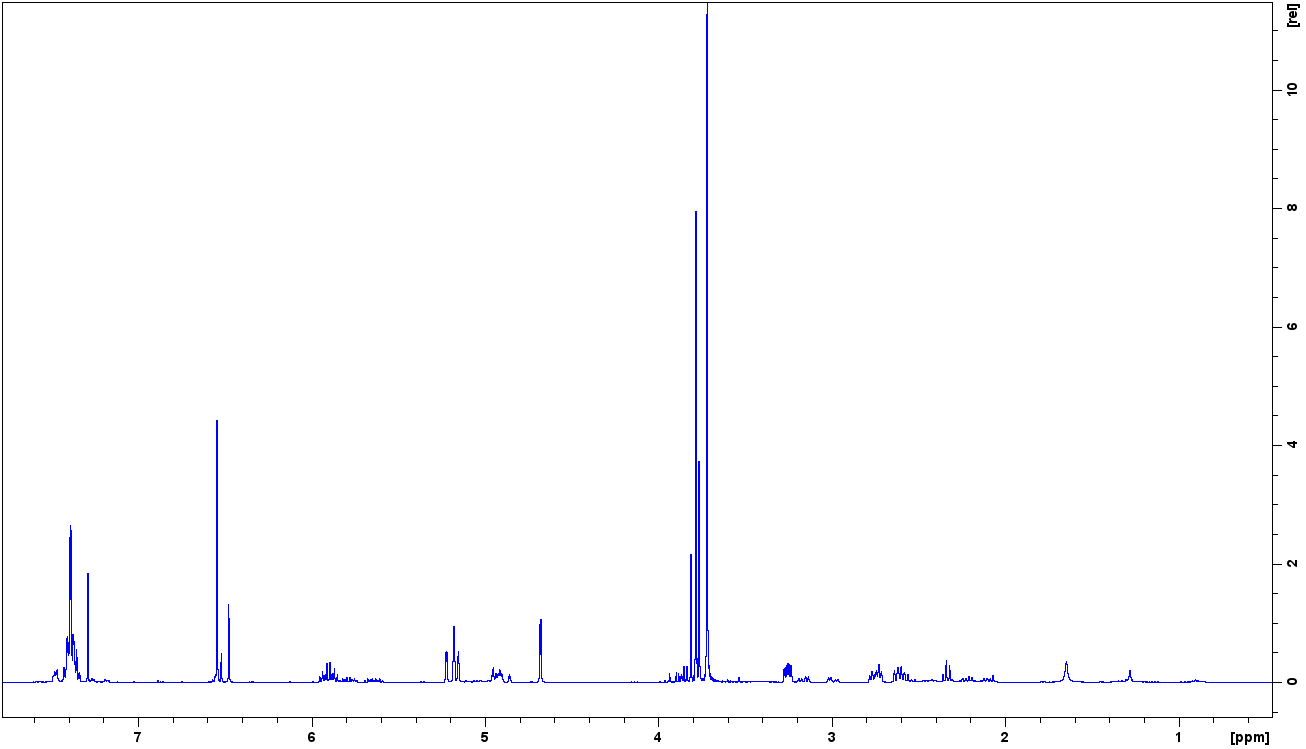

Supplement: Supplementary file 1 [file pharmaceuticals-16-01000-s001.zip › 10f mjm15807_1h.png]

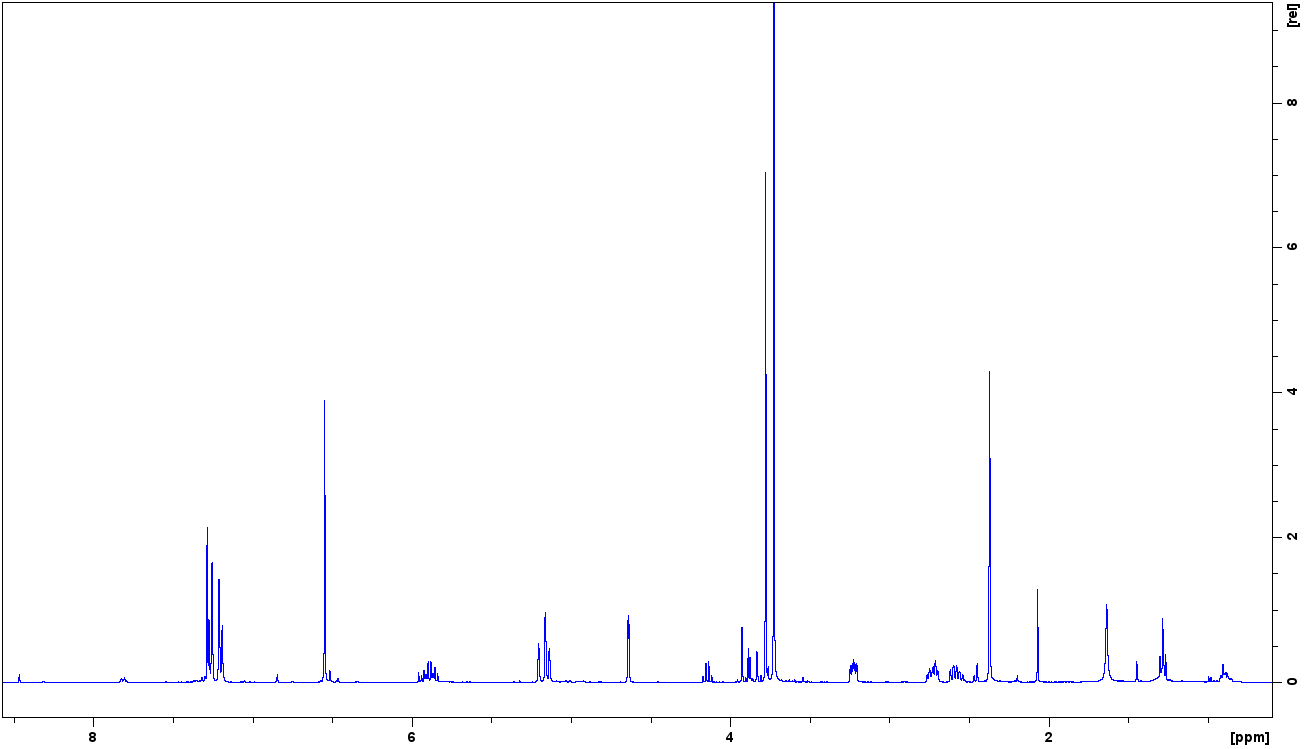

Supplement: Supplementary file 1 [file pharmaceuticals-16-01000-s001.zip › 10g mjm15896_1h.png]

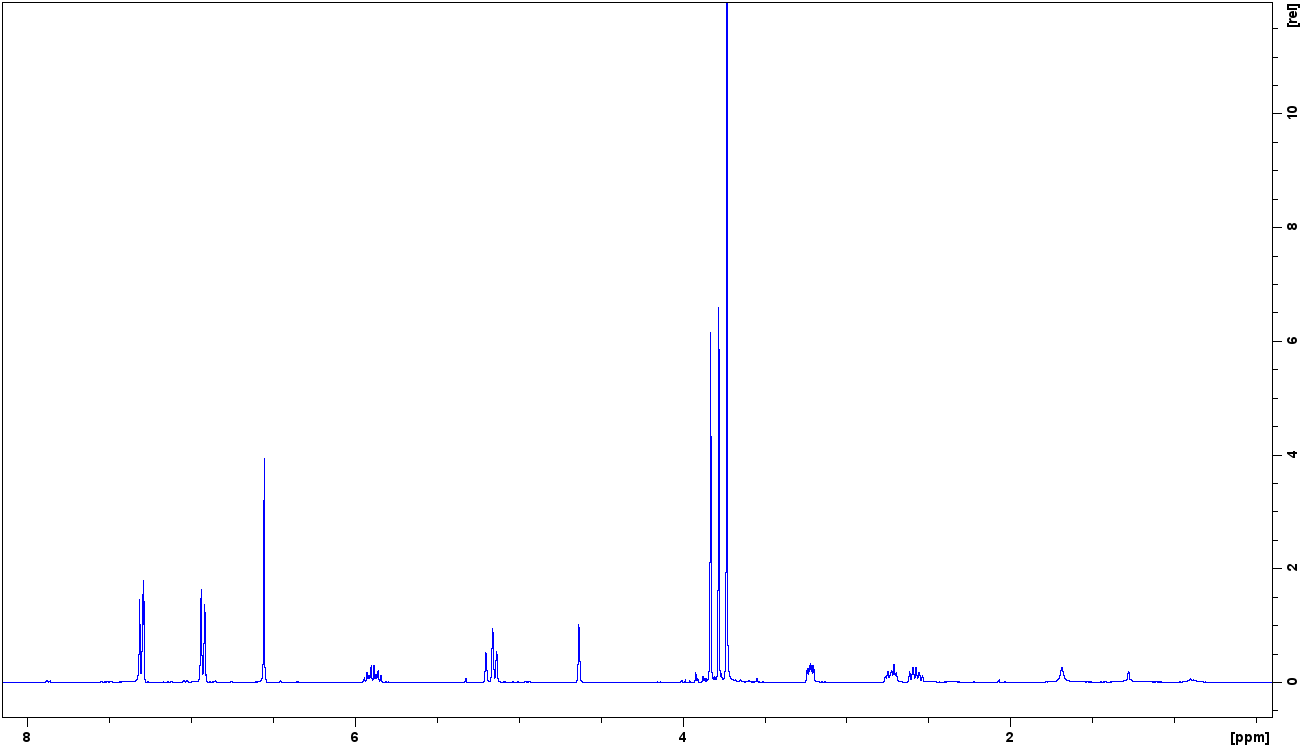

Supplement: Supplementary file 1 [file pharmaceuticals-16-01000-s001.zip › 10h mjm15736_1h.png]

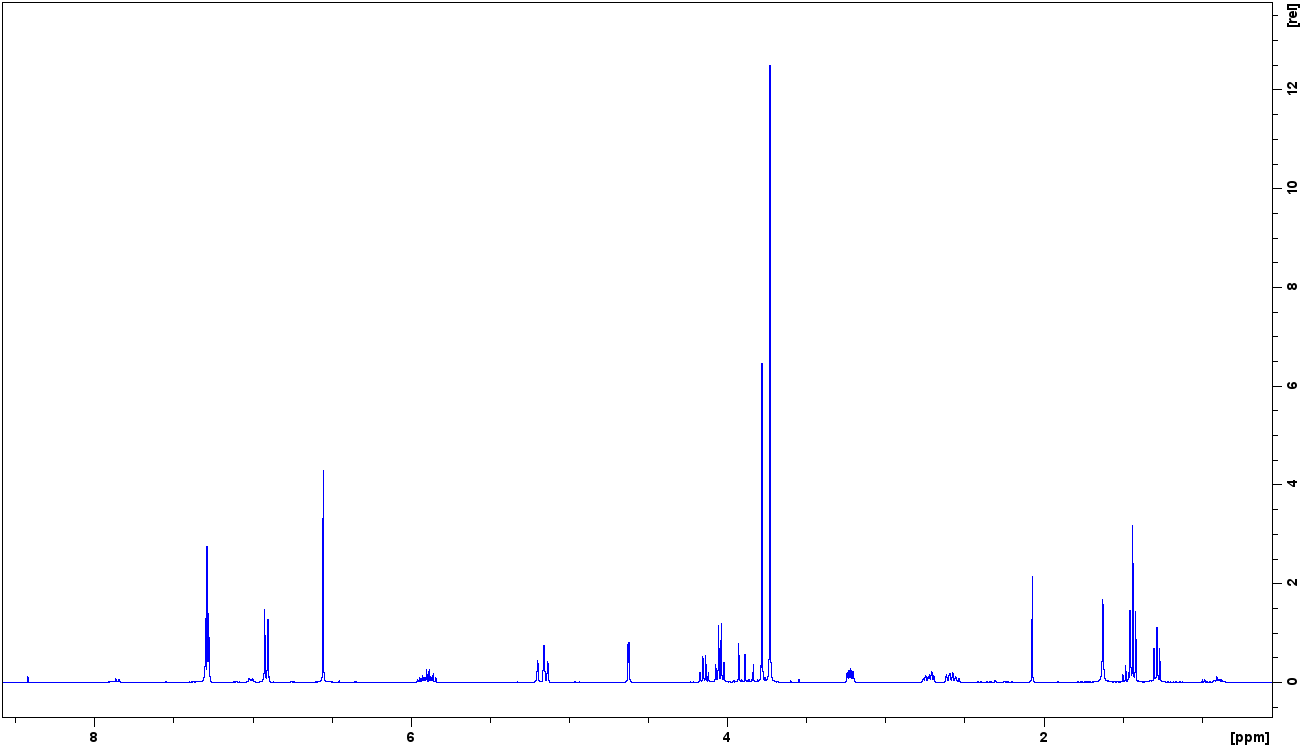

Supplement: Supplementary file 1 [file pharmaceuticals-16-01000-s001.zip › 10i mjm15863_1h.png]

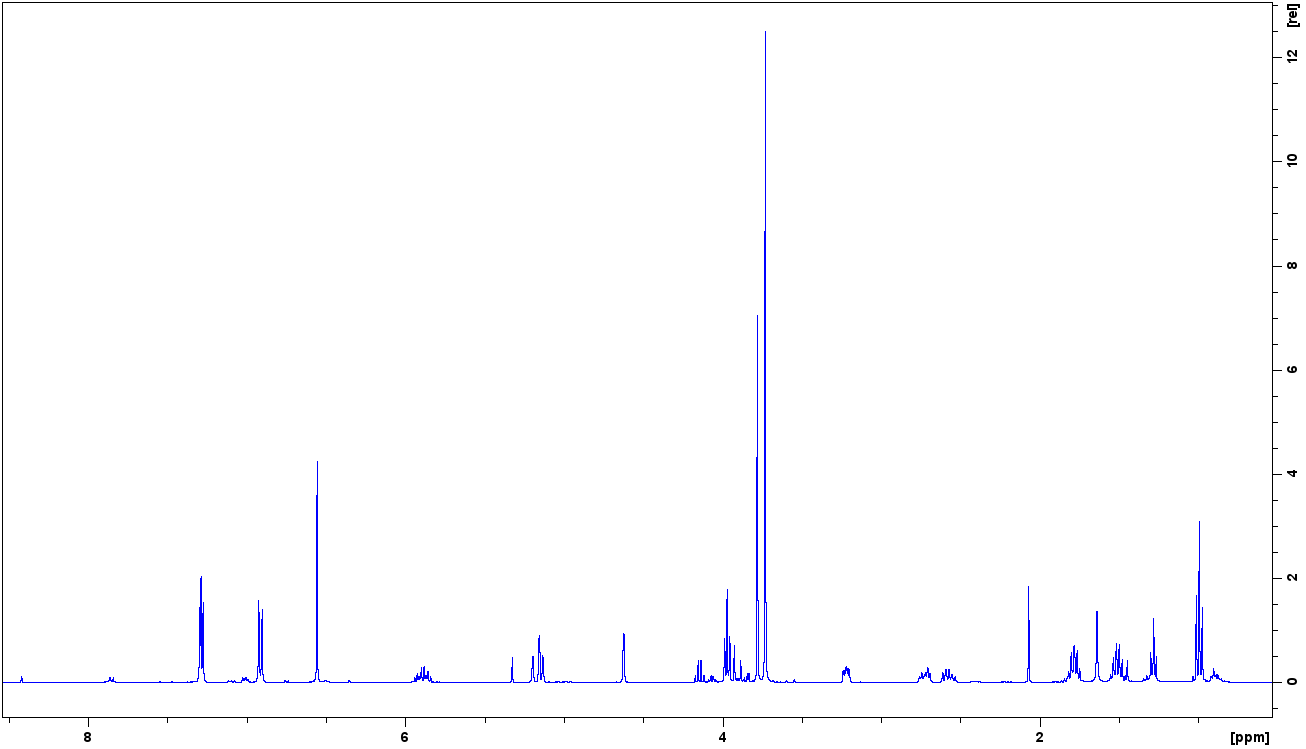

Supplement: Supplementary file 1 [file pharmaceuticals-16-01000-s001.zip › 10j mjm15873_1h.png]

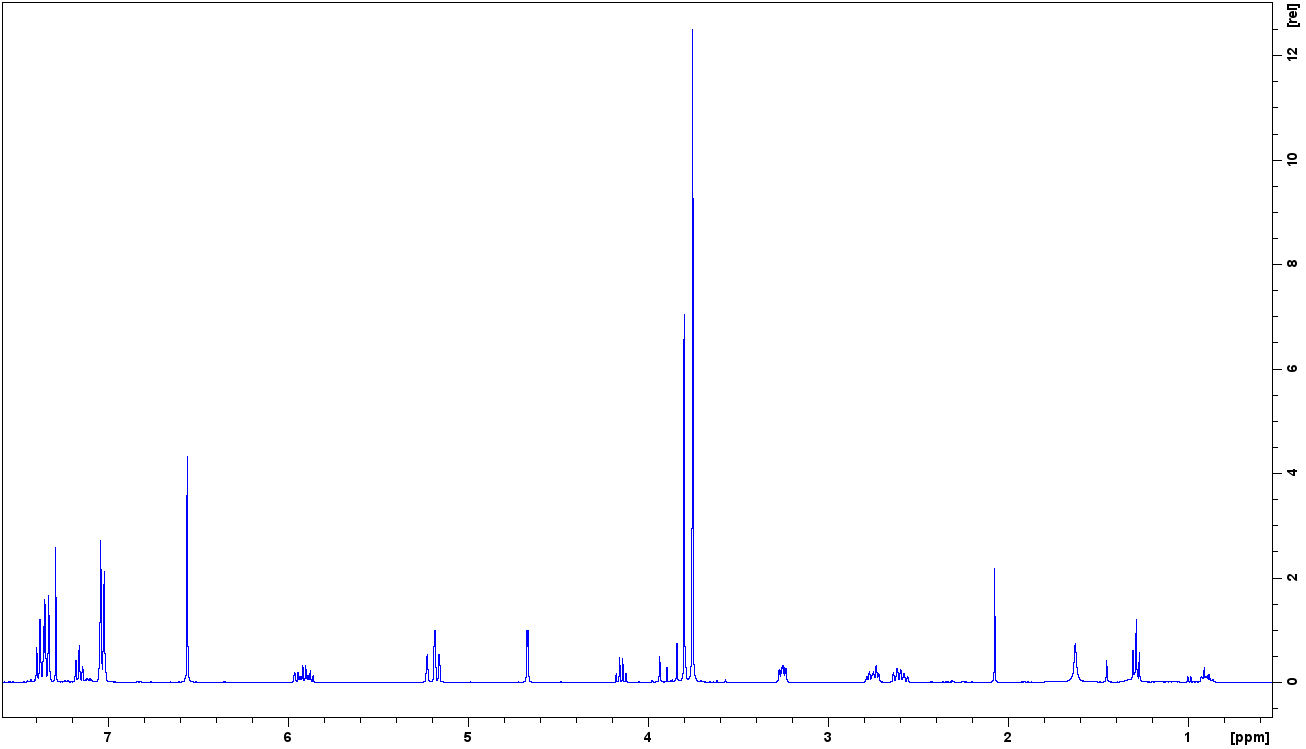

Supplement: Supplementary file 1 [file pharmaceuticals-16-01000-s001.zip › 10k mjm15844_1h.png]

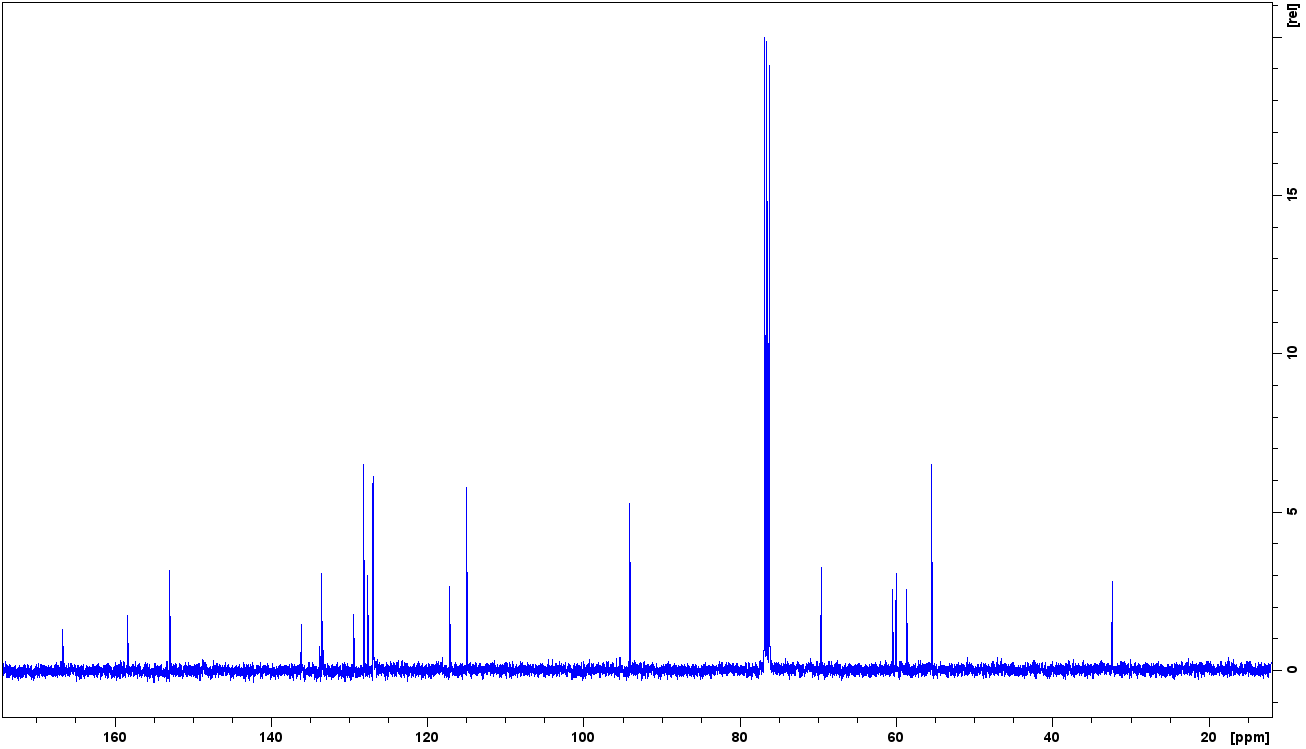

Supplement: Supplementary file 1 [file pharmaceuticals-16-01000-s001.zip › 10l mjm15908_13c.png]

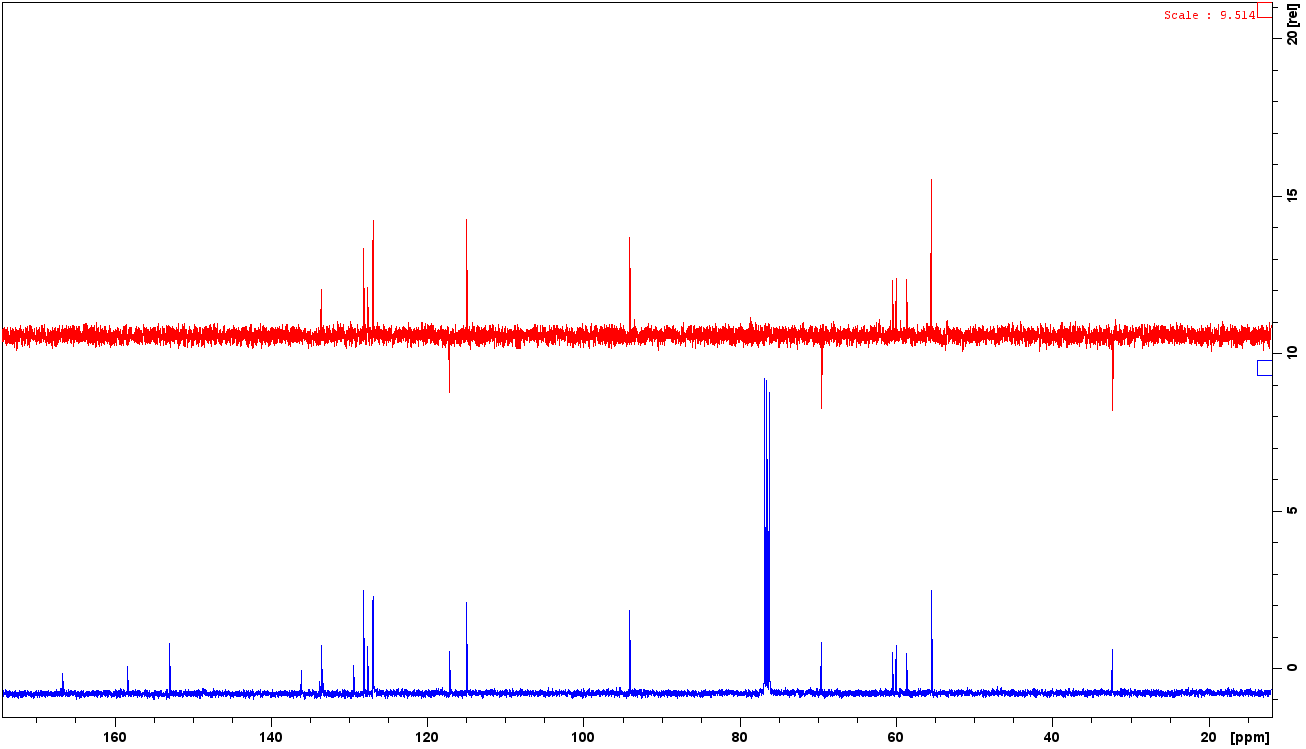

Supplement: Supplementary file 1 [file pharmaceuticals-16-01000-s001.zip › 10l mjm15908_13c_DEPT.png]

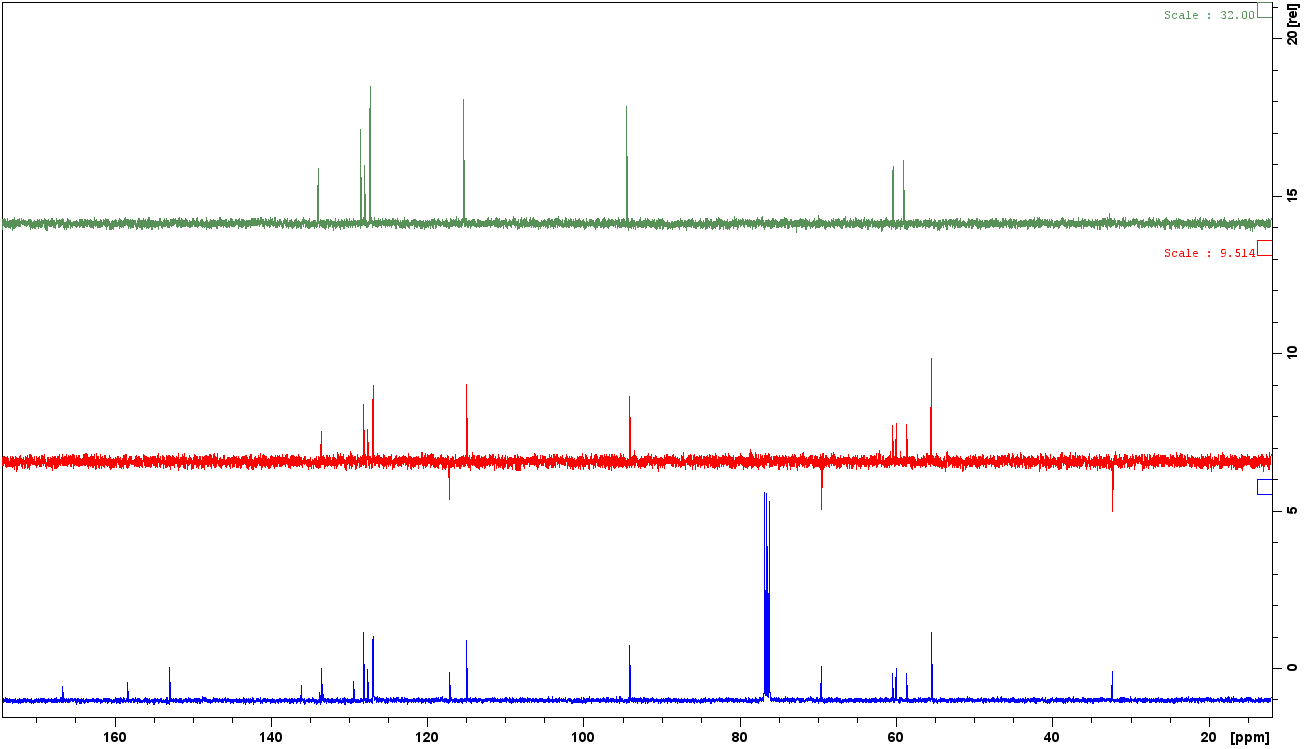

Supplement: Supplementary file 1 [file pharmaceuticals-16-01000-s001.zip › 10l mjm15908_13c_DEPTs.png]

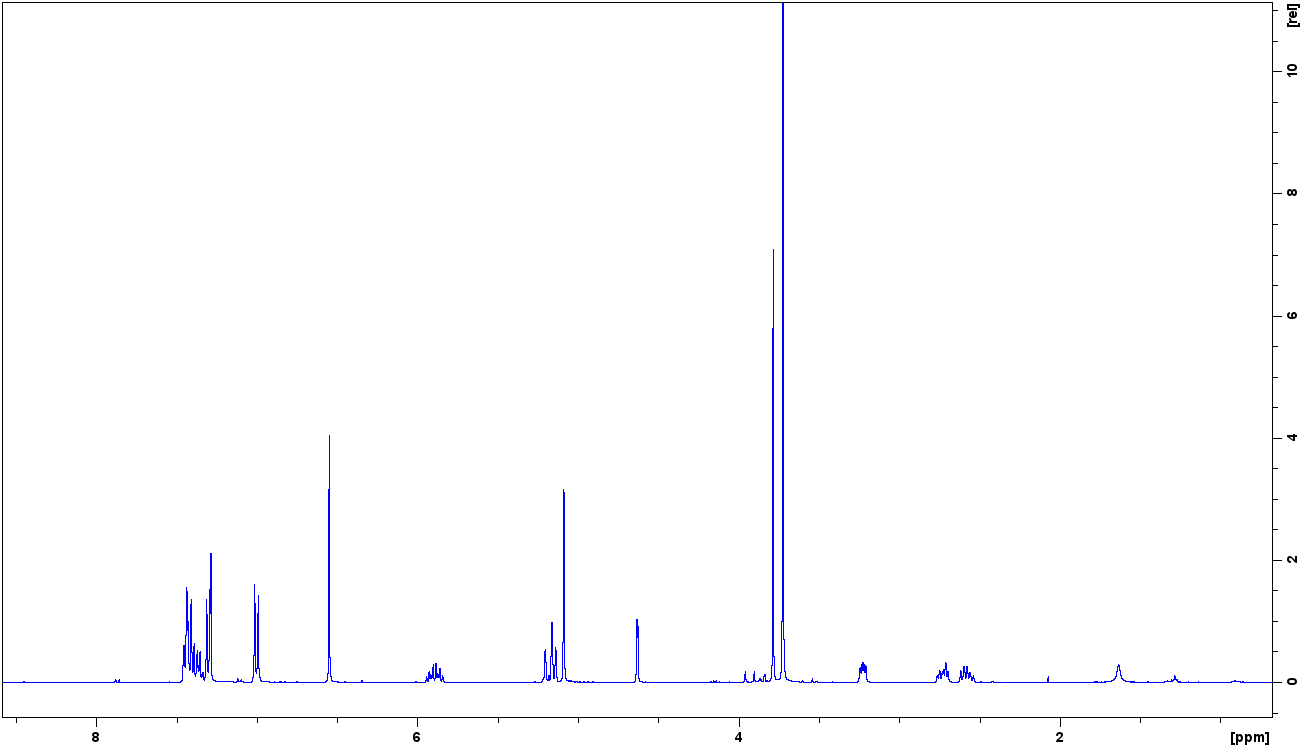

Supplement: Supplementary file 1 [file pharmaceuticals-16-01000-s001.zip › 10l mjm15908_1h.png]

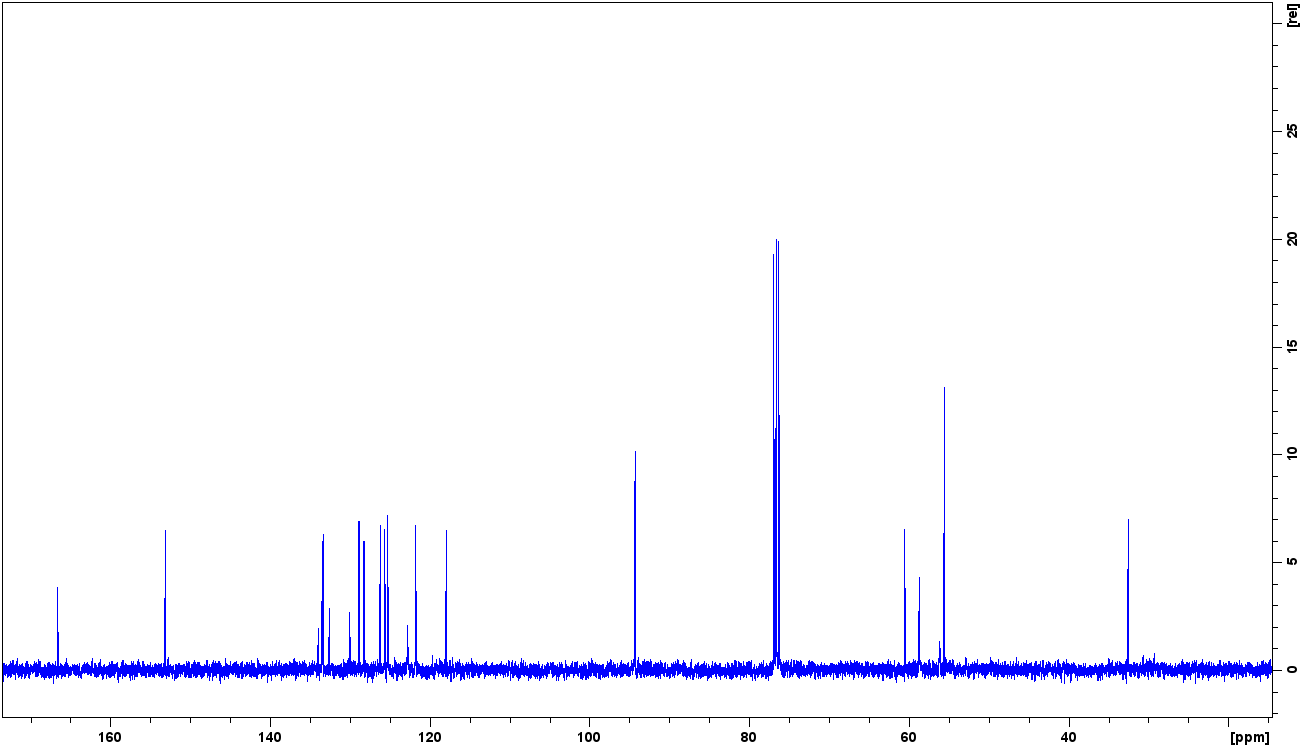

Supplement: Supplementary file 1 [file pharmaceuticals-16-01000-s001.zip › 10m mjm15923_13c.png]

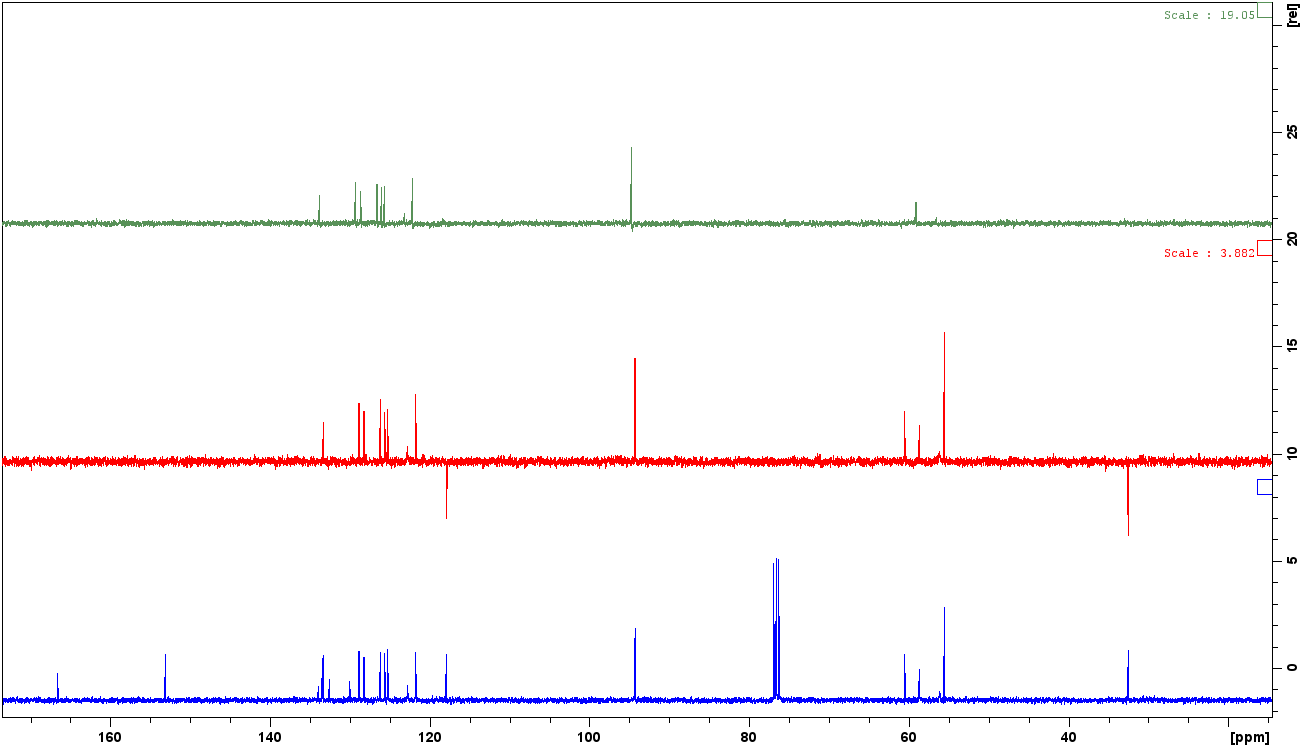

Supplement: Supplementary file 1 [file pharmaceuticals-16-01000-s001.zip › 10m mjm15923_13cDEPTs.png]

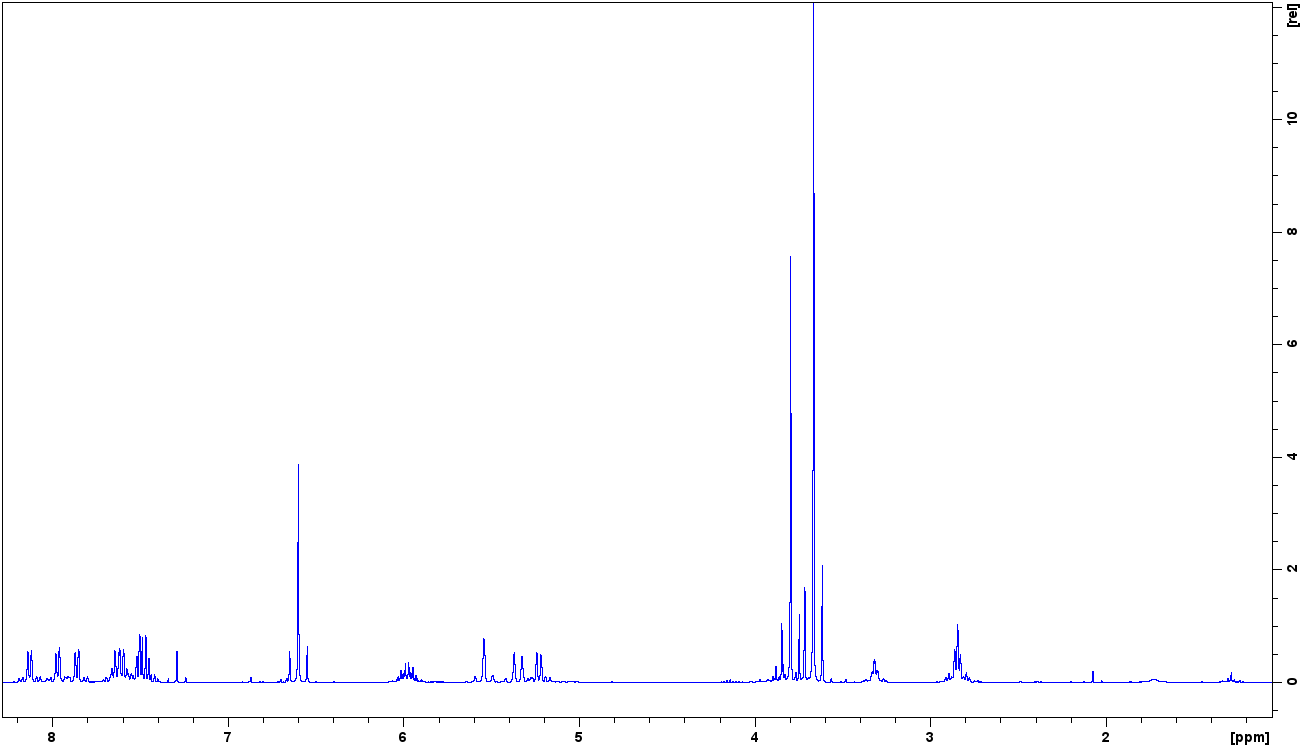

Supplement: Supplementary file 1 [file pharmaceuticals-16-01000-s001.zip › 10m mjm15923_1h.png]

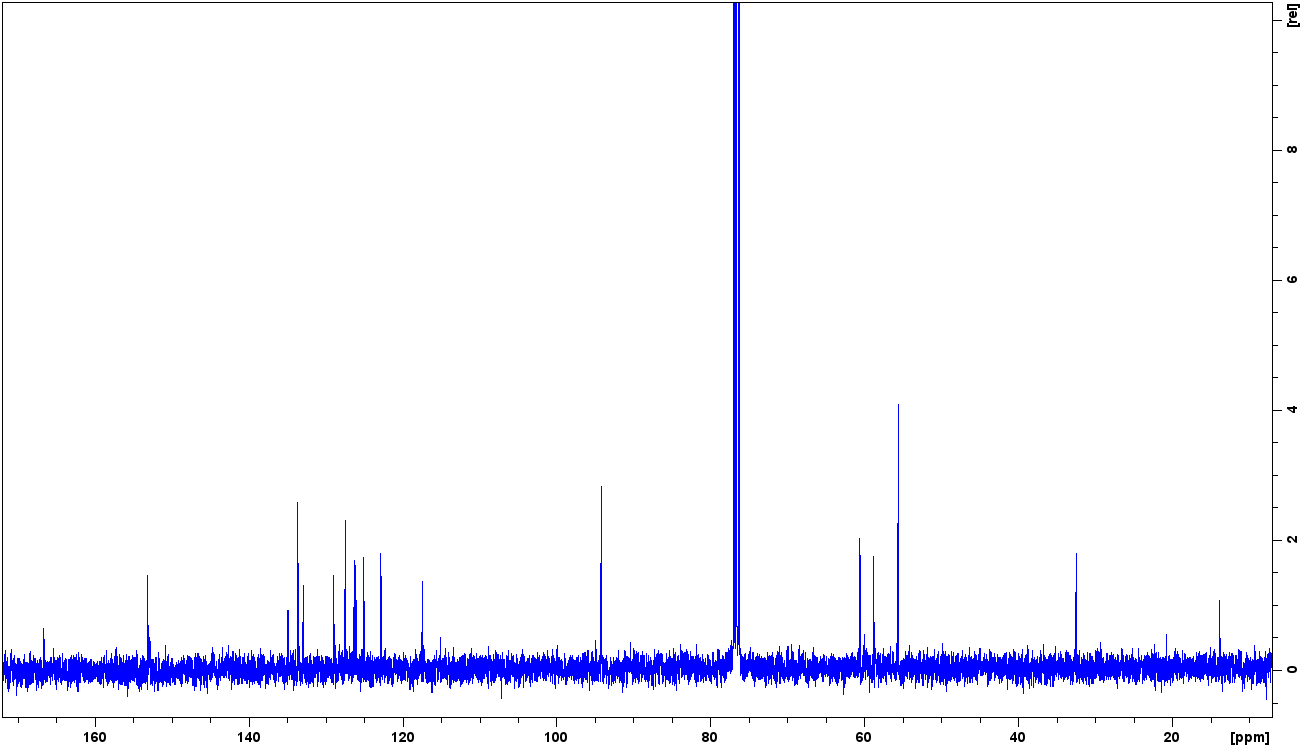

Supplement: Supplementary file 1 [file pharmaceuticals-16-01000-s001.zip › 10n mjm15940_13c.png]

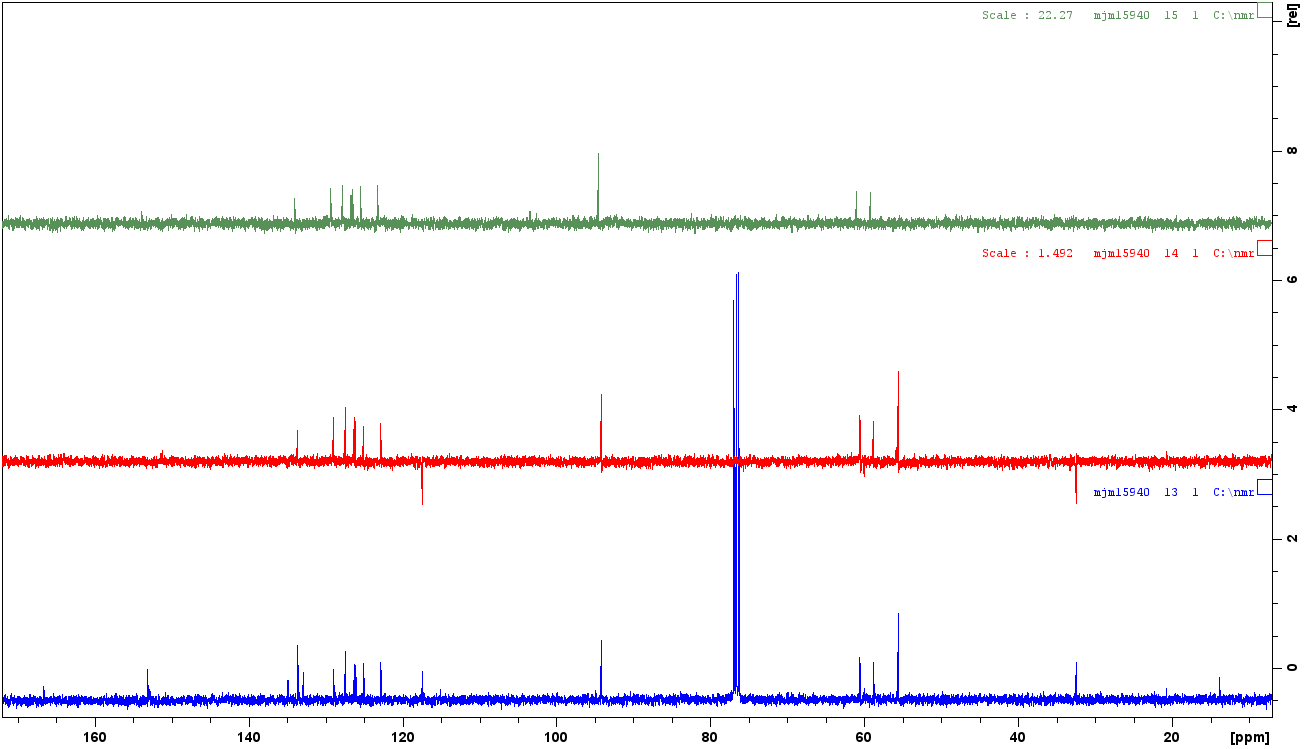

Supplement: Supplementary file 1 [file pharmaceuticals-16-01000-s001.zip › 10n mjm15940_13c_DEPTs.png]

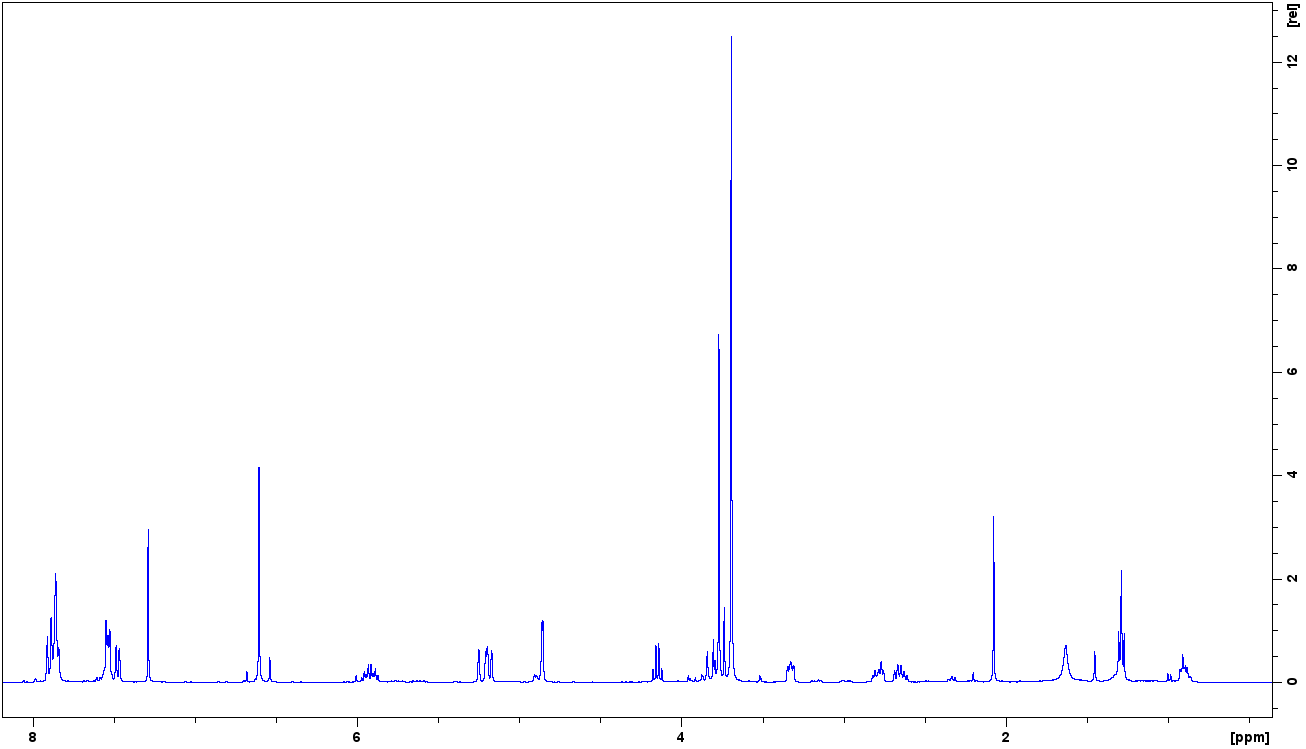

Supplement: Supplementary file 1 [file pharmaceuticals-16-01000-s001.zip › 10n mjm15940_1h.png]

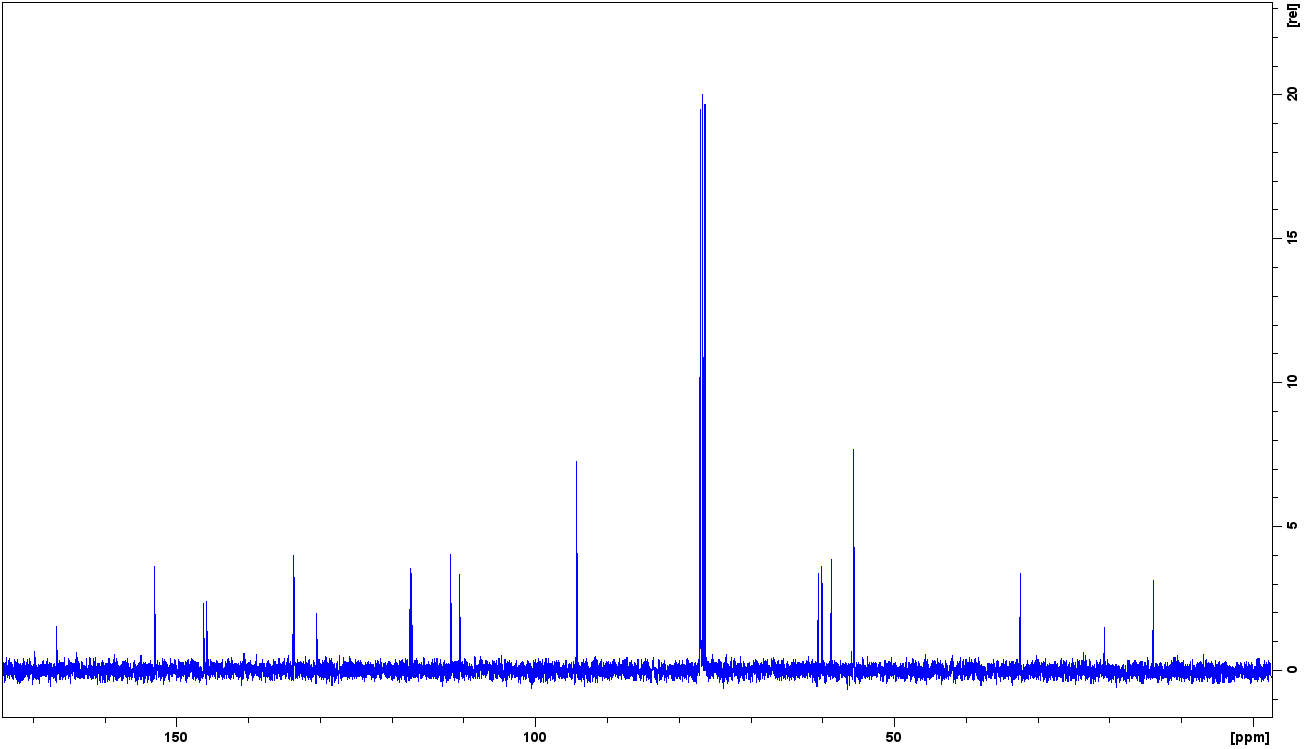

Supplement: Supplementary file 1 [file pharmaceuticals-16-01000-s001.zip › 10p mjm16513_13c.png]

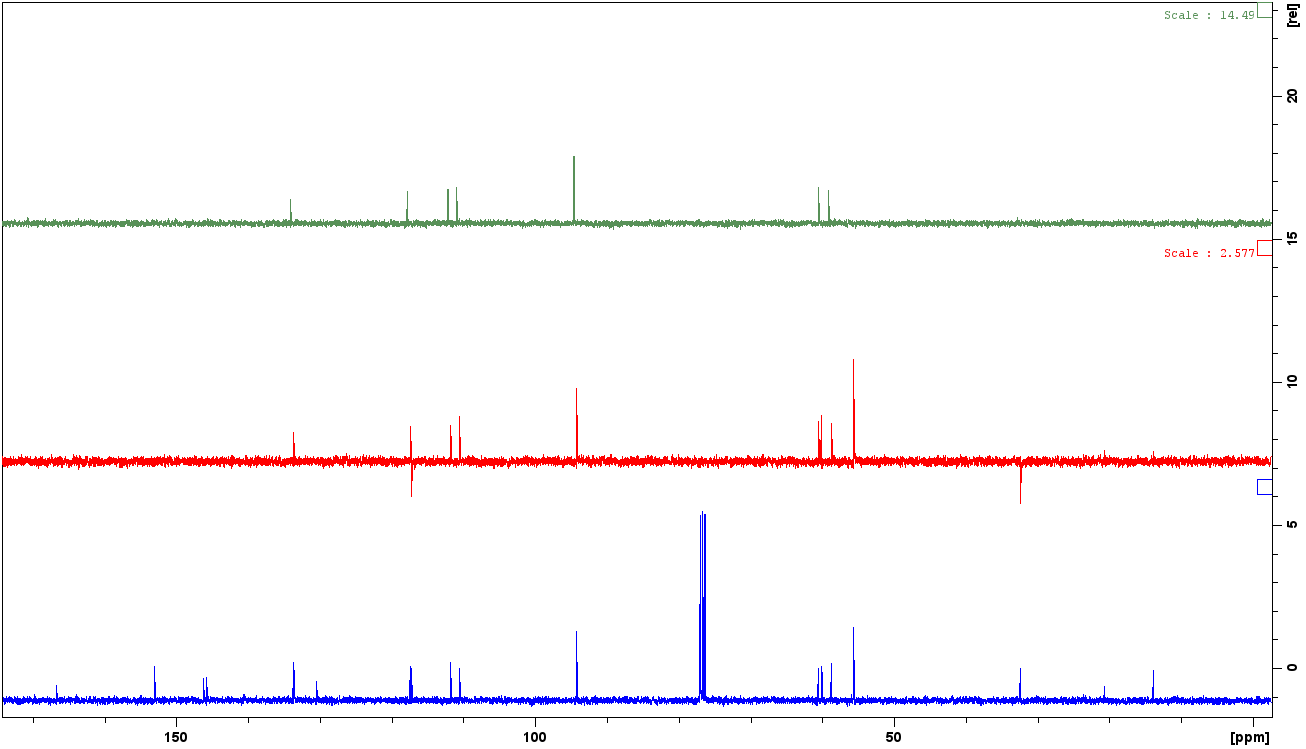

Supplement: Supplementary file 1 [file pharmaceuticals-16-01000-s001.zip › 10p mjm16513_13c_DEPTs.png]

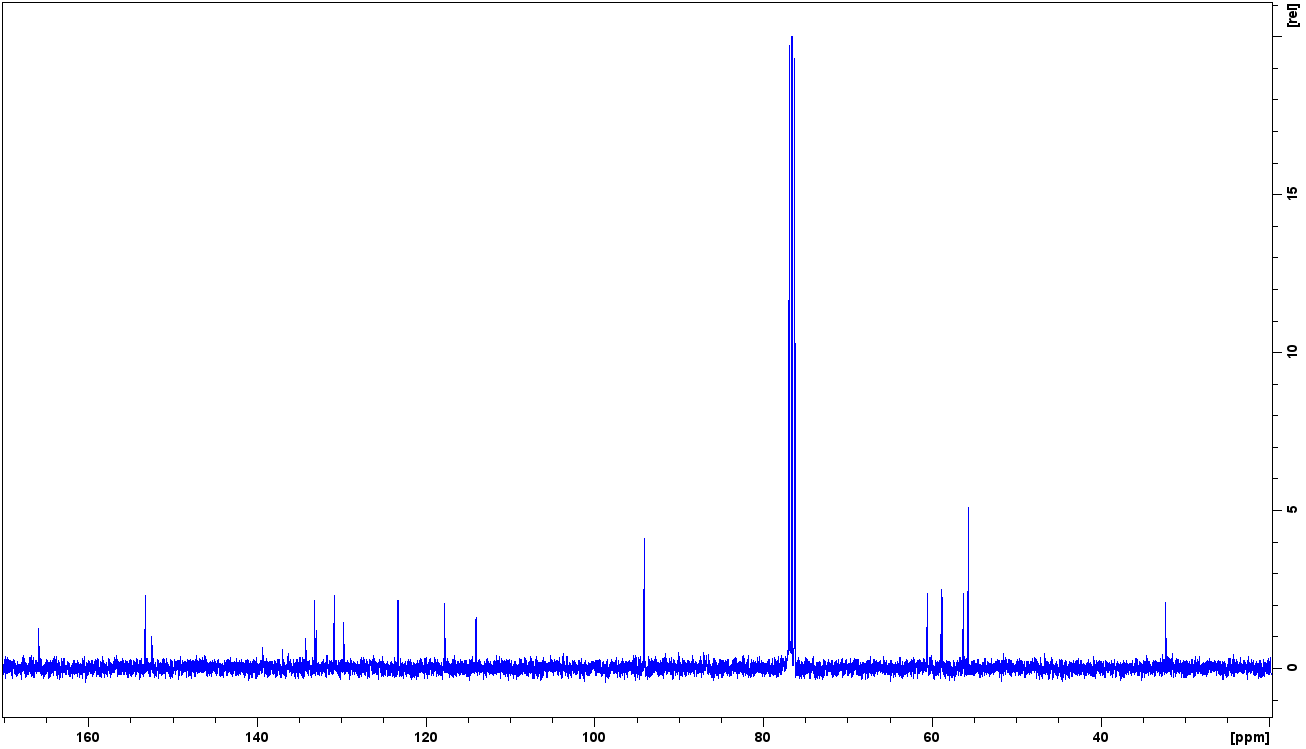

Supplement: Supplementary file 1 [file pharmaceuticals-16-01000-s001.zip › 10q mjm16806_13c.png]

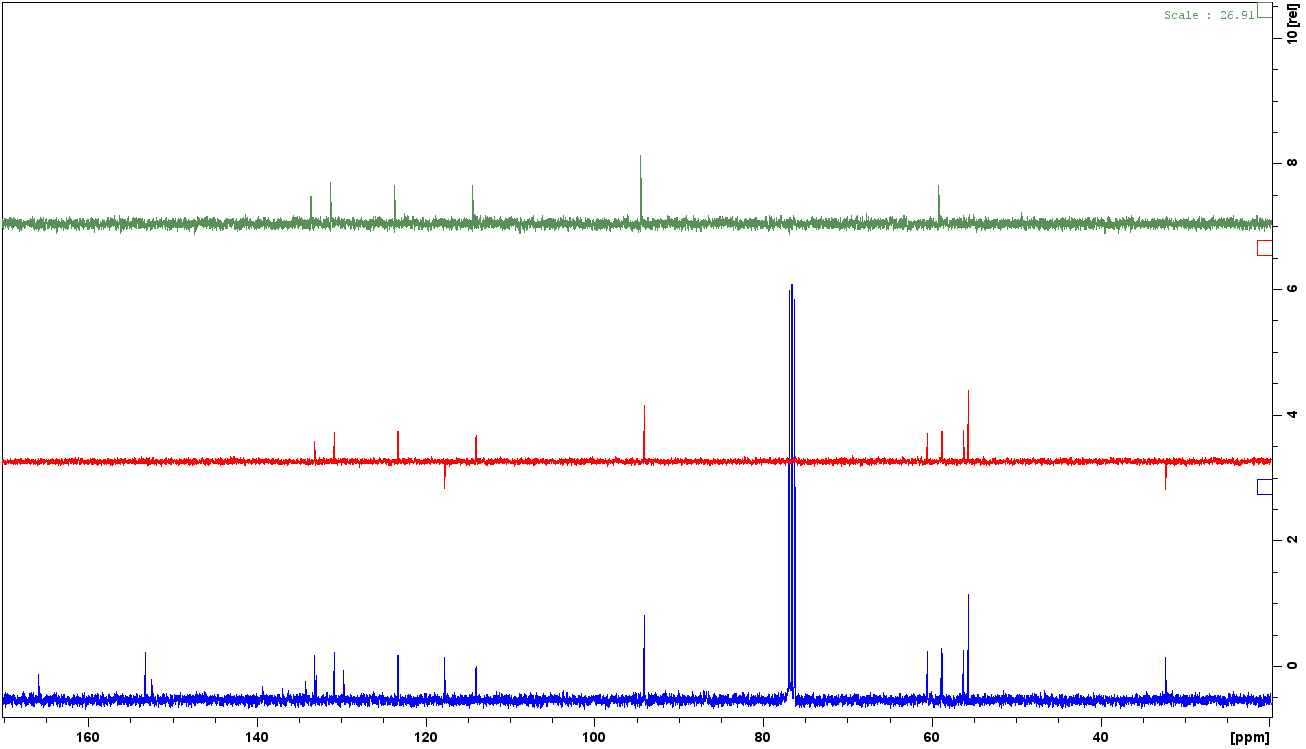

Supplement: Supplementary file 1 [file pharmaceuticals-16-01000-s001.zip › 10q mjm16806_13c_DEPTs.png]

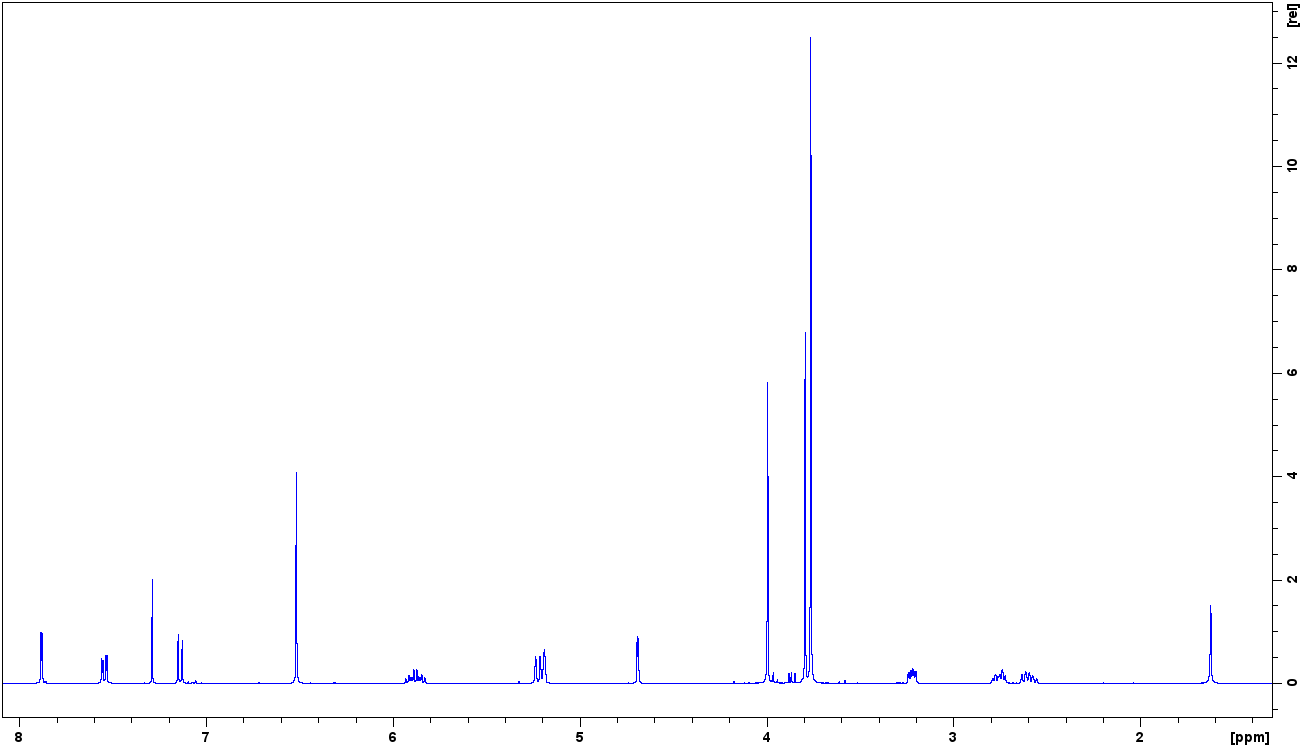

Supplement: Supplementary file 1 [file pharmaceuticals-16-01000-s001.zip › 10q mjm16806_1h.png]

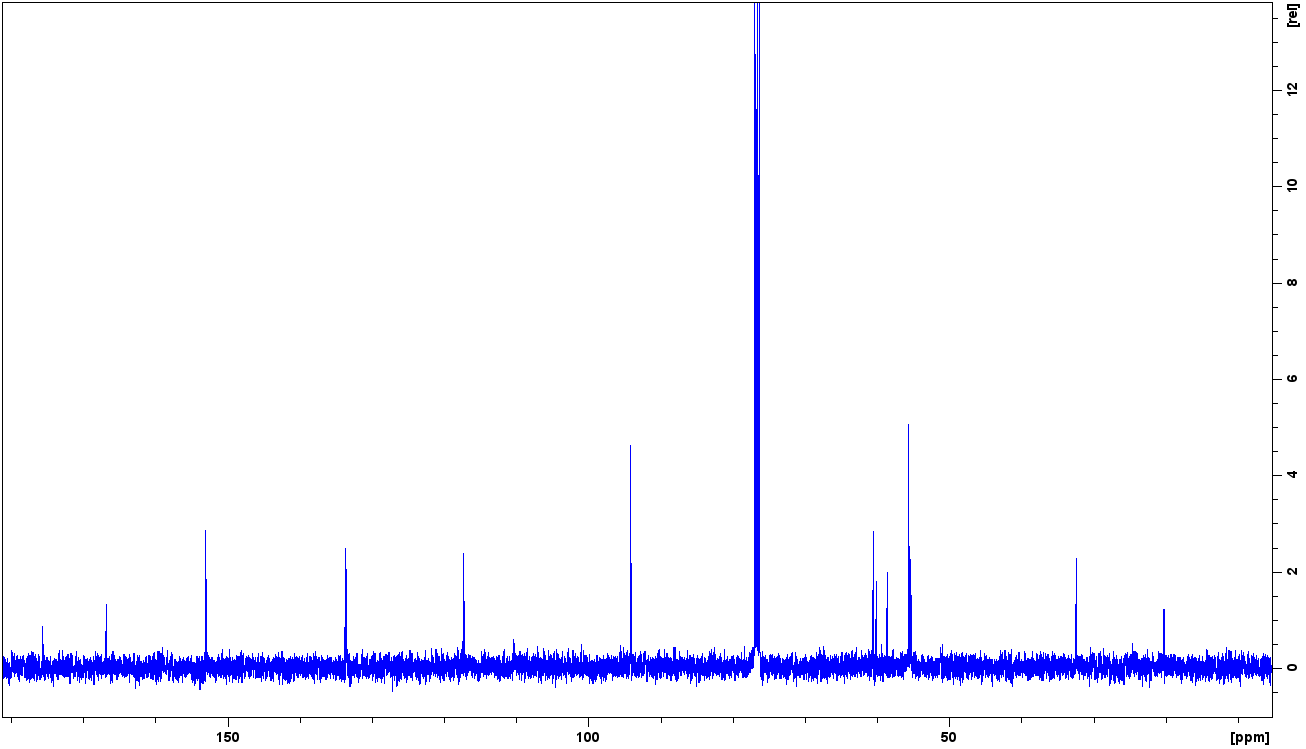

Supplement: Supplementary file 1 [file pharmaceuticals-16-01000-s001.zip › 10r mjm16845_13c.png]

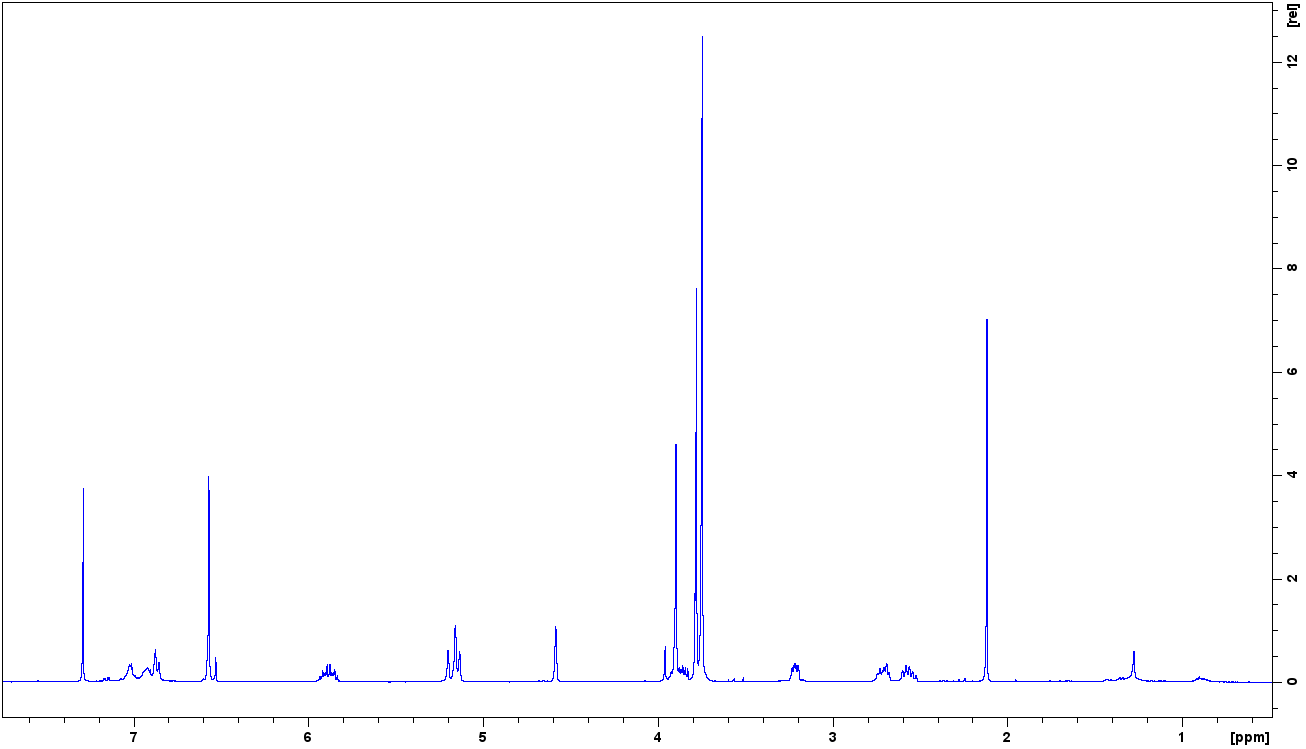

Supplement: Supplementary file 1 [file pharmaceuticals-16-01000-s001.zip › 10r mjm16845_1h.png]

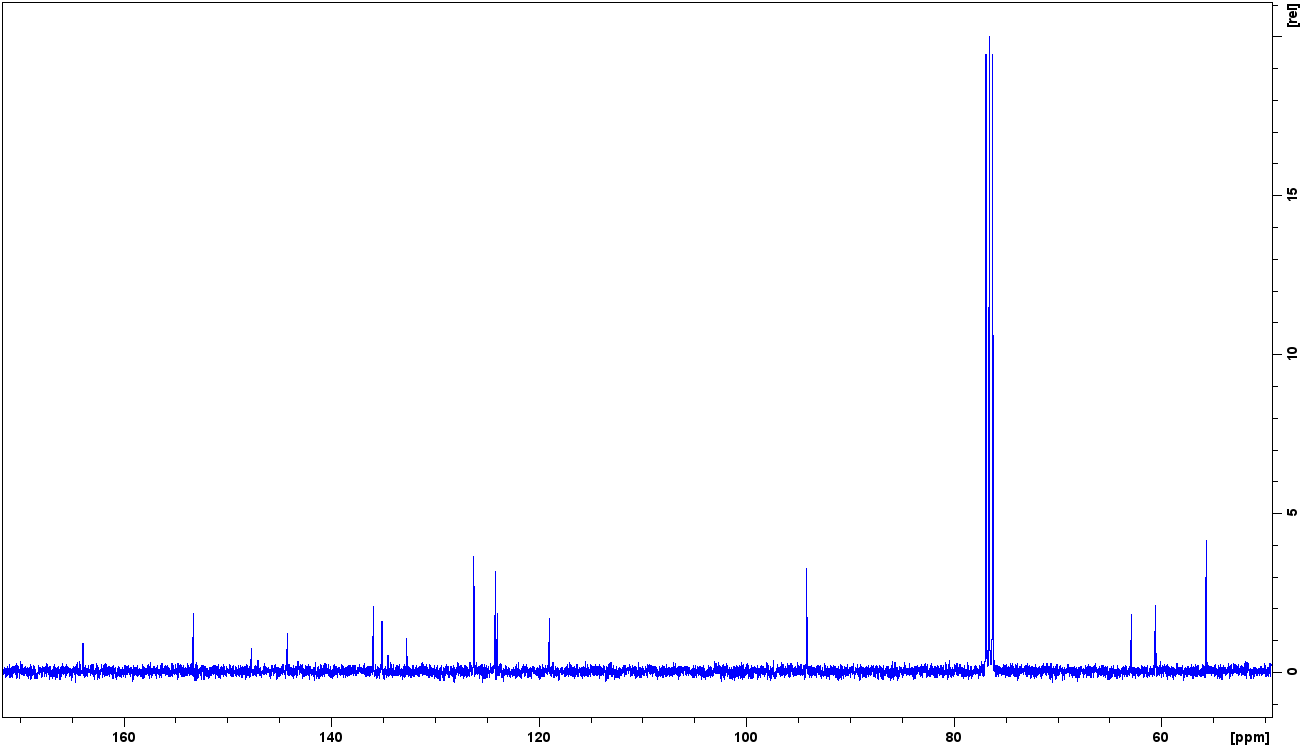

Supplement: Supplementary file 1 [file pharmaceuticals-16-01000-s001.zip › 11a mjm16205_13.png]

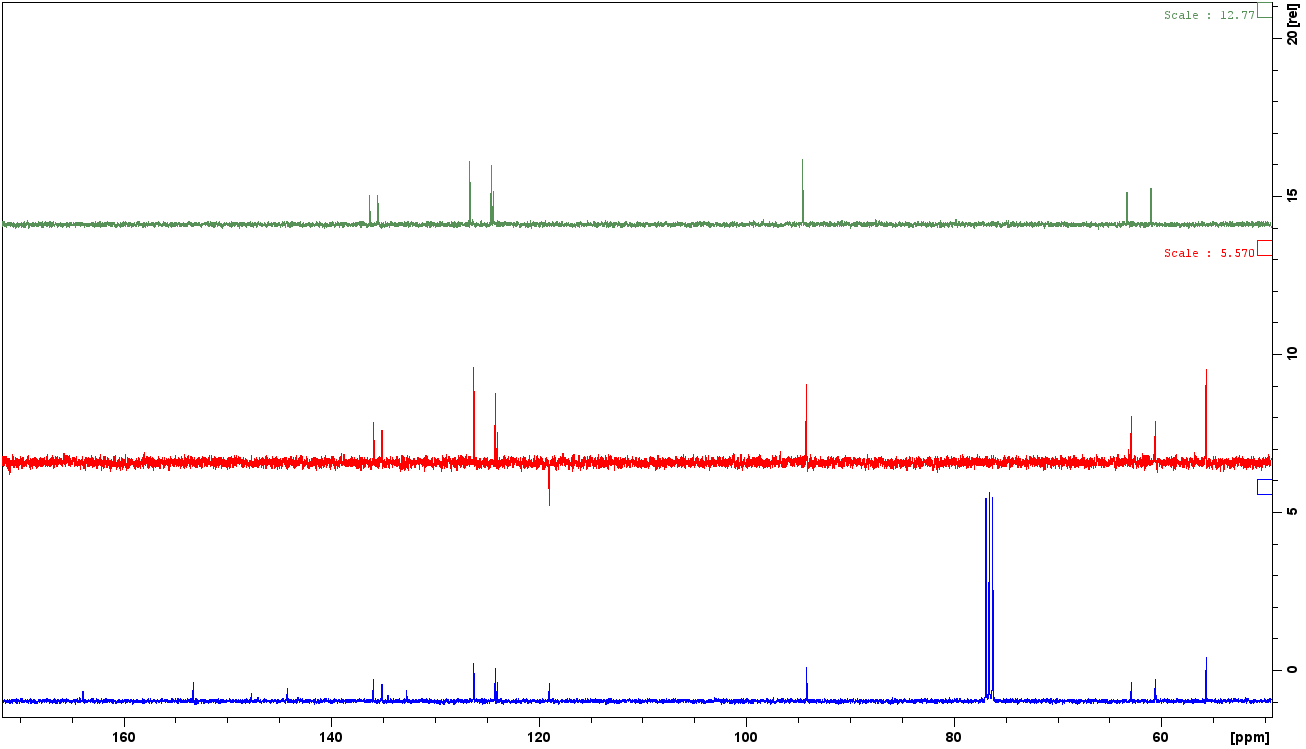

Supplement: Supplementary file 1 [file pharmaceuticals-16-01000-s001.zip › 11a mjm16205_13c_DEPTs.png]

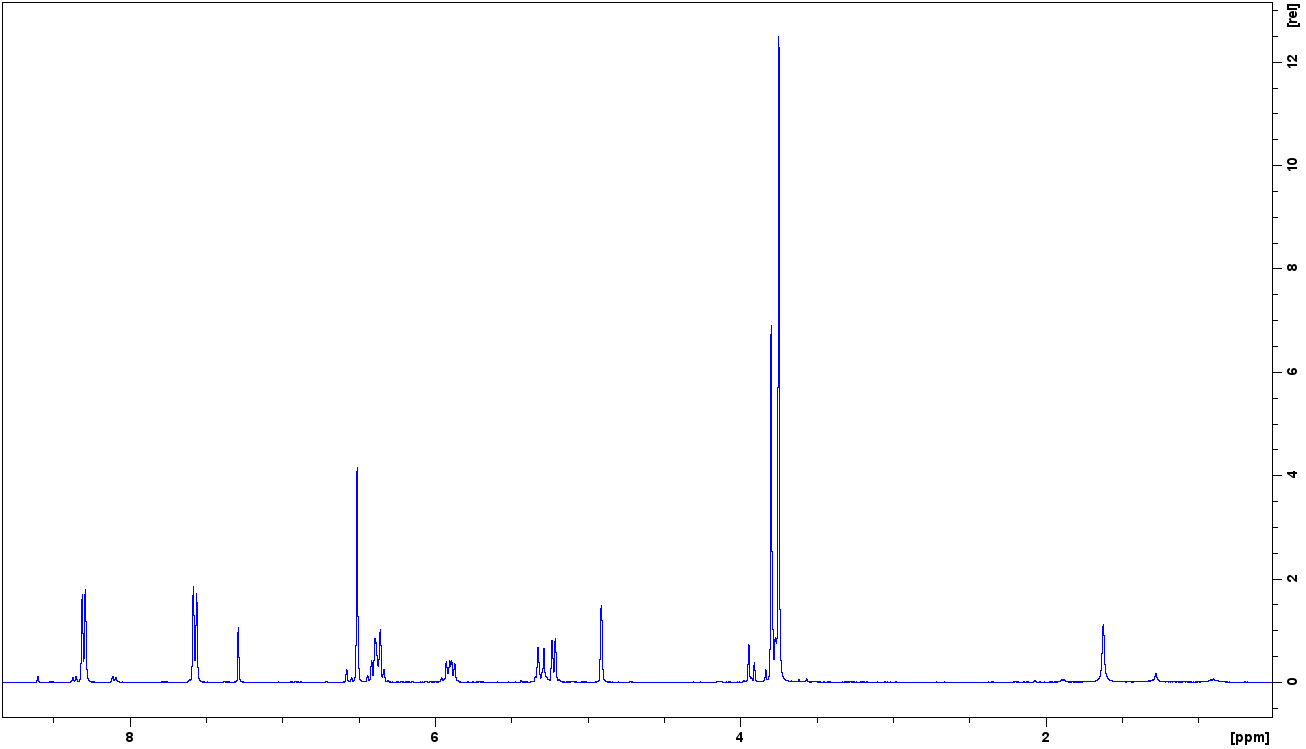

Supplement: Supplementary file 1 [file pharmaceuticals-16-01000-s001.zip › 11a mjm16205_1h.png]

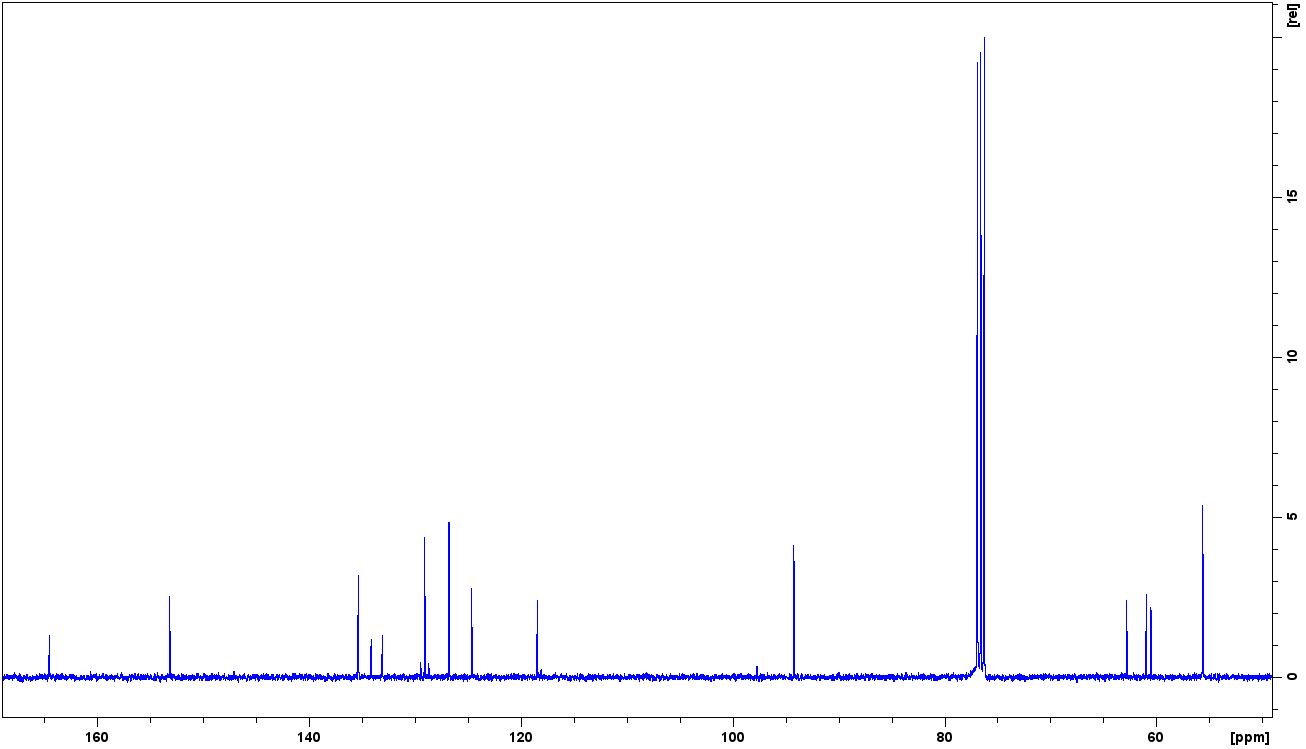

Supplement: Supplementary file 1 [file pharmaceuticals-16-01000-s001.zip › 11b mjm16202_13c.png]

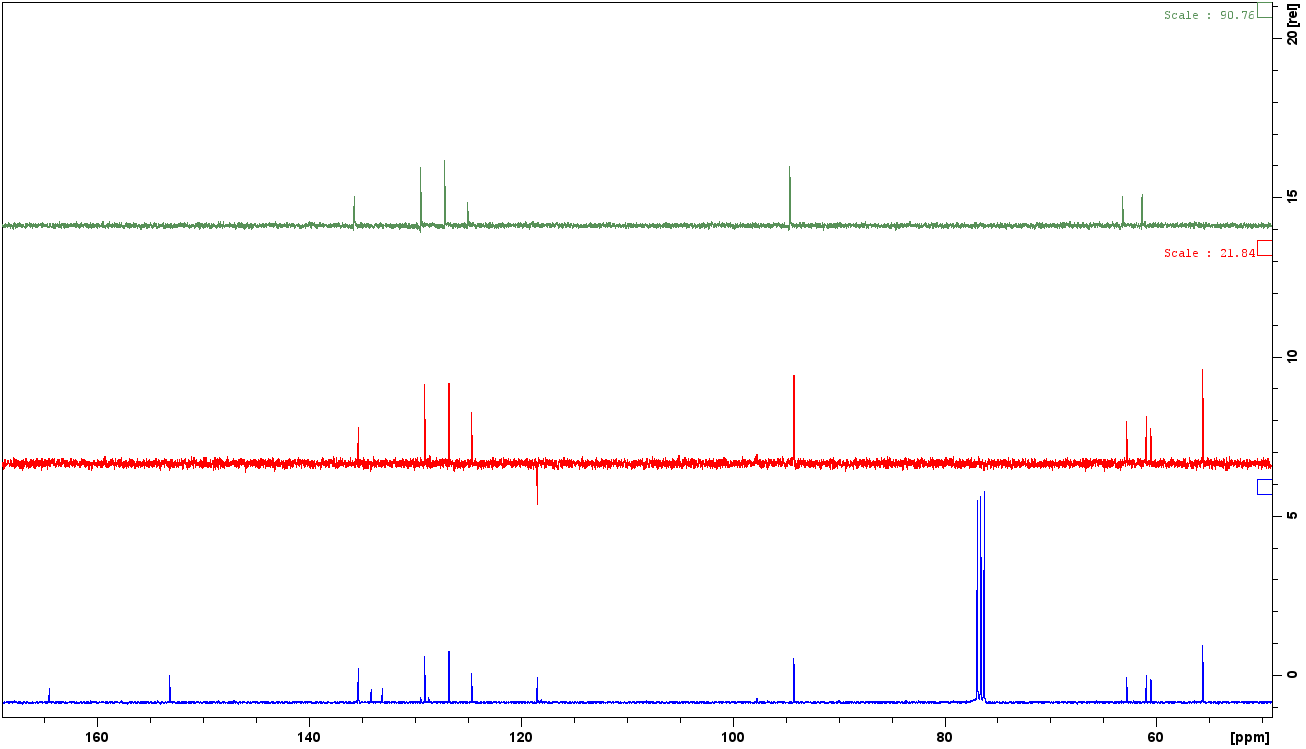

Supplement: Supplementary file 1 [file pharmaceuticals-16-01000-s001.zip › 11b mjm16202_13c_DEPTs.png]

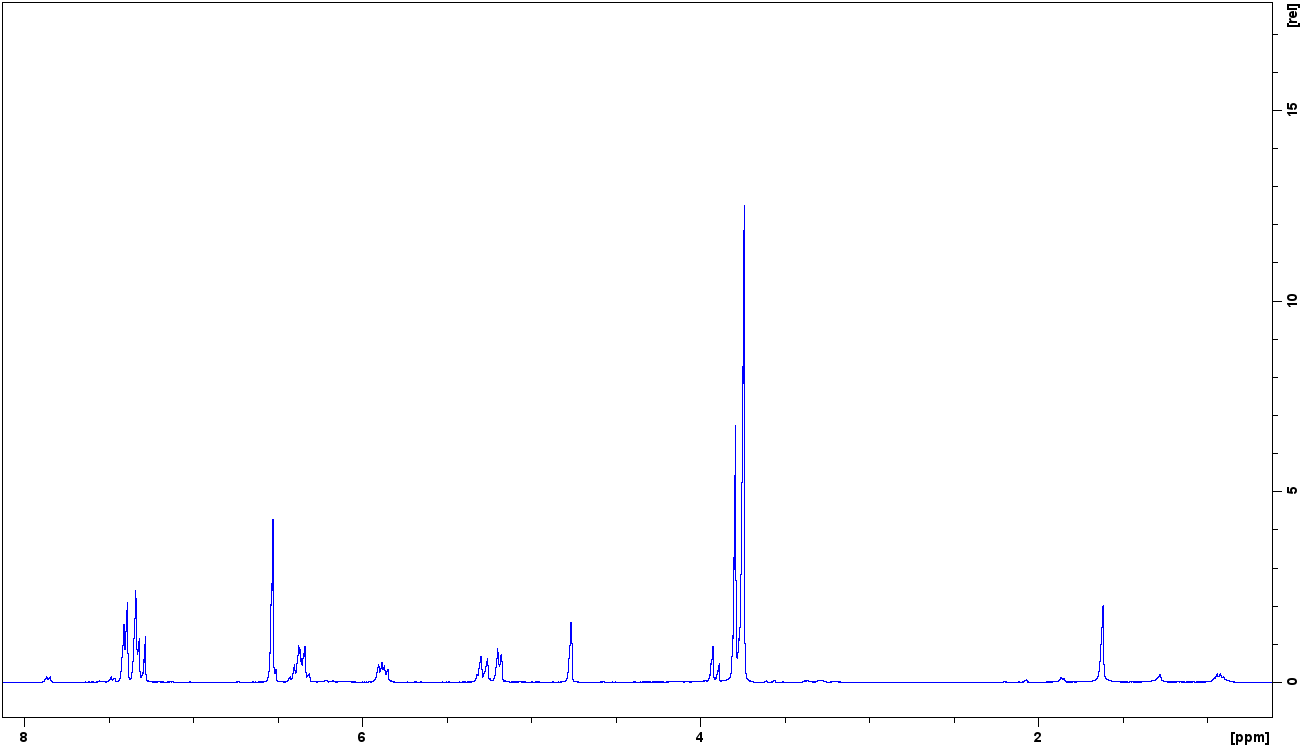

Supplement: Supplementary file 1 [file pharmaceuticals-16-01000-s001.zip › 11b mjm16202_1h.png]

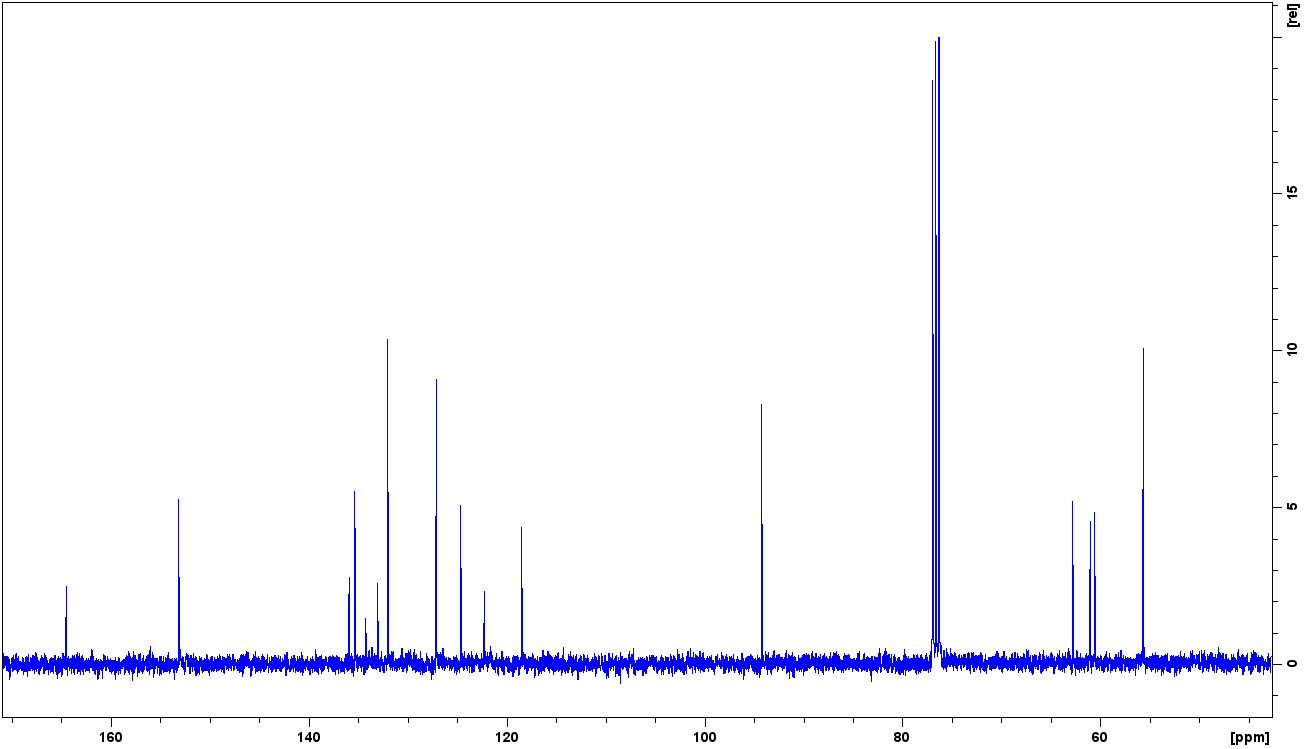

Supplement: Supplementary file 1 [file pharmaceuticals-16-01000-s001.zip › 11c mjm16119_13c.png]

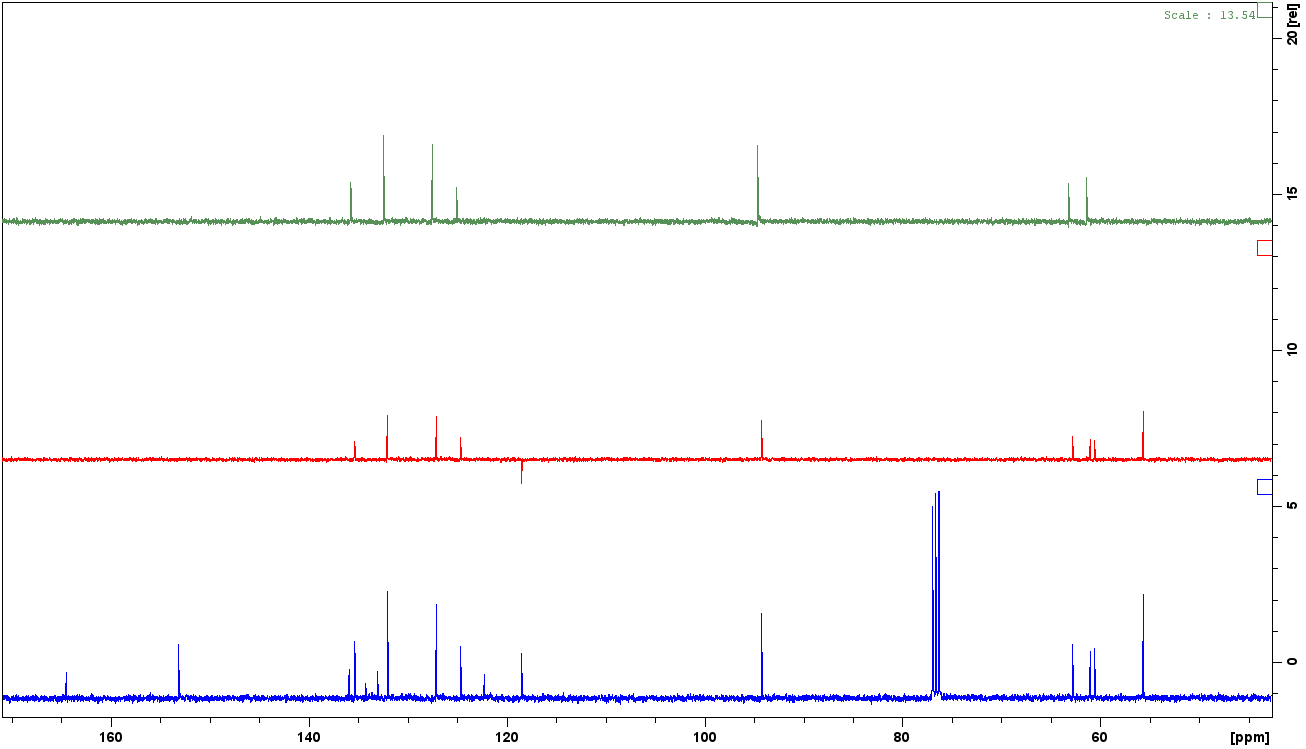

Supplement: Supplementary file 1 [file pharmaceuticals-16-01000-s001.zip › 11c mjm16119_13c_DEPTs.png]

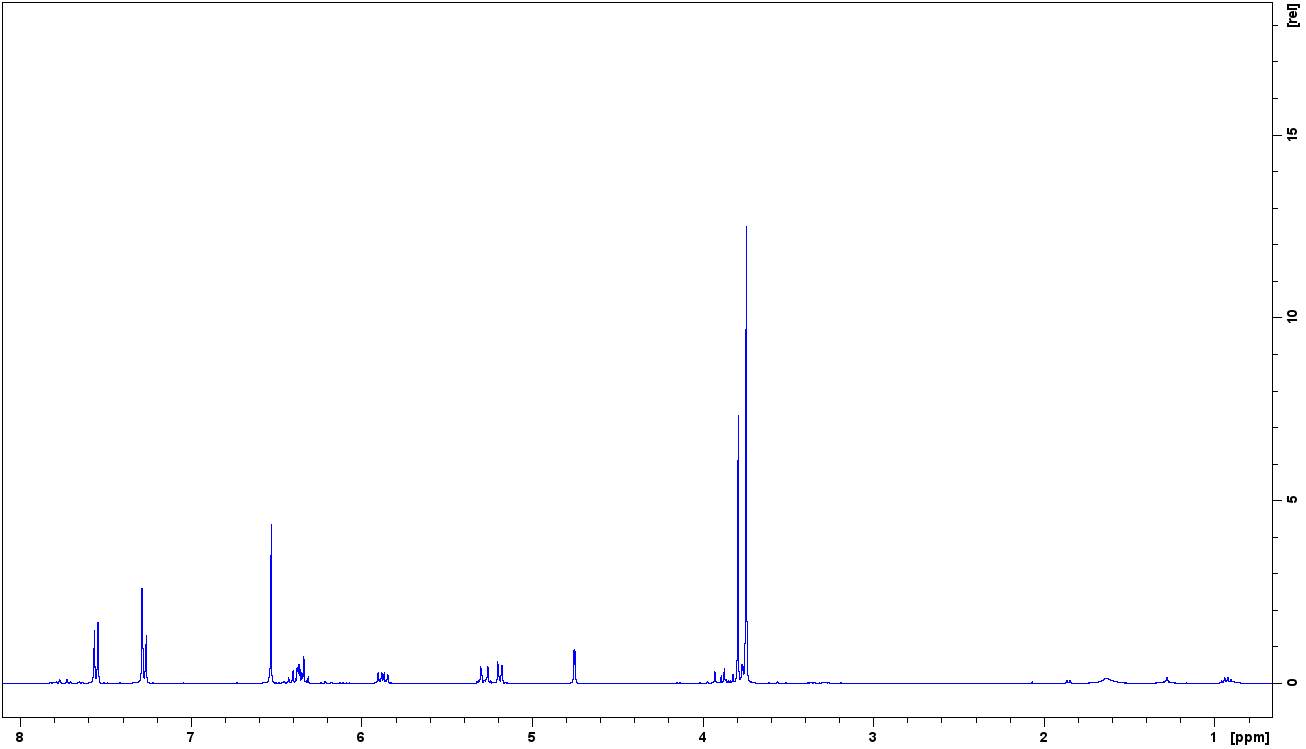

Supplement: Supplementary file 1 [file pharmaceuticals-16-01000-s001.zip › 11c mjm16119_1h.png]

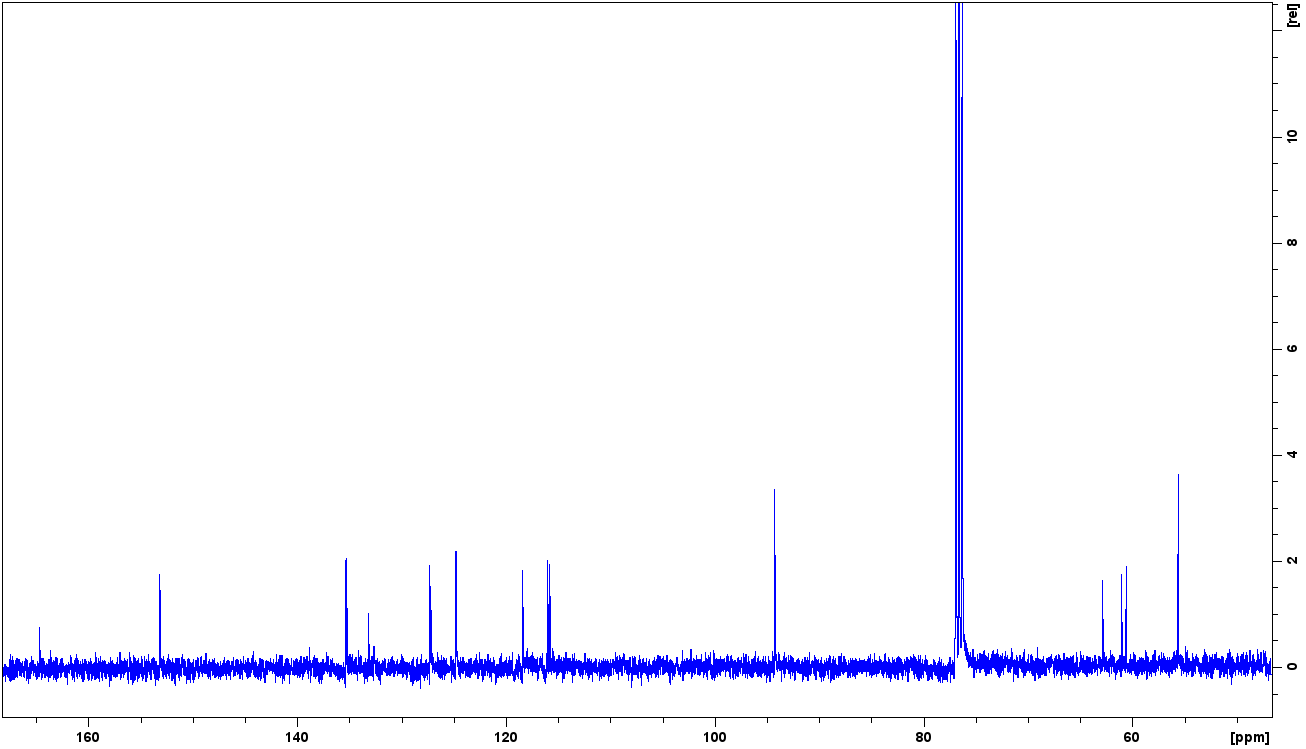

Supplement: Supplementary file 1 [file pharmaceuticals-16-01000-s001.zip › 11d mjm16102_13c.png]

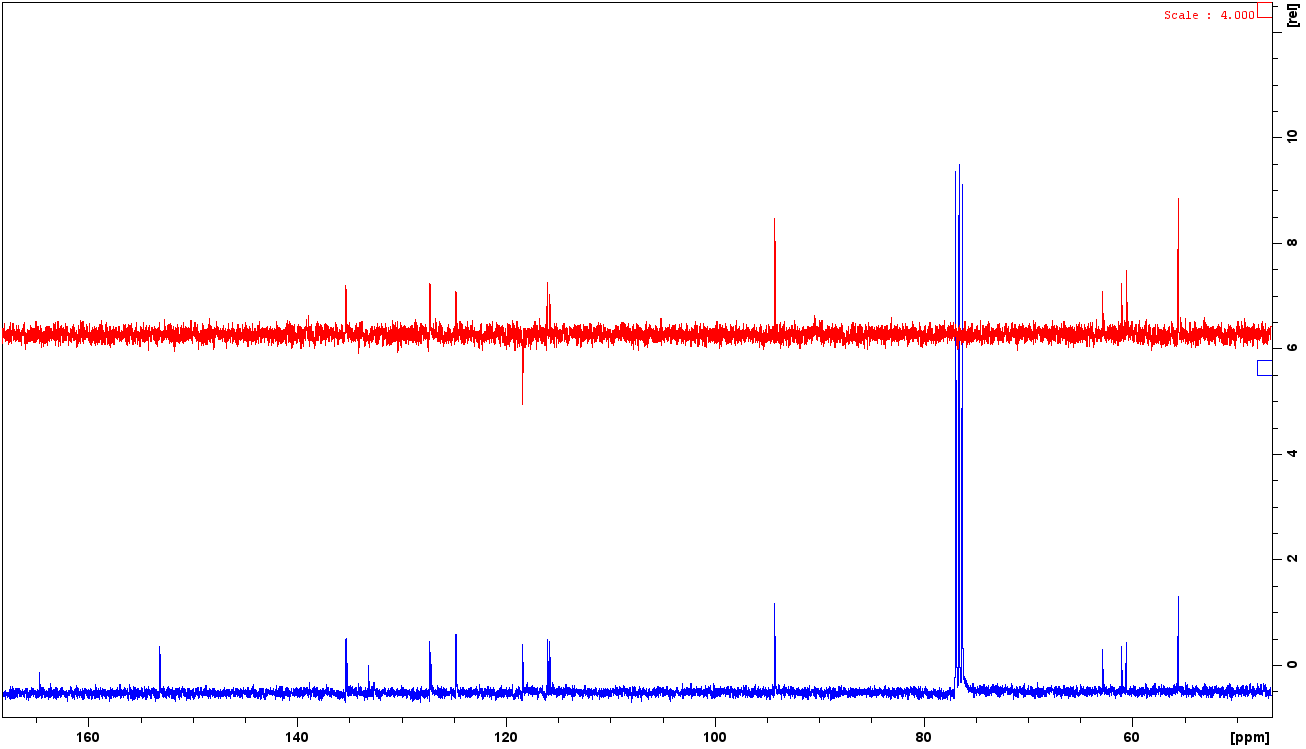

Supplement: Supplementary file 1 [file pharmaceuticals-16-01000-s001.zip › 11d mjm16102_13c_DEPT.png]

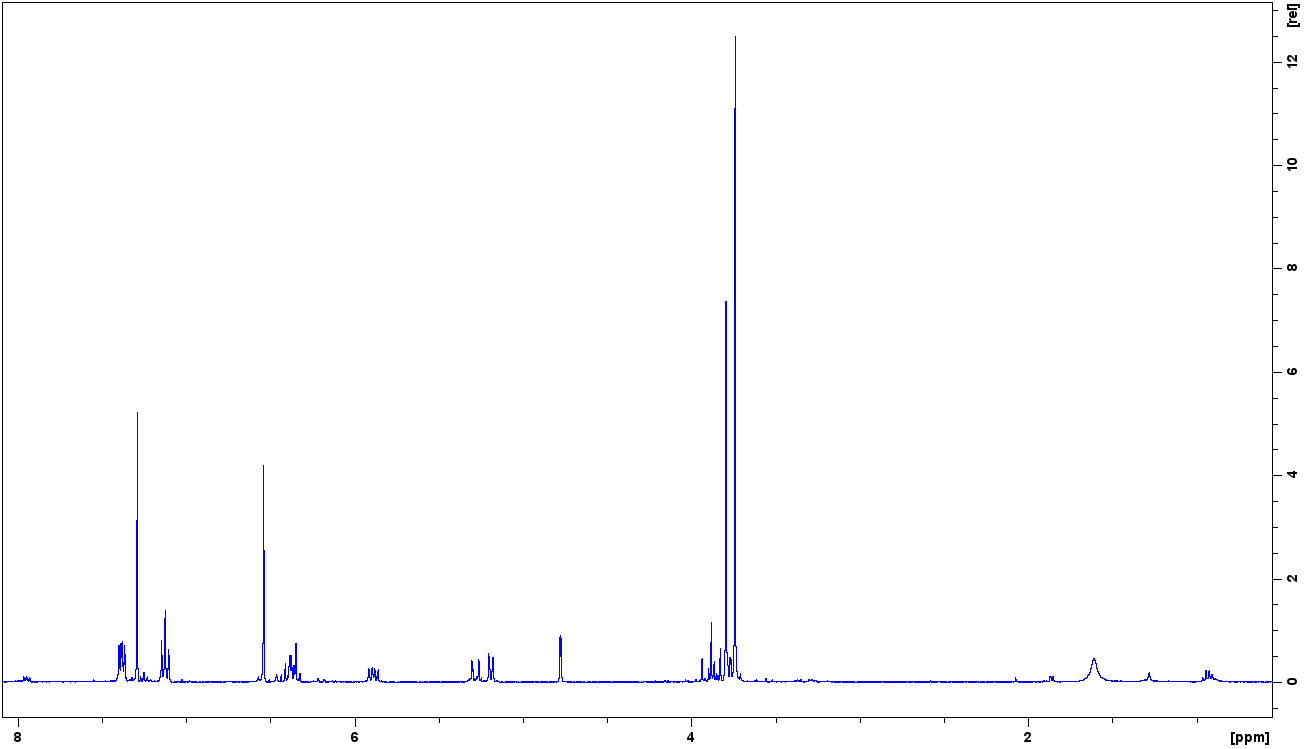

Supplement: Supplementary file 1 [file pharmaceuticals-16-01000-s001.zip › 11d mjm16102_1h.png]

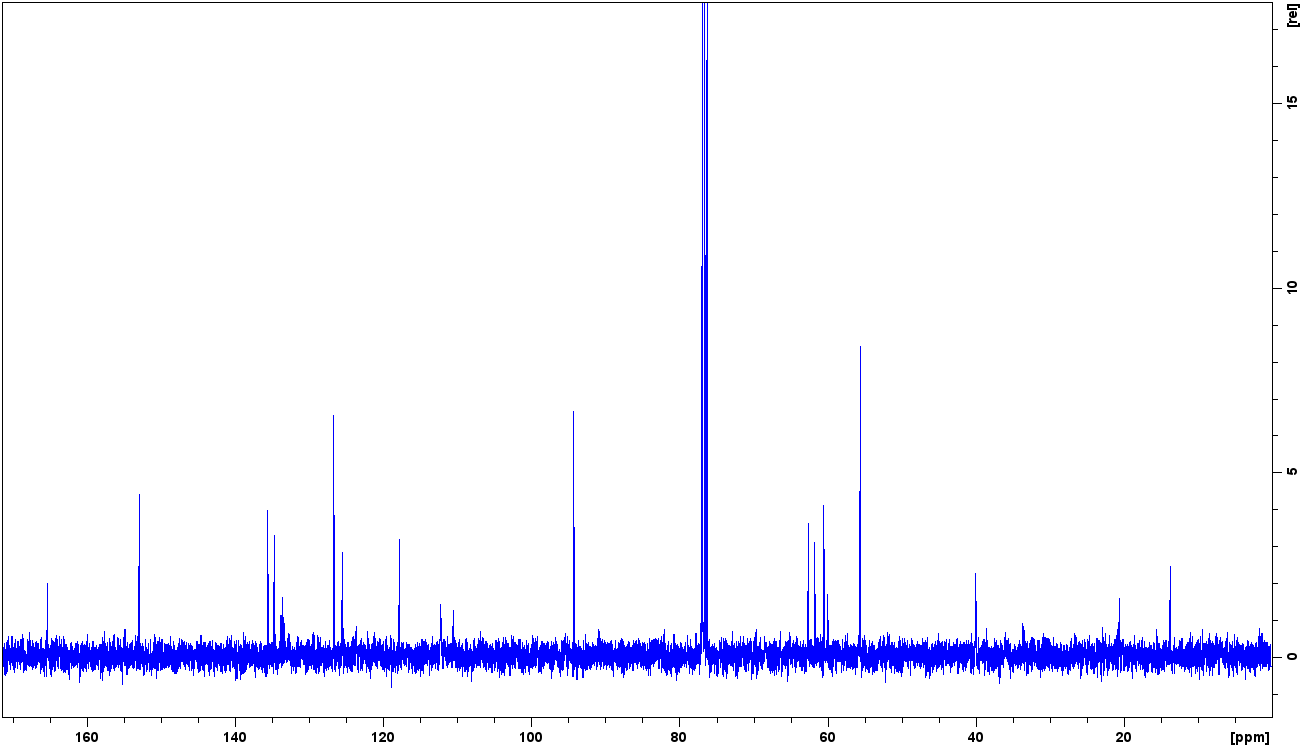

Supplement: Supplementary file 1 [file pharmaceuticals-16-01000-s001.zip › 11e mjm16133_13c.png]

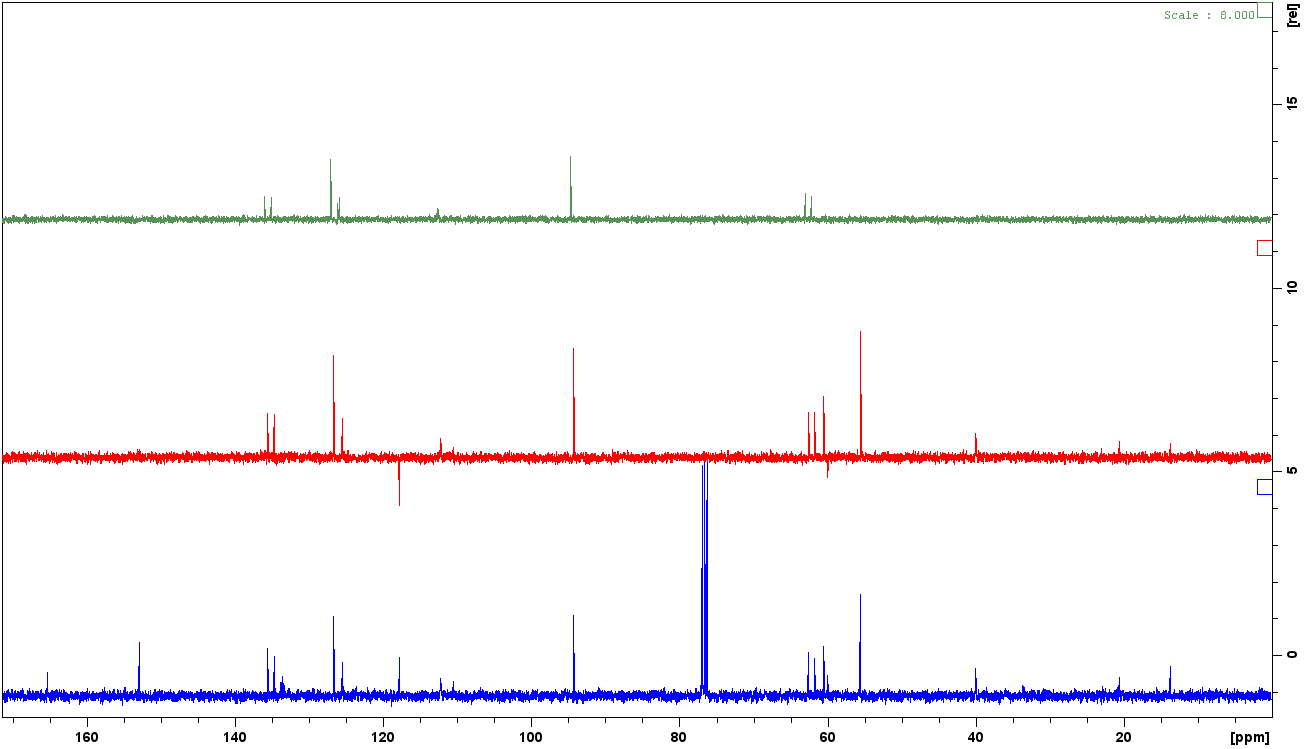

Supplement: Supplementary file 1 [file pharmaceuticals-16-01000-s001.zip › 11e mjm16133_13c_DEPTs.png]

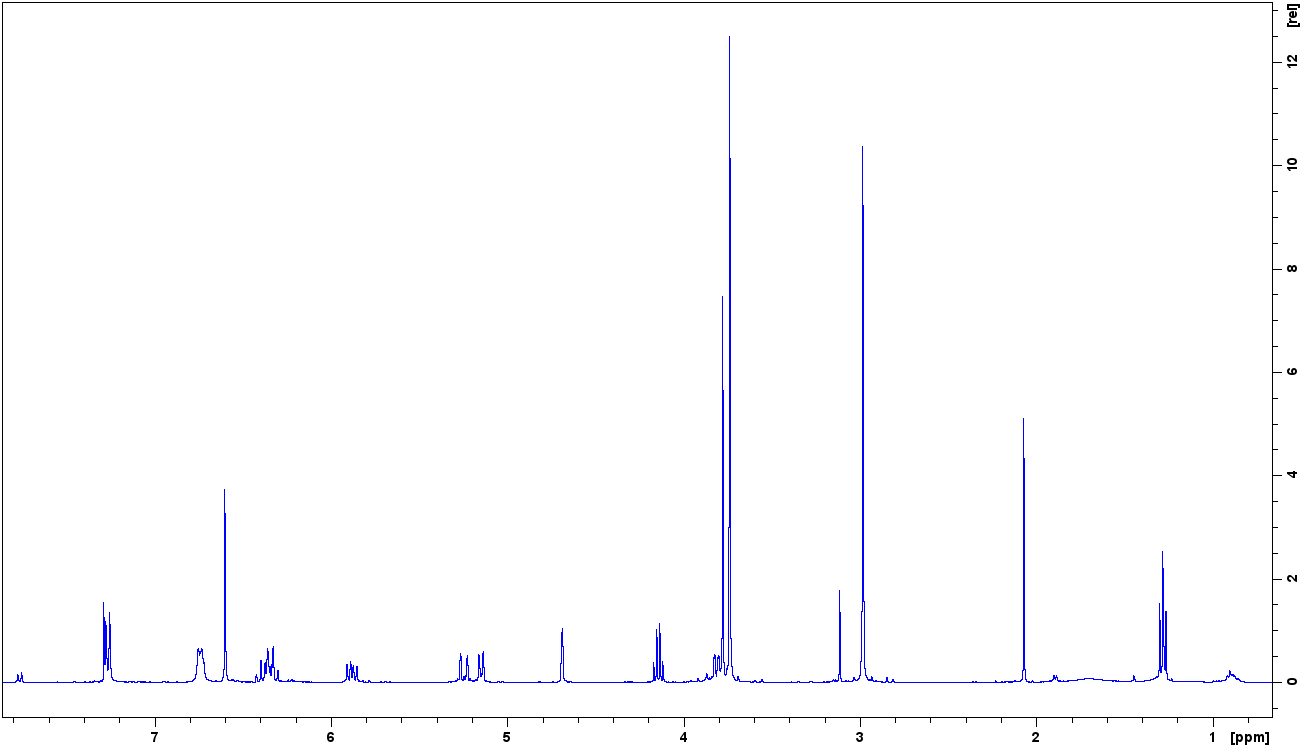

Supplement: Supplementary file 1 [file pharmaceuticals-16-01000-s001.zip › 11e mjm16133_1h.png]

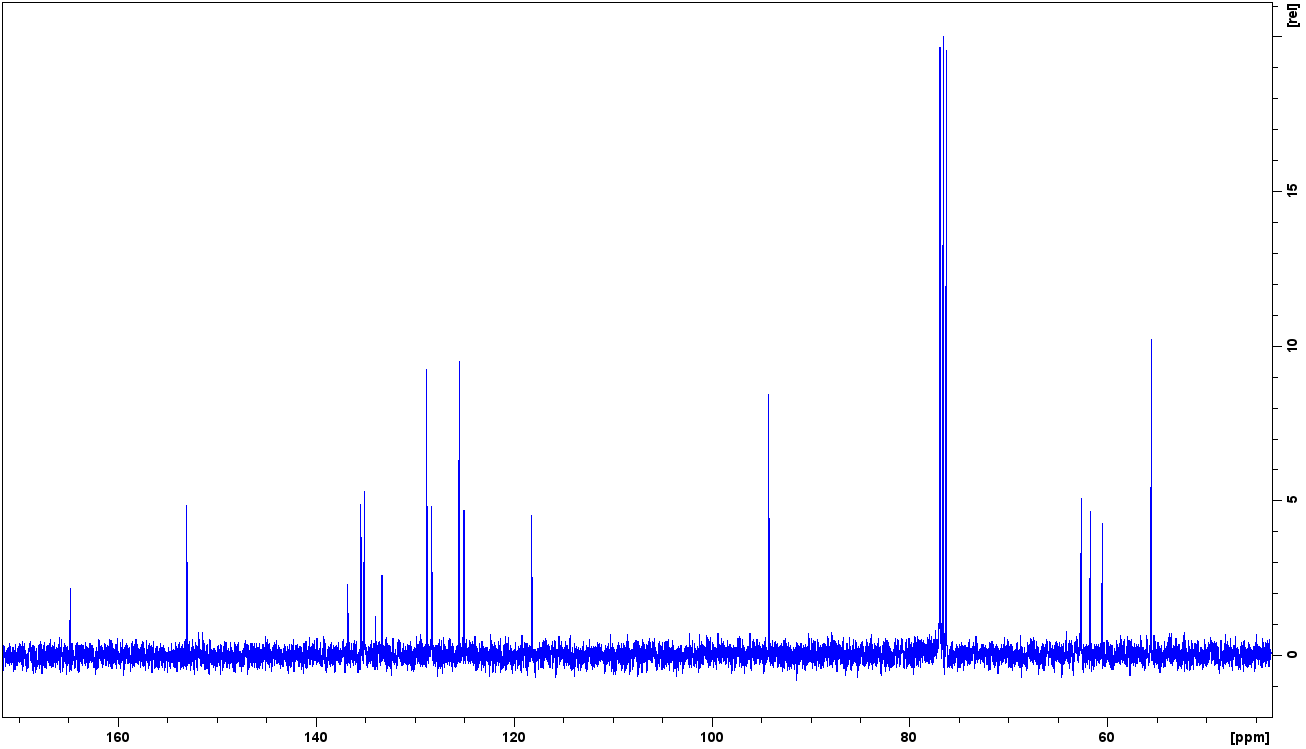

Supplement: Supplementary file 1 [file pharmaceuticals-16-01000-s001.zip › 11f mjm16136_13c.png]

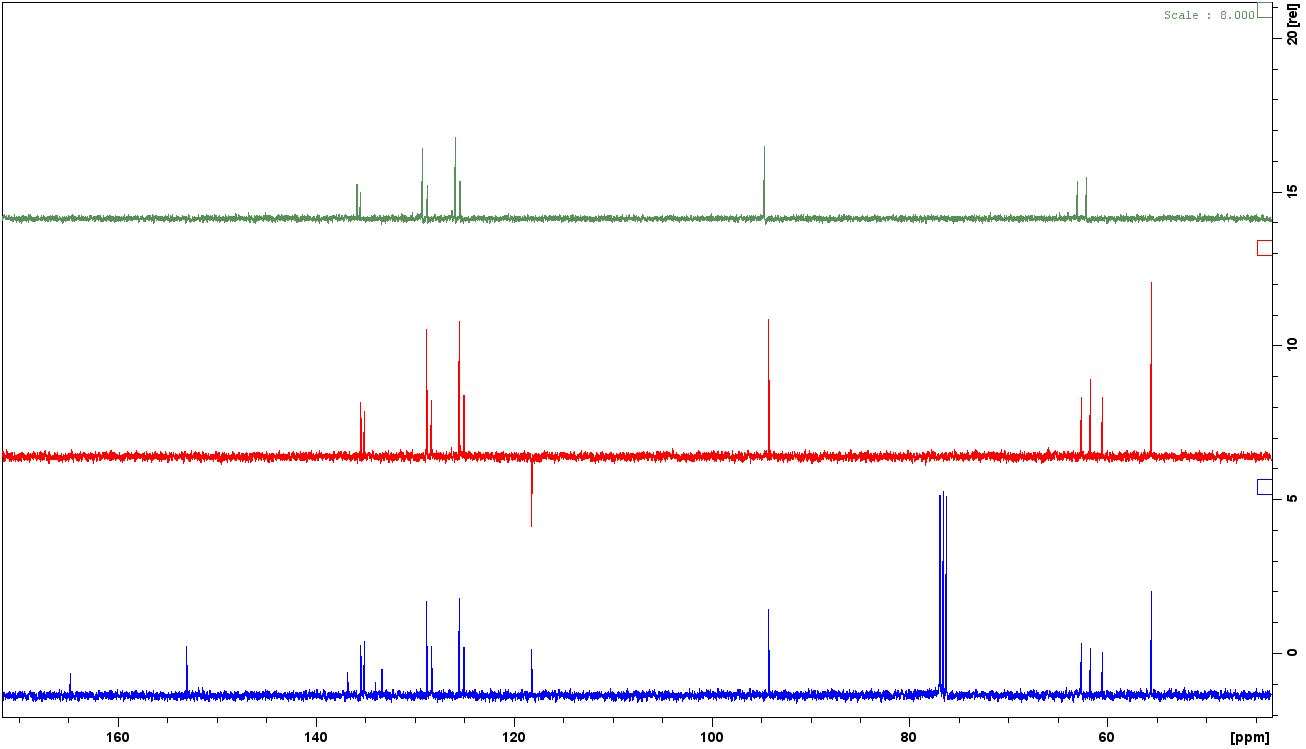

Supplement: Supplementary file 1 [file pharmaceuticals-16-01000-s001.zip › 11f mjm16136_13c_DEPTs.png]

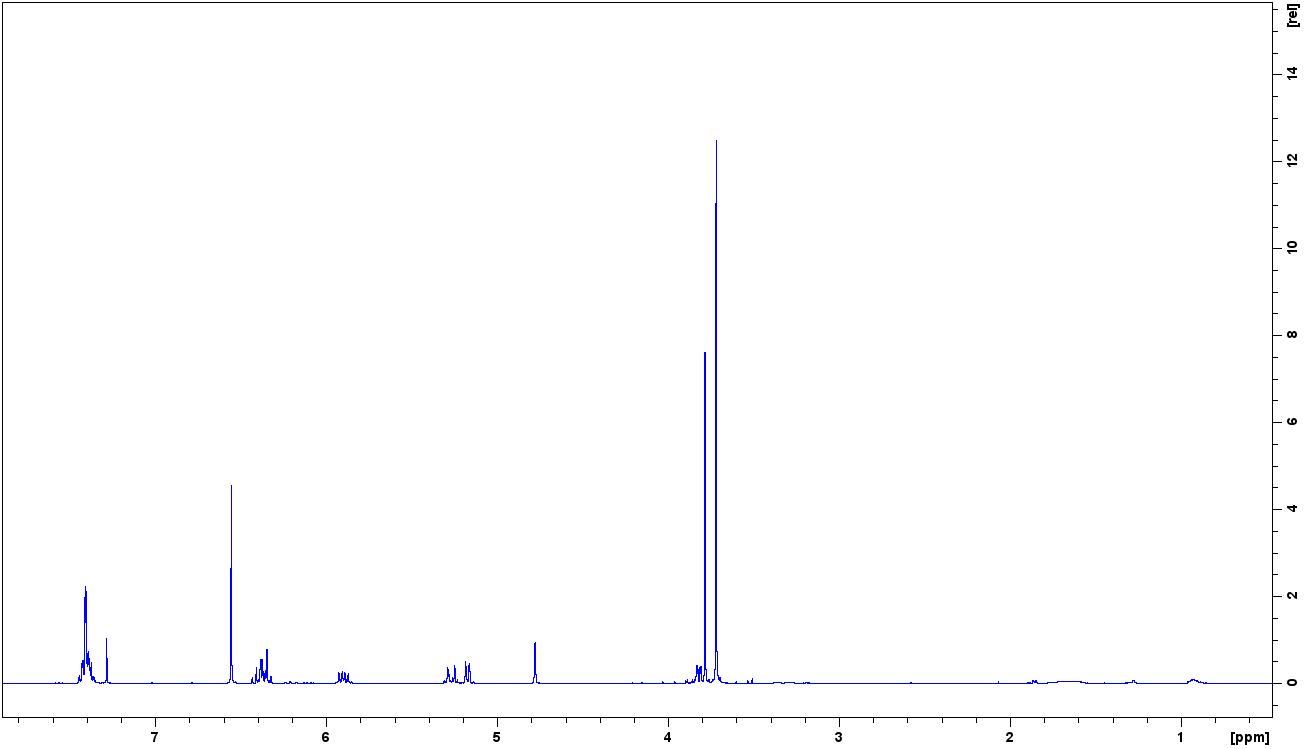

Supplement: Supplementary file 1 [file pharmaceuticals-16-01000-s001.zip › 11f mjm16136_1h.png]

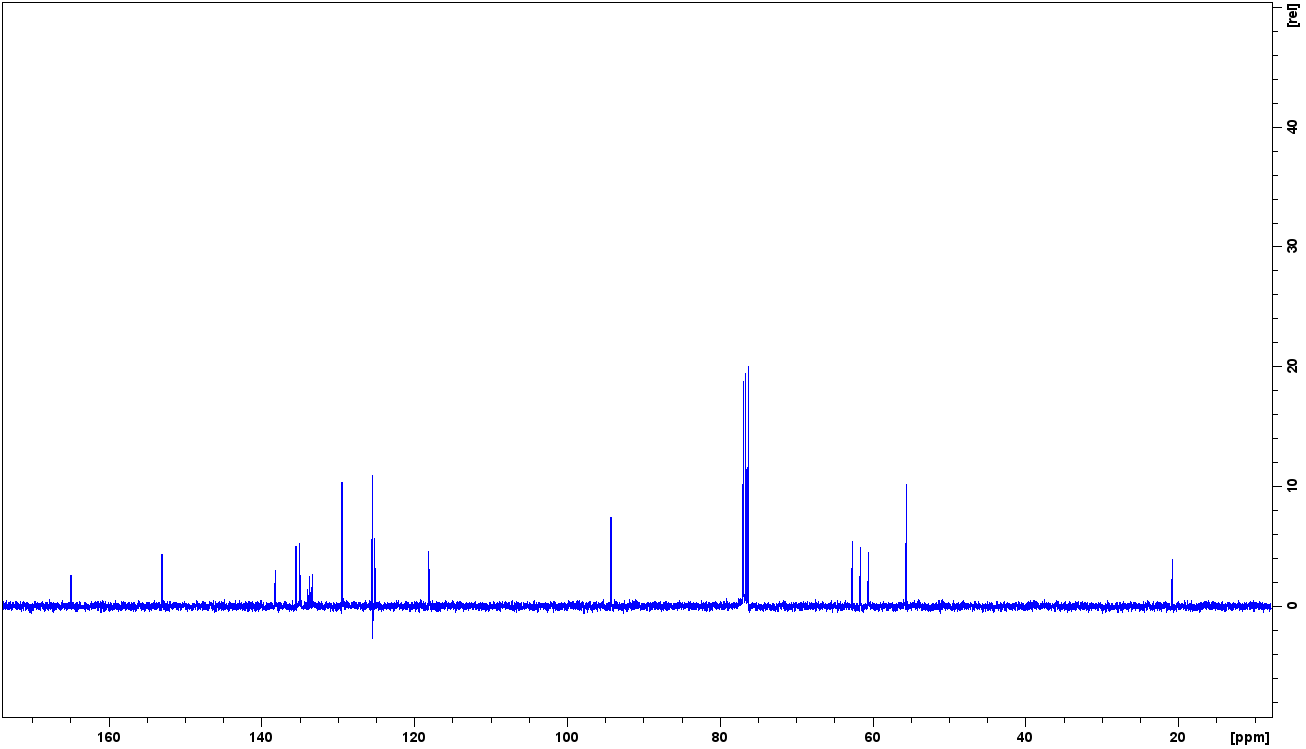

Supplement: Supplementary file 1 [file pharmaceuticals-16-01000-s001.zip › 11g mjm16232_13c.png]

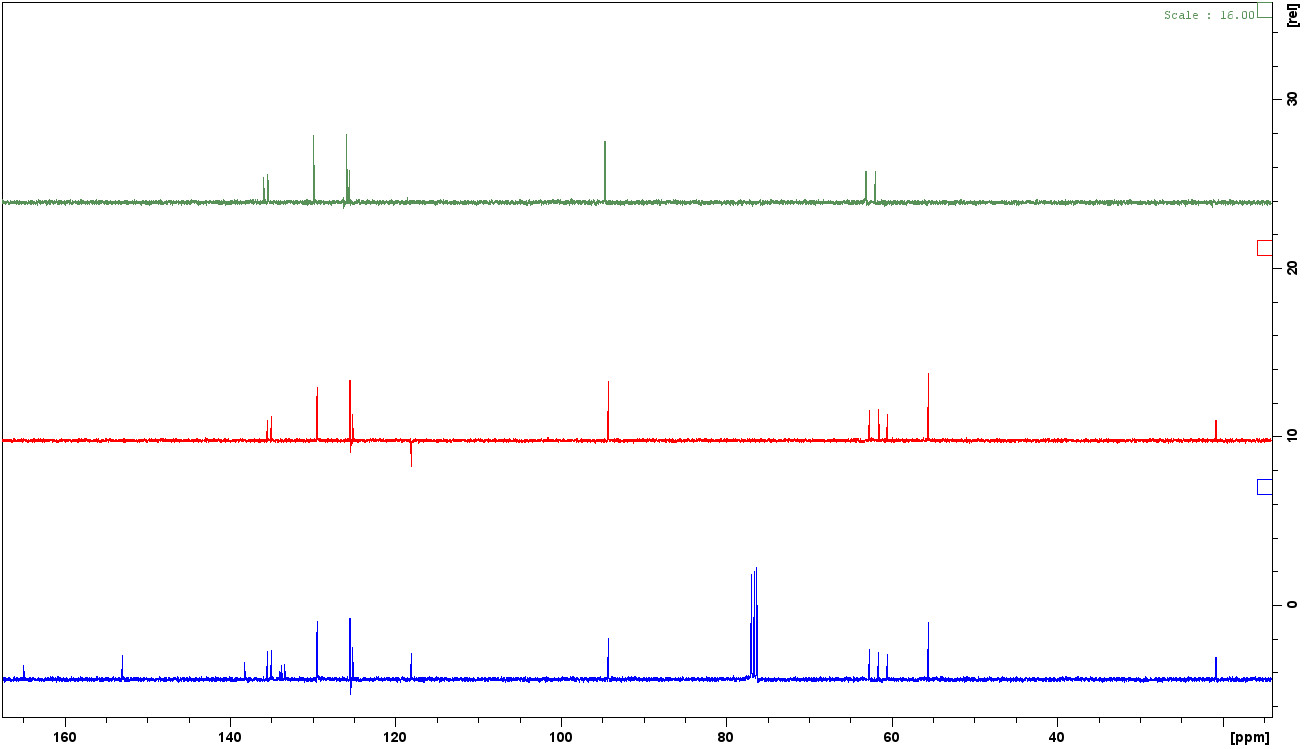

Supplement: Supplementary file 1 [file pharmaceuticals-16-01000-s001.zip › 11g mjm16232_13cDEPTs.png]

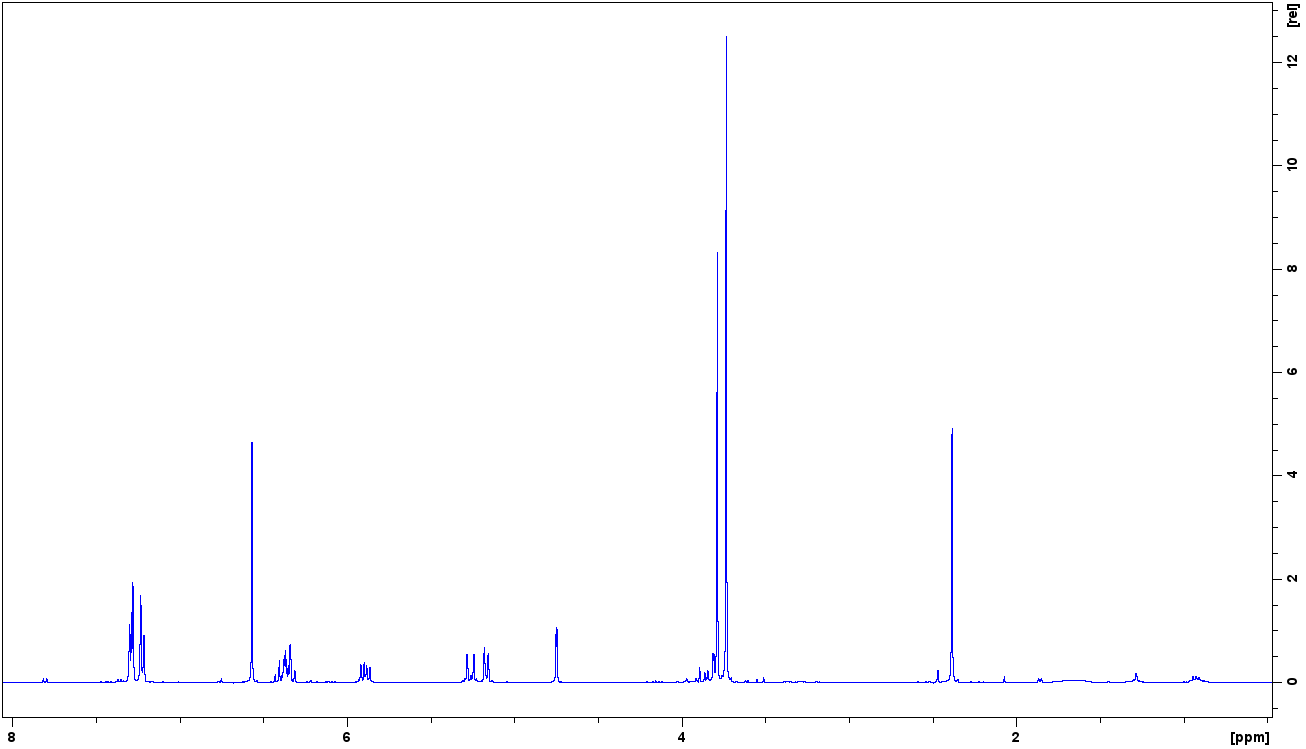

Supplement: Supplementary file 1 [file pharmaceuticals-16-01000-s001.zip › 11g mjm16232_1h.png]

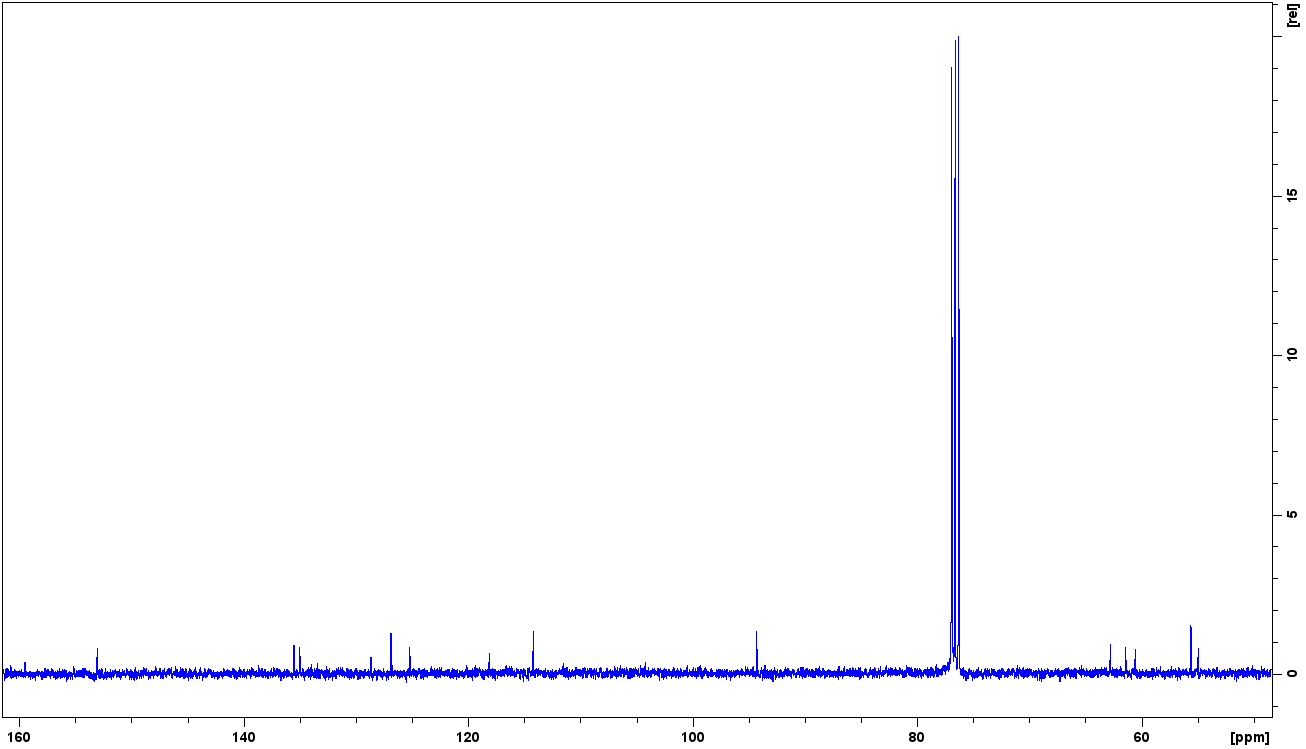

Supplement: Supplementary file 1 [file pharmaceuticals-16-01000-s001.zip › 11h mjm16070_13c.png]

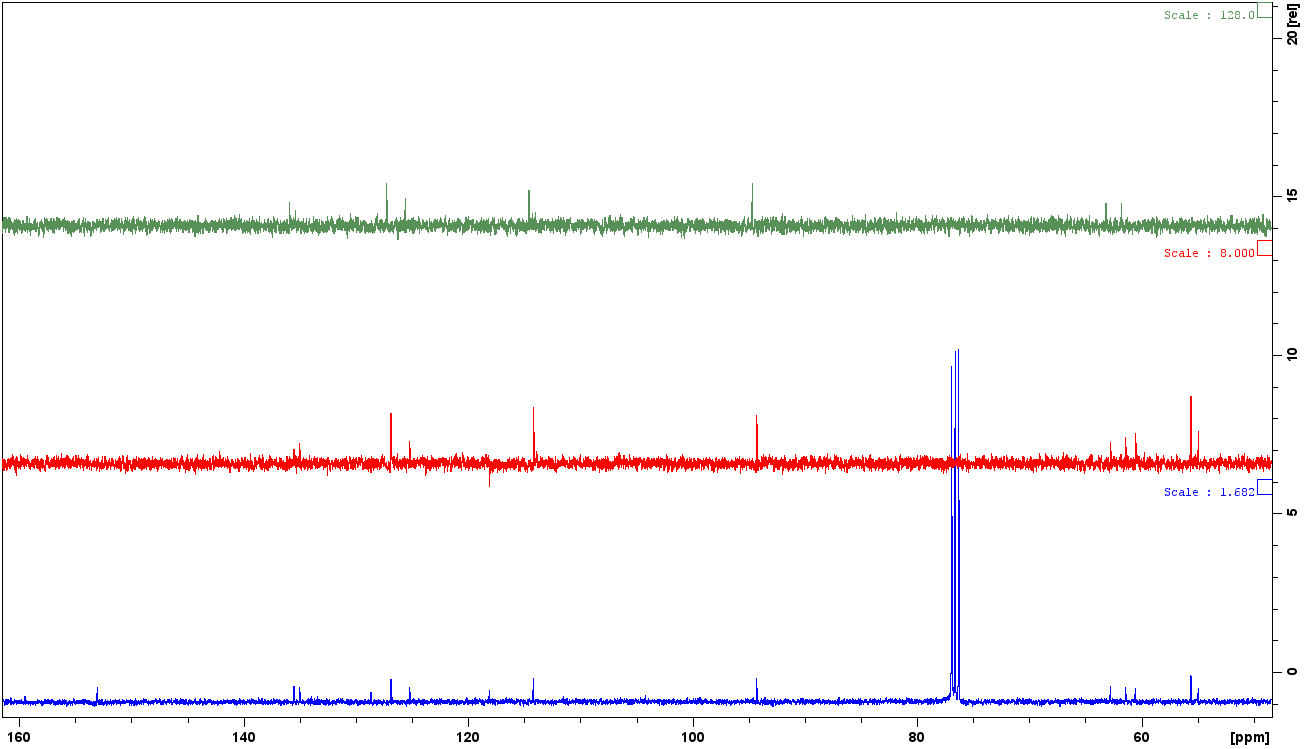

Supplement: Supplementary file 1 [file pharmaceuticals-16-01000-s001.zip › 11h mjm16070_13cDEPTs.png]

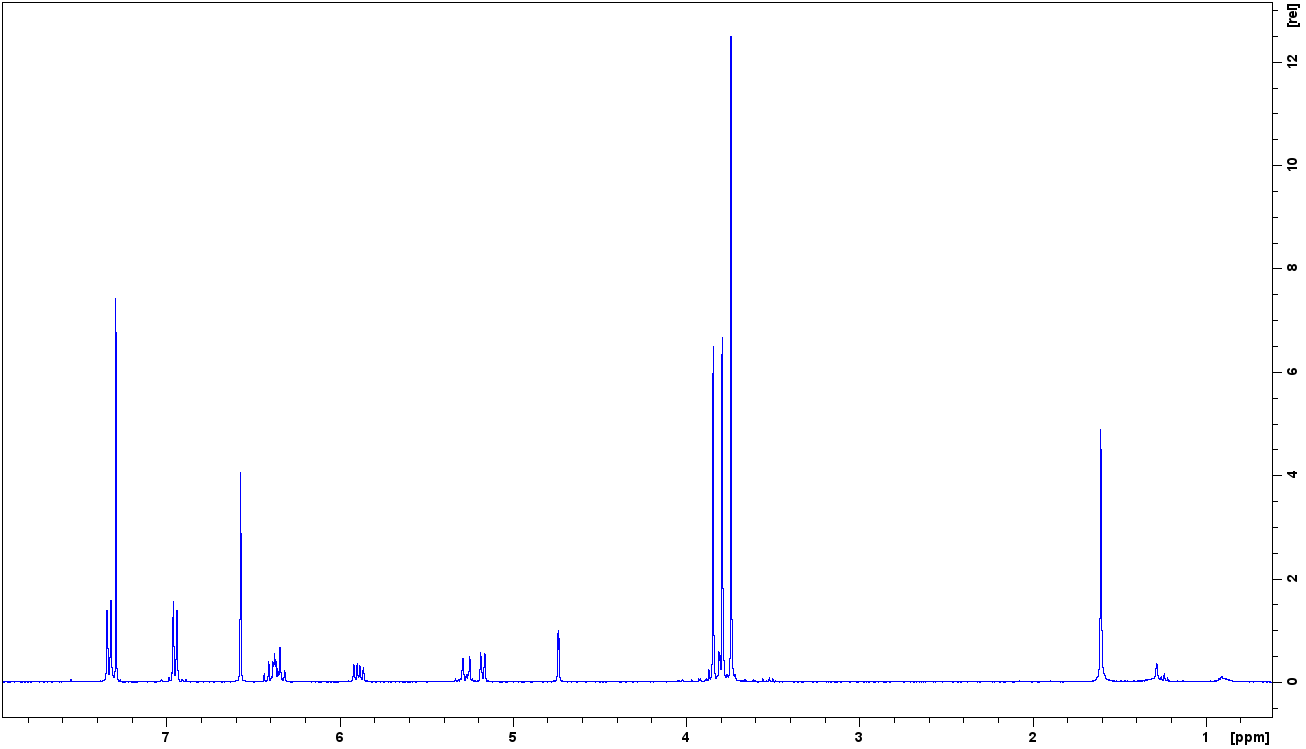

Supplement: Supplementary file 1 [file pharmaceuticals-16-01000-s001.zip › 11h mjm16070_1h.png]

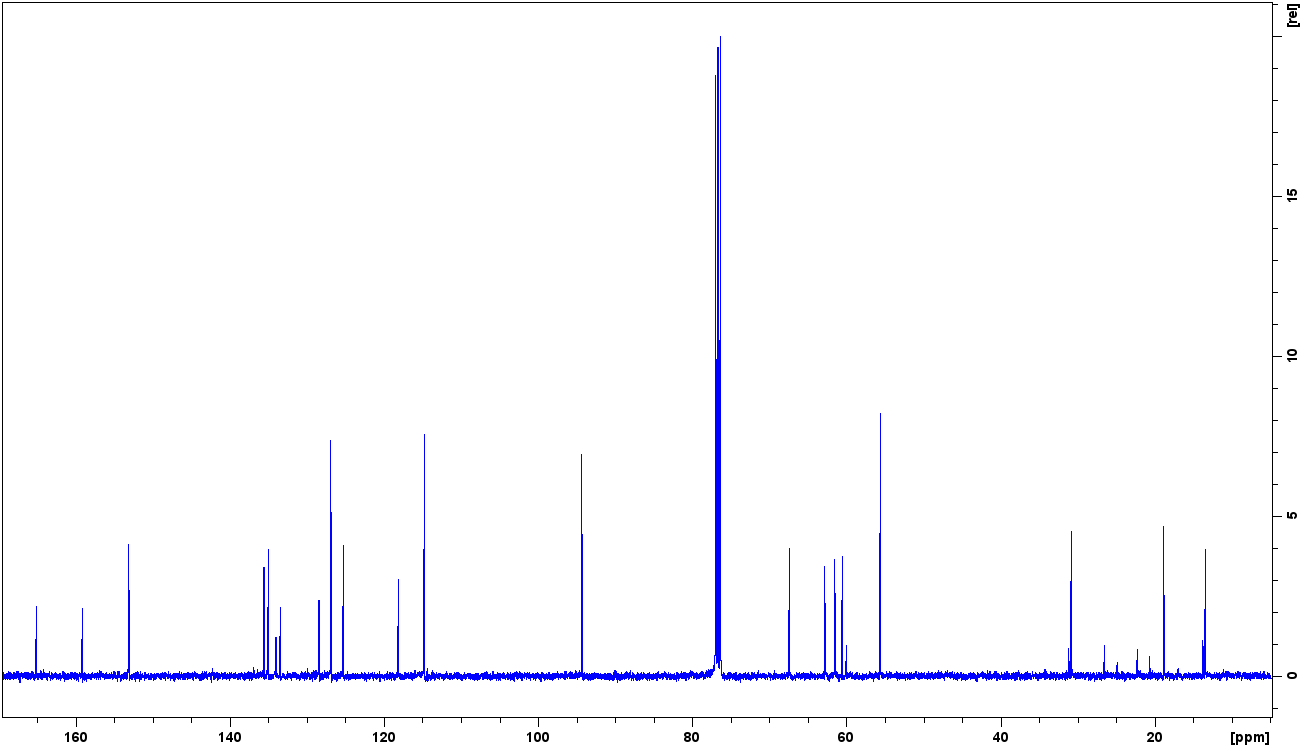

Supplement: Supplementary file 1 [file pharmaceuticals-16-01000-s001.zip › 11j mjm16177_13c.png]

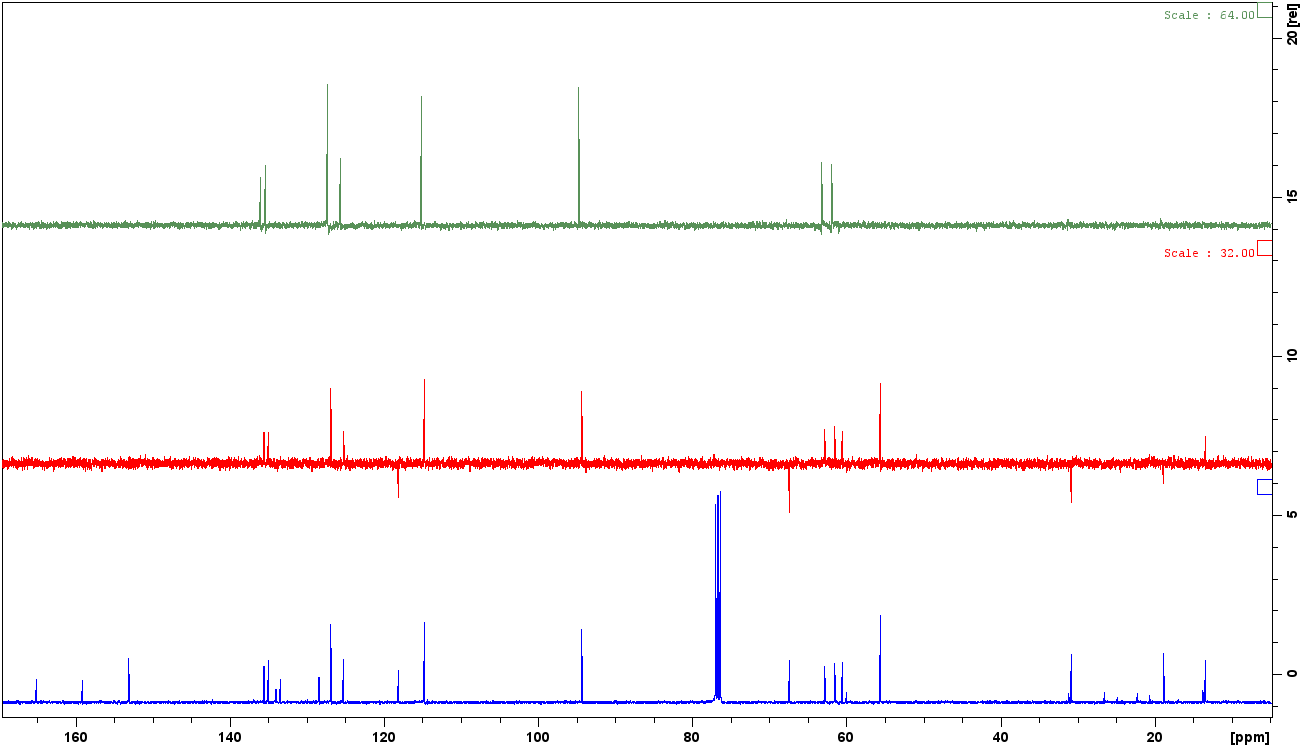

Supplement: Supplementary file 1 [file pharmaceuticals-16-01000-s001.zip › 11j mjm16177_13c_DEPTs.png]

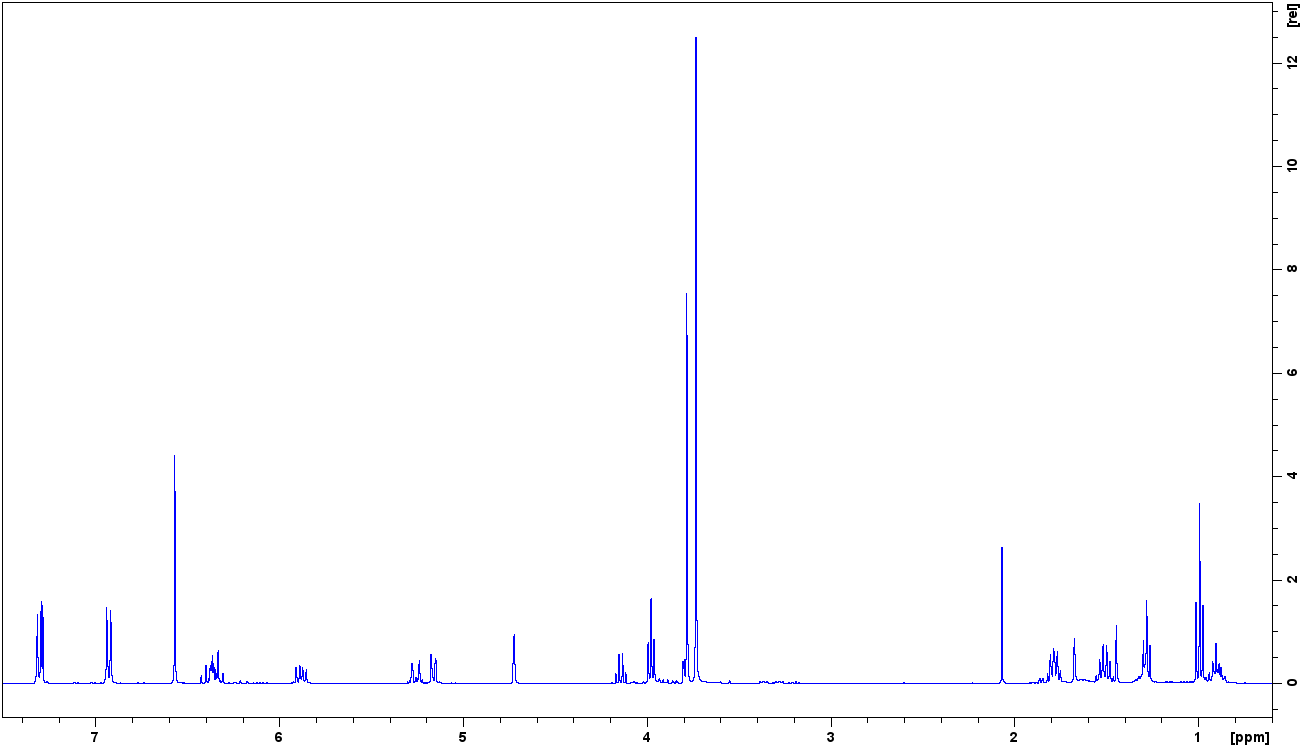

Supplement: Supplementary file 1 [file pharmaceuticals-16-01000-s001.zip › 11j mjm16177_1h.png]

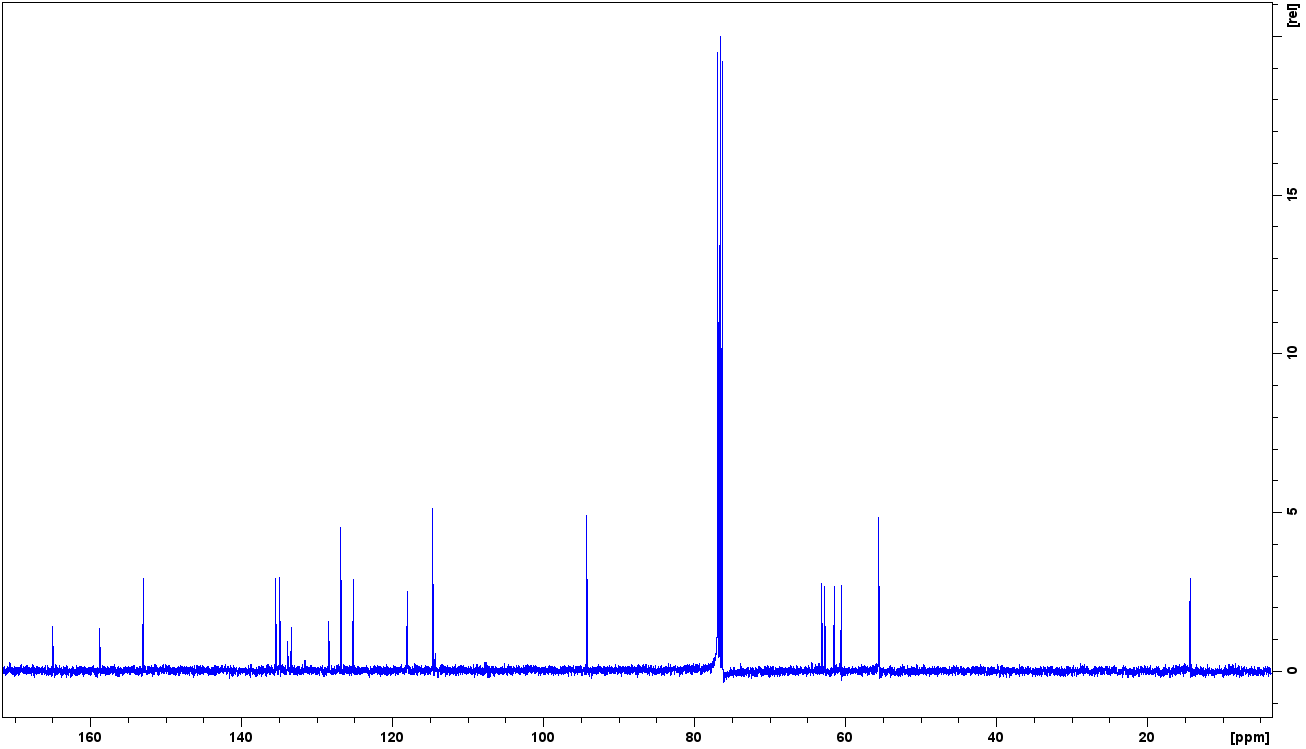

Supplement: Supplementary file 1 [file pharmaceuticals-16-01000-s001.zip › 11j mjm16230_13c.png]

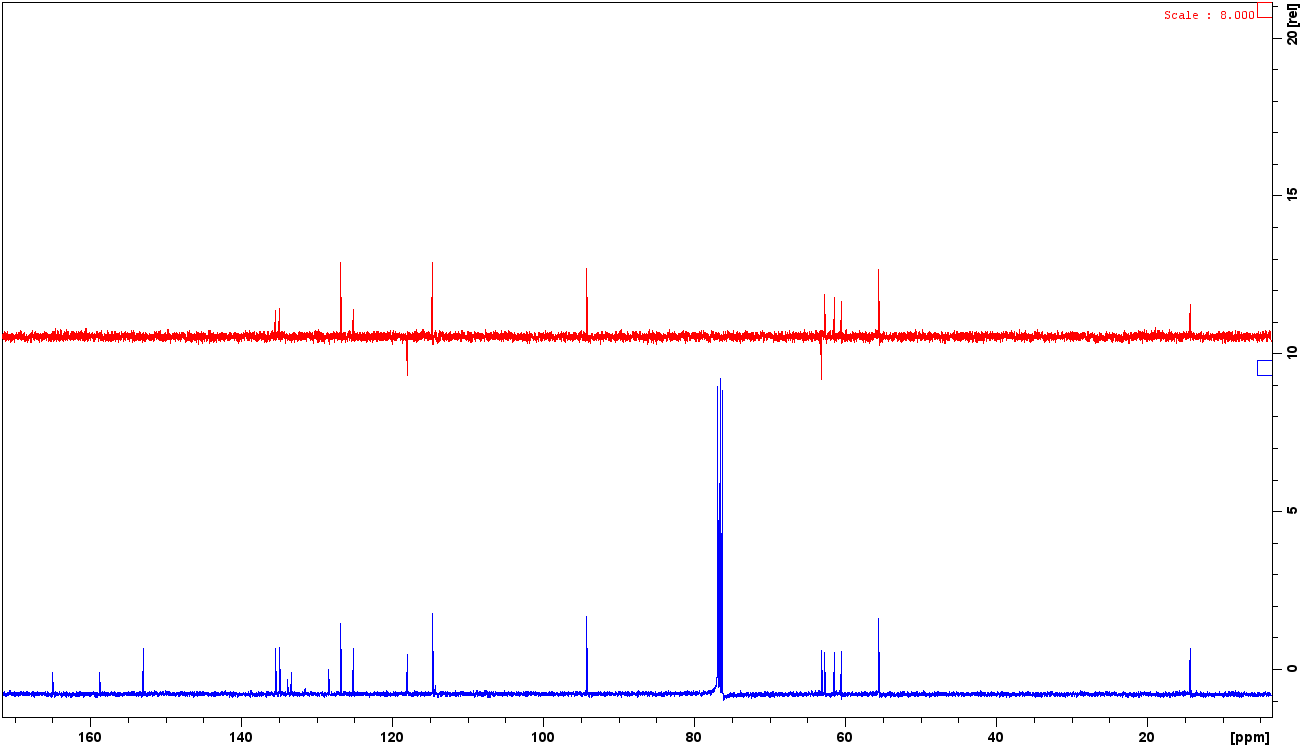

Supplement: Supplementary file 1 [file pharmaceuticals-16-01000-s001.zip › 11j mjm16230_13c_DEPT.png]

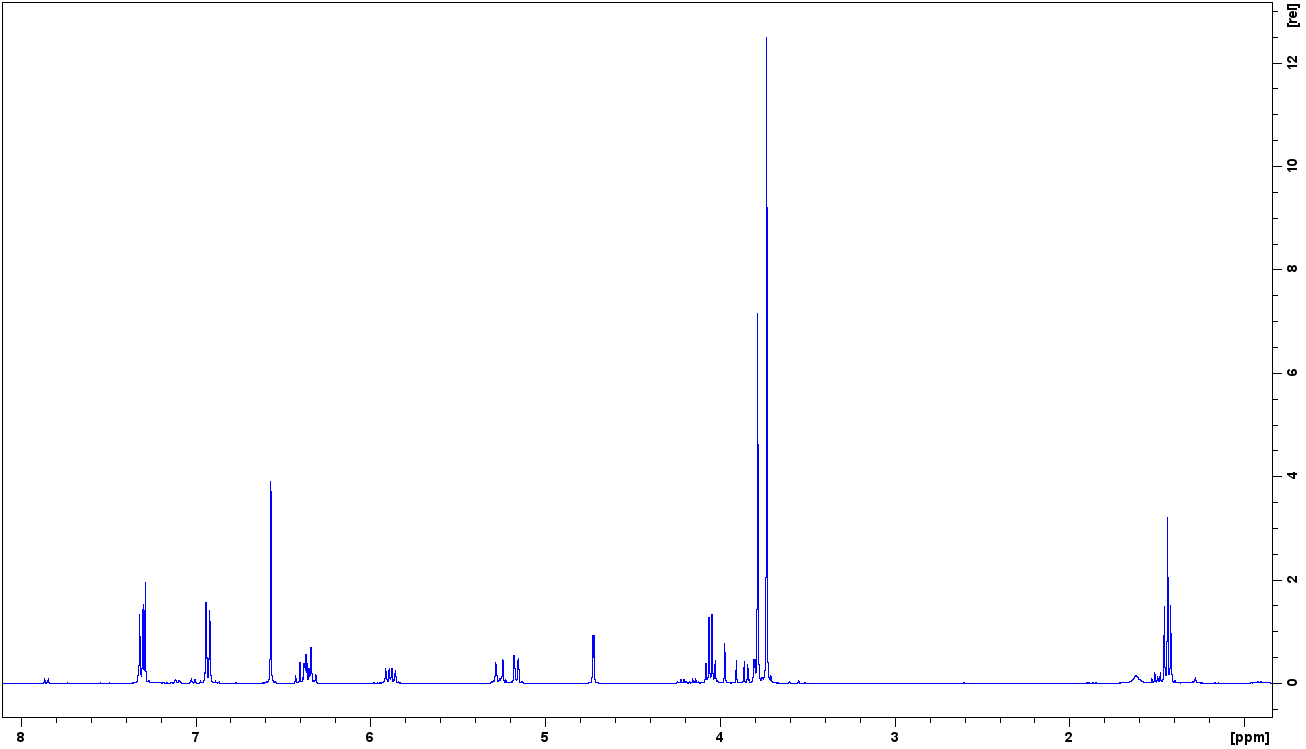

Supplement: Supplementary file 1 [file pharmaceuticals-16-01000-s001.zip › 11j mjm16230_1h.png]

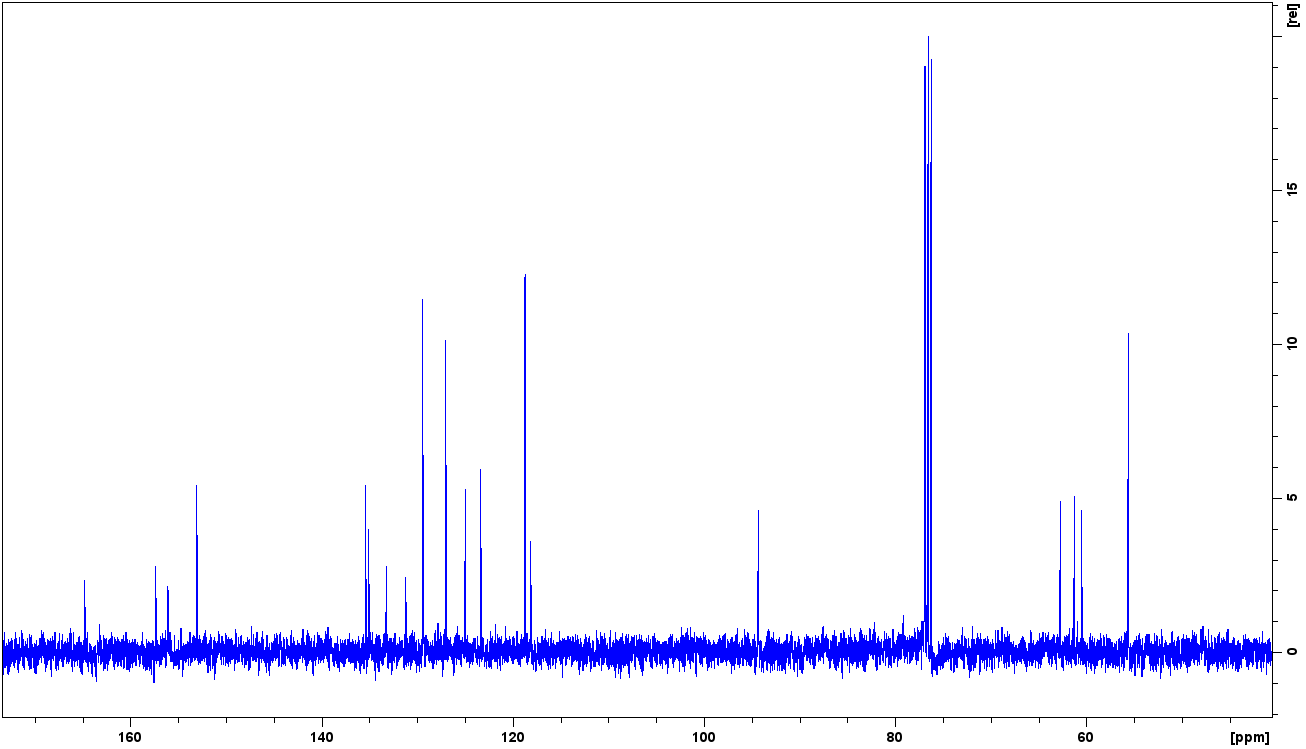

Supplement: Supplementary file 1 [file pharmaceuticals-16-01000-s001.zip › 11k mjm16400_13c.png]

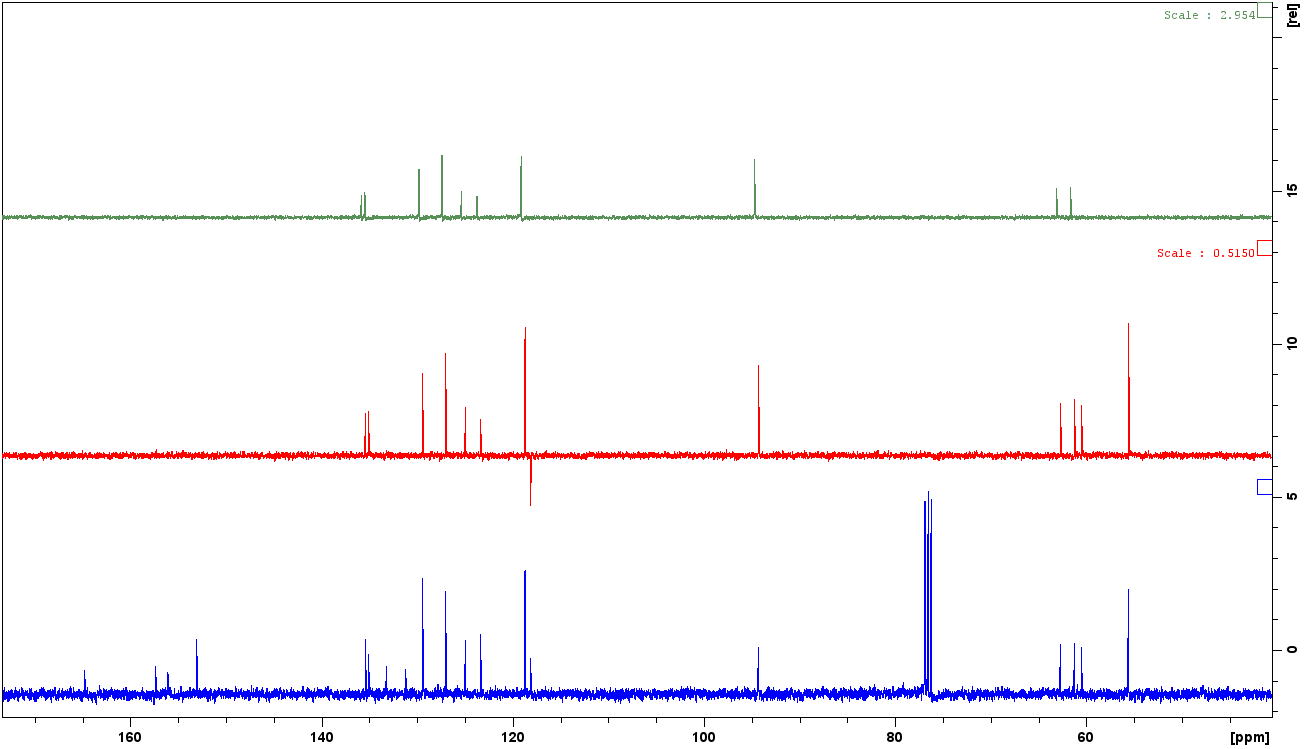

Supplement: Supplementary file 1 [file pharmaceuticals-16-01000-s001.zip › 11k mjm16400_13c_DEPTs.png]

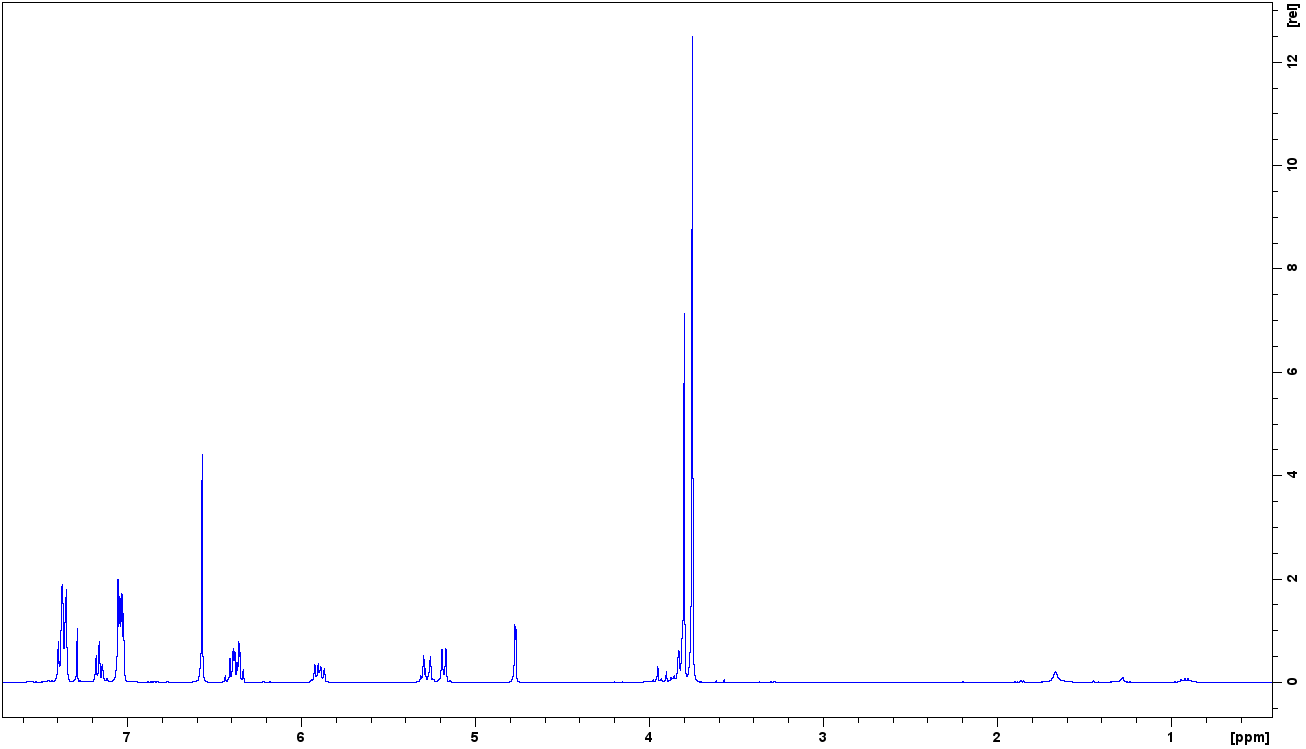

Supplement: Supplementary file 1 [file pharmaceuticals-16-01000-s001.zip › 11k mjm16400_1h.png]

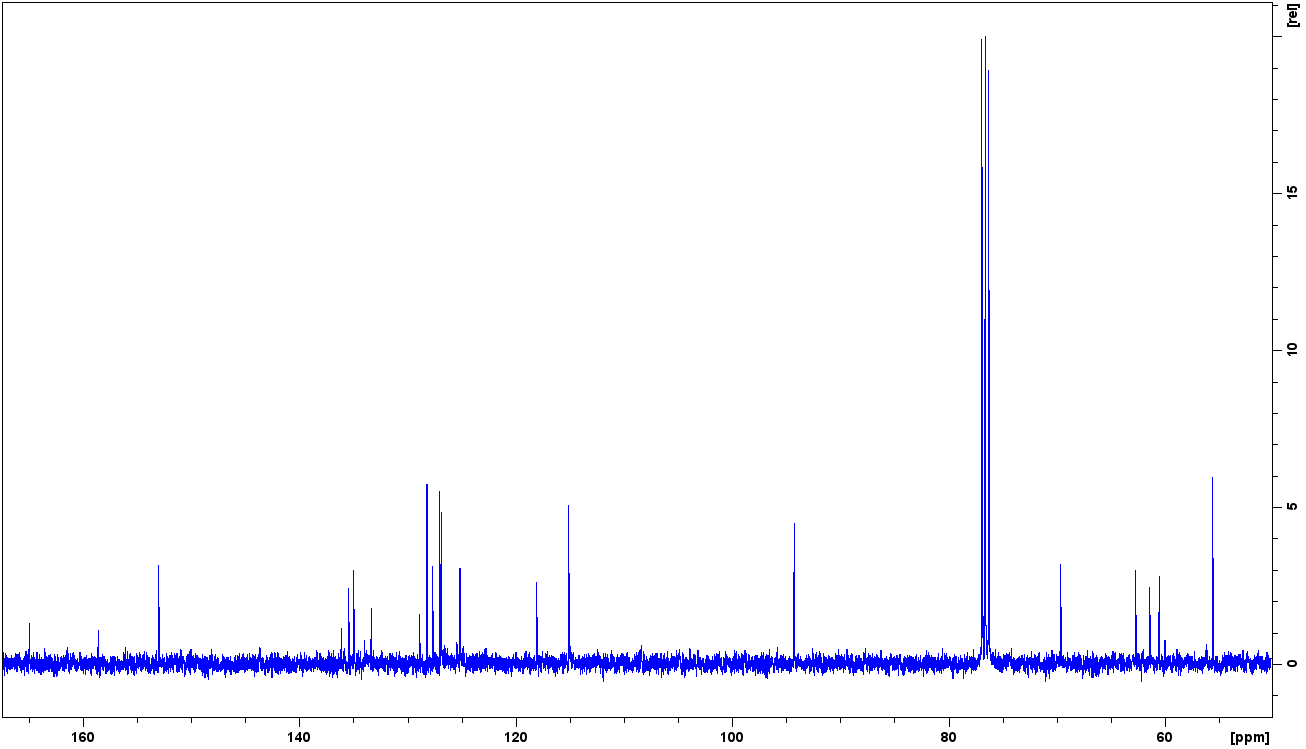

Supplement: Supplementary file 1 [file pharmaceuticals-16-01000-s001.zip › 11l mjm16187_13c.png]

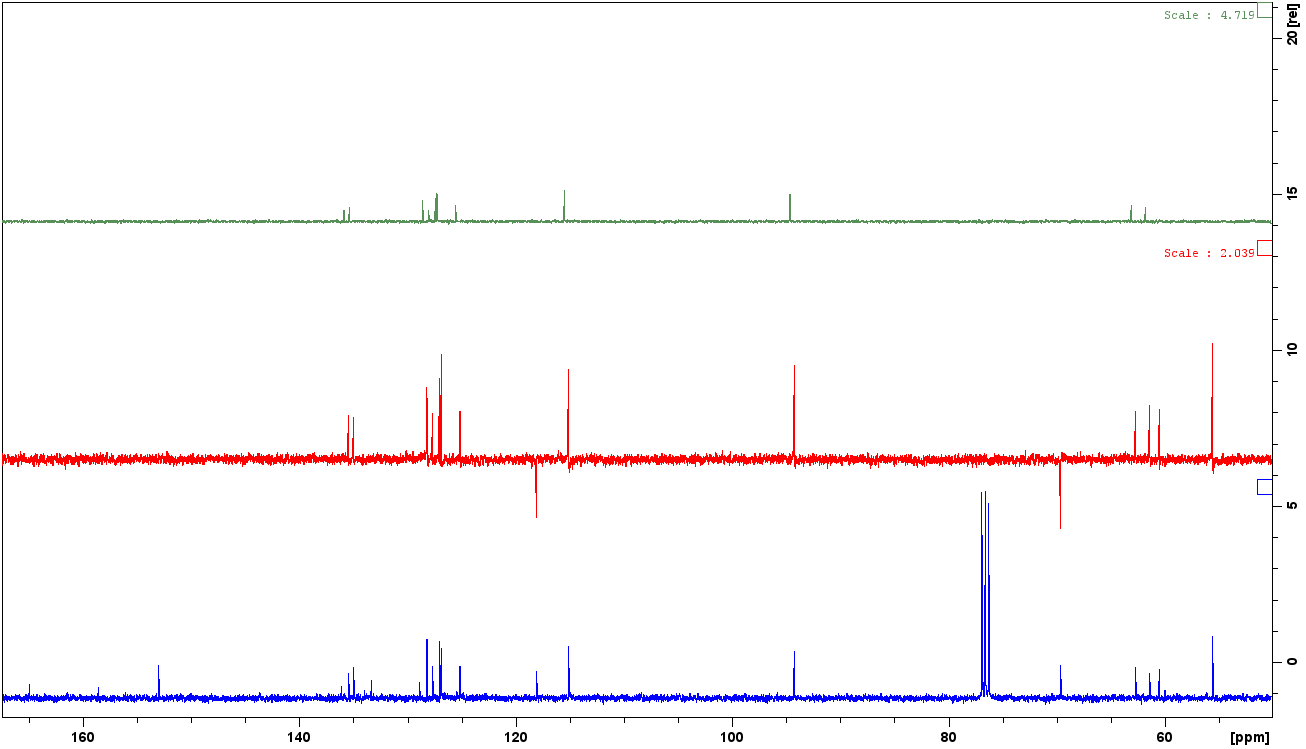

Supplement: Supplementary file 1 [file pharmaceuticals-16-01000-s001.zip › 11l mjm16187_13c_depts.png]

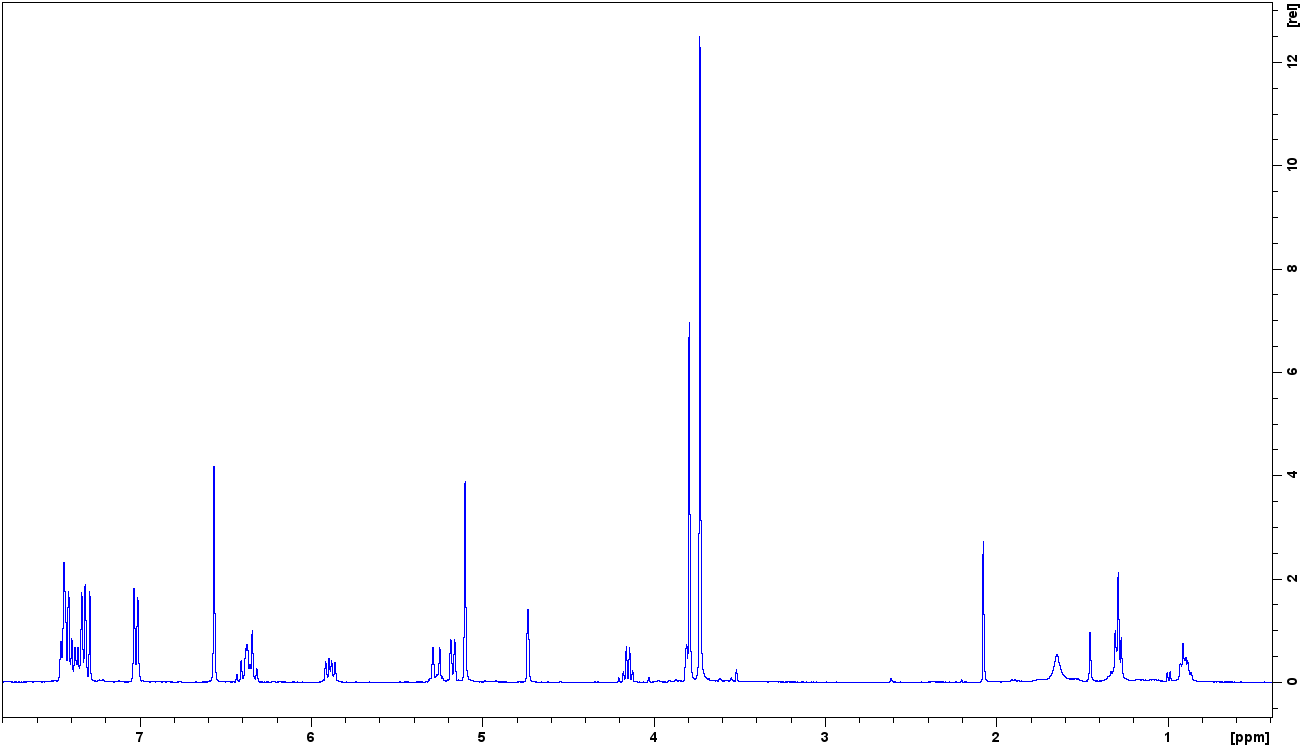

Supplement: Supplementary file 1 [file pharmaceuticals-16-01000-s001.zip › 11l mjm16187_1h.png]

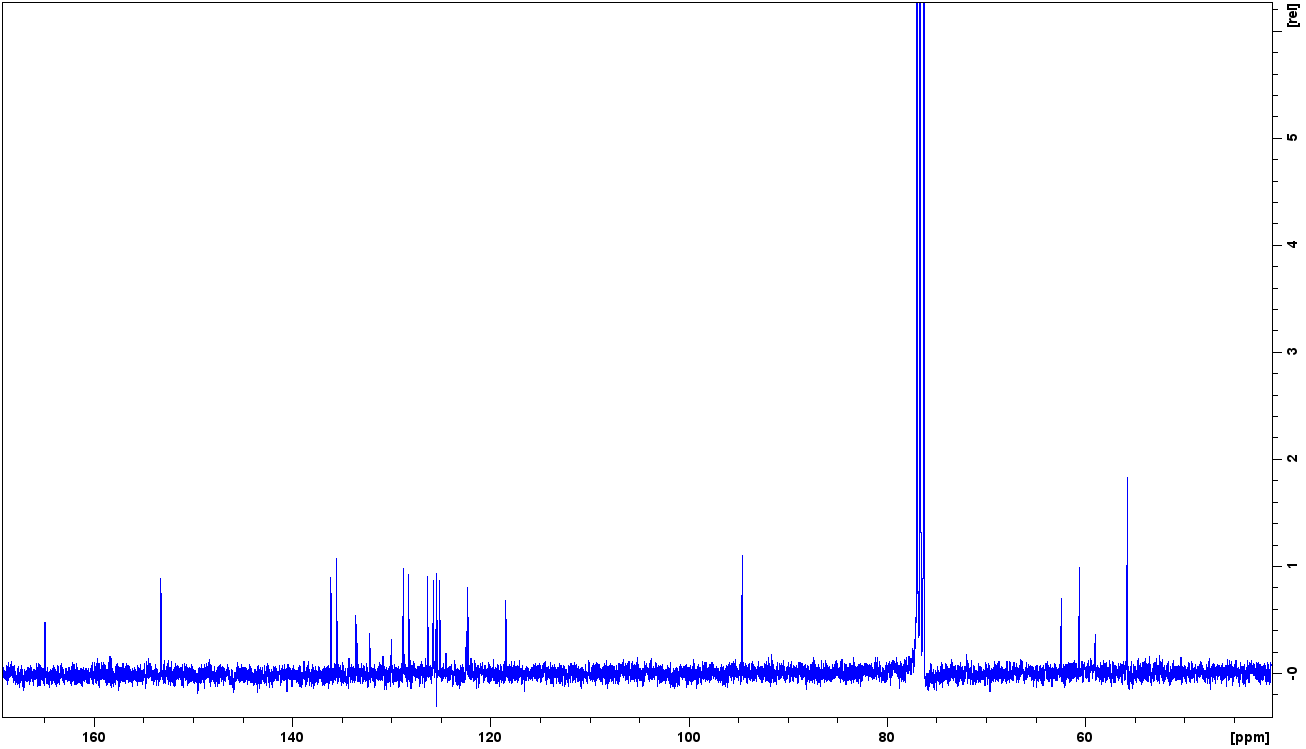

Supplement: Supplementary file 1 [file pharmaceuticals-16-01000-s001.zip › 11m mjm16439_13c.png]

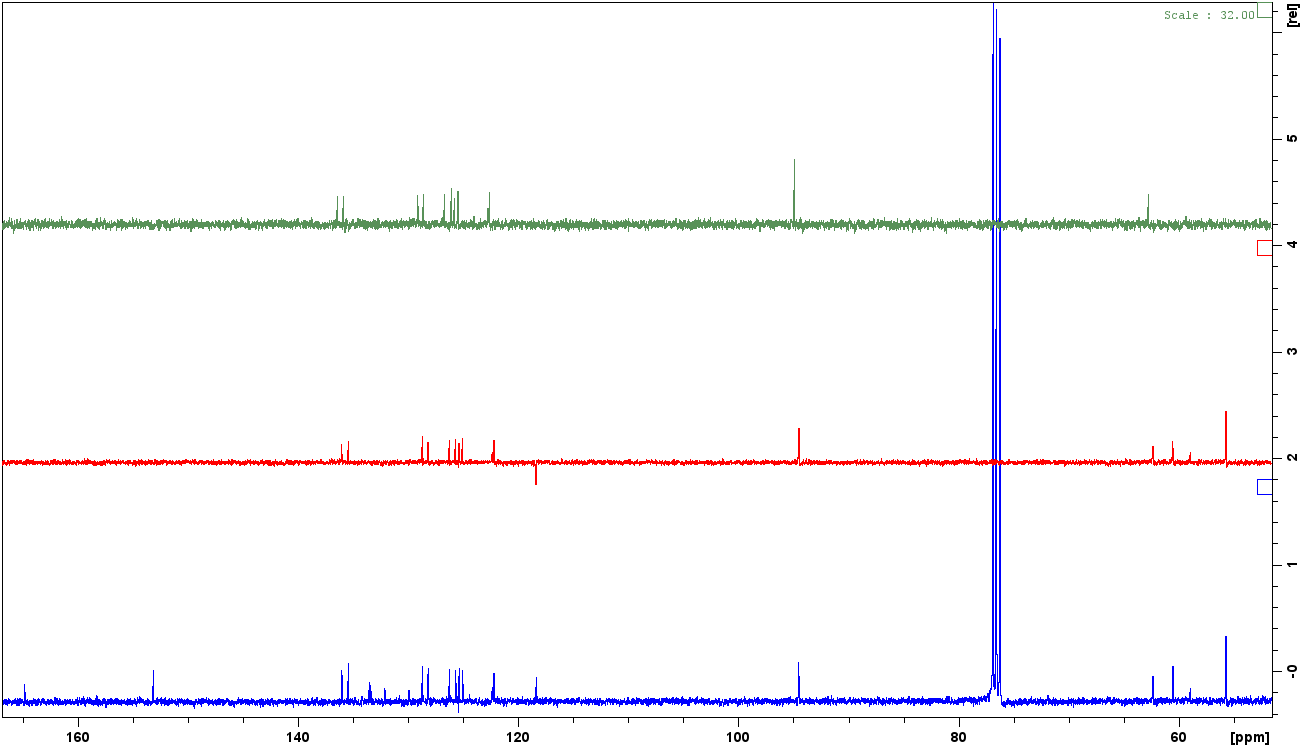

Supplement: Supplementary file 1 [file pharmaceuticals-16-01000-s001.zip › 11m mjm16439_13c_DEPTs.png]

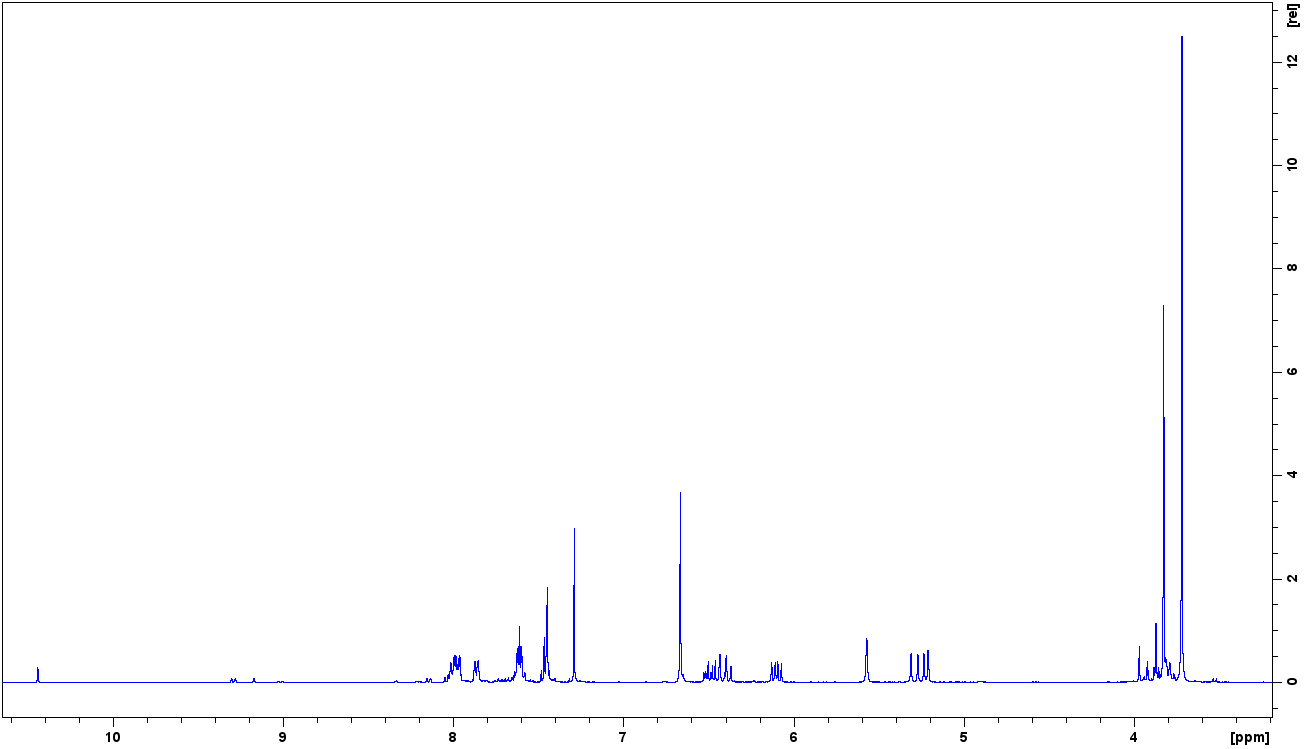

Supplement: Supplementary file 1 [file pharmaceuticals-16-01000-s001.zip › 11m mjm16439_1h.png]

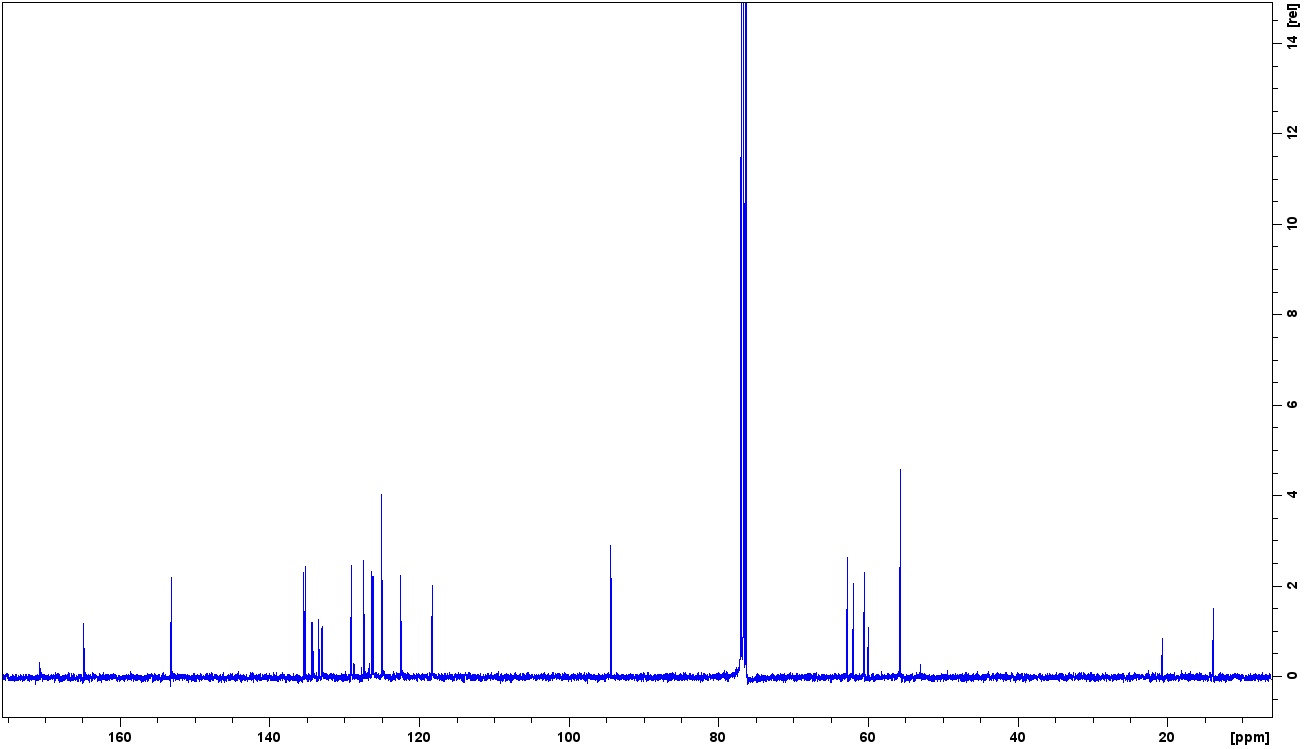

Supplement: Supplementary file 1 [file pharmaceuticals-16-01000-s001.zip › 11n mjm16483_13c.png]

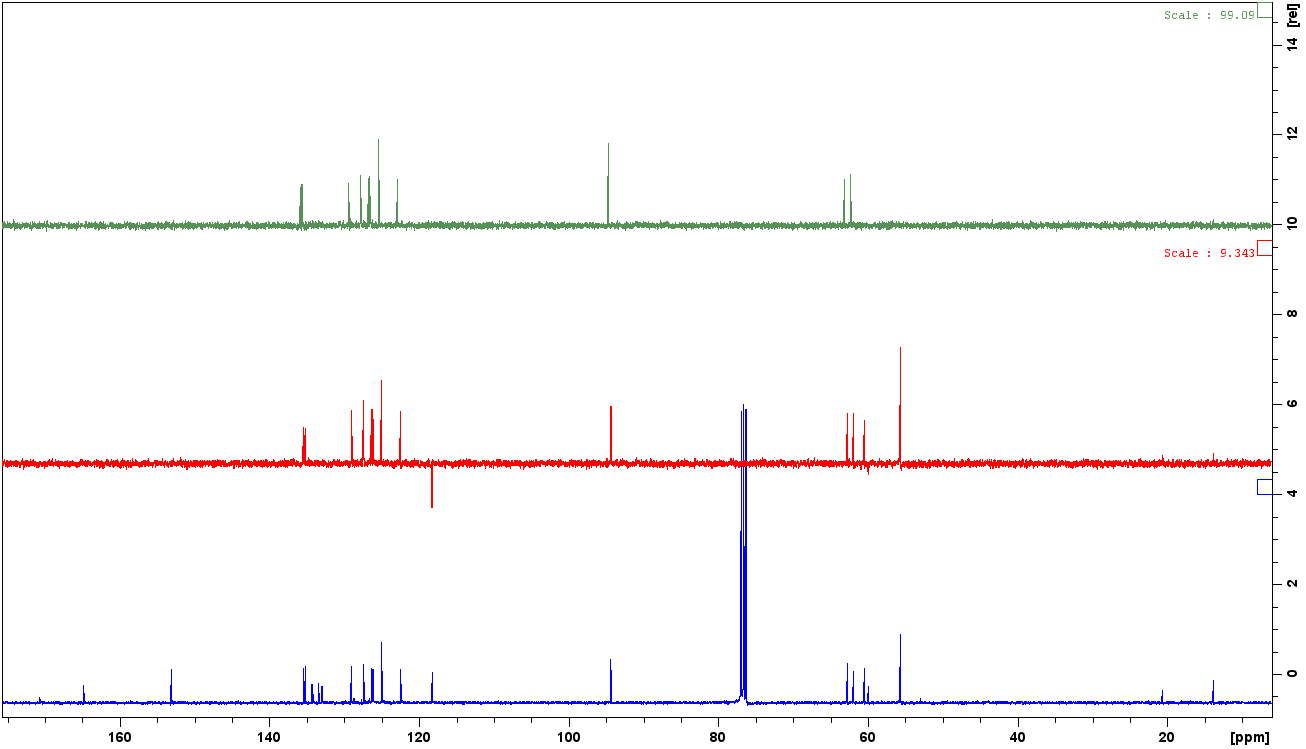

Supplement: Supplementary file 1 [file pharmaceuticals-16-01000-s001.zip › 11n mjm16483_13c_DEPTs.png]

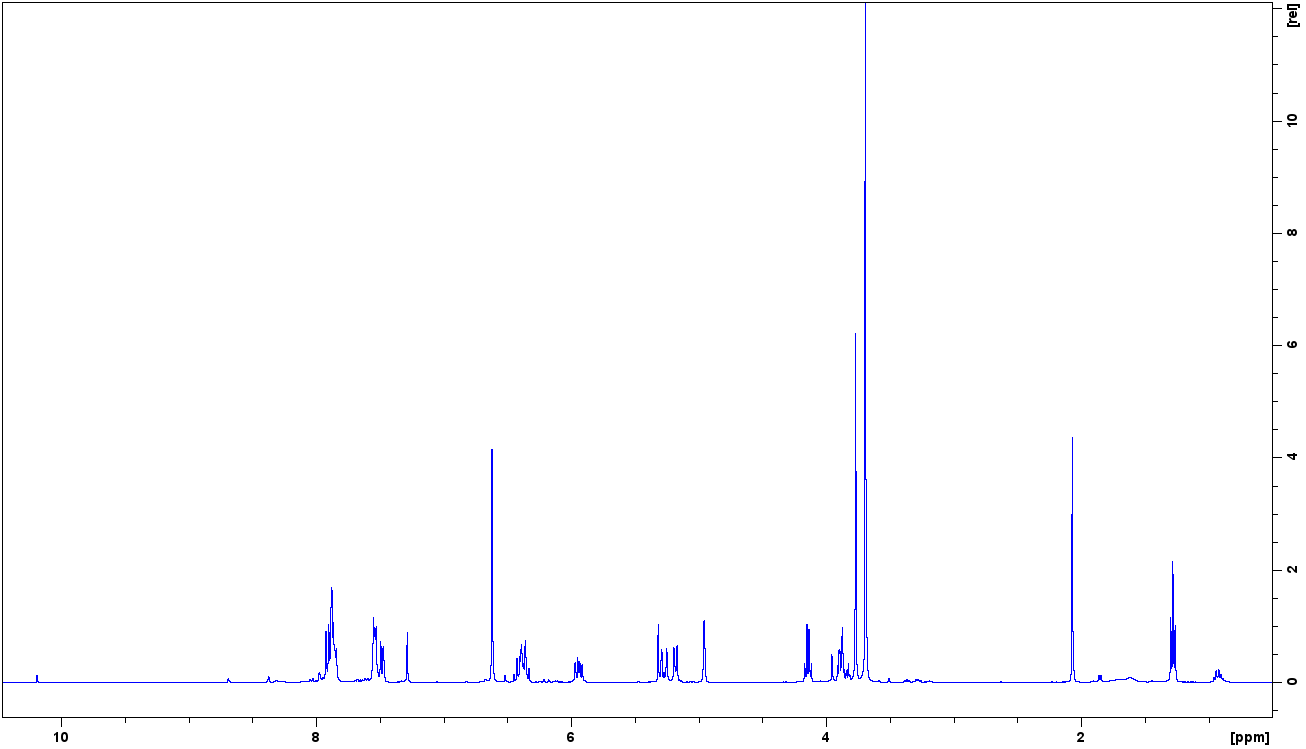

Supplement: Supplementary file 1 [file pharmaceuticals-16-01000-s001.zip › 11n mjm16483_1h.png]

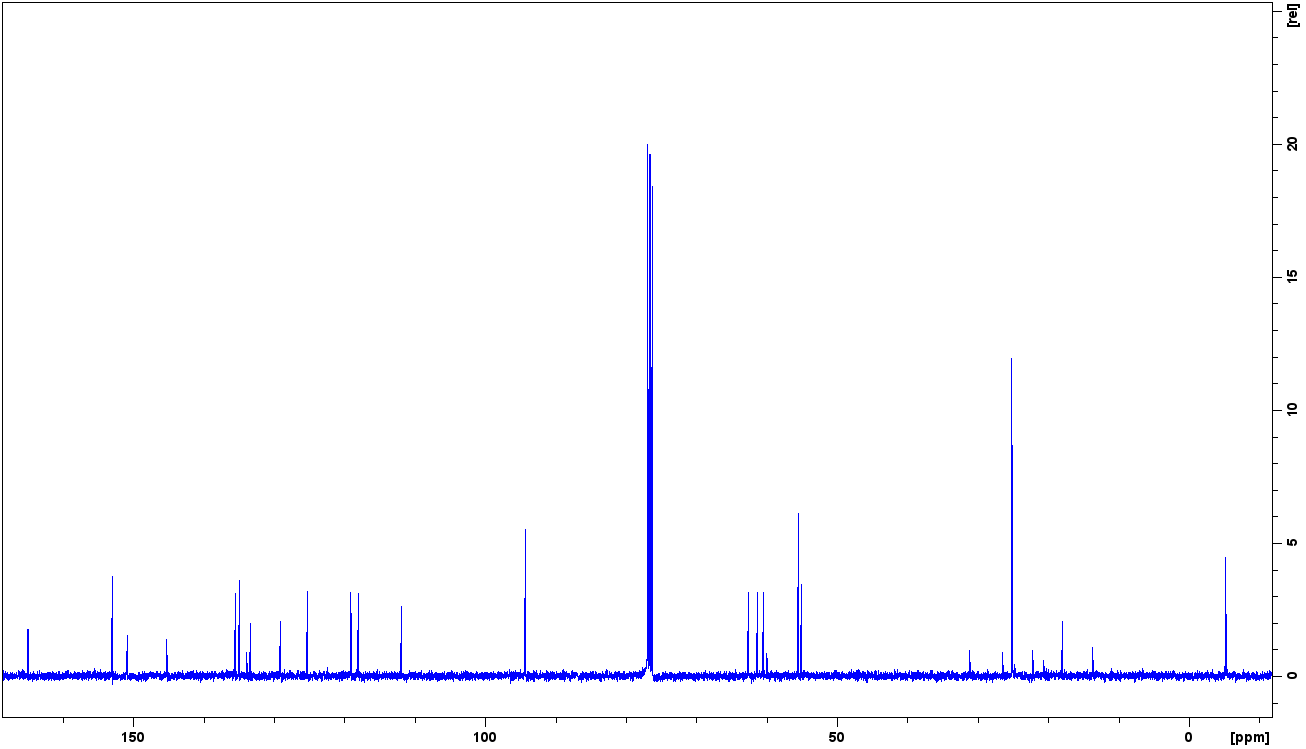

Supplement: Supplementary file 1 [file pharmaceuticals-16-01000-s001.zip › 11p mjm16518_13c.png]

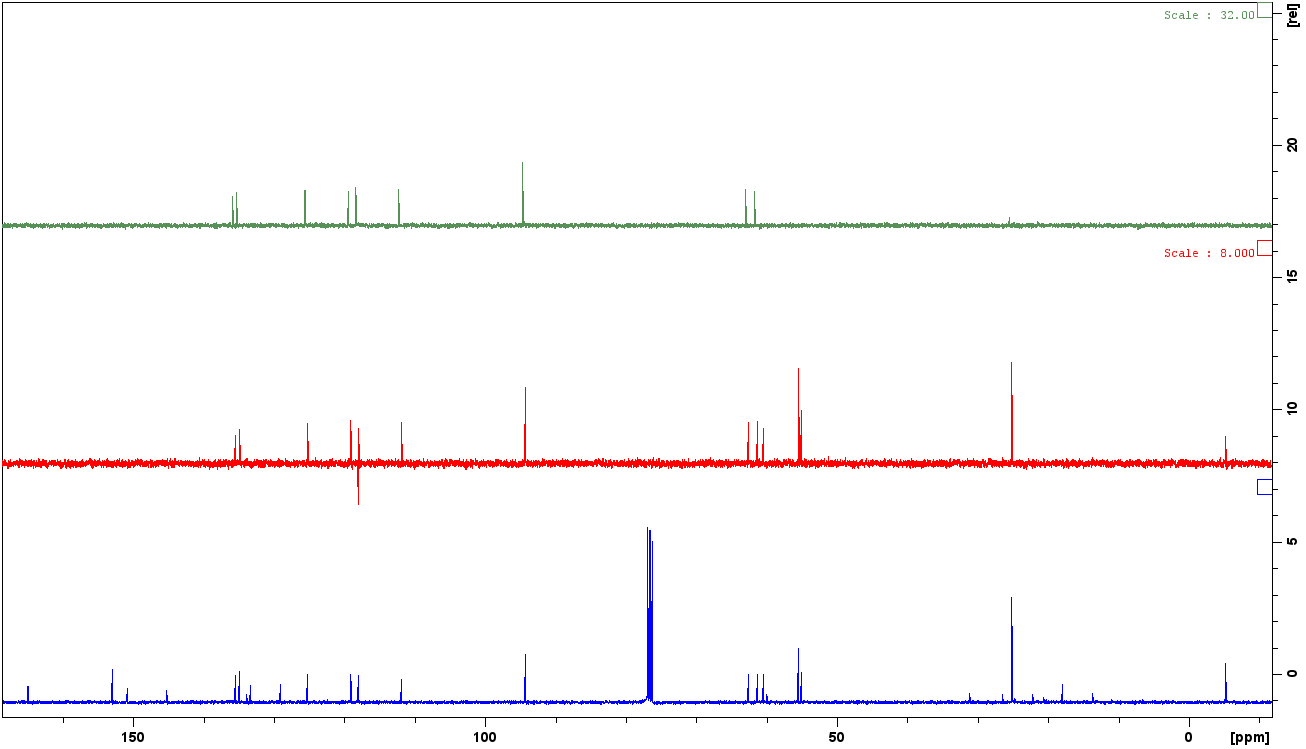

Supplement: Supplementary file 1 [file pharmaceuticals-16-01000-s001.zip › 11p mjm16518_13c_DEPTs.png]

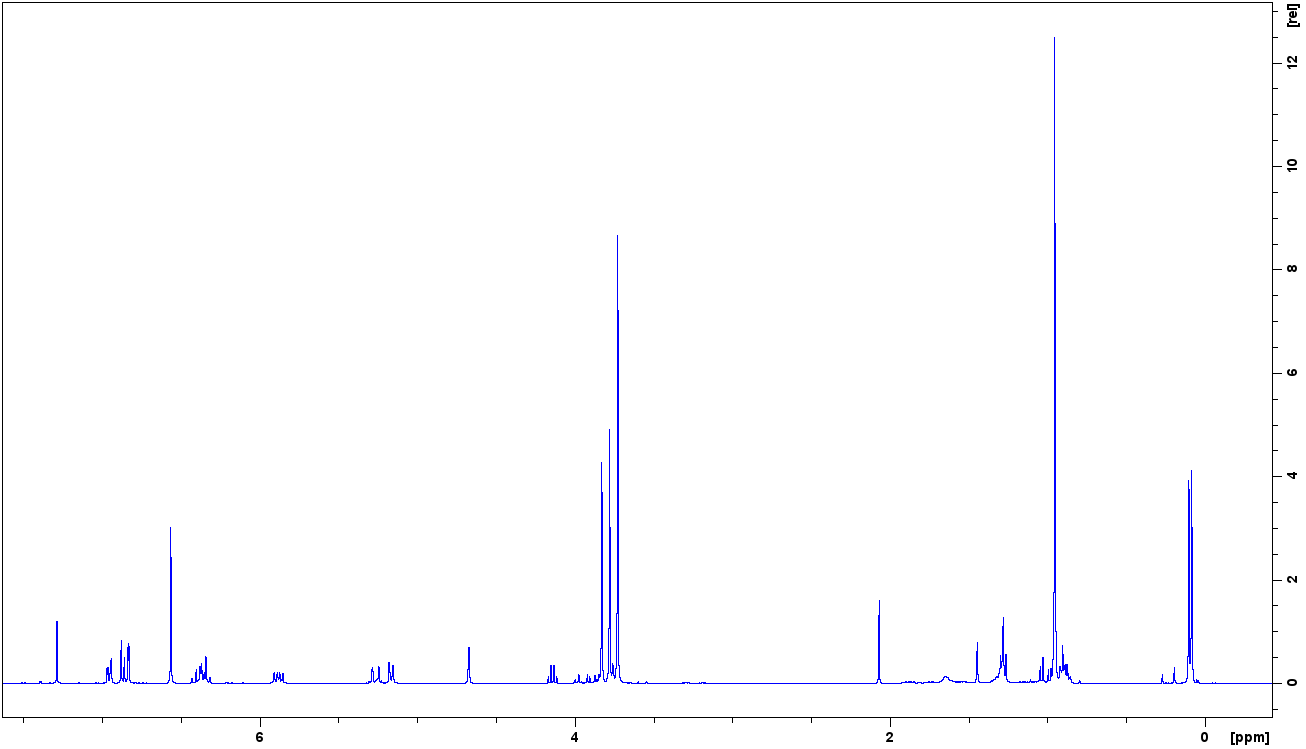

Supplement: Supplementary file 1 [file pharmaceuticals-16-01000-s001.zip › 11p mjm16518_1h.png]

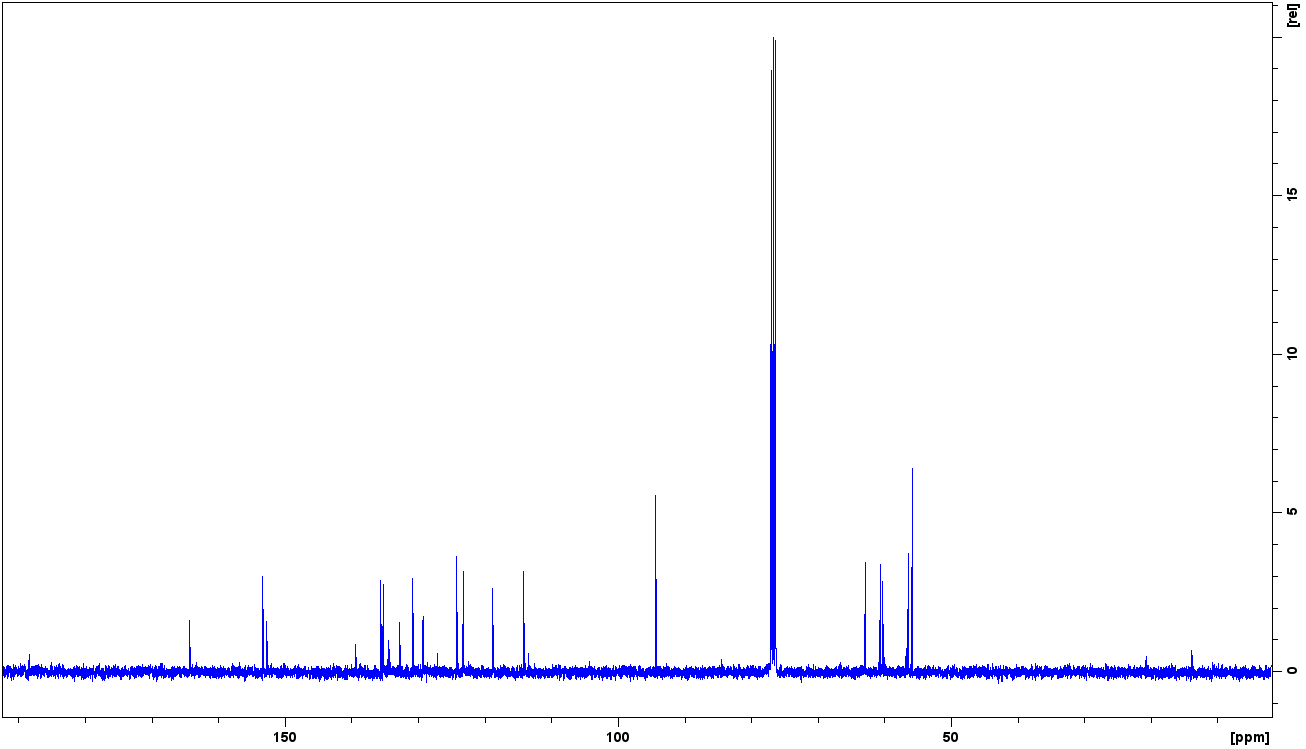

Supplement: Supplementary file 1 [file pharmaceuticals-16-01000-s001.zip › 11q mjm17078_13c.png]

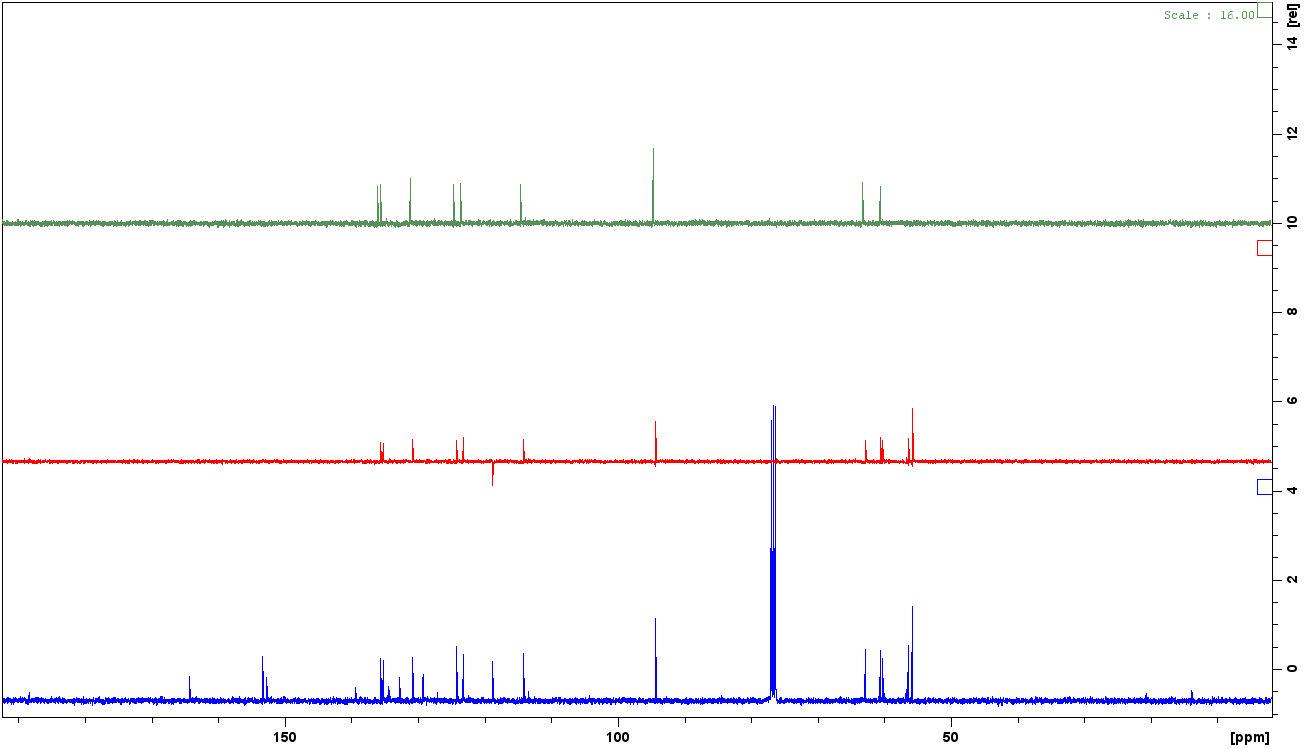

Supplement: Supplementary file 1 [file pharmaceuticals-16-01000-s001.zip › 11q mjm17078_13c_DEPTs.png]

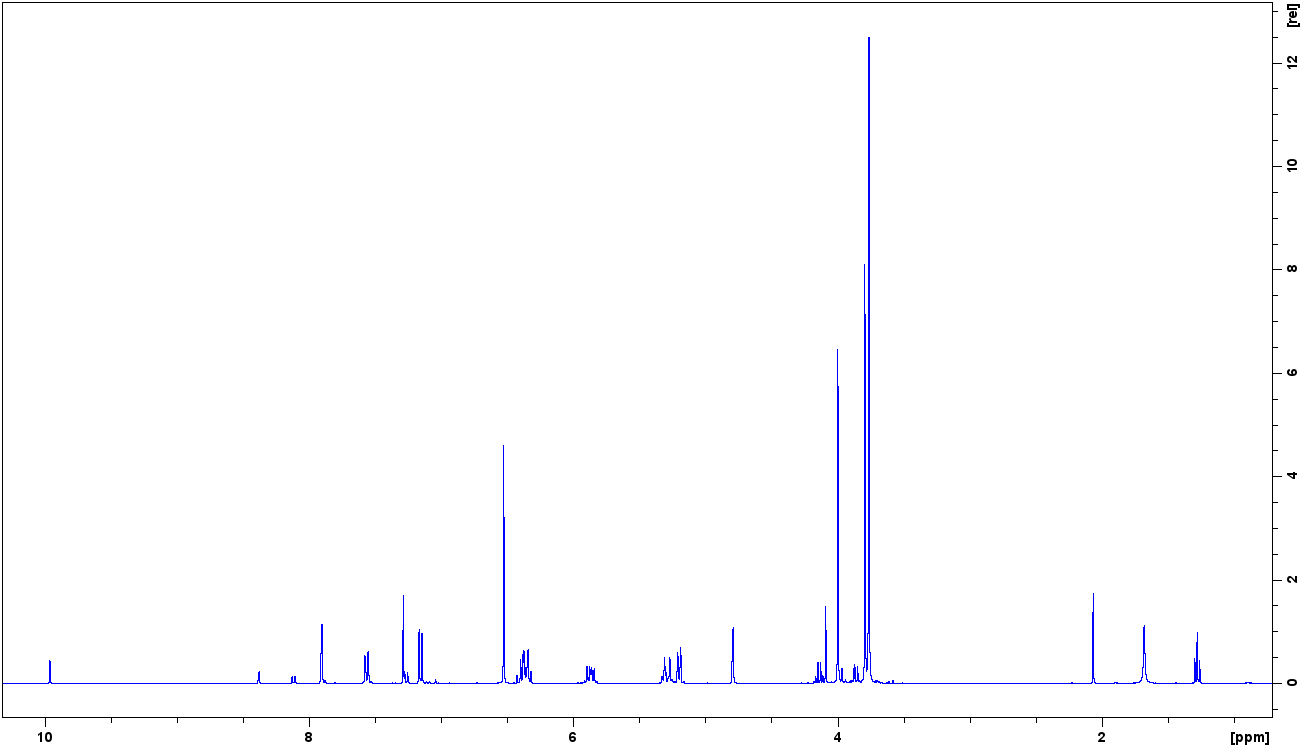

Supplement: Supplementary file 1 [file pharmaceuticals-16-01000-s001.zip › 11q mjm17078_1h.png]

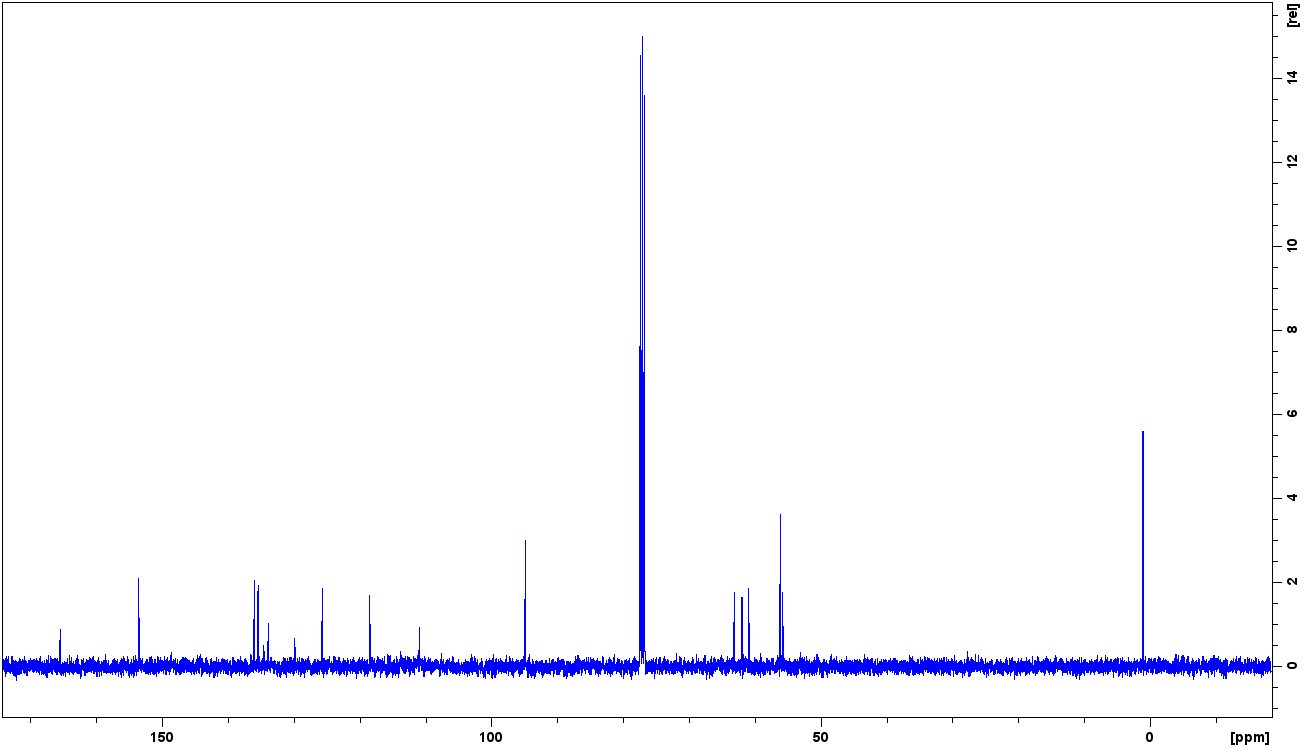

Supplement: Supplementary file 1 [file pharmaceuticals-16-01000-s001.zip › 11r mjm17118_13c.png]

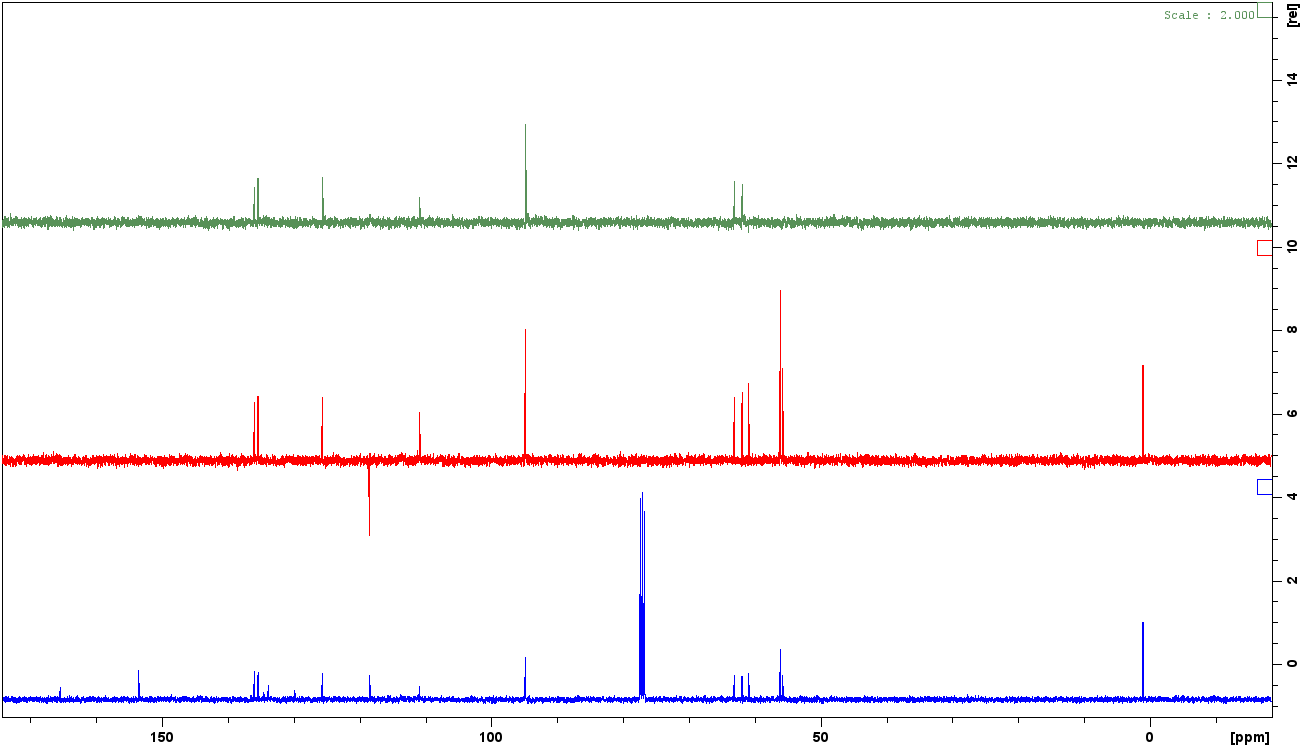

Supplement: Supplementary file 1 [file pharmaceuticals-16-01000-s001.zip › 11r mjm17118_13c_DEPTs.png]

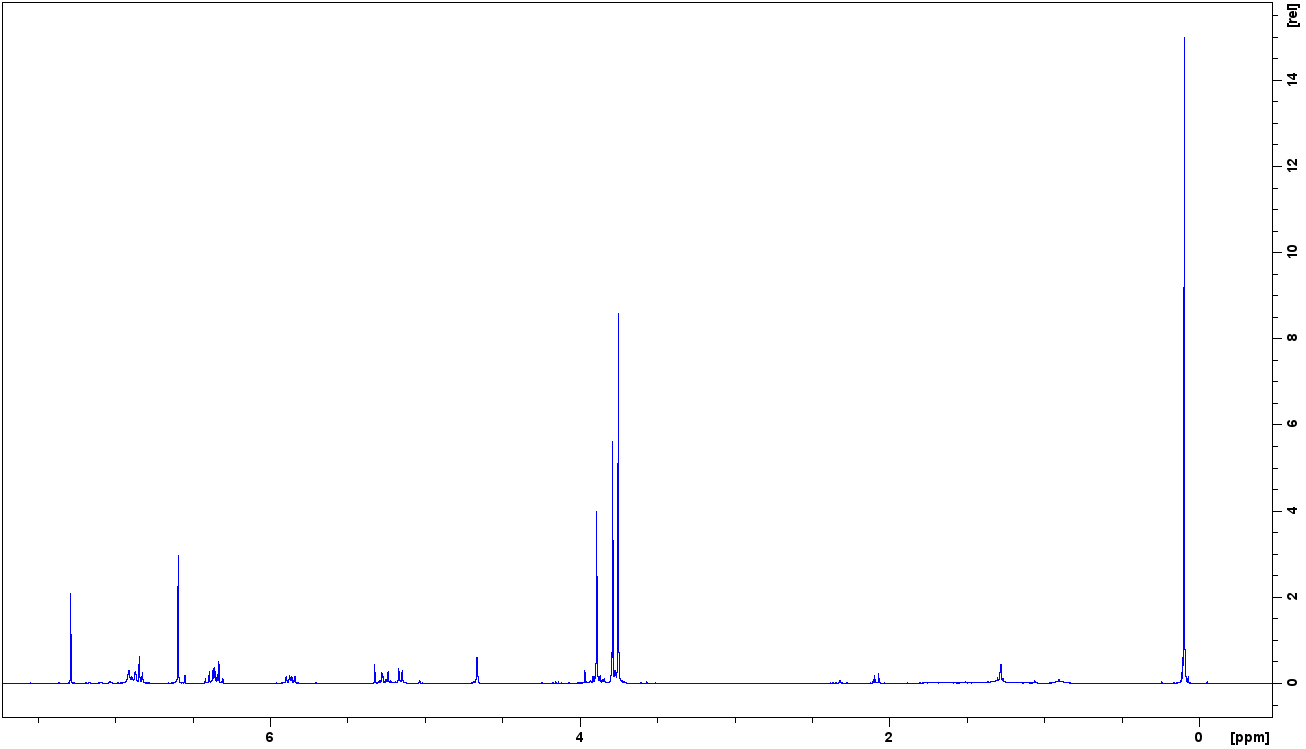

Supplement: Supplementary file 1 [file pharmaceuticals-16-01000-s001.zip › 11r mjm17118_1h.png]

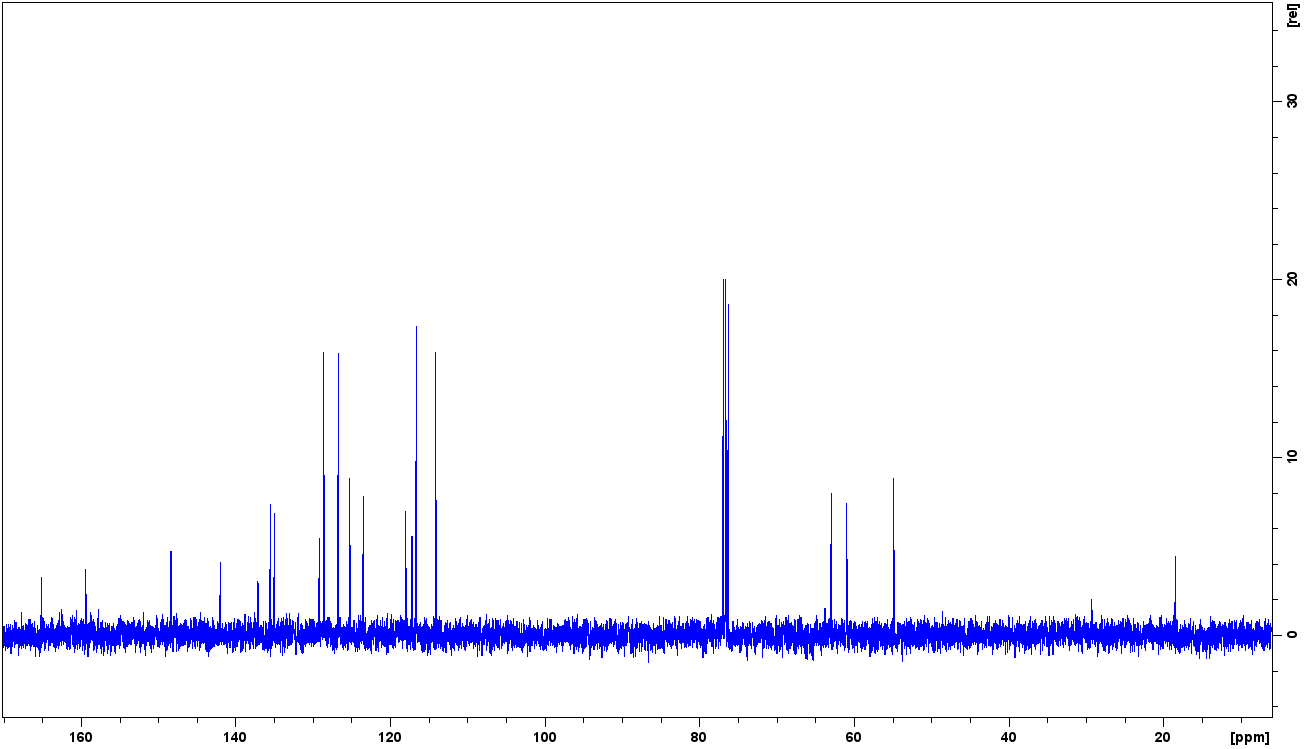

Supplement: Supplementary file 1 [file pharmaceuticals-16-01000-s001.zip › 11s mjm16440_13c.png]

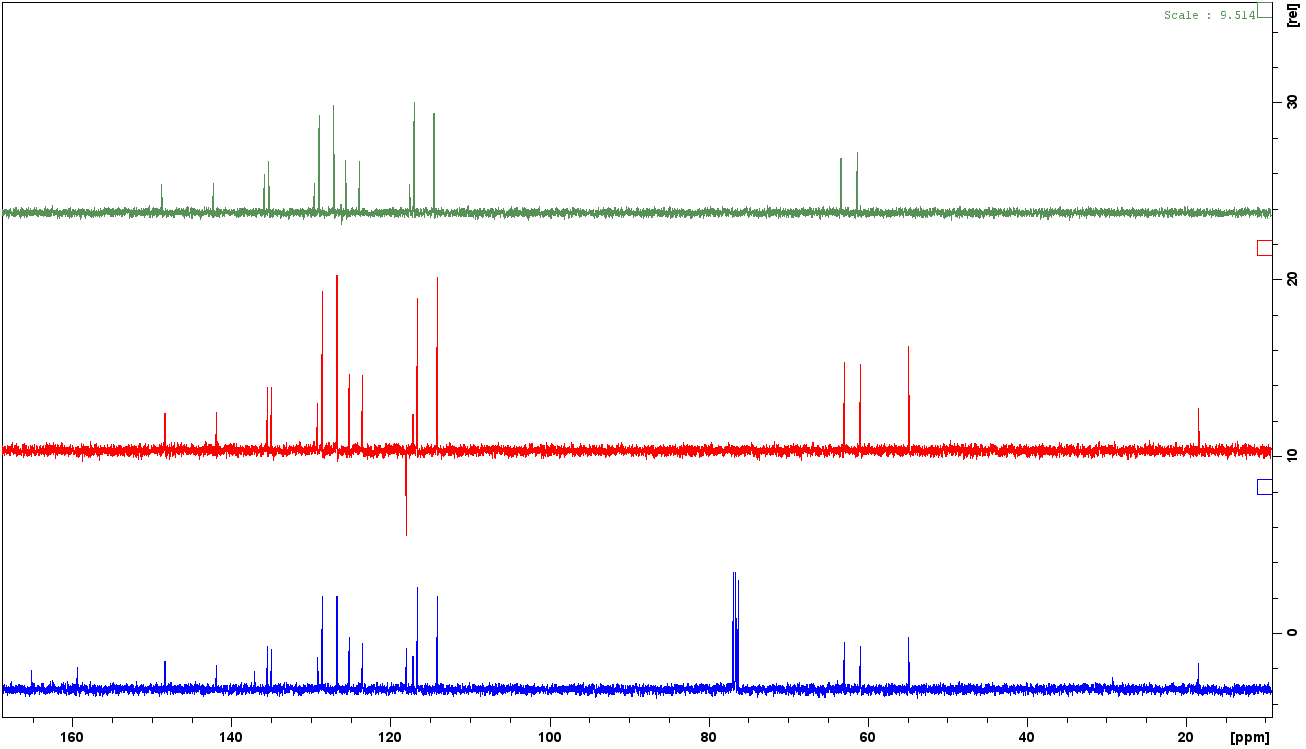

Supplement: Supplementary file 1 [file pharmaceuticals-16-01000-s001.zip › 11s mjm16440_13c_DEPTs.png]

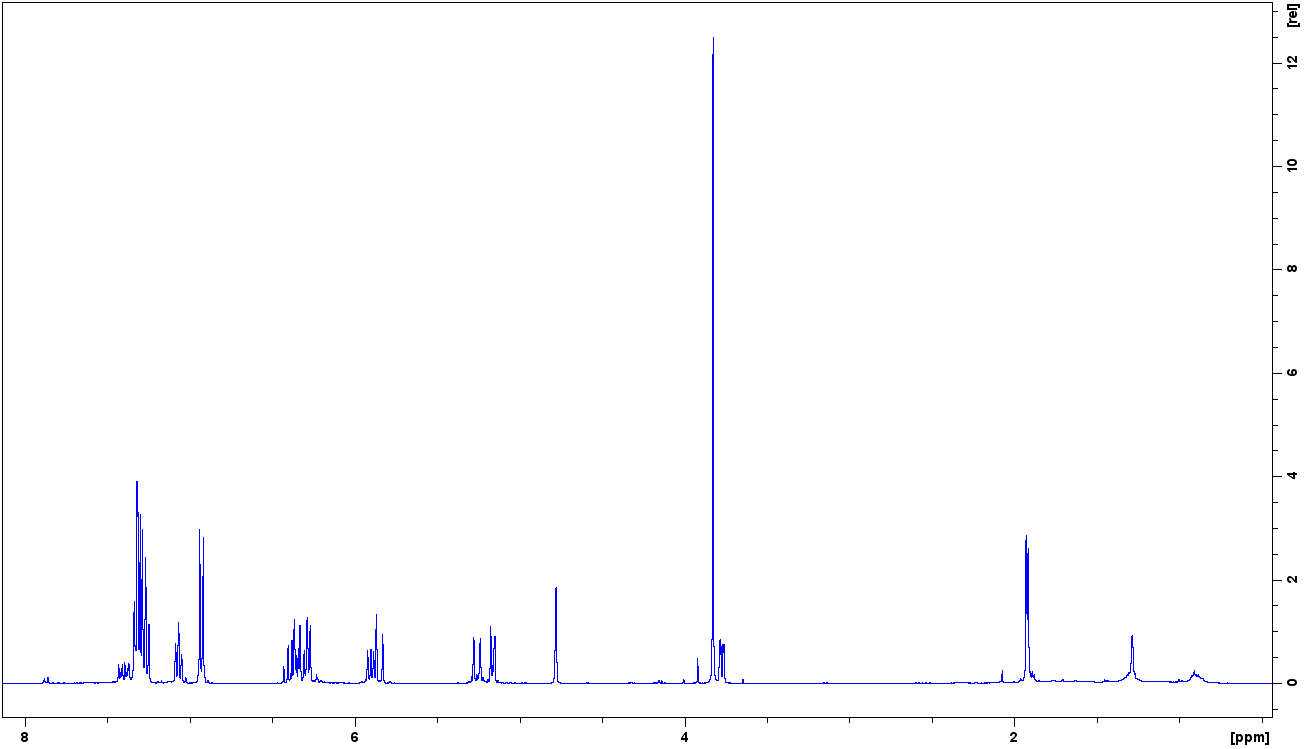

Supplement: Supplementary file 1 [file pharmaceuticals-16-01000-s001.zip › 11s mjm16440_1h.png]

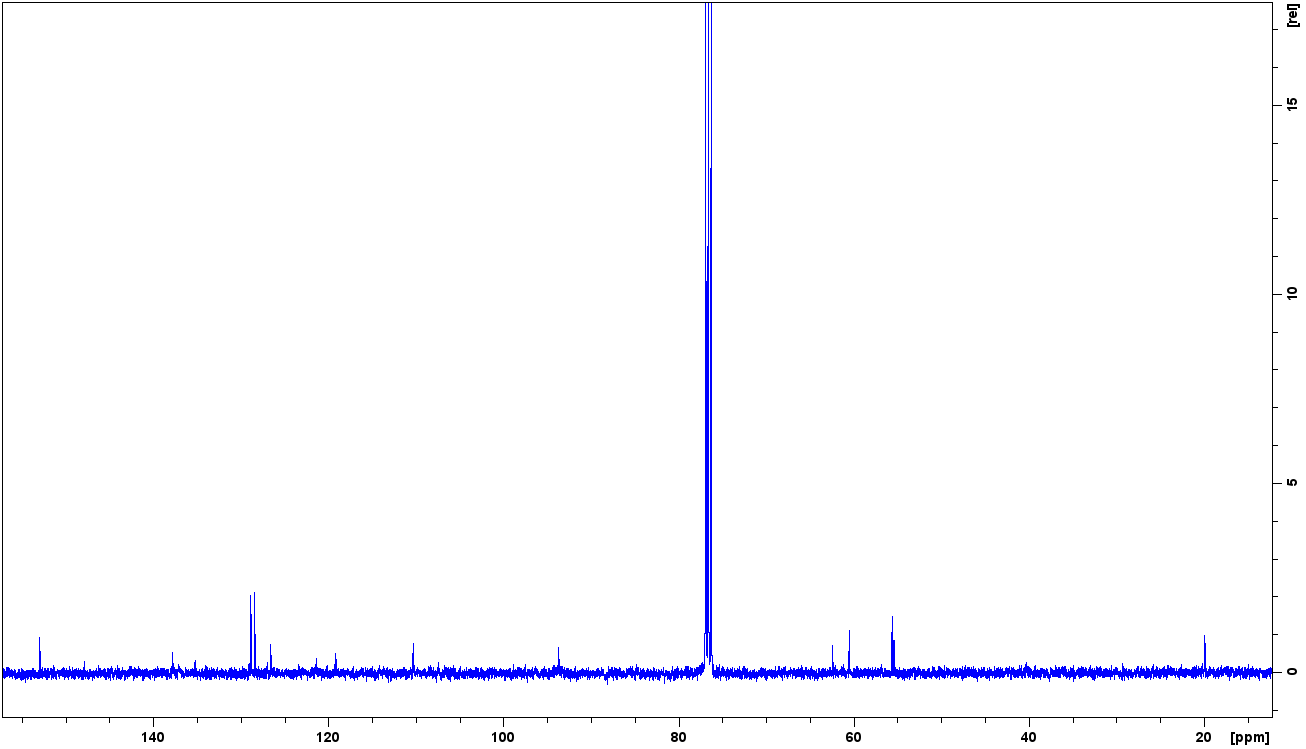

Supplement: Supplementary file 1 [file pharmaceuticals-16-01000-s001.zip › 13a mjm18333_13c.png]

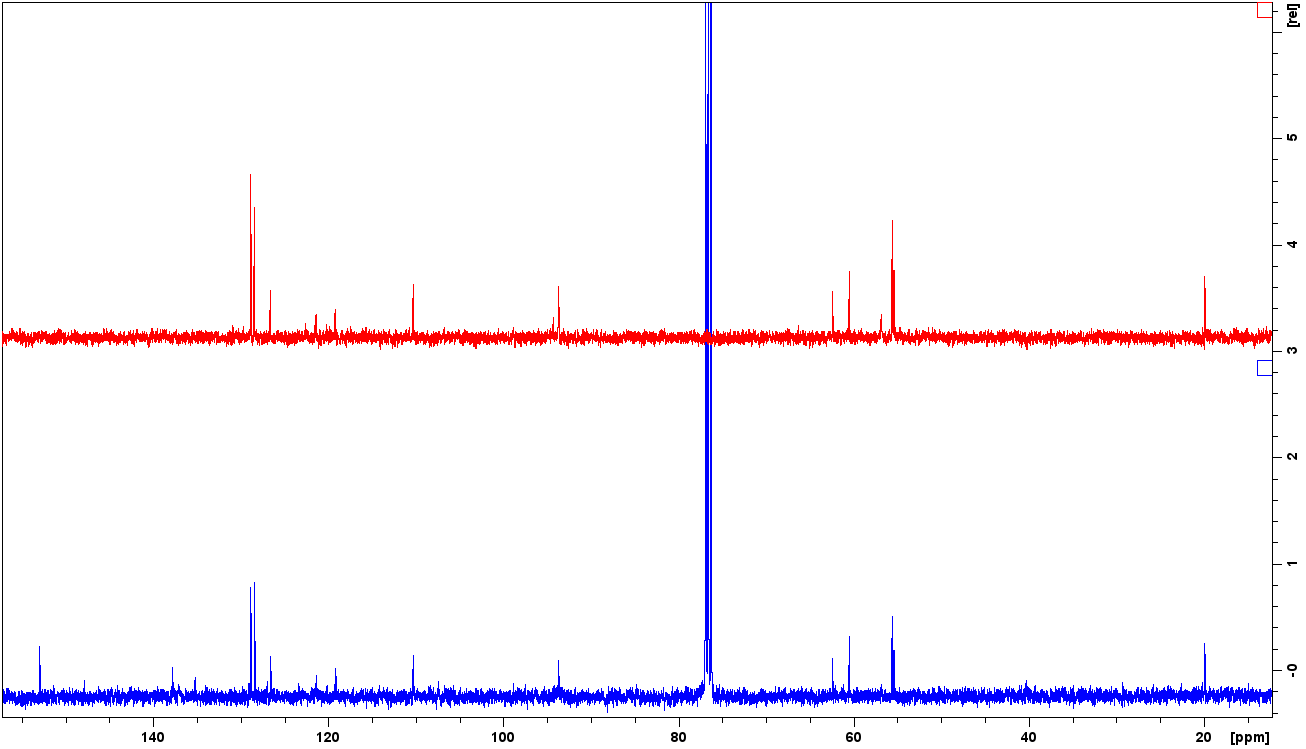

Supplement: Supplementary file 1 [file pharmaceuticals-16-01000-s001.zip › 13a mjm18333_13c_DEPT.png]

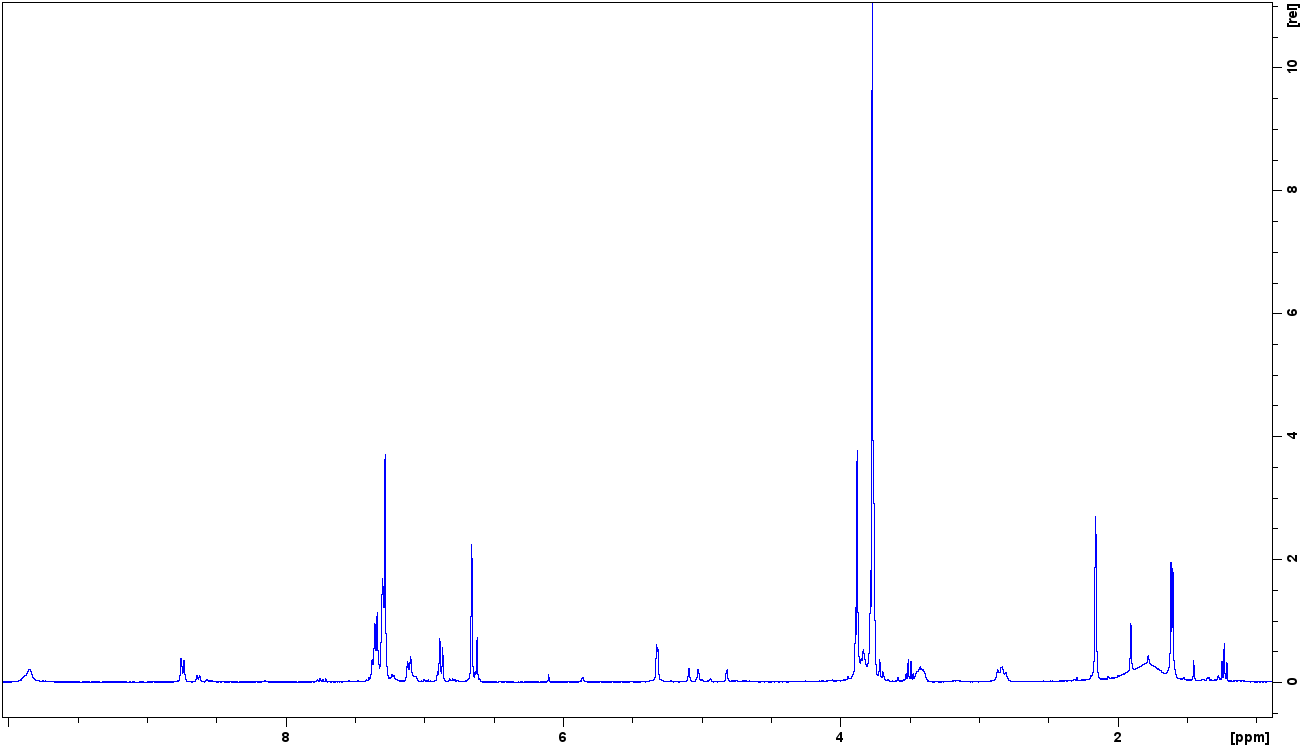

Supplement: Supplementary file 1 [file pharmaceuticals-16-01000-s001.zip › 13a mjm18333_1h.png]

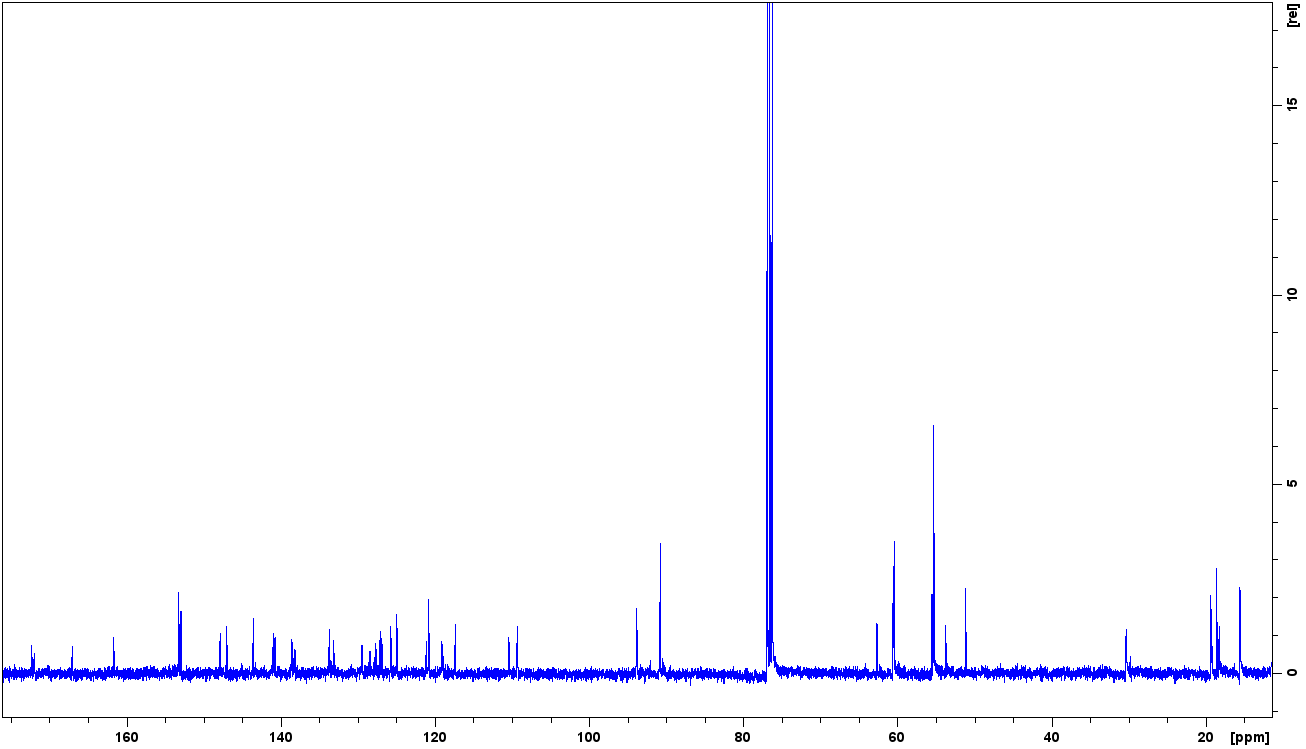

Supplement: Supplementary file 1 [file pharmaceuticals-16-01000-s001.zip › 13d mjm18344_13c.png]

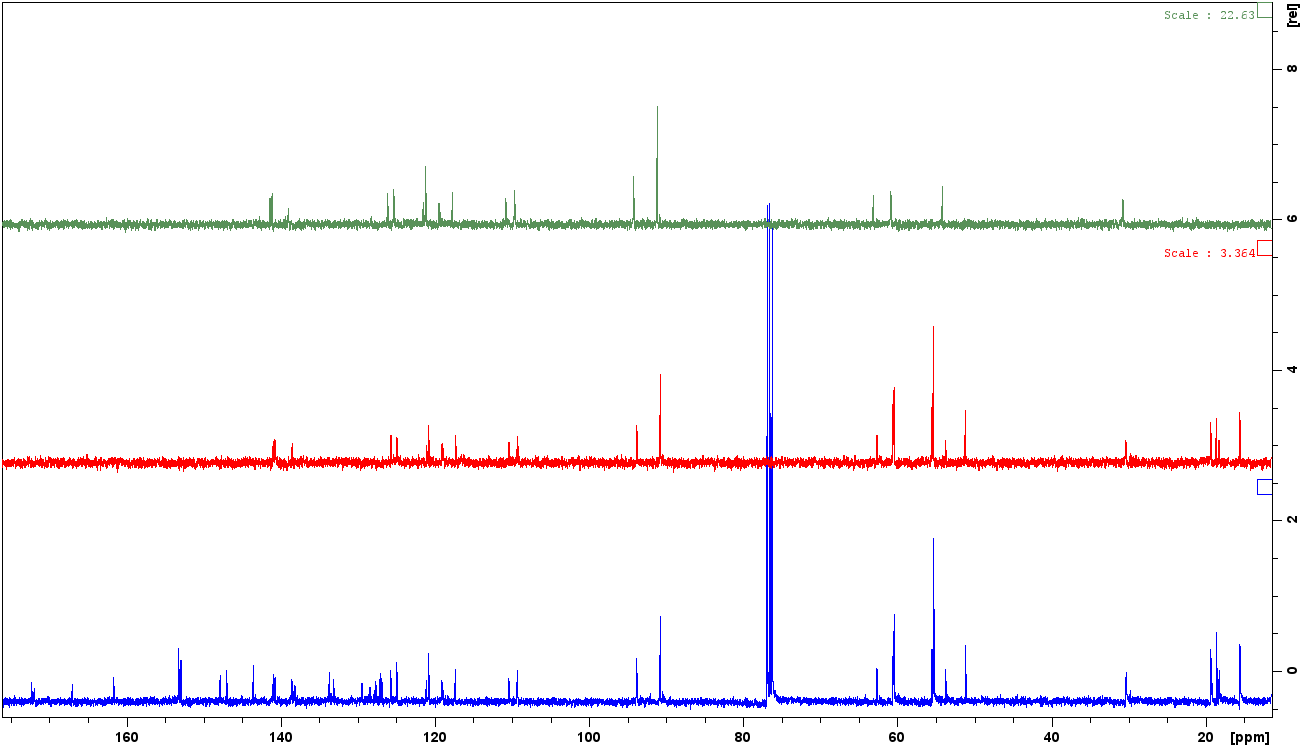

Supplement: Supplementary file 1 [file pharmaceuticals-16-01000-s001.zip › 13d mjm18344_13c_DEPTs.png]

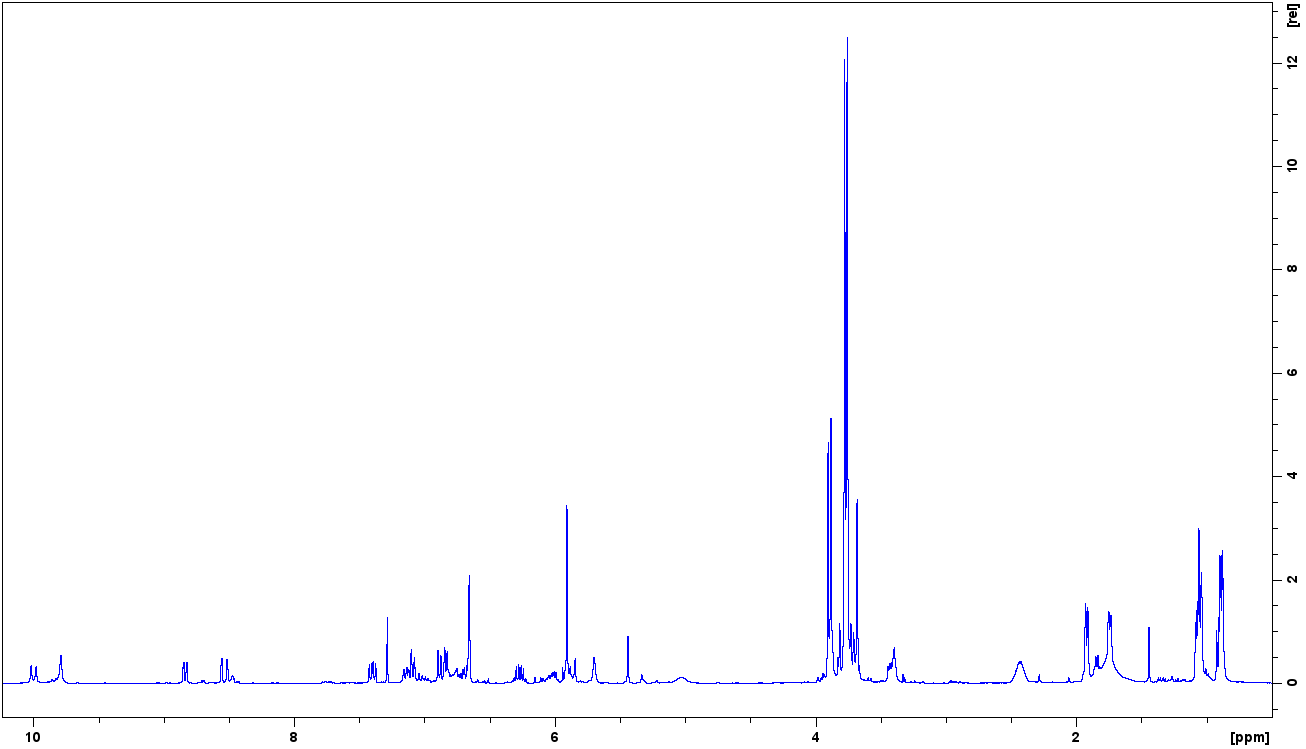

Supplement: Supplementary file 1 [file pharmaceuticals-16-01000-s001.zip › 13d mjm18344_1h.png]

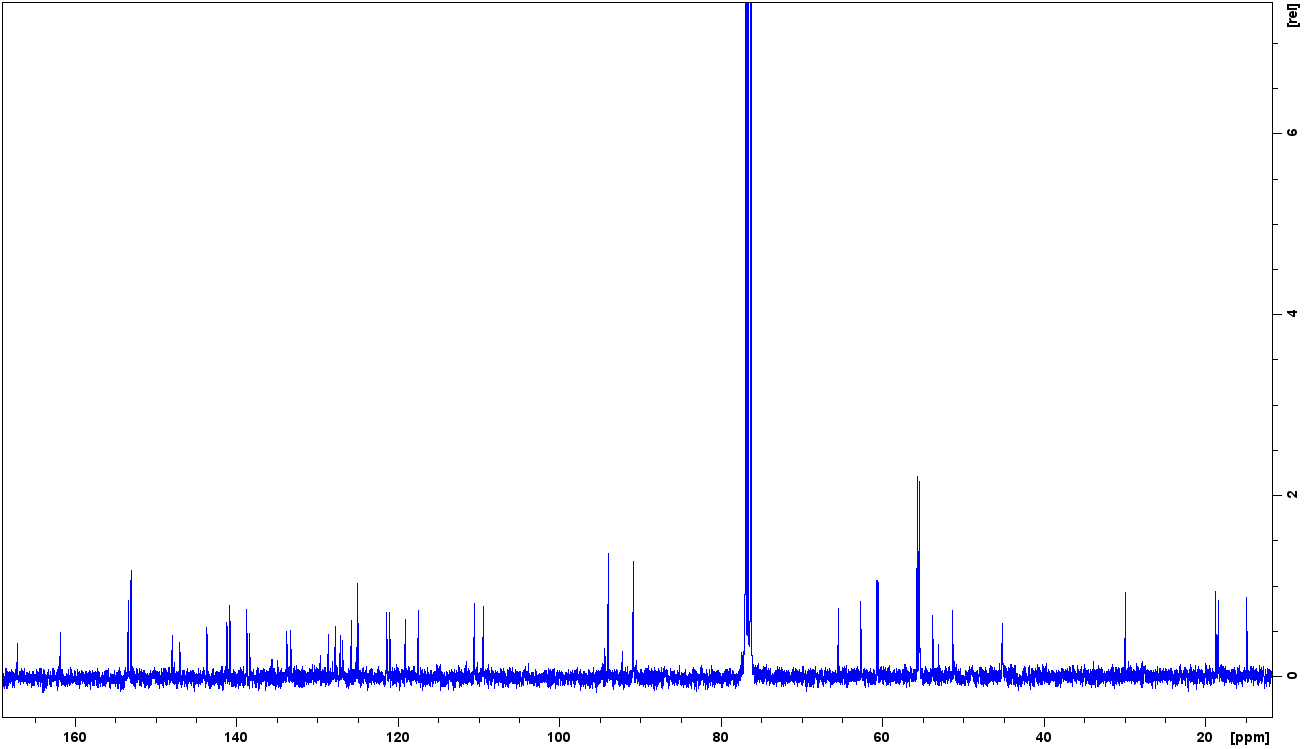

Supplement: Supplementary file 1 [file pharmaceuticals-16-01000-s001.zip › 13e mjm18369_13c.png]

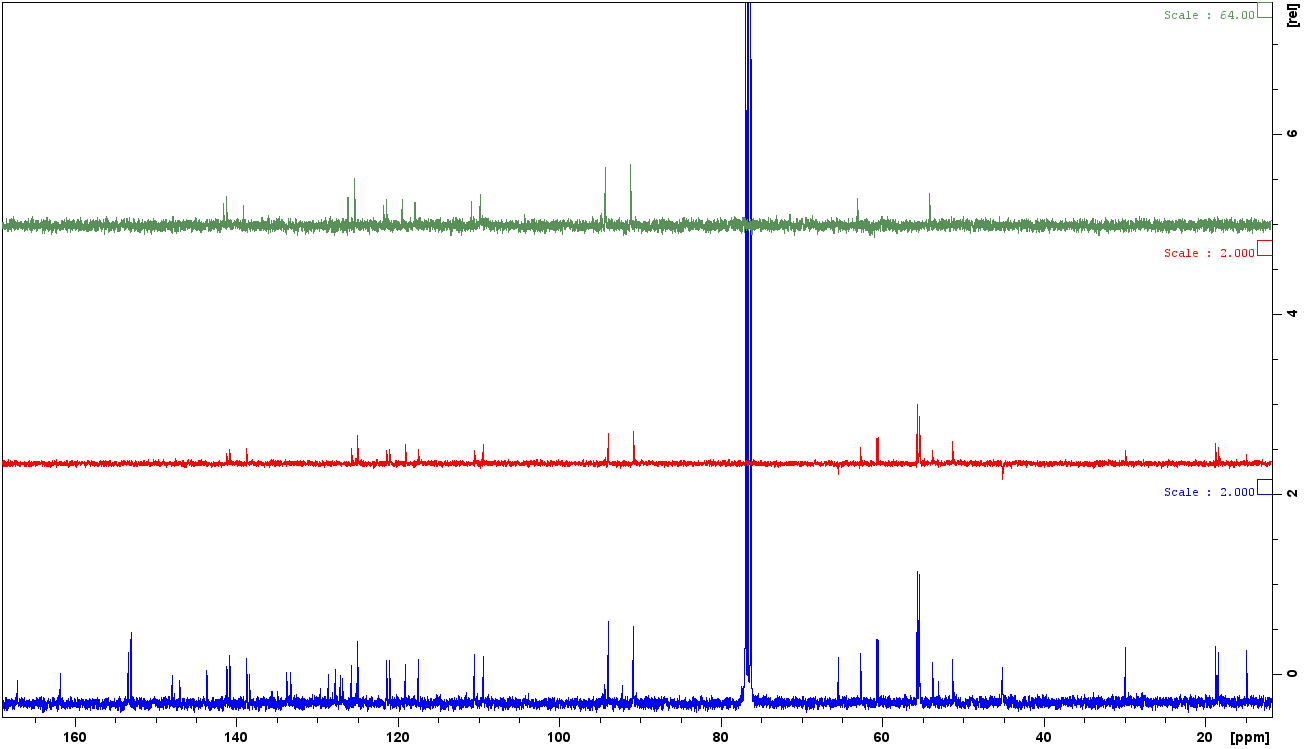

Supplement: Supplementary file 1 [file pharmaceuticals-16-01000-s001.zip › 13e mjm18369_13c_DEPTs.png]

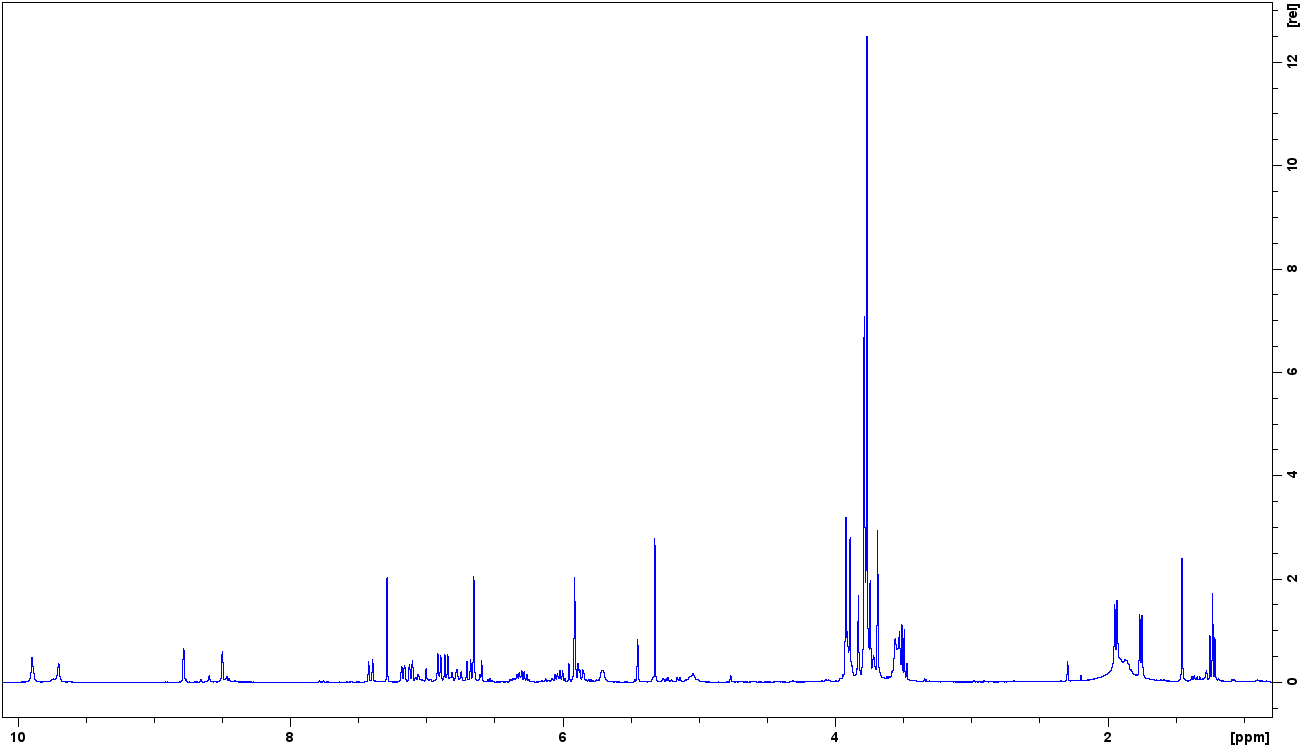

Supplement: Supplementary file 1 [file pharmaceuticals-16-01000-s001.zip › 13e mjm18369_1h.png]

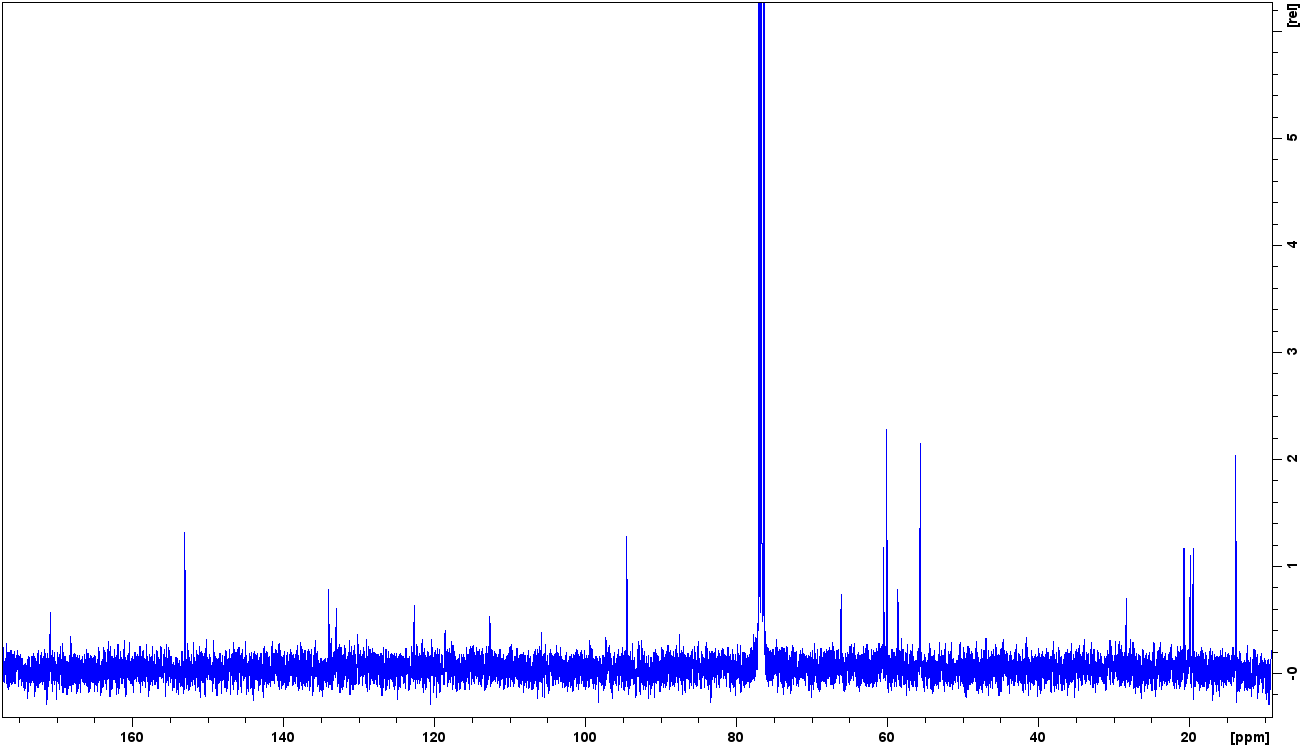

Supplement: Supplementary file 1 [file pharmaceuticals-16-01000-s001.zip › 15b mjm17973_13c.png]

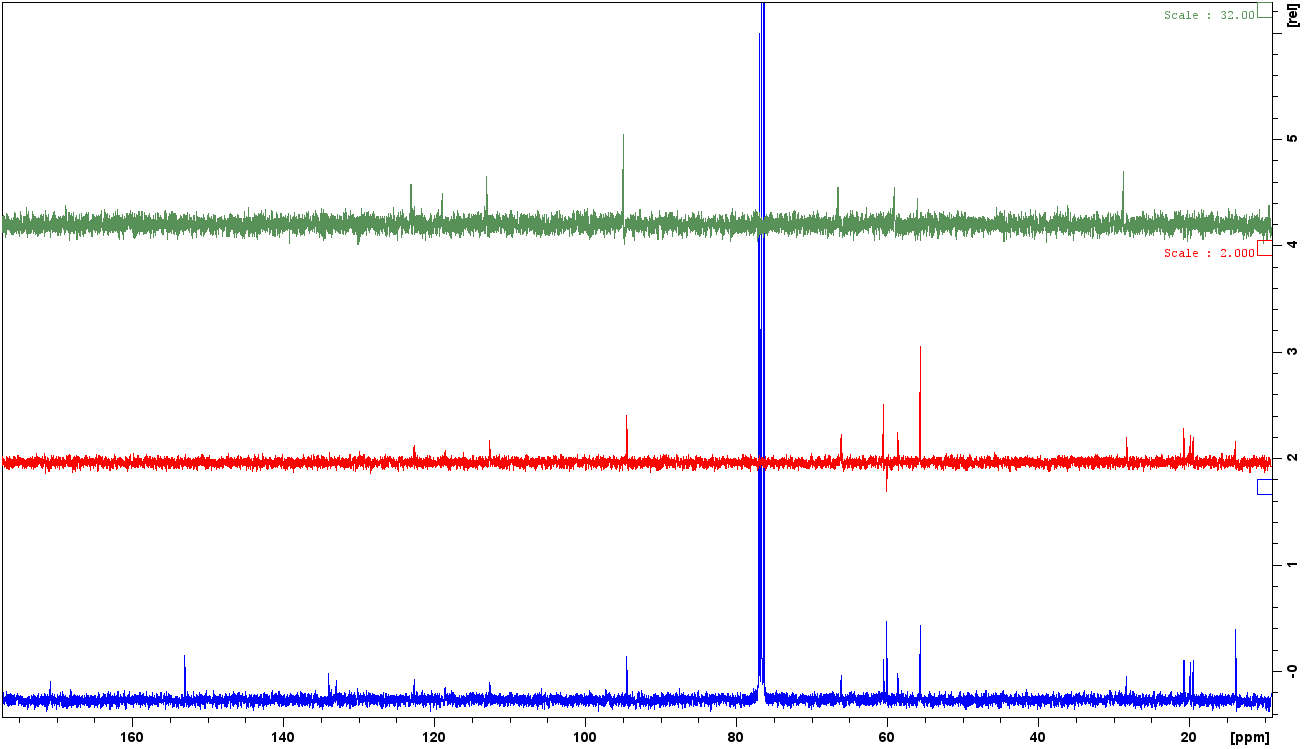

Supplement: Supplementary file 1 [file pharmaceuticals-16-01000-s001.zip › 15b mjm17973_13c_DEPTS.png]

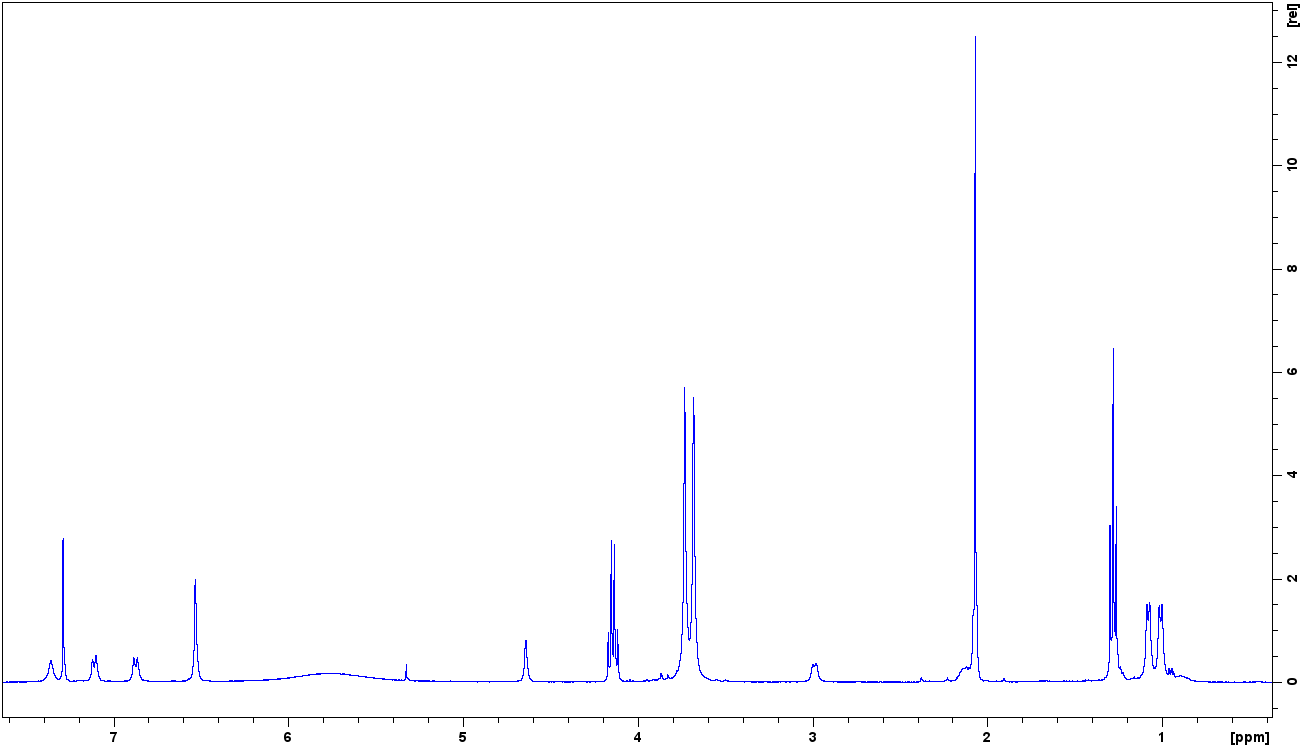

Supplement: Supplementary file 1 [file pharmaceuticals-16-01000-s001.zip › 15b mjm17973_1h.png]

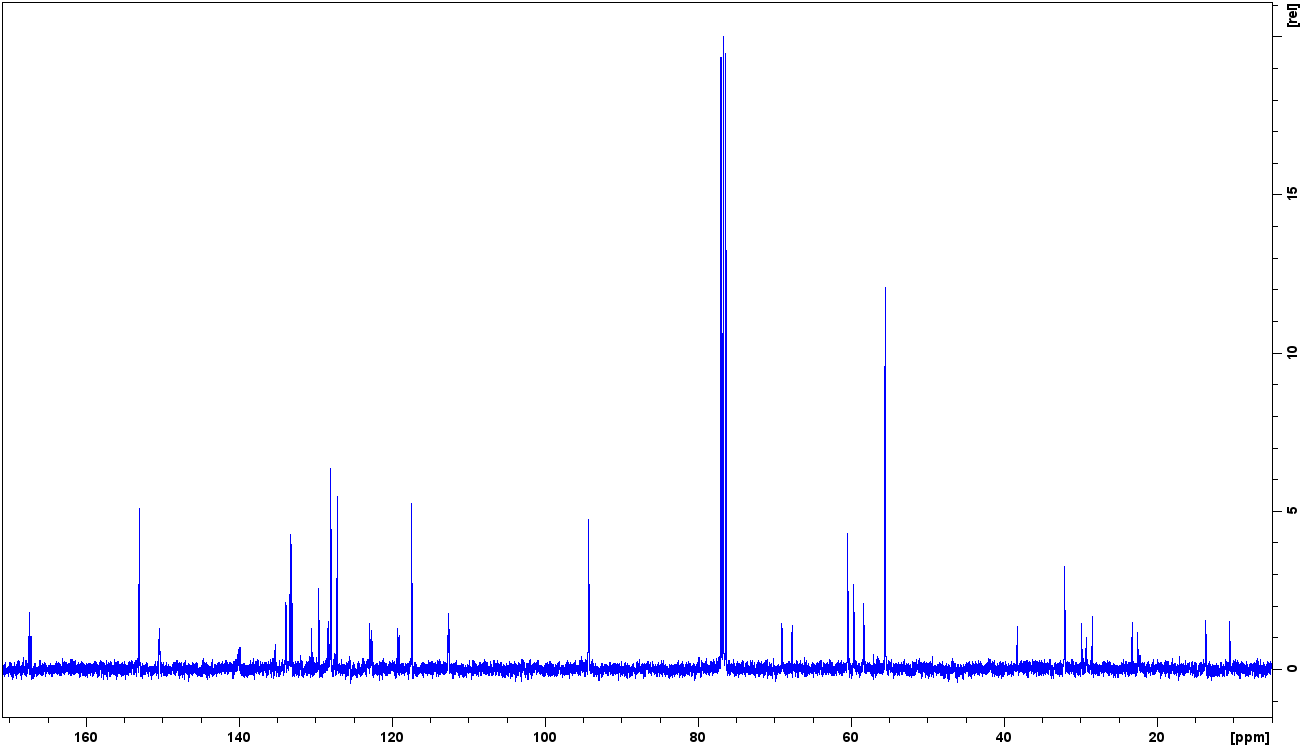

Supplement: Supplementary file 1 [file pharmaceuticals-16-01000-s001.zip › 15c mjm18050_13c.png]

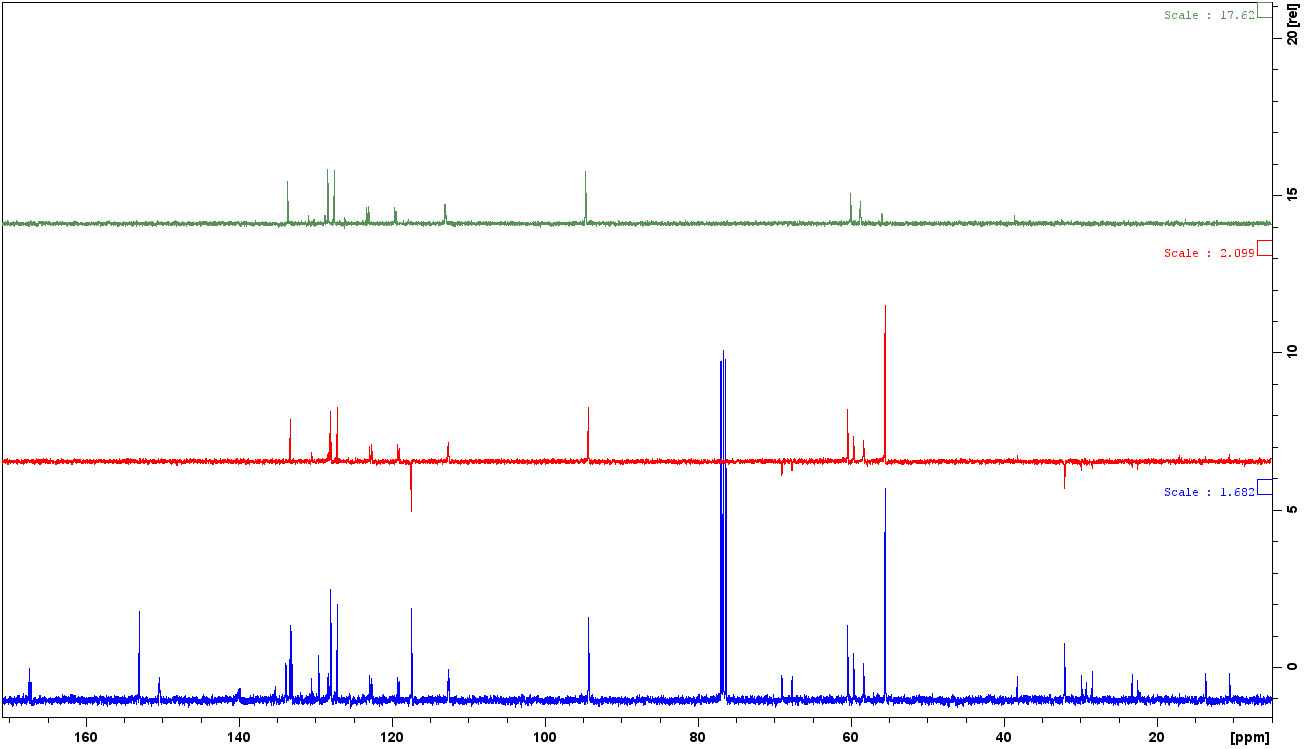

Supplement: Supplementary file 1 [file pharmaceuticals-16-01000-s001.zip › 15c mjm18050_13c_DEPTs.png]

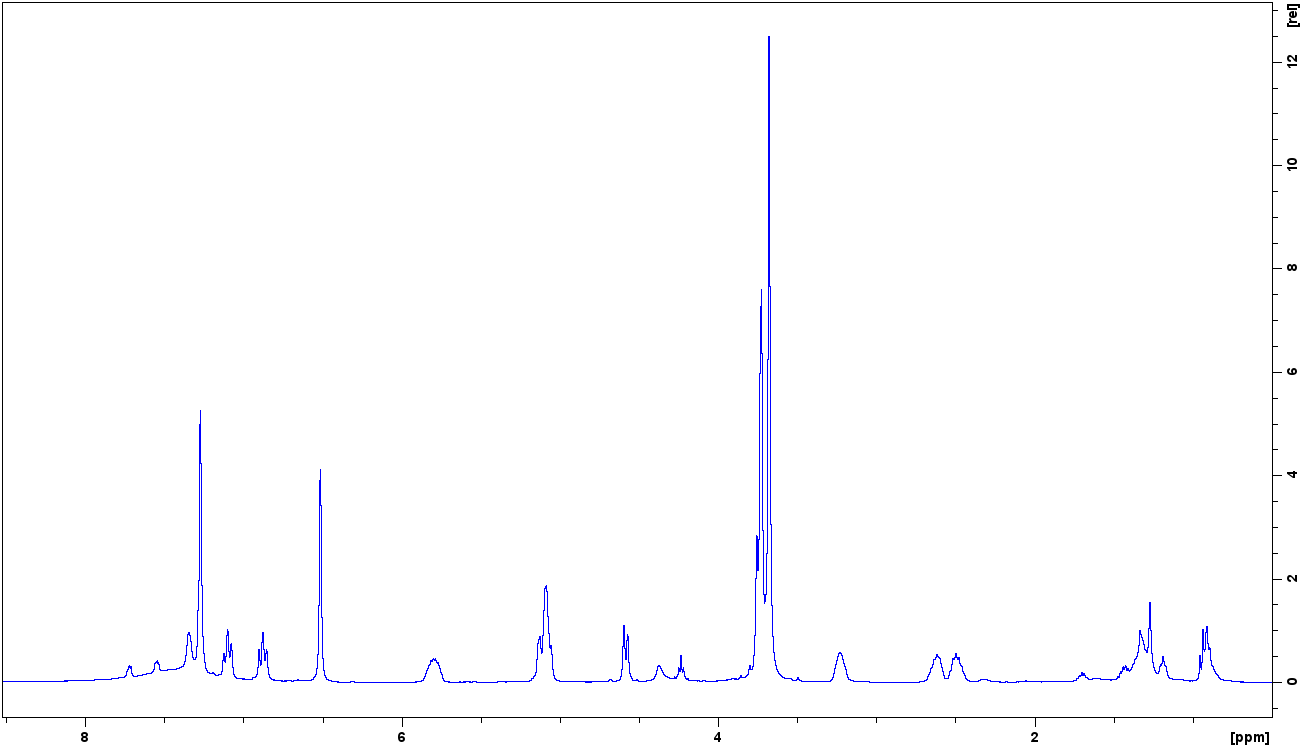

Supplement: Supplementary file 1 [file pharmaceuticals-16-01000-s001.zip › 15c mjm18050_1h.png]
